# Integrative transcriptomics and peptidomics approach reveals unexpectedly diverse endogenous secretory peptides in *Odorrana grahami* frog skin

**DOI:** 10.1186/s12915-025-02463-w

**Published:** 2025-11-28

**Authors:** Jiongyu Liu, Wei Zhu, Liming Chang, Tian Zhao, Meihua Zhang, Bing Wang, Feng Xie, Jianping Jiang

**Affiliations:** 1 Mountain Ecological Restoration and Biodiversity Conservation Key Laboratory of Sichuan Province, Chengdu Institute of Biology, Chinese Academy of Sciences, Chengdu, 610213 China; 2 China-Croatia Belt and Road Joint Laboratory On Biodiversity and Ecosystem Services, Chengdu Institute of Biology, Chinese Academy of Sciences, Chengdu, 610213 China; 3 University of Chinese Academy of Sciences, Beijing, 100049 China

**Keywords:** Endogenous secretory peptide, Signal peptide, ORF_shift protein, Transcriptome, Peptidome

## Abstract

**Background:**

Endogenous secretory peptides (ESPs) play crucial roles in amphibian skin, yet their identification remains challenging in species lacking genomic data. This study developed an innovative pipeline integrating transcriptomics and peptidomics to identify ESPs in the skin of *Odorrana grahami*. This approach enhances endogenous secretory protein prediction accuracy by rescuing candidates erroneously discarded during SignalP-based screening. Such false negatives typically result from inaccurate annotation of N-terminal start sites within 5′-UTRs by protein prediction tools like TransDecoder.

**Results:**

Our approach enhanced potential endogenous secretory protein identification rates by 61.6%, discovering 107 putative ESPs (16 validated at the protein level). Among these, 74 ESPs are newly reported in *O. grahami* (including 62 novel peptides). These ESPs span 14 known families (11 newly reported in *O. grahami*, 8 of which are first reported within the genus *Odorrana*). The frog skin active peptide (FSAP) family (*n* = 83)—comprising the largest subset of ESPs identified in this study—showed unexpected diversity between our studied individual and previously reported individuals within the population. Collectively, *O. grahami* (*n* = 226) and *Odorrana andersonii* (*n* = 205) currently hold the highest documented counts of FSAP family peptides in amphibians. Phylogenetic analysis delineated five FSAP clades (A–E) containing 18 clustered groups, with the hypervariable clade D harboring diverse non-AMPs and tachykinin-convergent peptides. GO and KEGG pathway analyses indicated that ESPs in *O. grahami* skin are predominantly immunity-related.

**Conclusions:**

This study highlights underestimated FSAP family peptide diversity in *Odorrana* and provides an adaptable framework for ESP discovery across taxa.

**Supplementary Information:**

The online version contains supplementary material available at 10.1186/s12915-025-02463-w.

## Background

Amphibian skin, as a mucosal surface, is integral to a multitude of physiological processes including water uptake, ion transport, respiration, heat transfer, camouflage, and predator deterrence. It also plays a pivotal role in defense mechanisms, acting as a complex immune organ that provides multiple barriers—physical, chemical, immunological, and microbiological—against pathogens [1]. Biologically active peptides, particularly host defense peptides (HDPs) primarily composed of antimicrobial peptides (AMPs) in conjunction with other bioactive peptides, serve as the principal barrier. Most natural bioactive peptides which have presently been described are endogenous secretory peptides (ESPs) [2–4]. Representing a specialized subclass of endogenous secretory proteins, these gene-encoded peptides range from 3 to 100 amino acids (aa) in length and are processed from precursor proteins typically containing N-terminal signal peptides. They are activated through multiple steps to function in the extracellular space [5, 6].

Methods for discovering ESPs include the isolation-purification approach and the cDNA-based strategy. The isolation-purification method generally requires multiple specimens to obtain a limited number of mature peptides while providing quantitative data [7]. In contrast, the cDNA method employs degenerate primers targeting conserved sequences (e.g., those adjacent to the signal peptide’s start codon) to screen ESPs from a cDNA library. This widely adopted targeted approach enables the identification of nearly all peptides within a specific ESP family (as classified in UniProt [8]) or DADP clade [2] within the sampled biological material, yielding structural features of precursor proteins and their nucleic acid sequences, albeit lacking quantitative data [9–11]. Due to the low sequence conservation of signal peptide regions across different ESP families, comprehensive detection of ESPs within a single specimen by the cDNA method remains challenging. For instance, the diskless-fingered odorous frog *Odorrana grahami* (Boulenger, 1917) exhibits a remarkable repertoire of 178 skin ESPs (see Additional file 1: Table S1) [7, 9, 11–27], yet these are confined to merely three families as per UniProt classification criteria [8, 28]. Specifically, 171 peptides are classified into the frog skin active peptide (FSAP) family (based on conserved signal peptide and propeptide domains PF03032 or IPR004275 in InterPro, corresponding to the class-1 clade in DADP, combined with mature peptide similarity), which is unique to anuran species (UniProt query: family “FSAP family”). The remaining seven peptides are distributed across two additional families: two (ranamargarin and tachykinin-OG1) belong to the tachykinin family, and five (odorranain-BLP-1 to 5) to the bombesin/neuromedin-B/ranatensin family. This apparent paradox—high peptide multiplicity but low family diversity—highlights a critical gap: while amphibians as a class harbor diverse ESP families, species other than those with sequenced genomes (e.g., *Xenopus* spp. and *Lithobates catesbeianus*) exhibit reported constraints on ESP family diversification at the species level, especially in families showing homology to mammalian counterparts [3, 4, 29, 30].

Untargeted high-throughput RNA-Seq is an effective method for screening peptide sequences. This approach involves using transcriptome sequences to perform BLAST or HMMER searches [31] or employing deep learning, both based on reference databases to annotate sequences [32]. Recent studies on venomous organisms indicate that, besides primary sequence similarity, utilizing structural features, particularly signal peptides predicted by SignalP, is useful for screening ESPs [33, 34]. However, strategies relying on six-frame translation to generate protein sequence databases significantly expand database size and introduce spurious sequences [35]. Moreover, peptidogenomics, integrated with transcriptome and LC–MS/MS (liquid chromatography-tandem mass spectrometry)-based peptidomics, has recently emerged as a promising approach for deep analysis of endogenous peptides in complex samples. Peptidomic searches are more complex than proteomic analyses due to the requirement for “no-digest” or “non-specific digest” settings in MS/MS database searches, exacerbated by the use of six-frame-derived protein databases [36, 37]. Tools like TransDecoder mitigate this by prioritizing the most likely coding sequences, significantly reducing the size of the protein database and generating a more precise database. However, most protein prediction tools (including TransDecoder) that select the longest open reading frame (ORF) among multiple start codons (ATGs) may erroneously place N-terminal start sites in the 5′-UTRs, leading to artificially elongated sequences, which may lead to misidentification of signal peptides by SignalP.

Over the past several decades, many toxins have been discovered in amphibian skin through traditional methods (isolation-purification or cDNA library screening). However, although modern high-throughput RNA-seq-based toxin discovery has been extensively studied, amphibian-focused applications of this methodology remain scarce [31, 38–41], with only one peptidogenomics study reported [42]. Existing studies often present aligned mature peptide sequences without full structural features from transcriptomes, risking incomplete peptide reporting and hindering data utility for non-experts. Additionally, some reported “peptides” exceed 100 aa, representing proteins rather than true peptides.

**Fig. 1:**
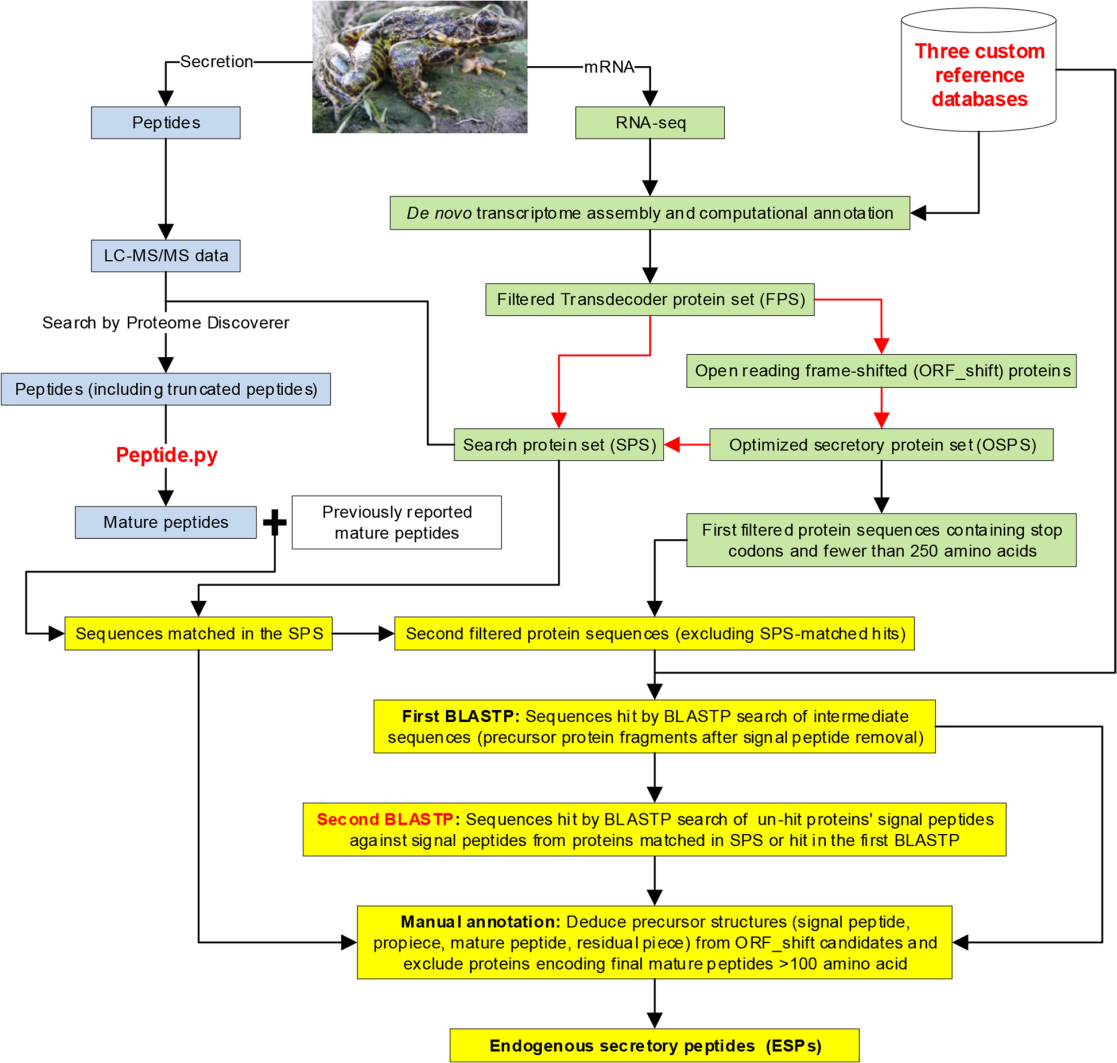
Overview of the integrated pipeline. Green, detection of endogenous secretory proteins at the transcript level following the construction of transcriptome; blue, identification of peptides at the protein level; yellow, peptidome discovery by integrating the data at the protein and transcript levels; red lines, analysis pipeline executed by the ORF_all.sh script; “Sequences matched in the SPS”, full-length query sequences with 100% identity to any contiguous subsequence in SPS

Here, we developed a pipeline integrating transcriptomics and peptidomics-based screenings to identify ESPs from the skin of a single *O. grahami* specimen (Fig. 1). Our analysis offers comprehensive structural features of precursor proteins and their nucleic acids, providing quantitative data at the protein and transcript levels. It addresses knowledge gaps on non-FSAP family peptides in *Odorrana*, reveals the highest FSAP family diversity among amphibians, and demonstrates convergent evolution between two FSAP family peptides and the tachykinin family.

## Results

### Construction of transcriptome

RNA sequencing generated 26.3 million 150 bp paired-end reads (base-calling accuracy 99.96%). Trimmomatic-based quality control retained 25.5 million reads (25–150 bp). FastQ Screen analysis showed minimal alignments (0–0.2%) of the trimmed reads to common contaminant sequences, supporting data reliability (Additional file 2: Fig. S1). De novo assembly using Trinity produced 217,270 transcripts (N50 = 1272 bp, GC = 44.8%). Bowtie2 alignment confirmed 94.6% reads mapped to the transcriptome. BUSCO assessment (eukaryota_odb10) indicated 96.9% complete genes. TransDecoder predicted 181,817 proteins (62.0% of Trinity transcripts). For detailed information, please refer to Section 2.1 of Additional file 2. The transcriptome-related datasets are provided in [43].

### Recognition of endogenous secretory proteins at the transcript level

The filtered TransDecoder protein set (FPS, see the “Methods” section) contained 152,077 sequences, including 6246 endogenous secretory protein sequences (4.1% of FPS). The optimized secretory protein set (OSPS) created through signal peptide prediction on the open reading frame-shifted (ORF_shift) proteins (see the “Methods” section) contained 12,248 endogenous secretory protein sequences, of which 10,092 were non-redundant (i.e., each series of ORF_shift proteins was counted as one). The search protein set (SPS) generated by merging the FPS and the OSPS contained 154,233 sequences, representing an increase of only 2156 protein sequences (1.4%) relative to the FPS. However, the number of non-redundant endogenous secretory protein sequences increased from 6246 to 10,092, indicating a recognition rate increase of 61.6% (Data S1 of [44]).

### Identification of peptides at the protein level

A total of 13,335 precursors were generated from the LC–MS/MS data, of which 867 MS/MS spectra were matched with high confidence to 1054 peptide-spectrum matches (PSMs) (Additional file 2: Fig. S2). A set of peptide isoforms belonging to 21 master proteins (longest sequence isoforms selected by PD’s parsimony algorithm within peptide-equivalent protein groups) were identified by Proteome Discoverer (PD), including 817 unambiguous PSMs from 980 high-confidence PSMs (Additional file 3, Data S2 of [44]). We finally obtained 16 FSAP family mature peptides with their master proteins, including the novel odorranain-C13 and ranatuerin-2P-OG1, accompanied by 126 truncated peptides derived from the same set. Each mature peptide exhibits higher abundance and greater length compared to its respective truncated peptides (Table 1; Additional file 2: Fig. S3; Additional file 4). Notably, none of the peptides we detected belonged to the signal peptide or acidic peptide regions of the master protein. This finding suggests that FSAP family peptides either lack N-terminally extended forms or have them in very low abundance, unlike insect toxins, which are typically detected with such extensions [45]. The raw LC–MS/MS data is provided in [43].

**Table 1:** Identification of mature peptides via LC–MS/MS

Peptide names	Mature peptides	Master proteins	#Truncated peptides
brevinin-1E-OG3	FLPLLAGLAANFLPKLFCKITKKC	TRINITY_DN0_c1_g1_i24.p1	7
brevinin-2E-OG1	GLLDTFKNMALNAAKSAGVSVLNALSCKLSKTC	TRINITY_DN440_c40_g1_i1.p1	13
brevinin-2GRa	GLLDTFKNLALNAAKSAGVSVLNSLSCKLSKTC	TRINITY_DN0_c1_g1_i14.p1,TRINITY_DN0_c1_g1_i4.p1	28
brevinin-2GRb	GVLGTVKNLLIGAGKSAAQSVLKTLSCKLSNDC	TRINITY_DN0_c1_g1_i11.p1	17
esculentin-1-OG5	GLFSKFAGKGIKDLIFKGVKHIGKEVGMDVIRTGIDVAGCKIKGEC	TRINITY_DN0_c1_g1_i16.p1	9
esculentin-2-OG10	GLFTLIKGAAKLIGKTVAKEAGKTGLELMACKITNQC	TRINITY_DN0_c1_g1_i22.p1	26
esculentin-2-OG8	GIFSILKIATKLIGKTLAKAAGKAGAELAACKAANQC	TRINITY_DN96_c0_g2_i2.p1	1
esculentin-2-RA1	GIFAILKIATKLIGKTLAKAAGKAGTGLLACKAAKEC	TRINITY_DN96_c0_g1_i2.p1	1
nigrocin-2GRa	GLLSGILGAGKHIVCGLSGLC	TRINITY_DN77_c0_g1_i1.p1_ORF1	3
nigrocin-2GRb	GLFGKILGVGKKVLCGLSGMC	TRINITY_DN49_c0_g1_i1.p1_ORF1	6
nigrocin-2GRc	GLLSGILGAGKNIVCGLSGLC	TRINITY_DN0_c1_g1_i17.p1	1
odorranain-C13	GVLGTVKNLLIGASKSAAQSVLKTLSCKLSNDC	TRINITY_DN2658_c0_g2_i1.p1	10
odorranain-F2	GFMDTAKNVAKNVAVTLLDNLKCKITKAC	TRINITY_DN10285_c0_g1_i1.p1	3
odorranain-P1b	VIPFVASVAAEMMQHVYCAASKKC	TRINITY_DN5345_c0_g1_i2.p1	0
OGTI	AVNIPFKVHFRCKAAFC	TRINITY_DN0_c1_g1_i20.p1	0
ranatuerin-2P-OG1	GLMNTVLNVLTNVAGTVKDKIKCKFTGGC	TRINITY_DN6_c2_g1_i1.p1	1

All mature peptides identified in this study belong to the FSAP family, among which odorranain-C13 and ranatuerin-2P-OG1 are novel (Additional file 1: Table S2). Sequence mappings of mature peptides and their truncations (all detected by mass spectrometry) onto corresponding master proteins are provided in Additional file 2: Fig. S3a (exemplified by brevinin-2GRa) and in Additional file 4 (other peptides). Representative annotated MS/MS spectra at the peptide group level for brevinin-2GRa and its truncations are detailed in Fig. S3b. The native Proteome Discoverer result file (SPS.pdResult) is provided in Data S2 of [44]. Protein and peptide isoforms identified from this file, along with annotated MS/MS spectra at the PSM level for the 16 ESPs and their truncations, are provided in Additional file 3 (SPS folder) as exported datasets: Search_file_export.xlsx (tabular data) and Annotated_Spectra.zip (spectral annotations)

### Discovery of ESPs via integrated transcriptomics and peptidomics

#### Families and structural features of ESPs

**Fig. 2:**
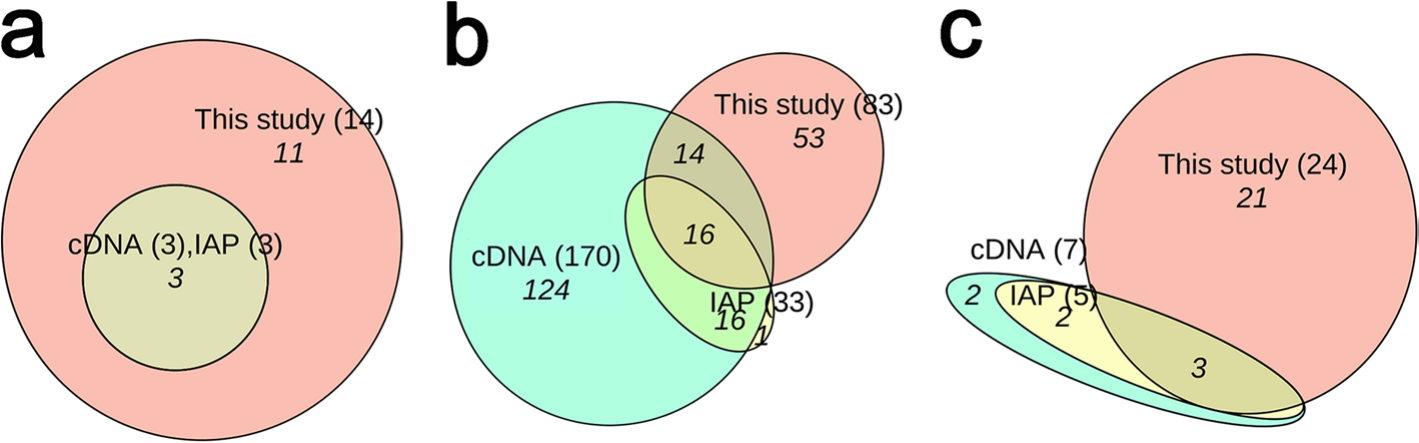
ESPs identified by different methods from *O. grahami*. **a** ESP families. **b** FSAP family peptides. **c** non-FSAP family peptides. IAP refers to the isolation-purification methods, and cDNA refers to the cDNA methods (Additional file 1: Tables S1–S2). This Euler diagram was created online by EVenn [51]

Through the integration of transcriptomic and peptidomic analyses, we discovered 107 putative ESPs, 16 of which were validated at the protein level by LC–MS/MS (see Additional file 1: Table S2) [7, 9, 11, 12, 15–19, 21, 23, 25, 26, 46–50]. These peptides corresponded to 133 precursor proteins (Additional file 1: Table S3) and 138 transcripts (Additional file 1: Table S4), and were classified into 14 known families. In these tables, the OG in the “Source” column refers to peptides previously reported in *O. grahami*, OD refers to peptides reported in other species of the genus *Odorrana*, and OF refers to peptides reported in non-*Odorrana* species of the order Anura. The suffix “P” indicates similar but not identical novel peptides, while Orphan refers to novel peptides with no similarity to any known peptides. Therefore, OGP/OD/ODP/OF/OFP/Orphan represent newly identified peptides in *O. grahami*, whereas OGP/ODP/OFP/Orphan are considered novel peptides. Among the 14 families, only three—FSAP, tachykinin, and bombesin/neuromedin-B/ranatensin—contained members previously reported in *O. grahami* (Table 2, Fig. 2a). Notably, eight of these families—GnRH, amotoxin, NPY, 7B2, galanin, vasopressin/oxytocin, insulin, and intercrine alpha (chemokine CxC)—were reported for the first time in the genus *Odorrana*. Additionally, these families, along with the calcitonin, cathelicidin, and FARP (FMRFamide-related peptide) families, were also reported for the first time in *O. grahami*. Out of the 107 ESPs, 33 had been previously reported in *O. grahami* and 74 were newly reported, of which 62 were novel peptides. All these peptides share a similar structural motif consisting of a signal peptide → optional propiece → enzyme cleavage site → mature peptide → optional enzyme cleavage site → optional residual piece → stop codon. In four families, such as the GnRH, 7B2, vasopressin/oxytocin, and FARP families, the mature peptide section contains 2 to 3 final mature peptides (Additional file 1: Tables S2–S3).

**Table 2:**
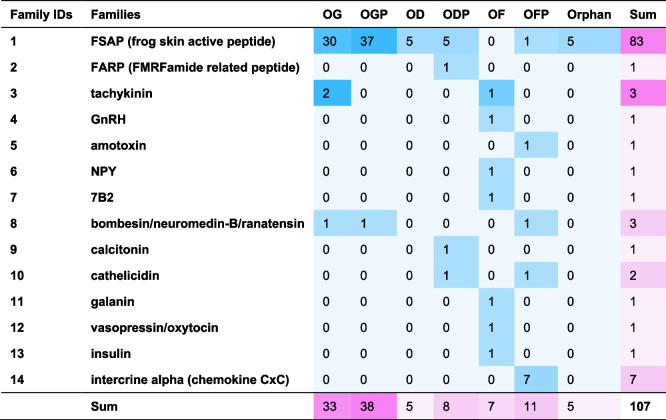
Families of ESPs

OG refers to peptides previously reported in* O. grahami*, OD refers to peptides reported in other species of the genus *Odorrana*, and OF refers to peptides reported in non-*Odorrana* species of the order Anura. The suffix “P” indicates similar but not identical novel peptides, while Orphan refers to novel peptides with no similarity to any known peptides. Therefore, OGP/OD/ODP/OF/OFP/Orphan represent newly identified peptides in *O. grahami*, whereas OGP/ODP/OFP/Orphan are considered novel peptides (Additional file 1: Tables S2–S4)

Consistent with prior research, the majority of ESPs found in the skin of this specimen also belong to the FSAP family, comprising a total of 83 peptides (77.6% of the total) (Additional file 1: Table S2). These peptides corresponded to 101 precursor proteins (Additional file 1: Table S3) and 102 transcripts (Additional file 1: Table S4). Among these peptides, 78 were classified into 31 known subfamilies based on traditional classification, whereas the remaining five were peptides without any sequence similarity. These Orphan peptides were categorized into five new subfamilies, namely odorranain-X1 to X5. Of the 83 peptides, 30 had been previously reported in *O. grahami* and 53 were newly identified, in which 48 were novel peptides (Table 3, Fig. 2b). All these peptides share a conserved structural motif consisting of a signal peptide → acidic propiece → enzyme cleavage site at the C-terminal side of X-Arg (X is usually Lys) → mature peptide → stop codon. Interestingly, an opioid peptide named odorranaopin was only previously detected in the brain [21].

**Table 3:**
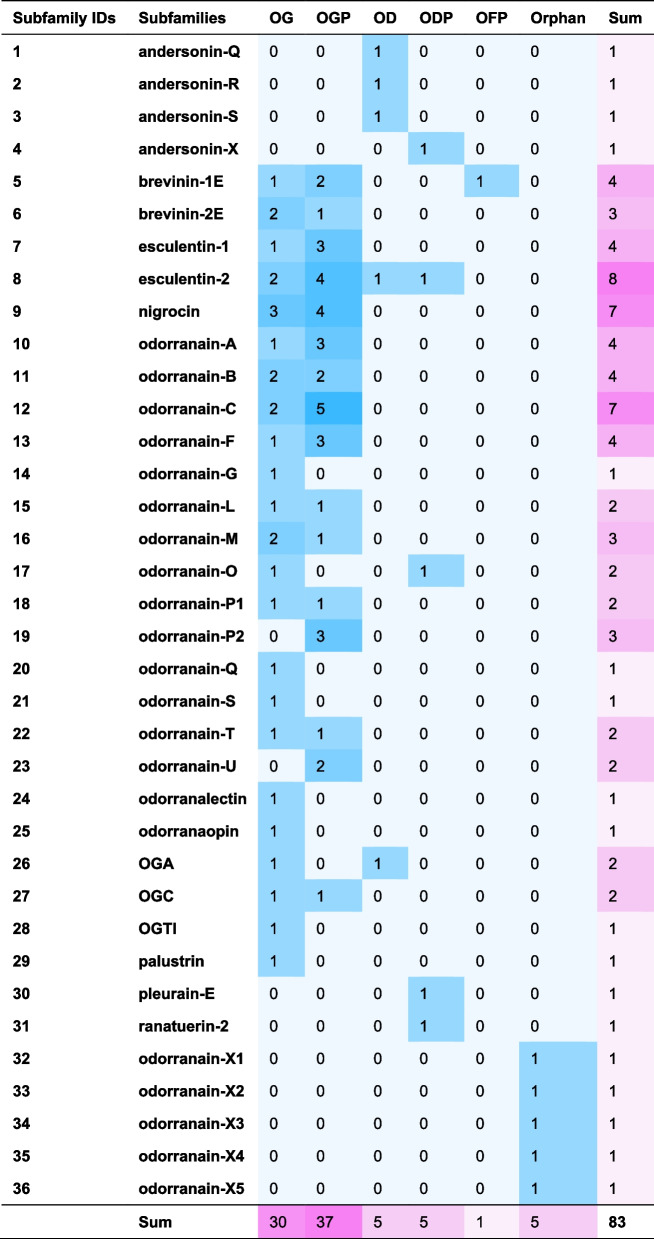
FSAP family peptides

This table has the same notes as Table 2

The remaining 13 families of ESPs comprised a total of 24 peptides (Table 2; Additional file 1: Table S2). These peptides corresponded to 32 precursor proteins (Additional file 1: Table S3) and 36 transcripts (Additional file 1: Table S4). Among these peptides, three had been previously reported in *O. grahami* and 21 were newly identified (Fig. 2c), in which 14 were novel peptides. Detailed structural features of the remaining 13 families of ESPs are shown in Figs. S4–S16 and Section 2.2 in Additional file 2 [12, 25, 47–49, 52–58].

#### Conservation and diversity of ESPs

There are 97 peptides corresponding to 116 precursor proteins and 121 transcripts with complete CDS regions in this study (Additional file 1: Tables S3–S4). The nucleic acid and “translated” amino acid sequences of the signal peptide and upstream 5′-UTR up to 45 nucleotides region show high conservation in the FSAP family, but only low conservation with the same region in the other 13 families. Interestingly, two peptides, tachykinin OG1 and ranamargarin, that traditionally belong to the tachykinin family, show high conservation with the FSAP family in this region, while the remaining peptide, ranatachykinin-A, shows only low similarity (Additional file 5). Although their mature peptide regions are similar to that of ranatachykinin-A, their precursor protein sequences differ greatly (17.0% and 14.3% similarity, respectively, Additional file 2: Fig. S5). Therefore, from now on, we will describe these two peptides as belonging to the FSAP family. Furthermore, the nucleic acid sequences of the FSAP family also show conservation in the acidic propiece region, and the stop codon and downstream 3′-UTR (3′-untranslated region) down to 45 nucleotides region, while the mature peptide region shows a great diversity (Additional file 5). Among these FSAP family precursor proteins, 82 have a signal peptide start site amino acid sequence of Met-Phe-Thr (91.1% of proteins) (Additional file 1: Table S5), 74 signal peptides have a cleavage site of Leu-Cys (82.2% of all proteins) (Additional file 1: Table S6), and 76 mature peptides have an enzyme cleavage site of Lys-Arg (84.4% of all proteins) (Additional file 1: Table S7). These findings indicate that the peptides in the FSAP family are highly conserved at these sites. Notably, the specific proteolytic processing required for the release of mature peptides in amphibians has not yet been experimentally confirmed. The precise cleavage sites are inferred by comparing transcript-derived precursor protein sequences with mature peptide sequences validated at the protein level, and further speculated from mechanisms experimentally established in mammalian systems, highlighting the need for further investigation [30, 59–61]. Although multiple potentially relevant proprotein convertases, such as Furin, PCSK5, PCSK6, and PCSK7 [62], were detected in the transcripts of this skin specimen (the annotation report), their functional roles in peptide maturation remain to be characterized.

**Fig. 3:**
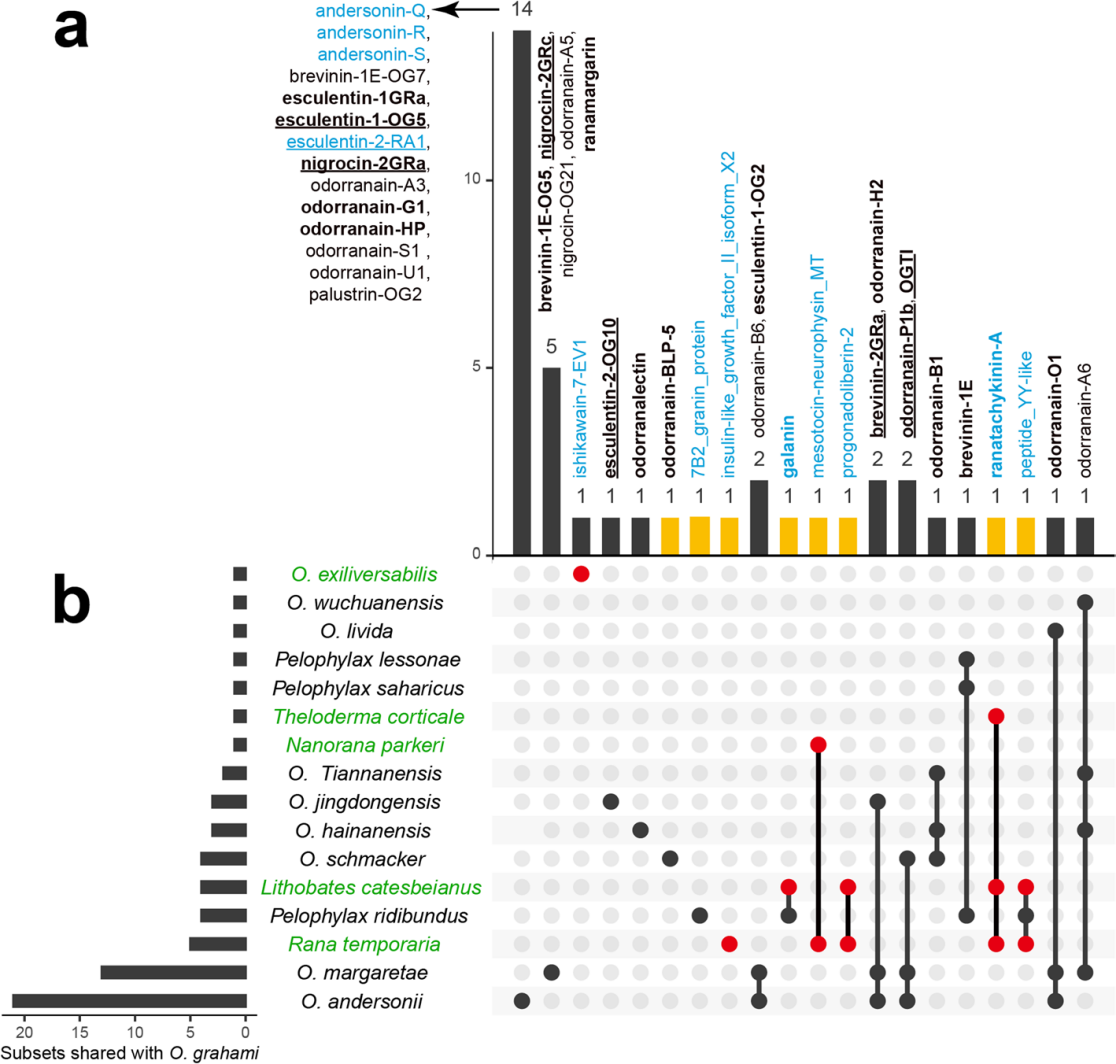
Shared ESPs between *O. grahami* and other amphibian species. Of the 226 FSAP family and 26 non-FSAP family peptides identified in *O. grahami*, including those previously reported and newly discovered in this study, 32 FSAP family (14.2%) and 8 non-FSAP family peptides (30.8%) are shared with 16 other species. **a** Bar chart of shared peptide subsets: FSAP‐family peptides are shown in black; non-FSAP peptides are shown in yellow. Labels show peptide names and the total number per subset; blue text marks peptides newly reported in *O. grahami*, underlined text indicates those validated at the protein level in this study, and bold text denotes those previously reported at the protein level. **b** Network of species sharing ESP subsets with *O. grahami*: Species names in green denote newly discovered sharing partners; red nodes highlight peptides newly identified in *O. grahami* and species with which peptide sharing is newly reported. Lines connect species that share the same peptide subset

In *O. grahami*, 226 putative FSAP family and 26 putative non-FSAP family peptides were identified, including those previously reported and newly discovered in this study (Additional file 1: Tables S1–S2). Together with *O. andersonii* (*n* = 205, Additional file 1: Table S8) [4], these two species currently represent the amphibian taxa with the most extensively documented FSAP family peptides based on existing records. On the other hand, the cross-species distribution of ESPs in *O. grahami* is prominent. Thirty-two FSAP family (14.2%) and eight non-FSAP family peptides (30.8%) are shared across 16 other species (Fig. 3). Among these, *O. andersonii* shares the greatest number of peptides (22), whereas among non-*Odorrana* species, *Rana temporaria* exhibits the highest level of peptide sharing (five peptides). This highlights the conservation of the mature peptide sequences. However, the conservation is less pronounced at the precursor protein sequence, and even less so at the nucleic acid sequence, reflecting the polymorphism of ESPs.

#### Phylogenetic tree of FSAP family peptides

**Fig. 4:**
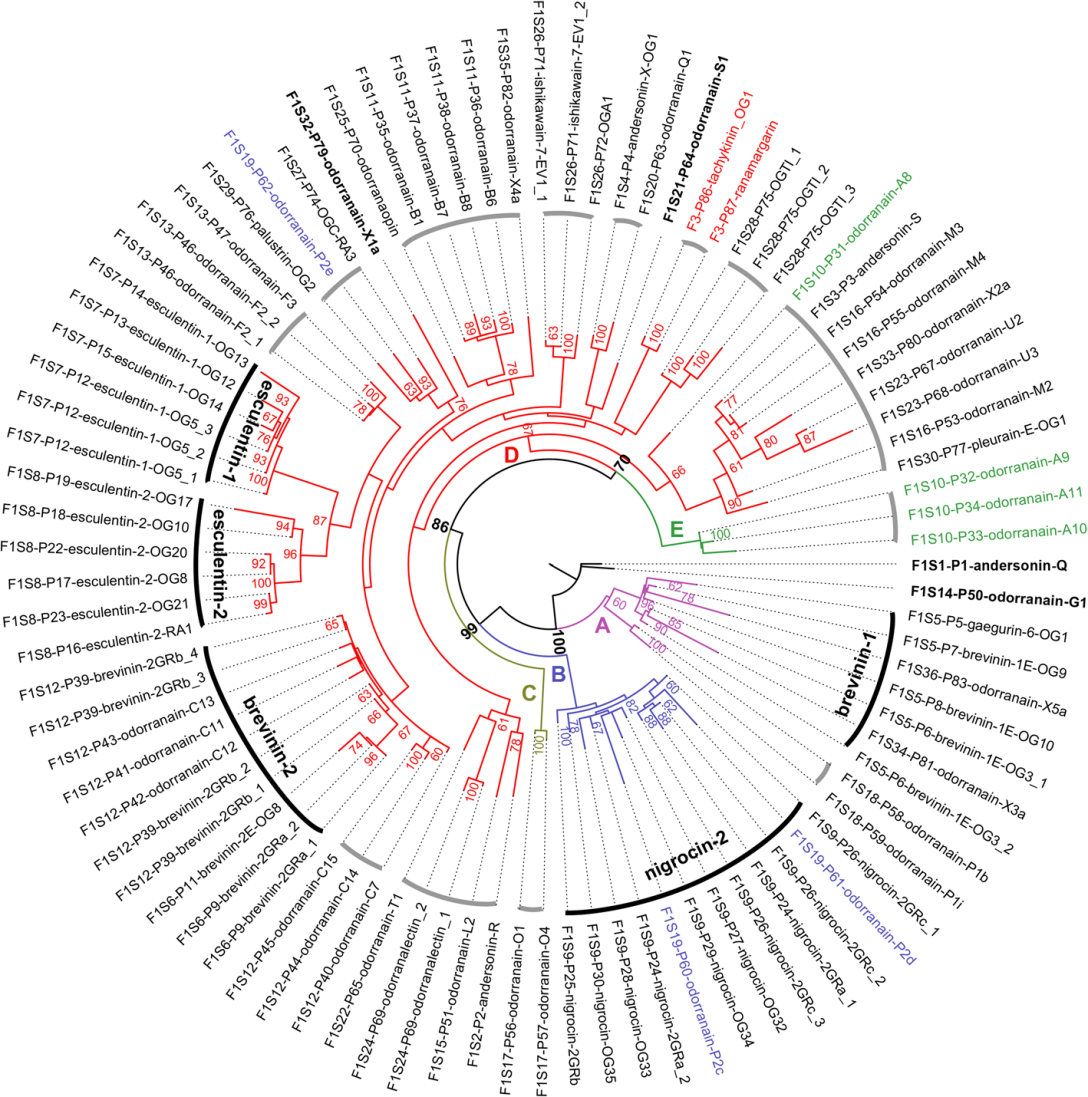
Phylogenetic tree of FSAP family peptides. The maximum likelihood phylogenetic tree of FSAP family peptides in this specimen is divided into five major clades (A–E), supported by ultrafast bootstrap values ≥ 60% (annotated at nodes if ≥ 60%). Taxa names follow the format F[family ID]S[subfamily ID]-P[peptide ID]-peptide name (Additional file 1: Table S4), with duplicates (identical mature peptide) marked by “_number.” Four peptides (bold font) failed to cluster into any groups due to low branch support (bootstrap < 60%), each representing a single transcript and a subfamily. The remaining 72 peptides clustered into 18 groups (segmented circular arcs), contrasting with the 32 traditional subfamilies. Among these, five groups aligned with previously reported well-established antimicrobial peptide families (dark arcs). Taxa names are labeled by color: blue font highlights odorranain-P2e (divergent from P2c/d), green font marks odorranain-A8 (divergent from A9–A11), and red font identifies tachykinin OG1 and ranamargarin (exhibiting convergent evolution with the tachykinin family). The largest clade (clade D) contains 56 peptides, including all known non-AMPs (odorranalectin, odorranaopin, andersonin-S, tachykinin OG1, and ranamargarin)

A total of 76 FSAP family peptides with complete CDS regions were identified in this specimen, representing 36 subfamilies through traditional classification. The dataset includes the tachykinin_OG1 and ranamargarin, which were counted as one additional FSAP subfamily. These peptides are associated with 90 precursor proteins and 91 transcripts. We aligned and trimmed the above four nucleic acid regions of their transcripts separately, and then used the merged sequences to construct a maximum likelihood phylogenetic tree (Fig. 4). With ultrafast bootstrap support of 60% or higher [10], the FSAP family peptides of this specimen are divided into five major clades (clade A–E). Within this structure, four peptides (bold font) failed to cluster into any groups due to low branch support (bootstrap < 60%), each representing a single transcript and a subfamily, while the remaining 72 peptides clustered into 18 groups (segmented circular arcs), approximately half the number of 32 subfamilies. Among these, five groups containing 33 peptides aligned with previously reported well-established antimicrobial peptide families (dark arcs) [63]. Specifically, clade A contains brevinin-1, clade B contains nigrocin-2, and clade D contains brevinin-2, esculentin-1, and esculentin-2. The largest clade (clade D) contained 56 peptides, including all known non-AMPs in this specimen: odorranalectin (lectin), odorranaopin (opioid), andersonin-S (antioxidant), as well as two peptides previously classified as members of the tachykinin family (tachykinin OG1 and ranamargarin: red font) (Additional file 2: Fig. S5), which demonstrate convergent evolution with peptides from the tachykinin family. Members belonging to the same subfamily typically cluster within the same group, with exceptions such as peptides from the odorranain-P2 and -A subfamilies. Specifically, P2e is distinct from P2c and P2d (blue font), while A8 diverges from A9, A10, and A11 (green font).

#### Functional distribution of ESPs

**Fig. 5:**
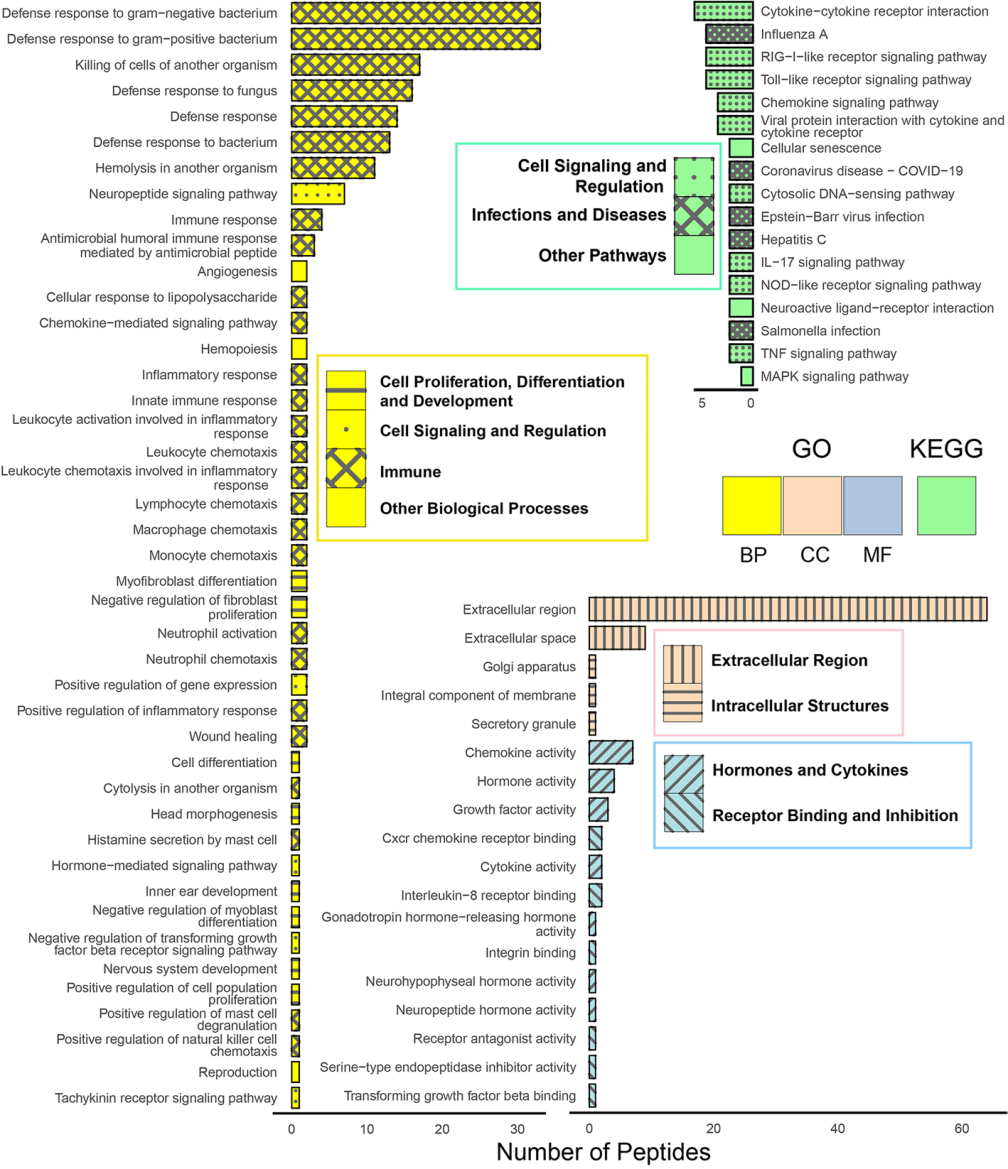
Functional distribution of ESPs. Among the 107 ESPs identified in this study (Additional file 1: Table S2), 75 ESPs were annotated with 61 Gene Ontology (GO) terms, while 8 ESPs were mapped to 17 Kyoto Encyclopedia of Genes and Genomes (KEGG) pathways via UniProt ID mapping (Additional file 1: Tables S9–S10). BP, biological process; CC, cellular component; MF, molecular function

Among the 107 ESPs identified in this study (Additional file 1: Table S2), 75 ESPs were annotated with 61 Gene Ontology (GO) terms, while 8 ESPs were mapped to 17 Kyoto Encyclopedia of Genes and Genomes (KEGG) pathways via UniProt ID mapping (Fig. 5; Additional file 1: Tables S9–S10). Within the GO biological process (BP) terms, i.e., pathways (43 pathways), the majority (62.8%, 27 pathways) relate to “immunity,” involving 64 ESPs. The remaining pathways pertain to “cell proliferation, differentiation, and development” (18.6%, 8 pathways), “cell signaling and regulation” (11.6%, 5 pathways), and “other biological processes” (7.0%, including angiogenesis, hemopoiesis, and reproduction), involving 13 ESPs. In the cellular component (CC), all five terms relate to the “extracellular region” and “intracellular structures,” while for the molecular function (MF), all 13 terms are associated with “hormones and cytokines” and “receptor binding and inhibition.” The significant majority of KEGG pathways (82.4%, 14 pathways) link to immune-related “cell signaling and regulation” (9 pathways) and “infections and diseases” (5 pathways), involving 5 ESPs. The “other pathways” (17.6%, including the MAPK signaling pathway, cellular senescence, and neuroactive ligand-receptor interaction) include 5 ESPs.

## Discussion

### Integrating transcriptomics and peptidomics for enhanced identification of ESPs

Building on conventional transcriptomics and peptidomics, we have incorporated additional steps to screen ESPs (Fig. 1: red text). This pipeline offers several advantages: (1) BLASTP custom reference sub-databases with taxonomic restrictions can improve both efficiency and accuracy. (2) Using ORF_all.sh to construct OSPS and SPS increased the identification rate of potential endogenous secretory proteins by 61.6%. Serial ORF_shift variants contributed to identification of signal peptide initiation sites (Additional file 1: Table S11). Additionally, SPS provides a relatively precise and comprehensive protein database for peptidomic or proteomic mass spectrometry searches. (3) Peptide.py filters out unreliable and unreasonable peptides, distinguishing between mature peptides and their truncated forms based on abundance. (4) The second BLASTP focuses on the conserved signal peptide region to “fish out” additional ESPs from the same family, especially those whose mature peptide regions lack similar sequences. Additionally, this pipeline is not only cost-effective but also provides quantitative data, enabling broader research possibilities.

The pipeline’s transcript and protein levels are complementary. The short reads generated by second-generation RNA-seq often result in incomplete transcript assemblies. Missing or incomplete signal peptide regions can cause SignalP to sometimes fail to recognize endogenous secretory protein. However, the mature peptide region may be detected at the protein level. For example, ranatuerin-2P-OG1, a novel ESP, is identified solely at the protein level and not recognized as a secretory precursor protein at the transcript level. Without peptidomic validation, ranatuerin-2P-OG1 would have been omitted in the “Sequences matched in the SPS” step (Fig. 1, the “Methods” section), as its transcript lacked a signal peptide region, and its mature peptide region is not 100% identical to previously reported homologs. Examining residual structural features on the precursor protein (propiece region and mature peptide region, Additional file 1: Table S3) and conducting antimicrobial activity assays (Section 1.3 and 2.3 of Additional file 2) [64, 65] can confirm it as an FSAP family peptide.

Although only 16 ESPs (15.0%) were validated at the protein level in this study, the remaining reported 91 putative ESPs were derived from transcript level evidence. This rate aligns with published ranges. Studies in amphibians, marine mollusks, and plant have demonstrated that only 4.9–35.1% of theoretical sequences derived from cDNA or transcriptomic methods could be validated at the protein level (see Additional file 1: Table S12) [9, 11, 66–68]. Furthermore, the scale of identified FSAP family peptides at the transcript level aligns with prior studies: 83 from our method vs. 107 from the cDNA method, both derived from single skin specimens of *O. grahami*. We ensured the reliability of transcripts lacking peptidomic validation through stringent quality control of transcriptomic data, bioinformatic filtering (excluding very low-abundance transcripts, retaining sequences with termination codons and secretory signal domains), and homology validation (BLASTP alignment). Furthermore, the CDS regions of the ESPs reported in this study exhibited ≥ 2 nucleotide differences between each other, with only two pairs of alternative splicing isoforms observed in the C-X-C_motif_chemokine family (10-OG1 vs. -OG2, 8-OG1 vs. -OG2; Additional file 1: Table S4), indicating a low probability of false positives caused by single-base sequencing errors or misassembly.

The limited overlap between transcriptomic predictions and peptidomic detection stems from both technical and biological factors [69, 70]. Current data-dependent acquisition (DDA)-based mass spectrometry exhibits an inherent bias toward high-abundance peptides and is constrained by limited sensitivity and dynamic range. These limitations are further compounded by challenges in chromatographic separation, which is influenced by peptide hydrophobicity, and by ionization efficiency, which is affected by the presence of basic amino acid residues, ultimately resulting in incomplete coverage of transcriptome-predicted proteins. Additionally, while deriving proteins from RNA-seq transcriptomic data is a common approach [38, 71, 72], algorithmic artifacts or short-read sequencing flaws may introduce false-positive predictions (e.g., false chimeric transcripts and splice variants). Biologically, post-translational modifications (PTMs) generate proteoforms, thereby hindering direct transcript-protein correspondence. Advances in data-independent acquisition (DIA), targeted proteomics, genomic data integration, and long-read sequencing may mitigate these issues.

Transcript levels and their cognate protein levels generally align, though translational regulation and protein turnover preclude strict correlation [73]. Logistic regression analyses revealed a significant association between peptide detection by mass spectrometry and an increase of one thousandth in the TPM value of the peptide’s transcript abundance (odds ratio = 1.462, *p* < 0.0001, *X* at 50% = 6.903). However, no linear correlation was observed between them (data not shown). These findings suggest that higher transcript abundance enhances protein detectability. We infer that peptides with higher transcript levels also tend to exhibit higher protein levels. This indicates that, irrespective of type or quantity, FSAP family peptides are the main ESPs in the skin of *O. grahami* (Additional file 1: Table S2).

### FSAP family peptides’ detection across protein databases: a pan-transcriptome and pan-peptidome perspective

We also conducted an MS/MS spectra search using a protein database created by integrating all ESP sequences from this specimen with those previously reported from the same species (Additional file 1: Tables S1–S2). This led to the identification of 34 ESPs belonging to the FSAP family, 18 more than those identified in the SPS. However, among these, six of these identifications exclusively corresponded to truncated peptide forms rather than mature peptides (highlighted in yellow in Result.xlsx of Additional file 3). Additionally, three had their “Rana Box” disulfide bonds in a reduced form (highlighted in red). None of these peptides originated from the transcriptome of this specimen. This study shows that truncated peptides are less abundant than their corresponding mature forms, consistent with early findings obtained through isolation-purification methods, such as ranatuerin-2GR (11–28), MRP-GR (1–18), and esculentin-1 (19–46) [59, 74]. The non-detection of these mature peptides was classified as tentative identifications, as the DDA method primarily detects high-abundance peptides. Furthermore, the unusual “Rana Box” in its reduced form has not been previously observed in the FSAP family. The reduced disulfide ring region (Cys1-SH, Cys2-SH) should have produced many fragment ions in the mass spectrometry [15]. However, manual inspection revealed near absence of fragment ions from these regions (Additional file 3, Data S2 of [44]), indicating that these identifications should be considered false positive. In light of these findings, half of these 18 additional ESPs (comprising 6 tentative and 3 false positive identifications) that lacked sequence matches in the SPS were deemed unreliable. To ensure the reliability of our findings, the results generated through this integrated search strategy were systematically excluded from the final analytical framework.

Therefore, we infer that some FSAP family peptides detected in other specimens may not be present in this specimen, leading us to exclude these peptides from further analysis. One piece of evidence supporting this inference is that a high-abundance peptide, esculentin-1GRa, isolated from skin secretions [7], was not detected in the transcripts of our specimen or the cDNA of another individual [11], despite all these animals originating from the Kunming population. However, it was found in the cDNA of an individual from *O. andersonii* (Fig. 2b; Additional file 1: Table S1). Another piece of evidence is that the production of the peptide brevinin-1SY within the same population of *Rana sylvatica* can be triggered by environmental temperature [75]. These findings indicate that individual-level FSAP family peptide expression profiles exhibit considerable diversity, even within the same population, although the considerable diversity between populations is already well known [76]. Therefore, the diversity of the FSAP family peptide is likely considerably underestimated, at least within the *Odorrana* genus. Therefore, these peptides are proposed to be considered within the context of the pan-transcriptome and pan-peptidome frameworks. In a previous study, significant variation in skin antimicrobial capacity among three populations and over three seasons was observed [77]. Here, we suggest that the diversity in the types of AMPs within the FSAP family may be a crucial molecular factor contributing to this observed variation.

### Challenges in FSAP family classification: discordance between sequence, function, and evolution in frog skin peptides

Current mainstream classification of AMPs is based on structural similarity of mature peptides. Early studies have identified at least 14 well-established peptide families (brevinin-1, brevinin-2, esculentin-1, esculentin-2, japonicin-1, japonicin-2, nigrocin-2, palustrin-1, palustrin-2, ranacyclin, ranalexin, ranatuerin-1, ranatuerin-2, temporin) [63]. With the explosive growth of AMPs, the number of families has rapidly expanded; for example, 30 families were reported in *Odorrana grahami* alone, while 97 families were reported across 9 other *Odorrana* species [9]. Since these AMPs are unique to Anura, their excessive proliferation directly contradicts the conservative principles of protein family evolution. In UniProt, these AMP families are unified under the FSAP family, where classical peptide families (e.g., brevinin, esculentin) are classified as subfamilies of FSAP. The total number of Anura-specific skin AMP families is reduced to three (including maximin-S and bombinin families) and a few scattered peptides (e.g., Riparins). UniProt classification advantages include the following: classification consistency (avoiding submerging cross-vertebrate AMP families like Magainin subfamily of gastrin/cholecystokinin family and cathelicidin family into numerous FSAP subfamilies); multifunctional integration (addressing peptides with multiple bioactivities beyond antimicrobial roles to prevent function-oriented classification failure); and functional prediction optimization (mitigating misjudgment from sequence-based predictions when some “antimicrobial peptides” lack experimental activity). Therefore, despite the limited adoption of FSAP family in literature [78, 79], the UniProt classification proves more rational for this study, which categorized 24 ESPs into 13 non-FSAP families and grouped 83 ESPs under the FSAP family (Additional file 1: Table S2).

At the subfamily level of the FSAP family, phylogenetic analysis revealed that five major clades (A–E) were supported by ultrafast bootstrap values ≥ 60% (Fig. 4), with many branches exhibiting ultrafast bootstrap support rates below 95%, consistent with prior studies (where support rate thresholds were set at 30–60%) [10, 80, 81]. This instability likely stems from the hypervariability of mature peptides, which provides insufficient phylogenetic signal. Extending conserved regions (e.g., 5′-UTR and 3′-UTR adjacent to CDS) could theoretically improve tree resolution, but previously reported *Odorrana* FSAP peptides universally lack 5′-UTR sequences; thus, these peptides were excluded from phylogenetic analysis. Traditional classification divided FSAP family peptides into 36 subfamilies in this specimen. However, phylogenetic analysis revealed significant contradictions: 72 peptides clustered into 18 groups—approximately half the number of traditional 32 subfamilies—underscoring the discordance between classifications based on nucleic acid sequences (including 5′-UTR and 3′-UTR adjacent to CDS) and those relying on mature peptide amino acid sequences. Notably, peptides from traditional subfamilies could fail to cluster cohesively. For example, odorranain-P2e (blue font) diverged from P2c/d, while odorranain-A8 (green font) diverged from A9 to A11. These inconsistencies highlight the limitations of traditional classification. On the other hand, UniProt subfamily classification appears overly restrictive: UniProt lists 1254 FSAP members (mature peptides or precursors) divided into 26 subfamilies (9 unclassified). *Odorrana* contributes 359 members across only four subfamilies (brevinin, temporin, esculentin, ranatuerin), with 120 members of *O. grahami* further restricted to just two subfamilies (brevinin, esculentin). However, phylogenetic analysis reveals a more complex picture: only five clades (dark arcs) align with well-established AMP families (brevinin-1, brevinin-2, nigrocin-2, esculentin-1, and esculentin-2), covering merely 45.8% of peptides (33 of 72). This discordance between the static UniProt subfamilies and dynamic evolutionary clades demonstrates that the current classification underestimates the divergence of FSAP peptides. Consequently, both the evolutionary relationships and subfamily delineation among FSAP peptides remain unresolved. To address this, future studies should integrate FSAP nucleic acid sequences with complete conserved regions across representative species to reconstruct robust evolutionary relationships and clarify subfamily classifications.

The phylogenetic clustering of non-AMPs (e.g., lectin, opioid, and antioxidant peptides) and functionally convergent tachykinin-like peptides (tachykinin OG1 and ranamargarin: red font) within clade D challenges function-based naming conventions, as peptides with the same function do not necessarily reflect their evolutionary origin, and naming by function can lead to confusion [3]. Such functional convergence suggests that peptide nomenclature must prioritize evolutionary origins over functional traits, particularly in clades such as clade D that exhibit rapid evolutionary processes and non-canonical functions.

### Immune-related ESP families in the skin of *O. grahami*

The adult anuran immune system is fundamentally similar to that of other jawed vertebrates and responds similarly to antigenic stimulation. However, there are some differences [82, 83]. From the perspective of ESPs, the most significant difference is the large number of anuran-specific FSAP family peptides. Based on their activities, the peptides of this family are primarily AMPs. However, increasing experimental evidence suggests that some FSAP family peptides have a substantial effect on mammalian cell processes such as cell migration, inflammation, immunity, and repair [1, 84, 85]. Thus, some members are customarily classified solely or additionally as antioxidant peptides, myotropic peptides, opioid peptides, protease inhibitor peptides, lectins, insulin-releasing peptides, mast cell degradation/histamine-releasing peptides, wound-healing peptides, immunomodulatory peptides, and neuronal nitric oxide synthase inhibitors [4]. For the peptides of *O. grahami*, some members of this family also have been found to possess non-AMP functions (Additional file 1: Table S1). Therefore, it is essential to enhance the testing of the multiple functions of these peptides, reinterpreting their role in the immune system.

Based on the GO and KEGG pathways, the other potentially immune-related ESP families, which have mammalian counterparts, present in the skin of *O. grahami* are as follows: Members of the cathelicidin family from mammals, frogs, and fishes possess significant immunomodulatory activity in addition to their antimicrobial activity [86–88], while a non-bactericidal member (*Popu*CATH) from frog *Polypedates puerensis* acts as a host-based immune defense regulator [89]. Although intercrine alpha and bombesin/neuromedin-B/ranatensin family peptides lack antimicrobial activity, they play important roles in the immune system of mammals [90, 91]. Members of the intercrine alpha family, CXCL8 (also known as C-X-C motif chemokine 8, Interleukin-8, or IL8), from both *Xenopus laevis* and zebrafish, have been confirmed to be involved in inflammatory responses and the recruitment of neutrophil/granulocyte subsets [92, 93]. A member of the bombesin/neuromedin-B/ranatensin family, bombesin from *Bombina bombina*, has demonstrated in vitro wound healing potential by enhancing the expression of CXCL8, TGFβ, COX-2, VEGF, and TLR2 in mechanically injured HaCat cells [94]. Although current GO and KEGG pathway annotations do not directly link tachykinin family peptides of amphibians to immune functions, some mammalian members of this family have been proven to possess immunological roles [95]. Despite these insights, our knowledge of the cellular and molecular mechanisms underlying amphibian skin immunity and pathogen defense remains limited. Most of our understanding comes from inferences based on mammalian immunology or evidence from mammalian cells [1, 82], rather than frog cells [96]. Even with advanced sequencing technologies, there are still gaps in the annotations of the immunome that need to be addressed [3, 93].

## Conclusions

Our work exemplifies the challenges of discovering and annotating ESPs, demonstrating that even well-studied species harbor numerous undiscovered ESPs. This suggests that the diversity of the FSAP family peptide is likely considerably underestimated, at least within the *Odorrana* genus, and underscores the critical need for more comprehensive approaches in peptide discovery. This study not only advances our understanding of the molecular diversity of ESPs in amphibians but also provides a valuable resource for further research in comparative vertebrate skin biology. Since ESPs universally contain signal peptides, our comprehensive approach can be adapted to discover ESPs in other organisms by substituting appropriate taxonomic reference sub-databases. Future studies should focus on elucidating their functional roles in the immune system at the individual level and exploring their potential applications.

## Methods

### Overview of the integrated pipeline

In brief, the pipeline consists of three parts, as shown in Fig. 1. The first part (green) is about the detection of endogenous secretory proteins at the transcript level following the construction of transcriptome, including the generation of the potential ORF_shift proteins, OSPS and SPS from the FPS by our script ORF_all.sh. The second part (blue) is about the identification of peptides at the protein level, including the extraction of peptides from secretion, the analysis of peptide mass and composition by LC–MS/MS, and the identification of mature peptides by our Python script Peptide.py. The third part (yellow) is about the peptidome discovery by integrating protein- and transcript-level data, which includes extracting the ESPs through a multi-step process that involves assigning signal peptides, executing two-step homology BLASTP searches, and finally performing manual annotations. Detailed methodologies for each component are described in subsequent sections, following the workflow order outlined in Fig. 1.

### Collection of skin secretion and skin total RNA

An adult male *O. grahami* was collected in July 2015 from Kunming, Yunnan Province, China. The collection site was at an altitude of 2068 m with coordinates E 102° 55.490′ N 25° 00.750′ and the frog weighed 39.8 g. Skin secretion was collected using the mild electrical stimulation technique as previously reported [77]. The collected secretion was lyophilized following air pump filtration and stored at − 80 °C until use. After the secretion collection, the frog was anesthetized and sacrificed using chlorobutanol, and the skin was collected immediately and stored in liquid nitrogen. Total skin RNA was extracted using the Trizol method and stored at − 80 °C until use.

### Construction of transcriptome

We constructed the transcriptome using mRNA purified with poly-T oligo-attached magnetic beads. Sequencing was performed on an Illumina NovaSeq 6000 system (PE150) by Novogene, Beijing, China. Read quality was assessed using FastQC v0.11.9 and summarized with MultiQC v1.9. Raw read base calling accuracy was calculated using our Base_calling_accuracy_calculator.py [97]. Trimming was applied using Trimmomatic v0.39, and genome alignment was performed with FastQ Screen v0.15.2 using bowtie2 v2.4.1. Transcriptome assembly was conducted with Trinity v2.11.0 without read normalization, and assembly quality was evaluated using QUAST v5.1.0rc1 and BUSCO v4.1.4 against the eukaryota_odb10 database. Read representation and transcript abundance were quantified using Bowtie2 and Salmon v1.3.0, respectively. The computational annotation was performed using the Trinotate v.3.2.1 pipeline. Three custom reference sub-databases comprising Swiss-Prot (all vertebrates, Data S3 of [44]), TrEMBL (all vertebrates, Data S4 of [44]), and NCBI nr (Amphibia, Data S5 of [44]) were respectively created for BLAST homology searches using DIAMOND v2.0.15. For detailed information, please refer to Section 1.1 of Additional file 2 [34, 98–107].

### Detection of endogenous secretory proteins at the transcript level

We established a workflow to comprehensively capture endogenous secretory protein candidates through the following steps in sequence (Fig. 1):

#### Filtered TransDecoder protein set construction

From the computationally annotated TransDecoder (v5.5.0) proteins, we generated the FPS through three sequential filters: (1) expression filtering: excluded proteins corresponding to transcripts with read counts < 1; (2) length exclusion: removed sequences shorter than 20 amino acids (aa); (3) deduplication: collapsed 100% identical sequences with FASTA header consolidation using comma (“,”) delimiters.

#### Open reading frame-shifted protein generation

Using the ORF_all.sh script [108], we systematically generated ORF_shift proteins from FPS through the following step: (1) truncation series generation: methionine residues were utilized as truncation sites to systematically generate a series of truncated variants from the N-terminus of each protein sequence (starting from the second amino acid). The derivative protein variants were annotated by appending “_ORF” with incrementing numerals (e.g., TRINITY_DN1_c1_g1_i1.p1_ORF1, _ORF2), where suffix numeration reflects increasing removal of N-terminal regions; (2) length filtering: variants shorter than 20 aa were discarded; (3) deduplication: 100% identical sequences were collapsed, with consolidated FASTA headers using pipe (“|”) delimiters.

#### Optimized secretory protein set identification

The ORF_all.sh script automated secretory protein prediction by executing SignalP v5.0b on ORF_shift proteins, and positive hits were extracted to form the OSPS.

#### Search protein set construction

The SPS was constructed through ORF_all.sh-integrated merging of the FPS and OSPS with subsequent collapse of 100% sequence identity duplicates in the integrated dataset.

### Identification of peptides at the protein level

#### Peptide extraction and LC–MS/MS

The skin secretion was initially dissolved in a mixture of ethanol and HCl. The solution was then subjected to a SpeedVac concentrator to remove the ethanol and HCl. Deionized water was added to the residue, and the suspension was centrifuged. The supernatant was filtered using Amicon Ultra-4 10kDa MWCO centrifugal filter units. The eluent was desalted using a C18 cartridge. The peptide sample was eluted onto an analytical C18 column using gradient elution on an LC-20AD nanoHPLC (Shimadzu, Kyoto, Japan), coupled with a Q Exactive mass spectrometer (ThermoFisher Scientific, San Jose, CA) operating in positive polarity mode and employing the top 20 method of data-dependent acquisition (DDA).

For protein identification and quantification, we used Proteome Discoverer v2.5 (PD) (Fig. 1). The SPS served as the search protein database, while the contaminants database (contaminants.fasta file) was obtained from MaxQuant. Parameters included No-Enzyme, a precursor mass tolerance of 3 ppm, and a fragment mass tolerance of 0.02 Da. Apart from the default dynamic modifications, dehydro of cysteine (− 1.008 Da) [45] and deamidated asparagine (0.984 Da) were included as additional dynamic modifications, which were the most abundant (30.40% and 27.27% of total modifications, respectively) detected by pFind v3.1.6 [109]. For detailed information, please refer to Parameters S1–S2 and Sect. 1.2 in Additional file 2 [15, 45, 109].

#### Identification of mature peptides at the protein level

We established a workflow as part of our overall pipeline to determine the mature peptide at the protein level (Fig. 1). Initially, we extracted the master protein data frame from PD (Additional file 3). Next, we processed it using Peptide.py [110], a Python script designed to eliminate proteins with medium or low FDR confidence and peptides with an odd number of dehydro of cysteine, combine peptide isoforms, accumulate their abundances, and identify the most abundant peptide among exclusive peptides of each master protein as the candidate mature peptide. Finally, the final list of mature peptides was obtained by removing any candidate mature peptides for which all precursor proteins had fewer than 3 peptide-spectrum matches (PSMs) or lacked a stop codon.

### Discovery of ESPs via integrated transcriptomics and peptidomics

#### Identification of ESPs from the SPS and the OSPS

We identified the ESPs in the skin of this specimen through a multi-step process (Fig. 1). Initially, we merged the mature peptide sequences detected at the protein level in this study with the previously reported mature peptide sequences in *O. grahami* (Additional file 1: Table S1) and matched them to the protein sequences in the SPS, where a “match” was defined as a full-length query sequence with 100% identity to any contiguous subsequence in the SPS. Subsequently, we extracted sequences containing stop codons and fewer than 250 aa from OSPS, and excluded those sequences that had already been matched in the SPS. Using SignalP, we annotated both the signal peptide sequences and the intermediate sequences, which encompass the propiece, the mature peptide, and the residual piece of the precursor protein remaining after signal peptide removal.

Next, we conducted a homology BLASTP search of their intermediate sequences against the three reference sub-databases using DIAMOND v2.0.15. The parameters were set as follows: for sequences of 30 aa or fewer, “–evalue 1 –id 65 –short-query-ungapped-bitscore 15 –gapped-filter-evalue 0 –shape-mask 1111 –block-size 3”; for sequences exceeding 30 aa, “–evalue 1e-3 –more-sensitive”. Thereafter, we performed a second homology BLASTP search of un-hit proteins’ signal peptides against signal peptides from proteins matched in SPS or hit in the first BLASTP.

Finally, we manually annotated all matched or hit sequences obtained from the preceding steps. This included selecting the correct signal peptide from serial ORF_shift variants based on their conserved structures in the signal peptide region and their 5′-UTR. The remaining structural features in each intermediate sequence (such as the propiece, the mature peptide, and the residual piece) were inferred based on their high similarity with known proteins and/or conserved structural motifs. For FSAP family peptides, in addition to general sequence similarity-based predictions, the mature peptide’s N-terminus is primarily predicted to result from cleavage at the propiece’s C-terminal single or paired basic residues (predominantly Lys-Arg dipeptide motifs), based on mechanisms experimentally validated in mammalian proprotein convertase systems [9, 11, 62, 111]. Precursor proteins where all final mature peptides exceeded 100 amino acids were removed.

The family classification adheres to the UniProt framework [8, 28], integrating InterPro domain signatures (Protein_Pfam_Top_hit and Protein_InterPro_hit in Additional file 1: Table S3), sequence homology (Protein_sprot_Top_hit, Protein_trembl_Top_hit and Protein_nr_Top_hit), and scientific literature analysis. For FSAP family peptides, subfamily assignments and nomenclature are determined by homology-based identification (Peptide_sprot_Top_hit, Peptide_trembl_Top_hit and Peptide_nr_Top_hit in Additional file 1: Table S2). Mature peptides aligning with known references are assigned to their corresponding subfamilies and named according to conventional nomenclature derived from homologous peptides, while unassigned Orphans are designated as odorranain-X with a numeric-alphabetic suffix (e.g., odorranain-X1).

#### Phylogenetic analysis of FSAP family peptides

The sequences were aligned using MAFFT v7.486 with the E-INS-i algorithm. Ambiguously aligned positions were removed using trimAl v1.4.rev15 with the option “-automated1.” The phylogenetic reconstruction of FSAP family peptides was performed using IQ-TREE v2.06 under the maximum likelihood framework, incorporating ModelFinder Plus for best-fit model selection and 1000 replicates of ultrafast bootstrap. The resulting alignment and tree were visualized using MView v1.67 and FigTree v1.4.4, respectively.

#### Logistic regression analysis of transcript abundance and peptide detection

We performed binary logistic regression analysis with GraphPad Prism v8.3.0 (GraphPad Software) to evaluate the effect of the TPM value of the peptide’s transcript abundance on the likelihood of peptide detection by mass spectrometry.

## Supplementary Information

Additional file 1: Table S1. Previously reported ESPs in *O. grahami*. Tables S2–S7. Identified ESPs in this study, including ESP lists (S2), precursor protein sequences (S3), transcript sequences (S4), signal peptide start (S5), signal peptide cleavage sites (S6), and enzyme cleavage sites before ESPs (S7). Table S8. Reported FSAP family peptides in *O. andersonii.* Tables S9–S10. GO terms (S9) and KEGG pathways (S10) of ESPs. Table S11. Candidate ORF_shift protein sequences of identified ESPs. Table S12. Previously reported validation of identified transcripts at the protein level.

Additional file 2: Supplementary methods, results, and analysis parameters. Fig. S1. Quality assessment and cross-species sequence conservation analyses. Fig. S2. Precursor detection by LC-MS/MS. Fig. S3. Overview of mature peptide and its truncated peptides identified by LC-MS/MS. Figs. S4–S16. Structural features of 13 ESP families identified in this study. Table Sa – Transcriptome annotation items generated by the Trinotate pipeline. Table Sb –MICs of ranatuerin-2P-OG1. Parameters S1–S2 – Search parameters used in pFind (S1) and Proteome Discoverer (S2) analyses.

Additional file 3. Exported and processed results derived from the native Proteome Discoverer result files (*.pdResult; Data S2 of [44]). SPS/ – Results of searching against the SPS database. All_ESP_sequences_from_this_species/ – Results of searching against all ESP sequences from this species, including previously reported ones; contains suspected false positives (e.g., esculentin-2-OG1/OG2, odorranain-C3). Annotated Spectra.zip, *_export.xlsx, MSMS Scans and PSMs.xlsx – Files exported from *.pdResult, including annotated MS/MS spectra, protein/peptide isoform identifications, and MS/MS scan metadata. Result.xlsx – Final filtered peptide list obtained after processing by the Peptide.py script.

Additional file 4. Mass spectrometry-detected mature peptides and truncations mapped to corresponding master proteins (excluding brevinin-2GRa, shown in Additional file 2: Fig. S3a).

Additional file 5. Alignments of ESP sequences identified in this study across different regions.

## Data Availability

The project was deposited into the National Genomics Data Center (NGDC) [43]. The FPS, ORF_shift protein, OSPS, and SPS (Data S1); the native Proteome Discoverer result files (Data S2); and three custom reference sub-databases comprising Swiss-Prot (all vertebrates, Data S3), TrEMBL (all vertebrates, Data S4), and NCBI nr (Amphibia, Data S5) can be accessed at Figshare [44]. Custom scripts used in the analyses—including base calling accuracy evaluation, ORF_shift protein extraction, and processing of peptide sequences exported from Proteome Discoverer—are available on Zenodo [97, 108, 110]. The 84 transcripts encoding 81 proteins, which correspond to 74 newly identified peptides in this study, including 62 novel peptides, have been jointly deposited in GenBank [112] and GenBase [113]. The accession numbers for these sequences are also provided in the “GenBase accession number” and “GenBank accession number” columns of Additional file 1: Tables S2-S4. The Additional files 1-5 are available as supporting information.

## Abbreviations

FPS: Filtered TransDecoder protein set
ORF_shift protein: Open reading frame-shifted protein
OSPS: Optimized secretory protein set
SPS: Search protein set
