# Supplementary material for: Integrative transcriptomics and peptidomics approach reveals unexpectedly diverse endogenous secretory peptides in Odorrana grahami frog skin
Source: BMC Biol. 2025 Nov 28;23:354. doi: 10.1186/s12915-025-02463-w (PMC12664280; doi:10.1186/s12915-025-02463-w)
Supplement: Supplementary file 2 — Additional file 2: Supplementary methods, results, and analysis parameters. Fig. S1. Quality assessment and cross-species sequence conservation analyses. Fig. S2. Precursor detection by LC-MS/MS. Fig. S3. Overview of mature peptide and its truncated peptides identified by LC-MS/MS. Figs. S4–S16. Structural features of 13 ESP families identified in this study. Table Sa – Transcriptome annotation items generated by the Trinotate pipeline. Table Sb –MICs of ranatuerin-2P-OG1. Parameters S1–S2 – Search parameters used in pFind (S1) and Proteome Discoverer (S2) analyses. [file 12915_2025_2463_MOESM2_ESM.docx]

Additional file 2

目录

[Additional file 2 1](#_Toc213286062)

[1. Material and methods 3](#_Toc213286063)

[1.1 Construction of transcriptome 3](#_Toc213286064)

[1.2 Peptide extraction and LC-MS/MS 5](#_Toc213286065)

[1.3 Antimicrobial assays 7](#_Toc213286066)

[2. Results 7](#_Toc213286067)

[2.1 Construction of transcriptome 7](#_Toc213286068)

[2.2 Families and structural features of the remaining 13 families of ESPs 8](#_Toc213286069)

[2.3 Antimicrobial activity of ranatuerin-2P-OG1 12](#_Toc213286070)

[Tables 13](#_Toc213286071)

[Table Sa. Transcriptome annotation items generated by the Trinotate pipeline. 13](#_Toc213286072)

[Table Sb. The minimum inhibitory concentrations (MICs) of ranatuerin-2P-OG1 (μg/mL). 14](#_Toc213286073)

[Figures 15](#_Toc213286074)

[Figure S1. Quality assessment and cross-species sequence conservation analyses. 15](#_Toc213286075)

[Figure S2. Precursor detection by LC-MS/MS. 16](#_Toc213286076)

[Figure S3. Overview of mature peptide and its truncated peptides identified by LC-MS/MS. 17](#_Toc213286077)

[Figure S4. The FARP (FMRFamide-related peptide) family. 20](#_Toc213286078)

[Figure S5. The tachykinin family. 21](#_Toc213286079)

[Figure S6. The GnRH family. 22](#_Toc213286080)

[Figure S7. The amotoxin family. 23](#_Toc213286081)

[Figure S8. The NPY family. 24](#_Toc213286082)

[Figure S9. The 7B2 family. 25](#_Toc213286083)

[Figure S10. The bombesin/neuromedin-B/ranatensin family. 26](#_Toc213286084)

[Figure S11. The calcitonin family. 27](#_Toc213286085)

[Figure S12. The cathelicidin family. 28](#_Toc213286086)

[Figure S13. The Galanin family. 29](#_Toc213286087)

[Figure S14. The vasopressin/oxytocin family. 30](#_Toc213286088)

[Figure S15. The insulin family. 31](#_Toc213286089)

[Figure S16. The intercrine alpha (chemokine CxC) family. 32](#_Toc213286090)

[Parameters 34](#_Toc213286091)

[Parameter S1: Parameters used in pFind. 34](#_Toc213286092)

[Parameter S2: Parameters used in PD. 34](#_Toc213286093)

# 1. Material and methods

## 1.1 Construction of transcriptome

The RNA sample preparations in this study utilized total RNA as input material. Sequencing libraries were generated using NEBNext® Ultra™ RNA Library Prep Kit for Illumina® (NEB, USA) following manufacturer’s recommendations. Purification of mRNA was performed using poly-T oligo-attached magnetic beads, followed by fragmentation. First-strand cDNA was synthesized using M-MuLV Reverse Transcriptase and random hexamer primers, and RNaseH was used to degrade the RNA. Subsequently, second-strand cDNA synthesis was conducted using DNA Polymerase I and dNTP. The remaining overhangs were converted into blunt ends via exonuclease/polymerase activities. Adenylation of 3’ ends of DNA fragments was performed, and an adaptor with a hairpin loop structure was ligated to prepare for hybridization. The library fragments were purified using the AMPure XP system (Beckman Coulter, Beverly, USA). Following PCR amplification, the PCR product was purified by AMPure XP beads, and the library was finally obtained. The library was initially quantified using a Qubit2.0 Fluorometer (Invitrogen, Carlsbad, CA, USA), and the insert size of the library was detected using an Agilent 2100 bioanalyzer. Finally, qRT-PCR was used to accurately quantify the effective concentration of the library. The library preparations were sequenced on an Illumina NovaSeq 6000 with a paired-end sequencing length of 150 bp (PE150) by Novogene (Beijing, China).

The quality of the reads was assessed using FastQC v0.11.9 (https://www.bioinformatics.babraham.ac.uk/projects/fastqc/) and summarized by MultiQC v1.9 [98]. Raw read base calling accuracy was calculated using a Python script (Base_calling_accuracy_calculator.py, [97]), which relied on the Phred Score exported from the Per Sequence Quality Scores item in MultiQC. Trimmomatic v0.39 was utilized to remove adapters and perform quality trimming with the following settings: “LEADING:5 TRAILING:5 SLIDINGWINDOW:4:5 MINLEN:25” [99]. The trimmed reads were analyzed using FastQ Screen v0.15.2 (--aligner bowtie2) primarily to assess exogenous contamination against reference sequences of common contaminants such as Drosophila, Worm, Yeast, Arabidopsis, Ecoli, rRNA, MT (mitochondria), PhiX, Lambda, Vectors, and Adapters; secondarily to evaluate sequence conservation through alignments to amphibian reference genomes including *Xenopus tropicalis, Leptobrachium leishanense, Bufo gargarizans, Rana temporaria, Nanorana parkeri, Lithobates catesbeianus, Spea multiplicata, and Microcaecilia unicolor* [100]. The transcriptome was assembled with Trinity v2.11.0, using the parameter “--no_normalize_reads” [101]. Assembly statistics were evaluated using QUAST v5.1.0rc1 [102]. The completeness concerning gene content was assessed using BUSCO v4.1.4, with the eukaryota_odb10 database as a reference [103]. The quality of the transcriptome in terms of read representation was evaluated by mapping the trimmed reads back onto the transcriptome using Bowtie2 v2.4.1 [104]. Transcript abundance was quantified with Salmon v1.3.0 [105], with the “--gcBias” option executed by the script “align_and_estimate_abundance.pl” of Trinity. The transcript count matrix and transcript TPM matrix were created using abundance_estimates_to_matrix.pl of Trinity.

The Trinotate pipeline v.3.2.1 was utilized to computational annotate the biological data [106]. In addition to the standard Trinotate pipeline, which leverages TransDecoder v.5.5.0 with customed parameter “-m 50” to predict candidate open reading frames (ORFs) (https://github.com/TransDecoder/TransDecoder), HMMER v3.3.1 to identify protein domains, SignalP v5.0b to predict signal peptides and their cleavage sites of secretory precursor proteins [34], TMHMM v2.0c to predict transmembrane helices of proteins, and RNAmmer v1.2 to predict ribosomal RNA subunits, alterations were made to reference databases for BLAST homology searches. Specifically, we created a custom SQLite v 3.33.0 database containing background information from the UniProt Knowledgebase for all vertebrates. To do this, we merged the protein sequences from human, rodents, mammals, and other vertebrates to extract protein sequences of all vertebrates in Swiss-Prot and TrEMBL, respectively (ftp.uniprot.org/pub/databases/uniprot/current_release/knowledgebase/taxonomic_divisions/). Meanwhile, we retrieved protein sequences of Amphibia from the NCBI non-redundant protein database (nr) (https://ftp.ncbi.nlm.nih.gov/blast/db/). In brief, we employed the “blastdbcmd” command in NCBI BLAST+ v2.12.0 to extract the sequences based on taxonomy_ids, which we obtained from the NCBI Taxonomy database dump files (ftp.ncbi.nlm.nih.gov/pub/taxonomy/) using TaxonKit v0.8.0 with the parameter “--ids 8292” (https://github.com/shenwei356/taxonkit). Finally, we created three reference sub-databases using DIAMOND v2.0.15 [107] from the above protein sequences separately [44]: Swiss-Prot (all vertebrates), TrEMBL (all vertebrates), and nr (Amphibia). We utilized the Swiss-Prot reference sub-database for BLASTX and BLASTP homology searches, and the TrEMBL and nr reference sub-databases for BLASTP homology searches, respectively. The parameters for homology search in DIAMOND was set to “--evalue 1e-5 --more-sensitive”. GO and KEGG annotations for nr items, outside of the Trinotate pipeline, were performed by mapping RefSeq IDs to GO IDs using the NCBI ID mapping files (gene2go.gz and gene2refseq.gz, https://ftp.ncbi.nih.gov/gene/DATA/) and mapping RefSeq IDs to KEGG IDs using the UniProt ID mapping file (idmapping.dat.gz, https://ftp.uniprot.org/pub/databases/uniprot/current_release/knowledgebase/idmapping/).

## 1.2 Peptide extraction and LC-MS/MS

The skin secretion was initially dissolved in a mixture of ethanol and 0.7 M HCl (3:1 v/v, 10 mL). The resulting solution was then subjected to a SpeedVac concentrator to remove the ethanol and HCl. To the resulting residue, 2 mL of deionized water was added, and the suspension was centrifuged at 13000 × g for 25 min at 4 °C. The supernatant was then filtered using Amicon Ultra-4 10k MWCO centrifugal filter units at 4 °C and 4000 × g. The eluent was subsequently desalted using a C18 cartridge and dried using a SpeedVac concentrator. The peptide sample was quantified by measuring the absorbance of the peptide bonds at 215 nm and 225 nm, using the relation: concentration (μg/mL)=144 × (A215 - A225) [15].

The peptide sample was resuspended in buffer A (5% ACN, 0.1% FA) and centrifuged at 20,000 × g for 10 minutes, resulting in a final peptide concentration of approximately 0.5 μg/μl. Subsequently, 10 μl of the supernatant was loaded onto an LC-20AD nanoHPLC (Shimadzu, Kyoto, Japan) and eluted onto a 10 cm analytical C18 column (inner diameter 75 μm) packed in-house. A 38-minute gradient was run at 300 nL/min, beginning at 5% buffer B (98% ACN, 0.1% FA) and progressing to 30% buffer B, followed by a 5-minute linear gradient to 80% buffer B, and a 5-minute maintenance period. The Q Exactive mass spectrometer (ThermoFisher Scientific, San Jose, CA) was operated in positive polarity mode with a capillary temperature of 275 °C. The full MS scan resolution was set to 70,000 with an AGC target value of 3e6 for a scan range of 350-1800 m/z. Using a 2.0 m/z isolation window and an NCE of 27, HCD spectra were acquired at a resolution of 17,500 MS^2^ with an AGC target of 5e4 and a maximum IT of 100 ms, by the top 20 method of data-dependent acquisition (DDA). Dynamic exclusion was set to 8 seconds.

Peptide identification and quantification were performed using Proteome Discoverer v2.5 (PD, Thermo Fisher Scientific). The SPS database was used as the search protein database, while the contaminants database (contaminants.fasta) was obtained from MaxQuant (http://www.coxdocs.org/doku.php?id=maxquant:start_downloads.htm). At the Processing step, precursor mass between 350 Da and 6000 Da with S/N Threshold 1.5 was selected in the Sequest HT node. The following parameters were used: No-Enzyme, precursor mass tolerance of 3 ppm, and fragment mass tolerance of 0.02 Da. A maximum of two equal modifications and four dynamic modifications per peptide were allowed. Apart from the default dynamic modifications, such as oxidation of Methionine (15.995 Da), N-terminal acetylation (42.011 Da), N-terminal loss of Methionine (-131.040 Da), and N-terminal loss of Methionine + acetylation (-89.030 Da), dehydro of Cysteine (-1.008 Da) [45] and deamidated of Asparagine (0.984 Da) were included as additional dynamic modifications. The last two modifications were the most abundant (30.40% and 27.27% of total modifications, respectively) detected by pFind v3.1.6, a program that considers accidental modifications [109]. The validation was performed using the Percolator with false discovery rates (FDR) between 0.01 (Strict) and 0.05 (Relaxed). At the Consensus step, peptide confidence was set at high with a minimum peptide length of six. A minimum of one peptide was required to identify a protein. Precursor abundance was based on intensity with the Minora algorithm. A complete list of the parameters used in pFind and PD is provided in Appendix S1-S2.

We applied the Shapiro-Wilk test to evaluate the normality of the Δppm values for the PSMs with GraphPad Prism v8.3.0 (GraphPad Software).

## 1.3 Antimicrobial assays

Ranatuerin-2P-OG1 synthesis was performed by Royobiotech Co., Ltd. (Shanghai, China) using solid-phase peptide synthesis. The crude peptide was purified by RP-HPLC and its purity was confirmed to exceed 90% via ESI-MS analysis. Antimicrobial assays with various microbial strains were conducted, including gram-positive *Staphylococcus aureus* ATCC 29213 and its methicillin-resistant variant (MRSA) CIB 8546, *Enterococcus faecalis* ATCC 29212, gram-negative *Pseudomonas aeruginosa* ATCC 27853 and *Escherichia coli* ATCC 25922, and fungal *Candida albicans* CCMCC(F) 98001. Bacterial cultures were maintained in cation-adjusted Mueller-Hinton broth (MHB) at 35°C for 16-20 h, whereas fungal cultures were grown in Sabouraud dextrose broth (SDB) at 28°C for 48 h. The minimum inhibitory concentrations (MICs), defined as the lowest peptide concentration that completely inhibited visible microbial growth, were determined using standard microdilution methodology in 96-well microtiter plates: bacterial assays followed CLSI (2009) M07-A8 guidelines, while antifungal testing adhered to NCCLS (2002) M27-A2 document [64, 65].

# 2. Results

## 2.1 Construction of transcriptome

The RNA sequencing generated 26.3 million 150 bp paired-end reads with a base calling accuracy of 99.96%. After quality control using Trimmomatic, the retained reads were 25.5 million 25-150 bp paired-end reads. Trinity generated 217,270 transcripts clustered into 167,425 “genes” with N50 of 1272 bp and a GC content of 44.8%. Bowtie2 mapped 94.6% of the reads back onto the transcriptome. The BUSCO results supported near-complete gene sequence information for 96.9% of genes in the transcriptome against the eukaryota_odb10 database. Gene annotation via the Trinotate pipeline provided biological context to the assembled transcriptome. TransDecoder recovered 181,817 proteins, corresponding to 62.0% of transcripts and 58.9% of “genes” identified by Trinity. Of these, 31.8%, 35.7%, and 46.3% of genes, transcripts, and proteins, respectively, were annotated at least once (Table Sa). The FastQ Screen analysis supported the reliability of the RNA-seq data due to minimal alignments (0-0.2%) of the trimmed reads to common contaminant sequences. Cross-species alignment revealed that 63.6% and 68.2% of reads mapped to *R. temporaria* and *L. catesbeianus* genomes, respectively, suggesting sequence conservation (Fig. S1a). Complementary BLASTP homology analysis of TransDecoder proteins against the nr sub-database of Amphibia identified these species as the most frequent sources of homologous sequences, with proportions of 52.6% and 19.3% (Fig. S1b).

## 2.2 Families and structural features of the remaining 13 families of ESPs

The remaining 13 families of ESPs comprised a total of 24 peptides (Table 2, Additional file 1: Table S2). These peptides corresponded to 32 precursor proteins (Additional file 1: Table S3) and 36 transcripts (Additional file 1: Table S4). Among these peptides, three had been previously reported in *O. grahami* (OG) and 21 were newly identified (Fig. 2c), in which, 14 were novel peptides (OGP, OGD, OFP and Orphan). Detailed structural features of the remaining 13 families of ESPs are shown in below:

**The FARP (FMRFamide-related peptide) family:** In this study, we report the discovery of a new ESP precursor protein, pro-FMRFamide-related neuropeptide FF-OG. This protein has distinct structural features from two invertebrate neuropeptide analogs, IF-8 amide and EF-10 amide, obtained from the skin secretions of frogs *Kassina maculata* and *Phylictimantis verrucosus*, respectively [49]. However, it shares considerable sequence similarity with an uncharacterized protein in *L. catesbeianus* and the pro-FMRFamide-related neuropeptide FF in *X. tropicalis*, while showing less similarity with its counterpart in human. The three regions of the mature peptides (neuropeptide SF, FF, and AF) share highly similar structural motifs among these species, confirming its classification as a new member of this peptide family. Notably, the amino acid sequences of the three mature peptides in *L. catesbeianus* and the current species are identical (Fig. S4).

**The tachykinin family:** In this study, we report the discovery of an ESP, ranatachykinin-A, in this genus for the first time. This peptide, which has synonyms in frogs *L. catesbeianus*, *R. temporaria*, and *Theloderma corticale*, has been previously reported. [47, 48]. Although the mature peptide sequence is identical among these species, the precursor protein sequence is not identical (Fig. S5a). Additionally, two peptides of this family have been reported in this genus, one is tachykinin OG1 from *O. grahami* and the other is ranamargarin from *O. grahami* and *O. margaretae* [25, 50]. In this study, we were able to detect both peptides and confirmed that their precursor protein sequences in tachykinin OG1 are identical, while there is only a single amino acid difference for ranamargarin (Fig. S5b). Although the mature peptide region of ranamargarin and tachykinin OG1 is similar to that of ranatachykinin-A, their precursor protein sequences differ greatly (17.0% and 14.3% similarity, respectively, Fig. S5c).

**The GnRH family:** In this study, we report the discovery of an ESP precursor protein progonadoliberin-2 in this genus for the first time. Its mature peptides are Gonadoliberin-2 and GnRH-associated peptide 2. This precursor protein has also been reported in frogs *L. catesbeianus* and *R. temporaria*, with extremely high similarity, differing by only two amino acids located within the signal peptide region, respectively (Fig. S6).

**The amotoxin family:** According to the UniProt database, amotoxin is a unique ESP derived from the skin of the frog *Amolops loloensis*. In this study, we report a novel peptide named amotoxin-OG, which exhibits remarkable similarities to amotoxin. Intriguingly, both peptides share structural features with serine protease inhibitors Tabkunins found in horsefly *Tabanus yao* and Kunitz-type Protease Inhibitor 3 found in human. The mature forms of these peptides consist of six half-cystines and a conserved Kunitz-like domain (XYXGCGGN), which are commonly present in many other serine protease inhibitors [54]. Notably, the peptides mentioned here do not belong to any family in the UniProt database. Therefore, we have temporarily named it the "amotoxin family" for convenience in the description (Fig. S7).

**The NPY family:** In this study, we report the discovery of a precursor protein of an ESP, peptide YY like, in this genus for the first time. This protein has been previously detected in other frog species such as *L. catesbeianus* and *R. temporaria*. Furthermore, the mature peptide sequence is identical to that discovered in the frog *Pelophylax ridibundus* [49], although the precursor protein sequence remains unknown (Fig. S8).

**The 7B2 family:** In this study, we present the discovery of an ESP precursor protein, 7B2 granin protein, in this genus for the first time. This protein exhibits significant similarity to the protein previously reported in *P. ridibundus*, differing solely by two amino acids located within the signal peptide region. The mature peptides derived from this protein consist of two peptides, named the N-terminal peptide and the C-terminal peptide (Fig. S9) [55].

**The bombesin/neuromedin-B/ranatensin family:** Previous studies have designated five ESPs in the skin secretions of *O. grahami*, specifically as odorranain-BLP-1 to -5 [12]. In our study, we identified odorranain-BLP-4 and two novel peptides, odorranain-BLP-6 and odorranain-BLP-7, which together correspond to nine precursor proteins and 11 transcripts; four of these proteins possess partially detected N-terminal signal peptide regions. Notably, we found that one of the N-terminally truncated proteins of odorranain-BLP-4 that we discovered shares its entire protein sequence as the one which was previously reported. Overall, the precursor protein sequences of these peptides exhibit substantial similarities (Fig. S10).

**The calcitonin family:** Two ESPs, Calcitonin-like peptide 1 and Calcitonin-like peptide 2, were purified from the skin secretions of the *O. schmackeri*, and their amino acid sequences exhibit low homology with calcitonin, calcitonin gene-related peptide (CGRP), and adrenomedullin from other vertebrates. However, these peptides do not completely meet all the criteria of any of the mentioned peptides [56], suggesting that they may belong to a novel subfamily. In our investigation, we have identified a novel peptide, calcitonin-like peptide 1-OG1, which shows significant similarity to these peptides, confirming that they are not isolated peptides. In addition, the peptides we identified also contain precursor protein sequences, thus, this discovery enhances our understanding of the complete structural feature of these peptides and provides further evidence for the existence of a novel subfamily within the calcitonin family (Fig. S11).

**The cathelicidin family:** In this study, we report two new ESPs, OG-CATH1 and OG-CATH2, with OG-CATH1 corresponding to two precursor proteins. These peptides are similar to OL-CATH1 and OL-CATH2 from the skin of *O. livida*, and Cathelicidin-RC1 from frog *L. catesbeianus* (Fig. S12) [57]. However, they show low similarity (20.3%, data not shown) to Cathelicidin-OA1 from the skin of *O. andersonii* [58]. The sequence differences may account for the antimicrobial activity of OL-CATH1 and OL-CATH2. In contrast, Cathelicidin-OA1 lacks antimicrobial activity but exhibits antioxidant properties and accelerates skin wound healing.

**The Galanin Family:** In this study, we report the first discovery of an ESP, galanin, in this genus. This peptide has previously been detected in other frog species, such as *L. catesbeianus* and *P. ridibundus*. Notably, A0A2G9NC14_LITCT from *L. catesbeianus* contains an additional sequence (MLAGSTCSTPLQDSRDRNCKHTYPNQLK) at the N-terminal signal peptide region when compared to its mammalian counterpart in bovine (GALA_BOVIN). SignalP predicted that this sequence had a signal peptide probability of only 0.0966, which increased to 0.9741 when the sequence was removed. Consequently, this sequence is likely a “translated” 5’-UTR that was incorrectly assigned to the predicted precursor protein sequence and forms an artificially elongated ORF_shift protein. Notably, this protein, including the 5’-UTR, displays extremely high similarity between *O. grahami* and *L. catesbeianus*, with only one amino acid difference at the C-terminus (Fig. S13).

**The vasopressin/oxytocin family:** In this study, we present the discovery of an ESP precursor protein, mesotocin-neurophysin_MT, in this genus for the first time. This protein exhibits significant similarity to the protein previously reported in *N. parkeri* and *R. temporaria*, differing by only one and two amino acids located within the signal peptide region, respectively. The final mature peptides derived from this protein consist of Mesotocin and Neurophysin MT, with the Mesotocin also being identical to that found in *B. japonicus* (Fig. S14).

**The insulin family:** In this study, we report the first discovery of an ESP, insulin-like growth factor II isoform X2, in *O. grahami*. This peptide has previously been detected in the frog *R. temporaria*. Apart from the first few amino acids at the N-terminus of the peptide, the rest of the amino acid sequence has high similarity to that in *X. laevis* and chicken (Fig. S15).

**The intercrine alpha (chemokine CxC) family:** In this study, we report the discovery of seven novel ESPs that can be classified into four groups based on amino acid sequence similarity: C-X-C motif chemokine 8-OG1 and 8-OG2 (Fig. S16a), C-X-C motif chemokine 10-OG1 and 10-OG2 (Fig. S16b), C-X-C motif chemokine 11-OG1 and 11-OG2 (Fig. S16c), and C-X-C motif chemokine 14-OG1 (Fig. S16d). Our classification of C-X-C motif chemokine 8 and 14 agrees with their closest sequences of C-X-C motif chemokines found in both frogs (A0A1L8HMI3_XENLA from *X. laevis* and C1C478_LITCT from *L. catesbeianus*, respectively) and mammals (IL8_CHICK from chicken and CXL14_MOUSE from mouse, correspondingly). However, for the remaining two groups, C-X-C motif chemokine 10 and 11, the most comparable sequences discovered in frogs are assigned to the C-X-C motif chemokine 8 group (A0A8T2KED6_9PIP from *Hymenochirus boettgeri* and A0A2G9RDX0_LITCT from *L. catesbeianus*, respectively). As these two sequences have only a low similarity with A0A1L8HMI3_XENLA (25% and 21%, respectively, data not shown), we classified these two groups according to their most analogous mammalian counterparts (CXL10_BOVIN and CXL11_BOVIN, both from bovine).

## 2.3 Antimicrobial activity of ranatuerin-2P-OG1

The ranatuerin-2P-OG1 is a novel ESP of the FSAP family, the only one that was identified at the protein level but not recognized as a secretory precursor protein at the transcript level due to the undetected signal peptide region (Additional file 1: Tables S3-S4). Antimicrobial activity assays showed that ranatuerin-2P-OG1 exhibited broad-spectrum antimicrobial activity (Table Sb). This is consistent with the feature of FSAP family peptides, which often have antimicrobial activity.

# Tables

## Table Sa. Transcriptome annotation items generated by the Trinotate pipeline.

|  | Genes | Transcripts | Proteins |
| --- | --- | --- | --- |
| Total | 167425 | 217270 | 181817 |
| ORF | 98585 | 134716 | 181817 |
| ORF % | 58.9 | 62.0 | 100% |
| Blast | 45280 | 67900 | 70510 |
| Blast % | 27.0 | 31.3 | 38.8 |
| GO | 35849 | 54779 | 55524 |
| GO % | 21.4 | 25.2 | 30.5 |
| KEGG | 26609 | 41475 | 41412 |
| KEGG % | 15.9 | 19.1 | 22.8 |
| Pfam | 25111 | 39912 | 41970 |
| Pfam % | 15.0 | 18.4 | 23.1 |
| SignalP | 5205 | 7304 | 7511 |
| SignalP % | 3.1 | 3.4 | 4.1 |
| TmHMM | 12929 | 17919 | 19226 |
| TmHMM % | 7.7 | 8.2 | 10.6 |
| RNAMMER | 15 | 28 | / |
| RNAMMER % | 0.01 | 0.01 | / |
| Sum annotation | 53229 | 77617 | 84212 |
| Sum annotation % | 31.8 | 35.7 | 46.3 |

## Table Sb. The minimum inhibitory concentrations (MICs) of ranatuerin-2P-OG1 (μg/mL).

|  | *Staphylococcus aureus* | MRSA | *Enterococcus faecalis* | *Escherichia coli* | *Pseudomonas aeruginosa* | *Candida albicans* |
| --- | --- | --- | --- | --- | --- | --- |
| ranatuerin-2P-OG1 | 64 | 64 | 256 | 128 | ND | 512 |
| Ampicillin | 1 | 128 | 2 | 8 | ND |  |
| Amphotericin B |  |  |  |  |  | 2 |

Note: Ampicillin and Amphotericin B are conventional antibiotics. ND means no detectable activity at the highest test concentration. The highest test concentration for ranatuerin-2P-OG1 was 512 μg/mL and for Ampicillin was 128 μg/mL.

# Figures


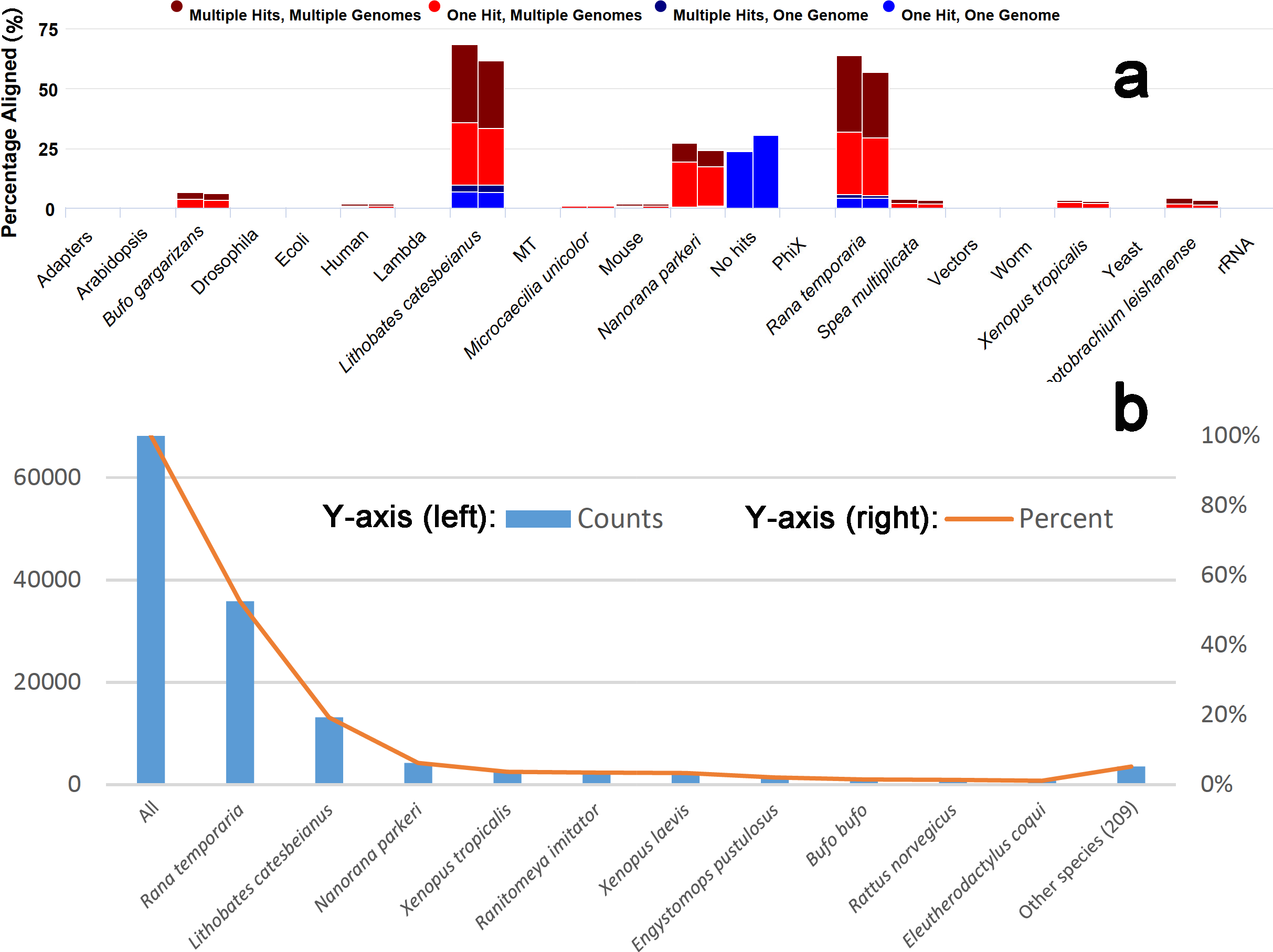


## Figure S1. Quality assessment and cross-species sequence conservation analyses.

(a) FastQ Screen contamination screening showed minimal alignments (0-0.2%) of the trimmed reads to common contaminant sequences such as Drosophila, Worm, Yeast, Arabidopsis, Ecoli, rRNA, MT (mitochondria), PhiX, Lambda, Vectors, and Adapters and highest alignment rates to *R. temporaria* (63.6%) and *L. catesbeianus* (68.2%) among eight amphibian reference genomes including *Xenopus tropicalis, Leptobrachium leishanense, Bufo gargarizans, Rana temporaria, Nanorana parkeri, Lithobates catesbeianus, Spea multiplicata, and Microcaecilia unicolor*. (b) BLASTP homology analysis of TransDecoder proteins against the nr sub-database of Amphibia revealed *R. temporaria* (52.6%) and *L. catesbeianus* (19.3%) as the predominant taxonomic sources of homologous sequences.


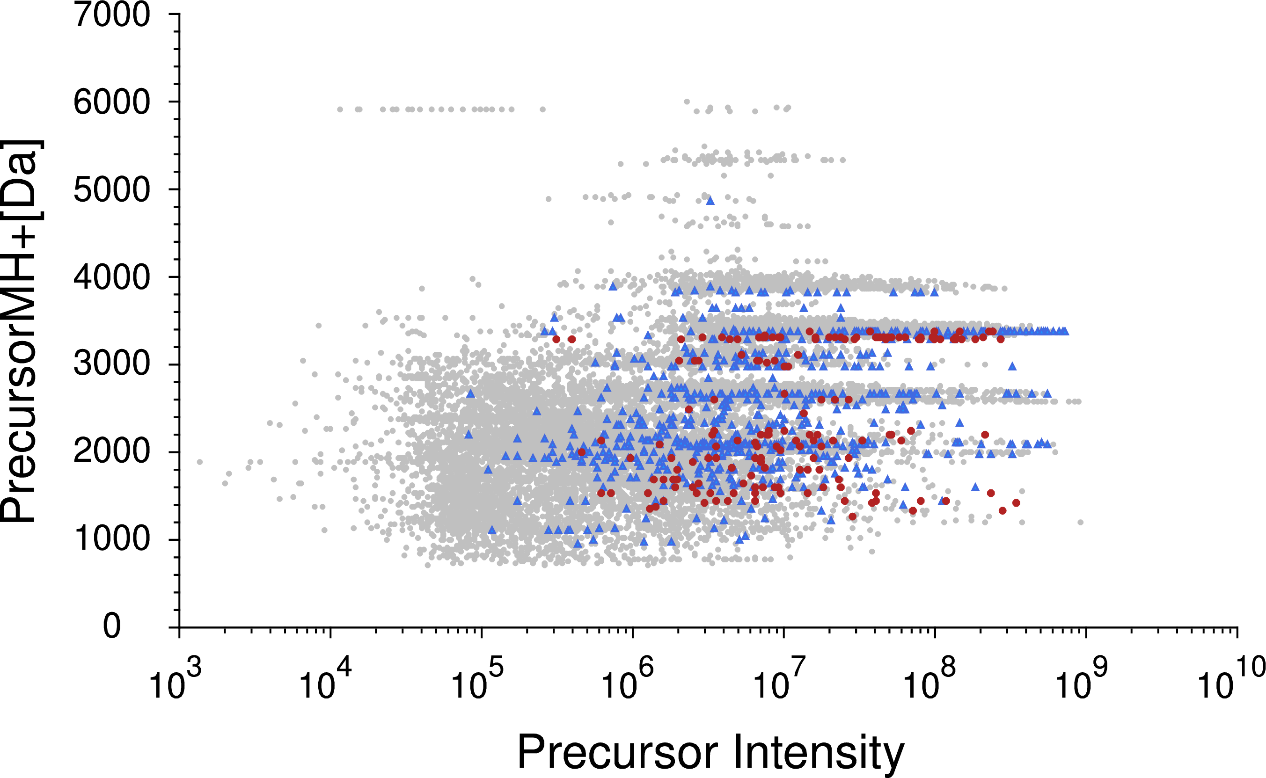


## Figure S2. Precursor detection by LC-MS/MS.

This figure shows a total of 13,335 precursors sacned by LC-MS/MS. The colored dots (867) represent the precursors with high confidence matched to peptide-spectrum matches (PSMs) (1,054), with blue dots indicating the precursors matched to one PSM, and red dots indicating the precursors matched to more than one PSM. The gray dots highlight the remaining unmatched precursors.


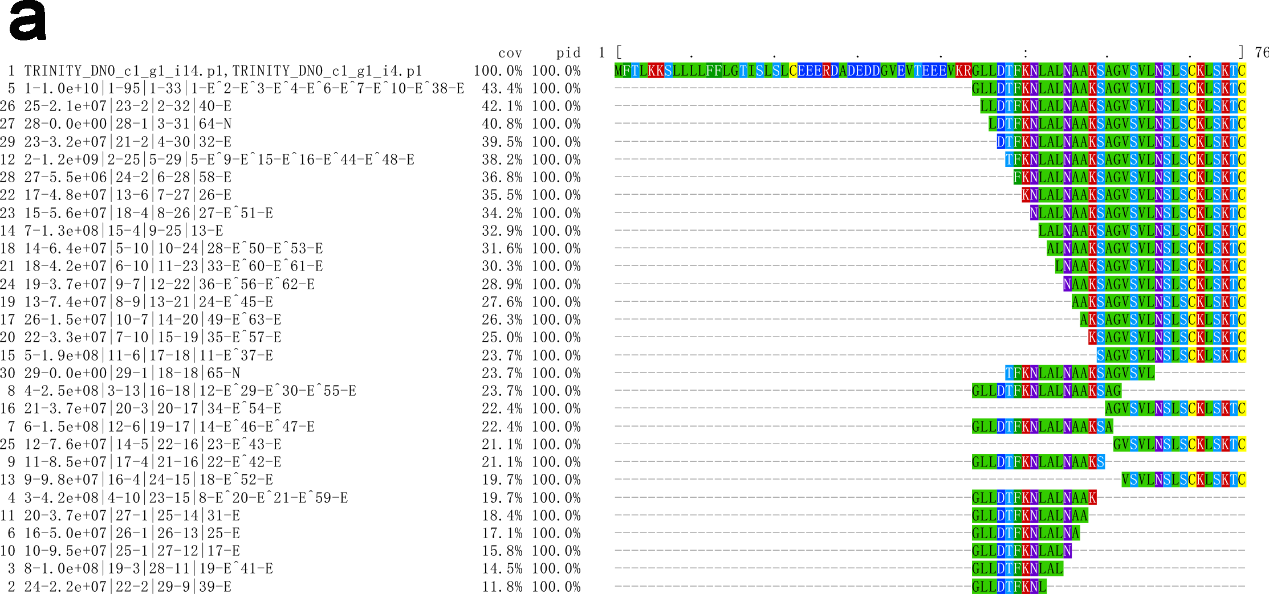


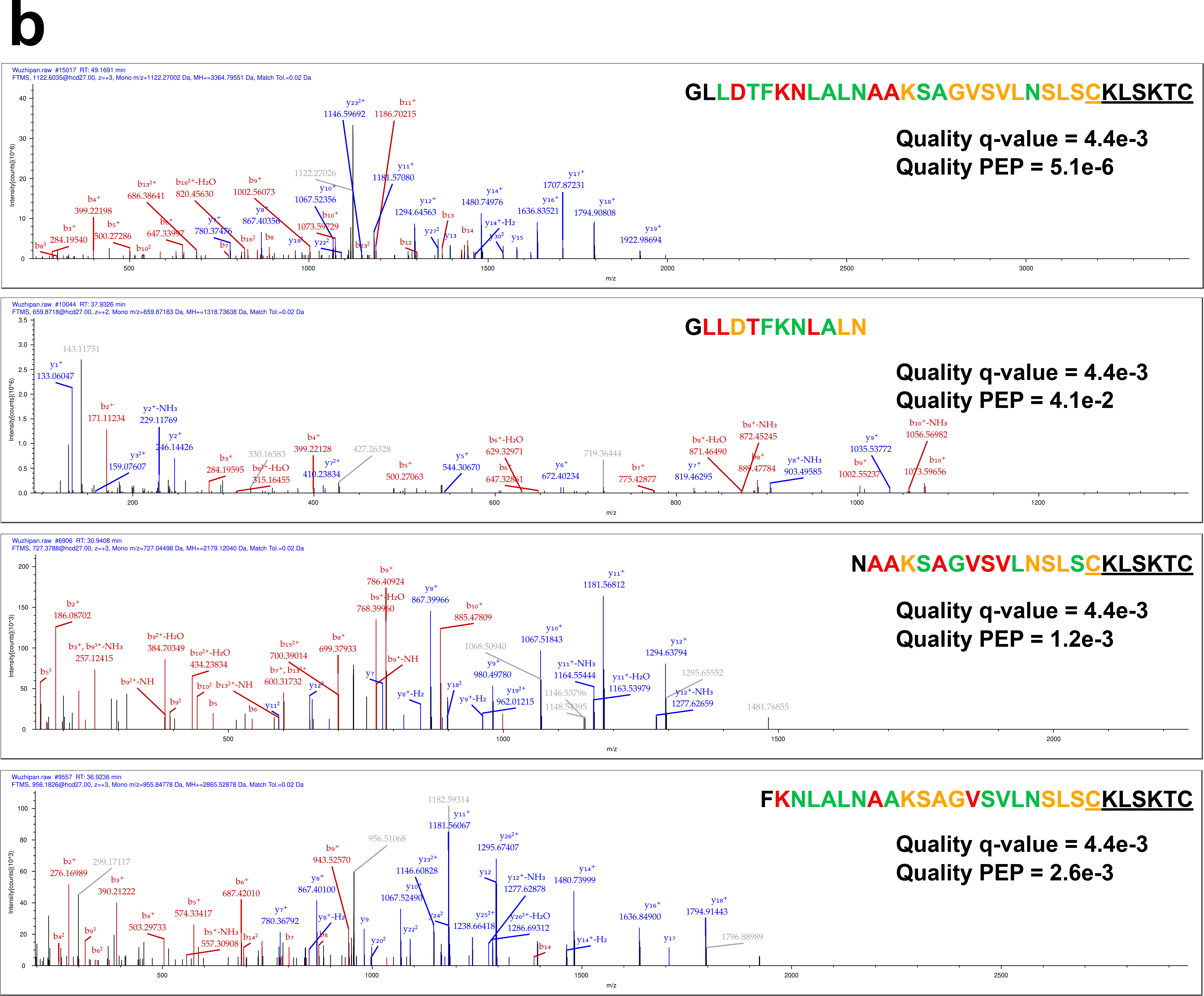


## Figure S3. Overview of mature peptide and its truncated peptides identified by LC-MS/MS.

(a) Sequence mapping of brevinin-2GRa and its truncations, all detected by mass spectrometry, onto corresponding master protein. The first column displays the peptide labels: Abundance rank (descending) - Peptide abundance | PSM count rank (descending) - PSM count | Length rank (descending) - Peptide length | Abundance rank of Isomer 1 (descending) - Quantification type (Isomer 1) ^ ... Abundance rank of Isomer N - Quantification type (Isomer N). Quantification type is denoted by E for exclusive peptides, N for "No Quan Values" or "Not Reliable" in the "Quan Info" column, and S for "Shared" in the "Quan Info" column, followed by Coverage (Cov) and Identity (Pid). The rectangle box indicates the signal peptide, the solid line indicates the mature peptide, and the remaining portion comprises an acidic propiece with a predicted proprotein convertase cleavage site at its C-terminal single or paired basic residues (predominantly Lys-Arg dipeptide motifs). The first row represents the master protein, which is the longest one in ORF_shift proteins if present. The second row represents the mature peptide, which exhibits higher abundance and greater length compared to its truncated peptides (as shown in the peptide labels). This peptide also conforms to the structural motif of the FSAP family. The remaining rows show the truncated peptides of the brevinin-2GRa. Brevinin-2GRa has the highest abundance among all mature peptides detected in mass spectrometry and also has the most truncated peptides (28 truncated peptides). Other ESPs (Table 1) and their respective truncated peptides can be found in Additional file 4.

(b) Representative annotated MS/MS spectra of brevinin-2GRa and its truncations. Spectra at the peptide group level are vertically ordered by the abundance ranking system defined in panel (a), with labels sampled at every 9th rank interval: 1 (highest, brevinin-2GRa), 10, 19, and 28. In the figure, Color coding of amino acid residues: red (*b*-ions annotated at these positions), yellow (*y*-ions annotated), green (both *b*- and *y*-ions annotated), black (no fragment ion matches); underlining indicates the disulfide ring (Rana box), with near absence of fragment ions (including but not limited to *b*/*y* ions) from this region. Quality q-value: The false discovery rate (FDR) for the peptide group is indicated (q < 0.01); Quality PEP: The posterior error probability (PEP) for the peptide group is shown (PEP < 0.05). The native Proteome Discoverer result file (SPS.pdResult) is provided in Data S2 of [44]. Protein and peptide isoforms identified from this file, along with annotated MS/MS spectra at the PSM level for the 16 ESPs and their truncations (Table 1), are provided in Additional file 3 (SPS folder) as exported datasets: Search_file_export.xlsx (tabular data) and Annotated_Spectra.zip (spectral annotations).


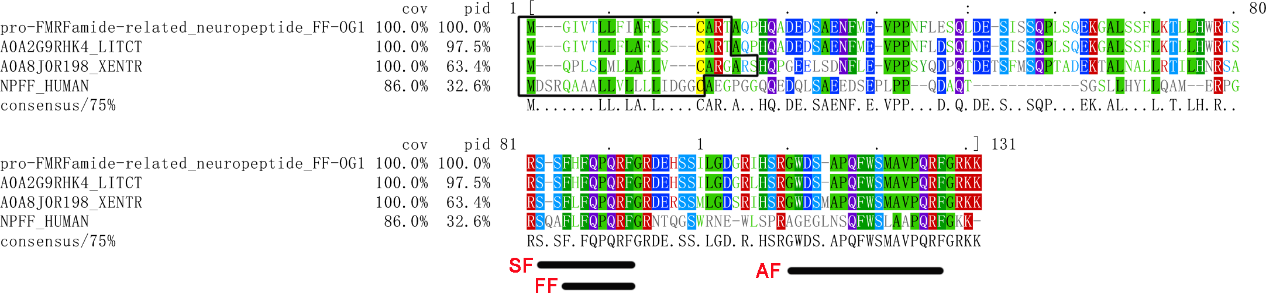


## Figure S4. The FARP (FMRFamide-related peptide) family.

The first line of this figure shows the new ESP precursor protein, pro-FMRFamide-related neuropeptide FF-OG1. The subsequent lines list proteins belonging to this family from different species, arranged from top to bottom for *L. catesbeianus*, *X. tropicalis*, and human species with their UniProt entry names. This protein is composed of three final mature peptides: neuropeptide SF, FF, and AF. Percent coverage (%) is represented by Cov and percent identity (%) is denoted by Pid. The signal peptide is highlighted in a rectangle box, while the mature peptide is marked by a solid line. This applies to all figures below.


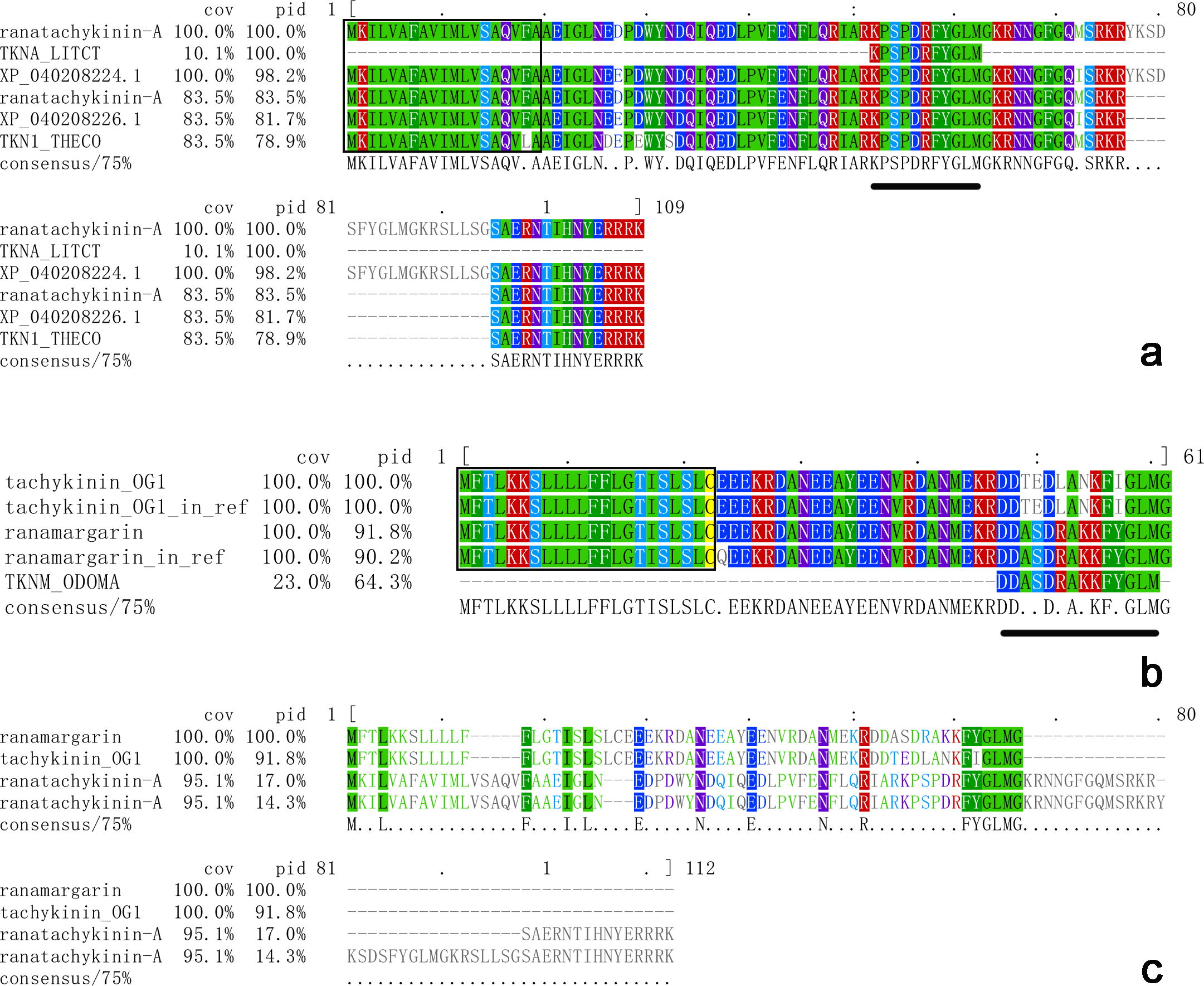


## Figure S5. The tachykinin family.

(a) The first and fourth lines of this figure show an ESP, ranatachykinin-A, with its precursor proteins, which was discovered in this genus for the first time. The remaining lines list the same peptide with its precursor proteins if present from *L. catesbeianus* on the second line, *R. temporaria* on the third and fifth lines with RefSeq accession numbers, and *T. corticale* on the sixth line. (b) The ranamargarin and tachykinin OG1 with their precursor proteins in this study. The suffix “_in_ref” indicates sequences that have already been reported in *O. grahami*. The last line shows the sequence reported in *O. margareta*. (c) The precursor protein sequences of ranamargarin or tachykinin OG1 have very low similarity to that of ranatachykinin-A (17.0% and 14.3%, respectively). We will further discuss in the following text that ranamargarin and tachykinin OG1 should belong to the FSAP family.


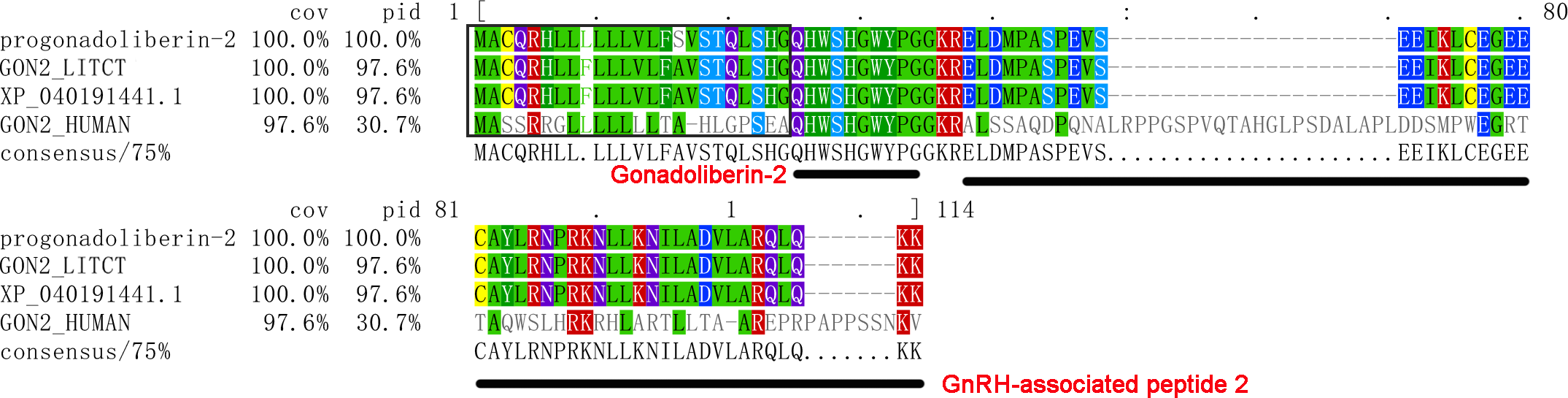


## Figure S6. The GnRH family.

The first line of this figure shows the ESP precursor protein progonadoliberin-2, which was discovered in this genus for the first time. The subsequent lines list proteins belonging to this family from different species, arranged from top to bottom for *L. catesbeianus* and *R. temporaria* with RefSeq accession numbers, and human species. This protein is composed of two final mature peptides: Gonadoliberin-2 and GnRH-associated peptide 2.


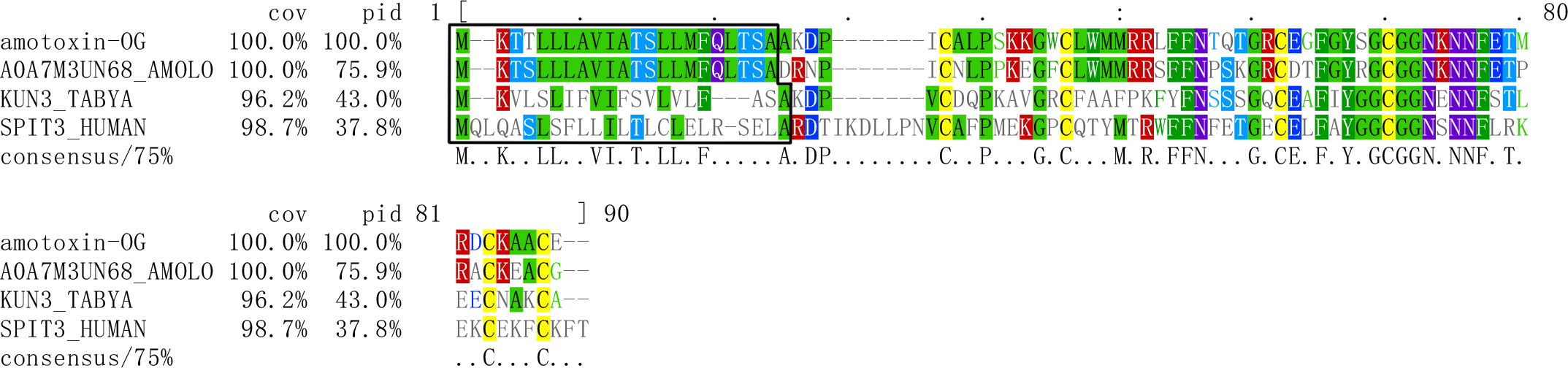


## Figure S7. The amotoxin family.

The top row of this figure displays the precursor protein for a novel ESP, amotoxin-OG. The second row shows the amotoxin from *Amolops loloensis*, belonging to this family. Subsequent rows list proteins similar to it from various species, with horsefly *Tabanus yao* and human species arranged top to bottom. The mature peptide comprises the remaining sequence, except for the signal peptide.


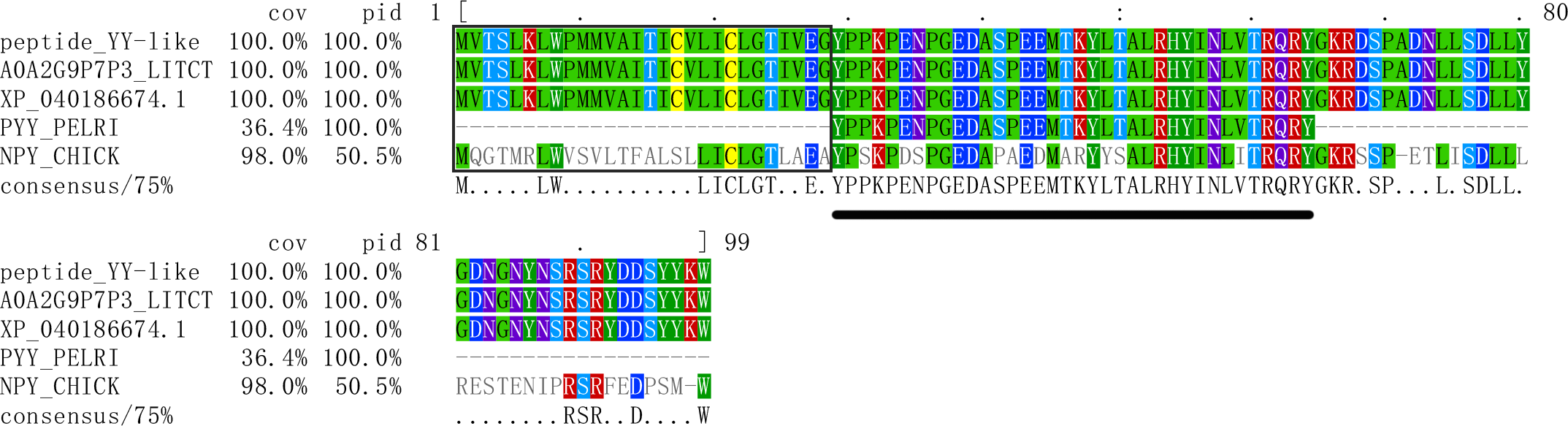


## Figure S8. The NPY family.

The first line of this figure shows the precursor protein of an ESP, peptide YY-like, which was discovered in this genus for the first time. The last line shows a similar protein from chicken. The remaining lines list the same peptide or proteins from different species of Anura order, arranged from top to bottom for *L. catesbeianus*, *R. temporaria* with RefSeq accession numbers, and *Pelophylax ridibundus*.


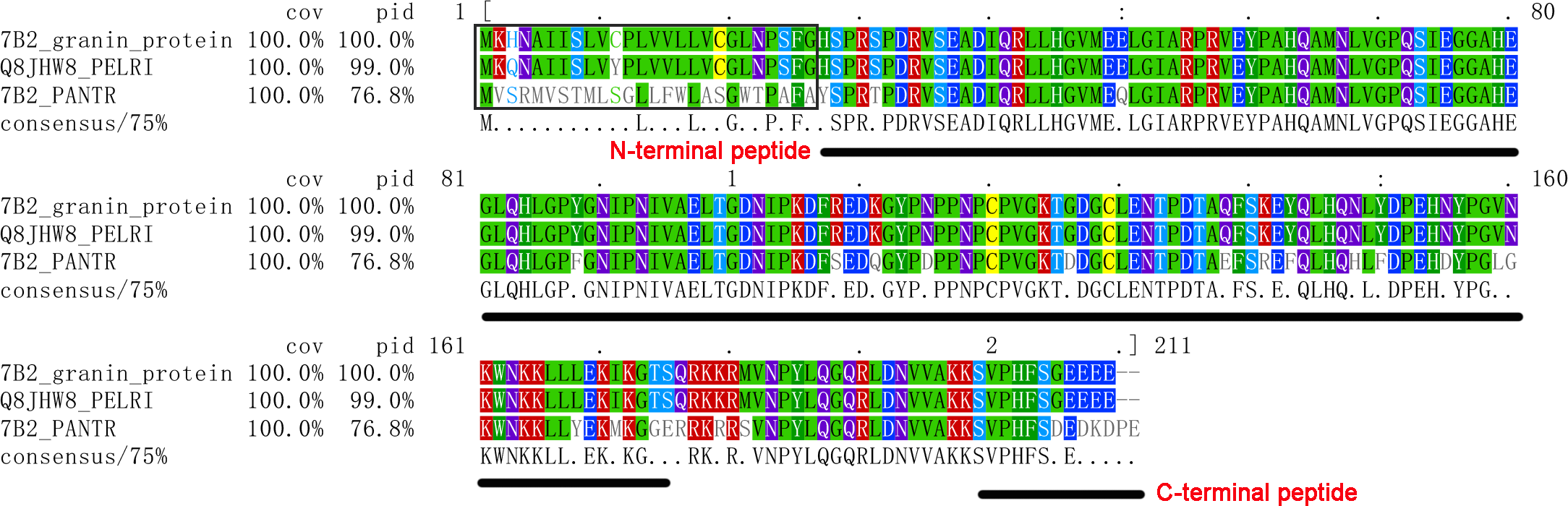


## Figure S9. The 7B2 family.

The first line of this figure shows the ESP precursor protein 7B2 granin protein, which was discovered in this genus for the first time. The subsequent lines list proteins belonging to this family from different species, arranged from top to bottom for *P. ridibundus* and chimpanzee species. This protein is composed of two final mature peptides named the N-terminal peptide and the C-terminal peptide.


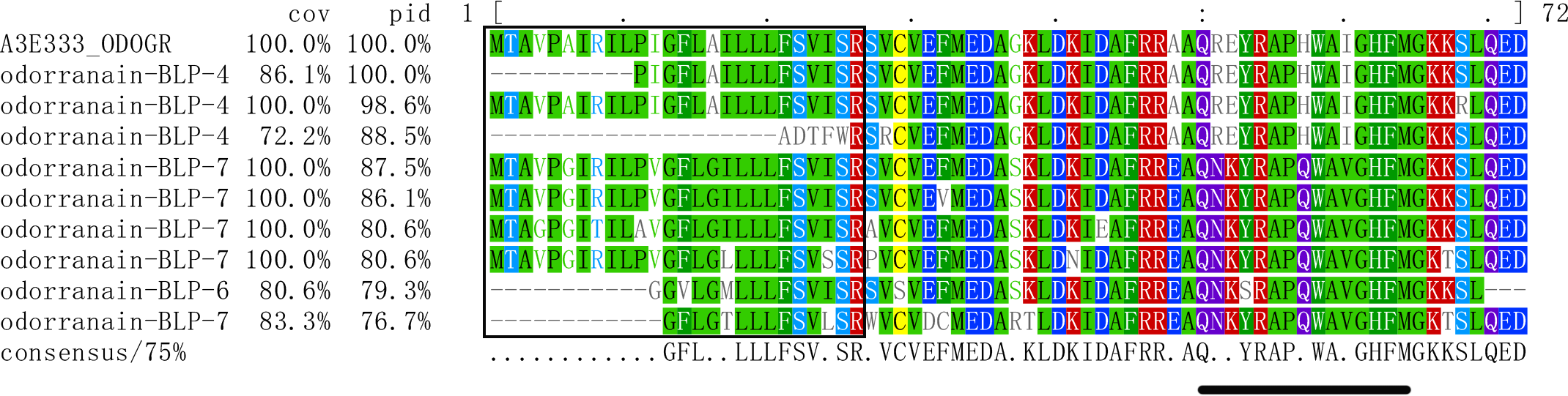


## Figure S10. The bombesin/neuromedin-B/ranatensin family.

The top row of this figure illustrates the precursor protein of an ESP, odorranain-BLP-4, previously reported in *O. grahami*. The subsequent rows list the same peptide and two novel peptides (odorranain-BLP-6 and odorranain-BLP-7) discovered in this study, which corresponds to a total of nine proteins, four of which possess partially detected N-terminal signal peptide regions.


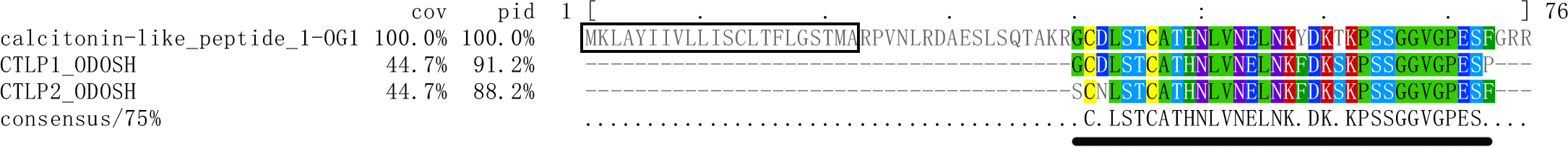


## Figure S11. The calcitonin family.

The top row of this figure displays the precursor protein for a novel ESP, calcitonin-like peptide 1-OG1. The subsequent rows list other peptides from the same family, arranged from top to bottom for Calcitonin-like peptide 1 and Calcitonin-like peptide 2, both found in *O. schmackeri*.


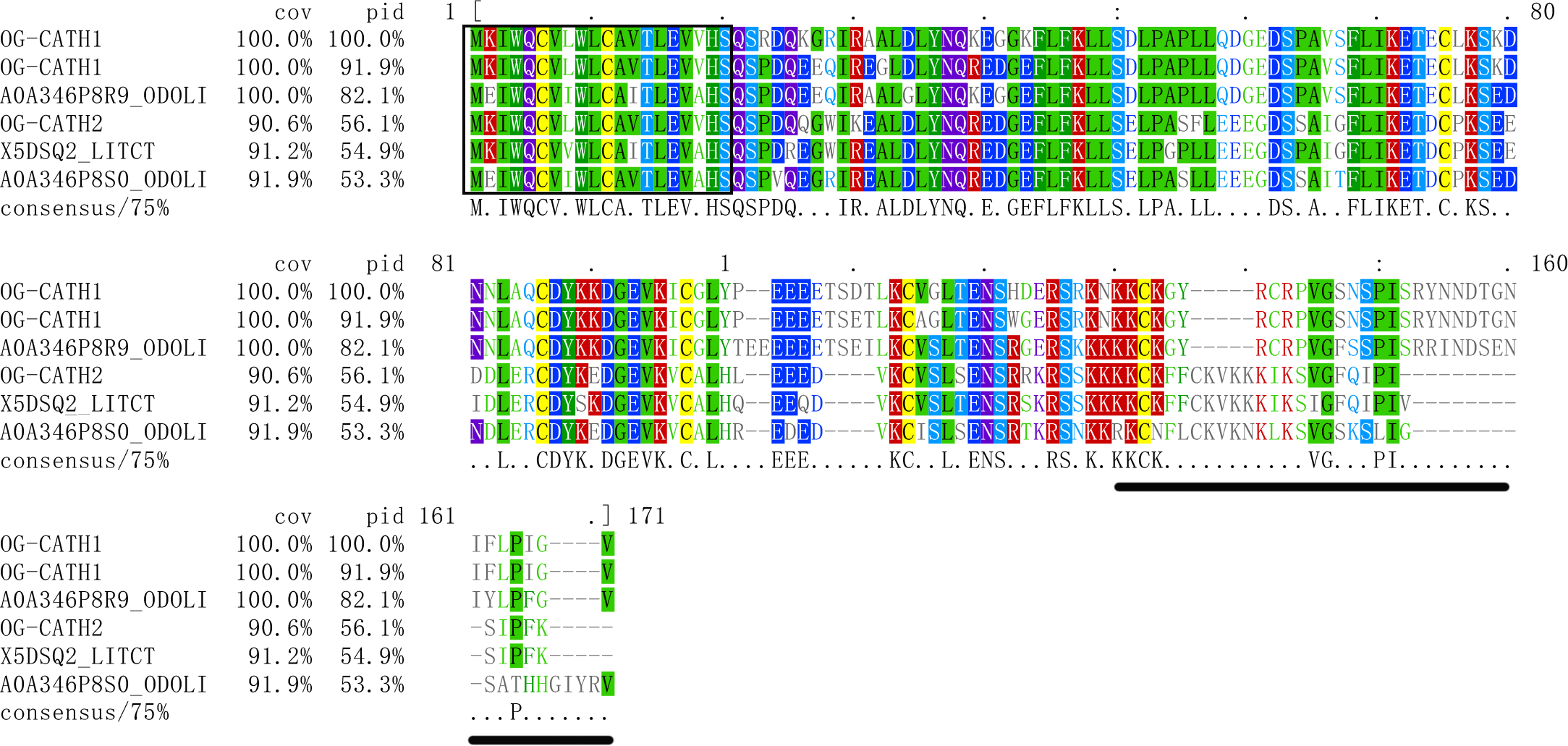


## Figure S12. The cathelicidin family.

This figure displays the precursor proteins of two novel ESPs, OG-CATH1 and OG-CATH2. The subsequent rows list proteins from various species that belong to the same family. Specifically, the fifth row shows Cathelicidin-RC1 from *L. catesbeianus*, while the third and sixth rows depict OL-CATH1 and OL-CATH2, respectively, both from *O. livida*.


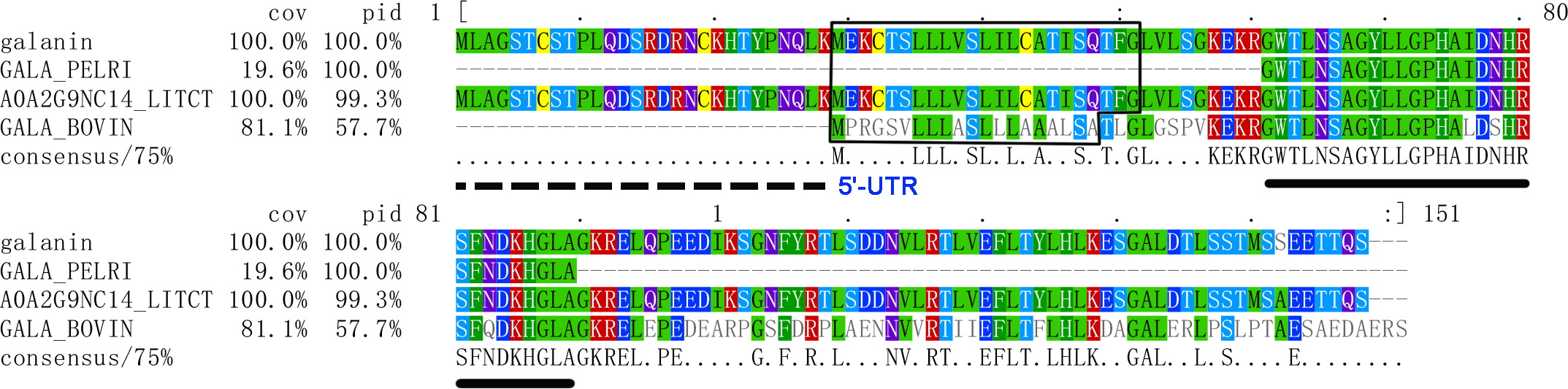


## Figure S13. The Galanin family.

The first row of this figure displays the precursor protein of an ESP galanin, which was discovered in this genus for the first time. The final row shows a similar protein from bovine. The remaining rows list the same peptide with its associated protein, if present, from various species, arranged from top to bottom for *P. ridibundus* and *L. catesbeianus* species. Notably, A0A2G9NC14_LITCT contains an additional sequence (dashed line segment) at the N-terminal signal peptide region when compared to its mammalian counterpart in bovine, GALA_BOVIN. SignalP predicted that this sequence had a signal peptide probability of only 0.0966, which increased to 0.9741 when the segment was removed. Consequently, it is highly likely that this sequence is a “translated” 5’-UTR that was incorrectly assigned to the predicted protein sequence and forms an artificially elongated ORF_shift protein.


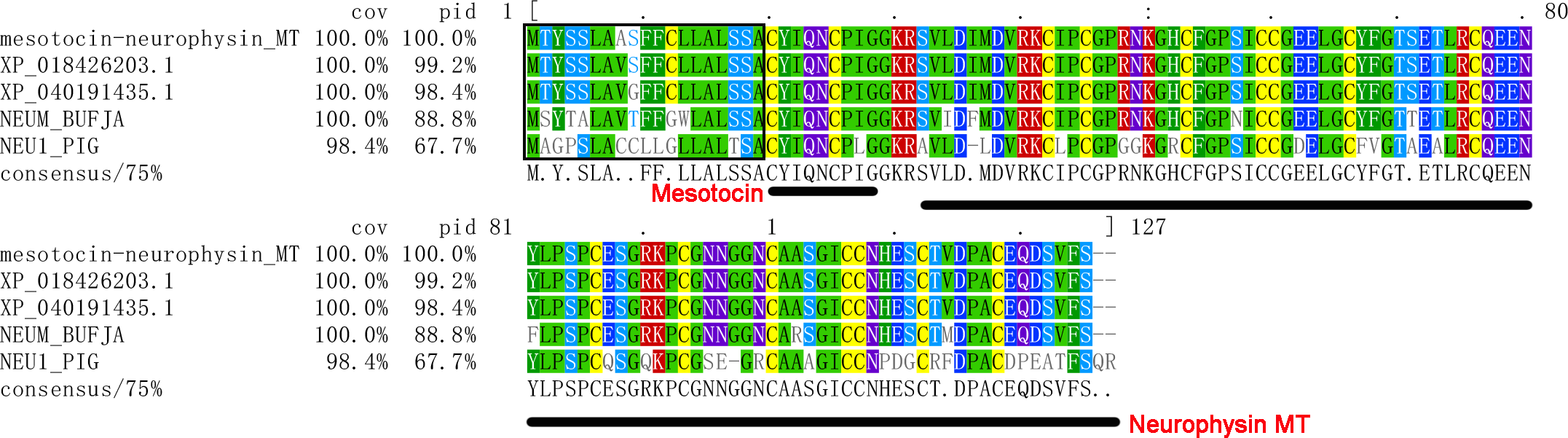


## Figure S14. The vasopressin/oxytocin family.

The first row of this figure displays the ESP precursor protein mesotocin-neurophysin_MT, which was newly discovered in this genus. The subsequent rows list proteins belonging to this family from various species, arranged from top to bottom for *N. parkeri* with RefSeq accession numbers, *R. temporaria* with RefSeq accession numbers, *B. japonicus* and pig species. This protein is composed of two final mature peptides: Mesotocin and Neurophysin MT.


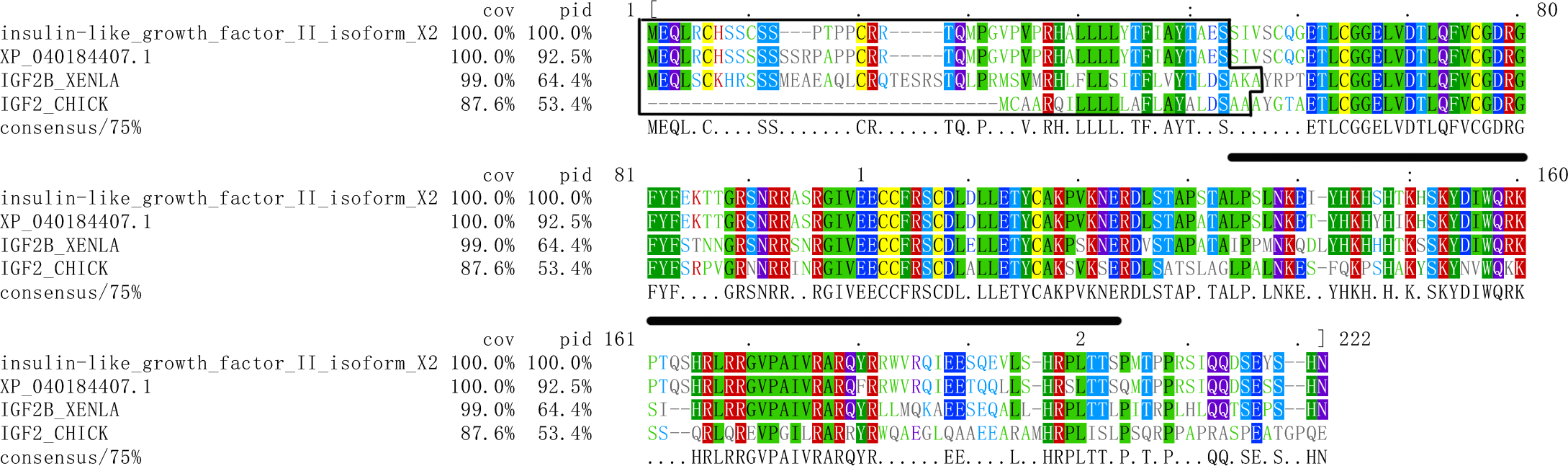


## Figure S15. The insulin family.

The first row of this figure displays the precursor protein of an ESP insulin-like growth factor II isoform X2, which was discovered in this genus for the first time. The subsequent rows list proteins belonging to this family from various species, arranged from top to bottom for *R. temporaria* with RefSeq accession numbers, *X. laevis* and chicken species.


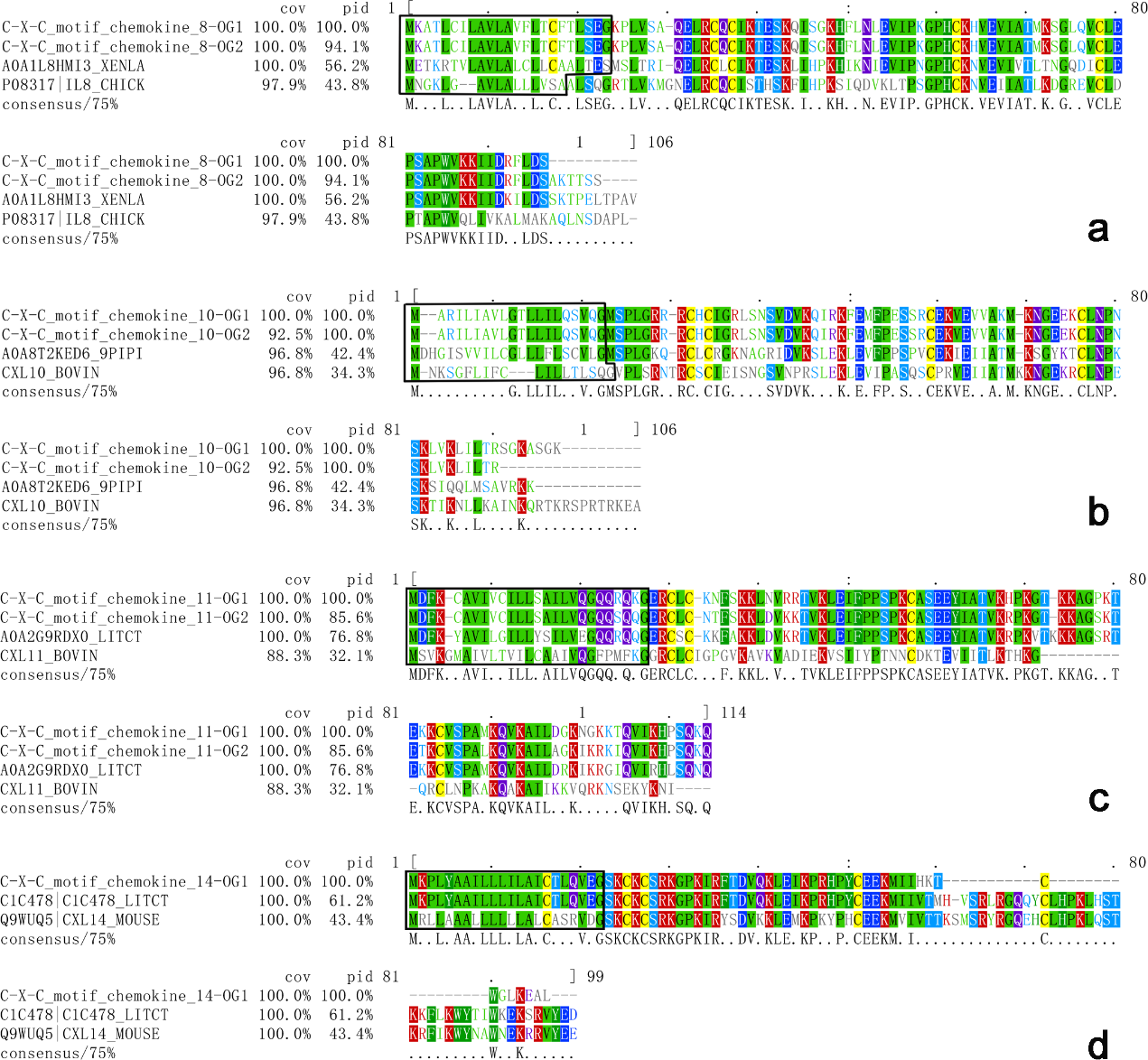


## Figure S16. The intercrine alpha (chemokine CxC) family.

(a) The top two rows of this figure display the precursor protein for two novel ESPs, C-X-C motif chemokine 8-OG1 to 2. The subsequent rows list proteins belonging to this family from various species, arranged from top to bottom for *X. laevis* and chicken species.

(b) The top two rows of this figure display the precursor protein for two novel ESPs, C-X-C motif chemokine 10-OG1 to 2. The subsequent rows list proteins belonging to this family from various species, arranged from top to bottom for *H. boettgeri* and bovine species.

(c) The top two rows of this figure display the precursor protein for two novel ESP, C-X-C motif chemokine 11-OG1 to 2. The subsequent rows list proteins belonging to this family from various species, arranged from top to bottom for *L. catesbeianus* and bovine species.

(d) The top row of this figure displays the precursor protein for a novel ESP, C-X-C motif chemokine 14-OG1. The subsequent rows list proteins belonging to this family from various species, arranged from top to bottom for *L. catesbeianus* and mouse species.

# Parameters

## Parameter S1: Parameters used in pFind.

MS Instrument: HCD-FTMS

Mixture Spectra: True

Enzyme: NoEnzymeU_C and Non-specific

Precursor Tolerance: 3 ppm

Fragment Tolerance: 0.02 Da

Open Search: True

FDR <=1 %

350 Da <= Peptide Mass <= 6000 Da

6 <= Peptide Length <= 144

Number of Peptides >= 1

## Parameter S2: Parameters used in PD.

+++++++++++++++++++++++++++++++++++++++++++++++++++++

Processing Step A: Workflow

------------------------------------------------------------------

The workflow tree:

------------------------------------------------------------------

|-(6) Spectrum Files

|-(5) Minora Feature Detector

|-(1) Spectrum Selector

|-(2) Precursor Detector

|-(3) Sequest HT

|-(4) Percolator

|-(7) IMP-ptmRS

------------------------------------------------------------------

Processing node 6: Spectrum Files

------------------------------------------------------------------

Input Data:

- File Name(s): Z:\质谱\Wuzhipan.raw

------------------------------------------------------------------

Processing node 5: Minora Feature Detector

------------------------------------------------------------------

1. Peak & Feature Detection:

- Min. Trace Length: 5

- S/N Threshold: 1

- Max. ΔRT of Isotope Pattern Multiplets [min]: 0.2

2. Feature to ID Linking:

- PSM Confidence At Least: High

------------------------------------------------------------------

Processing node 1: Spectrum Selector

------------------------------------------------------------------

1. General Settings:

- Precursor Selection: Use MS1 Precursor

- Use Isotope Pattern in Precursor Reevaluation: True

- Provide Profile Spectra: Automatic

2. Spectrum Properties Filter:

- Lower RT Limit: 0

- Upper RT Limit: 0

- First Scan: 0

- Last Scan: 0

- Lowest Charge State: 1

- Highest Charge State: 0

- Min. Precursor Mass: 350 Da

- Max. Precursor Mass: 6000 Da

- Total Intensity Threshold: 0

- Minimum Peak Count: 1

3. Scan Event Filters:

- Mass Analyzer: Is FTMS

- MS Order: Is Not MS1

- Activation Type: Is HCD

- Min. Collision Energy: 0

- Max. Collision Energy: 1000

- Scan Type: Is Full

- Polarity Mode: Is +

4. Peak Filters:

- S/N Threshold (FT-only): 1.5

5. Replacements for Unrecognized Properties:

- Unrecognized Charge Replacements: Automatic

- Unrecognized Mass Analyzer Replacements: ITMS

- Unrecognized MS Order Replacements: MS2

- Unrecognized Activation Type Replacements: CID

- Unrecognized Polarity Replacements: +

- Unrecognized MS Resolution@200 Replacements: 60000

- Unrecognized MSn Resolution@200 Replacements: 30000

6. Precursor Pattern Extraction:

- Precursor Clipping Range Before: 2.5 Da

- Precursor Clipping Range After: 5.5 Da

------------------------------------------------------------------

Processing node 2: Precursor Detector

------------------------------------------------------------------

1. General:

- S/N Threshold: 1.5

- S/N Threshold for most abundant peak: 1.5

------------------------------------------------------------------

Processing node 3: Sequest HT

------------------------------------------------------------------

1. Input Data:

- Protein Database: Search_file.fasta

- Enzyme Name: No-Enzyme (Unspecific)

- Max. Missed Cleavage Sites: 2

- Min. Peptide Length: 6

- Max. Peptide Length: 144

- Max. Number of Peptides Reported: 10

2. Tolerances:

- Precursor Mass Tolerance: 3 ppm

- Fragment Mass Tolerance: 0.02 Da

- Use Average Precursor Mass: False

- Use Average Fragment Mass: False

3. Spectrum Matching:

- Use Neutral Loss a Ions: True

- Use Neutral Loss b Ions: True

- Use Neutral Loss y Ions: True

- Use Flanking Ions: True

- Weight of a Ions: 0

- Weight of b Ions: 1

- Weight of c Ions: 0

- Weight of x Ions: 0

- Weight of y Ions: 1

- Weight of z Ions: 0

4. Dynamic Modifications:

- Max. Equal Modifications Per Peptide: 2

- Max. Dynamic Modifications Per Peptide: 4

- 1. Dynamic Modification: Oxidation / +15.995 Da (M)

- 2. Dynamic Modification: Deamidated / +0.984 Da (N)

- 3. Dynamic Modification: Dehydro / -1.008 Da (C)

6. Dynamic Modifications (protein terminus):

- 1. N-Terminal Modification: Acetyl / +42.011 Da (N-Terminus)

- 2. N-Terminal Modification: Met-loss / -131.040 Da (M)

- 3. N-Terminal Modification: Met-loss+Acetyl / -89.030 Da (M)

------------------------------------------------------------------

Processing node 4: Percolator

------------------------------------------------------------------

1. Target/Decoy Strategy:

- Target/Decoy Selection: Separate

- Validation based on: q-Value

2. Input Data:

- Maximum Delta Cn: 0.05

- Maximum Rank: 0

3. FDR Targets:

- Target FDR (Strict): 0.01

- Target FDR (Relaxed): 0.05

------------------------------------------------------------------

Processing node 7: IMP-ptmRS

------------------------------------------------------------------

1. Scoring:

- PhosphoRS Mode: False

- Report Only PTMs: False

- Use Diagnostic Ions: True

- Use Fragment Mass Tolerance of Search Node: True

- Fragment Mass Tolerance: 0.5 Da

- Consider neutral loss peaks for CID, HCD and EThcD: Automatic

- Maximum Peak Depth: 8

- Use a mass accuracy correction: True

2. Performance:

- Maximum Number of Position Isoforms: 25

- Maximum PTMs per peptide: 10

+++++++++++++++++++++++++++++++++++++++++++++++++++++

Consensus Step : Workflow

Result name: Search_file

Result file: D:\Search_file.pdResult

Description: Result filtered for high confident peptides, with enhanced peptide and protein annotations. Add FASTA file with common contaminants to the Protein Marker node. Quan abunaces are normalized to the same total peptide amount per channel and scaled, so that the average abunce per protein and peptide is 100.

Workflow based on template: CWF_Comprehensive_Enhanced Annotation_LFQ_and_Precursor_Quan

Creation date: 6/12/2022 12:17:31 PM

Created with Discoverer version: 2.5.0.400

------------------------------------------------------------------

The workflow tree:

------------------------------------------------------------------

|-(0) MSF Files

|-(10) Feature Mapper

|-(11) Precursor Ions Quantifier

|-(1) PSM Grouper

|-(2) Peptide Validator

|-(3) Peptide and Protein Filter

|-(4) Protein Scorer

|-(7) Protein FDR Validator

|-(5) Protein Grouping

|-(6) Peptide in Protein Annotation

|-(15) Modification Sites

|-(9) Protein Marker

|-(16) Peptide Isoform Grouper

Post-processing nodes:

--------------------------------

|-(12) Result Statistics

|-(13) Display Settings

|-(14) Data Distributions

------------------------------------------------------------------

Processing node 0: MSF Files

------------------------------------------------------------------

1. Storage Settings:

- Spectra to Store: Identified or Quantified

- Feature Traces to Store: All

2. Merging of Identified Peptide and Proteins:

- Merge Mode: Globally by Search Engine Type

3. FASTA Title Line Display:

- Reported FASTA Title Lines: Best match

- Title Line Rule: standard

4. PSM Filters:

- Maximum Delta Cn: 0.05

- Maximum Rank: 0

- Maximum Delta Mass: 0 ppm

Hidden Parameters:

- MSF File(s): D:\OG_skin\Search_file.msf

------------------------------------------------------------------

Processing node 10: Feature Mapper

------------------------------------------------------------------

1. Chromatographic Alignment:

- Perform RT Alignment: True

- Maximum RT Shift [min]: 10

- Mass Tolerance: 6 ppm

- Parameter Tuning: Fine

2. Feature Linking and Mapping:

- RT Tolerance [min]: 0

- Mass Tolerance: 0 ppm

- Min. S/N Threshold: 5

------------------------------------------------------------------

Processing node 11: Precursor Ions Quantifier

------------------------------------------------------------------

1. General Quantification Settings:

- Peptides to Use: Unique + Razor

- Consider Protein Groups for Peptide Uniqueness: True

- Use Shared Quan Results: True

- Reject Quan Results with Missing Channels: False

2. Precursor Quantification:

- Precursor Abundance Based On: Intensity

- Min. # Replicate Features [%]: 0

3. Normalization and Scaling:

- Normalization Mode: None

- Scaling Mode: None

4. Exclude Peptides from Protein Quantification:

- For Normalization: Use All Peptides

- For Protein Roll-Up: Use All Peptides

- For Pairwise Ratios: Exclude Modified

5. Quan Rollup and Hypothesis Testing:

- Protein Abundance Calculation: Summed Abundances

- N for Top N: 3

- Protein Ratio Calculation: Pairwise Ratio Based

- Maximum Allowed Fold Change: 1000

- Imputation Mode: None

- Hypothesis Test: t-test (Background Based)

6. Quan Ratio Distributions:

- 1st Fold Change Threshold: 2

- 2nd Fold Change Threshold: 4

- 3rd Fold Change Threshold: 6

- 4th Fold Change Threshold: 8

- 5th Fold Change Threshold: 10

------------------------------------------------------------------

Processing node 1: PSM Grouper

------------------------------------------------------------------

1. Peptide Group Modifications:

- Site Probability Threshold: 75

------------------------------------------------------------------

Processing node 2: Peptide Validator

------------------------------------------------------------------

1. General Validation Settings:

- Validation Mode: Automatic (Control peptide level error rate if possible)

- Target FDR (Strict) for PSMs: 0.01

- Target FDR (Relaxed) for PSMs: 0.05

- Target FDR (Strict) for Peptides: 0.01

- Target FDR (Relaxed) for Peptides: 0.05

2. Specific Validation Settings:

- Validation Based on: q-Value

- Target/Decoy Selection for PSM Level FDR Calculation Based on Score: Automatic

- Reset Confidences for Nodes without Decoy Search (Fixed score thresholds): False

------------------------------------------------------------------

Processing node 3: Peptide and Protein Filter

------------------------------------------------------------------

1. Peptide Filters:

- Peptide Confidence At Least: High

- Keep Lower Confident PSMs: False

- Minimum Peptide Length: 6

- Remove Peptides Without Protein Reference: False

2. Protein Filters:

- Minimum Number of Peptide Sequences: 1

- Count Only Rank 1 Peptides: False

- Count Peptides Only for Top Scored Protein: False

------------------------------------------------------------------

Processing node 4: Protein Scorer

------------------------------------------------------------------

No parameters

------------------------------------------------------------------

Processing node 7: Protein FDR Validator

------------------------------------------------------------------

1. Confidence Thresholds:

- Target FDR (Strict): 0.01

- Target FDR (Relaxed): 0.05

------------------------------------------------------------------

Processing node 5: Protein Grouping

------------------------------------------------------------------

1. Protein Grouping:

- Apply strict parsimony principle: True

------------------------------------------------------------------

Processing node 6: Peptide in Protein Annotation

------------------------------------------------------------------

1. Flanking Residues:

- Annotate Flanking Residues of the Peptide: True

- Number Flanking Residues in Connection Tables: 1

2. Modifications in Peptide:

- Protein Modifications Reported: For All Proteins

3. Modifications in Protein:

- Modification Sites Reported: All And Specific

- Minimum PSM Confidence: High

- Report Only PTMs: True

4. Positions in Protein:

- Protein Positions for Peptides: For All Proteins

------------------------------------------------------------------

Processing node 15: Modification Sites

------------------------------------------------------------------

1. General:

- Report Only PTMs: False

- Only Master Proteins: False

- Motif Radius: 6

------------------------------------------------------------------

Processing node 9: Protein Marker

------------------------------------------------------------------

1. Contaminant Database:

- Protein Database: contaminants.fasta

5. Annotate Species:

- As Species Map: True

- As Species Names: True

6. Mark Additional Entities:

- Annotation Groups: True

- Pathway Groups: True

- Modification Sites: True

- Peptide Isoform Groups: True

------------------------------------------------------------------

Processing node 16: Peptide Isoform Grouper

------------------------------------------------------------------

No parameters

------------------------------------------------------------------

Processing node 12: Result Statistics

------------------------------------------------------------------

No parameters

------------------------------------------------------------------

Processing node 13: Display Settings

------------------------------------------------------------------

1. General:

- Filter Set:

### Filter Set MasterProteinFilter.filterset contains the following filters:

### Row Filter for TargetProtein:

### Master is equal to Master

###

'magellan filter set' 1 'MasterProteinFilter.filterset' FiltersetProperties 1 'LastFileName' 'C:\Users\frank.berg\Desktop\MasterProteinFilter.filterset' Filter 'TargetProtein' 1 NARY_AND 1 = FilterConditionProperties 1 'NamedComparableFilterCondition/DisplayPropertyHint' 'Master' property 'Thermo.PD.EntityDataFramework.MasterProteinAssessment, Thermo.Magellan.EntityDataFramework' 'IsMasterProtein' constant 'Thermo.PD.EntityDataFramework.MasterProteinAssessment, Thermo.Magellan.EntityDataFramework' 'IsMasterProtein'

------------------------------------------------------------------

Processing node 14: Data Distributions

------------------------------------------------------------------

1. ID Distributions (Bottom-up):

- Peptides to Use: Only unique peptides based on protein groups
