# Supplementary material for: Integrative transcriptomics and peptidomics approach reveals unexpectedly diverse endogenous secretory peptides in Odorrana grahami frog skin
Source: BMC Biol. 2025 Nov 28;23:354. doi: 10.1186/s12915-025-02463-w (PMC12664280; doi:10.1186/s12915-025-02463-w)
Supplement: Supplementary file 3 — Additional file 3. Exported and processed results derived from the native Proteome Discoverer result files (*.pdResult; Data S2 of [44]). SPS/ – Results of searching against the SPS database. All_ESP_sequences_from_this_species/ – Results of searching against all ESP sequences from this species, including previously reported ones; contains suspected false positives (e.g., esculentin-2-OG1/OG2, odorranain-C3). Annotated Spectra.zip, *_export.xlsx, MSMS Scans and PSMs.xlsx – Files exported from *.pdResult, including annotated MS/MS spectra, protein/peptide isoform identifications, and MS/MS scan metadata. Result.xlsx – Final filtered peptide list obtained after processing by the Peptide.py script. [file 12915_2025_2463_MOESM3_ESM.zip › Additional file 3/readme.pdf]

Mass Spectrometry Database Search Supported Materials

The folders contain different mass spectrometry search results:

The **SPS** folder contains the results of searching against SPS.

The **All_ESP_sequences_from_this_species** folder contains the results of searching against all ESP sequences from this specific frog and those previously reported from the same species with no dynamic modifications (protein terminus) due to them being mature peptide sequences.

The files in every folder:

The **Annotated Spectra.zip** contains the annotated MS/MS spectra exported from the native result file *.pdResult (Data S2 of [44]) using Proteome Discoverer v2.5 (PD). In SPS folder , this file includes all unambiguous and high-confidence annotated MS/MS spectra of the 16 final ESPs identified in this study. The file within the All_ESP_sequences_from_this_species folder contains 3 peptides such as esculentin-2- OG1, esculentin-2-OG2, and odorranain-C3. Their “Rana Box” disulfide bonds are in a reduced state. The reduced disulfide ring region (Cys1-SH, Cys2-SH) should have produced many fragment ions in the mass spectrometry, but manual inspection showed an absence of fragment ions from these regions, suggesting a high false positive rate for these sequences.

The file ***_export.xlsx** contains the protein and peptide isoforms identified exported from the native result file *.pdResult (Data S2 of [44]) using PD. When exporting results, the layout setting uses the file **ORF Layout.pdLayout** in the root directory by the “File → Export → To Microsoft Excel” function, with Level 1: Proteins and Level 2: Peptide Isoforms selected.

The file **MSMS Scans and PSMs.xlsx** contains MS/MS scan metadata and PSM identification details exported from the native result file *.pdResult (Data S2 of [44]) using PD.

The file **Result.xlsx** contains the final results. It contains the list of mature peptides (in the “Peptide” column), which were obtained by manually removing any candidate mature peptides that had less than 3 PSMs or lacked a stop codon for all their corresponding proteins, such as master and non-master proteins, after applying Peptide.py processing.

Note: The other parameters used in PD are provided in Parameters S2 of Additional file 2.
