# Supplementary material for: Integrative transcriptomics and peptidomics approach reveals unexpectedly diverse endogenous secretory peptides in Odorrana grahami frog skin
Source: BMC Biol. 2025 Nov 28;23:354. doi: 10.1186/s12915-025-02463-w (PMC12664280; doi:10.1186/s12915-025-02463-w)
Supplement: Supplementary file 4 — Additional file 4. Mass spectrometry-detected mature peptides and truncations mapped to corresponding master proteins (excluding brevinin-2GRa, shown in Additional file 2: Fig. S3a). [file 12915_2025_2463_MOESM4_ESM.zip › Additional file 4/TRINITY_DN0_c1_g1_i11.p1.html]

MView


|  |
| --- |
| ``` Reference sequence (1): TRINITY_DN0_c1_g1_i11.p1 Identities normalised by aligned length. Colored by: property ``` |
| ```                                       cov    pid  1 [        .         .         .         .         :         .         .     ] 76  1 TRINITY_DN0_c1_g1_i11.p1        100.0% 100.0%    MFTLKKSLLLLFFLGTISLSLCEEERDADEDDGVEVTEEEVKRGVLGTVKNLLIGAGKSAAQSVLKTLSCKLSNDC     6 1-1.7e+09|1-35|1-33|1-E^2-E^4-E  43.4% 100.0%    -------------------------------------------GVLGTVKNLLIGAGKSAAQSVLKTLSCKLSNDC     8 9-4.1e+07|13-3|2-29|11-E^27-N    38.2% 100.0%    -----------------------------------------------TVKNLLIGAGKSAAQSVLKTLSCKLSNDC     5 2-4.2e+08|2-9|3-25|3-S^5-S       32.9% 100.0%    ---------------------------------------------------LLIGAGKSAAQSVLKTLSCKLSNDC    17 7-4.3e+07|12-3|4-23|10-E         30.3% 100.0%    -------------------------------------------GVLGTVKNLLIGAGKSAAQSVLK----------     7 14-9.5e+06|10-4|5-23|21-S^26-N   30.3% 100.0%    -----------------------------------------------------IGAGKSAAQSVLKTLSCKLSNDC     4 4-8.3e+07|3-8|6-21|8-S^19-S      27.6% 100.0%    -------------------------------------------------------AGKSAAQSVLKTLSCKLSNDC    10 10-4.1e+07|8-6|7-20|13-S^18-S    26.3% 100.0%    --------------------------------------------------------GKSAAQSVLKTLSCKLSNDC    14 16-2.7e+06|16-2|8-20|23-E        26.3% 100.0%    -------------------------------------------GVLGTVKNLLIGAGKSAAQS-------------    13 13-1.4e+07|15-2|10-19|17-E       25.0% 100.0%    -------------------------------------------GVLGTVKNLLIGAGKSAAQ--------------     3 8-4.2e+07|6-7|9-19|12-S^20-S     25.0% 100.0%    ---------------------------------------------------------KSAAQSVLKTLSCKLSNDC    15 12-1.4e+07|5-8|12-18|16-E        23.7% 100.0%    -------------------------------------------GVLGTVKNLLIGAGKSAA---------------     9 6-7.8e+07|7-6|11-18|9-S^14-S     23.7% 100.0%    ----------------------------------------------------------SAAQSVLKTLSCKLSNDC    12 11-1.5e+07|9-6|13-17|15-E        22.4% 100.0%    -------------------------------------------GVLGTVKNLLIGAGKSA----------------     2 17-1.1e+06|17-1|14-17|24-S       22.4% 100.0%    -----------------------------------------------------------AAQSVLKTLSCKLSNDC    18 15-7.5e+06|11-4|15-16|22-E       21.1% 100.0%    -------------------------------------------GVLGTVKNLLIGAGKS-----------------    19 18-8.8e+05|18-1|16-16|25-S       21.1% 100.0%    ------------------------------------------------------------AQSVLKTLSCKLSNDC    16 5-8.2e+07|4-8|17-15|7-E          19.7% 100.0%    -------------------------------------------GVLGTVKNLLIGAGK------------------    11 3-1.0e+08|14-2|18-13|6-S         17.1% 100.0%    -------------------------------------------GVLGTVKNLLIGA-------------------- ``` |

MView 1.67, Copyright © 1997-2020 Nigel P. Brown
