# Supplementary material for: Integrative transcriptomics and peptidomics approach reveals unexpectedly diverse endogenous secretory peptides in Odorrana grahami frog skin
Source: BMC Biol. 2025 Nov 28;23:354. doi: 10.1186/s12915-025-02463-w (PMC12664280; doi:10.1186/s12915-025-02463-w)
Supplement: Supplementary file 4 — Additional file 4. Mass spectrometry-detected mature peptides and truncations mapped to corresponding master proteins (excluding brevinin-2GRa, shown in Additional file 2: Fig. S3a). [file 12915_2025_2463_MOESM4_ESM.zip › Additional file 4/TRINITY_DN0_c1_g1_i14.p1,TRINITY_DN0_c1_g1_i4.p1.html]

MView


|  |
| --- |
| ``` Reference sequence (1): TRINITY_DN0_c1_g1_i14.p1,TRINITY_DN0_c1_g1_i4.p1 Identities normalised by aligned length. Colored by: property ``` |
| ```                                                             cov    pid  1 [        .         .         .         .         :         .         .     ] 76  1 TRINITY_DN0_c1_g1_i14.p1,TRINITY_DN0_c1_g1_i4.p1      100.0% 100.0%    MFTLKKSLLLLFFLGTISLSLCEEERDADEDDGVEVTEEEVKRGLLDTFKNLALNAAKSAGVSVLNSLSCKLSKTC     5 1-1.0e+10|1-95|1-33|1-E^2-E^3-E^4-E^6-E^7-E^10-E^38-E  43.4% 100.0%    -------------------------------------------GLLDTFKNLALNAAKSAGVSVLNSLSCKLSKTC    26 25-2.1e+07|23-2|2-32|40-E                              42.1% 100.0%    --------------------------------------------LLDTFKNLALNAAKSAGVSVLNSLSCKLSKTC    27 28-0.0e+00|28-1|3-31|64-N                              40.8% 100.0%    ---------------------------------------------LDTFKNLALNAAKSAGVSVLNSLSCKLSKTC    29 23-3.2e+07|21-2|4-30|32-E                              39.5% 100.0%    ----------------------------------------------DTFKNLALNAAKSAGVSVLNSLSCKLSKTC    12 2-1.2e+09|2-25|5-29|5-E^9-E^15-E^16-E^44-E^48-E        38.2% 100.0%    -----------------------------------------------TFKNLALNAAKSAGVSVLNSLSCKLSKTC    28 27-5.5e+06|24-2|6-28|58-E                              36.8% 100.0%    ------------------------------------------------FKNLALNAAKSAGVSVLNSLSCKLSKTC    22 17-4.8e+07|13-6|7-27|26-E                              35.5% 100.0%    -------------------------------------------------KNLALNAAKSAGVSVLNSLSCKLSKTC    23 15-5.6e+07|18-4|8-26|27-E^51-E                         34.2% 100.0%    --------------------------------------------------NLALNAAKSAGVSVLNSLSCKLSKTC    14 7-1.3e+08|15-4|9-25|13-E                               32.9% 100.0%    ---------------------------------------------------LALNAAKSAGVSVLNSLSCKLSKTC    18 14-6.4e+07|5-10|10-24|28-E^50-E^53-E                   31.6% 100.0%    ----------------------------------------------------ALNAAKSAGVSVLNSLSCKLSKTC    21 18-4.2e+07|6-10|11-23|33-E^60-E^61-E                   30.3% 100.0%    -----------------------------------------------------LNAAKSAGVSVLNSLSCKLSKTC    24 19-3.7e+07|9-7|12-22|36-E^56-E^62-E                    28.9% 100.0%    ------------------------------------------------------NAAKSAGVSVLNSLSCKLSKTC    19 13-7.4e+07|8-9|13-21|24-E^45-E                         27.6% 100.0%    -------------------------------------------------------AAKSAGVSVLNSLSCKLSKTC    17 26-1.5e+07|10-7|14-20|49-E^63-E                        26.3% 100.0%    --------------------------------------------------------AKSAGVSVLNSLSCKLSKTC    20 22-3.3e+07|7-10|15-19|35-E^57-E                        25.0% 100.0%    ---------------------------------------------------------KSAGVSVLNSLSCKLSKTC    15 5-1.9e+08|11-6|17-18|11-E^37-E                         23.7% 100.0%    ----------------------------------------------------------SAGVSVLNSLSCKLSKTC    30 29-0.0e+00|29-1|18-18|65-N                             23.7% 100.0%    -----------------------------------------------TFKNLALNAAKSAGVSVL-----------     8 4-2.5e+08|3-13|16-18|12-E^29-E^30-E^55-E               23.7% 100.0%    -------------------------------------------GLLDTFKNLALNAAKSAG---------------    16 21-3.7e+07|20-3|20-17|34-E^54-E                        22.4% 100.0%    -----------------------------------------------------------AGVSVLNSLSCKLSKTC     7 6-1.5e+08|12-6|19-17|14-E^46-E^47-E                    22.4% 100.0%    -------------------------------------------GLLDTFKNLALNAAKSA----------------    25 12-7.6e+07|14-5|22-16|23-E^43-E                        21.1% 100.0%    ------------------------------------------------------------GVSVLNSLSCKLSKTC     9 11-8.5e+07|17-4|21-16|22-E^42-E                        21.1% 100.0%    -------------------------------------------GLLDTFKNLALNAAKS-----------------    13 9-9.8e+07|16-4|24-15|18-E^52-E                         19.7% 100.0%    -------------------------------------------------------------VSVLNSLSCKLSKTC     4 3-4.2e+08|4-10|23-15|8-E^20-E^21-E^59-E                19.7% 100.0%    -------------------------------------------GLLDTFKNLALNAAK------------------    11 20-3.7e+07|27-1|25-14|31-E                             18.4% 100.0%    -------------------------------------------GLLDTFKNLALNAA-------------------     6 16-5.0e+07|26-1|26-13|25-E                             17.1% 100.0%    -------------------------------------------GLLDTFKNLALNA--------------------    10 10-9.5e+07|25-1|27-12|17-E                             15.8% 100.0%    -------------------------------------------GLLDTFKNLALN---------------------     3 8-1.0e+08|19-3|28-11|19-E^41-E                         14.5% 100.0%    -------------------------------------------GLLDTFKNLAL----------------------     2 24-2.2e+07|22-2|29-9|39-E                              11.8% 100.0%    -------------------------------------------GLLDTFKNL------------------------ ``` |

MView 1.67, Copyright © 1997-2020 Nigel P. Brown
