# Supplementary material for: Integrative transcriptomics and peptidomics approach reveals unexpectedly diverse endogenous secretory peptides in Odorrana grahami frog skin
Source: BMC Biol. 2025 Nov 28;23:354. doi: 10.1186/s12915-025-02463-w (PMC12664280; doi:10.1186/s12915-025-02463-w)
Supplement: Supplementary file 4 — Additional file 4. Mass spectrometry-detected mature peptides and truncations mapped to corresponding master proteins (excluding brevinin-2GRa, shown in Additional file 2: Fig. S3a). [file 12915_2025_2463_MOESM4_ESM.zip › Additional file 4/TRINITY_DN0_c1_g1_i16.p1.html]

MView


|  |
| --- |
| ``` Reference sequence (1): TRINITY_DN0_c1_g1_i16.p1 Identities normalised by aligned length. Colored by: property ``` |
| ```                                  cov    pid  1 [        .         .         .         .         :         .         .         .   ] 84  1 TRINITY_DN0_c1_g1_i16.p1   100.0% 100.0%    MFTLKKSMLLLFFLGTISLSLCEQERAADEDEGNEIKRGLFSKFAGKGIKDLIFKGVKHIGKEVGMDVIRTGIDVAGCKIKGEC     6 1-2.1e+07|7-1|1-46|1-E      54.8% 100.0%    --------------------------------------GLFSKFAGKGIKDLIFKGVKHIGKEVGMDVIRTGIDVAGCKIKGEC     7 4-5.8e+06|8-1|2-36|4-E      42.9% 100.0%    --------------------------------------GLFSKFAGKGIKDLIFKGVKHIGKEVGMDVIRTGID----------    10 3-1.1e+07|1-5|3-33|3-E^9-E  39.3% 100.0%    ---------------------------------------------------LIFKGVKHIGKEVGMDVIRTGIDVAGCKIKGEC     5 2-1.2e+07|2-3|4-29|2-E      34.5% 100.0%    --------------------------------------GLFSKFAGKGIKDLIFKGVKHIGKEVGMD-----------------     4 6-3.1e+06|4-2|5-23|6-E      27.4% 100.0%    --------------------------------------GLFSKFAGKGIKDLIFKGVKHIG-----------------------     9 7-2.2e+06|5-2|6-20|7-E      23.8% 100.0%    --------------------------------------GLFSKFAGKGIKDLIFKGVK--------------------------    11 5-3.8e+06|3-2|7-19|5-E      22.6% 100.0%    -----------------------------------------------------------------MDVIRTGIDVAGCKIKGEC     8 8-1.3e+06|6-2|8-18|8-E      21.4% 100.0%    --------------------------------------GLFSKFAGKGIKDLIFKG----------------------------     3 9-8.0e+05|9-1|9-17|10-E     20.2% 100.0%    --------------------------------------GLFSKFAGKGIKDLIFK-----------------------------     2 10-7.7e+05|10-1|10-9|11-S   10.7% 100.0%    --------------------------------------GLFSKFAGK------------------------------------- ``` |

MView 1.67, Copyright © 1997-2020 Nigel P. Brown
