# Supplementary material for: Integrative transcriptomics and peptidomics approach reveals unexpectedly diverse endogenous secretory peptides in Odorrana grahami frog skin
Source: BMC Biol. 2025 Nov 28;23:354. doi: 10.1186/s12915-025-02463-w (PMC12664280; doi:10.1186/s12915-025-02463-w)
Supplement: Supplementary file 4 — Additional file 4. Mass spectrometry-detected mature peptides and truncations mapped to corresponding master proteins (excluding brevinin-2GRa, shown in Additional file 2: Fig. S3a). [file 12915_2025_2463_MOESM4_ESM.zip › Additional file 4/TRINITY_DN0_c1_g1_i22.p1.html]

MView


|  |
| --- |
| ``` Reference sequence (1): TRINITY_DN0_c1_g1_i22.p1 Identities normalised by aligned length. Colored by: property ``` |
| ```                                             cov    pid  1 [        .         .         .         .         :         .         .       ] 78  1 TRINITY_DN0_c1_g1_i22.p1              100.0% 100.0%    MFTLNKSLLLLFFLGTISLSLCQEERAADEEDNGEVEEVKRGLFTLIKGAAKLIGKTVAKEAGKTGLELMACKITNQC     3 1-6.5e+08|1-19|1-37|6-E^7-E^12-E^13-E  47.4% 100.0%    -----------------------------------------GLFTLIKGAAKLIGKTVAKEAGKTGLELMACKITNQC    24 21-3.6e+06|20-2|2-30|26-E^28-E         38.5% 100.0%    ------------------------------------------------GAAKLIGKTVAKEAGKTGLELMACKITNQC    13 16-1.0e+07|17-2|3-29|19-E              37.2% 100.0%    -----------------------------------------GLFTLIKGAAKLIGKTVAKEAGKTGLELM--------    26 24-1.2e+06|25-1|4-29|29-E              37.2% 100.0%    -------------------------------------------------AAKLIGKTVAKEAGKTGLELMACKITNQC    12 14-1.6e+07|15-2|5-28|17-E              35.9% 100.0%    -----------------------------------------GLFTLIKGAAKLIGKTVAKEAGKTGLEL---------     7 19-4.7e+06|22-1|6-27|21-E              34.6% 100.0%    -----------------------------------------GLFTLIKGAAKLIGKTVAKEAGKTGLE----------    21 9-6.3e+07|10-4|7-26|10-E               33.3% 100.0%    -----------------------------------------GLFTLIKGAAKLIGKTVAKEAGKTGL-----------    14 7-1.8e+08|9-4|8-25|8-E                 32.1% 100.0%    -----------------------------------------GLFTLIKGAAKLIGKTVAKEAGKTG------------    19 10-6.3e+07|11-4|9-24|11-E              30.8% 100.0%    -----------------------------------------GLFTLIKGAAKLIGKTVAKEAGKT-------------    25 25-1.1e+06|26-1|11-23|30-E             29.5% 100.0%    -------------------------------------------------------KTVAKEAGKTGLELMACKITNQC     4 3-5.0e+08|7-7|10-23|2-E                29.5% 100.0%    -----------------------------------------GLFTLIKGAAKLIGKTVAKEAGK--------------    23 27-6.6e+05|27-1|14-22|32-E             28.2% 100.0%    ------------------------------------------LFTLIKGAAKLIGKTVAKEAGK--------------    27 22-2.7e+06|23-1|13-22|25-E             28.2% 100.0%    --------------------------------------------------------TVAKEAGKTGLELMACKITNQC     5 4-4.5e+08|2-18|12-22|3-E               28.2% 100.0%    -----------------------------------------GLFTLIKGAAKLIGKTVAKEAG---------------    10 5-3.3e+08|4-12|15-21|4-E               26.9% 100.0%    -----------------------------------------GLFTLIKGAAKLIGKTVAKEA----------------    22 26-9.6e+05|21-2|16-21|31-E             26.9% 100.0%    ------------------------------------------LFTLIKGAAKLIGKTVAKEAG---------------    20 8-1.3e+08|6-12|17-20|9-E               25.6% 100.0%    -----------------------------------------GLFTLIKGAAKLIGKTVAKE-----------------     9 11-5.4e+07|3-16|18-19|14-E             24.4% 100.0%    -----------------------------------------GLFTLIKGAAKLIGKTVAK------------------    18 15-1.2e+07|16-2|19-18|18-E             23.1% 100.0%    -----------------------------------------GLFTLIKGAAKLIGKTVA-------------------    15 20-4.5e+06|19-2|20-17|22-E             21.8% 100.0%    -----------------------------------------GLFTLIKGAAKLIGKTV--------------------    17 13-1.9e+07|12-4|21-16|16-E             20.5% 100.0%    -----------------------------------------GLFTLIKGAAKLIGKT---------------------    28 23-2.0e+06|24-1|22-16|27-E             20.5% 100.0%    --------------------------------------------------------------GKTGLELMACKITNQC     2 17-7.6e+06|18-2|24-15|23-E^24-E        19.2% 100.0%    ---------------------------------------------------------------KTGLELMACKITNQC     8 6-3.2e+08|5-12|23-15|5-E               19.2% 100.0%    -----------------------------------------GLFTLIKGAAKLIGK----------------------    16 2-5.1e+08|8-6|25-14|1-E                17.9% 100.0%    -----------------------------------------GLFTLIKGAAKLIG-----------------------    11 12-2.3e+07|14-2|26-12|15-E             15.4% 100.0%    -----------------------------------------GLFTLIKGAAKL-------------------------     6 18-6.6e+06|13-3|27-11|20-E             14.1% 100.0%    -----------------------------------------GLFTLIKGAAK-------------------------- ``` |

MView 1.67, Copyright © 1997-2020 Nigel P. Brown
