# Supplementary material for: Integrative transcriptomics and peptidomics approach reveals unexpectedly diverse endogenous secretory peptides in Odorrana grahami frog skin
Source: BMC Biol. 2025 Nov 28;23:354. doi: 10.1186/s12915-025-02463-w (PMC12664280; doi:10.1186/s12915-025-02463-w)
Supplement: Supplementary file 4 — Additional file 4. Mass spectrometry-detected mature peptides and truncations mapped to corresponding master proteins (excluding brevinin-2GRa, shown in Additional file 2: Fig. S3a). [file 12915_2025_2463_MOESM4_ESM.zip › Additional file 4/TRINITY_DN0_c1_g1_i24.p1.html]

MView


|  |
| --- |
| ``` Reference sequence (1): TRINITY_DN0_c1_g1_i24.p1 Identities normalised by aligned length. Colored by: property ``` |
| ```                                  cov    pid  1 [        .         .         .         .         :         .         .] 71 1 TRINITY_DN0_c1_g1_i24.p1    100.0% 100.0%    MFTLKKSMLLLFFLGTISLSLCEQERDADEEERRDDPEERDVEVEKRFLPLLAGLAANFLPKLFCKITKKC    4 1-2.1e+09|1-55|1-24|1-E^2-E  33.8% 100.0%    -----------------------------------------------FLPLLAGLAANFLPKLFCKITKKC    7 6-8.3e+06|6-2|2-23|8-E       32.4% 100.0%    ------------------------------------------------LPLLAGLAANFLPKLFCKITKKC    8 7-5.3e+06|7-1|3-22|9-E       31.0% 100.0%    -------------------------------------------------PLLAGLAANFLPKLFCKITKKC    3 2-1.9e+08|2-6|4-21|3-E^7-E   29.6% 100.0%    --------------------------------------------------LLAGLAANFLPKLFCKITKKC    5 5-1.9e+07|5-4|5-19|6-E       26.8% 100.0%    ----------------------------------------------------AGLAANFLPKLFCKITKKC    6 4-3.0e+07|4-4|6-18|5-E       25.4% 100.0%    -----------------------------------------------------GLAANFLPKLFCKITKKC    2 3-5.7e+07|3-5|7-17|4-E^11-E  23.9% 100.0%    ------------------------------------------------------LAANFLPKLFCKITKKC    9 8-3.4e+06|8-1|8-16|10-E      22.5% 100.0%    -------------------------------------------------------AANFLPKLFCKITKKC ``` |

MView 1.67, Copyright © 1997-2020 Nigel P. Brown
