# Supplementary material for: Integrative transcriptomics and peptidomics approach reveals unexpectedly diverse endogenous secretory peptides in Odorrana grahami frog skin
Source: BMC Biol. 2025 Nov 28;23:354. doi: 10.1186/s12915-025-02463-w (PMC12664280; doi:10.1186/s12915-025-02463-w)
Supplement: Supplementary file 4 — Additional file 4. Mass spectrometry-detected mature peptides and truncations mapped to corresponding master proteins (excluding brevinin-2GRa, shown in Additional file 2: Fig. S3a). [file 12915_2025_2463_MOESM4_ESM.zip › Additional file 4/TRINITY_DN10285_c0_g1_i1.p1.html]

MView


|  |
| --- |
| ``` Reference sequence (1): TRINITY_DN10285_c0_g1_i1.p1 Identities normalised by aligned length. Colored by: property ``` |
| ```                                                  cov    pid  1 [        .         .         .         .         :         .         .] 71 1 TRINITY_DN10285_c0_g1_i1.p1                 100.0% 100.0%    MFTMKKSLLVLFFLGIVSLSLCQEERSADDEEGEDIEEEVKRGFMDTAKNVAKNVAVTLLDNLKCKITKAC    4 1-5.1e+08|1-22|1-29|1-E^2-E^3-E^5-E^6-E^7-E  40.8% 100.0%    ------------------------------------------GFMDTAKNVAKNVAVTLLDNLKCKITKAC    3 2-7.4e+07|2-8|2-25|4-E^9-E^10-E              35.2% 100.0%    ----------------------------------------------TAKNVAKNVAVTLLDNLKCKITKAC    5 4-2.4e+06|4-1|3-19|11-E                      26.8% 100.0%    ----------------------------------------------------KNVAVTLLDNLKCKITKAC    2 3-7.0e+06|3-2|4-17|8-E                       23.9% 100.0%    ------------------------------------------------------VAVTLLDNLKCKITKAC ``` |

MView 1.67, Copyright © 1997-2020 Nigel P. Brown
