# Supplementary material for: Integrative transcriptomics and peptidomics approach reveals unexpectedly diverse endogenous secretory peptides in Odorrana grahami frog skin
Source: BMC Biol. 2025 Nov 28;23:354. doi: 10.1186/s12915-025-02463-w (PMC12664280; doi:10.1186/s12915-025-02463-w)
Supplement: Supplementary file 4 — Additional file 4. Mass spectrometry-detected mature peptides and truncations mapped to corresponding master proteins (excluding brevinin-2GRa, shown in Additional file 2: Fig. S3a). [file 12915_2025_2463_MOESM4_ESM.zip › Additional file 4/TRINITY_DN2658_c0_g2_i1.p1.html]

MView


|  |
| --- |
| ``` Reference sequence (1): TRINITY_DN2658_c0_g2_i1.p1 Identities normalised by aligned length. Colored by: property ``` |
| ```                                           cov    pid  1 [        .         .         .         .         :         .         .     ] 76  1 TRINITY_DN2658_c0_g2_i1.p1          100.0% 100.0%    KMFTMKKPLLLPFFLRTISLSLCEEERDADEEDGEMTEEEVKRGVLGTVKNLLIGASKSAAQSVLKTLSCKLSNDC     4 1-2.3e+09|1-21|1-33|1-E^2-E^3-E^6-E  43.4% 100.0%    -------------------------------------------GVLGTVKNLLIGASKSAAQSVLKTLSCKLSNDC     6 5-2.9e+07|6-1|2-29|8-E               38.2% 100.0%    -----------------------------------------------TVKNLLIGASKSAAQSVLKTLSCKLSNDC     8 8-4.5e+06|8-1|3-25|13-E              32.9% 100.0%    ---------------------------------------------------LLIGASKSAAQSVLKTLSCKLSNDC    10 6-1.7e+07|7-1|4-23|10-E              30.3% 100.0%    -------------------------------------------GVLGTVKNLLIGASKSAAQSVLK----------     7 9-2.9e+06|9-1|5-21|15-E              27.6% 100.0%    -------------------------------------------------------ASKSAAQSVLKTLSCKLSNDC    11 7-1.6e+07|5-2|7-19|11-E^14-E         25.0% 100.0%    -------------------------------------------GVLGTVKNLLIGASKSAAQ--------------     3 4-4.2e+07|2-7|6-19|7-S^12-S          25.0% 100.0%    ---------------------------------------------------------KSAAQSVLKTLSCKLSNDC     9 3-7.8e+07|3-6|8-18|5-S^9-S           23.7% 100.0%    ----------------------------------------------------------SAAQSVLKTLSCKLSNDC     2 10-1.1e+06|10-1|9-17|16-S            22.4% 100.0%    -----------------------------------------------------------AAQSVLKTLSCKLSNDC    12 11-8.8e+05|11-1|10-16|17-S           21.1% 100.0%    ------------------------------------------------------------AQSVLKTLSCKLSNDC     5 2-1.0e+08|4-2|11-13|4-S              17.1% 100.0%    -------------------------------------------GVLGTVKNLLIGA-------------------- ``` |

MView 1.67, Copyright © 1997-2020 Nigel P. Brown
