# Supplementary material for: Integrative transcriptomics and peptidomics approach reveals unexpectedly diverse endogenous secretory peptides in Odorrana grahami frog skin
Source: BMC Biol. 2025 Nov 28;23:354. doi: 10.1186/s12915-025-02463-w (PMC12664280; doi:10.1186/s12915-025-02463-w)
Supplement: Supplementary file 4 — Additional file 4. Mass spectrometry-detected mature peptides and truncations mapped to corresponding master proteins (excluding brevinin-2GRa, shown in Additional file 2: Fig. S3a). [file 12915_2025_2463_MOESM4_ESM.zip › Additional file 4/TRINITY_DN440_c40_g1_i1.p1.html]

MView


|  |
| --- |
| ``` Reference sequence (1): TRINITY_DN440_c40_g1_i1.p1 Identities normalised by aligned length. Colored by: property ``` |
| ```                                         cov    pid  1 [        .         .         .         .         ] 50  1 TRINITY_DN440_c40_g1_i1.p1        100.0% 100.0%    MNRKSSDVEVTEEEVKRGLLDTFKNMALNAAKSAGVSVLNALSCKLSKTC     4 1-4.3e+08|1-6|1-33|1-E^20-N^21-N   66.0% 100.0%    -----------------GLLDTFKNMALNAAKSAGVSVLNALSCKLSKTC    12 13-1.3e+06|13-1|2-27|17-E          54.0% 100.0%    -----------------------KNMALNAAKSAGVSVLNALSCKLSKTC     9 6-5.3e+06|9-2|3-24|6-E             48.0% 100.0%    --------------------------ALNAAKSAGVSVLNALSCKLSKTC    13 12-1.6e+06|12-1|4-23|16-E          46.0% 100.0%    ---------------------------LNAAKSAGVSVLNALSCKLSKTC    10 8-4.8e+06|5-4|5-22|13-E^14-E       44.0% 100.0%    ----------------------------NAAKSAGVSVLNALSCKLSKTC     7 4-1.1e+07|4-4|6-21|3-E^22-N        42.0% 100.0%    -----------------------------AAKSAGVSVLNALSCKLSKTC    11 11-2.9e+06|7-3|7-19|10-E^23-N      38.0% 100.0%    -------------------------------KSAGVSVLNALSCKLSKTC    15 9-4.3e+06|10-1|8-18|8-E            36.0% 100.0%    --------------------------------SAGVSVLNALSCKLSKTC     6 10-4.0e+06|11-1|9-18|9-E           36.0% 100.0%    -----------------GLLDTFKNMALNAAKSAG---------------     8 7-5.2e+06|6-3|10-17|7-E            34.0% 100.0%    ---------------------------------AGVSVLNALSCKLSKTC    14 5-9.3e+06|8-2|11-16|4-E            32.0% 100.0%    ----------------------------------GVSVLNALSCKLSKTC     3 3-1.3e+07|3-4|12-15|5-E^11-E^12-E  30.0% 100.0%    -----------------GLLDTFKNMALNAAK------------------     5 2-1.9e+07|2-4|13-12|2-E^15-E^19-E  24.0% 100.0%    -----------------GLLDTFKNMALN---------------------     2 14-1.1e+06|14-1|14-11|18-E         22.0% 100.0%    -----------------GLLDTFKNMAL---------------------- ``` |

MView 1.67, Copyright © 1997-2020 Nigel P. Brown
