# Supplementary material for: Integrative transcriptomics and peptidomics approach reveals unexpectedly diverse endogenous secretory peptides in Odorrana grahami frog skin
Source: BMC Biol. 2025 Nov 28;23:354. doi: 10.1186/s12915-025-02463-w (PMC12664280; doi:10.1186/s12915-025-02463-w)
Supplement: Supplementary file 4 — Additional file 4. Mass spectrometry-detected mature peptides and truncations mapped to corresponding master proteins (excluding brevinin-2GRa, shown in Additional file 2: Fig. S3a). [file 12915_2025_2463_MOESM4_ESM.zip › Additional file 4/TRINITY_DN49_c0_g1_i1.p1_ORF1.html]

MView


|  |
| --- |
| ``` Reference sequence (1): TRINITY_DN49_c0_g1_i1.p1_ORF1 Identities normalised by aligned length. Colored by: property ``` |
| ```                                      cov    pid  1 [        .         .         .         .         :         .     ] 66 1 TRINITY_DN49_c0_g1_i1.p1_ORF1   100.0% 100.0%    MFTLKKSLLLLFFLGTINLSLCQDETNAEEERRDEEVAKMEEIKRGLFGKILGVGKKVLCGLSGMC    3 1-4.4e+09|1-69|1-21|1-E^2-E^3-E  31.8% 100.0%    ---------------------------------------------GLFGKILGVGKKVLCGLSGMC    7 2-2.3e+08|2-9|2-20|4-E^5-E       30.3% 100.0%    ----------------------------------------------LFGKILGVGKKVLCGLSGMC    5 6-8.1e+06|7-1|3-18|9-E           27.3% 100.0%    ------------------------------------------------GKILGVGKKVLCGLSGMC    8 3-1.6e+07|5-2|4-17|6-E           25.8% 100.0%    -------------------------------------------------KILGVGKKVLCGLSGMC    6 5-8.4e+06|3-6|5-14|8-E           21.2% 100.0%    ---------------------------------------------GLFGKILGVGKKVL-------    2 7-2.3e+06|4-6|6-11|10-E          16.7% 100.0%    ---------------------------------------------GLFGKILGVGK----------    4 4-1.2e+07|6-1|7-10|7-E           15.2% 100.0%    ---------------------------------------------GLFGKILGVG----------- ``` |

MView 1.67, Copyright © 1997-2020 Nigel P. Brown
