# Supplementary material for: Integrative transcriptomics and peptidomics approach reveals unexpectedly diverse endogenous secretory peptides in Odorrana grahami frog skin
Source: BMC Biol. 2025 Nov 28;23:354. doi: 10.1186/s12915-025-02463-w (PMC12664280; doi:10.1186/s12915-025-02463-w)
Supplement: Supplementary file 4 — Additional file 4. Mass spectrometry-detected mature peptides and truncations mapped to corresponding master proteins (excluding brevinin-2GRa, shown in Additional file 2: Fig. S3a). [file 12915_2025_2463_MOESM4_ESM.zip › Additional file 4/TRINITY_DN6_c2_g1_i1.p1.html]

MView


|  |
| --- |
| ``` Reference sequence (1): TRINITY_DN6_c2_g1_i1.p1 Identities normalised by aligned length. Colored by: property ``` |
| ```                                  cov    pid  1 [        .         .         .         .        ] 49 1 TRINITY_DN6_c2_g1_i1.p1     100.0% 100.0%    QEERSADEDEGEVIEQEVKRGLMNTVLNVLTNVAGTVKDKIKCKFTGGC    3 1-6.7e+07|1-22|1-29|1-E^2-E  59.2% 100.0%    --------------------GLMNTVLNVLTNVAGTVKDKIKCKFTGGC    2 2-6.2e+05|2-1|2-21|3-E       42.9% 100.0%    ----------------------------VLTNVAGTVKDKIKCKFTGGC ``` |

MView 1.67, Copyright © 1997-2020 Nigel P. Brown
