# Supplementary material for: Integrative transcriptomics and peptidomics approach reveals unexpectedly diverse endogenous secretory peptides in Odorrana grahami frog skin
Source: BMC Biol. 2025 Nov 28;23:354. doi: 10.1186/s12915-025-02463-w (PMC12664280; doi:10.1186/s12915-025-02463-w)
Supplement: Supplementary file 4 — Additional file 4. Mass spectrometry-detected mature peptides and truncations mapped to corresponding master proteins (excluding brevinin-2GRa, shown in Additional file 2: Fig. S3a). [file 12915_2025_2463_MOESM4_ESM.zip › Additional file 4/TRINITY_DN77_c0_g1_i1.p1_ORF1.html]

MView


|  |
| --- |
| ``` Reference sequence (1): TRINITY_DN77_c0_g1_i1.p1_ORF1 Identities normalised by aligned length. Colored by: property ``` |
| ```                                    cov    pid  1 [        .         .         .         .         :         .      ] 67 1 TRINITY_DN77_c0_g1_i1.p1_ORF1 100.0% 100.0%    MFTLKKSMLLLCFLGTISLSLCEQERNAEEEERREEEVAKMEEITRGLLSGILGAGKHIVCGLSGLC    3 1-1.6e+09|1-11|1-21|1-E        31.3% 100.0%    ----------------------------------------------GLLSGILGAGKHIVCGLSGLC    4 2-5.9e+07|2-2|2-18|2-E         26.9% 100.0%    -------------------------------------------------SGILGAGKHIVCGLSGLC    5 3-5.3e+07|4-1|3-16|3-E         23.9% 100.0%    ---------------------------------------------------ILGAGKHIVCGLSGLC    2 4-6.3e+06|3-2|4-11|4-S         16.4% 100.0%    ----------------------------------------------GLLSGILGAGK---------- ``` |

MView 1.67, Copyright © 1997-2020 Nigel P. Brown
