# Supplementary material for: Integrative transcriptomics and peptidomics approach reveals unexpectedly diverse endogenous secretory peptides in Odorrana grahami frog skin
Source: BMC Biol. 2025 Nov 28;23:354. doi: 10.1186/s12915-025-02463-w (PMC12664280; doi:10.1186/s12915-025-02463-w)
Supplement: Supplementary file 4 — Additional file 4. Mass spectrometry-detected mature peptides and truncations mapped to corresponding master proteins (excluding brevinin-2GRa, shown in Additional file 2: Fig. S3a). [file 12915_2025_2463_MOESM4_ESM.zip › Additional file 4/TRINITY_DN96_c0_g1_i2.p1.html]

MView


|  |
| --- |
| ``` Reference sequence (1): TRINITY_DN96_c0_g1_i2.p1 Identities normalised by aligned length. Colored by: property ``` |
| ```                               cov    pid  1 [        .         .         .         .         :         .         .         ] 80 1 TRINITY_DN96_c0_g1_i2.p1 100.0% 100.0%    MFTLKKSLLLFFFLGTISLSLCEQERSADEDDGGEMKGEEAKRGIFAILKIATKLIGKTLAKAAGKAGTGLLACKAAKEC    2 1-2.1e+08|1-5|1-37|1-E    46.2% 100.0%    -------------------------------------------GIFAILKIATKLIGKTLAKAAGKAGTGLLACKAAKEC    3 2-7.5e+06|2-1|2-25|2-E    31.2% 100.0%    -------------------------------------------GIFAILKIATKLIGKTLAKAAGKAG------------ ``` |

MView 1.67, Copyright © 1997-2020 Nigel P. Brown
