# Supplementary material for: Integrative transcriptomics and peptidomics approach reveals unexpectedly diverse endogenous secretory peptides in Odorrana grahami frog skin
Source: BMC Biol. 2025 Nov 28;23:354. doi: 10.1186/s12915-025-02463-w (PMC12664280; doi:10.1186/s12915-025-02463-w)
Supplement: Supplementary file 5 — Additional file 5. Alignments of ESP sequences identified in this study across different regions. [file 12915_2025_2463_MOESM5_ESM.zip › Additional file 5/All 14 families - signal peptide plus up to 45 nucleotides upstream of the 5’-UTR.html]

MView


|  |
| --- |
| ``` Reference sequence (1): F1S1-P1-TRINITY_DN175_c1_g1_i1-9.3e+02-andersonin-Q Identities normalised by aligned length. Colored by: consensus group/60% ``` |
| ```                                                                                           cov    pid  1 [        .         .         .         .         :         .         .         .         .         1         .         .         .         .         :         .         .         .         .         2         .         .         .         .         :] 251   1 F1S1-P1-TRINITY_DN175_c1_g1_i1-9.3e+02-andersonin-Q                                100.0% 100.0%    -----------------------------------caccaactg-------aactaccc------------------gaacccaaagatgttcaccttgaa------------------gaaatccctgttactcct-------------------------------------------tttcttccttgcgaccatcaatttatctctctgt-------------------------------------      11 F1S9-P30-TRINITY_DN11504_c0_g1_i1-4.8e+00-nigrocin-OG35                             87.1%  95.1%    ------------------------------------------------------taccc------------------gagcccaaagatgttcaccttgaa------------------gaaatccctgttactcct-------------------------------------------tttcttccttggtaccatcaacttatctctctgt-------------------------------------       3 F1S5-P5-TRINITY_DN23413_c1_g1_i1-1.3e+00-gaegurin-6-OG1                             74.2%  94.2%    ------------------------------------------------------------------------------------aagatgttcaccttgaa------------------gaaatccctgttactcct-------------------------------------------ttttttccctgggaccatcaacttatctctctgt-------------------------------------       7 F1S5-P8-TRINITY_DN33233_c1_g1_i1-1.1e+02-brevinin-1E-OG10                           80.6%  93.3%    ------------------------------------------------------------------------------agcccaaagatgttcaccatgaa------------------gaaatccctgttactcct-------------------------------------------ttttttccttgggaccatcaacttatctctctgt-------------------------------------      21 F1S10-P32-TRINITY_DN25_c1_g1_i1-7.6e+03-odorranain-A9                               74.2%  92.8%    ------------------------------------------------------------------------------------aagatgttcaccttgaa------------------gaaatccctgttactcct-------------------------------------------tttctttcttgggaccatctccttatctctctgt-------------------------------------      18 F1S4-P4-TRINITY_DN836_c0_g1_i2-1.3e+01-andersonin-X-OG1                             83.9%  91.0%    ---------------------------------------------------------cc------------------gagcccaaagatgttcacctttaa------------------gaaatccctgttactcct-------------------------------------------tttctttcttgggaccatctccttatctctctgt-------------------------------------      10 F1S17-P57-TRINITY_DN38944_c0_g1_i1-6.3e-01-odorranain-O4                            93.5%  90.8%    -----------------------------------------ctg-------atctgcgt------------------gagcccaaagatgttcaccttgaa------------------gaaatccctgttactcct-------------------------------------------tttcttccttggaaccatcaacttatctctctgt-------------------------------------      35 F1S10-P34-TRINITY_DN6115_c1_g1_i1-2.5e+03-odorranain-A11                           100.0%  89.6%    --------------------------------cagcaccaactg-------aactaccc------------------gagcccaaagatgttcaccatgaa------------------gaaatccctgttactcct-------------------------------------------tttctttcttgggaccatctccttatctctctgt-------------------------------------       8 F1S36-P83-TRINITY_DN14764_c0_g1_i2-4.9e+02-odorranain-X5a                          100.0%  89.6%    --------------------------------cacaaccaactg-------aaccaccc------------------gagcccaaagatgttcaccatgaa------------------gaaatccctgttactcct-------------------------------------------ttttttccttgggaccatcaacttatctctctgt-------------------------------------      29 F1S8-P23-TRINITY_DN96_c0_g2_i1-3.5e-01-esculentin-2-OG21                            71.0%  89.4%    ---------------------------------------------------------------------------------------atgttcaccttgaa------------------gaaatccttgttactctt-------------------------------------------tttctttcttgggaccatctccttatctctctgt-------------------------------------      77 F1S18-P58-TRINITY_DN5345_c0_g1_i2-5.3e+03-odorranain-P1b                           100.0%  88.5%    ----------------------------------gcaccaactgc-----caagtcctc------------------gagcccaaagatgttcaccttgaa------------------gaaatcactgttactcct-------------------------------------------tttcttgcttgggaccatcaacttatctctctgt-------------------------------------      52 F1S11-P37-TRINITY_DN56_c1_g1_i1-5.0e+01-odorranain-B7                               93.5%  87.4%    -----------------------------------------ctg-------aactaccc------------------gagcccaaaaatgttcaccttgaa------------------gaaacccctgttactcct-------------------------------------------tttctttcttgggagcgtctccttatctgtctgt-------------------------------------      86 F1S23-P68-TRINITY_DN128039_c0_g1_i1-2.6e+03-odorranain-U3                           74.2%  87.0%    ------------------------------------------------------------------------------------aagatgtgcaccgggaa------------------gaaatccctgttactcct-------------------------------------------tttctttcttgtgagcatcgccttatctctctgt-------------------------------------      53 F1S11-P38-TRINITY_DN1399_c4_g1_i1-2.5e+01-odorranain-B8                             96.8%  86.7%    --------------------------------------cagctg-------aactaccc------------------gagcccaaaaatgttcaccttgaa------------------gaaacccctgttactcct-------------------------------------------tttctttcttgggatcgtcgccttatctgtctgt-------------------------------------      40 F1S12-P42-TRINITY_DN1218_c4_g1_i1-2.4e+00-odorranain-C12                            90.3%  86.0%    ------------------------------------------tg-------aactaccc--------------------gtccaaagatgttcaccatgca------------------gaaatccctgttactcct-------------------------------------------tttctttcttggggccatctccttatctctctgt-------------------------------------      41 F1S12-P41-TRINITY_DN10924_c1_g1_i1-2.4e+00-odorranain-C11                           74.2%  85.5%    ------------------------------------------------------------------------------------aagatgttcaccatgaa------------------gaaatacctgttagtcct-------------------------------------------tttctttcttgggatcgtctccttatctctctgt-------------------------------------      60 F1S12-P43-TRINITY_DN2658_c0_g2_i1-3.3e-01-odorranain-C13                            74.2%  85.5%    ------------------------------------------------------------------------------------aagatgttcaccatgaa------------------gaaacccctgttactccc-------------------------------------------tttctttcttcggaccatctccttatctctctgc-------------------------------------      74 F1S7-P14-TRINITY_DN4249_c0_g1_i1-2.1e+03-esculentin-1-OG13                          93.5%  85.1%    -----------------------------------------ctg-------aactaccc------------------gagcccaaagatgttcaccttgaa------------------gaaacccctgttactgat-------------------------------------------tgtccttcttgggatcatctccttagctctctgt-------------------------------------      81 F1S8-P19-TRINITY_DN2168_c4_g1_i1-6.8e+00-esculentin-2-OG17                         100.0%  84.9%    -----------------------------------caccaaccg-------aactaccc------------------gagcccaaagatgttaaccatgaa------------------gaaatgcatgttagtcct-------------------------------------------tttctttcgtgggaccatctccttgtctctctgt-------------------------------------      43 F1S13-P46-TRINITY_DN10285_c0_g1_i1-3.0e+00-odorranain-F2                            96.8%  84.8%    ------------------------------------accaactg-------atctaccc--------------------gtccaaagatgttcaccatgaa------------------gaaatccctgttagtcct-------------------------------------------tttctttcttgggatcgtctccttatctctctgt-------------------------------------      80 F1S6-P11-TRINITY_DN6490_c1_g1_i1-8.1e+00-brevinin-2E-OG8                            96.8%  84.8%    ------------------------------------accaactg-------aactaccc--------------------gtccaaagatgttcaccatgaa------------------gaaatccgtgttactcct-------------------------------------------tttctttcttgggacaatatcaatatctctctgt-------------------------------------      82 F1S9-P28-TRINITY_DN4414_c6_g1_i1-5.3e+00-nigrocin-OG33                             100.0%  84.4%    --------------------------------cagcccccactg-------aaatactg------------------gggcccaatgatgttccccttgaa------------------gaaatccctgttactcct-------------------------------------------tttcttccttgggaccattaacttatctctctgt-------------------------------------      87 F1S9-P29-TRINITY_DN16_c2_g1_i1-5.9e+00-nigrocin-OG34                               100.0%  84.4%    --------------------------------cagaaccaactg-------aaccacca------------------gagcccacagatgttatcgttgaa------------------gaaatccctgttacacct-------------------------------------------tttcttccttgggaccatcaacttatccctctgt-------------------------------------      54 F1S11-P36-TRINITY_DN79_c1_g3_i1-3.4e+03-odorranain-B6                              100.0%  82.8%    -------------------------t----ctccgcaccaactg-------aactaccc------------------gagcccaaagatgttcaccttgca------------------gaaacccctgttactcct-------------------------------------------tttctttcttgggatcgtctccttatctttctgt-------------------------------------      88 F1S12-P44-TRINITY_DN2213_c1_g1_i1-5.8e+00-odorranain-C14                            93.5%  82.8%    -----------------------------------------atg-------aactaacc------------------gagcccaaagatgttcacctttag------------------gaaatcccgggtactcct-------------------------------------------tctggttcttgggaccatctccttatctctctgt-------------------------------------      89 F1S12-P45-TRINITY_DN2213_c1_g1_i2-2.4e+00-odorranain-C15                            93.5%  82.8%    -----------------------------------------atg-------aactaacc------------------gagcccaaagatgttcacctttag------------------gaaatcccgggtactcct-------------------------------------------tctggttcttgggaccatctccttatctctctgt-------------------------------------      83 F1S18-P59-TRINITY_DN38049_c0_g1_i1-9.8e+01-odorranain-P1i                          100.0%  81.8%    -------------------------------ccagcaccaactgc-----caagttctc------------------gacccccacgatgttccccttgaa------------------gacatctctgttactcct-------------------------------------------tttcttccttcggaccatcaacttatctctctgt-------------------------------------      64 F1S16-P55-TRINITY_DN3181_c1_g1_i1-3.5e+02-odorranain-M4                             71.0%  81.8%    ------------------------------------------------------------------------------agcccaaagatgttcaccttgaa------------------gaaattcctattgctcct-------------------------------------------tttctttcttgggatcgtctcctca----------------------------------------------      75 F1S7-P15-TRINITY_DN12856_c2_g1_i1-2.7e-01-esculentin-1-OG14                         93.5%  80.5%    -----------------------------------------cgg-------gactactc------------------gagcccaaagatgttcaccttgaa------------------gaaacccctgatactgat-------------------------------------------tgtccttcttgggatcatctccttatccctctgt-------------------------------------      58 F1S34-P81-TRINITY_DN17503_c0_g1_i1-1.2e+00-odorranain-X3a                          100.0%  80.4%    ----------------------catt----ctcatcaccaactg-------aactaccc------------------gatccagaagatgttcaccttgaa------------------gaaatccatgttactcct-------------------------------------------tttctttcttggagccatctcattatctctctgt-------------------------------------       2 F1S14-P50-TRINITY_DN603_c2_g1_i1-5.0e+02-odorranain-G1                             100.0%  80.2%    -------------ttcttctgcattt----ctcagtaccaactg-------aactacct------------------gaacccaaagatgctcaccttgaa------------------gaaatccctgttactcct-------------------------------------------tttcttccttgcgaccatcaacttatctctctgt-------------------------------------       4 F1S9-P26-TRINITY_DN0_c1_g1_i2-2.2e+04-nigrocin-2GRc                                100.0%  80.2%    -------------agctgtccacatt----ctcatcaccaactg-------aactaccc------------------gatcccgaagatgttcaccttgaa------------------gaaatccctgttactcct-------------------------------------------tttcttccttgggaccatcaacttatctctctgt-------------------------------------      13 F1S19-P61-TRINITY_DN4628_c1_g1_i1-1.2e+00-odorranain-P2d                           100.0%  80.0%    -------------------ctacatt----ctcagcaccaactg-------aaccaccc------------------gagcccaaagatgttcaccaggaa------------------gaaatccctgttactcct-------------------------------------------ttttttccttgggaccatcgacttatgtctctgt-------------------------------------      50 F1S32-P79-TRINITY_DN13210_c0_g1_i1-9.0e+00-odorranain-X1a                          100.0%  80.0%    -------------------ctacatt----ctcagcaccaactg-------aactaccc------------------gagcccaaagatgttcaccttgaa------------------gaaatccctagtactcct-------------------------------------------tttctttcttgggaccatctccttaactctctgt-------------------------------------       5 F1S9-P25-TRINITY_DN49_c0_g1_i1-5.8e+03-nigrocin-2GRb                               100.0%  79.3%    -------------agctgtttacatt----ctcagcaccaactg-------aactacct------------------gagcccaaagatgttcaccttgaa------------------gaaatccctgttactcct-------------------------------------------tttctttcttgggaccatcaacttatctctctgt-------------------------------------       6 F1S5-P7-TRINITY_DN23816_c1_g1_i1-4.5e+02-brevinin-1E-OG9                           100.0%  79.3%    -------------agctgtctacatt----ctcagcaccaactg-------aactaccc------------------gagcccaaagatgttcaccatgaa------------------gaaatccctgttactcct-------------------------------------------ttttttccttgggaccatcaacttatctctctgt-------------------------------------      15 F1S2-P2-TRINITY_DN142_c0_g1_i5-5.0e+01-andersonin-R                                100.0%  79.0%    -------------------ctacatt----ctcagcaccaactg-------aactaccc------------------gagctcaaagatgttcaccttgaa------------------gaaatccctgttactcct-------------------------------------------tttcttcattggaatgatctccttatctctctgt-------------------------------------      12 F1S17-P56-TRINITY_DN122946_c2_g1_i1-1.8e+03-odorranain-O1                          100.0%  78.4%    -------------agctgtctacaat----ctcagcaccaactg-------aactatcc------------------gagcgcaaagatgttcaccttgaa------------------gaaatccctgttactcct-------------------------------------------tttcttccttggaaccatcaacttatctctctgt-------------------------------------      26 F1S27-P74-TRINITY_DN139_c0_g1_i1-3.1e+02-OGC-RA3                                   100.0%  78.4%    -------------agctgtctacatt----ctcaccaccaactg-------aactaccc------------------gagcccaaagatgttcaccttgaa------------------gaaatccctgttactcct-------------------------------------------tttctttcttgggaccatctccttatctctctgt-------------------------------------      67 F1S23-P67-TRINITY_DN12170_c0_g1_i1-1.1e+00-odorranain-U2                            64.5%  78.3%    ------------------------------------------------------------------------------------aagatgttcacctttaa------------------gaaattcctattgctcct-------------------------------------------tttctttcttgggatcgcctcctca----------------------------------------------      61 F1S15-P51-TRINITY_DN45_c1_g1_i1-3.1e+03-odorranain-L2                               90.3%  77.8%    ---------------------------------------------agagataaggagat------------------ggtcccaaagatgttcaccatgac------------------gaaatccctgttactcct-------------------------------------------tttctttcttgggaccatctccttatctctctgt-------------------------------------      19 F1S6-P9-TRINITY_DN0_c1_g1_i4-9.2e-01-brevinin-2GRa                                 100.0%  77.5%    -------------agctgtccacatt----ctcatcaccaactg-------aactaccc------------------gatcccgaagatgttcaccttgaa------------------gaaatccctgttactcct-------------------------------------------tttctttcttgggaccatctccttatctctctgt-------------------------------------      20 F1S6-P9-TRINITY_DN0_c1_g1_i14-1.3e+04-brevinin-2GRa                                100.0%  77.5%    -------------agctgtccacatt----ctcatcaccaactg-------aactaccc------------------gatcccgaagatgttcaccttgaa------------------gaaatccctgttactcct-------------------------------------------tttctttcttgggaccatctccttatctctctgt-------------------------------------      22 F1S12-P39-TRINITY_DN0_c1_g1_i10-8.0e+03-brevinin-2GRb                              100.0%  77.5%    -------------agctgtccacatt----ctcatcaccaactg-------aactaccc------------------gatcccgaagatgttcaccttgaa------------------gaaatccctgttactcct-------------------------------------------tttctttcttgggaccatctccttatctctctgt-------------------------------------      23 F1S12-P39-TRINITY_DN0_c1_g1_i11-1.7e+00-brevinin-2GRb                              100.0%  77.5%    -------------agctgtccacatt----ctcatcaccaactg-------aactaccc------------------gatcccgaagatgttcaccttgaa------------------gaaatccctgttactcct-------------------------------------------tttctttcttgggaccatctccttatctctctgt-------------------------------------      24 F3-P86-TRINITY_DN6_c0_g1_i12-1.0e+03-tachykinin_OG1                                100.0%  76.6%    -------------agctgtctacatt----ctcatcaccaactg-------atctaccc------------------gatcccgaagatgttcaccttgaa------------------gaaatccctgttactcct-------------------------------------------tttctttcttgggaccatctccttatctctctgt-------------------------------------      25 F3-P87-TRINITY_DN6_c0_g1_i6-8.0e+02-ranamargarin                                   100.0%  76.6%    -------------agctgtctacatt----ctcatcaccaactg-------atctaccc------------------gatcccgaagatgttcaccttgaa------------------gaaatccctgttactcct-------------------------------------------tttctttcttgggaccatctccttatctctctgt-------------------------------------      37 F1S19-P62-TRINITY_DN638_c0_g1_i2-3.9e+00-odorranain-P2e                            100.0%  76.6%    -------------tataggctacatt----ctcagcaccaactg-------aactacct------------------gagcccaaagatgttcaccttgaa------------------gaaacccctgttactcct-------------------------------------------tttctttcttgggaccatctccttatctctctgt-------------------------------------      44 F1S13-P47-TRINITY_DN1102_c1_g1_i1-1.1e+00-odorranain-F3                            100.0%  75.9%    ----------------cggctacatt----ctcagcaccaactg-------aactatcc------------------gagcccaaagatgttcaccatgaa------------------gaaatccctgttagtcct-------------------------------------------tttctttcttgggatcgtctccttatctctctgt-------------------------------------      14 F1S19-P60-TRINITY_DN39_c0_g1_i2-7.2e+00-odorranain-P2c                             100.0%  75.7%    -------------attggtctacatt----cttagttccagctg-------aaccacca------------------gagcccaaagatgttcaccttgaa------------------gaaatccctgttactcct-------------------------------------------gttcttccttgggaccatcaacttatctctctgt-------------------------------------      27 F1S24-P69-TRINITY_DN122936_c0_g1_i1-4.3e+02-odorranalectin                         100.0%  75.7%    -------------agctgtctacatt----ctcagcaccaacta-------gactaccc------------------gagcccaaagatgttcaccttgaa------------------gaaatccctgttactcct-------------------------------------------tttctttcttgggatcatctccttatctctctgt-------------------------------------      30 F1S25-P70-TRINITY_DN1048_c0_g1_i1-1.9e+02-odorranaopin                             100.0%  75.7%    -------------ggttgtctacatt----ctcagcaccaactg-------aactaccc------------------gagtccaaagatgttcaccttgaa------------------gaaatccttgttacttct-------------------------------------------tttctttcttgggaccatctccttatctctctgt-------------------------------------      31 F1S20-P63-TRINITY_DN132_c0_g1_i4-8.5e+02-odorranain-Q1                             100.0%  75.7%    -------------ggctatctacatt----ctcagcaccaattg-------aactaccc------------------aagcccaaagatgttcaccttgaa------------------gaaatccctgttactcct-------------------------------------------tttctttcttggaaccatctccttatctctctgt-------------------------------------      32 F1S26-P71-TRINITY_DN132_c0_g1_i3-1.8e+02-ishikawain-7-EV1                          100.0%  75.7%    -------------ggctatctacatt----ctcagcaccaattg-------aactaccc------------------aagcccaaagatgttcaccttgaa------------------gaaatccctgttactcct-------------------------------------------tttctttcttggaaccatctccttatctctctgt-------------------------------------      36 F1S24-P69-TRINITY_DN106_c6_g1_i1-2.1e+01-odorranalectin                            100.0%  75.7%    -------------tgtgatctacatt----ctcagcaccaactg-------aaccaccc------------------gagcccaaagatgttcaccatgaa------------------gaaatccctgttactcct-------------------------------------------tttctttcttgggatcatctccttatctctctgt-------------------------------------      55 F1S5-P6-TRINITY_DN0_c1_g1_i24-1.9e+03-brevinin-1E-OG3                              100.0%  75.7%    -------------agctgtccacatt----ctcatcaccaactg-------aactaccc------------------gatcccgaagatgttcaccttgaa------------------gaaatccatgttactcct-------------------------------------------tttctttcttggaaccatctcattatctctctgt-------------------------------------      56 F1S7-P12-TRINITY_DN0_c1_g1_i16-1.0e+00-esculentin-1-OG5                            100.0%  75.7%    -------------agctgtccacatt----ctcatcaccaactg-------aactaccc------------------gatcccgaagatgttcaccttgaa------------------gaaatccatgttactcct-------------------------------------------tttctttcttggaaccatctcattatctctctgt-------------------------------------      57 F1S9-P26-TRINITY_DN0_c1_g1_i17-1.5e+04-nigrocin-2GRc                               100.0%  75.7%    -------------agctgtccacatt----ctcatcaccaactg-------aactaccc------------------gatcccgaagatgttcaccttgaa------------------gaaatccatgttactcct-------------------------------------------tttctttcttggaaccatctcattatctctctgt-------------------------------------       9 F1S9-P27-TRINITY_DN9643_c0_g1_i4-2.5e+00-nigrocin-OG32                             100.0%  75.7%    -------------agctgtctacatt----ctcagaaccaactg-------aaccaccc------------------gagtccaaagatgttcaccatgaa------------------gaaatccctgttactcct-------------------------------------------ttttttccttgggaccatcaacttatctatctgt-------------------------------------      79 F1S9-P24-TRINITY_DN1399_c0_g1_i1-4.6e+01-nigrocin-2GRa                             100.0%  75.4%    --------------------------------aagcaccagctg-------aactaccc------------------gagcacaaagatgttcaccttgaa------------------gaaatccctgttcctcct-------------------------------------------tttcttccttgggaccatcaacttatctctctggcaggatgagacaaatgcc-------------------      16 F1S12-P40-TRINITY_DN45_c27_g1_i1-7.5e+02-odorranain-C7                             100.0%  74.8%    -------------agctgtctacatt----ctcagcaccagctg-------aactaccc------------------gagcccaaagatgttcaccttgaa------------------gaaatccctcttactcct-------------------------------------------tttctttattggaaccatctccttatctctctgt-------------------------------------      17 F1S29-P76-TRINITY_DN8472_c0_g1_i1-2.5e+03-palustrin-OG2                            100.0%  74.8%    -------------agctgtctacatt----ctcagcaccagctg-------aactaccc------------------gagcccaaagatgttcaccttgaa------------------gaaatccctcttactcct-------------------------------------------tttctttattggaaccatctccttatctctctgt-------------------------------------      33 F1S8-P17-TRINITY_DN96_c0_g2_i2-1.4e+02-esculentin-2-OG8                            100.0%  74.8%    -------------agctgtctacatt----ctcagcaccaacca-------aactaccc------------------aagcccaaagatgttcaccttgaa------------------gaaatccctgttactcct-------------------------------------------tttctttcttgggaccatatccttatctctctgt-------------------------------------      34 F1S8-P22-TRINITY_DN96_c0_g1_i1-6.4e+00-esculentin-2-OG20                           100.0%  74.8%    -------------agctgtctacatt----ctcagcaccaacca-------aactaccc------------------aagcccaaagatgttcaccttgaa------------------gaaatccctgttactcct-------------------------------------------tttctttcttgggaccatatccttatctctctgt-------------------------------------      38 F1S26-P71-TRINITY_DN132_c0_g1_i1-3.5e+02-ishikawain-7-EV1                          100.0%  74.8%    -------------ggctatctacatt----ctcagcaccaattg-------aactaccc------------------aagcccaaagatgttcaccttgaa------------------gaaaaccctgttaatcct-------------------------------------------tttctttcttgggaccatctccttatctctctgt-------------------------------------      39 F1S26-P72-TRINITY_DN132_c0_g1_i5-2.4e+02-OGA1                                      100.0%  74.8%    -------------ggctatctacatt----ctcagcaccaattg-------aactaccc------------------aagcccaaagatgttcaccttgaa------------------gaaaaccctgttaatcct-------------------------------------------tttctttcttgggaccatctccttatctctctgt-------------------------------------      28 F1S8-P16-TRINITY_DN96_c0_g1_i2-2.3e+01-esculentin-2-RA1                            100.0%  73.9%    -------------agctgtctacatt----ctcagcaccaacca-------aactaccc------------------aagcccaaagatgttcaccttgaa------------------gaaatccttgttactctt-------------------------------------------tttctttcttgggaccatctccttatctctctgt-------------------------------------      69 F1S9-P24-TRINITY_DN77_c0_g1_i1-7.4e+01-nigrocin-2GRa                               100.0%  73.9%    -------------agctgtctacatt----gtcagcaccaactg-------aaccaccc------------------gagccgaaagatgttcaccttgaa------------------gaaatccatgttactcct-------------------------------------------ttgctttcttggaaccatctcattatctctctgt-------------------------------------      78 F1S35-P82-TRINITY_DN360_c0_g1_i1-7.9e+02-odorranain-X4a                            100.0%  73.9%    -----------------------------------caccaactg-------aactacccgattccaaattatccacagagctcaaagatgttcaccttgaa------------------gaaatccctattattcct-------------------------------------------tttctttcttgggatcatctccttctctctctgt-------------------------------------      42 F1S13-P46-TRINITY_DN6_c27_g1_i1-4.8e+03-odorranain-F2                              100.0%  73.0%    -------------agctgtctacatt----ctcagcaccaactg-------aactactc------------------gagtccaaagatgttcaccatgaa------------------gaaatccctgttagtcct-------------------------------------------tttctttcttgggatcgtctccttatctctctgt-------------------------------------      73 F1S7-P12-TRINITY_DN81_c0_g1_i1-9.5e+03-esculentin-1-OG5                            100.0%  73.0%    -------------agctgtctgcatt----ctcagcaccaactg-------aactaccc------------------gagcccaaagatgttcaccttgaa------------------gaaacccctgttactgat-------------------------------------------tgtccttcttgggatcatctccttatctctctgt-------------------------------------      45 F1S8-P18-TRINITY_DN0_c1_g1_i22-8.3e+03-esculentin-2-OG10                            96.8%  72.8%    ----------tccagctgtctacatt----ctcaacaccaactg-------aactac---------------------agcccaaagatgttcaccttgaa------------------taaatccctgttactcct-------------------------------------------tttctttcttgggaccatctccttatctctctgt-------------------------------------      46 F1S12-P39-TRINITY_DN0_c1_g1_i15-2.2e+01-brevinin-2GRb                               96.8%  72.8%    ----------tccagctgtctacatt----ctcaacaccaactg-------aactac---------------------agcccaaagatgttcaccttgaa------------------taaatccctgttactcct-------------------------------------------tttctttcttgggaccatctccttatctctctgt-------------------------------------      47 F1S12-P39-TRINITY_DN0_c1_g1_i23-8.4e+00-brevinin-2GRb                               96.8%  72.8%    ----------tccagctgtctacatt----ctcaacaccaactg-------aactac---------------------agcccaaagatgttcaccttgaa------------------taaatccctgttactcct-------------------------------------------tttctttcttgggaccatctccttatctctctgt-------------------------------------      48 F1S22-P65-TRINITY_DN98_c53_g1_i1-3.6e+03-odorranain-T1                              96.8%  72.8%    ----------tccagctgtctacatt----ctcaacaccaactg-------aactac---------------------agcccaaagatgttcaccttgaa------------------taaatccctgttactcct-------------------------------------------tttctttcttgggaccatctccttatctctctgt-------------------------------------      49 F1S28-P75-TRINITY_DN0_c1_g1_i20-5.5e+03-OGTI                                        96.8%  72.8%    ----------tccagctgtctacatt----ctcaacaccaactg-------aactac---------------------agcccaaagatgttcaccttgaa------------------taaatccctgttactcct-------------------------------------------tttctttcttgggaccatctccttatctctctgt-------------------------------------      70 F1S5-P6-TRINITY_DN0_c1_g1_i6-6.1e+02-brevinin-1E-OG3                               100.0%  72.1%    -------------agctgtccacatt----ctcatcaccaactg-------aactaccc------------------gatcccgaagatgttcaccttgaa------------------gaaacccctgttactgat-------------------------------------------tgtccttcttgggatcatctccttatctctctgt-------------------------------------      71 F1S9-P26-TRINITY_DN0_c1_g1_i3-1.2e+00-nigrocin-2GRc                                100.0%  72.1%    -------------agctgtccacatt----ctcatcaccaactg-------aactaccc------------------gatcccgaagatgttcaccttgaa------------------gaaacccctgttactgat-------------------------------------------tgtccttcttgggatcatctccttatctctctgt-------------------------------------      51 F1S11-P35-TRINITY_DN11239_c0_g1_i2-7.5e+03-odorranain-B1                           100.0%  71.9%    -------------agctgtctacact----ctcagcaccaactg-------aactaccc------------------gagcccaaaaatgttcaccttgaa------------------gaaacccctgttactcct-------------------------------------------tttctttcttgggatcgtctccttatctgtctgtggt----------------------------------      68 F1S21-P64-TRINITY_DN638_c6_g1_i1-2.4e+02-odorranain-S1                             100.0%  71.2%    -------------agctgtctacatt----ctcagcatcaactg-------aactatcc------------------aagcgcaacaatgttcaccttgaa------------------gaaatccctgttactcct-------------------------------------------tttctttctgggggccatctccttatctctctgt-------------------------------------      72 F1S7-P12-TRINITY_DN0_c1_g1_i18-2.7e+03-esculentin-1-OG5                            100.0%  71.2%    -------------agctgtccacatt----ctcatcaccaactg-------aactaccc------------------gatcccgaagatgttcaccttgaa------------------gaaacccctgttactgat-------------------------------------------tgtccttcttgggatcatctccctatctctctgt-------------------------------------      62 F1S3-P3-TRINITY_DN25_c0_g1_i2-5.2e+02-andersonin-S                                  90.3%  69.6%    -------------agctgtctacatt----ctaagcaccagctg-------aactaccc------------------gagcccaaagatgttcaccttgaa------------------gaaattcctattgctcct-------------------------------------------tttctttcttgggatcgtctcctca----------------------------------------------      63 F1S16-P54-TRINITY_DN25_c0_g1_i3-2.7e+03-odorranain-M3                               90.3%  69.6%    -------------agctgtctacatt----ctaagcaccagctg-------aactaccc------------------gagcccaaagatgttcaccttgaa------------------gaaattcctattgctcct-------------------------------------------tttctttcttgggatcgtctcctca----------------------------------------------      65 F1S33-P80-TRINITY_DN1399_c2_g1_i1-7.7e+00-odorranain-X2a                            90.3%  69.6%    -------------agctgtctacatt----ctaagcaccagctg-------aactaccc------------------gagcccaaagatgttcaccttgaa------------------gaaattcctattgctcct-------------------------------------------tttctttcttgggatcgtctcctca----------------------------------------------      66 F1S16-P53-TRINITY_DN25_c0_g1_i1-2.1e+03-odorranain-M2                               90.3%  69.6%    -------------agctgtctacatt----ctaagcaccagctg-------aactaccc------------------gagcccaaagatgttcaccttgaa------------------gaaattcctgttgctcct-------------------------------------------tttctttcttgggattgtctcctca----------------------------------------------      84 F1S30-P77-TRINITY_DN0_c174_g2_i1-9.7e+03-pleurain-E-OG1                            100.0%  67.5%    ----------------tgtctacatt----ctcagcaccaaccg-------aactaccc------------------gagcccaaagatgttatccttgaa------------------gacatccctgttactcct-------------------------------------------tttctttattgggattgtctcctcatctccctgtcgagga-------------------------------      91 F1S28-P75-TRINITY_DN603_c0_g1_i3-5.1e-01-OGTI                                       93.5%  66.7%    -----------------------------------------ctg-------cactacaa------------------gagcgataaaatgttcgcaatgaa------------------gaaatcactgttcgtccg-------------------------------------------attgtgtgttggggggatccaattatctctctggggt----------------------------------      59 F1S10-P31-TRINITY_DN7347_c0_g1_i1-4.9e+03-odorranain-A8                             90.3%  65.8%    a----agttctccagctgtcaacatt----ctcatcaccaactg-------aactaccc---------------------------gatgttcaccttgaa------------------gaaatccctgttactcct-------------------------------------------tttctttcttgggaccatctccttatctctctgt-------------------------------------      76 F1S7-P13-TRINITY_DN259_c0_g1_i1-1.7e+02-esculentin-1-OG12                          100.0%  64.9%    -------------agctgtgtacatc----ccgggcgccgccgg-------aactaccc------------------aagaccaaagatgttcaccttgaa------------------gaaacccctgttactgat-------------------------------------------tgtccttcttgggatcatctccttatcccaatgt-------------------------------------      85 F1S10-P33-TRINITY_DN25595_c0_g1_i1-3.1e+00-odorranain-A10                           90.3%  60.8%    actgtggcaacaaggcgctttattgt----c--------------------tactatcc------------------gagcccaaagatgttcaccatgac------------------gaaatccctgttactgct-------------------------------------------tttctttcttgggaccatctccttatctctctgt-------------------------------------      90 F1S28-P75-TRINITY_DN603_c0_g1_i1-4.5e+01-OGTI                                      100.0%  56.6%    -------------agctgtctacatt----ctcagcaccagctg-------aactaccc------------------gagcccaaagatgttcaccatgaa------------------gaaatccatgttactcct-------------------------------------------attatttgttggggtgatcttcgggtccctctgggaggaacatagagatgcc-------------------     116 F14-P102-TRINITY_DN17623_c0_g1_i1-3.4e-01-C-X-C_motif_chemokine_8-OG1              100.0%  36.8%    -----------------------------gaaaaccacagcttc-------agtcataaact---------------gacaagaacacttctcacaatgaa------------------agctacactgtgtatcct-------------------------------------------tgcagttctggcagtcttcctgacatgttttactctctcagaaggg-------------------------     117 F14-P103-TRINITY_DN17623_c0_g1_i2-1.2e+01-C-X-C_motif_chemokine_8-OG2              100.0%  36.8%    -----------------------------gaaaaccacagcttc-------agtcataaact---------------gacaagaacacttctcacaatgaa------------------agctacactgtgtatcct-------------------------------------------tgcagttctggcagtcttcctgacatgttttactctctcagaaggg-------------------------      96 F5-P89-TRINITY_DN62991_c0_g1_i1-4.9e+02-amotoxin-OG                                 95.7%  35.7%    --------------------------------caacaccaactctc-----cattgtgg----------------------taaacaagacacgagactacaacat-------------gaaaaccacattgctcct-------------------------------------------tgctgttattgccaccagtctcttgatgtttcagctcacctctgca-------------------------     100 F12-P99-TRINITY_DN46746_c0_g1_i2-1.3e+00-mesotocin-neurophysin_MT                   89.2%  34.9%    -----------------------------------------ctttc-----aaatacag----------------------tcaataatcttgacactagc------------------aacatgatagccatgact---tatagctccct------------------------ggctgccagcttcttctgcctgttagccctttcctcagca------------------------------------     118 F11-P98-TRINITY_DN52727_c0_g1_i1-4.9e-01-galanin                                    92.5%  34.7%    ---------------------------tcaagggacagaaactgc------aaacacac------------------ctacccaaatcagctcaagatgga------------------aaagtgcacaagtctgct--------------------------------------------------tctggtgtctttaatactgtgtgccacaatctcacagacatttgga------------------      99 F2-P84-TRINITY_DN9272_c0_g2_i1-2.5e+01-pro-FMRFamide-related_neuropeptide_FF-OG1    86.0%  34.0%    ---------------------------------------------------aattacaa----------------------agacagaattcgacacctgacata-----------cacagccatggggatagtcac-------------------------------------------cctcctcttcatcgccttcctctcctgtgccagaacg----------------------------------     120 F9-P95-TRINITY_DN24491_c0_g1_i1-1.0e+02-calcitonin-like_peptide_1-OG1               92.5%  31.4%    g--------------------------------------aactctct-ttggattatccctctcg------------caacaggaaccggtccataat---------------------gaagttggcttacatcat-------------------------------------------cgtgctcctcataagttgtctgaccttccttggctccaccatggca-------------------------      97 F14-P104-TRINITY_DN36485_c0_g1_i1-4.4e+00-C-X-C_motif_chemokine_10-OG1              89.2%  31.3%    ---gt----------------------taaaccagcatttcttc---------------------------------tagtccaggtaaacctgttctacac-----------------gatggccagaatactcat-------------------------------------------tgctgtcctgggcaca--ctgctcattcttcagtccgtgcaagga--------------------------      98 F14-P105-TRINITY_DN36485_c0_g1_i3-2.0e-01-C-X-C_motif_chemokine_10-OG2              89.2%  31.3%    ---gt----------------------taaaccagcatttcttc---------------------------------tagtccaggtaaacctgttctacac-----------------gatggccagaatactcat-------------------------------------------tgctgtcctgggcaca--ctgctcattcttcagtccgtgcaagga--------------------------     114 F3-P85-TRINITY_DN3001_c0_g1_i6-3.0e-01-ranatachykinin-A                             87.1%  30.7%    ---------------------------------agcgccggtcccgt----------------------------------tcaaagggttctgtggcgaaagcg--------------ggaaagcatgaagatcct-------------------------------------------cgtagcctttgctgtcattatgttggtttcagcgcaagtgtttgcg-------------------------     115 F3-P85-TRINITY_DN3001_c0_g1_i4-3.6e+00-ranatachykinin-A                             87.1%  30.7%    ---------------------------------agcgccggtcccgt----------------------------------tcaaagggttctgtggcgaaagcg--------------ggaaagcatgaagatcct-------------------------------------------cgtagcctttgctgtcattatgttggtttcagcgcaagtgtttgcg-------------------------     101 F8-P92-TRINITY_DN593_c2_g1_i4-3.3e+00-odorranain-BLP-4                             100.0%  30.2%    ----------------------------------gcatccctca-------gactctca------------------catctctacagagcacacagcaca------------------gacatgactgcagttcctgccatcagaatcctgcc--------------------------cattggcttcctggctattctgctgctcttctccgtcatctcccgctct----------------------     102 F8-P92-TRINITY_DN593_c2_g1_i3-4.7e+02-odorranain-BLP-4                             100.0%  30.2%    ----------------------------------gcatccctca-------gactctca------------------catctctacagagcacacagcaca------------------gacatgactgcagttcctgccatcagaatcctgcc--------------------------cattggcttcctggctattctgctgctcttctccgtcatctcccgctct----------------------     103 F8-P94-TRINITY_DN593_c2_g1_i2-4.6e+01-odorranain-BLP-7                             100.0%  29.4%    ----------------------------------gcatccctca-------gactctca------------------catctctacagagcacacagcaca------------------gacatgactgcagttcctggcatcagaatcctgcc--------------------------cgttggcttcctgggtattctgctgctcttctccgtcatctcccgctct----------------------     104 F8-P94-TRINITY_DN593_c2_g1_i1-4.5e+03-odorranain-BLP-7                             100.0%  29.4%    ----------------------------------gcatccctca-------gactctca------------------catctctacagagcacacagcaca------------------gacatgactgcagttcctggcatcagaatcctgcc--------------------------cgttggcttcctgggtattctgctgctcttctccgtcatctcccgctct----------------------     107 F8-P94-TRINITY_DN3333_c1_g1_i3-2.2e+01-odorranain-BLP-7                             81.7%  28.7%    -----------------------------------------------------------------------------catctctacagagcacacagcaca------------------gacatgactgcgggtcctggcatcacaatcctggc--------------------------cgttggcttcctgggtattcttctgctcttctccgtcatctcccgcgct----------------------     119 F6-P90-TRINITY_DN15119_c0_g1_i1-1.9e+02-peptide_YY-like                            100.0%  28.7%    g----------------------------aagcatccttaccttcct-gcacacccccttcctt-------------ccaacagataatggtgacctcctt------------------gaagctctggccaatgat-------------------------------------------ggtagccatcacaatctgcgtcttaatatgtctaggaacgatagtagaggga-------------------     105 F8-P94-TRINITY_DN3618_c0_g1_i1-3.4e+01-odorranain-BLP-7                             94.6%  28.3%    ----------------------------------------ctca-------gactctca------------------gatctctacagagcacgcagcaca------------------gacatgactgcagttcctggcatcagaatcctgcc--------------------------cgttggcttcctgggtattctgctgctcttctccgtcatctcccgctct----------------------      93 F4-P88-TRINITY_DN9548_c0_g1_i2-3.5e-01-progonadoliberin-2                           86.0%  26.9%    ------------------------------acatttgcaaggttcct-gtgaactgtgagtca--------------catctactaaccaatggcctgtca------------------gagacacttgctgctcct--------------------------------------------------------gctcctggttctgttttcagtcagtacacagctgtcacacggc---------------      94 F4-P88-TRINITY_DN9548_c0_g1_i3-1.8e+00-progonadoliberin-2                           86.0%  26.9%    ------------------------------ccaagtgcctggttcct-gtgaactgtgagtca--------------catctactaaccaatggcctgtca------------------gagacacttgctgctcct--------------------------------------------------------gctcctggttctgttttcagtcagtacacagctgtcacacggc---------------     106 F8-P94-TRINITY_DN9594_c0_g1_i2-2.0e+00-odorranain-BLP-7                             72.0%  26.9%    --------------------------------------------------------------------------------------agagcacacagcaca------------------gacatgactgcagttcctggcatcagaatcctgcc--------------------------cgttggcttcctgggtcttctgctgctcttctccgtctcctcc----------------------------      92 F7-P91-TRINITY_DN110119_c0_g1_i1-4.5e-01-7B2_granin_protein                         91.4%  26.7%    ------------------------------aacaggactggctg-----------------------------aatggaattaaaaaacattttgtaggtg------------------acaatgaaacacaatgcc-------------------------------------------atcatctctttggtctgtcccttggtggtattgctggtgtgtgggctgaatccatcctttgg--------t      95 F4-P88-TRINITY_DN9548_c0_g1_i1-6.6e-01-progonadoliberin-2                           86.0%  26.2%    ------------------------------atttaacaaaggttcct-gtgaactgtgagtca--------------catctactaaccaatggcctgtca------------------gagacacttgctgctcct--------------------------------------------------------gctcctggttctgttttcagtcagtacacagctgtcacacggc---------------     121 F14-P101-TRINITY_DN2072_c0_g1_i1-6.2e-01-C-X-C_motif_chemokine_14-OG1               84.9%  25.6%    --------------------------------taaaactgatctcca----tattataagt----------------aacagaataggaattgggacatgaaacct-----ctttatgcagcaatcctattactcat-------------------------------------------tctagcaatttgcacactcc----------------------------------------aagtggaaggg     111 F10-P96-TRINITY_DN988_c0_g1_i1-3.4e+00-OG-CATH1                                     68.8%  25.4%    ---at-----------tatcagggcc----atcaccatcacctt-------caccatccat--------------------cgaaggaagatgaagatctg------------------gcagtgtgtgttatggct--------------------------------------------------------------------ctgcgcagtcacattggaggtggttcactct---------------     112 F10-P96-TRINITY_DN988_c0_g1_i2-2.8e+00-OG-CATH1                                     68.8%  25.4%    ---at-----------tatcagggcc----atcaccatcacctt-------caccatccat--------------------cgaaggaagatgaagatctg------------------gcagtgtgtgttatggct--------------------------------------------------------------------ctgcgcagtcacattggaggtggttcactct---------------     113 F10-P97-TRINITY_DN988_c0_g1_i4-3.8e+00-OG-CATH2                                     68.8%  25.4%    ---at-----------tatcagggcc----atcaccatcacctt-------caccatccat--------------------cgaaggaagatgaagatctg------------------gcagtgtgtgttatggct--------------------------------------------------------------------ctgcgcagtcacattggaggtggttcactct---------------     108 F13-P100-TRINITY_DN98392_c0_g1_i1-3.7e-01-insulin-like_growth_factor_II_isoform_X2  91.4%  18.6%    --------------------------gcagtccgtcctcatcca----------------------------t----caccaggaaaacactcacattggaacatggagcaactaa---ggtgtcacagcagctgcagcagcccgacccctccgtgcaggaggacacagatgccaggagtgccagtcccccgacatgctcttctactcctctacaccttcatagcatacacagcagagtca----------     109 F14-P106-TRINITY_DN5647_c0_g1_i2-5.1e-01-C-X-C_motif_chemokine_11-OG1               74.2%  16.3%    ----t-----------------------------gtgtcagtcc-------tgccatgaaa----------------------------------------------------------caagtccgggcccctgctgtcatcatggacttcaa--------------------------gtgtgctgtcatcgtctgtattctgctctctgctatccttgtacaagga----------------------     110 F14-P107-TRINITY_DN5647_c0_g1_i1-6.3e-01-C-X-C_motif_chemokine_11-OG2               74.2%  16.3%    ----t-----------------------------gtgtcagtcc-------tgccatgaaa----------------------------------------------------------caagtccgggcccctgctgtcatcatggacttcaa--------------------------gtgtgctgtcatcgtctgtattctgctctctgctatccttgtacaagga----------------------         clustal                                                                                                                                                                                                                                                                                                                                                                 consensus/75%                                                                                       ....................................................A.........................A...C.AAGATGTTCACC.TGAA..................GAAATCCCTGTTACTCCT...........................................T.T..T.CTTGG.A.C.TC...TTAT...TCTGT..................................... ``` |

MView 1.67, Copyright © 1997-2020 Nigel P. Brown
