# Supplementary material for: Integrative transcriptomics and peptidomics approach reveals unexpectedly diverse endogenous secretory peptides in Odorrana grahami frog skin
Source: BMC Biol. 2025 Nov 28;23:354. doi: 10.1186/s12915-025-02463-w (PMC12664280; doi:10.1186/s12915-025-02463-w)
Supplement: Supplementary file 5 — Additional file 5. Alignments of ESP sequences identified in this study across different regions. [file 12915_2025_2463_MOESM5_ESM.zip › Additional file 5/All 14 families - signal peptide plus up to 45 nucleotides upstream of the 5’-UTR“translated”.html]

MView


|  |
| --- |
| ``` Reference sequence (1): F1S1-P1-TRINITY_DN175_c1_g1_i1-9.3e+02-andersonin-Q Identities normalised by aligned length. Colored by: consensus group/60% ``` |
| ```                                                                                           cov    pid  1 [        .         .         .         .         :         .         .         .        ] 89   1 F1S1-P1-TRINITY_DN175_c1_g1_i1-9.3e+02-andersonin-Q                                100.0% 100.0%    ------------HQLNYPNP--------KMFTLKK---------------------------SLLLL----FFLATINLSLC-------     23 F1S9-P30-TRINITY_DN11504_c0_g1_i1-4.8e+00-nigrocin-OG35                             87.1%  92.6%    ----------------YPSP--------KMFTLKK---------------------------SLLLL----FFLGTINLSLC-------     42 F1S5-P5-TRINITY_DN23413_c1_g1_i1-1.3e+00-gaegurin-6-OG1                             74.2%  91.3%    ----------------------------KMFTLKK---------------------------SLLLL----FFPGTINLSLC-------      9 F1S10-P32-TRINITY_DN25_c1_g1_i1-7.6e+03-odorranain-A9                               74.2%  91.3%    ----------------------------KMFTLKK---------------------------SLLLL----FFLGTISLSLC-------     21 F1S5-P8-TRINITY_DN33233_c1_g1_i1-1.1e+02-brevinin-1E-OG10                           80.6%  88.0%    ------------------SP--------KMFTMKK---------------------------SLLLL----FFLGTINLSLC-------     17 F1S8-P23-TRINITY_DN96_c0_g2_i1-3.5e-01-esculentin-2-OG21                            71.0%  86.4%    -----------------------------MFTLKK---------------------------SLLLF----FFLGTISLSLC-------      3 F1S4-P4-TRINITY_DN836_c0_g1_i2-1.3e+01-andersonin-X-OG1                             83.9%  84.6%    -----------------PSP--------KMFTFKK---------------------------SLLLL----FFLGTISLSLC-------     25 F1S10-P34-TRINITY_DN6115_c1_g1_i1-2.5e+03-odorranain-A11                           100.0%  84.4%    -----------QHQLNYPSP--------KMFTMKK---------------------------SLLLL----FFLGTISLSLC-------     43 F1S17-P57-TRINITY_DN38944_c0_g1_i1-6.3e-01-odorranain-O4                            93.5%  82.8%    --------------LICVSP--------KMFTLKK---------------------------SLLLL----FFLGTINLSLC-------     22 F1S36-P83-TRINITY_DN14764_c0_g1_i2-4.9e+02-odorranain-X5a                          100.0%  81.2%    -----------HNQLNHPSP--------KMFTMKK---------------------------SLLLL----FFLGTINLSLC-------     15 F1S9-P26-TRINITY_DN0_c1_g1_i2-2.2e+04-nigrocin-2GRc                                100.0%  78.4%    -----SCP-HSHHQLNYPIP--------KMFTLKK---------------------------SLLLL----FFLGTINLSLC-------     76 F1S12-P43-TRINITY_DN2658_c0_g2_i1-3.3e-01-odorranain-C13                            74.2%  78.3%    ----------------------------KMFTMKK---------------------------PLLLP----FFLRTISLSLC-------     82 F1S23-P68-TRINITY_DN128039_c0_g1_i1-2.6e+03-odorranain-U3                           74.2%  78.3%    ----------------------------KMCTGKK---------------------------SLLLL----FFLVSIALSLC-------     56 F1S11-P38-TRINITY_DN1399_c4_g1_i1-2.5e+01-odorranain-B8                             96.8%  76.7%    -------------QLNYPSP--------KMFTLKK---------------------------PLLLL----FFLGIVALSVC-------     57 F1S11-P37-TRINITY_DN56_c1_g1_i1-5.0e+01-odorranain-B7                               93.5%  75.9%    --------------LNYPSP--------KMFTLKK---------------------------PLLLL----FFLGSVSLSVC-------     10 F1S12-P39-TRINITY_DN0_c1_g1_i10-8.0e+03-brevinin-2GRb                              100.0%  75.7%    -----SCP-HSHHQLNYPIP--------KMFTLKK---------------------------SLLLL----FFLGTISLSLC-------     11 F1S12-P39-TRINITY_DN0_c1_g1_i11-1.7e+00-brevinin-2GRb                              100.0%  75.7%    -----SCP-HSHHQLNYPIP--------KMFTLKK---------------------------SLLLL----FFLGTISLSLC-------     12 F1S27-P74-TRINITY_DN139_c0_g1_i1-3.1e+02-OGC-RA3                                   100.0%  75.7%    -----SCL-HSHHQLNYPSP--------KMFTLKK---------------------------SLLLL----FFLGTISLSLC-------     20 F1S5-P7-TRINITY_DN23816_c1_g1_i1-4.5e+02-brevinin-1E-OG9                           100.0%  75.7%    -----SCL-HSQHQLNYPSP--------KMFTMKK---------------------------SLLLL----FFLGTINLSLC-------     26 F1S25-P70-TRINITY_DN1048_c0_g1_i1-1.9e+02-odorranaopin                             100.0%  75.7%    -----GCL-HSQHQLNYPSP--------KMFTLKK---------------------------SLLLL----FFLGTISLSLC-------     27 F1S20-P63-TRINITY_DN132_c0_g1_i4-8.5e+02-odorranain-Q1                             100.0%  75.7%    -----GYL-HSQHQLNYPSP--------KMFTLKK---------------------------SLLLL----FFLGTISLSLC-------     28 F1S26-P71-TRINITY_DN132_c0_g1_i3-1.8e+02-ishikawain-7-EV1                          100.0%  75.7%    -----GYL-HSQHQLNYPSP--------KMFTLKK---------------------------SLLLL----FFLGTISLSLC-------     34 F1S9-P25-TRINITY_DN49_c0_g1_i1-5.8e+03-nigrocin-2GRb                               100.0%  75.7%    -----SCL-HSQHQLNYLSP--------KMFTLKK---------------------------SLLLL----FFLGTINLSLC-------     41 F1S17-P56-TRINITY_DN122946_c2_g1_i1-1.8e+03-odorranain-O1                          100.0%  75.7%    -----SCL-QSQHQLNYPSA--------KMFTLKK---------------------------SLLLL----FFLGTINLSLC-------      7 F1S6-P9-TRINITY_DN0_c1_g1_i4-9.2e-01-brevinin-2GRa                                 100.0%  75.7%    -----SCP-HSHHQLNYPIP--------KMFTLKK---------------------------SLLLL----FFLGTISLSLC-------     77 F1S14-P50-TRINITY_DN603_c2_g1_i1-5.0e+02-odorranain-G1                             100.0%  75.7%    -----FFC-ISQYQLNYLNP--------KMLTLKK---------------------------SLLLL----FFLATINLSLC-------      8 F1S6-P9-TRINITY_DN0_c1_g1_i14-1.3e+04-brevinin-2GRa                                100.0%  75.7%    -----SCP-HSHHQLNYPIP--------KMFTLKK---------------------------SLLLL----FFLGTISLSLC-------     31 F1S32-P79-TRINITY_DN13210_c0_g1_i1-9.0e+00-odorranain-X1a                          100.0%  74.3%    -------L-HSQHQLNYPSP--------KMFTLKK---------------------------SLVLL----FFLGTISLTLC-------     68 F1S34-P81-TRINITY_DN17503_c0_g1_i1-1.2e+00-odorranain-X3a                          100.0%  73.5%    ---------HSHHQLNYPIQ--------KMFTLKK---------------------------SMLLL----FFLGAISLSLC-------     13 F3-P86-TRINITY_DN6_c0_g1_i12-1.0e+03-tachykinin_OG1                                100.0%  73.0%    -----SCL-HSHHQLIYPIP--------KMFTLKK---------------------------SLLLL----FFLGTISLSLC-------     14 F3-P87-TRINITY_DN6_c0_g1_i6-8.0e+02-ranamargarin                                   100.0%  73.0%    -----SCL-HSHHQLIYPIP--------KMFTLKK---------------------------SLLLL----FFLGTISLSLC-------     18 F1S8-P17-TRINITY_DN96_c0_g2_i2-1.4e+02-esculentin-2-OG8                            100.0%  73.0%    -----SCL-HSQHQPNYPSP--------KMFTLKK---------------------------SLLLL----FFLGTISLSLC-------     19 F1S8-P22-TRINITY_DN96_c0_g1_i1-6.4e+00-esculentin-2-OG20                           100.0%  73.0%    -----SCL-HSQHQPNYPSP--------KMFTLKK---------------------------SLLLL----FFLGTISLSLC-------     32 F1S12-P40-TRINITY_DN45_c27_g1_i1-7.5e+02-odorranain-C7                             100.0%  73.0%    -----SCL-HSQHQLNYPSP--------KMFTLKK---------------------------SLLLL----FFIGTISLSLC-------     33 F1S29-P76-TRINITY_DN8472_c0_g1_i1-2.5e+03-palustrin-OG2                            100.0%  73.0%    -----SCL-HSQHQLNYPSP--------KMFTLKK---------------------------SLLLL----FFIGTISLSLC-------      4 F1S5-P6-TRINITY_DN0_c1_g1_i24-1.9e+03-brevinin-1E-OG3                              100.0%  73.0%    -----SCP-HSHHQLNYPIP--------KMFTLKK---------------------------SMLLL----FFLGTISLSLC-------      5 F1S7-P12-TRINITY_DN0_c1_g1_i16-1.0e+00-esculentin-1-OG5                            100.0%  73.0%    -----SCP-HSHHQLNYPIP--------KMFTLKK---------------------------SMLLL----FFLGTISLSLC-------      6 F1S9-P26-TRINITY_DN0_c1_g1_i17-1.5e+04-nigrocin-2GRc                               100.0%  73.0%    -----SCP-HSHHQLNYPIP--------KMFTLKK---------------------------SMLLL----FFLGTISLSLC-------     90 F1S9-P28-TRINITY_DN4414_c6_g1_i1-5.3e+00-nigrocin-OG33                             100.0%  71.9%    -----------QPPLKYWGP--------MMFPLKK---------------------------SLLLL----FFLGTINLSLC-------      2 F1S2-P2-TRINITY_DN142_c0_g1_i5-5.0e+01-andersonin-R                                100.0%  71.4%    -------L-HSQHQLNYPSS--------KMFTLKK---------------------------SLLLL----FFIGMISLSLC-------     72 F1S19-P61-TRINITY_DN4628_c1_g1_i1-1.2e+00-odorranain-P2d                           100.0%  71.4%    -------L-HSQHQLNHPSP--------KMFTRKK---------------------------SLLLL----FFLGTIDLCLC-------     16 F1S8-P16-TRINITY_DN96_c0_g1_i2-2.3e+01-esculentin-2-RA1                            100.0%  70.3%    -----SCL-HSQHQPNYPSP--------KMFTLKK---------------------------SLLLF----FFLGTISLSLC-------     29 F1S26-P71-TRINITY_DN132_c0_g1_i1-3.5e+02-ishikawain-7-EV1                          100.0%  70.3%    -----GYL-HSQHQLNYPSP--------KMFTLKK---------------------------TLLIL----FFLGTISLSLC-------     30 F1S26-P72-TRINITY_DN132_c0_g1_i5-2.4e+02-OGA1                                      100.0%  70.3%    -----GYL-HSQHQLNYPSP--------KMFTLKK---------------------------TLLIL----FFLGTISLSLC-------     35 F1S24-P69-TRINITY_DN122936_c0_g1_i1-4.3e+02-odorranalectin                         100.0%  70.3%    -----SCL-HSQHQLDYPSP--------KMFTLKK---------------------------SLLLL----FFLGIISLSLC-------     44 F1S19-P60-TRINITY_DN39_c0_g1_i2-7.2e+00-odorranain-P2c                             100.0%  70.3%    -----IGL-HSXFQLNHQSP--------KMFTLKK---------------------------SLLLL----FFLGTINLSLC-------     60 F1S19-P62-TRINITY_DN638_c0_g1_i2-3.9e+00-odorranain-P2e                            100.0%  70.3%    -----YRL-HSQHQLNYLSP--------KMFTLKK---------------------------PLLLL----FFLGTISLSLC-------     58 F1S11-P36-TRINITY_DN79_c1_g3_i1-3.4e+03-odorranain-B6                              100.0%  69.7%    ----------SPHQLNYPSP--------KMFTLQK---------------------------PLLLL----FFLGIVSLSFC-------     45 F1S12-P41-TRINITY_DN10924_c1_g1_i1-2.4e+00-odorranain-C11                           74.2%  69.6%    ----------------------------KMFTMKK---------------------------YLLVL----FFLGIVSLSLC-------     54 F1S7-P14-TRINITY_DN4249_c0_g1_i1-2.1e+03-esculentin-1-OG13                          93.5%  69.0%    --------------LNYPSP--------KMFTLKK---------------------------PLLLI----VLLGIISLALC-------     79 F1S18-P58-TRINITY_DN5345_c0_g1_i2-5.3e+03-odorranain-P1b                           100.0%  68.8%    -----------APTAKSSSP--------KMFTLKK---------------------------SLLLL----FLLGTINLSLC-------     83 F1S9-P29-TRINITY_DN16_c2_g1_i1-5.9e+00-nigrocin-OG34                               100.0%  68.8%    -----------QNQLNHQSP--------QMLSLKK---------------------------SLLHL----FFLGTINLSLC-------     84 F1S9-P24-TRINITY_DN1399_c0_g1_i1-4.6e+01-nigrocin-2GRa                             100.0%  68.4%    -----------KHQLNYPST--------KMFTLKK---------------------------SLFLL----FFLGTINLSLWQDETNA-     36 F1S8-P18-TRINITY_DN0_c1_g1_i22-8.3e+03-esculentin-2-OG10                            96.8%  68.4%    ----SSCL-HSQHQLNY-SP--------KMFTLNK---------------------------SLLLL----FFLGTISLSLC-------     37 F1S12-P39-TRINITY_DN0_c1_g1_i15-2.2e+01-brevinin-2GRb                               96.8%  68.4%    ----SSCL-HSQHQLNY-SP--------KMFTLNK---------------------------SLLLL----FFLGTISLSLC-------     38 F1S12-P39-TRINITY_DN0_c1_g1_i23-8.4e+00-brevinin-2GRb                               96.8%  68.4%    ----SSCL-HSQHQLNY-SP--------KMFTLNK---------------------------SLLLL----FFLGTISLSLC-------     39 F1S22-P65-TRINITY_DN98_c53_g1_i1-3.6e+03-odorranain-T1                              96.8%  68.4%    ----SSCL-HSQHQLNY-SP--------KMFTLNK---------------------------SLLLL----FFLGTISLSLC-------     40 F1S28-P75-TRINITY_DN0_c1_g1_i20-5.5e+03-OGTI                                        96.8%  68.4%    ----SSCL-HSQHQLNY-SP--------KMFTLNK---------------------------SLLLL----FFLGTISLSLC-------     64 F1S16-P55-TRINITY_DN3181_c1_g1_i1-3.5e+02-odorranain-M4                             71.0%  68.2%    ------------------SP--------KMFTLKK---------------------------FLLLL----FFLGIVSS----------     70 F1S12-P42-TRINITY_DN1218_c4_g1_i1-2.4e+00-odorranain-C12                            90.3%  67.9%    ---------------XTTRP--------KMFTMQK---------------------------SLLLL----FFLGAISLSLC-------     85 F1S8-P19-TRINITY_DN2168_c4_g1_i1-6.8e+00-esculentin-2-OG17                         100.0%  67.7%    ------------HQPNYPSP--------KMLTMKK---------------------------CMLVL----FFRGTISLSLC-------     24 F1S9-P27-TRINITY_DN9643_c0_g1_i4-2.5e+00-nigrocin-OG32                             100.0%  67.6%    -----SCL-HSQNQLNHPSP--------KMFTMKK---------------------------SLLLL----FFLGTINLSIC-------     49 F1S24-P69-TRINITY_DN106_c6_g1_i1-2.1e+01-odorranalectin                            100.0%  67.6%    -----CDL-HSQHQLNHPSP--------KMFTMKK---------------------------SLLLL----FFLGIISLSLC-------     59 F1S21-P64-TRINITY_DN638_c6_g1_i1-2.4e+02-odorranain-S1                             100.0%  67.6%    -----SCL-HSQHQLNYPSA--------TMFTLKK---------------------------SLLLL----FFLGAISLSLC-------     81 F1S35-P82-TRINITY_DN360_c0_g1_i1-7.9e+02-odorranain-X4a                            100.0%  67.6%    ------------HQLNYPIP--NYPQSSKMFTLKK---------------------------SLLFL----FFLGIISFSLC-------     47 F1S13-P47-TRINITY_DN1102_c1_g1_i1-1.1e+00-odorranain-F3                            100.0%  66.7%    ------RL-HSQHQLNYPSP--------KMFTMKK---------------------------SLLVL----FFLGIVSLSLC-------     78 F1S15-P51-TRINITY_DN45_c1_g1_i1-3.1e+03-odorranain-L2                               96.8%  66.7%    -------------RDKEMVP--------KMFTMTK---------------------------SLLLL----FFLGTISLSLC-------     67 F1S10-P31-TRINITY_DN7347_c0_g1_i1-4.9e+03-odorranain-A8                             90.3%  65.0%    --KFSSCQ-HSHHQLNYP-----------MFTLKK---------------------------SLLLL----FFLGTISLSLC-------     66 F1S23-P67-TRINITY_DN12170_c0_g1_i1-1.1e+00-odorranain-U2                            64.5%  65.0%    ----------------------------KMFTFKK---------------------------FLLLL----FFLGIASS----------     75 F1S9-P24-TRINITY_DN77_c0_g1_i1-7.4e+01-nigrocin-2GRa                               100.0%  64.9%    -----SCL-HCQHQLNHPSR--------KMFTLKK---------------------------SMLLL----CFLGTISLSLC-------     69 F1S6-P11-TRINITY_DN6490_c1_g1_i1-8.1e+00-brevinin-2E-OG8                            96.8%  63.3%    -------------TNXTTRP--------KMFTMKK---------------------------SVLLL----FFLGTISISLC-------     55 F1S11-P35-TRINITY_DN11239_c0_g1_i2-7.5e+03-odorranain-B1                           100.0%  63.2%    -----SCL-HSQHQLNYPSP--------KMFTLKK---------------------------PLLLL----FFLGIVSLSVCG------     46 F1S13-P46-TRINITY_DN6_c27_g1_i1-4.8e+03-odorranain-F2                              100.0%  62.2%    -----SCL-HSQHQLNYSSP--------KMFTMKK---------------------------SLLVL----FFLGIVSLSLC-------     50 F1S5-P6-TRINITY_DN0_c1_g1_i6-6.1e+02-brevinin-1E-OG3                               100.0%  62.2%    -----SCP-HSHHQLNYPIP--------KMFTLKK---------------------------PLLLI----VLLGIISLSLC-------     51 F1S7-P12-TRINITY_DN0_c1_g1_i18-2.7e+03-esculentin-1-OG5                            100.0%  62.2%    -----SCP-HSHHQLNYPIP--------KMFTLKK---------------------------PLLLI----VLLGIISLSLC-------     52 F1S9-P26-TRINITY_DN0_c1_g1_i3-1.2e+00-nigrocin-2GRc                                100.0%  62.2%    -----SCP-HSHHQLNYPIP--------KMFTLKK---------------------------PLLLI----VLLGIISLSLC-------     53 F1S7-P12-TRINITY_DN81_c0_g1_i1-9.5e+03-esculentin-1-OG5                            100.0%  62.2%    -----SCL-HSQHQLNYPSP--------KMFTLKK---------------------------PLLLI----VLLGIISLSLC-------     86 F1S12-P44-TRINITY_DN2213_c1_g1_i1-5.8e+00-odorranain-C14                            93.5%  62.1%    --------------MNXPSP--------KMFTFRK---------------------------SRVLL----LVLGTISLSLC-------     87 F1S12-P45-TRINITY_DN2213_c1_g1_i2-2.4e+00-odorranain-C15                            93.5%  62.1%    --------------MNXPSP--------KMFTFRK---------------------------SRVLL----LVLGTISLSLC-------     61 F1S3-P3-TRINITY_DN25_c0_g1_i2-5.2e+02-andersonin-S                                  90.3%  61.8%    -----SCL-HSKHQLNYPSP--------KMFTLKK---------------------------FLLLL----FFLGIVSS----------     62 F1S16-P53-TRINITY_DN25_c0_g1_i1-2.1e+03-odorranain-M2                               90.3%  61.8%    -----SCL-HSKHQLNYPSP--------KMFTLKK---------------------------FLLLL----FFLGIVSS----------     63 F1S16-P54-TRINITY_DN25_c0_g1_i3-2.7e+03-odorranain-M3                               90.3%  61.8%    -----SCL-HSKHQLNYPSP--------KMFTLKK---------------------------FLLLL----FFLGIVSS----------     65 F1S33-P80-TRINITY_DN1399_c2_g1_i1-7.7e+00-odorranain-X2a                            90.3%  61.8%    -----SCL-HSKHQLNYPSP--------KMFTLKK---------------------------FLLLL----FFLGIVSS----------     80 F1S18-P59-TRINITY_DN38049_c0_g1_i1-9.8e+01-odorranain-P1i                          100.0%  60.6%    ----------PAPTAKFSTP--------TMFPLKT---------------------------SLLLL----FFLRTINLSLC-------     48 F1S13-P46-TRINITY_DN10285_c0_g1_i1-3.0e+00-odorranain-F2                            96.8%  60.0%    -------------TNXSTRP--------KMFTMKK---------------------------SLLVL----FFLGIVSLSLC-------     71 F1S10-P33-TRINITY_DN25595_c0_g1_i1-3.1e+00-odorranain-A10                          100.0%  59.5%    -----TVA-TRRFIVYYPSP--------KMFTMTK---------------------------SLLLL----FFLGTISLSLC-------     74 F1S7-P15-TRINITY_DN12856_c2_g1_i1-2.7e-01-esculentin-1-OG14                         93.5%  58.6%    --------------RDYSSP--------KMFTLKK---------------------------PLILI----VLLGIISLSLC-------     73 F1S7-P13-TRINITY_DN259_c0_g1_i1-1.7e+02-esculentin-1-OG12                          100.0%  51.4%    -----SCV-HPGRRRNYPRP--------KMFTLKK---------------------------PLLLI----VLLGIISLSQC-------     88 F1S30-P77-TRINITY_DN0_c174_g2_i1-9.7e+03-pleurain-E-OG1                            100.0%  50.0%    ------CL-HSQHQPNYPSP--------KMLSLKT---------------------------SLLLL----FFIGIVSSSPCRG-----     89 F1S28-P75-TRINITY_DN603_c0_g1_i1-4.5e+01-OGTI                                      100.0%  48.8%    -----SCL-HSQHQLNYPSP--------KMFTMKK---------------------------SMLLL----LFVGVIFGSLWEEHRDA-     91 F1S28-P75-TRINITY_DN603_c0_g1_i3-5.1e-01-OGTI                                       93.5%  43.3%    --------------LHYKSD--------KMFAMKK---------------------------SLFVR----LCVGGIQLSLWG------    101 F14-P102-TRINITY_DN17623_c0_g1_i1-3.4e-01-C-X-C_motif_chemokine_8-OG1              100.0%  23.7%    ----------ENHSFSHKLT-------RTLLTMKA---------------------------TLCILAVLAVFLTCFTLSEG-------    102 F14-P103-TRINITY_DN17623_c0_g1_i2-1.2e+01-C-X-C_motif_chemokine_8-OG2              100.0%  23.7%    ----------ENHSFSHKLT-------RTLLTMKA---------------------------TLCILAVLAVFLTCFTLSEG-------    117 F2-P84-TRINITY_DN9272_c0_g2_i1-2.5e+01-pro-FMRFamide-related_neuropeptide_FF-OG1    80.6%  23.3%    ---------------NYKDR-------IRHLTYTA-----------------------MGIVTLLFI----AFLSCART----------    118 F12-P99-TRINITY_DN46746_c0_g1_i2-1.3e+00-mesotocin-neurophysin_MT                  100.0%  20.6%    ---------LSNTVNNLDTS--------NMIAMTY---------------------------SSLAA----SFFCLLALSSA-------    107 F5-P89-TRINITY_DN62991_c0_g1_i1-4.9e+02-amotoxin-OG                                100.0%  18.9%    -----------QHQLSIVVN------KTRDYNMKT---------------------------TLLLA----VIATSLLMFQLTSA----    116 F14-P101-TRINITY_DN2072_c0_g1_i1-6.2e-01-C-X-C_motif_chemokine_14-OG1              100.0%  18.9%    ----------XNXSPYYKXQ-------NRNWDMKP-------------------------LYAAILL----LILAICTLQVE------G     97 F11-P98-TRINITY_DN52727_c0_g1_i1-4.9e-01-galanin                                   100.0%  18.4%    ---------SRDRNCKHTYP--------NQLKMEK------CT-------------------SLLLV----SLILCATISQTFG-----    100 F4-P88-TRINITY_DN9548_c0_g1_i3-1.8e+00-progonadoliberin-2                          100.0%  17.9%    ------PSAWFLXTVSHIYX--------PMACQRH---------------------------LLLLL----LVLFSVSTQLSHG-----     98 F4-P88-TRINITY_DN9548_c0_g1_i2-3.5e-01-progonadoliberin-2                          100.0%  17.9%    ------TFARFLXTVSHIYX--------PMACQRH---------------------------LLLLL----LVLFSVSTQLSHG-----     99 F4-P88-TRINITY_DN9548_c0_g1_i1-6.6e-01-progonadoliberin-2                          100.0%  17.9%    ------IXQRFLXTVSHIYX--------PMACQRH---------------------------LLLLL----LVLFSVSTQLSHG-----    104 F3-P85-TRINITY_DN3001_c0_g1_i6-3.0e-01-ranatachykinin-A                             96.8%  14.7%    -------------SAGPVQR--------VLWRKRE---------------------------SMKIL----VAFAVIMLVSAQVFA---    105 F3-P85-TRINITY_DN3001_c0_g1_i4-3.6e+00-ranatachykinin-A                             96.8%  14.7%    -------------SAGPVQR--------VLWRKRE---------------------------SMKIL----VAFAVIMLVSAQVFA---    108 F8-P92-TRINITY_DN593_c2_g1_i4-3.3e+00-odorranain-BLP-4                             100.0%  14.3%    ----------ASLRLSHLYR-------AHSTDMTA-----------------------VPAIRILPI----GFLAILLLFSVISRS---    109 F8-P92-TRINITY_DN593_c2_g1_i3-4.7e+02-odorranain-BLP-4                             100.0%  14.3%    ----------ASLRLSHLYR-------AHSTDMTA-----------------------VPAIRILPI----GFLAILLLFSVISRS---     92 F6-P90-TRINITY_DN15119_c0_g1_i1-1.9e+02-peptide_YY-like                             93.5%  14.0%    EASLPSCTPPSFQQIMVTSL--------KLWPMMVA-------------------------ITICVL----ICLGTIVEG---------     93 F14-P106-TRINITY_DN5647_c0_g1_i2-5.1e-01-C-X-C_motif_chemokine_11-OG1               93.5%  13.9%    ------CVSPAMKQVRAPA---------VIMDFKC---------------------------AVIVC----ILLSAILVQG--------     94 F14-P107-TRINITY_DN5647_c0_g1_i1-6.3e-01-C-X-C_motif_chemokine_11-OG2               93.5%  13.9%    ------CVSPAMKQVRAPA---------VIMDFKC---------------------------AVIVC----ILLSAILVQG--------     95 F14-P104-TRINITY_DN36485_c0_g1_i1-4.4e+00-C-X-C_motif_chemokine_10-OG1              77.4%  13.5%    -----------------VKPAFLLVQVNLFYTMAR-----------------------------ILI----AVLGTLLILQSVQG----     96 F14-P105-TRINITY_DN36485_c0_g1_i3-2.0e-01-C-X-C_motif_chemokine_10-OG2              77.4%  13.5%    -----------------VKPAFLLVQVNLFYTMAR-----------------------------ILI----AVLGTLLILQSVQG----    112 F8-P94-TRINITY_DN9594_c0_g1_i2-2.0e+00-odorranain-BLP-7                             77.4%  12.9%    -------------------R-------AHSTDMTA-----------------------VPGIRILPV----GFLGLLLLFSVSS-----    113 F8-P94-TRINITY_DN3618_c0_g1_i1-3.4e+01-odorranain-BLP-7                            100.0%  12.5%    ------------LRLSDLYR-------ARSTDMTA-----------------------VPGIRILPV----GFLGILLLFSVISRS---    110 F8-P94-TRINITY_DN593_c2_g1_i2-4.6e+01-odorranain-BLP-7                             100.0%  11.9%    ----------ASLRLSHLYR-------AHSTDMTA-----------------------VPGIRILPV----GFLGILLLFSVISRS---    111 F8-P94-TRINITY_DN593_c2_g1_i1-4.5e+03-odorranain-BLP-7                             100.0%  11.9%    ----------ASLRLSHLYR-------AHSTDMTA-----------------------VPGIRILPV----GFLGILLLFSVISRS---    114 F8-P94-TRINITY_DN3333_c1_g1_i3-2.2e+01-odorranain-BLP-7                             87.1%  11.1%    ----------------HLYR-------AHSTDMTA-----------------------GPGITILAV----GFLGILLLFSVISRA---    106 F9-P95-TRINITY_DN24491_c0_g1_i1-1.0e+02-calcitonin-like_peptide_1-OG1              100.0%  10.8%    -----------ELSLDYPSR-------NRNRSIMK--------------------------LAYIIV----LLISCLTFLGSTMA----    115 F13-P100-TRINITY_DN98392_c0_g1_i1-3.7e-01-insulin-like_growth_factor_II_isoform_X2  96.8%  10.0%    ------------AVRPHPSP-G----KHSHWNMEQLRCHSSCSSPTPPCRRTQMPGVPVPRHALLLL----YTFIAYTAES--------    103 F7-P91-TRINITY_DN110119_c0_g1_i1-4.5e-01-7B2_granin_protein                        100.0%   9.8%    -----------NRTGXMELK------NILXVTMKH---------------------------NAIIS----LVCPLVVLLVCGLNPSFG    119 F10-P96-TRINITY_DN988_c0_g1_i1-3.4e+00-OG-CATH1                                     83.9%   7.5%    ----------IIRAITITFT-------IHRRKMKIWQ----CV----------------------------LWLCAVTLEVVHS-----    120 F10-P96-TRINITY_DN988_c0_g1_i2-2.8e+00-OG-CATH1                                     83.9%   7.5%    ----------IIRAITITFT-------IHRRKMKIWQ----CV----------------------------LWLCAVTLEVVHS-----    121 F10-P97-TRINITY_DN988_c0_g1_i4-3.8e+00-OG-CATH2                                     83.9%   7.5%    ----------IIRAITITFT-------IHRRKMKIWQ----CV----------------------------LWLCAVTLEVVHS-----        clustal                                                                                                                                                                                              consensus/75%                                                                                       .............................M.T..K.............................L.......FLG...L.......... ``` |

MView 1.67, Copyright © 1997-2020 Nigel P. Brown
