# Supplementary material for: Integrative transcriptomics and peptidomics approach reveals unexpectedly diverse endogenous secretory peptides in Odorrana grahami frog skin
Source: BMC Biol. 2025 Nov 28;23:354. doi: 10.1186/s12915-025-02463-w (PMC12664280; doi:10.1186/s12915-025-02463-w)
Supplement: Supplementary file 5 — Additional file 5. Alignments of ESP sequences identified in this study across different regions. [file 12915_2025_2463_MOESM5_ESM.zip › Additional file 5/FSAP family - acidic propiece region.html]

MView


|  |
| --- |
| ``` Reference sequence (1): F1S1-P1-TRINITY_DN175_c1_g1_i1-9.3e+02-andersonin-Q Identities normalised by aligned length. Colored by: consensus group/60% ``` |
| ```                                                                   cov    pid  1 [        .         .         .         .         :         .         .         .         .         1         .         .         .         .         :         .    ] 165  1 F1S1-P1-TRINITY_DN175_c1_g1_i1-9.3e+02-andersonin-Q         100.0% 100.0%    ---------gaggaagagagaaatgccgaagaagaaag---------------------aagagatgatcaagataaaatggatgttgaaatggaaaaacga---------------------------------------------------------------      2 F1S14-P50-TRINITY_DN603_c2_g1_i1-5.0e+02-odorranain-G1      100.0%  91.7%    ---------gaggaagagagaaatgcagaagaagaaag---------------------aagagatgatccagatgaaatgaatgctgaagtggaaaaacga---------------------------------------------------------------      4 F1S5-P6-TRINITY_DN0_c1_g1_i24-1.9e+03-brevinin-1E-OG3       100.0%  80.0%    ---------gagcaagagagagatgctgatgaggaaga------------------aagaagagatgatccagaagaaagggatgttgaagtggaaaaacga---------------------------------------------------------------      5 F1S5-P6-TRINITY_DN0_c1_g1_i6-6.1e+02-brevinin-1E-OG3        100.0%  80.0%    ---------gagcaagagagagatgctgatgaggaaga------------------aagaagagatgatccagaagaaagggatgttgaagtggaaaaacga---------------------------------------------------------------      6 F1S36-P83-TRINITY_DN14764_c0_g1_i2-4.9e+02-odorranain-X5a   100.0%  80.0%    ---------gagcaagagagagatgctgatgaggaaga------------------aagaagagatgatctagaagaaagggatgttgaagtggaaaaacga---------------------------------------------------------------      3 F1S5-P5-TRINITY_DN23413_c1_g1_i1-1.3e+00-gaegurin-6-OG1     100.0%  77.3%    ---------gagcaagcgagagatgctgatgaggaaga------------------aagaagcgatgatccagacgaaagggatgttgaagtggaaaaacga---------------------------------------------------------------      7 F1S5-P7-TRINITY_DN23816_c1_g1_i1-4.5e+02-brevinin-1E-OG9    100.0%  77.3%    ---------gagcaagagagagattctgatgaggaaga------------------aagaagagatgatccagaagaaagggatgttgaagttgaaaaacga---------------------------------------------------------------      8 F1S5-P8-TRINITY_DN33233_c1_g1_i1-1.1e+02-brevinin-1E-OG10   100.0%  77.3%    ---------gagcaagagacagatgcggatgaggaaga------------------aagaagagatgagccagaagaaagggatgttgaagtggaaaaacga---------------------------------------------------------------     11 F1S34-P81-TRINITY_DN17503_c0_g1_i1-1.2e+00-odorranain-X3a    87.5%  72.0%    ---------gagctagagagaaatgccgatgaggaaga------------------aagaagagat---------gaaagagatgttgaaatggaaaaacga---------------------------------------------------------------      9 F1S18-P58-TRINITY_DN5345_c0_g1_i2-5.3e+03-odorranain-P1b     87.5%  69.2%    ------caggaggtagagagaaatgcagatgaggaaga------------------aagaagagat---------gaaagggatgttgaagtggaaaaacga---------------------------------------------------------------     10 F1S18-P59-TRINITY_DN38049_c0_g1_i1-9.8e+01-odorranain-P1i    87.5%  66.7%    ------cagcaggaagagagaaatgcagatgaggaaga------------------aagaagagat---------gaaagagatgttgaagtggaagaacga---------------------------------------------------------------     63 F1S15-P51-TRINITY_DN45_c1_g1_i1-3.1e+03-odorranain-L2        58.3%  64.7%    ---------gagcaagagagaaatgccgatgaagaagg------------------aaatgaagaa------aataga---------------------------------------------------------------------------------------     19 F1S9-P26-TRINITY_DN0_c1_g1_i17-1.5e+04-nigrocin-2GRc         87.5%  58.0%    ---------gagcaagagagaaatgccgatgaggaaga------------------aagaagagat---------gaagaagttgctaaaatggaagagataaaacgc---------------------------------------------------------     20 F1S9-P26-TRINITY_DN0_c1_g1_i3-1.2e+00-nigrocin-2GRc          87.5%  58.0%    ---------gagcaagagagaaatgccgatgaggaaga------------------aagaagagat---------gaagaagttgctaaaatggaagagataaaacgc---------------------------------------------------------     23 F1S9-P29-TRINITY_DN16_c2_g1_i1-5.9e+00-nigrocin-OG34         87.5%  58.0%    ---------caggatgagacaaatgccgaagaagaaag---------------------aagagat---------gaagcagctgcaaaaatcgaagagataccccgc------------------------------------------------------cgg     30 F1S26-P71-TRINITY_DN132_c0_g1_i3-1.8e+02-ishikawain-7-EV1    91.7%  57.7%    ---------gaggaagagagagatgccgacgaagaaga------------------aaatgaagta------aatgaaggggaagctaaagtggaagtaaaaaga------------------------------------------------------------     12 F1S9-P24-TRINITY_DN77_c0_g1_i1-7.4e+01-nigrocin-2GRa         87.5%  56.8%    ---------gagcaagagagaaatgccgaggaggaaga------------------aagaagagag---------gaagaagttgctaaaatggaagagataacacgc---------------------------------------------------------     90 F3-P86-TRINITY_DN6_c0_g1_i12-1.0e+03-tachykinin_OG1          91.7%  56.4%    ------gaggaagagaagagagatgccaatgaagaagc------------------atatgaagaa------aatgtaagagatgccaacatggaaaaaaga---------------------------------------------------------------     91 F3-P87-TRINITY_DN6_c0_g1_i6-8.0e+02-ranamargarin             91.7%  56.4%    ------gaggaagagaagagagatgccaatgaagaagc------------------atatgaagaa------aatgtaagagatgccaacatggaaaaaaga---------------------------------------------------------------     31 F1S26-P71-TRINITY_DN132_c0_g1_i1-3.5e+02-ishikawain-7-EV1    87.5%  56.4%    ---------gaggaagagagagatgccgacgaaga---------------------aaatgaagta------aatgaaggggaagctaaagtggaagtaaaaaga------------------------------------------------------------     55 F1S21-P64-TRINITY_DN638_c6_g1_i1-2.4e+02-odorranain-S1       87.5%  55.1%    ---------gaacaagagagagatgccgatgaagaaga------------------------ggaa------aatggaggggaagctaaagtggaagaaataaaaaga---------------------------------------------------------     64 F1S22-P65-TRINITY_DN98_c53_g1_i1-3.6e+03-odorranain-T1       91.7%  54.3%    ---------gagcaagaaagagatgccgatgaagaaag------------------caatgaagaa------aatggagtagaagctaaagttaaagagctaaaaagg---------------------------------------------------------     14 F1S9-P26-TRINITY_DN0_c1_g1_i2-2.2e+04-nigrocin-2GRc          83.3%  54.3%    ---------caggatgagacaaatgcc---gaagaaga------------------aagaagagat---------gaagaagttgctaaaatggaagagataaaacgc---------------------------------------------------------     16 F1S9-P25-TRINITY_DN49_c0_g1_i1-5.8e+03-nigrocin-2GRb         83.3%  54.3%    ---------caggatgagacaaatgcc---gaagaaga------------------aagaagagat---------gaagaagttgctaaaatggaagagataaaacgt---------------------------------------------------------     15 F1S9-P30-TRINITY_DN11504_c0_g1_i1-4.8e+00-nigrocin-OG35      83.3%  53.1%    ---------cagtatgagacaaatgcc---gaagaaga------------------aagaagagat---------gaagaagttgctaaaatggaagagataaaacgc---------------------------------------------------------     17 F1S9-P28-TRINITY_DN4414_c6_g1_i1-5.3e+00-nigrocin-OG33       83.3%  53.1%    ---------caggatgatacaaatgcc---gaagaaga------------------aagaagagat---------gaagaagttgctaaaatggaagagataaaacgt---------------------------------------------------------     18 F1S9-P27-TRINITY_DN9643_c0_g1_i4-2.5e+00-nigrocin-OG32       83.3%  53.1%    ---------caggatgagacaaatgcc---gaagaaga------------------aagaagagat---------gaagaagtggctaaaatggaagagataaaacgc---------------------------------------------------------     21 F1S19-P60-TRINITY_DN39_c0_g1_i2-7.2e+00-odorranain-P2c       83.3%  53.1%    ---------caggaggagacaaatgcc---gaagaaga------------------aagaagagat---------gaagaagttgctaaaaaggaagagataaaacgc---------------------------------------------------------     57 F1S17-P56-TRINITY_DN122946_c2_g1_i1-1.8e+03-odorranain-O1    79.2%  52.6%    ---------gaggaagagagaggtgccgatgaagaag------------------------------------atggaggggaagctaaactggaagacataaaaaga---------------------------------------------------------     62 F1S10-P31-TRINITY_DN7347_c0_g1_i1-4.9e+03-odorranain-A8      91.7%  52.4%    ---------gaggaagagagagatgctgatgaagaagg------------------aagtgaagaa------aatggatcggaagctaaagttgaagacataaaaagaagg------------------------------------------------------     59 F1S23-P68-TRINITY_DN128039_c0_g1_i1-2.6e+03-odorranain-U3    91.7%  51.9%    ---------gaggaagagagagttgctgatgaagaagg------------------aaatgaagaa------aatggaggggaagccaaattggaagtcgtaaaaaga---------------------------------------------------------     22 F1S19-P61-TRINITY_DN4628_c1_g1_i1-1.2e+00-odorranain-P2d     87.5%  51.9%    ---------gagcaagatagagattcggatgaggaaga------------------aagaagagat---------gaagaagttgctacaatggaagagataaaacgc---------------------------------------------------------     74 F1S32-P79-TRINITY_DN13210_c0_g1_i1-9.0e+00-odorranain-X1a    83.3%  51.3%    ---------aaggaagagagagatgcctatgaagaaga------------------------ggaa------aatggaggggaagttaaa---gaggatgtaaaaaga---------------------------------------------------------     38 F1S6-P9-TRINITY_DN0_c1_g1_i4-9.2e-01-brevinin-2GRa           79.2%  51.3%    ---------gaggaagagagagatgctgatgaagatg------------------------------------atggagtggaagtgacagaggaagaagtaaaaaga---------------------------------------------------------     39 F1S6-P9-TRINITY_DN0_c1_g1_i14-1.3e+04-brevinin-2GRa          79.2%  51.3%    ---------gaggaagagagagatgctgatgaagatg------------------------------------atggagtggaagtgacagaggaagaagtaaaaaga---------------------------------------------------------     40 F1S12-P39-TRINITY_DN0_c1_g1_i23-8.4e+00-brevinin-2GRb        79.2%  51.3%    ---------gaggaagagagagatgctgatgaagatg------------------------------------atggagtggaagtgacagaggaagaagtaaaaaga---------------------------------------------------------     41 F1S12-P39-TRINITY_DN0_c1_g1_i11-1.7e+00-brevinin-2GRb        79.2%  51.3%    ---------gaggaagagagagatgctgatgaagatg------------------------------------atggagtggaagtgacagaggaagaagtaaaaaga---------------------------------------------------------     51 F1S11-P36-TRINITY_DN79_c1_g3_i1-3.4e+03-odorranain-B6        79.2%  51.3%    ---------gagcaagagagagatgccgatgaagaag------------------------------------atggagggggagttacaggggaagaagtaaaaaga---------------------------------------------------------     53 F1S11-P37-TRINITY_DN56_c1_g1_i1-5.0e+01-odorranain-B7        79.2%  51.3%    ---------gatcaagagagagatgccgatgaagaag------------------------------------atggaggggcagttacaggggaagaaggaaaaaga---------------------------------------------------------     54 F1S11-P38-TRINITY_DN1399_c4_g1_i1-2.5e+01-odorranain-B8      79.2%  51.3%    ---------gatcaagagagagatgccgatgaagaag------------------------------------atggaggggacgttacaggggaagaagtaaaacga---------------------------------------------------------     58 F1S17-P57-TRINITY_DN38944_c0_g1_i1-6.3e-01-odorranain-O4     79.2%  51.3%    ---------gaggaagagagaggcgccgatgaagaag------------------------------------atggaggggaagctaaactggaagacataaaaaga---------------------------------------------------------     50 F1S11-P35-TRINITY_DN11239_c0_g1_i2-7.5e+03-odorranain-B1     75.0%  50.7%    ------------caagagagagatgccgatgaagaag------------------------------------atggaggggaagttacaggggaagaagtaaaaaga---------------------------------------------------------     24 F1S2-P2-TRINITY_DN142_c0_g1_i5-5.0e+01-andersonin-R          81.9%  50.6%    ---------gagcaagagagagatgccgatgaagaagaa---------------------------------aacggagtggaagataaagtggaggacataaaaaga---------------------------------------------------------     65 F1S24-P69-TRINITY_DN122936_c0_g1_i1-4.3e+02-odorranalectin   81.9%  50.6%    ---------gaggaagagagagatgctgatgaagaagaa---------------------------------aatgaagtggaggctaaacttaaagacatcaaacga---------------------------------------------------------     66 F1S24-P69-TRINITY_DN106_c6_g1_i1-2.1e+01-odorranalectin      81.9%  49.4%    ---------gaggaagagagagatgctgatgaagaagaa---------------------------------aacgaagtggaggctaaacttaaagacatcaaacga---------------------------------------------------------     73 F1S30-P77-TRINITY_DN0_c174_g2_i1-9.7e+03-pleurain-E-OG1      51.4%  49.0%    ---------------------------------------------------------aaaagagaa------gatggagaggaagctgaaatagaagacataaaaaga---------------------------------------------------------     42 F1S6-P11-TRINITY_DN6490_c1_g1_i1-8.1e+00-brevinin-2E-OG8     79.2%  48.7%    ---------gaagaagagagagatgctgatgaagatt------------------------------------atggagtggaagtgacagaagaagaagtaaaaaga---------------------------------------------------------     56 F1S29-P76-TRINITY_DN8472_c0_g1_i1-2.5e+03-palustrin-OG2      70.8%  48.7%    ---------gagcaagagagagatgccgatgaag---------------------------------------atgaaggggaagctcta---gaagaagtaaaaaga---------------------------------------------------------     13 F1S9-P24-TRINITY_DN1399_c0_g1_i1-4.6e+01-nigrocin-2GRa       58.3%  48.3%    ------------------------------gaagaaga------------------aagaagagat---------gaagaagttgctaaaatggaagagataaaacgc---------------------------------------------------------     52 F1S25-P70-TRINITY_DN1048_c0_g1_i1-1.9e+02-odorranaopin       81.9%  48.1%    ---------gagcaagagagagatgctgatgaagaggaa---------------------------------aatggaggggaagttacagagaaagaagtaaaaaga---------------------------------------------------------     61 F1S35-P82-TRINITY_DN360_c0_g1_i1-7.9e+02-odorranain-X4a      81.9%  48.1%    ---------gaggaagagagaggagccgaagaagaggaa---------------------------------aatggtggggaagttacagagaaagaagtaaaaaga---------------------------------------------------------     76 F1S8-P16-TRINITY_DN96_c0_g1_i2-2.3e+01-esculentin-2-RA1      79.2%  47.4%    ---------gagcaagagagatctgccgatgaagacg------------------------------------atggaggggaaatgaaaggggaagaagcgaaaaga---------------------------------------------------------     77 F1S8-P22-TRINITY_DN96_c0_g1_i1-6.4e+00-esculentin-2-OG20     79.2%  47.4%    ---------gagcaagagagatctgccgatgaagacg------------------------------------atggaggggaaatgaaaggggaagaagcgaaaaga---------------------------------------------------------     78 F1S8-P17-TRINITY_DN96_c0_g2_i2-1.4e+02-esculentin-2-OG8      79.2%  47.4%    ---------gagcaagagagagctgccgatgaagacg------------------------------------atggaggggaaatgaaaggggaagaagtgaaaaga---------------------------------------------------------     79 F1S8-P23-TRINITY_DN96_c0_g2_i1-3.5e-01-esculentin-2-OG21     79.2%  47.4%    ---------gagcaagagagagctgccgatgaagacg------------------------------------atggaggggaaatgaaaggggaagaagtgaaaaga---------------------------------------------------------     43 F1S12-P39-TRINITY_DN0_c1_g1_i15-2.2e+01-brevinin-2GRb        75.0%  47.4%    ---------gaggaagagagagatgccgatgaagagga---------------------------------------aggggaaatgacagaggaagaagtaaaaaga---------------------------------------------------------     44 F1S12-P39-TRINITY_DN0_c1_g1_i10-8.0e+03-brevinin-2GRb        75.0%  47.4%    ---------gaggaagagagagatgccgatgaagagga---------------------------------------aggggaaatgacagaggaagaagtaaaaaga---------------------------------------------------------     45 F1S12-P40-TRINITY_DN45_c27_g1_i1-7.5e+02-odorranain-C7       75.0%  47.4%    ---------gaggaagagagagatgccgatgaagagga---------------------------------------aggggaaatgacagaggaagaagtaaaaaga---------------------------------------------------------     25 F1S4-P4-TRINITY_DN836_c0_g1_i2-1.3e+01-andersonin-X-OG1      91.7%  46.5%    ---------gaggaagagagggatgcagatgaagaaagaagagatgatcccgctgaatataaggac------aatggaggggaagctgaagtgaaagaaacaaaaaga---------------------------------------------------------     27 F1S28-P75-TRINITY_DN0_c1_g1_i20-5.5e+03-OGTI                 91.7%  46.5%    ---------gaggaagagagagatgccaatgaagaaagaagagatgatccagatgaaaatgaagca------aatgagggggaagctaaagtggaagaaataaaaaga---------------------------------------------------------     46 F1S12-P42-TRINITY_DN1218_c4_g1_i1-2.4e+00-odorranain-C12     75.0%  46.2%    ---------gaggaagagggagatgccgatgaagagga---------------------------------------aggggaaatgacagaggaagaagtaaaaaga---------------------------------------------------------     47 F1S12-P43-TRINITY_DN2658_c0_g2_i1-3.3e-01-odorranain-C13     75.0%  46.2%    ---------gaggaagagagagatgccgatgaagagga---------------------------------------cggggaaatgacagaggaagaagtaaaaaga---------------------------------------------------------     83 F1S13-P46-TRINITY_DN6_c27_g1_i1-4.8e+03-odorranain-F2        75.0%  46.2%    ---------caggaagagagatctgccgatgacgagga---------------------------------------aggggaagttatagaggaagaagtaaaaaga---------------------------------------------------------     85 F1S19-P62-TRINITY_DN638_c0_g1_i2-3.9e+00-odorranain-P2e      75.0%  46.2%    ---------gaggaagagagatctgccgatgacgacga---------------------------------------aggggaagttatggaggaagaagtaagaaga---------------------------------------------------------     75 F1S26-P72-TRINITY_DN132_c0_g1_i5-2.4e+02-OGA1                87.5%  45.7%    ---------gaggaagagagagatgccgacgaagaaga------------------aaatgaagta------agcggatatgcagctaatgtg---aatataaaaaga---------------------------------------------------------     67 F1S3-P3-TRINITY_DN25_c0_g1_i2-5.2e+02-andersonin-S           91.7%  45.6%    tctccctgttttagaaagagagatgccgatgaagaagg------------------aaatgaagaa------aatggaggggaagctaaaatggaagacataaaaaga---------------------------------------------------------     68 F1S16-P54-TRINITY_DN25_c0_g1_i3-2.7e+03-odorranain-M3        91.7%  45.6%    tctccctgttttagaaagagagatgccgatgaagaagg------------------aaatgaagaa------aatggaggggaagctaaaatggaagacataaaaaga---------------------------------------------------------     69 F1S33-P80-TRINITY_DN1399_c2_g1_i1-7.7e+00-odorranain-X2a     91.7%  45.6%    tctccctgttttagaaagagagatgccgatgaagaagg------------------aaatgaagaa------aatggaggggaagctaaaatggaagacataaaaaga---------------------------------------------------------     70 F1S16-P53-TRINITY_DN25_c0_g1_i1-2.1e+03-odorranain-M2        91.7%  45.6%    tctccctgtcttagaaagagagatgccgatgaagaagg------------------aaatgaagaa------aatggaggggaagctaaaatggaagacataaaaaga---------------------------------------------------------     71 F1S16-P55-TRINITY_DN3181_c1_g1_i1-3.5e+02-odorranain-M4      91.7%  45.6%    tcgccctgttttagaaagagagatgccgaggaagaagg------------------aaatgaagaa------aatggaggggaagctaaaatggaagacataaaaaga---------------------------------------------------------     82 F1S12-P41-TRINITY_DN10924_c1_g1_i1-2.4e+00-odorranain-C11    75.0%  44.9%    ---------caggaagagagatctgacgatgacgagga---------------------------------------aggggaagttatagaggaagaagtaaaaaga---------------------------------------------------------     84 F1S13-P46-TRINITY_DN10285_c0_g1_i1-3.0e+00-odorranain-F2     75.0%  44.9%    ---------caggaagagagatctgccgatgacgagga---------------------------------------aggggaagatatagaggaagaagtaaaaaga---------------------------------------------------------     88 F1S10-P33-TRINITY_DN25595_c0_g1_i1-3.1e+00-odorranain-A10    75.0%  43.7%    ---------gagcaagagagagatgctgatgaagaaga------------------aggaagtgaa------------------gatggagcggaagacataaaattgaac---------------------------------------------------agg     86 F1S13-P47-TRINITY_DN1102_c1_g1_i1-1.1e+00-odorranain-F3      75.0%  43.6%    ---------caggaagagagatctgccgatgacgcgga---------------------------------------aggggaagttatagaggaagacgtaacacga---------------------------------------------------------     87 F1S10-P32-TRINITY_DN25_c1_g1_i1-7.6e+03-odorranain-A9        75.0%  42.5%    ---------gagcaagagagagatgctgatgaagaaga------------------aggaagtgaa------------------aatggagcggaagacataaaattaaac---------------------------------------------------agg     26 F1S20-P63-TRINITY_DN132_c0_g1_i4-8.5e+02-odorranain-Q1       62.5%  42.5%    ---------gaggaagagagggatgcagatgaagaaagaagagatga---------------------------------------tgaagtggaagaaacaaaaaga---------------------------------------------------------     80 F1S8-P18-TRINITY_DN0_c1_g1_i22-8.3e+03-esculentin-2-OG10     66.7%  42.0%    ---------caggaagagagagctgccgatgaagaaga------------------taatggag------------------------aagttgaagaagtgaaaaga---------------------------------------------------------     48 F1S12-P44-TRINITY_DN2213_c1_g1_i1-5.8e+00-odorranain-C14     75.0%  41.4%    ---------gaggaagagagagatgcagatgaagagga---------------------------------------aggggaaatgacagaggaagaagtaaaaagaggtgtcc------------------------------------------------gg     49 F1S12-P45-TRINITY_DN2213_c1_g1_i2-2.4e+00-odorranain-C15     75.0%  41.4%    ---------gaggaagagagagatgcagatgaagagga---------------------------------------aggggaaatgacagaggaagaagtaaaaagaggtgtcc------------------------------------------------gg     81 F1S8-P19-TRINITY_DN2168_c4_g1_i1-6.8e+00-esculentin-2-OG17   66.7%  40.7%    ---------caggaagagagagcggccgatgaagaaga------------------taatggag------------------------aagttgaagaagtgaaaaga---------------------------------------------------------     29 F1S28-P75-TRINITY_DN603_c0_g1_i3-5.1e-01-OGTI                87.5%  40.6%    ------------gaagagggaggtagcaatgaagaaagaagagatggtccagatgaaaatgaagca------aatgagggggaagctaaagtggaagaaataaaaaga---------------------------------------------------------     89 F1S10-P34-TRINITY_DN6115_c1_g1_i1-2.5e+03-odorranain-A11     75.0%  39.1%    ---------gcgccagagagagctgctgatgaagaaga------------------aggaagtgaa------------------aatggagcggaagacataaaattaaac---------------------------------------------------agg     72 F1S23-P67-TRINITY_DN12170_c0_g1_i1-1.1e+00-odorranain-U2     91.7%  36.3%    tctccatgttttagaaagagagatgacgatgaagcagg------------------aaatgaagaa------aatggaggggaagccaaattggaagtagtaaaaagaggatgctcaaga---------------------------------------------     28 F1S28-P75-TRINITY_DN603_c0_g1_i1-4.5e+01-OGTI                66.7%  33.3%    ---------------------------aatgagggaagaagagatgatccagatgaaaatgaagca------aatgagggggaagctaaagtggaagaaataaaaaga---------------------------------------------------------     33 F1S7-P12-TRINITY_DN0_c1_g1_i16-1.0e+00-esculentin-1-OG5      58.3%  33.3%    ---------gagcaagagagagctgccgatgaagatga---------------------------------------------------gggaaacgaaataaaaaga---------------------------------------------------------     34 F1S7-P12-TRINITY_DN0_c1_g1_i18-2.7e+03-esculentin-1-OG5      58.3%  33.3%    ---------gagcaagagagagctgccgatgaagatga---------------------------------------------------gggaaacgaaataaaaaga---------------------------------------------------------     35 F1S7-P14-TRINITY_DN4249_c0_g1_i1-2.1e+03-esculentin-1-OG13   58.3%  33.3%    ---------gagcaagagagagctgccgatgaagatga---------------------------------------------------gggaaacgaaataaaaaga---------------------------------------------------------     32 F1S7-P12-TRINITY_DN81_c0_g1_i1-9.5e+03-esculentin-1-OG5      58.3%  32.1%    ---------gagcaagagagagctgccgatgaagatga---------------------------------------------------gggaagcgaaataaaaaga---------------------------------------------------------     36 F1S7-P15-TRINITY_DN12856_c2_g1_i1-2.7e-01-esculentin-1-OG14  58.3%  32.1%    ---------gagcaagagagagctgccgacgaagatga---------------------------------------------------gggaagcgaaataaaaaga---------------------------------------------------------     37 F1S7-P13-TRINITY_DN259_c0_g1_i1-1.7e+02-esculentin-1-OG12    58.3%  32.1%    ---------gagcaagagagagctgccgatgaagatgc---------------------------------------------------gggaagcgaaataaaaaga---------------------------------------------------------     60 F1S27-P74-TRINITY_DN139_c0_g1_i1-3.1e+02-OGC-RA3             81.9%  26.5%    ---------gaggaacagagagaagctgatgaagaggaa---------------------------------aatggaggggaagttacagaaaaagaagtaaaaagaatcgtaccaaattgcaactataaattttcaggtgcgaattgtttggaaaaagaacga        clustal                                                                                                                                                                                                                                                   consensus/75%                                                                ..........AG.AAGAGAGA..TGC.GATGAAGA.G........................................A...G.......A..GGAAGA..TAAAA.G.......................................................... ``` |

MView 1.67, Copyright © 1997-2020 Nigel P. Brown
