# Supplementary material for: Integrative transcriptomics and peptidomics approach reveals unexpectedly diverse endogenous secretory peptides in Odorrana grahami frog skin
Source: BMC Biol. 2025 Nov 28;23:354. doi: 10.1186/s12915-025-02463-w (PMC12664280; doi:10.1186/s12915-025-02463-w)
Supplement: Supplementary file 5 — Additional file 5. Alignments of ESP sequences identified in this study across different regions. [file 12915_2025_2463_MOESM5_ESM.zip › Additional file 5/FSAP family - mature peptide region.html]

MView


|  |
| --- |
| ``` Reference sequence (1): F1S1-P1-TRINITY_DN175_c1_g1_i1-9.3e+02-andersonin-Q Identities normalised by aligned length. Colored by: consensus group/60% ``` |
| ```                                                                   cov    pid  1 [        .         .         .         .         :         .         .         .         .         1         .         .         .         .         :         .         .         .         .         2         .] 211  1 F1S1-P1-TRINITY_DN175_c1_g1_i1-9.3e+02-andersonin-Q         100.0% 100.0%    ----------------------------------------------------------------------------------------------------------------------tttttgccagcagctatttgtttagttatcaaaacttgt------------------------------------------------------     11 F1S14-P50-TRINITY_DN603_c2_g1_i1-5.0e+02-odorranain-G1       89.7%  51.2%    ------------------------------------------------------------------------------------------------------------ttta----------tgccaatacttagttgttcaaggtttaaaagatgt------------------------------------------------------     69 F1S23-P67-TRINITY_DN12170_c0_g1_i1-1.1e+00-odorranain-U2     66.7%  35.1%    -----------------------------------------------------------------------------------------------------------tggattattg--gactccatggacaaatttgtagagat------------------------------------------------------------------     70 F1S23-P68-TRINITY_DN128039_c0_g1_i1-2.6e+03-odorranain-U3    66.7%  28.6%    g-----------------------------------------------------------------------------------------------gatgctcaagatgtattattg--gaatccatggacaaatttgtatagat------------------------------------------------------------------      3 F1S5-P6-TRINITY_DN0_c1_g1_i24-1.9e+03-brevinin-1E-OG3        89.7%  27.6%    ---------------------------------------------------------------------------tttttaccacttcttgcaggtctggctgctaatttct----------tgccgaaattattttgtaaaataaccaaaaaatgt------------------------------------------------------      4 F1S5-P6-TRINITY_DN0_c1_g1_i6-6.1e+02-brevinin-1E-OG3         89.7%  27.6%    ---------------------------------------------------------------------------tttttaccacttcttgcaggtctggctgctaatttct----------tgccgaaattattttgtaaaataaccaaaaaatgt------------------------------------------------------      7 F1S36-P83-TRINITY_DN14764_c0_g1_i2-4.9e+02-odorranain-X5a    84.6%  27.3%    ---------------------------------------------------------------------------ttttttccacttattgcagacctcctc--------------------ctgatgaagcgatccagtta--cagcgaaacatgt------------------------------------------------------      5 F1S5-P7-TRINITY_DN23816_c1_g1_i1-4.5e+02-brevinin-1E-OG9     89.7%  26.3%    ---------------------------------------------------------------------------tttttaccacttctttcaggtctggctgctaatttct----------tgcagaaattattttgtaaaataaccaaaaaatgt------------------------------------------------------      9 F1S18-P59-TRINITY_DN38049_c0_g1_i1-9.8e+01-odorranain-P1i    89.7%  26.3%    ---------------------------------------------------------------------------gttataccatttgtggcaagtgtggctggcgaaatga----------tgcagcacgtgtattgtgcagcttccaaaacatgt------------------------------------------------------      6 F1S5-P8-TRINITY_DN33233_c1_g1_i1-1.1e+02-brevinin-1E-OG10    89.7%  25.0%    ---------------------------------------------------------------------------tttttaccacttcttgaaggtctggctgctaatttct----------tgccgaaaatattttgtaaaataaccacaaaacgt------------------------------------------------------      8 F1S18-P58-TRINITY_DN5345_c0_g1_i2-5.3e+03-odorranain-P1b     89.7%  25.0%    ---------------------------------------------------------------------------gttataccattcgtggcaagtgtggctgccgaaatga----------tgcagcacgtgtattgtgcagcttccaaaaaatgt------------------------------------------------------     81 F1S32-P79-TRINITY_DN13210_c0_g1_i1-9.0e+00-odorranain-X1a    74.4%  23.0%    ------------------------------------cattttct------------------------------------------------ggacatactcgcgagcgggc----------tgcctaagctgatttctctaattaagggt------------------------------------------------------------     77 F1S22-P65-TRINITY_DN98_c53_g1_i1-3.6e+03-odorranain-T1       51.3%  22.4%    a------cctctagatgctata-----------taggatatc--------------------------------------------------------------------------------ggcgcaaagtagtttgttca---------------------------------------------------------------------     57 F1S9-P30-TRINITY_DN11504_c0_g1_i1-4.8e+00-nigrocin-OG35      89.7%  22.4%    ------------------------------------ggtctttt------------------------------------------------acgtggcatcctcggtgcgg----------ggaagacaatactatgtgaacttagccggatgtgc------------------------------------------------------     58 F1S9-P25-TRINITY_DN49_c0_g1_i1-5.8e+03-nigrocin-2GRb         89.7%  22.4%    ------------------------------------ggtctttt------------------------------------------------cggtaaaatcctcggtgtgg----------ggaagaaagtactatgtggacttagcgggatgtgc------------------------------------------------------     84 F1S20-P63-TRINITY_DN132_c0_g1_i4-8.5e+02-odorranain-Q1       97.4%  21.9%    -----------------------------------------------------------------------------------------gcgccgtttcgtatgtggtatatg------taccacaaactgaaagatatggaaccaaaaccaatggcg-----------------------------------------------------     85 F1S21-P64-TRINITY_DN638_c6_g1_i1-2.4e+02-odorranain-S1       59.0%  19.6%    t---------------------------------------------------------------------------------------------ttttgccccctagtcctt----------ggaaagaaacattccgaacaagt------------------------------------------------------------------     23 F1S12-P45-TRINITY_DN2213_c1_g1_i2-2.4e+00-odorranain-C15     89.7%  19.1%    ---------------------------------------------------------ggtacggtaaaggatttgcttattggtgcaggcaagagtgcggcccagcgtgtgc----------tgaaaacattatcttgtacactttctaacgattgt------------------------------------------------------     49 F1S9-P29-TRINITY_DN16_c2_g1_i1-5.9e+00-nigrocin-OG34         89.7%  18.8%    ---------------------------------------ctttt------------------------------------------------aagtggcatcctcggtacgg----------ggaagcatatagtatgtggacttagcgggctgtgc------------------------------------------------------     14 F1S6-P9-TRINITY_DN0_c1_g1_i4-9.2e-01-brevinin-2GRa           89.7%  18.4%    ------------------------------------ggtctcctg------------gatacattcaagaatctggcccttaatgcggccaagagtgcgggtgtgagtgtac----------tgaattcattatcttgtaaactttctaaaacatgt------------------------------------------------------     15 F1S6-P9-TRINITY_DN0_c1_g1_i14-1.3e+04-brevinin-2GRa          89.7%  18.4%    ------------------------------------ggtctcctg------------gatacattcaagaatctggcccttaatgcggccaagagtgcgggtgtgagtgtac----------tgaattcattatcttgtaaactttctaaaacatgt------------------------------------------------------     22 F1S12-P43-TRINITY_DN2658_c0_g2_i1-3.3e-01-odorranain-C13     89.7%  18.4%    ------------------------------------ggtgtcctg------------ggtacagtaaagaatttgcttattggtgcaagcaagagtgcggcccagagtgtgc----------tgaaaacattatcttgtaaactttccaacgattgt------------------------------------------------------     24 F1S12-P44-TRINITY_DN2213_c1_g1_i1-5.8e+00-odorranain-C14     89.7%  18.1%    ---------------------------------------------------------ggtacggtaaaggatttgcttattggtgcaggcaagagtgcggcccagcgtgtgc----------tgaaaacattatcttgtaaacattctaatgattgt------------------------------------------------------     48 F1S9-P24-TRINITY_DN77_c0_g1_i1-7.4e+01-nigrocin-2GRa         89.7%  17.9%    ------------------------------------ggtctttt------------------------------------------------aagtggcatcctcggtgcgg----------ggaagcatatagtctgtggacttagcgggctgtgc------------------------------------------------------     52 F1S9-P26-TRINITY_DN0_c1_g1_i17-1.5e+04-nigrocin-2GRc         89.7%  17.9%    ------------------------------------ggtctttt------------------------------------------------aagtggcatcctcggtgcgg----------ggaagaacatagtatgtggacttagcgggctgtgc------------------------------------------------------     53 F1S9-P26-TRINITY_DN0_c1_g1_i3-1.2e+00-nigrocin-2GRc          89.7%  17.9%    ------------------------------------ggtctttt------------------------------------------------aagtggcatcctcggtgcgg----------ggaagaacatagtatgtggacttagcgggctgtgc------------------------------------------------------     54 F1S9-P26-TRINITY_DN0_c1_g1_i2-2.2e+04-nigrocin-2GRc          89.7%  17.9%    ------------------------------------ggtctttt------------------------------------------------aagtggcatcctcggtgcgg----------ggaagaacatagtatgtggacttagcgggctgtgc------------------------------------------------------     68 F1S30-P77-TRINITY_DN0_c174_g2_i1-9.7e+03-pleurain-E-OG1      74.4%  17.7%    g-------------------------------caacaccctg--------------------------------gggaattccaccaaatggg----ataccaccaattgtagctg---ttcggataagaccactttgtggaactgtt---------------------------------------------------------------     27 F1S13-P46-TRINITY_DN6_c27_g1_i1-4.8e+03-odorranain-F2        89.7%  17.6%    ------------------------------------ggtttcatg------------gatacggccaagaa------------cgtagccaagaatgtggccgtgactttgc----------tagacaatttaaaatgtaaaattactaaagcatgt------------------------------------------------------     28 F1S13-P46-TRINITY_DN10285_c0_g1_i1-3.0e+00-odorranain-F2     89.7%  17.6%    ------------------------------------ggattcatg------------gatacggccaagaa------------cgtagccaagaatgtggccgtgactttgc----------tagacaatttaaaatgtaaaattacaaaagcatgt------------------------------------------------------     17 F1S12-P39-TRINITY_DN0_c1_g1_i15-2.2e+01-brevinin-2GRb        89.7%  17.5%    ------------------------------------ggtgtcctg------------ggtacggtaaagaatttgcttattggtgcaggcaagagtgcggcccagagtgtgc----------tgaaaacattatcttgtaaactttctaacgattgt------------------------------------------------------     18 F1S12-P39-TRINITY_DN0_c1_g1_i10-8.0e+03-brevinin-2GRb        89.7%  17.5%    ------------------------------------ggtgtcctg------------ggtacggtaaagaatttgcttattggtgcaggcaagagtgcggcccagagtgtgc----------tgaaaacattatcttgtaaactttctaacgattgt------------------------------------------------------     19 F1S12-P39-TRINITY_DN0_c1_g1_i23-8.4e+00-brevinin-2GRb        89.7%  17.5%    ------------------------------------ggtgtcctg------------ggtacggtaaagaatttgcttattggtgcaggcaagagtgcggcccagagtgtgc----------tgaaaacattatcttgtaaactttctaacgattgt------------------------------------------------------     20 F1S12-P39-TRINITY_DN0_c1_g1_i11-1.7e+00-brevinin-2GRb        89.7%  17.5%    ------------------------------------ggtgtcctg------------ggtacggtaaagaatttgcttattggtgcaggcaagagtgcggcccagagtgtgc----------tgaaaacattatcttgtaaactttctaacgattgt------------------------------------------------------     21 F1S12-P40-TRINITY_DN45_c27_g1_i1-7.5e+02-odorranain-C7       89.7%  17.5%    ------------------------------------ggtgtcctg------------ggtacggtaaaggatttgcttattggtgcaggcaagagtgcggcccagagtgtgc----------tgaaaacattatcttgtaaactttctaacgattgt------------------------------------------------------     89 F1S2-P2-TRINITY_DN142_c0_g1_i5-5.0e+01-andersonin-R          82.1%  17.1%    ------------------------------------------------------------------------------agcgcggaccagacaggaatgaacaaagctgcgc----------tgagtccaatacgttttatatcaaaaagtgtt---------------------------------------------------------     16 F1S6-P11-TRINITY_DN6490_c1_g1_i1-8.1e+00-brevinin-2E-OG8     89.7%  16.5%    ------------------------------------ggtctcctg------------gataccttcaagaatctggcccttaatgcggccacgagtgcgggggtgtgtgtac----------ggaattcattatctcgtaaactttctaaaacatgt------------------------------------------------------     25 F1S12-P41-TRINITY_DN10924_c1_g1_i1-2.4e+00-odorranain-C11    89.7%  16.5%    ------------------------------------ggtttcctg------------ggtacggtaaagaatttgcgtattggtgcaggcaagagtgcggcacagagtgtgc----------tgaaaacattatctggtaaactttctaacgattgt------------------------------------------------------     29 F1S13-P47-TRINITY_DN1102_c1_g1_i1-1.1e+00-odorranain-F3      89.7%  16.5%    ------------------------------------ggcttaatg------------gatacggacaagaa------------cgtaacccagaatgttgccgggactttgc----------tagacaatttaaaatgtaaaatcactaaagcatgt------------------------------------------------------     67 F1S16-P53-TRINITY_DN25_c0_g1_i1-2.1e+03-odorranain-M2        82.1%  16.5%    g-------------------------------caacagcctg--------------------------------ggacttcggaccacacggg----ctgctaccaatacgcccta---ttcggataagaccactttgtggaaaagataaa---------------------------------------------------------tct     50 F1S9-P27-TRINITY_DN9643_c0_g1_i4-2.5e+00-nigrocin-OG32       89.7%  16.4%    ------------------------------------tgtctttt------------------------------------------------aagtggcatcctcggtgcgg----------ggaagcatatagtatgtggactgagcgggctgtgc------------------------------------------------------     51 F1S9-P24-TRINITY_DN1399_c0_g1_i1-4.6e+01-nigrocin-2GRa       89.7%  16.4%    ------------------------------------gggctttt------------------------------------------------aagtggcatcctcggtgcgg----------gtaagcatatagtatgtggacttagcgggctgtgc------------------------------------------------------     55 F1S9-P28-TRINITY_DN4414_c6_g1_i1-5.3e+00-nigrocin-OG33       89.7%  16.4%    ------------------------------------ggtctttt------------------------------------------------aagtggcatcctcggtgcgg----------ggaagcatataatatgtggactgagtggggtgtgc------------------------------------------------------     86 F1S28-P75-TRINITY_DN0_c1_g1_i20-5.5e+03-OGTI                 89.7%  16.4%    ---------------------------------------------------------------------------------------------------gctgtgaacattc----------cttttaaagtacattttaggtgtaaagccgcgttctg----------------------------------------------t-----     87 F1S28-P75-TRINITY_DN603_c0_g1_i1-4.5e+01-OGTI                89.7%  16.4%    ---------------------------------------------------------------------------------------------------gctgtgaacattc----------cttttaaagtacattttaggtgtaaagccgcgttctg----------------------------------------------t-----     88 F1S28-P75-TRINITY_DN603_c0_g1_i3-5.1e-01-OGTI                89.7%  16.4%    ---------------------------------------------------------------------------------------------------gctgtgaacattc----------cttttaaagtacattttaggtgtaaagccgcgttctg----------------------------------------------t-----     72 F1S11-P35-TRINITY_DN11239_c0_g1_i2-7.5e+03-odorranain-B1     64.1%  15.9%    ------------------------------------------------------------------------------gctgcgctcaaagggtgctggaccaagagtatacc--------accaaagccgtgttttggaaaaaga-----------------------------------------------------------------     26 F1S12-P42-TRINITY_DN1218_c4_g1_i1-2.4e+00-odorranain-C12     89.7%  15.5%    ------------------------------------gggggcctg------------ggtacggtaaagaattggcgtattggtgcaggcaagagtgcggcccagcgtgtgc----------tgaaaaccttatcttttaaaatttctaacgattgt------------------------------------------------------     61 F3-P86-TRINITY_DN6_c0_g1_i12-1.0e+03-tachykinin_OG1          59.0%  15.2%    ---------------------------------------------------------------------------------------------gacgacaccgaggacttgg----------caaacaaattcatcggcctaatg------------------------------------------------------------------     42 F1S8-P17-TRINITY_DN96_c0_g2_i2-1.4e+02-esculentin-2-OG8      89.7%  14.8%    ------------------------------------ggtattttctcgatacttaaaattgcaaccaagttgattggcaagactctggccaaggcagcaggcaaggctgggg----------cggaacttgcggcttgtaaagctgccaatcaatgt------------------------------------------------------     43 F1S8-P22-TRINITY_DN96_c0_g1_i1-6.4e+00-esculentin-2-OG20     89.7%  14.8%    ------------------------------------ggcattttcgcaatacttaaaattgcaaccaagttgattggcaagactctggccaaggcagcaggcaaggctgggg----------cggaacttgcggcttgtaaagctgccaatcaatgt------------------------------------------------------     33 F1S29-P76-TRINITY_DN8472_c0_g1_i1-2.5e+03-palustrin-OG2      89.7%  14.4%    ------------------------------------ggtctctgg------------gacaccatcaagca------------agcggggaagaagttttttctgaatgtgt----------tggataagataagatgtaaagttgctggaggatgtagaa------------------------------------------------ca     73 F1S11-P38-TRINITY_DN1399_c4_g1_i1-2.5e+01-odorranain-B8      64.1%  14.3%    ------------------------------------------------------------------------------gctgcactcaaagggcgctggaccaagagtatacc--------accaaagccttggtttggaaaaaga-----------------------------------------------------------------     40 F1S8-P16-TRINITY_DN96_c0_g1_i2-2.3e+01-esculentin-2-RA1      89.7%  13.9%    ------------------------------------ggcattttcgcaatacttaaaattgcaaccaagttgattggcaagactctggccaaggcagcaggcaaggctggga----------cgggacttttggcttgtaaagctgccaaagaatgt------------------------------------------------------     41 F1S8-P23-TRINITY_DN96_c0_g2_i1-3.5e-01-esculentin-2-OG21     89.7%  13.9%    ------------------------------------ggtattttctcgatacttaaaattgcaaccaagttgattggcaagactctggccaaggcagcaggcaaggctggga----------cgggacttttggcttgtaaagctgccaaagaatgt------------------------------------------------------     44 F1S8-P18-TRINITY_DN0_c1_g1_i22-8.3e+03-esculentin-2-OG10     89.7%  13.9%    ------------------------------------ggtcttttcacgttaatcaaaggtgcagccaagttgattggcaagactgtggccaaagaagcaggcaagactgggc----------ttgaacttatggcttgtaaaattaccaaccaatgt------------------------------------------------------     63 F1S3-P3-TRINITY_DN25_c0_g1_i2-5.2e+02-andersonin-S           53.8%  13.9%    g-------------------------------caacagcctt--------------------------------aggcattccaccacgtggg----tttctaccaatagttaataagtttaaggatataatactttgt------------------------------------------------------------------------     83 F1S4-P4-TRINITY_DN836_c0_g1_i2-1.3e+01-andersonin-X-OG1      76.9%  13.8%    -----------------------------------------------------------------------------------------cagatgtttcatttgtggtatttg---------agacaaatgaaaaataggaaaccaatggcg-----------------------------------------------------------     64 F1S16-P54-TRINITY_DN25_c0_g1_i3-2.7e+03-odorranain-M3        76.9%  13.6%    g-------------------------------caacagcctt--------------------------------aggcctttcatcacgtggg----ttgctaccaataggttttatgtttaaggatacaatacgttgtagaaaatat---------------------------------------------------------------     65 F1S16-P55-TRINITY_DN3181_c1_g1_i1-3.5e+02-odorranain-M4      76.9%  13.6%    g-------------------------------caacagcctt--------------------------------aggcctttcatcacgtgga----gtactaacaataggttttatgtttaaggatacaagacgttgtagaaaatat---------------------------------------------------------------     90 F1S17-P56-TRINITY_DN122946_c2_g1_i1-1.8e+03-odorranain-O1   100.0%  13.3%    ----------------------------------------------------------------------------------------------gccgtgcccttgatatataa----ccgccctggtatatatgtcaccaaaagaccaaaaggaaaa-----------------------------------------------------     91 F1S17-P57-TRINITY_DN38944_c0_g1_i1-6.3e-01-odorranain-O4    100.0%  13.3%    ----------------------------------------------------------------------------------------------gccgtgcccttgatatataa----ccgcccttgtatatatgtcaccaaaagaccaaaaggaaaa-----------------------------------------------------     62 F3-P87-TRINITY_DN6_c0_g1_i6-8.0e+02-ranamargarin             59.0%  13.0%    ---------------------------------------------------------------------------------------------gacgacgcctcagacaggg----------caaaaaaattctacggcctaatg------------------------------------------------------------------     59 F1S19-P61-TRINITY_DN4628_c1_g1_i1-1.2e+00-odorranain-P2d     89.7%  11.8%    ------------------------------------ggtctttt------------------------------------------------aagtggcatcatccgcgcgg----------gcaagcacatagtacgtggacttagcgcgccgtcccaaagctccaatacca------------------------------------aa     71 F1S10-P31-TRINITY_DN7347_c0_g1_i1-4.9e+03-odorranain-A8      51.3%  11.5%    g------tctttaaatgct--------------ataagcct----------------------------------------------------------------gacagtc----------gagggtttcaagtttgtgaa---------------------------------------------------------------------     74 F1S11-P36-TRINITY_DN79_c1_g3_i1-3.4e+03-odorranain-B6        64.1%  11.1%    ------------------------------------------------------------------------------gctgcactcagagggtgctggaccaagagtatacc--------accaaagccttgttcaggaaaaaga-----------------------------------------------------------------     46 F1S24-P69-TRINITY_DN122936_c0_g1_i1-4.3e+02-odorranalectin   51.3%  10.9%    tatgcatcccccaaatgcttta--------------ggtacccaa-----------------------------------------------------------------------------atggagttttagcgtgtaca---------------------------------------------------------------------     47 F1S24-P69-TRINITY_DN106_c6_g1_i1-2.1e+01-odorranalectin      51.3%  10.9%    tatgcatcccccaaatgcttta--------------ggtacccaa-----------------------------------------------------------------------------atggagttttagcgtgtaca---------------------------------------------------------------------      2 F1S5-P5-TRINITY_DN23413_c1_g1_i1-1.3e+00-gaegurin-6-OG1      35.9%  10.9%    ---------------------------------------------------------------------------tttttacaacttcttgcaggtctggctgctaatttct----------tgccgtcaatatca---------------------------------------------------------------------------     56 F1S19-P60-TRINITY_DN39_c0_g1_i2-7.2e+00-odorranain-P2c       89.7%  10.7%    ------------------------------------ggtctttt------------------------------------------------aagtggcatcctcggtgcgg----------ggaagcatatagtatgtggacttagcgggccatctgctgctctcagtgtgtcgccggtaaacgttcgccttgtggtggtagaacggcaa     34 F1S7-P12-TRINITY_DN81_c0_g1_i1-9.5e+03-esculentin-1-OG5      89.7%  10.6%    ggtcttttctccaaatttgccgggaaagggattaaggatttgatc---------ttcaaaggggtcaagcacataggcaaggaagttggcatggatgtgatcagaactggga----------tagatgttgcaggttgtaaaattaaaggtgaatgt------------------------------------------------------     35 F1S7-P12-TRINITY_DN0_c1_g1_i16-1.0e+00-esculentin-1-OG5      89.7%  10.6%    gggcttttctccaaatttgccgggaaagggattaaggatttgatc---------ttcaaaggggtcaagcacataggcaaggaagttggcatggatgtgatcagaactggga----------tagatgttgcaggttgtaaaattaaaggtgaatgt------------------------------------------------------     36 F1S7-P12-TRINITY_DN0_c1_g1_i18-2.7e+03-esculentin-1-OG5      89.7%  10.6%    gggcttttctccaaatttgccgggaaagggattaaggatttgatc---------ttcaaaggggtcaagcacataggcaaggaagttggcatggatgtgatcagaactggga----------tagatgttgcaggttgtaaaattaaaggtgaatgt------------------------------------------------------     38 F1S7-P13-TRINITY_DN259_c0_g1_i1-1.7e+02-esculentin-1-OG12    89.7%  10.6%    gggcttttctccaaatttgccgggaaaggggttaagaatttctta---------atcaaagggggcaagcacataggcaaggaagttggcatggatgtgatcagaactggga----------tagatgttgcaggttgtaaaattaaagttgaatgt------------------------------------------------------     60 F1S15-P51-TRINITY_DN45_c1_g1_i1-3.1e+03-odorranain-L2        66.7%   9.6%    gtgga---------------------------------------------------------------------agttcaagtgagagacaaaggcaaaggcatatatgggc----------tgagcccattacgtcaaccagcacct---------------------------------------------------------------     32 F1S26-P72-TRINITY_DN132_c0_g1_i5-2.4e+02-OGA1                25.6%   9.3%    -----------------------------------------------------------------------------------tgcgggtacaaacatggcagggcgaattg----------tggacgagga-------------------------------------------------------------------------------     76 F1S35-P82-TRINITY_DN360_c0_g1_i1-7.9e+02-odorranain-X4a      35.9%   9.2%    ---------------------------------------------------------------------------cgttttgtatatacaatagattcttccggtaatatac--------------------attttggaaaaaga-----------------------------------------------------------------     37 F1S7-P15-TRINITY_DN12856_c2_g1_i1-2.7e-01-esculentin-1-OG14  89.7%   9.1%    ggtcttttctccaaatttgccgggaaagtgattaaggatttgctc---------ttcaaaggggtcaagcacataggcaaggaagttggcatggatgtgatcagaactggga----------tagatgttgcaggttataaaattaaaggtgactgttcaaacctgaat------------------------------------------     12 F1S19-P62-TRINITY_DN638_c0_g1_i2-3.9e+00-odorranain-P2e      89.7%   8.9%    ------------------------------------ggtctcatc------------gatacatccaagggt----------------------gcactgccaatag---------------tagatcagatattatgtcaaatggctggaggatgtaaaaagttgaatcagaagtcatctgatgtgaaatatcatttagcta------aa     66 F1S33-P80-TRINITY_DN1399_c2_g1_i1-7.7e+00-odorranain-X2a     28.2%   7.6%    g-------------------------------caacagcctt--------------------------------aggcctttcatcacgtggggagacctctactgaagtcttccagctgtctacattc----------------------------------------------------------------------------------     30 F1S26-P71-TRINITY_DN132_c0_g1_i3-1.8e+02-ishikawain-7-EV1    17.9%   7.5%    -----------------------------------------------------------------------------------tgcgggtacagacatggcaggttgtattg----------tggacgt----------------------------------------------------------------------------------     31 F1S26-P71-TRINITY_DN132_c0_g1_i1-3.5e+02-ishikawain-7-EV1    17.9%   7.5%    -----------------------------------------------------------------------------------tgcgggtacagacatggcaggttgtattg----------tggacgt----------------------------------------------------------------------------------     82 F1S25-P70-TRINITY_DN1048_c0_g1_i1-1.9e+02-odorranaopin       23.1%   4.9%    --------------------------------------------------------------------------gattacaccattagaaccaggcttcaccaggaatcatcaa----------------------------------ggaaagttttg----------------------------------------------------     75 F1S11-P37-TRINITY_DN56_c1_g1_i1-5.0e+01-odorranain-B7        25.6%   4.2%    ------------------------------------------------------------------------------gctgcactcaaagggtgctggaccaagagtataac--------accaacgcct--------------------------------------------------------------------------------     78 F1S10-P32-TRINITY_DN25_c1_g1_i1-7.6e+03-odorranain-A9         2.6%   1.9%    g------tcgttaaatgctccta-----tcgaccagggtc------------------------------------------------------gcctgactcccgatgtaa----------t----------------------------------------------------------------------------------------     79 F1S10-P33-TRINITY_DN25595_c0_g1_i1-3.1e+00-odorranain-A10     2.6%   1.9%    g------tcgttaaatgctccta-----tctaccagggtc------------------------------------------------------gcctgactcccgatgtaa----------t----------------------------------------------------------------------------------------     80 F1S10-P34-TRINITY_DN6115_c1_g1_i1-2.5e+03-odorranain-A11      2.6%   1.9%    g------tcgttacatgctccta-----tcgaccagggtc------------------------------------------------------gcctgtctcccgatgtaa----------t----------------------------------------------------------------------------------------     10 F1S34-P81-TRINITY_DN17503_c0_g1_i1-1.2e+00-odorranain-X3a     0.0%   0.0%    ---------------------------------------------------------------------------gttataccgtttgtggcaagcggggctgcc----------------------------------------------------------------------------------------------------------     13 F1S27-P74-TRINITY_DN139_c0_g1_i1-3.1e+02-OGC-RA3              0.0%   0.0%    t--------------------------------aca--------------------------------------------------------------------------------------------------------------------------------tgaattggaagtcacctgatgtgatatatcatttggcta------aa     39 F1S7-P14-TRINITY_DN4249_c0_g1_i1-2.1e+03-esculentin-1-OG13    0.0%   0.0%    gggcttttctccaaattttccgggaaagggattaagaatttc-------------------------------------------------------------------------------------------------------------------------------------------------------------------------     45 F1S8-P19-TRINITY_DN2168_c4_g1_i1-6.8e+00-esculentin-2-OG17    0.0%   0.0%    ------------------------------------ggtctttgcacgttaatcaaaggtgcagccaagttgattggcaagactgtggccaaa----------------------------------------------------------------------------------------------------------------------        clustal                                                                                                                                                                                                                                                                                                 consensus/75%                                                                .............................................................................................................................................A..................................................................... ``` |

MView 1.67, Copyright © 1997-2020 Nigel P. Brown
