# Supplementary material for: Integrative transcriptomics and peptidomics approach reveals unexpectedly diverse endogenous secretory peptides in Odorrana grahami frog skin
Source: BMC Biol. 2025 Nov 28;23:354. doi: 10.1186/s12915-025-02463-w (PMC12664280; doi:10.1186/s12915-025-02463-w)
Supplement: Supplementary file 5 — Additional file 5. Alignments of ESP sequences identified in this study across different regions. [file 12915_2025_2463_MOESM5_ESM.zip › Additional file 5/FSAP family - signal peptide plus up to 45 nucleotides upstream of the 5’-UTR “translated”.html]

MView


|  |
| --- |
| ``` Reference sequence (1): F1S1-P1-TRINITY_DN175_c1_g1_i1-9.3e+02-andersonin-Q Identities normalised by aligned length. Colored by: consensus group/60% ``` |
| ```                                                                   cov    pid  1 [        .         .         .         .         : ] 52  1 F1S1-P1-TRINITY_DN175_c1_g1_i1-9.3e+02-andersonin-Q         100.0% 100.0%    ---------HQLNYPNP------KMFTLKKSLLLLFFLATINLSLC------    23 F1S9-P30-TRINITY_DN11504_c0_g1_i1-4.8e+00-nigrocin-OG35      87.1%  92.6%    -------------YPSP------KMFTLKKSLLLLFFLGTINLSLC------    42 F1S5-P5-TRINITY_DN23413_c1_g1_i1-1.3e+00-gaegurin-6-OG1      74.2%  91.3%    -----------------------KMFTLKKSLLLLFFPGTINLSLC------     9 F1S10-P32-TRINITY_DN25_c1_g1_i1-7.6e+03-odorranain-A9        74.2%  91.3%    -----------------------KMFTLKKSLLLLFFLGTISLSLC------    21 F1S5-P8-TRINITY_DN33233_c1_g1_i1-1.1e+02-brevinin-1E-OG10    80.6%  88.0%    ---------------SP------KMFTMKKSLLLLFFLGTINLSLC------    17 F1S8-P23-TRINITY_DN96_c0_g2_i1-3.5e-01-esculentin-2-OG21     71.0%  86.4%    ------------------------MFTLKKSLLLFFFLGTISLSLC------     3 F1S4-P4-TRINITY_DN836_c0_g1_i2-1.3e+01-andersonin-X-OG1      83.9%  84.6%    --------------PSP------KMFTFKKSLLLLFFLGTISLSLC------    25 F1S10-P34-TRINITY_DN6115_c1_g1_i1-2.5e+03-odorranain-A11    100.0%  84.4%    --------QHQLNYPSP------KMFTMKKSLLLLFFLGTISLSLC------    43 F1S17-P57-TRINITY_DN38944_c0_g1_i1-6.3e-01-odorranain-O4     93.5%  82.8%    -----------LICVSP------KMFTLKKSLLLLFFLGTINLSLC------    22 F1S36-P83-TRINITY_DN14764_c0_g1_i2-4.9e+02-odorranain-X5a   100.0%  81.2%    --------HNQLNHPSP------KMFTMKKSLLLLFFLGTINLSLC------    15 F1S9-P26-TRINITY_DN0_c1_g1_i2-2.2e+04-nigrocin-2GRc         100.0%  78.4%    ---SCPHSHHQLNYPIP------KMFTLKKSLLLLFFLGTINLSLC------    76 F1S12-P43-TRINITY_DN2658_c0_g2_i1-3.3e-01-odorranain-C13     74.2%  78.3%    -----------------------KMFTMKKPLLLPFFLRTISLSLC------    82 F1S23-P68-TRINITY_DN128039_c0_g1_i1-2.6e+03-odorranain-U3    74.2%  78.3%    -----------------------KMCTGKKSLLLLFFLVSIALSLC------    56 F1S11-P38-TRINITY_DN1399_c4_g1_i1-2.5e+01-odorranain-B8      96.8%  76.7%    ----------QLNYPSP------KMFTLKKPLLLLFFLGIVALSVC------    57 F1S11-P37-TRINITY_DN56_c1_g1_i1-5.0e+01-odorranain-B7        93.5%  75.9%    -----------LNYPSP------KMFTLKKPLLLLFFLGSVSLSVC------    10 F1S12-P39-TRINITY_DN0_c1_g1_i10-8.0e+03-brevinin-2GRb       100.0%  75.7%    ---SCPHSHHQLNYPIP------KMFTLKKSLLLLFFLGTISLSLC------    11 F1S12-P39-TRINITY_DN0_c1_g1_i11-1.7e+00-brevinin-2GRb       100.0%  75.7%    ---SCPHSHHQLNYPIP------KMFTLKKSLLLLFFLGTISLSLC------    12 F1S27-P74-TRINITY_DN139_c0_g1_i1-3.1e+02-OGC-RA3            100.0%  75.7%    ---SCLHSHHQLNYPSP------KMFTLKKSLLLLFFLGTISLSLC------    20 F1S5-P7-TRINITY_DN23816_c1_g1_i1-4.5e+02-brevinin-1E-OG9    100.0%  75.7%    ---SCLHSQHQLNYPSP------KMFTMKKSLLLLFFLGTINLSLC------    26 F1S25-P70-TRINITY_DN1048_c0_g1_i1-1.9e+02-odorranaopin      100.0%  75.7%    ---GCLHSQHQLNYPSP------KMFTLKKSLLLLFFLGTISLSLC------    27 F1S20-P63-TRINITY_DN132_c0_g1_i4-8.5e+02-odorranain-Q1      100.0%  75.7%    ---GYLHSQHQLNYPSP------KMFTLKKSLLLLFFLGTISLSLC------    28 F1S26-P71-TRINITY_DN132_c0_g1_i3-1.8e+02-ishikawain-7-EV1   100.0%  75.7%    ---GYLHSQHQLNYPSP------KMFTLKKSLLLLFFLGTISLSLC------    34 F1S9-P25-TRINITY_DN49_c0_g1_i1-5.8e+03-nigrocin-2GRb        100.0%  75.7%    ---SCLHSQHQLNYLSP------KMFTLKKSLLLLFFLGTINLSLC------    41 F1S17-P56-TRINITY_DN122946_c2_g1_i1-1.8e+03-odorranain-O1   100.0%  75.7%    ---SCLQSQHQLNYPSA------KMFTLKKSLLLLFFLGTINLSLC------     7 F1S6-P9-TRINITY_DN0_c1_g1_i4-9.2e-01-brevinin-2GRa          100.0%  75.7%    ---SCPHSHHQLNYPIP------KMFTLKKSLLLLFFLGTISLSLC------    77 F1S14-P50-TRINITY_DN603_c2_g1_i1-5.0e+02-odorranain-G1      100.0%  75.7%    ---FFCISQYQLNYLNP------KMLTLKKSLLLLFFLATINLSLC------     8 F1S6-P9-TRINITY_DN0_c1_g1_i14-1.3e+04-brevinin-2GRa         100.0%  75.7%    ---SCPHSHHQLNYPIP------KMFTLKKSLLLLFFLGTISLSLC------    31 F1S32-P79-TRINITY_DN13210_c0_g1_i1-9.0e+00-odorranain-X1a   100.0%  74.3%    -----LHSQHQLNYPSP------KMFTLKKSLVLLFFLGTISLTLC------    68 F1S34-P81-TRINITY_DN17503_c0_g1_i1-1.2e+00-odorranain-X3a   100.0%  73.5%    ------HSHHQLNYPIQ------KMFTLKKSMLLLFFLGAISLSLC------    13 F3-P86-TRINITY_DN6_c0_g1_i12-1.0e+03-tachykinin_OG1         100.0%  73.0%    ---SCLHSHHQLIYPIP------KMFTLKKSLLLLFFLGTISLSLC------    14 F3-P87-TRINITY_DN6_c0_g1_i6-8.0e+02-ranamargarin            100.0%  73.0%    ---SCLHSHHQLIYPIP------KMFTLKKSLLLLFFLGTISLSLC------    18 F1S8-P17-TRINITY_DN96_c0_g2_i2-1.4e+02-esculentin-2-OG8     100.0%  73.0%    ---SCLHSQHQPNYPSP------KMFTLKKSLLLLFFLGTISLSLC------    19 F1S8-P22-TRINITY_DN96_c0_g1_i1-6.4e+00-esculentin-2-OG20    100.0%  73.0%    ---SCLHSQHQPNYPSP------KMFTLKKSLLLLFFLGTISLSLC------    32 F1S12-P40-TRINITY_DN45_c27_g1_i1-7.5e+02-odorranain-C7      100.0%  73.0%    ---SCLHSQHQLNYPSP------KMFTLKKSLLLLFFIGTISLSLC------    33 F1S29-P76-TRINITY_DN8472_c0_g1_i1-2.5e+03-palustrin-OG2     100.0%  73.0%    ---SCLHSQHQLNYPSP------KMFTLKKSLLLLFFIGTISLSLC------     4 F1S5-P6-TRINITY_DN0_c1_g1_i24-1.9e+03-brevinin-1E-OG3       100.0%  73.0%    ---SCPHSHHQLNYPIP------KMFTLKKSMLLLFFLGTISLSLC------     5 F1S7-P12-TRINITY_DN0_c1_g1_i16-1.0e+00-esculentin-1-OG5     100.0%  73.0%    ---SCPHSHHQLNYPIP------KMFTLKKSMLLLFFLGTISLSLC------     6 F1S9-P26-TRINITY_DN0_c1_g1_i17-1.5e+04-nigrocin-2GRc        100.0%  73.0%    ---SCPHSHHQLNYPIP------KMFTLKKSMLLLFFLGTISLSLC------    90 F1S9-P28-TRINITY_DN4414_c6_g1_i1-5.3e+00-nigrocin-OG33      100.0%  71.9%    --------QPPLKYWGP------MMFPLKKSLLLLFFLGTINLSLC------     2 F1S2-P2-TRINITY_DN142_c0_g1_i5-5.0e+01-andersonin-R         100.0%  71.4%    -----LHSQHQLNYPSS------KMFTLKKSLLLLFFIGMISLSLC------    72 F1S19-P61-TRINITY_DN4628_c1_g1_i1-1.2e+00-odorranain-P2d    100.0%  71.4%    -----LHSQHQLNHPSP------KMFTRKKSLLLLFFLGTIDLCLC------    16 F1S8-P16-TRINITY_DN96_c0_g1_i2-2.3e+01-esculentin-2-RA1     100.0%  70.3%    ---SCLHSQHQPNYPSP------KMFTLKKSLLLFFFLGTISLSLC------    29 F1S26-P71-TRINITY_DN132_c0_g1_i1-3.5e+02-ishikawain-7-EV1   100.0%  70.3%    ---GYLHSQHQLNYPSP------KMFTLKKTLLILFFLGTISLSLC------    30 F1S26-P72-TRINITY_DN132_c0_g1_i5-2.4e+02-OGA1               100.0%  70.3%    ---GYLHSQHQLNYPSP------KMFTLKKTLLILFFLGTISLSLC------    35 F1S24-P69-TRINITY_DN122936_c0_g1_i1-4.3e+02-odorranalectin  100.0%  70.3%    ---SCLHSQHQLDYPSP------KMFTLKKSLLLLFFLGIISLSLC------    44 F1S19-P60-TRINITY_DN39_c0_g1_i2-7.2e+00-odorranain-P2c      100.0%  70.3%    ---IGLHSXFQLNHQSP------KMFTLKKSLLLLFFLGTINLSLC------    60 F1S19-P62-TRINITY_DN638_c0_g1_i2-3.9e+00-odorranain-P2e     100.0%  70.3%    ---YRLHSQHQLNYLSP------KMFTLKKPLLLLFFLGTISLSLC------    58 F1S11-P36-TRINITY_DN79_c1_g3_i1-3.4e+03-odorranain-B6       100.0%  69.7%    -------SPHQLNYPSP------KMFTLQKPLLLLFFLGIVSLSFC------    45 F1S12-P41-TRINITY_DN10924_c1_g1_i1-2.4e+00-odorranain-C11    74.2%  69.6%    -----------------------KMFTMKKYLLVLFFLGIVSLSLC------    54 F1S7-P14-TRINITY_DN4249_c0_g1_i1-2.1e+03-esculentin-1-OG13   93.5%  69.0%    -----------LNYPSP------KMFTLKKPLLLIVLLGIISLALC------    79 F1S18-P58-TRINITY_DN5345_c0_g1_i2-5.3e+03-odorranain-P1b    100.0%  68.8%    --------APTAKSSSP------KMFTLKKSLLLLFLLGTINLSLC------    83 F1S9-P29-TRINITY_DN16_c2_g1_i1-5.9e+00-nigrocin-OG34        100.0%  68.8%    --------QNQLNHQSP------QMLSLKKSLLHLFFLGTINLSLC------    84 F1S9-P24-TRINITY_DN1399_c0_g1_i1-4.6e+01-nigrocin-2GRa      100.0%  68.4%    --------KHQLNYPST------KMFTLKKSLFLLFFLGTINLSLWQDETNA    36 F1S8-P18-TRINITY_DN0_c1_g1_i22-8.3e+03-esculentin-2-OG10     96.8%  68.4%    --SSCLHSQHQLNY-SP------KMFTLNKSLLLLFFLGTISLSLC------    37 F1S12-P39-TRINITY_DN0_c1_g1_i15-2.2e+01-brevinin-2GRb        96.8%  68.4%    --SSCLHSQHQLNY-SP------KMFTLNKSLLLLFFLGTISLSLC------    38 F1S12-P39-TRINITY_DN0_c1_g1_i23-8.4e+00-brevinin-2GRb        96.8%  68.4%    --SSCLHSQHQLNY-SP------KMFTLNKSLLLLFFLGTISLSLC------    39 F1S22-P65-TRINITY_DN98_c53_g1_i1-3.6e+03-odorranain-T1       96.8%  68.4%    --SSCLHSQHQLNY-SP------KMFTLNKSLLLLFFLGTISLSLC------    40 F1S28-P75-TRINITY_DN0_c1_g1_i20-5.5e+03-OGTI                 96.8%  68.4%    --SSCLHSQHQLNY-SP------KMFTLNKSLLLLFFLGTISLSLC------    64 F1S16-P55-TRINITY_DN3181_c1_g1_i1-3.5e+02-odorranain-M4      71.0%  68.2%    ---------------SP------KMFTLKKFLLLLFFLGIVSS---------    70 F1S12-P42-TRINITY_DN1218_c4_g1_i1-2.4e+00-odorranain-C12     90.3%  67.9%    ------------XTTRP------KMFTMQKSLLLLFFLGAISLSLC------    85 F1S8-P19-TRINITY_DN2168_c4_g1_i1-6.8e+00-esculentin-2-OG17  100.0%  67.7%    ---------HQPNYPSP------KMLTMKKCMLVLFFRGTISLSLC------    24 F1S9-P27-TRINITY_DN9643_c0_g1_i4-2.5e+00-nigrocin-OG32      100.0%  67.6%    ---SCLHSQNQLNHPSP------KMFTMKKSLLLLFFLGTINLSIC------    49 F1S24-P69-TRINITY_DN106_c6_g1_i1-2.1e+01-odorranalectin     100.0%  67.6%    ---CDLHSQHQLNHPSP------KMFTMKKSLLLLFFLGIISLSLC------    59 F1S21-P64-TRINITY_DN638_c6_g1_i1-2.4e+02-odorranain-S1      100.0%  67.6%    ---SCLHSQHQLNYPSA------TMFTLKKSLLLLFFLGAISLSLC------    81 F1S35-P82-TRINITY_DN360_c0_g1_i1-7.9e+02-odorranain-X4a     100.0%  67.6%    ---------HQLNYPIPNYPQSSKMFTLKKSLLFLFFLGIISFSLC------    47 F1S13-P47-TRINITY_DN1102_c1_g1_i1-1.1e+00-odorranain-F3     100.0%  66.7%    ----RLHSQHQLNYPSP------KMFTMKKSLLVLFFLGIVSLSLC------    78 F1S15-P51-TRINITY_DN45_c1_g1_i1-3.1e+03-odorranain-L2        96.8%  66.7%    ----------RDKEMVP------KMFTMTKSLLLLFFLGTISLSLC------    67 F1S10-P31-TRINITY_DN7347_c0_g1_i1-4.9e+03-odorranain-A8      90.3%  65.0%    KFSSCQHSHHQLNYP---------MFTLKKSLLLLFFLGTISLSLC------    66 F1S23-P67-TRINITY_DN12170_c0_g1_i1-1.1e+00-odorranain-U2     64.5%  65.0%    -----------------------KMFTFKKFLLLLFFLGIASS---------    75 F1S9-P24-TRINITY_DN77_c0_g1_i1-7.4e+01-nigrocin-2GRa        100.0%  64.9%    ---SCLHCQHQLNHPSR------KMFTLKKSMLLLCFLGTISLSLC------    69 F1S6-P11-TRINITY_DN6490_c1_g1_i1-8.1e+00-brevinin-2E-OG8     96.8%  63.3%    ----------TNXTTRP------KMFTMKKSVLLLFFLGTISISLC------    55 F1S11-P35-TRINITY_DN11239_c0_g1_i2-7.5e+03-odorranain-B1    100.0%  63.2%    ---SCLHSQHQLNYPSP------KMFTLKKPLLLLFFLGIVSLSVCG-----    46 F1S13-P46-TRINITY_DN6_c27_g1_i1-4.8e+03-odorranain-F2       100.0%  62.2%    ---SCLHSQHQLNYSSP------KMFTMKKSLLVLFFLGIVSLSLC------    50 F1S5-P6-TRINITY_DN0_c1_g1_i6-6.1e+02-brevinin-1E-OG3        100.0%  62.2%    ---SCPHSHHQLNYPIP------KMFTLKKPLLLIVLLGIISLSLC------    51 F1S7-P12-TRINITY_DN0_c1_g1_i18-2.7e+03-esculentin-1-OG5     100.0%  62.2%    ---SCPHSHHQLNYPIP------KMFTLKKPLLLIVLLGIISLSLC------    52 F1S9-P26-TRINITY_DN0_c1_g1_i3-1.2e+00-nigrocin-2GRc         100.0%  62.2%    ---SCPHSHHQLNYPIP------KMFTLKKPLLLIVLLGIISLSLC------    53 F1S7-P12-TRINITY_DN81_c0_g1_i1-9.5e+03-esculentin-1-OG5     100.0%  62.2%    ---SCLHSQHQLNYPSP------KMFTLKKPLLLIVLLGIISLSLC------    86 F1S12-P44-TRINITY_DN2213_c1_g1_i1-5.8e+00-odorranain-C14     93.5%  62.1%    -----------MNXPSP------KMFTFRKSRVLLLVLGTISLSLC------    87 F1S12-P45-TRINITY_DN2213_c1_g1_i2-2.4e+00-odorranain-C15     93.5%  62.1%    -----------MNXPSP------KMFTFRKSRVLLLVLGTISLSLC------    61 F1S3-P3-TRINITY_DN25_c0_g1_i2-5.2e+02-andersonin-S           90.3%  61.8%    ---SCLHSKHQLNYPSP------KMFTLKKFLLLLFFLGIVSS---------    62 F1S16-P53-TRINITY_DN25_c0_g1_i1-2.1e+03-odorranain-M2        90.3%  61.8%    ---SCLHSKHQLNYPSP------KMFTLKKFLLLLFFLGIVSS---------    63 F1S16-P54-TRINITY_DN25_c0_g1_i3-2.7e+03-odorranain-M3        90.3%  61.8%    ---SCLHSKHQLNYPSP------KMFTLKKFLLLLFFLGIVSS---------    65 F1S33-P80-TRINITY_DN1399_c2_g1_i1-7.7e+00-odorranain-X2a     90.3%  61.8%    ---SCLHSKHQLNYPSP------KMFTLKKFLLLLFFLGIVSS---------    80 F1S18-P59-TRINITY_DN38049_c0_g1_i1-9.8e+01-odorranain-P1i   100.0%  60.6%    -------PAPTAKFSTP------TMFPLKTSLLLLFFLRTINLSLC------    48 F1S13-P46-TRINITY_DN10285_c0_g1_i1-3.0e+00-odorranain-F2     96.8%  60.0%    ----------TNXSTRP------KMFTMKKSLLVLFFLGIVSLSLC------    71 F1S10-P33-TRINITY_DN25595_c0_g1_i1-3.1e+00-odorranain-A10   100.0%  59.5%    ---TVATRRFIVYYPSP------KMFTMTKSLLLLFFLGTISLSLC------    74 F1S7-P15-TRINITY_DN12856_c2_g1_i1-2.7e-01-esculentin-1-OG14  93.5%  58.6%    -----------RDYSSP------KMFTLKKPLILIVLLGIISLSLC------    73 F1S7-P13-TRINITY_DN259_c0_g1_i1-1.7e+02-esculentin-1-OG12   100.0%  51.4%    ---SCVHPGRRRNYPRP------KMFTLKKPLLLIVLLGIISLSQC------    88 F1S30-P77-TRINITY_DN0_c174_g2_i1-9.7e+03-pleurain-E-OG1     100.0%  50.0%    ----CLHSQHQPNYPSP------KMLSLKTSLLLLFFIGIVSSSPCRG----    89 F1S28-P75-TRINITY_DN603_c0_g1_i1-4.5e+01-OGTI               100.0%  48.8%    ---SCLHSQHQLNYPSP------KMFTMKKSMLLLLFVGVIFGSLWEEHRDA    91 F1S28-P75-TRINITY_DN603_c0_g1_i3-5.1e-01-OGTI                93.5%  43.3%    -----------LHYKSD------KMFAMKKSLFVRLCVGGIQLSLWG-----       clustal                                                                                              * .  .  .                          consensus/75%                                                                ................P......KMFT.KK.LLLLFFLG.I.LSLC...... ``` |

MView 1.67, Copyright © 1997-2020 Nigel P. Brown
