# Supplementary material for: Integrative transcriptomics and peptidomics approach reveals unexpectedly diverse endogenous secretory peptides in Odorrana grahami frog skin
Source: BMC Biol. 2025 Nov 28;23:354. doi: 10.1186/s12915-025-02463-w (PMC12664280; doi:10.1186/s12915-025-02463-w)
Supplement: Supplementary file 5 — Additional file 5. Alignments of ESP sequences identified in this study across different regions. [file 12915_2025_2463_MOESM5_ESM.zip › Additional file 5/FSAP family - signal peptide plus up to 45 nucleotides upstream of the 5’-UTR.html]

MView


|  |
| --- |
| ``` Reference sequence (1): F1S1-P1-TRINITY_DN175_c1_g1_i1-9.3e+02-andersonin-Q Identities normalised by aligned length. Colored by: consensus group/60% ``` |
| ```                                                                   cov    pid  1 [        .         .         .         .         :         .         .         .         .         1         .         .         .         .         :         .     ] 166  1 F1S1-P1-TRINITY_DN175_c1_g1_i1-9.3e+02-andersonin-Q         100.0% 100.0%    ---------------------------caccaactg----------aactaccc------------------gaacccaaagatgttcaccttgaagaaatccctgttactccttttcttccttgcgaccatcaatttatctctctgt------------------     11 F1S9-P30-TRINITY_DN11504_c0_g1_i1-4.8e+00-nigrocin-OG35      87.1%  95.1%    -------------------------------------------------taccc------------------gagcccaaagatgttcaccttgaagaaatccctgttactccttttcttccttggtaccatcaacttatctctctgt------------------      3 F1S5-P5-TRINITY_DN23413_c1_g1_i1-1.3e+00-gaegurin-6-OG1      74.2%  94.2%    -------------------------------------------------------------------------------aagatgttcaccttgaagaaatccctgttactcctttttttccctgggaccatcaacttatctctctgt------------------      7 F1S5-P8-TRINITY_DN33233_c1_g1_i1-1.1e+02-brevinin-1E-OG10    80.6%  93.3%    -------------------------------------------------------------------------agcccaaagatgttcaccatgaagaaatccctgttactcctttttttccttgggaccatcaacttatctctctgt------------------     21 F1S10-P32-TRINITY_DN25_c1_g1_i1-7.6e+03-odorranain-A9        74.2%  92.8%    -------------------------------------------------------------------------------aagatgttcaccttgaagaaatccctgttactccttttctttcttgggaccatctccttatctctctgt------------------     18 F1S4-P4-TRINITY_DN836_c0_g1_i2-1.3e+01-andersonin-X-OG1      83.9%  91.0%    ----------------------------------------------------cc------------------gagcccaaagatgttcacctttaagaaatccctgttactccttttctttcttgggaccatctccttatctctctgt------------------     10 F1S17-P57-TRINITY_DN38944_c0_g1_i1-6.3e-01-odorranain-O4     93.5%  90.8%    ---------------------------------ctg----------atctgcgt------------------gagcccaaagatgttcaccttgaagaaatccctgttactccttttcttccttggaaccatcaacttatctctctgt------------------     35 F1S10-P34-TRINITY_DN6115_c1_g1_i1-2.5e+03-odorranain-A11    100.0%  89.6%    ------------------------cagcaccaactg----------aactaccc------------------gagcccaaagatgttcaccatgaagaaatccctgttactccttttctttcttgggaccatctccttatctctctgt------------------      8 F1S36-P83-TRINITY_DN14764_c0_g1_i2-4.9e+02-odorranain-X5a   100.0%  89.6%    ------------------------cacaaccaactg----------aaccaccc------------------gagcccaaagatgttcaccatgaagaaatccctgttactcctttttttccttgggaccatcaacttatctctctgt------------------     29 F1S8-P23-TRINITY_DN96_c0_g2_i1-3.5e-01-esculentin-2-OG21     71.0%  89.4%    ----------------------------------------------------------------------------------atgttcaccttgaagaaatccttgttactctttttctttcttgggaccatctccttatctctctgt------------------     77 F1S18-P58-TRINITY_DN5345_c0_g1_i2-5.3e+03-odorranain-P1b    100.0%  88.5%    --------------------------gcaccaactg--------ccaagtcctc------------------gagcccaaagatgttcaccttgaagaaatcactgttactccttttcttgcttgggaccatcaacttatctctctgt------------------     52 F1S11-P37-TRINITY_DN56_c1_g1_i1-5.0e+01-odorranain-B7        93.5%  87.4%    ---------------------------------ctg----------aactaccc------------------gagcccaaaaatgttcaccttgaagaaacccctgttactccttttctttcttgggagcgtctccttatctgtctgt------------------     86 F1S23-P68-TRINITY_DN128039_c0_g1_i1-2.6e+03-odorranain-U3    74.2%  87.0%    -------------------------------------------------------------------------------aagatgtgcaccgggaagaaatccctgttactccttttctttcttgtgagcatcgccttatctctctgt------------------     53 F1S11-P38-TRINITY_DN1399_c4_g1_i1-2.5e+01-odorranain-B8      96.8%  86.7%    ------------------------------cagctg----------aactaccc------------------gagcccaaaaatgttcaccttgaagaaacccctgttactccttttctttcttgggatcgtcgccttatctgtctgt------------------     40 F1S12-P42-TRINITY_DN1218_c4_g1_i1-2.4e+00-odorranain-C12     90.3%  86.0%    ----------------------------------tg----------aactaccc--------------------gtccaaagatgttcaccatgcagaaatccctgttactccttttctttcttggggccatctccttatctctctgt------------------     41 F1S12-P41-TRINITY_DN10924_c1_g1_i1-2.4e+00-odorranain-C11    74.2%  85.5%    -------------------------------------------------------------------------------aagatgttcaccatgaagaaatacctgttagtccttttctttcttgggatcgtctccttatctctctgt------------------     60 F1S12-P43-TRINITY_DN2658_c0_g2_i1-3.3e-01-odorranain-C13     74.2%  85.5%    -------------------------------------------------------------------------------aagatgttcaccatgaagaaacccctgttactccctttctttcttcggaccatctccttatctctctgc------------------     74 F1S7-P14-TRINITY_DN4249_c0_g1_i1-2.1e+03-esculentin-1-OG13   93.5%  85.1%    ---------------------------------ctg----------aactaccc------------------gagcccaaagatgttcaccttgaagaaacccctgttactgattgtccttcttgggatcatctccttagctctctgt------------------     81 F1S8-P19-TRINITY_DN2168_c4_g1_i1-6.8e+00-esculentin-2-OG17  100.0%  84.9%    ---------------------------caccaaccg----------aactaccc------------------gagcccaaagatgttaaccatgaagaaatgcatgttagtccttttctttcgtgggaccatctccttgtctctctgt------------------     43 F1S13-P46-TRINITY_DN10285_c0_g1_i1-3.0e+00-odorranain-F2     96.8%  84.8%    ----------------------------accaactg----------atctaccc--------------------gtccaaagatgttcaccatgaagaaatccctgttagtccttttctttcttgggatcgtctccttatctctctgt------------------     80 F1S6-P11-TRINITY_DN6490_c1_g1_i1-8.1e+00-brevinin-2E-OG8     96.8%  84.8%    ----------------------------accaactg----------aactaccc--------------------gtccaaagatgttcaccatgaagaaatccgtgttactccttttctttcttgggacaatatcaatatctctctgt------------------     82 F1S9-P28-TRINITY_DN4414_c6_g1_i1-5.3e+00-nigrocin-OG33      100.0%  84.4%    ------------------------cagcccccactg----------aaatactg------------------gggcccaatgatgttccccttgaagaaatccctgttactccttttcttccttgggaccattaacttatctctctgt------------------     87 F1S9-P29-TRINITY_DN16_c2_g1_i1-5.9e+00-nigrocin-OG34        100.0%  84.4%    ------------------------cagaaccaactg----------aaccacca------------------gagcccacagatgttatcgttgaagaaatccctgttacaccttttcttccttgggaccatcaacttatccctctgt------------------     54 F1S11-P36-TRINITY_DN79_c1_g3_i1-3.4e+03-odorranain-B6       100.0%  82.8%    ---------------------tctccgcaccaactg----------aactaccc------------------gagcccaaagatgttcaccttgcagaaacccctgttactccttttctttcttgggatcgtctccttatctttctgt------------------     88 F1S12-P44-TRINITY_DN2213_c1_g1_i1-5.8e+00-odorranain-C14     93.5%  82.8%    ---------------------------------atg----------aactaacc------------------gagcccaaagatgttcacctttaggaaatcccgggtactccttctggttcttgggaccatctccttatctctctgt------------------     89 F1S12-P45-TRINITY_DN2213_c1_g1_i2-2.4e+00-odorranain-C15     93.5%  82.8%    ---------------------------------atg----------aactaacc------------------gagcccaaagatgttcacctttaggaaatcccgggtactccttctggttcttgggaccatctccttatctctctgt------------------     83 F1S18-P59-TRINITY_DN38049_c0_g1_i1-9.8e+01-odorranain-P1i   100.0%  81.8%    -----------------------ccagcaccaactg--------ccaagttctc------------------gacccccacgatgttccccttgaagacatctctgttactccttttcttccttcggaccatcaacttatctctctgt------------------     64 F1S16-P55-TRINITY_DN3181_c1_g1_i1-3.5e+02-odorranain-M4      71.0%  81.8%    -------------------------------------------------------------------------agcccaaagatgttcaccttgaagaaattcctattgctccttttctttcttgggatcgtctcctca---------------------------     75 F1S7-P15-TRINITY_DN12856_c2_g1_i1-2.7e-01-esculentin-1-OG14  93.5%  80.5%    ---------------------------------cgg----------gactactc------------------gagcccaaagatgttcaccttgaagaaacccctgatactgattgtccttcttgggatcatctccttatccctctgt------------------     58 F1S34-P81-TRINITY_DN17503_c0_g1_i1-1.2e+00-odorranain-X3a   100.0%  80.4%    ------------------cattctcatcaccaactg----------aactaccc------------------gatccagaagatgttcaccttgaagaaatccatgttactccttttctttcttggagccatctcattatctctctgt------------------      2 F1S14-P50-TRINITY_DN603_c2_g1_i1-5.0e+02-odorranain-G1      100.0%  80.2%    ---------ttcttctgcatttctcagtaccaactg----------aactacct------------------gaacccaaagatgctcaccttgaagaaatccctgttactccttttcttccttgcgaccatcaacttatctctctgt------------------      4 F1S9-P26-TRINITY_DN0_c1_g1_i2-2.2e+04-nigrocin-2GRc         100.0%  80.2%    ---------agctgtccacattctcatcaccaactg----------aactaccc------------------gatcccgaagatgttcaccttgaagaaatccctgttactccttttcttccttgggaccatcaacttatctctctgt------------------     13 F1S19-P61-TRINITY_DN4628_c1_g1_i1-1.2e+00-odorranain-P2d    100.0%  80.0%    ---------------ctacattctcagcaccaactg----------aaccaccc------------------gagcccaaagatgttcaccaggaagaaatccctgttactcctttttttccttgggaccatcgacttatgtctctgt------------------     50 F1S32-P79-TRINITY_DN13210_c0_g1_i1-9.0e+00-odorranain-X1a   100.0%  80.0%    ---------------ctacattctcagcaccaactg----------aactaccc------------------gagcccaaagatgttcaccttgaagaaatccctagtactccttttctttcttgggaccatctccttaactctctgt------------------      5 F1S9-P25-TRINITY_DN49_c0_g1_i1-5.8e+03-nigrocin-2GRb        100.0%  79.3%    ---------agctgtttacattctcagcaccaactg----------aactacct------------------gagcccaaagatgttcaccttgaagaaatccctgttactccttttctttcttgggaccatcaacttatctctctgt------------------      6 F1S5-P7-TRINITY_DN23816_c1_g1_i1-4.5e+02-brevinin-1E-OG9    100.0%  79.3%    ---------agctgtctacattctcagcaccaactg----------aactaccc------------------gagcccaaagatgttcaccatgaagaaatccctgttactcctttttttccttgggaccatcaacttatctctctgt------------------     15 F1S2-P2-TRINITY_DN142_c0_g1_i5-5.0e+01-andersonin-R         100.0%  79.0%    ---------------ctacattctcagcaccaactg----------aactaccc------------------gagctcaaagatgttcaccttgaagaaatccctgttactccttttcttcattggaatgatctccttatctctctgt------------------     12 F1S17-P56-TRINITY_DN122946_c2_g1_i1-1.8e+03-odorranain-O1   100.0%  78.4%    ---------agctgtctacaatctcagcaccaactg----------aactatcc------------------gagcgcaaagatgttcaccttgaagaaatccctgttactccttttcttccttggaaccatcaacttatctctctgt------------------     26 F1S27-P74-TRINITY_DN139_c0_g1_i1-3.1e+02-OGC-RA3            100.0%  78.4%    ---------agctgtctacattctcaccaccaactg----------aactaccc------------------gagcccaaagatgttcaccttgaagaaatccctgttactccttttctttcttgggaccatctccttatctctctgt------------------     67 F1S23-P67-TRINITY_DN12170_c0_g1_i1-1.1e+00-odorranain-U2     64.5%  78.3%    -------------------------------------------------------------------------------aagatgttcacctttaagaaattcctattgctccttttctttcttgggatcgcctcctca---------------------------     61 F1S15-P51-TRINITY_DN45_c1_g1_i1-3.1e+03-odorranain-L2        90.3%  77.8%    ----------------------------------------agagataaggagat------------------ggtcccaaagatgttcaccatgacgaaatccctgttactccttttctttcttgggaccatctccttatctctctgt------------------     19 F1S6-P9-TRINITY_DN0_c1_g1_i4-9.2e-01-brevinin-2GRa          100.0%  77.5%    ---------agctgtccacattctcatcaccaactg----------aactaccc------------------gatcccgaagatgttcaccttgaagaaatccctgttactccttttctttcttgggaccatctccttatctctctgt------------------     20 F1S6-P9-TRINITY_DN0_c1_g1_i14-1.3e+04-brevinin-2GRa         100.0%  77.5%    ---------agctgtccacattctcatcaccaactg----------aactaccc------------------gatcccgaagatgttcaccttgaagaaatccctgttactccttttctttcttgggaccatctccttatctctctgt------------------     22 F1S12-P39-TRINITY_DN0_c1_g1_i10-8.0e+03-brevinin-2GRb       100.0%  77.5%    ---------agctgtccacattctcatcaccaactg----------aactaccc------------------gatcccgaagatgttcaccttgaagaaatccctgttactccttttctttcttgggaccatctccttatctctctgt------------------     23 F1S12-P39-TRINITY_DN0_c1_g1_i11-1.7e+00-brevinin-2GRb       100.0%  77.5%    ---------agctgtccacattctcatcaccaactg----------aactaccc------------------gatcccgaagatgttcaccttgaagaaatccctgttactccttttctttcttgggaccatctccttatctctctgt------------------     24 F3-P86-TRINITY_DN6_c0_g1_i12-1.0e+03-tachykinin_OG1         100.0%  76.6%    ---------agctgtctacattctcatcaccaactg----------atctaccc------------------gatcccgaagatgttcaccttgaagaaatccctgttactccttttctttcttgggaccatctccttatctctctgt------------------     25 F3-P87-TRINITY_DN6_c0_g1_i6-8.0e+02-ranamargarin            100.0%  76.6%    ---------agctgtctacattctcatcaccaactg----------atctaccc------------------gatcccgaagatgttcaccttgaagaaatccctgttactccttttctttcttgggaccatctccttatctctctgt------------------     37 F1S19-P62-TRINITY_DN638_c0_g1_i2-3.9e+00-odorranain-P2e     100.0%  76.6%    ---------tataggctacattctcagcaccaactg----------aactacct------------------gagcccaaagatgttcaccttgaagaaacccctgttactccttttctttcttgggaccatctccttatctctctgt------------------     44 F1S13-P47-TRINITY_DN1102_c1_g1_i1-1.1e+00-odorranain-F3     100.0%  75.9%    ------------cggctacattctcagcaccaactg----------aactatcc------------------gagcccaaagatgttcaccatgaagaaatccctgttagtccttttctttcttgggatcgtctccttatctctctgt------------------     14 F1S19-P60-TRINITY_DN39_c0_g1_i2-7.2e+00-odorranain-P2c      100.0%  75.7%    ---------attggtctacattcttagttccagctg----------aaccacca------------------gagcccaaagatgttcaccttgaagaaatccctgttactcctgttcttccttgggaccatcaacttatctctctgt------------------     27 F1S24-P69-TRINITY_DN122936_c0_g1_i1-4.3e+02-odorranalectin  100.0%  75.7%    ---------agctgtctacattctcagcaccaacta----------gactaccc------------------gagcccaaagatgttcaccttgaagaaatccctgttactccttttctttcttgggatcatctccttatctctctgt------------------     30 F1S25-P70-TRINITY_DN1048_c0_g1_i1-1.9e+02-odorranaopin      100.0%  75.7%    ---------ggttgtctacattctcagcaccaactg----------aactaccc------------------gagtccaaagatgttcaccttgaagaaatccttgttacttcttttctttcttgggaccatctccttatctctctgt------------------     31 F1S20-P63-TRINITY_DN132_c0_g1_i4-8.5e+02-odorranain-Q1      100.0%  75.7%    ---------ggctatctacattctcagcaccaattg----------aactaccc------------------aagcccaaagatgttcaccttgaagaaatccctgttactccttttctttcttggaaccatctccttatctctctgt------------------     32 F1S26-P71-TRINITY_DN132_c0_g1_i3-1.8e+02-ishikawain-7-EV1   100.0%  75.7%    ---------ggctatctacattctcagcaccaattg----------aactaccc------------------aagcccaaagatgttcaccttgaagaaatccctgttactccttttctttcttggaaccatctccttatctctctgt------------------     36 F1S24-P69-TRINITY_DN106_c6_g1_i1-2.1e+01-odorranalectin     100.0%  75.7%    ---------tgtgatctacattctcagcaccaactg----------aaccaccc------------------gagcccaaagatgttcaccatgaagaaatccctgttactccttttctttcttgggatcatctccttatctctctgt------------------     55 F1S5-P6-TRINITY_DN0_c1_g1_i24-1.9e+03-brevinin-1E-OG3       100.0%  75.7%    ---------agctgtccacattctcatcaccaactg----------aactaccc------------------gatcccgaagatgttcaccttgaagaaatccatgttactccttttctttcttggaaccatctcattatctctctgt------------------     56 F1S7-P12-TRINITY_DN0_c1_g1_i16-1.0e+00-esculentin-1-OG5     100.0%  75.7%    ---------agctgtccacattctcatcaccaactg----------aactaccc------------------gatcccgaagatgttcaccttgaagaaatccatgttactccttttctttcttggaaccatctcattatctctctgt------------------     57 F1S9-P26-TRINITY_DN0_c1_g1_i17-1.5e+04-nigrocin-2GRc        100.0%  75.7%    ---------agctgtccacattctcatcaccaactg----------aactaccc------------------gatcccgaagatgttcaccttgaagaaatccatgttactccttttctttcttggaaccatctcattatctctctgt------------------      9 F1S9-P27-TRINITY_DN9643_c0_g1_i4-2.5e+00-nigrocin-OG32      100.0%  75.7%    ---------agctgtctacattctcagaaccaactg----------aaccaccc------------------gagtccaaagatgttcaccatgaagaaatccctgttactcctttttttccttgggaccatcaacttatctatctgt------------------     79 F1S9-P24-TRINITY_DN1399_c0_g1_i1-4.6e+01-nigrocin-2GRa      100.0%  75.4%    ------------------------aagcaccagctg----------aactaccc------------------gagcacaaagatgttcaccttgaagaaatccctgttcctccttttcttccttgggaccatcaacttatctctctggcaggatgagacaaatgcc     16 F1S12-P40-TRINITY_DN45_c27_g1_i1-7.5e+02-odorranain-C7      100.0%  74.8%    ---------agctgtctacattctcagcaccagctg----------aactaccc------------------gagcccaaagatgttcaccttgaagaaatccctcttactccttttctttattggaaccatctccttatctctctgt------------------     17 F1S29-P76-TRINITY_DN8472_c0_g1_i1-2.5e+03-palustrin-OG2     100.0%  74.8%    ---------agctgtctacattctcagcaccagctg----------aactaccc------------------gagcccaaagatgttcaccttgaagaaatccctcttactccttttctttattggaaccatctccttatctctctgt------------------     33 F1S8-P17-TRINITY_DN96_c0_g2_i2-1.4e+02-esculentin-2-OG8     100.0%  74.8%    ---------agctgtctacattctcagcaccaacca----------aactaccc------------------aagcccaaagatgttcaccttgaagaaatccctgttactccttttctttcttgggaccatatccttatctctctgt------------------     34 F1S8-P22-TRINITY_DN96_c0_g1_i1-6.4e+00-esculentin-2-OG20    100.0%  74.8%    ---------agctgtctacattctcagcaccaacca----------aactaccc------------------aagcccaaagatgttcaccttgaagaaatccctgttactccttttctttcttgggaccatatccttatctctctgt------------------     38 F1S26-P71-TRINITY_DN132_c0_g1_i1-3.5e+02-ishikawain-7-EV1   100.0%  74.8%    ---------ggctatctacattctcagcaccaattg----------aactaccc------------------aagcccaaagatgttcaccttgaagaaaaccctgttaatccttttctttcttgggaccatctccttatctctctgt------------------     39 F1S26-P72-TRINITY_DN132_c0_g1_i5-2.4e+02-OGA1               100.0%  74.8%    ---------ggctatctacattctcagcaccaattg----------aactaccc------------------aagcccaaagatgttcaccttgaagaaaaccctgttaatccttttctttcttgggaccatctccttatctctctgt------------------     28 F1S8-P16-TRINITY_DN96_c0_g1_i2-2.3e+01-esculentin-2-RA1     100.0%  73.9%    ---------agctgtctacattctcagcaccaacca----------aactaccc------------------aagcccaaagatgttcaccttgaagaaatccttgttactctttttctttcttgggaccatctccttatctctctgt------------------     69 F1S9-P24-TRINITY_DN77_c0_g1_i1-7.4e+01-nigrocin-2GRa        100.0%  73.9%    ---------agctgtctacattgtcagcaccaactg----------aaccaccc------------------gagccgaaagatgttcaccttgaagaaatccatgttactcctttgctttcttggaaccatctcattatctctctgt------------------     78 F1S35-P82-TRINITY_DN360_c0_g1_i1-7.9e+02-odorranain-X4a     100.0%  73.9%    ---------------------------caccaactg----------aactacccgattccaaattatccacagagctcaaagatgttcaccttgaagaaatccctattattccttttctttcttgggatcatctccttctctctctgt------------------     42 F1S13-P46-TRINITY_DN6_c27_g1_i1-4.8e+03-odorranain-F2       100.0%  73.0%    ---------agctgtctacattctcagcaccaactg----------aactactc------------------gagtccaaagatgttcaccatgaagaaatccctgttagtccttttctttcttgggatcgtctccttatctctctgt------------------     73 F1S7-P12-TRINITY_DN81_c0_g1_i1-9.5e+03-esculentin-1-OG5     100.0%  73.0%    ---------agctgtctgcattctcagcaccaactg----------aactaccc------------------gagcccaaagatgttcaccttgaagaaacccctgttactgattgtccttcttgggatcatctccttatctctctgt------------------     45 F1S8-P18-TRINITY_DN0_c1_g1_i22-8.3e+03-esculentin-2-OG10     96.8%  72.8%    ------tccagctgtctacattctcaacaccaactg----------aactac---------------------agcccaaagatgttcaccttgaataaatccctgttactccttttctttcttgggaccatctccttatctctctgt------------------     46 F1S12-P39-TRINITY_DN0_c1_g1_i15-2.2e+01-brevinin-2GRb        96.8%  72.8%    ------tccagctgtctacattctcaacaccaactg----------aactac---------------------agcccaaagatgttcaccttgaataaatccctgttactccttttctttcttgggaccatctccttatctctctgt------------------     47 F1S12-P39-TRINITY_DN0_c1_g1_i23-8.4e+00-brevinin-2GRb        96.8%  72.8%    ------tccagctgtctacattctcaacaccaactg----------aactac---------------------agcccaaagatgttcaccttgaataaatccctgttactccttttctttcttgggaccatctccttatctctctgt------------------     48 F1S22-P65-TRINITY_DN98_c53_g1_i1-3.6e+03-odorranain-T1       96.8%  72.8%    ------tccagctgtctacattctcaacaccaactg----------aactac---------------------agcccaaagatgttcaccttgaataaatccctgttactccttttctttcttgggaccatctccttatctctctgt------------------     49 F1S28-P75-TRINITY_DN0_c1_g1_i20-5.5e+03-OGTI                 96.8%  72.8%    ------tccagctgtctacattctcaacaccaactg----------aactac---------------------agcccaaagatgttcaccttgaataaatccctgttactccttttctttcttgggaccatctccttatctctctgt------------------     70 F1S5-P6-TRINITY_DN0_c1_g1_i6-6.1e+02-brevinin-1E-OG3        100.0%  72.1%    ---------agctgtccacattctcatcaccaactg----------aactaccc------------------gatcccgaagatgttcaccttgaagaaacccctgttactgattgtccttcttgggatcatctccttatctctctgt------------------     71 F1S9-P26-TRINITY_DN0_c1_g1_i3-1.2e+00-nigrocin-2GRc         100.0%  72.1%    ---------agctgtccacattctcatcaccaactg----------aactaccc------------------gatcccgaagatgttcaccttgaagaaacccctgttactgattgtccttcttgggatcatctccttatctctctgt------------------     51 F1S11-P35-TRINITY_DN11239_c0_g1_i2-7.5e+03-odorranain-B1    100.0%  71.9%    ---------agctgtctacactctcagcaccaactg----------aactaccc------------------gagcccaaaaatgttcaccttgaagaaacccctgttactccttttctttcttgggatcgtctccttatctgtctgtggt---------------     68 F1S21-P64-TRINITY_DN638_c6_g1_i1-2.4e+02-odorranain-S1      100.0%  71.2%    ---------agctgtctacattctcagcatcaactg----------aactatcc------------------aagcgcaacaatgttcaccttgaagaaatccctgttactccttttctttctgggggccatctccttatctctctgt------------------     72 F1S7-P12-TRINITY_DN0_c1_g1_i18-2.7e+03-esculentin-1-OG5     100.0%  71.2%    ---------agctgtccacattctcatcaccaactg----------aactaccc------------------gatcccgaagatgttcaccttgaagaaacccctgttactgattgtccttcttgggatcatctccctatctctctgt------------------     62 F1S3-P3-TRINITY_DN25_c0_g1_i2-5.2e+02-andersonin-S           90.3%  69.6%    ---------agctgtctacattctaagcaccagctg----------aactaccc------------------gagcccaaagatgttcaccttgaagaaattcctattgctccttttctttcttgggatcgtctcctca---------------------------     63 F1S16-P54-TRINITY_DN25_c0_g1_i3-2.7e+03-odorranain-M3        90.3%  69.6%    ---------agctgtctacattctaagcaccagctg----------aactaccc------------------gagcccaaagatgttcaccttgaagaaattcctattgctccttttctttcttgggatcgtctcctca---------------------------     65 F1S33-P80-TRINITY_DN1399_c2_g1_i1-7.7e+00-odorranain-X2a     90.3%  69.6%    ---------agctgtctacattctaagcaccagctg----------aactaccc------------------gagcccaaagatgttcaccttgaagaaattcctattgctccttttctttcttgggatcgtctcctca---------------------------     66 F1S16-P53-TRINITY_DN25_c0_g1_i1-2.1e+03-odorranain-M2        90.3%  69.6%    ---------agctgtctacattctaagcaccagctg----------aactaccc------------------gagcccaaagatgttcaccttgaagaaattcctgttgctccttttctttcttgggattgtctcctca---------------------------     85 F1S10-P33-TRINITY_DN25595_c0_g1_i1-3.1e+00-odorranain-A10   100.0%  69.4%    -------------------actgtggcaacaaggcgctttattgtctactatcc------------------gagcccaaagatgttcaccatgacgaaatccctgttactgcttttctttcttgggaccatctccttatctctctgt------------------     84 F1S30-P77-TRINITY_DN0_c174_g2_i1-9.7e+03-pleurain-E-OG1     100.0%  67.5%    ------------tgtctacattctcagcaccaaccg----------aactaccc------------------gagcccaaagatgttatccttgaagacatccctgttactccttttctttattgggattgtctcctcatctccctgtcgagga------------     91 F1S28-P75-TRINITY_DN603_c0_g1_i3-5.1e-01-OGTI                93.5%  66.7%    ---------------------------------ctg----------cactacaa------------------gagcgataaaatgttcgcaatgaagaaatcactgttcgtccgattgtgtgttggggggatccaattatctctctggggt---------------     59 F1S10-P31-TRINITY_DN7347_c0_g1_i1-4.9e+03-odorranain-A8      90.3%  65.8%    aagttctccagctgtcaacattctcatcaccaactg----------aactaccc---------------------------gatgttcaccttgaagaaatccctgttactccttttctttcttgggaccatctccttatctctctgt------------------     76 F1S7-P13-TRINITY_DN259_c0_g1_i1-1.7e+02-esculentin-1-OG12   100.0%  64.9%    ---------agctgtgtacatcccgggcgccgccgg----------aactaccc------------------aagaccaaagatgttcaccttgaagaaacccctgttactgattgtccttcttgggatcatctccttatcccaatgt------------------     90 F1S28-P75-TRINITY_DN603_c0_g1_i1-4.5e+01-OGTI               100.0%  56.6%    ---------agctgtctacattctcagcaccagctg----------aactaccc------------------gagcccaaagatgttcaccatgaagaaatccatgttactcctattatttgttggggtgatcttcgggtccctctgggaggaacatagagatgcc        clustal                                                                                                                                                                                                                                                    consensus/75%                                                                .............................CCA.CTG..........AACTACC....................A.CCC.AAGATGTTCACCTTGAAGAAATCCCTGTTACTCCTTTTCTTTCTTGGGA.CATCTCCTTATCTCTCTGT.................. ``` |

MView 1.67, Copyright © 1997-2020 Nigel P. Brown
