# Supplementary material for: Integrative transcriptomics and peptidomics approach reveals unexpectedly diverse endogenous secretory peptides in Odorrana grahami frog skin
Source: BMC Biol. 2025 Nov 28;23:354. doi: 10.1186/s12915-025-02463-w (PMC12664280; doi:10.1186/s12915-025-02463-w)
Supplement: Supplementary file 5 — Additional file 5. Alignments of ESP sequences identified in this study across different regions. [file 12915_2025_2463_MOESM5_ESM.zip › Additional file 5/FSAP family - stop codon plus down to 45 nucleotides downstream of the 3’-UTR..html]

MView


|  |
| --- |
| ``` Reference sequence (1): F1S1-P1-TRINITY_DN175_c1_g1_i1-9.3e+02-andersonin-Q Identities normalised by aligned length. Colored by: consensus group/60% ``` |
| ```                                                                   cov    pid  1 [        .         .         .         .         :         .         .         .         .         1         .     ] 116  1 F1S1-P1-TRINITY_DN175_c1_g1_i1-9.3e+02-andersonin-Q         100.0% 100.0%    -------------------------tgaaacttgaat---------ttt-aaatcatctgat----------gtggaatatcatttagctaaa-----------------------     10 F1S14-P50-TRINITY_DN603_c2_g1_i1-5.0e+02-odorranain-G1      100.0%  95.8%    -------------------------tgaaacttgaat---------tgg-aaatcatctgat----------gtggaatatcatttagctaaa-----------------------     11 F1S11-P35-TRINITY_DN11239_c0_g1_i2-7.5e+03-odorranain-B1    100.0%  89.6%    -------------------------taaaacttgaaa---------tgg-aaaccatctgat----------gtggaatatcatttagctaaa-----------------------     12 F1S11-P36-TRINITY_DN79_c1_g3_i1-3.4e+03-odorranain-B6       100.0%  89.6%    -------------------------taaaacttgaaa---------tgg-aagtcatctgat----------gtggaatatcatttagctaaa-----------------------     20 F1S6-P9-TRINITY_DN0_c1_g1_i14-1.3e+04-brevinin-2GRa         100.0%  89.6%    -------------------------taaaacatgaat---------tgg-aagtcatctgat----------gtggaatatcatttagctaaa-----------------------     21 F1S8-P18-TRINITY_DN0_c1_g1_i22-8.3e+03-esculentin-2-OG10    100.0%  89.6%    -------------------------taaaacatgaat---------tgg-aagtcatctgat----------gtggaatatcatttagctaaa-----------------------     22 F1S8-P16-TRINITY_DN96_c0_g1_i2-2.3e+01-esculentin-2-RA1     100.0%  89.6%    -------------------------taaaacatgaat---------tgg-aaatcatctgat----------gtggaatatcatttagctaag-----------------------     23 F1S8-P17-TRINITY_DN96_c0_g2_i2-1.4e+02-esculentin-2-OG8     100.0%  89.6%    -------------------------taaaacatgaat---------tgg-aaatcatctgat----------gtggaatatcatttagctaag-----------------------     24 F1S8-P22-TRINITY_DN96_c0_g1_i1-6.4e+00-esculentin-2-OG20    100.0%  89.6%    -------------------------taaaacatgaat---------tgg-aaatcatctgat----------gtggaatatcatttagctaag-----------------------     25 F1S8-P23-TRINITY_DN96_c0_g2_i1-3.5e-01-esculentin-2-OG21    100.0%  89.6%    -------------------------taaaacatgaat---------tgg-aaatcatctgat----------gtggaatatcatttagctaag-----------------------     49 F1S9-P30-TRINITY_DN11504_c0_g1_i1-4.8e+00-nigrocin-OG35     100.0%  89.6%    -------------------------taaaacttctat---------ctt-aaatcatctgat----------gtggaatatcatgtagctaaa-----------------------      5 F1S9-P26-TRINITY_DN0_c1_g1_i17-1.5e+04-nigrocin-2GRc        100.0%  89.6%    -------------------------taaagcttgaat---------cgg-aaatcatctgat----------gtggaatatcatttagctaaa-----------------------      6 F1S9-P26-TRINITY_DN0_c1_g1_i3-1.2e+00-nigrocin-2GRc         100.0%  89.6%    -------------------------taaagcttgaat---------cgg-aaatcatctgat----------gtggaatatcatttagctaaa-----------------------      7 F1S9-P26-TRINITY_DN0_c1_g1_i2-2.2e+04-nigrocin-2GRc         100.0%  89.6%    -------------------------taaagcttgaat---------cgg-aaatcatctgat----------gtggaatatcatttagctaaa-----------------------     26 F1S13-P46-TRINITY_DN6_c27_g1_i1-4.8e+03-odorranain-F2       100.0%  87.5%    -------------------------taaaacgtgaat---------tgg-aagtcatctgat----------gtggaatatcgtttagctaaa-----------------------     31 F1S11-P38-TRINITY_DN1399_c4_g1_i1-2.5e+01-odorranain-B8     100.0%  87.5%    -------------------------taaaacttgaaa---------tgg-aaatcagctgat----------gttgaatatcatttagctaaa-----------------------      4 F1S9-P25-TRINITY_DN49_c0_g1_i1-5.8e+03-nigrocin-2GRb        100.0%  87.5%    -------------------------taaagcttgaat---------cgg-aaatcatttgat----------gtggaatatcatttagctaaa-----------------------      8 F1S9-P27-TRINITY_DN9643_c0_g1_i4-2.5e+00-nigrocin-OG32      100.0%  87.5%    -------------------------taaagcttgaat---------cgg-aaatcatctgat----------gtgtaatatcatttagctaaa-----------------------      9 F1S9-P29-TRINITY_DN16_c2_g1_i1-5.9e+00-nigrocin-OG34        100.0%  87.5%    -------------------------taaagcttgaag---------cgg-aaatcatctgat----------gtggaatatcatttagctaaa-----------------------     19 F1S12-P40-TRINITY_DN45_c27_g1_i1-7.5e+02-odorranain-C7       95.8%  87.0%    -------------------------taaaacatgaat---------tgg-aagtcatttgat----------gtggaatatcatttagcta-------------------------     13 F1S6-P9-TRINITY_DN0_c1_g1_i4-9.2e-01-brevinin-2GRa          100.0%  85.4%    -------------------------taaaacatgaat---------tgg-aagtcatttgat----------gtggaatatcatttagcgaaa-----------------------     14 F1S12-P39-TRINITY_DN0_c1_g1_i15-2.2e+01-brevinin-2GRb       100.0%  85.4%    -------------------------taaaacatgaat---------tgg-aagtcatttgat----------gtggaatatcatttagcgaaa-----------------------     15 F1S12-P39-TRINITY_DN0_c1_g1_i10-8.0e+03-brevinin-2GRb       100.0%  85.4%    -------------------------taaaacatgaat---------tgg-aagtcatttgat----------gtggaatatcatttagcgaaa-----------------------     43 F1S7-P12-TRINITY_DN81_c0_g1_i1-9.5e+03-esculentin-1-OG5     100.0%  85.4%    -------------------------taaaacctgaat---------tga-aagtcatctgat----------gtggaataaaatttagctaaa-----------------------     44 F1S7-P12-TRINITY_DN0_c1_g1_i16-1.0e+00-esculentin-1-OG5     100.0%  85.4%    -------------------------taaaacctgaat---------tga-aagtcatctgat----------gtggaataaaatttagctaaa-----------------------     45 F1S7-P12-TRINITY_DN0_c1_g1_i18-2.7e+03-esculentin-1-OG5     100.0%  85.4%    -------------------------taaaacctgaat---------tga-aagtcatctgat----------gtggaataaaatttagctaaa-----------------------     18 F1S12-P41-TRINITY_DN10924_c1_g1_i1-2.4e+00-odorranain-C11   100.0%  83.3%    -------------------------taaaacatgaat---------tgg-aagtcatttgat----------gtagaatatcatttagcgaaa-----------------------     29 F1S12-P44-TRINITY_DN2213_c1_g1_i1-5.8e+00-odorranain-C14    100.0%  83.3%    -------------------------taaaacatgaac---------ttg-aagtcatttgct----------gtggaatatcatttagcgaaa-----------------------     30 F1S13-P46-TRINITY_DN10285_c0_g1_i1-3.0e+00-odorranain-F2    100.0%  83.3%    -------------------------taaaacgtgaat---------tgg-tagtcatctgat----------gtggaatatcgtttaggtaaa-----------------------     35 F1S13-P47-TRINITY_DN1102_c1_g1_i1-1.1e+00-odorranain-F3     100.0%  83.3%    -------------------------taaatcgtgaat---------tgg-aagtcatctgat----------gtggaatatcgtgtagctaaa-----------------------     46 F1S7-P13-TRINITY_DN259_c0_g1_i1-1.7e+02-esculentin-1-OG12   100.0%  83.3%    -------------------------taaaacctgaat---------tga-aattcatctgat----------gtggaataaaattcagctaaa-----------------------     57 F1S18-P58-TRINITY_DN5345_c0_g1_i2-5.3e+03-odorranain-P1b     97.9%  83.3%    --------------------------taaaagtgaat---------tgg-aaatcatccgat----------gtggaaaatcatttagctaaat----------------------     39 F1S21-P64-TRINITY_DN638_c6_g1_i1-2.4e+02-odorranain-S1       95.8%  83.3%    ------------------------tgaaatcttgagt---------tggaaaatcatctgat----------gtggaatatcatttagcta-------------------------     59 F1S19-P61-TRINITY_DN4628_c1_g1_i1-1.2e+00-odorranain-P2d     47.9%  82.6%    -----------------------------------------------------taatcagat----------atggaataacattt------------------------------     27 F1S12-P42-TRINITY_DN1218_c4_g1_i1-2.4e+00-odorranain-C12    100.0%  81.2%    -------------------------taaaacataaat---------tgg-aagtcatttgct----------gtggaatatcatttagcgaaa-----------------------     28 F1S12-P43-TRINITY_DN2658_c0_g2_i1-3.3e-01-odorranain-C13    100.0%  81.2%    -------------------------taaaacatgaat---------tgg-gagtcatttgat----------gtggaatatcatttatcgaaa-----------------------     48 F1S9-P28-TRINITY_DN4414_c6_g1_i1-5.3e+00-nigrocin-OG33      100.0%  81.2%    -------------------------tgaagcttggat---------cgg-aaatcaggtgat----------gtggaatatcatgtagctaga-----------------------     36 F1S30-P77-TRINITY_DN0_c174_g2_i1-9.7e+03-pleurain-E-OG1      62.5%  80.0%    --------------------------taatcctgaat---------tgg-aagtcatctgat----------gtgg----------------------------------------     50 F1S4-P4-TRINITY_DN836_c0_g1_i2-1.3e+01-andersonin-X-OG1     100.0%  79.2%    -------------------------taaatattgagt---------agg-aagtcatctgat----------gtgaaatattatttagctaaa-----------------------     51 F1S20-P63-TRINITY_DN132_c0_g1_i4-8.5e+02-odorranain-Q1      100.0%  79.2%    -------------------------taaatattgagt---------agg-aagtcatctgat----------gtgaaatattatttagctaaa-----------------------     52 F1S35-P82-TRINITY_DN360_c0_g1_i1-7.9e+02-odorranain-X4a     100.0%  79.2%    -------------------------taaatcaagatc---------agg-aagtcatctgat----------gtggaatatcatttagctaaa-----------------------     42 F1S16-P55-TRINITY_DN3181_c1_g1_i1-3.5e+02-odorranain-M4      97.9%  79.2%    --------------------------taattttgaat---------tgg-aggtaatctgat----------gtggaatattatttagctaaat----------------------     32 F1S10-P31-TRINITY_DN7347_c0_g1_i1-4.9e+03-odorranain-A8      89.6%  79.2%    --------------------taagataaatcttgaat---------tgg-aaatcatctgat----------gtggaatattatttag----------------------------     33 F1S10-P32-TRINITY_DN25_c1_g1_i1-7.6e+03-odorranain-A9        89.6%  79.2%    --------------------taagataaatcttgaat---------tgg-aaatcatctgat----------gtggaatattatttag----------------------------     41 F1S16-P54-TRINITY_DN25_c0_g1_i3-2.7e+03-odorranain-M3        89.6%  79.1%    --------------------------taattttgaat---------tgg-aggtaatctgat----------gtggaatattatttagc---------------------------     61 F1S6-P11-TRINITY_DN6490_c1_g1_i1-8.1e+00-brevinin-2E-OG8     47.9%  78.3%    -------------------------taaaacatgaag---------tgt-aagtcatc----------------------------------------------------------     16 F1S12-P39-TRINITY_DN0_c1_g1_i23-8.4e+00-brevinin-2GRb        45.8%  77.3%    -------------------------taaaacatgaat---------tgg-aagtcat-----------------------------------------------------------     17 F1S12-P39-TRINITY_DN0_c1_g1_i11-1.7e+00-brevinin-2GRb        45.8%  77.3%    -------------------------taaaacatgaat---------tgg-aagtcat-----------------------------------------------------------     34 F1S10-P34-TRINITY_DN6115_c1_g1_i1-2.5e+03-odorranain-A11     89.6%  77.1%    --------------------taagataaatcttgaat---------tgg-aaatcatctgat----------gtcgaatattatttag----------------------------     53 F1S15-P51-TRINITY_DN45_c1_g1_i1-3.1e+03-odorranain-L2        85.4%  77.1%    --------------------------------tgatt---------tgg-aagtcatctgat----------gtggaatatcatttagctaaatgcttaa----------------      3 F1S9-P24-TRINITY_DN1399_c0_g1_i1-4.6e+01-nigrocin-2GRa       27.1%  76.9%    -------------------------taaagcttgaat---------c---------------------------------------------------------------------     60 F1S12-P45-TRINITY_DN2213_c1_g1_i2-2.4e+00-odorranain-C15    100.0%  75.0%    -------------------------taaaacatgaat---------tgg-acgtcatttcat----------ctggactatcatttagctcca-----------------------     58 F1S18-P59-TRINITY_DN38049_c0_g1_i1-9.8e+01-odorranain-P1i    97.9%  75.0%    --------------------------taacatagact---------tgg-aaatcctccgat----------gtggaaaatcatttagctcaat----------------------     54 F1S17-P56-TRINITY_DN122946_c2_g1_i1-1.8e+03-odorranain-O1    91.7%  75.0%    -----------------------------taatctac---------tgg-aaatcatctgat----------gtggaatatcatttagctaaatgca-------------------     55 F1S17-P57-TRINITY_DN38944_c0_g1_i1-6.3e-01-odorranain-O4     91.7%  75.0%    -----------------------------taatctac---------tgg-aaatcatctgat----------gtggaatatcatttagctaaatgca-------------------     37 F1S22-P65-TRINITY_DN98_c53_g1_i1-3.6e+03-odorranain-T1       89.6%  75.0%    --------------------taagatcaatcttgaat---------tgg-aggtcatctgat----------gtgaaatatcatttag----------------------------      2 F1S9-P24-TRINITY_DN77_c0_g1_i1-7.4e+01-nigrocin-2GRa         16.7%  75.0%    -------------------------taaagctt-----------------------------------------------------------------------------------     47 F1S7-P15-TRINITY_DN12856_c2_g1_i1-2.7e-01-esculentin-1-OG14  64.6%  74.2%    ----------------------------------------------tga-aagtcaactgat----------gtggaatcaaaattag----------------------------     38 F1S10-P33-TRINITY_DN25595_c0_g1_i1-3.1e+00-odorranain-A10    89.6%  72.9%    --------------------taagataaatcttgaat---------tgg-aaataatctgct----------gtggaatattctttag----------------------------     66 F1S5-P6-TRINITY_DN0_c1_g1_i24-1.9e+03-brevinin-1E-OG3        83.3%  70.8%    -----------------tgaaactttggcaatggaat---------tgg-aaatcatctgat----------gtggaatatcatt-------------------------------     67 F1S5-P6-TRINITY_DN0_c1_g1_i6-6.1e+02-brevinin-1E-OG3         83.3%  70.8%    -----------------tgaaactttggcaatggaat---------tgg-aaatcatctgat----------gtggaatatcatt-------------------------------     68 F1S5-P7-TRINITY_DN23816_c1_g1_i1-4.5e+02-brevinin-1E-OG9     83.3%  70.8%    -----------------tgaaactttggcaatggaat---------tgt-aaatcatcttat----------gtggaatatcatt-------------------------------     56 F1S16-P53-TRINITY_DN25_c0_g1_i1-2.1e+03-odorranain-M2        85.4%  68.8%    --------------------------------tgaat---------tgg-aagtaatctgat----------gtgaaatattgtttagcttaatggtaaa----------------     69 F1S5-P8-TRINITY_DN33233_c1_g1_i1-1.1e+02-brevinin-1E-OG10    83.3%  66.7%    -----------------tgaaactttggcaatggaat---------tgg-aaagcagctgat----------gtggaatatcatt-------------------------------     40 F1S3-P3-TRINITY_DN25_c0_g1_i2-5.2e+02-andersonin-S           83.3%  64.6%    -----------------tgaaaaggttaattttgaat---------tgg-aggtaatctgat----------gtggaatattatt-------------------------------     73 F1S24-P69-TRINITY_DN122936_c0_g1_i1-4.3e+02-odorranalectin   89.6%  62.5%    --------------------taagatacattttgaat---------ggg-aattcatcgcat----------ttaaaatatcatttag----------------------------     77 F1S2-P2-TRINITY_DN142_c0_g1_i5-5.0e+01-andersonin-R          85.4%  60.4%    --------------------------------taatc---------tta-aagatatcagtt----------tggaaatatcatttagctaaatgcacaa----------------     71 F1S28-P75-TRINITY_DN603_c0_g1_i1-4.5e+01-OGTI                56.2%  56.7%    -------------------------taaaactggaat---------tgg-aagctaattgctaac---------------------------------------------------     72 F1S28-P75-TRINITY_DN603_c0_g1_i3-5.1e-01-OGTI                56.2%  56.7%    -------------------------taaaactggaat---------tgg-aagctaattgctaac---------------------------------------------------     74 F1S24-P69-TRINITY_DN106_c6_g1_i1-2.1e+01-odorranalectin      89.6%  56.2%    --------------------taagatacattttgaat---------ggg-aattcatcgcgt----------tgaaaatatcagttag----------------------------     65 F1S26-P72-TRINITY_DN132_c0_g1_i5-2.4e+02-OGA1                62.5%  52.1%    -------tgattagaaaaaacatattgaaacatgaat---------tac-gaatcatctggt----------gtg-----------------------------------------     62 F1S25-P70-TRINITY_DN1048_c0_g1_i1-1.9e+02-odorranaopin       60.4%  50.0%    ------tgaatattttaaaaaccaataaaacatgaat---------tgg-aagtcatctgat----------gt------------------------------------------     76 F1S23-P68-TRINITY_DN128039_c0_g1_i1-2.6e+03-odorranain-U3    72.9%  48.2%    ------------taaaaaaaattactggaacttgaac---------tgg-aagtc------------------cggaatattattcag----------------------------     70 F1S28-P75-TRINITY_DN0_c1_g1_i20-5.5e+03-OGTI                 79.2%  45.8%    -------------------------taaaactggaat---------tgg-aagctaattgctaaatgtctaatcaaaataaaa---------------------------------     63 F1S26-P71-TRINITY_DN132_c0_g1_i3-1.8e+02-ishikawain-7-EV1    56.2%  45.8%    tg----ataattagacaaaatgtgttgaaacatgaat---------tac-gaatcatctggt------------------------------------------------------     64 F1S26-P71-TRINITY_DN132_c0_g1_i1-3.5e+02-ishikawain-7-EV1    56.2%  45.8%    tg----ataattagacaaaatgtgttgaaacatgaat---------tac-gaatcatctggt------------------------------------------------------     75 F1S23-P67-TRINITY_DN12170_c0_g1_i1-1.1e+00-odorranain-U2     72.9%  44.6%    ------------taaaaaaaattactggcacttgaac---------tgg-aagcc------------------cggaatattattcag----------------------------     84 F1S29-P76-TRINITY_DN8472_c0_g1_i1-2.5e+03-palustrin-OG2      70.8%  41.7%    --------------------------------tgaattagctaa--acg-ttaatgtcttattata---taataaaaatatcatat------------------------------     82 F1S19-P62-TRINITY_DN638_c0_g1_i2-3.9e+00-odorranain-P2e      75.0%  39.6%    ----------------------------------------------tga-taaatgtctgataaat---atatatatatatcatataaactattttca------------------     78 F1S8-P19-TRINITY_DN2168_c4_g1_i1-6.8e+00-esculentin-2-OG17   85.4%  37.5%    ------------------taagcaggcaagactgggc---------ttg-aacttatggctt----------gtaaaattaccaac------------------------------     87 F1S34-P81-TRINITY_DN17503_c0_g1_i1-1.2e+00-odorranain-X3a    77.1%  37.5%    ----------------taaattatgcagcacgtgtat---------tct-gcagcatccaaa--------aaatgtgacaat----------------------------------     83 F1S27-P74-TRINITY_DN139_c0_g1_i1-3.1e+02-OGC-RA3             75.0%  37.5%    ----------------------------------------------tag-taaatgtcttaaaaa-----aattaaaatatcacacgcaaaaaaaaaaaa----------------     86 F1S11-P37-TRINITY_DN56_c1_g1_i1-5.0e+01-odorranain-B7        52.1%  35.4%    ----------------------------------------------------------tgat----------ttgcaaaatcatccaactcgacatggatccgcaccatcattacg     85 F1S32-P79-TRINITY_DN13210_c0_g1_i1-9.0e+00-odorranain-X1a    79.2%  33.3%    --------------------------------taaaa-------------agttgactaagt----------ttaaaaagtaacatttctgaatgttaaaact-------------     88 F1S7-P14-TRINITY_DN4249_c0_g1_i1-2.1e+03-esculentin-1-OG13   79.2%  33.3%    --------------------------------tgaatca-----------aaggggtcaagcacat------aggcaaggaagttgggatggatgtg-------------------     91 F1S36-P83-TRINITY_DN14764_c0_g1_i2-4.9e+02-odorranain-X5a    68.8%  33.3%    --------------------------------------------------tgaggatcacat----------gtcccatacagttttgttgggcctcactatgtggat--------     81 F1S5-P5-TRINITY_DN23413_c1_g1_i1-1.3e+00-gaegurin-6-OG1      75.0%  31.2%    ----------------------------------------------tga-aacctaaccaaaaaat------gttgaaacttgtgcaagcccatagtaatg---------------     79 F3-P86-TRINITY_DN6_c0_g1_i12-1.0e+03-tachykinin_OG1          47.9%  31.2%    tgaaactactttgtggaaaagcagcccagtcttgaat---------tgg-aagccatc----------------------------------------------------------     80 F3-P87-TRINITY_DN6_c0_g1_i6-8.0e+02-ranamargarin             47.9%  31.2%    tgaaactactttgtggaaaagcagcccagtcttgaat---------tgg-aagccatc----------------------------------------------------------     89 F1S19-P60-TRINITY_DN39_c0_g1_i2-7.2e+00-odorranain-P2c       85.4%  25.0%    --------------------------------tgagcta-------cga-acagcgccgagga---------tggggccgaggccgaggtggccgtg-------------------     90 F1S33-P80-TRINITY_DN1399_c2_g1_i1-7.7e+00-odorranain-X2a     72.9%  25.0%    ---------------------taagcaccagctgaactacccgagccca-aagatgttcacc----------ttgaagaa------------------------------------        clustal                                                                                                                                                                                                  consensus/75%                                                                ................................TGAA...........G..AA.TCAT....T...........T..AA.A.................................... ``` |

MView 1.67, Copyright © 1997-2020 Nigel P. Brown
