# Supplementary material for: Integrative transcriptomics and peptidomics approach reveals unexpectedly diverse endogenous secretory peptides in Odorrana grahami frog skin
Source: BMC Biol. 2025 Nov 28;23:354. doi: 10.1186/s12915-025-02463-w (PMC12664280; doi:10.1186/s12915-025-02463-w)
Supplement: Supplementary file 5 — Additional file 5. Alignments of ESP sequences identified in this study across different regions. [file 12915_2025_2463_MOESM5_ESM.zip › Additional file 5/readme.pdf]

Alignment of corresponding sequences of endogenous secretory peptides (ESPs) identified in this study for different regions

1. For annotations of taxa names, see Additional file 2: Fig. S3
2. The **All 14 families - signal peptide plus up to 45 nucleotides upstream of the 5 -UTR.html** file includes the alignment results for the signal peptide and upstream 5’-UTR region up to 45 nucleotides across all 14 peptide families. The corresponding alignment results of "translated" amino acid sequences are contained within the file **All 14 families - signal peptide plus up to 45 nucleotides upstream of the 5 -UTR "translated".html**. The similarity between F1S28-P75-TRINITY_DN603_c0_g1_i1-4.5e+01-OGTI and F14-P102-TRINITY_DN17623_c0_g1_i1-3.4e-01-C-X-C_motif_chemokine_8-OG1 drops from 56.6% to 36.8% for nucleic acids, and from 43.3% to 23.7% for the "translated" amino acid sequences, at a breakpoint. This breakpoint distinguishes the FSAP family (F1) peptides from the peptides of other families (F2-F14).
3. The alignment results of the regions of the corresponding sequences of the FSAP family peptides are shown in the file name.
4. **The FSAP family - Trimal.fasta file**: Trimmed sequences for phylogenetic analysis.
