# Supplementary material for: Integrative transcriptomics and peptidomics approach reveals unexpectedly diverse endogenous secretory peptides in Odorrana grahami frog skin
Source: BMC Biol. 2025 Nov 28;23:354. doi: 10.1186/s12915-025-02463-w (PMC12664280; doi:10.1186/s12915-025-02463-w)
Supplement: Supplementary file 3 — Additional file 3. Exported and processed results derived from the native Proteome Discoverer result files (*.pdResult; Data S2 of [44]). SPS/ – Results of searching against the SPS database. All_ESP_sequences_from_this_species/ – Results of searching against all ESP sequences from this species, including previously reported ones; contains suspected false positives (e.g., esculentin-2-OG1/OG2, odorranain-C3). Annotated Spectra.zip, *_export.xlsx, MSMS Scans and PSMs.xlsx – Files exported from *.pdResult, including annotated MS/MS spectra, protein/peptide isoform identifications, and MS/MS scan metadata. Result.xlsx – Final filtered peptide list obtained after processing by the Peptide.py script. [file 12915_2025_2463_MOESM3_ESM.zip › Additional file 3/SPS/Annotated Spectra.zip!/index.html]

Annotated spectra


## Annotated spectra of D:\OG\_skin\Search\_file.msf

go to Peptides  
go to Search Summary  

### Peptides

|  |  |  |  |  |  |  |  |  |  |  |  |  |  |  |  |  |  |  |  |  |  |  |  |  |  |  |  |  |  |  |  |  |  |  |  |  |  |  |  |  |  |  |  |  |  |  |  |  |  |  |  |  |  |  |
| --- | --- | --- | --- | --- | --- | --- | --- | --- | --- | --- | --- | --- | --- | --- | --- | --- | --- | --- | --- | --- | --- | --- | --- | --- | --- | --- | --- | --- | --- | --- | --- | --- | --- | --- | --- | --- | --- | --- | --- | --- | --- | --- | --- | --- | --- | --- | --- | --- | --- | --- | --- | --- | --- | --- |
|  | | | | | | | | | | | | | | | | | | | | | | | | | | | | | | | | | | | | | | | | | | | | | | | | | | | | | |  |
| Confidence Sequence Activation Type Modifications XCorr Percolator q-Value Percolator PEP Percolator SVMScore ptmRS: Binomial Peptide Score ptmRS: Isoform Confidence Probability ptmRS: Isoform Group Report ptmRS: Best Site Probabilities ptmRS: Oxidation Site Probabilities ptmRS: Deamidated Site Probabilities ptmRS: Dehydro Site Probabilities ΔScore Rank Search Engine Charge Precursor m/z [Da] ΔM [ppm] RT [min] First Scan Last Scan Protein Accessions Annotated Spectrum Peak List  | | | | | | | | | | | | | | | | | | | | | | | | | | | | | | | | | | | | | | | | | | | | | | | | | | | | | | |  |  |  |  |  |  |  |  |  |  |  |  |  |  |  |  |  |  |  |  |  |  |  |  |  |  |  |  |  |  |  |  |  |  |  |  |  |  |  |  |  |  |  |  |  |  |  |  |  |  |  |  |  |  |
|  | | | | | | | | | | | | | | | | | | | | | | | | | | | | | | | | | | | | | | | | | | | | | | | | | | | | | |  |
|  | **TRINITY\_DN0\_c1\_g1\_i11.p1 - TRINITY\_DN0\_c1\_g1\_i11.p1** | | | | | | | | | | | | | | | | | | | | | | | | | | | | | | | | | | | | | | | | | | | | | | | | | | | |  |  |
|  | | | | | | | | | | | | | | | | | | | | | | | | | | | | | | | | | | | | | | | | | | | | | | | | | | | | | |  |
|  | High |  | [GS].KSAAQSVLKTLScKLSNDc.[-] |  | HCD |  | C13(Dehydro); C19(Dehydro) |  | 3.00 |  | 1.3e-3 |  | 4.8e-2 |  | 0.347 |  | 189.80 |  | 1.000 |  |  |  | C13(Dehydro): 100; C19(Dehydro): 100 |  |  |  |  |  |  |  | 0.60 |  | 1 |  | Sequest HT (3) |  | 3 |  | 665.33936 |  | -0.51 |  | 32.3240 |  | 7465 |  | 7465 |  | TRINITY\_DN0\_c1\_g1\_i15.p1; TRINITY\_DN0\_c1\_g1\_i23.p1; TRINITY\_DN0\_c1\_g1\_i11.p1; TRINITY\_DN45\_c27\_g1\_i1.p1; TRINITY\_DN2658\_c0\_g2\_i1.p1\_ORF2; TRINITY\_DN0\_c1\_g1\_i10.p1; TRINITY\_DN2658\_c0\_g2\_i1.p1\_ORF1; TRINITY\_DN2658\_c0\_g2\_i1.p1 |  | Image |  | Peak List |  |
|  | | | | | | | | | | | | | | | | | | | | | | | | | | | | | | | | | | | | | | | | | | | | | | | | | | | | | |  |
|  | High |  | [GS].KSAAQSVLKTLScKLSNDc.[-] |  | HCD |  | C13(Dehydro); C19(Dehydro) |  | 4.38 |  | 1.3e-3 |  | 4.1e-2 |  | 0.382 |  | 219.12 |  | 1.000 |  |  |  | C13(Dehydro): 100; C19(Dehydro): 100 |  |  |  |  |  |  |  | 0.58 |  | 1 |  | Sequest HT (3) |  | 4 |  | 499.25656 |  | -0.05 |  | 32.3418 |  | 7474 |  | 7474 |  | TRINITY\_DN0\_c1\_g1\_i15.p1; TRINITY\_DN0\_c1\_g1\_i23.p1; TRINITY\_DN0\_c1\_g1\_i11.p1; TRINITY\_DN45\_c27\_g1\_i1.p1; TRINITY\_DN2658\_c0\_g2\_i1.p1\_ORF2; TRINITY\_DN0\_c1\_g1\_i10.p1; TRINITY\_DN2658\_c0\_g2\_i1.p1\_ORF1; TRINITY\_DN2658\_c0\_g2\_i1.p1 |  | Image |  | Peak List |  |
|  | | | | | | | | | | | | | | | | | | | | | | | | | | | | | | | | | | | | | | | | | | | | | | | | | | | | | |  |
|  | High |  | [GS].KSAAQSVLKTLScKLSNDc.[-] |  | HCD |  | C13(Dehydro); C19(Dehydro) |  | 1.73 |  | 3.1e-3 |  | 1.1e-1 |  | 0.15 |  | 202.39 |  | 1.000 |  |  |  | C13(Dehydro): 100; C19(Dehydro): 100 |  |  |  |  |  |  |  | 0.46 |  | 1 |  | Sequest HT (3) |  | 2 |  | 997.50824 |  | 2.35 |  | 32.3444 |  | 7475 |  | 7475 |  | TRINITY\_DN0\_c1\_g1\_i15.p1; TRINITY\_DN0\_c1\_g1\_i23.p1; TRINITY\_DN0\_c1\_g1\_i11.p1; TRINITY\_DN45\_c27\_g1\_i1.p1; TRINITY\_DN2658\_c0\_g2\_i1.p1\_ORF2; TRINITY\_DN0\_c1\_g1\_i10.p1; TRINITY\_DN2658\_c0\_g2\_i1.p1\_ORF1; TRINITY\_DN2658\_c0\_g2\_i1.p1 |  | Image |  | Peak List |  |
|  | | | | | | | | | | | | | | | | | | | | | | | | | | | | | | | | | | | | | | | | | | | | | | | | | | | | | |  |
|  | High |  | [GS].KSAAQSVLKTLScKLSNDc.[-] |  | HCD |  | C13(Dehydro); C19(Dehydro) |  | 2.27 |  | 1.3e-3 |  | 4.4e-3 |  | 0.871 |  | 314.72 |  | 1.000 |  |  |  | C13(Dehydro): 100; C19(Dehydro): 100 |  |  |  |  |  |  |  | 0.54 |  | 1 |  | Sequest HT (3) |  | 2 |  | 997.50623 |  | 0.33 |  | 32.4897 |  | 7536 |  | 7536 |  | TRINITY\_DN0\_c1\_g1\_i15.p1; TRINITY\_DN0\_c1\_g1\_i23.p1; TRINITY\_DN0\_c1\_g1\_i11.p1; TRINITY\_DN45\_c27\_g1\_i1.p1; TRINITY\_DN2658\_c0\_g2\_i1.p1\_ORF2; TRINITY\_DN0\_c1\_g1\_i10.p1; TRINITY\_DN2658\_c0\_g2\_i1.p1\_ORF1; TRINITY\_DN2658\_c0\_g2\_i1.p1 |  | Image |  | Peak List |  |
|  | | | | | | | | | | | | | | | | | | | | | | | | | | | | | | | | | | | | | | | | | | | | | | | | | | | | | |  |
|  | High |  | [A].GKSAAQSVLKTLScKLSNDc.[-] |  | HCD |  | C14(Dehydro); C20(Dehydro) |  | 3.57 |  | 1.3e-3 |  | 6.3e-2 |  | 0.284 |  | 190.49 |  | 1.000 |  |  |  | C14(Dehydro): 100; C20(Dehydro): 100 |  |  |  |  |  |  |  | 0.44 |  | 1 |  | Sequest HT (3) |  | 4 |  | 513.51184 |  | -0.22 |  | 32.5742 |  | 7574 |  | 7574 |  | TRINITY\_DN0\_c1\_g1\_i15.p1; TRINITY\_DN0\_c1\_g1\_i23.p1; TRINITY\_DN0\_c1\_g1\_i11.p1; TRINITY\_DN45\_c27\_g1\_i1.p1; TRINITY\_DN0\_c1\_g1\_i10.p1 |  | Image |  | Peak List |  |
|  | | | | | | | | | | | | | | | | | | | | | | | | | | | | | | | | | | | | | | | | | | | | | | | | | | | | | |  |
|  | High |  | [A].GKSAAQSVLKTLScKLSNDc.[-] |  | HCD |  | C14(Dehydro); C20(Dehydro) |  | 2.88 |  | 1.3e-3 |  | 4.0e-2 |  | 0.385 |  | 212.66 |  | 1.000 |  |  |  | C14(Dehydro): 100; C20(Dehydro): 100 |  |  |  |  |  |  |  | 0.56 |  | 1 |  | Sequest HT (3) |  | 3 |  | 684.34680 |  | -0.07 |  | 32.6069 |  | 7588 |  | 7588 |  | TRINITY\_DN0\_c1\_g1\_i15.p1; TRINITY\_DN0\_c1\_g1\_i23.p1; TRINITY\_DN0\_c1\_g1\_i11.p1; TRINITY\_DN45\_c27\_g1\_i1.p1; TRINITY\_DN0\_c1\_g1\_i10.p1 |  | Image |  | Peak List |  |
|  | | | | | | | | | | | | | | | | | | | | | | | | | | | | | | | | | | | | | | | | | | | | | | | | | | | | | |  |
|  | High |  | [A].GKSAAQSVLKTLScKLSNDc.[-] |  | HCD |  | C14(Dehydro); C20(Dehydro) |  | 2.87 |  | 1.3e-3 |  | 1.2e-3 |  | 1.15 |  | 316.46 |  | 1.000 |  |  |  | C14(Dehydro): 100; C20(Dehydro): 100 |  |  |  |  |  |  |  | 0.44 |  | 1 |  | Sequest HT (3) |  | 2 |  | 1026.01685 |  | 0.21 |  | 32.6138 |  | 7592 |  | 7592 |  | TRINITY\_DN0\_c1\_g1\_i15.p1; TRINITY\_DN0\_c1\_g1\_i23.p1; TRINITY\_DN0\_c1\_g1\_i11.p1; TRINITY\_DN45\_c27\_g1\_i1.p1; TRINITY\_DN0\_c1\_g1\_i10.p1 |  | Image |  | Peak List |  |
|  | | | | | | | | | | | | | | | | | | | | | | | | | | | | | | | | | | | | | | | | | | | | | | | | | | | | | |  |
|  | High |  | [GS].KSAAQSVLKTLScKLSnDc.[-] |  | HCD |  | C13(Dehydro); N17(Deamidated); C19(Dehydro) |  | 2.66 |  | 3.1e-3 |  | 1.1e-1 |  | 0.162 |  | 164.04 |  | 1.000 |  |  |  | C13(Dehydro): 100; N17(Deamidated): 100; C19(Dehydro): 100 |  |  |  |  |  |  |  | 0.58 |  | 1 |  | Sequest HT (3) |  | 3 |  | 665.66742 |  | -0.42 |  | 32.6438 |  | 7606 |  | 7606 |  | TRINITY\_DN0\_c1\_g1\_i15.p1; TRINITY\_DN0\_c1\_g1\_i23.p1; TRINITY\_DN0\_c1\_g1\_i11.p1; TRINITY\_DN45\_c27\_g1\_i1.p1; TRINITY\_DN2658\_c0\_g2\_i1.p1\_ORF2; TRINITY\_DN0\_c1\_g1\_i10.p1; TRINITY\_DN2658\_c0\_g2\_i1.p1\_ORF1; TRINITY\_DN2658\_c0\_g2\_i1.p1 |  | Image |  | Peak List |  |
|  | | | | | | | | | | | | | | | | | | | | | | | | | | | | | | | | | | | | | | | | | | | | | | | | | | | | | |  |
|  | High |  | [GS].KSAAQSVLKTLScKLSnDc.[-] |  | HCD |  | C13(Dehydro); N17(Deamidated); C19(Dehydro) |  | 2.84 |  | 1.3e-3 |  | 2.6e-2 |  | 0.48 |  | 247.07 |  | 1.000 |  |  |  | C13(Dehydro): 100; N17(Deamidated): 100; C19(Dehydro): 100 |  |  |  |  |  |  |  | 0.48 |  | 1 |  | Sequest HT (3) |  | 2 |  | 997.99811 |  | 0.20 |  | 32.6558 |  | 7612 |  | 7612 |  | TRINITY\_DN0\_c1\_g1\_i15.p1; TRINITY\_DN0\_c1\_g1\_i23.p1; TRINITY\_DN0\_c1\_g1\_i11.p1; TRINITY\_DN45\_c27\_g1\_i1.p1; TRINITY\_DN2658\_c0\_g2\_i1.p1\_ORF2; TRINITY\_DN0\_c1\_g1\_i10.p1; TRINITY\_DN2658\_c0\_g2\_i1.p1\_ORF1; TRINITY\_DN2658\_c0\_g2\_i1.p1 |  | Image |  | Peak List |  |
|  | | | | | | | | | | | | | | | | | | | | | | | | | | | | | | | | | | | | | | | | | | | | | | | | | | | | | |  |
|  | High |  | [GS].KSAAQSVLKTLScKLSnDc.[-] |  | HCD |  | C13(Dehydro); N17(Deamidated); C19(Dehydro) |  | 2.77 |  | 1.3e-3 |  | 4.1e-2 |  | 0.383 |  | 217.58 |  | 1.000 |  |  |  | C13(Dehydro): 100; N17(Deamidated): 100; C19(Dehydro): 100 |  |  |  |  |  |  |  | 0.61 |  | 1 |  | Sequest HT (3) |  | 4 |  | 499.50272 |  | 0.25 |  | 32.6665 |  | 7616 |  | 7616 |  | TRINITY\_DN0\_c1\_g1\_i15.p1; TRINITY\_DN0\_c1\_g1\_i23.p1; TRINITY\_DN0\_c1\_g1\_i11.p1; TRINITY\_DN45\_c27\_g1\_i1.p1; TRINITY\_DN2658\_c0\_g2\_i1.p1\_ORF2; TRINITY\_DN0\_c1\_g1\_i10.p1; TRINITY\_DN2658\_c0\_g2\_i1.p1\_ORF1; TRINITY\_DN2658\_c0\_g2\_i1.p1 |  | Image |  | Peak List |  |
|  | | | | | | | | | | | | | | | | | | | | | | | | | | | | | | | | | | | | | | | | | | | | | | | | | | | | | |  |
|  | High |  | [G].AGKSAAQSVLKTLScKLSNDc.[-] |  | HCD |  | C15(Dehydro); C21(Dehydro) |  | 5.00 |  | 1.3e-3 |  | 2.8e-3 |  | 0.968 |  | 331.78 |  | 1.000 |  |  |  | C15(Dehydro): 100; C21(Dehydro): 100 |  |  |  |  |  |  |  | 0.45 |  | 1 |  | Sequest HT (3) |  | 3 |  | 708.02643 |  | 0.77 |  | 32.7077 |  | 7633 |  | 7633 |  | TRINITY\_DN0\_c1\_g1\_i15.p1; TRINITY\_DN0\_c1\_g1\_i23.p1; TRINITY\_DN0\_c1\_g1\_i11.p1; TRINITY\_DN45\_c27\_g1\_i1.p1; TRINITY\_DN0\_c1\_g1\_i10.p1 |  | Image |  | Peak List |  |
|  | | | | | | | | | | | | | | | | | | | | | | | | | | | | | | | | | | | | | | | | | | | | | | | | | | | | | |  |
|  | High |  | [G].AGKSAAQSVLKTLScKLSNDc.[-] |  | HCD |  | C15(Dehydro); C21(Dehydro) |  | 3.98 |  | 3.1e-3 |  | 1.0e-1 |  | 0.178 |  | 220.80 |  | 1.000 |  |  |  | C15(Dehydro): 100; C21(Dehydro): 100 |  |  |  |  |  |  |  | 0.38 |  | 1 |  | Sequest HT (3) |  | 4 |  | 531.27197 |  | 1.40 |  | 32.7545 |  | 7653 |  | 7653 |  | TRINITY\_DN0\_c1\_g1\_i15.p1; TRINITY\_DN0\_c1\_g1\_i23.p1; TRINITY\_DN0\_c1\_g1\_i11.p1; TRINITY\_DN45\_c27\_g1\_i1.p1; TRINITY\_DN0\_c1\_g1\_i10.p1 |  | Image |  | Peak List |  |
|  | | | | | | | | | | | | | | | | | | | | | | | | | | | | | | | | | | | | | | | | | | | | | | | | | | | | | |  |
|  | High |  | [G].AGKSAAQSVLKTLScKLSNDc.[-] |  | HCD |  | C15(Dehydro); C21(Dehydro) |  | 5.34 |  | 1.3e-3 |  | 1.6e-4 |  | 1.59 |  | 382.94 |  | 1.000 |  |  |  | C15(Dehydro): 100; C21(Dehydro): 100 |  |  |  |  |  |  |  | 0.47 |  | 1 |  | Sequest HT (3) |  | 2 |  | 1061.53540 |  | 0.20 |  | 32.7594 |  | 7656 |  | 7656 |  | TRINITY\_DN0\_c1\_g1\_i15.p1; TRINITY\_DN0\_c1\_g1\_i23.p1; TRINITY\_DN0\_c1\_g1\_i11.p1; TRINITY\_DN45\_c27\_g1\_i1.p1; TRINITY\_DN0\_c1\_g1\_i10.p1 |  | Image |  | Peak List |  |
|  | | | | | | | | | | | | | | | | | | | | | | | | | | | | | | | | | | | | | | | | | | | | | | | | | | | | | |  |
|  | High |  | [A].AQSVLKTLScKLSnDc.[-] |  | HCD |  | C10(Dehydro); N14(Deamidated); C16(Dehydro) |  | 2.69 |  | 2.4e-3 |  | 7.1e-2 |  | 0.26 |  | 200.69 |  | 1.000 |  |  |  | C10(Dehydro): 100; N14(Deamidated): 100; C16(Dehydro): 100 |  |  |  |  |  |  |  | 0.43 |  | 1 |  | Sequest HT (3) |  | 2 |  | 854.91431 |  | -1.81 |  | 32.8138 |  | 7680 |  | 7680 |  | TRINITY\_DN0\_c1\_g1\_i15.p1; TRINITY\_DN0\_c1\_g1\_i23.p1; TRINITY\_DN0\_c1\_g1\_i11.p1; TRINITY\_DN45\_c27\_g1\_i1.p1; TRINITY\_DN2658\_c0\_g2\_i1.p1\_ORF2; TRINITY\_DN0\_c1\_g1\_i10.p1; TRINITY\_DN2658\_c0\_g2\_i1.p1\_ORF1; TRINITY\_DN2658\_c0\_g2\_i1.p1 |  | Image |  | Peak List |  |
|  | | | | | | | | | | | | | | | | | | | | | | | | | | | | | | | | | | | | | | | | | | | | | | | | | | | | | |  |
|  | High |  | [G].AGKSAAQSVLKTLScKLSNDc.[-] |  | HCD |  | C15(Dehydro); C21(Dehydro) |  | 3.35 |  | 1.3e-3 |  | 1.9e-2 |  | 0.551 |  | 221.99 |  | 1.000 |  |  |  | C15(Dehydro): 100; C21(Dehydro): 100 |  |  |  |  |  |  |  | 0.61 |  | 1 |  | Sequest HT (3) |  | 3 |  | 708.02606 |  | 0.25 |  | 32.8465 |  | 7693 |  | 7693 |  | TRINITY\_DN0\_c1\_g1\_i15.p1; TRINITY\_DN0\_c1\_g1\_i23.p1; TRINITY\_DN0\_c1\_g1\_i11.p1; TRINITY\_DN45\_c27\_g1\_i1.p1; TRINITY\_DN0\_c1\_g1\_i10.p1 |  | Image |  | Peak List |  |
|  | | | | | | | | | | | | | | | | | | | | | | | | | | | | | | | | | | | | | | | | | | | | | | | | | | | | | |  |
|  | High |  | [A].GKSAAQSVLKTLScKLSnDc.[-] |  | HCD |  | C14(Dehydro); N18(Deamidated); C20(Dehydro) |  | 3.04 |  | 1.3e-3 |  | 2.8e-2 |  | 0.466 |  | 201.17 |  | 1.000 |  |  |  | C14(Dehydro): 100; N18(Deamidated): 100; C20(Dehydro): 100 |  |  |  |  |  |  |  | 0.42 |  | 1 |  | Sequest HT (3) |  | 4 |  | 513.75879 |  | 1.62 |  | 32.8697 |  | 7702 |  | 7702 |  | TRINITY\_DN0\_c1\_g1\_i15.p1; TRINITY\_DN0\_c1\_g1\_i23.p1; TRINITY\_DN0\_c1\_g1\_i11.p1; TRINITY\_DN45\_c27\_g1\_i1.p1; TRINITY\_DN0\_c1\_g1\_i10.p1 |  | Image |  | Peak List |  |
|  | | | | | | | | | | | | | | | | | | | | | | | | | | | | | | | | | | | | | | | | | | | | | | | | | | | | | |  |
|  | High |  | [G].AGKSAAQSVLKTLScKLSNDc.[-] |  | HCD |  | C15(Dehydro); C21(Dehydro) |  | 1.87 |  | 1.3e-3 |  | 2.3e-2 |  | 0.509 |  | 210.03 |  | 1.000 |  |  |  | C15(Dehydro): 100; C21(Dehydro): 100 |  |  |  |  |  |  |  | 0.40 |  | 1 |  | Sequest HT (3) |  | 2 |  | 1061.53552 |  | 0.32 |  | 32.9117 |  | 7718 |  | 7718 |  | TRINITY\_DN0\_c1\_g1\_i15.p1; TRINITY\_DN0\_c1\_g1\_i23.p1; TRINITY\_DN0\_c1\_g1\_i11.p1; TRINITY\_DN45\_c27\_g1\_i1.p1; TRINITY\_DN0\_c1\_g1\_i10.p1 |  | Image |  | Peak List |  |
|  | | | | | | | | | | | | | | | | | | | | | | | | | | | | | | | | | | | | | | | | | | | | | | | | | | | | | |  |
|  | High |  | [A].GKSAAQSVLKTLScKLSnDc.[-] |  | HCD |  | C14(Dehydro); N18(Deamidated); C20(Dehydro) |  | 3.15 |  | 1.3e-3 |  | 6.5e-3 |  | 0.785 |  | 238.55 |  | 1.000 |  |  |  | C14(Dehydro): 100; N18(Deamidated): 100; C20(Dehydro): 100 |  |  |  |  |  |  |  | 0.47 |  | 1 |  | Sequest HT (3) |  | 3 |  | 684.67438 |  | -0.69 |  | 32.9129 |  | 7719 |  | 7719 |  | TRINITY\_DN0\_c1\_g1\_i15.p1; TRINITY\_DN0\_c1\_g1\_i23.p1; TRINITY\_DN0\_c1\_g1\_i11.p1; TRINITY\_DN45\_c27\_g1\_i1.p1; TRINITY\_DN0\_c1\_g1\_i10.p1 |  | Image |  | Peak List |  |
|  | | | | | | | | | | | | | | | | | | | | | | | | | | | | | | | | | | | | | | | | | | | | | | | | | | | | | |  |
|  | High |  | [G].AGKSAAQSVLKTLScKLSnDc.[-] |  | HCD |  | C15(Dehydro); N19(Deamidated); C21(Dehydro) |  | 3.72 |  | 1.3e-3 |  | 9.3e-4 |  | 1.21 |  | 322.50 |  | 1.000 |  |  |  | C15(Dehydro): 100; N19(Deamidated): 100; C21(Dehydro): 100 |  |  |  |  |  |  |  | 0.49 |  | 1 |  | Sequest HT (3) |  | 3 |  | 708.35455 |  | 0.94 |  | 33.0154 |  | 7763 |  | 7763 |  | TRINITY\_DN0\_c1\_g1\_i15.p1; TRINITY\_DN0\_c1\_g1\_i23.p1; TRINITY\_DN0\_c1\_g1\_i11.p1; TRINITY\_DN45\_c27\_g1\_i1.p1; TRINITY\_DN0\_c1\_g1\_i10.p1 |  | Image |  | Peak List |  |
|  | | | | | | | | | | | | | | | | | | | | | | | | | | | | | | | | | | | | | | | | | | | | | | | | | | | | | |  |
|  | High |  | [S].AAQSVLKTLScKLSNDc.[-] |  | HCD |  | C11(Dehydro); C17(Dehydro) |  | 3.29 |  | 1.3e-3 |  | 8.3e-3 |  | 0.731 |  | 248.78 |  | 1.000 |  |  |  | C11(Dehydro): 100; C17(Dehydro): 100 |  |  |  |  |  |  |  | 0.56 |  | 1 |  | Sequest HT (3) |  | 2 |  | 889.94220 |  | -0.23 |  | 33.0598 |  | 7783 |  | 7783 |  | TRINITY\_DN0\_c1\_g1\_i15.p1; TRINITY\_DN0\_c1\_g1\_i23.p1; TRINITY\_DN0\_c1\_g1\_i11.p1; TRINITY\_DN45\_c27\_g1\_i1.p1; TRINITY\_DN2658\_c0\_g2\_i1.p1\_ORF2; TRINITY\_DN0\_c1\_g1\_i10.p1; TRINITY\_DN2658\_c0\_g2\_i1.p1\_ORF1; TRINITY\_DN2658\_c0\_g2\_i1.p1 |  | Image |  | Peak List |  |
|  | | | | | | | | | | | | | | | | | | | | | | | | | | | | | | | | | | | | | | | | | | | | | | | | | | | | | |  |
|  | High |  | [G].AGKSAAQSVLKTLScKLSnDc.[-] |  | HCD |  | C15(Dehydro); N19(Deamidated); C21(Dehydro) |  | 2.85 |  | 1.3e-3 |  | 4.8e-4 |  | 1.36 |  | 307.83 |  | 1.000 |  |  |  | C15(Dehydro): 100; N19(Deamidated): 100; C21(Dehydro): 100 |  |  |  |  |  |  |  | 0.47 |  | 1 |  | Sequest HT (3) |  | 2 |  | 1062.02576 |  | -1.36 |  | 33.1092 |  | 7804 |  | 7804 |  | TRINITY\_DN0\_c1\_g1\_i15.p1; TRINITY\_DN0\_c1\_g1\_i23.p1; TRINITY\_DN0\_c1\_g1\_i11.p1; TRINITY\_DN45\_c27\_g1\_i1.p1; TRINITY\_DN0\_c1\_g1\_i10.p1 |  | Image |  | Peak List |  |
|  | | | | | | | | | | | | | | | | | | | | | | | | | | | | | | | | | | | | | | | | | | | | | | | | | | | | | |  |
|  | High |  | [R].GVLGTVKNLLIGAGKS.[A] |  | HCD |  |  |  | 5.29 |  | 1.3e-3 |  | 5.7e-3 |  | 0.815 |  |  |  |  |  |  |  |  |  |  |  |  |  |  |  | 0.51 |  | 1 |  | Sequest HT (3) |  | 3 |  | 509.64667 |  | -0.66 |  | 33.1139 |  | 7806 |  | 7806 |  | TRINITY\_DN0\_c1\_g1\_i15.p1; TRINITY\_DN0\_c1\_g1\_i23.p1; TRINITY\_DN0\_c1\_g1\_i11.p1; TRINITY\_DN0\_c1\_g1\_i10.p1 |  | Image |  | Peak List |  |
|  | | | | | | | | | | | | | | | | | | | | | | | | | | | | | | | | | | | | | | | | | | | | | | | | | | | | | |  |
|  | High |  | [R].GVLGTVKNLLIGAGKS.[A] |  | HCD |  |  |  | 2.00 |  | 1.3e-3 |  | 1.8e-2 |  | 0.557 |  |  |  |  |  |  |  |  |  |  |  |  |  |  |  | 0.29 |  | 1 |  | Sequest HT (3) |  | 2 |  | 763.96631 |  | -0.73 |  | 33.1880 |  | 7837 |  | 7837 |  | TRINITY\_DN0\_c1\_g1\_i15.p1; TRINITY\_DN0\_c1\_g1\_i23.p1; TRINITY\_DN0\_c1\_g1\_i11.p1; TRINITY\_DN0\_c1\_g1\_i10.p1 |  | Image |  | Peak List |  |
|  | | | | | | | | | | | | | | | | | | | | | | | | | | | | | | | | | | | | | | | | | | | | | | | | | | | | | |  |
|  | High |  | [R].GVLGTVKNLLIGAGK.[S] |  | HCD |  |  |  | 3.18 |  | 1.3e-3 |  | 6.0e-4 |  | 1.3 |  |  |  |  |  |  |  |  |  |  |  |  |  |  |  | 0.60 |  | 1 |  | Sequest HT (3) |  | 2 |  | 720.45007 |  | -1.08 |  | 33.1892 |  | 7838 |  | 7838 |  | TRINITY\_DN0\_c1\_g1\_i15.p1; TRINITY\_DN0\_c1\_g1\_i23.p1; TRINITY\_DN0\_c1\_g1\_i11.p1; TRINITY\_DN0\_c1\_g1\_i10.p1 |  | Image |  | Peak List |  |
|  | | | | | | | | | | | | | | | | | | | | | | | | | | | | | | | | | | | | | | | | | | | | | | | | | | | | | |  |
|  | High |  | [R].GVLGTVKNLLIGAGK.[S] |  | HCD |  |  |  | 4.53 |  | 1.3e-3 |  | 1.2e-3 |  | 1.16 |  |  |  |  |  |  |  |  |  |  |  |  |  |  |  | 0.62 |  | 1 |  | Sequest HT (3) |  | 3 |  | 480.63620 |  | -0.26 |  | 33.1932 |  | 7841 |  | 7841 |  | TRINITY\_DN0\_c1\_g1\_i15.p1; TRINITY\_DN0\_c1\_g1\_i23.p1; TRINITY\_DN0\_c1\_g1\_i11.p1; TRINITY\_DN0\_c1\_g1\_i10.p1 |  | Image |  | Peak List |  |
|  | | | | | | | | | | | | | | | | | | | | | | | | | | | | | | | | | | | | | | | | | | | | | | | | | | | | | |  |
|  | High |  | [R].GVLGTVKNLLIGAGK.[S] |  | HCD |  |  |  | 2.71 |  | 1.3e-3 |  | 7.0e-3 |  | 0.767 |  |  |  |  |  |  |  |  |  |  |  |  |  |  |  | 0.67 |  | 1 |  | Sequest HT (3) |  | 2 |  | 720.45221 |  | 1.89 |  | 33.3415 |  | 7903 |  | 7903 |  | TRINITY\_DN0\_c1\_g1\_i15.p1; TRINITY\_DN0\_c1\_g1\_i23.p1; TRINITY\_DN0\_c1\_g1\_i11.p1; TRINITY\_DN0\_c1\_g1\_i10.p1 |  | Image |  | Peak List |  |
|  | | | | | | | | | | | | | | | | | | | | | | | | | | | | | | | | | | | | | | | | | | | | | | | | | | | | | |  |
|  | High |  | [R].GVLGTVKNLLIGAGK.[S] |  | HCD |  |  |  | 3.43 |  | 1.3e-3 |  | 4.0e-3 |  | 0.893 |  |  |  |  |  |  |  |  |  |  |  |  |  |  |  | 0.56 |  | 1 |  | Sequest HT (3) |  | 3 |  | 480.63742 |  | 2.28 |  | 33.3428 |  | 7904 |  | 7904 |  | TRINITY\_DN0\_c1\_g1\_i15.p1; TRINITY\_DN0\_c1\_g1\_i23.p1; TRINITY\_DN0\_c1\_g1\_i11.p1; TRINITY\_DN0\_c1\_g1\_i10.p1 |  | Image |  | Peak List |  |
|  | | | | | | | | | | | | | | | | | | | | | | | | | | | | | | | | | | | | | | | | | | | | | | | | | | | | | |  |
|  | High |  | [R].GVLGTVKNLLIGAGKSAAQ.[S] |  | HCD |  |  |  | 3.63 |  | 1.3e-3 |  | 1.8e-3 |  | 1.07 |  |  |  |  |  |  |  |  |  |  |  |  |  |  |  | 0.51 |  | 1 |  | Sequest HT (3) |  | 2 |  | 899.03107 |  | -2.45 |  | 33.5630 |  | 7990 |  | 7990 |  | TRINITY\_DN0\_c1\_g1\_i15.p1; TRINITY\_DN0\_c1\_g1\_i23.p1; TRINITY\_DN0\_c1\_g1\_i11.p1; TRINITY\_DN0\_c1\_g1\_i10.p1 |  | Image |  | Peak List |  |
|  | | | | | | | | | | | | | | | | | | | | | | | | | | | | | | | | | | | | | | | | | | | | | | | | | | | | | |  |
|  | High |  | [R].GVLGTVKNLLIGAGKSAAQ.[S] |  | HCD |  |  |  | 4.04 |  | 1.3e-3 |  | 2.2e-2 |  | 0.522 |  |  |  |  |  |  |  |  |  |  |  |  |  |  |  | 0.57 |  | 1 |  | Sequest HT (3) |  | 3 |  | 599.69171 |  | 0.74 |  | 33.5803 |  | 7998 |  | 7998 |  | TRINITY\_DN0\_c1\_g1\_i15.p1; TRINITY\_DN0\_c1\_g1\_i23.p1; TRINITY\_DN0\_c1\_g1\_i11.p1; TRINITY\_DN0\_c1\_g1\_i10.p1 |  | Image |  | Peak List |  |
|  | | | | | | | | | | | | | | | | | | | | | | | | | | | | | | | | | | | | | | | | | | | | | | | | | | | | | |  |
|  | High |  | [R].GVLGTVKNLLIGAGKSAAQS.[V] |  | HCD |  |  |  | 2.79 |  | 4.7e-3 |  | 2.0e-1 |  | 0.027 |  |  |  |  |  |  |  |  |  |  |  |  |  |  |  | 0.28 |  | 1 |  | Sequest HT (3) |  | 3 |  | 628.70282 |  | 1.39 |  | 33.7710 |  | 8085 |  | 8085 |  | TRINITY\_DN0\_c1\_g1\_i15.p1; TRINITY\_DN0\_c1\_g1\_i23.p1; TRINITY\_DN0\_c1\_g1\_i11.p1; TRINITY\_DN0\_c1\_g1\_i10.p1 |  | Image |  | Peak List |  |
|  | | | | | | | | | | | | | | | | | | | | | | | | | | | | | | | | | | | | | | | | | | | | | | | | | | | | | |  |
|  | High |  | [R].GVLGTVKNLLIGAGKSAAQS.[V] |  | HCD |  |  |  | 2.05 |  | 1.3e-3 |  | 1.0e-2 |  | 0.688 |  |  |  |  |  |  |  |  |  |  |  |  |  |  |  | 0.31 |  | 1 |  | Sequest HT (3) |  | 2 |  | 942.54987 |  | 0.62 |  | 33.7878 |  | 8092 |  | 8092 |  | TRINITY\_DN0\_c1\_g1\_i15.p1; TRINITY\_DN0\_c1\_g1\_i23.p1; TRINITY\_DN0\_c1\_g1\_i11.p1; TRINITY\_DN0\_c1\_g1\_i10.p1 |  | Image |  | Peak List |  |
|  | | | | | | | | | | | | | | | | | | | | | | | | | | | | | | | | | | | | | | | | | | | | | | | | | | | | | |  |
|  | High |  | [R].GVLGTVKNLLIGAGKSAA.[QP] |  | HCD |  |  |  | 3.45 |  | 1.3e-3 |  | 3.9e-2 |  | 0.395 |  |  |  |  |  |  |  |  |  |  |  |  |  |  |  | 0.41 |  | 1 |  | Sequest HT (3) |  | 3 |  | 557.00537 |  | 0.53 |  | 33.8167 |  | 8105 |  | 8105 |  | TRINITY\_DN0\_c1\_g1\_i15.p1; TRINITY\_DN0\_c1\_g1\_i23.p1; TRINITY\_DN0\_c1\_g1\_i11.p1; TRINITY\_DN0\_c1\_g1\_i10.p1 |  | Image |  | Peak List |  |
|  | | | | | | | | | | | | | | | | | | | | | | | | | | | | | | | | | | | | | | | | | | | | | | | | | | | | | |  |
|  | High |  | [R].GVLGTVKNLLIGAGKSAA.[QP] |  | HCD |  |  |  | 4.68 |  | 1.3e-3 |  | 1.1e-4 |  | 1.67 |  |  |  |  |  |  |  |  |  |  |  |  |  |  |  | 0.61 |  | 1 |  | Sequest HT (3) |  | 2 |  | 835.00354 |  | -0.53 |  | 33.8244 |  | 8108 |  | 8108 |  | TRINITY\_DN0\_c1\_g1\_i15.p1; TRINITY\_DN0\_c1\_g1\_i23.p1; TRINITY\_DN0\_c1\_g1\_i11.p1; TRINITY\_DN0\_c1\_g1\_i10.p1 |  | Image |  | Peak List |  |
|  | | | | | | | | | | | | | | | | | | | | | | | | | | | | | | | | | | | | | | | | | | | | | | | | | | | | | |  |
|  | High |  | [R].GVLGTVKNLLIGAGKSA.[A] |  | HCD |  |  |  | 3.01 |  | 1.3e-3 |  | 1.1e-4 |  | 1.67 |  |  |  |  |  |  |  |  |  |  |  |  |  |  |  | 0.44 |  | 1 |  | Sequest HT (3) |  | 2 |  | 799.48438 |  | -1.31 |  | 33.9039 |  | 8144 |  | 8144 |  | TRINITY\_DN0\_c1\_g1\_i15.p1; TRINITY\_DN0\_c1\_g1\_i23.p1; TRINITY\_DN0\_c1\_g1\_i11.p1; TRINITY\_DN0\_c1\_g1\_i10.p1 |  | Image |  | Peak List |  |
|  | | | | | | | | | | | | | | | | | | | | | | | | | | | | | | | | | | | | | | | | | | | | | | | | | | | | | |  |
|  | High |  | [R].GVLGTVKNLLIGAGKSA.[A] |  | HCD |  |  |  | 3.26 |  | 1.3e-3 |  | 7.0e-3 |  | 0.769 |  |  |  |  |  |  |  |  |  |  |  |  |  |  |  | 0.34 |  | 1 |  | Sequest HT (3) |  | 3 |  | 533.32581 |  | -0.44 |  | 33.9409 |  | 8160 |  | 8160 |  | TRINITY\_DN0\_c1\_g1\_i15.p1; TRINITY\_DN0\_c1\_g1\_i23.p1; TRINITY\_DN0\_c1\_g1\_i11.p1; TRINITY\_DN0\_c1\_g1\_i10.p1 |  | Image |  | Peak List |  |
|  | | | | | | | | | | | | | | | | | | | | | | | | | | | | | | | | | | | | | | | | | | | | | | | | | | | | | |  |
|  | High |  | [R].GVLGTVKNLLIGAGKSAA.[QP] |  | HCD |  |  |  | 2.77 |  | 3.1e-3 |  | 1.1e-1 |  | 0.154 |  |  |  |  |  |  |  |  |  |  |  |  |  |  |  | 0.43 |  | 1 |  | Sequest HT (3) |  | 3 |  | 557.00476 |  | -0.57 |  | 33.9634 |  | 8171 |  | 8171 |  | TRINITY\_DN0\_c1\_g1\_i15.p1; TRINITY\_DN0\_c1\_g1\_i23.p1; TRINITY\_DN0\_c1\_g1\_i11.p1; TRINITY\_DN0\_c1\_g1\_i10.p1 |  | Image |  | Peak List |  |
|  | | | | | | | | | | | | | | | | | | | | | | | | | | | | | | | | | | | | | | | | | | | | | | | | | | | | | |  |
|  | High |  | [R].GVLGTVKNLLIGAGKSAA.[QP] |  | HCD |  |  |  | 2.50 |  | 1.3e-3 |  | 4.0e-2 |  | 0.384 |  |  |  |  |  |  |  |  |  |  |  |  |  |  |  | 0.50 |  | 1 |  | Sequest HT (3) |  | 2 |  | 835.00330 |  | -0.82 |  | 33.9651 |  | 8172 |  | 8172 |  | TRINITY\_DN0\_c1\_g1\_i15.p1; TRINITY\_DN0\_c1\_g1\_i23.p1; TRINITY\_DN0\_c1\_g1\_i11.p1; TRINITY\_DN0\_c1\_g1\_i10.p1 |  | Image |  | Peak List |  |
|  | | | | | | | | | | | | | | | | | | | | | | | | | | | | | | | | | | | | | | | | | | | | | | | | | | | | | |  |
|  | High |  | [R].GVLGTVKNLLIGAGKSA.[A] |  | HCD |  |  |  | 1.74 |  | 3.1e-3 |  | 8.8e-2 |  | 0.211 |  |  |  |  |  |  |  |  |  |  |  |  |  |  |  | 0.34 |  | 1 |  | Sequest HT (3) |  | 2 |  | 799.48541 |  | -0.01 |  | 34.0540 |  | 8212 |  | 8212 |  | TRINITY\_DN0\_c1\_g1\_i15.p1; TRINITY\_DN0\_c1\_g1\_i23.p1; TRINITY\_DN0\_c1\_g1\_i11.p1; TRINITY\_DN0\_c1\_g1\_i10.p1 |  | Image |  | Peak List |  |
|  | | | | | | | | | | | | | | | | | | | | | | | | | | | | | | | | | | | | | | | | | | | | | | | | | | | | | |  |
|  | High |  | [L].IGAGKSAAQSVLKTLScKLSNDc.[-] |  | HCD |  | C17(Dehydro); C23(Dehydro) |  | 2.68 |  | 6.5e-3 |  | 2.2e-1 |  | 0.001 |  | 131.02 |  | 1.000 |  |  |  | C17(Dehydro): 100; C23(Dehydro): 100 |  |  |  |  |  |  |  | 0.49 |  | 1 |  | Sequest HT (3) |  | 4 |  | 573.79932 |  | 2.97 |  | 34.1545 |  | 8256 |  | 8256 |  | TRINITY\_DN0\_c1\_g1\_i15.p1; TRINITY\_DN0\_c1\_g1\_i23.p1; TRINITY\_DN0\_c1\_g1\_i11.p1; TRINITY\_DN45\_c27\_g1\_i1.p1; TRINITY\_DN0\_c1\_g1\_i10.p1 |  | Image |  | Peak List |  |
|  | | | | | | | | | | | | | | | | | | | | | | | | | | | | | | | | | | | | | | | | | | | | | | | | | | | | | |  |
|  | High |  | [L].IGAGKSAAQSVLKTLScKLSNDc.[-] |  | HCD |  | C17(Dehydro); C23(Dehydro) |  | 3.45 |  | 1.3e-3 |  | 2.7e-3 |  | 0.978 |  | 250.83 |  | 1.000 |  |  |  | C17(Dehydro): 100; C23(Dehydro): 100 |  |  |  |  |  |  |  | 0.54 |  | 1 |  | Sequest HT (3) |  | 3 |  | 764.72876 |  | 1.35 |  | 34.1971 |  | 8275 |  | 8275 |  | TRINITY\_DN0\_c1\_g1\_i15.p1; TRINITY\_DN0\_c1\_g1\_i23.p1; TRINITY\_DN0\_c1\_g1\_i11.p1; TRINITY\_DN45\_c27\_g1\_i1.p1; TRINITY\_DN0\_c1\_g1\_i10.p1 |  | Image |  | Peak List |  |
|  | | | | | | | | | | | | | | | | | | | | | | | | | | | | | | | | | | | | | | | | | | | | | | | | | | | | | |  |
|  | High |  | [A].GKSAAQSVLKTLScKLSnDc.[-] |  | HCD |  | C14(Dehydro); N18(Deamidated); C20(Dehydro) |  | 1.75 |  | 4.7e-3 |  | 2.0e-1 |  | 0.022 |  | 165.82 |  | 1.000 |  |  |  | C14(Dehydro): 100; N18(Deamidated): 100; C20(Dehydro): 100 |  |  |  |  |  |  |  | 0.01 |  | 1 |  | Sequest HT (3) |  | 3 |  | 684.67413 |  | -1.05 |  | 34.3390 |  | 8339 |  | 8339 |  | TRINITY\_DN0\_c1\_g1\_i15.p1; TRINITY\_DN0\_c1\_g1\_i23.p1; TRINITY\_DN0\_c1\_g1\_i11.p1; TRINITY\_DN45\_c27\_g1\_i1.p1; TRINITY\_DN0\_c1\_g1\_i10.p1 |  | Image |  | Peak List |  |
|  | | | | | | | | | | | | | | | | | | | | | | | | | | | | | | | | | | | | | | | | | | | | | | | | | | | | | |  |
|  | High |  | [L].IGAGKSAAQSVLKTLScKLSnDc.[-] |  | HCD |  | C17(Dehydro); N21(Deamidated); C23(Dehydro) |  | 5.01 |  | 1.3e-3 |  | 3.4e-3 |  | 0.929 |  | 298.49 |  | 1.000 |  |  |  | C17(Dehydro): 100; N21(Deamidated): 100; C23(Dehydro): 100 |  |  |  |  |  |  |  | 0.62 |  | 1 |  | Sequest HT (3) |  | 3 |  | 765.05560 |  | -0.17 |  | 34.3971 |  | 8366 |  | 8366 |  | TRINITY\_DN0\_c1\_g1\_i15.p1; TRINITY\_DN0\_c1\_g1\_i23.p1; TRINITY\_DN0\_c1\_g1\_i11.p1; TRINITY\_DN45\_c27\_g1\_i1.p1; TRINITY\_DN0\_c1\_g1\_i10.p1 |  | Image |  | Peak List |  |
|  | | | | | | | | | | | | | | | | | | | | | | | | | | | | | | | | | | | | | | | | | | | | | | | | | | | | | |  |
|  | High |  | [G].AGKSAAQSVLKTLScKLSnDc.[-] |  | HCD |  | C15(Dehydro); N19(Deamidated); C21(Dehydro) |  | 1.95 |  | 1.3e-3 |  | 1.8e-2 |  | 0.568 |  | 218.38 |  | 1.000 |  |  |  | C15(Dehydro): 100; N19(Deamidated): 100; C21(Dehydro): 100 |  |  |  |  |  |  |  | 0.15 |  | 1 |  | Sequest HT (3) |  | 2 |  | 1062.02722 |  | 0.02 |  | 34.4048 |  | 8369 |  | 8369 |  | TRINITY\_DN0\_c1\_g1\_i15.p1; TRINITY\_DN0\_c1\_g1\_i23.p1; TRINITY\_DN0\_c1\_g1\_i11.p1; TRINITY\_DN45\_c27\_g1\_i1.p1; TRINITY\_DN0\_c1\_g1\_i10.p1 |  | Image |  | Peak List |  |
|  | | | | | | | | | | | | | | | | | | | | | | | | | | | | | | | | | | | | | | | | | | | | | | | | | | | | | |  |
|  | High |  | [L].IGAGKSAAQSVLKTLScKLSnDc.[-] |  | HCD |  | C17(Dehydro); N21(Deamidated); C23(Dehydro) |  | 2.34 |  | 3.1e-3 |  | 1.1e-1 |  | 0.171 |  | 124.93 |  | 1.000 |  |  |  | C17(Dehydro): 100; N21(Deamidated): 100; C23(Dehydro): 100 |  |  |  |  |  |  |  | 0.21 |  | 1 |  | Sequest HT (3) |  | 4 |  | 574.04340 |  | -0.39 |  | 34.4275 |  | 8378 |  | 8378 |  | TRINITY\_DN0\_c1\_g1\_i15.p1; TRINITY\_DN0\_c1\_g1\_i23.p1; TRINITY\_DN0\_c1\_g1\_i11.p1; TRINITY\_DN45\_c27\_g1\_i1.p1; TRINITY\_DN0\_c1\_g1\_i10.p1 |  | Image |  | Peak List |  |
|  | | | | | | | | | | | | | | | | | | | | | | | | | | | | | | | | | | | | | | | | | | | | | | | | | | | | | |  |
|  | High |  | [R].GVLGTVKnLLIGAGKS.[A] |  | HCD |  | N8(Deamidated) |  | 3.63 |  | 1.3e-3 |  | 2.1e-4 |  | 1.53 |  | 430.06 |  | 1.000 |  |  |  | N8(Deamidated): 100 |  |  |  |  |  |  |  |  |  | 2 |  | Sequest HT (3) |  | 2 |  | 764.45837 |  | -0.65 |  | 34.4327 |  | 8381 |  | 8381 |  | TRINITY\_DN0\_c1\_g1\_i15.p1; TRINITY\_DN0\_c1\_g1\_i23.p1; TRINITY\_DN0\_c1\_g1\_i11.p1; TRINITY\_DN0\_c1\_g1\_i10.p1 |  | Image |  | Peak List |  |
|  | | | | | | | | | | | | | | | | | | | | | | | | | | | | | | | | | | | | | | | | | | | | | | | | | | | | | |  |
|  | High |  | [R].GVLGTVKnLLIGAGKS.[A] |  | HCD |  | N8(Deamidated) |  | 4.68 |  | 1.3e-3 |  | 5.5e-4 |  | 1.32 |  | 265.39 |  | 1.000 |  |  |  | N8(Deamidated): 100 |  |  |  |  |  |  |  |  |  | 2 |  | Sequest HT (3) |  | 3 |  | 509.97565 |  | 1.26 |  | 34.4767 |  | 8401 |  | 8401 |  | TRINITY\_DN0\_c1\_g1\_i15.p1; TRINITY\_DN0\_c1\_g1\_i23.p1; TRINITY\_DN0\_c1\_g1\_i11.p1; TRINITY\_DN0\_c1\_g1\_i10.p1 |  | Image |  | Peak List |  |
|  | | | | | | | | | | | | | | | | | | | | | | | | | | | | | | | | | | | | | | | | | | | | | | | | | | | | | |  |
|  | High |  | [R].GVLGTVKnLLIGAGK.[S] |  | HCD |  | N8(Deamidated) |  | 4.59 |  | 1.3e-3 |  | 3.6e-3 |  | 0.913 |  | 317.75 |  | 1.000 |  |  |  | N8(Deamidated): 100 |  |  |  |  |  |  |  |  |  | 2 |  | Sequest HT (3) |  | 3 |  | 480.96423 |  | -0.20 |  | 34.5184 |  | 8422 |  | 8422 |  | TRINITY\_DN0\_c1\_g1\_i15.p1; TRINITY\_DN0\_c1\_g1\_i23.p1; TRINITY\_DN0\_c1\_g1\_i11.p1; TRINITY\_DN0\_c1\_g1\_i10.p1 |  | Image |  | Peak List |  |
|  | | | | | | | | | | | | | | | | | | | | | | | | | | | | | | | | | | | | | | | | | | | | | | | | | | | | | |  |
|  | High |  | [R].GVLGTVKnLLIGAGK.[S] |  | HCD |  | N8(Deamidated) |  | 4.25 |  | 1.3e-3 |  | 2.2e-3 |  | 1.02 |  | 445.08 |  | 1.000 |  |  |  | N8(Deamidated): 100 |  |  |  |  |  |  |  |  |  | 2 |  | Sequest HT (3) |  | 2 |  | 720.94202 |  | -1.17 |  | 34.5557 |  | 8437 |  | 8437 |  | TRINITY\_DN0\_c1\_g1\_i15.p1; TRINITY\_DN0\_c1\_g1\_i23.p1; TRINITY\_DN0\_c1\_g1\_i11.p1; TRINITY\_DN0\_c1\_g1\_i10.p1 |  | Image |  | Peak List |  |
|  | | | | | | | | | | | | | | | | | | | | | | | | | | | | | | | | | | | | | | | | | | | | | | | | | | | | | |  |
|  | High |  | [R].GVLGTVKnLLIGAGKS.[A] |  | HCD |  | N8(Deamidated) |  | 2.45 |  | 3.9e-3 |  | 1.8e-1 |  | 0.047 |  | 153.83 |  | 1.000 |  |  |  | N8(Deamidated): 100 |  |  |  |  |  |  |  |  |  | 2 |  | Sequest HT (3) |  | 3 |  | 509.97418 |  | -1.62 |  | 34.6290 |  | 8472 |  | 8472 |  | TRINITY\_DN0\_c1\_g1\_i15.p1; TRINITY\_DN0\_c1\_g1\_i23.p1; TRINITY\_DN0\_c1\_g1\_i11.p1; TRINITY\_DN0\_c1\_g1\_i10.p1 |  | Image |  | Peak List |  |
|  | | | | | | | | | | | | | | | | | | | | | | | | | | | | | | | | | | | | | | | | | | | | | | | | | | | | | |  |
|  | High |  | [R].GVLGTVKnLLIGAGK.[S] |  | HCD |  | N8(Deamidated) |  | 3.58 |  | 1.3e-3 |  | 2.5e-2 |  | 0.488 |  | 273.88 |  | 1.000 |  |  |  | N8(Deamidated): 100 |  |  |  |  |  |  |  |  |  | 2 |  | Sequest HT (3) |  | 3 |  | 480.96405 |  | -0.59 |  | 34.6584 |  | 8485 |  | 8485 |  | TRINITY\_DN0\_c1\_g1\_i15.p1; TRINITY\_DN0\_c1\_g1\_i23.p1; TRINITY\_DN0\_c1\_g1\_i11.p1; TRINITY\_DN0\_c1\_g1\_i10.p1 |  | Image |  | Peak List |  |
|  | | | | | | | | | | | | | | | | | | | | | | | | | | | | | | | | | | | | | | | | | | | | | | | | | | | | | |  |
|  | High |  | [R].GVLGTVKnLLIGAGK.[S] |  | HCD |  | N8(Deamidated) |  | 2.80 |  | 1.3e-3 |  | 7.9e-4 |  | 1.24 |  | 315.23 |  | 1.000 |  |  |  | N8(Deamidated): 100 |  |  |  |  |  |  |  |  |  | 2 |  | Sequest HT (3) |  | 2 |  | 720.94226 |  | -0.83 |  | 34.7028 |  | 8504 |  | 8504 |  | TRINITY\_DN0\_c1\_g1\_i15.p1; TRINITY\_DN0\_c1\_g1\_i23.p1; TRINITY\_DN0\_c1\_g1\_i11.p1; TRINITY\_DN0\_c1\_g1\_i10.p1 |  | Image |  | Peak List |  |
|  | | | | | | | | | | | | | | | | | | | | | | | | | | | | | | | | | | | | | | | | | | | | | | | | | | | | | |  |
|  | High |  | [R].GVLGTVKnLLIGAGK.[S] |  | HCD |  | N8(Deamidated) |  | 3.67 |  | 1.3e-3 |  | 6.3e-3 |  | 0.792 |  | 262.99 |  | 1.000 |  |  |  | N8(Deamidated): 100 |  |  |  |  |  |  |  |  |  | 2 |  | Sequest HT (3) |  | 3 |  | 480.96420 |  | -0.27 |  | 34.8048 |  | 8551 |  | 8551 |  | TRINITY\_DN0\_c1\_g1\_i15.p1; TRINITY\_DN0\_c1\_g1\_i23.p1; TRINITY\_DN0\_c1\_g1\_i11.p1; TRINITY\_DN0\_c1\_g1\_i10.p1 |  | Image |  | Peak List |  |
|  | | | | | | | | | | | | | | | | | | | | | | | | | | | | | | | | | | | | | | | | | | | | | | | | | | | | | |  |
|  | High |  | [R].GVLGTVKnLLIGAGKSAAQ.[S] |  | HCD |  | N8(Deamidated) |  | 3.85 |  | 1.3e-3 |  | 1.6e-6 |  | 2.59 |  | 502.68 |  | 1.000 |  |  |  | N8(Deamidated): 100 |  |  |  |  |  |  |  |  |  | 2 |  | Sequest HT (3) |  | 2 |  | 899.52441 |  | -0.96 |  | 34.8122 |  | 8555 |  | 8555 |  | TRINITY\_DN0\_c1\_g1\_i15.p1; TRINITY\_DN0\_c1\_g1\_i23.p1; TRINITY\_DN0\_c1\_g1\_i11.p1; TRINITY\_DN0\_c1\_g1\_i10.p1 |  | Image |  | Peak List |  |
|  | | | | | | | | | | | | | | | | | | | | | | | | | | | | | | | | | | | | | | | | | | | | | | | | | | | | | |  |
|  | High |  | [R].GVLGTVKnLLIGAGKSAAQ.[S] |  | HCD |  | N8(Deamidated) |  | 3.53 |  | 3.1e-3 |  | 1.2e-1 |  | 0.142 |  | 210.36 |  | 1.000 |  |  |  | N8(Deamidated): 100 |  |  |  |  |  |  |  |  |  | 2 |  | Sequest HT (3) |  | 3 |  | 600.01990 |  | 1.04 |  | 34.8572 |  | 8574 |  | 8574 |  | TRINITY\_DN0\_c1\_g1\_i15.p1; TRINITY\_DN0\_c1\_g1\_i23.p1; TRINITY\_DN0\_c1\_g1\_i11.p1; TRINITY\_DN0\_c1\_g1\_i10.p1 |  | Image |  | Peak List |  |
|  | | | | | | | | | | | | | | | | | | | | | | | | | | | | | | | | | | | | | | | | | | | | | | | | | | | | | |  |
|  | High |  | [R].GVLGTVKnLLIGAGKSAAQ.[S] |  | HCD |  | N8(Deamidated) |  | 3.53 |  | 1.3e-3 |  | 4.0e-4 |  | 1.39 |  | 351.96 |  | 1.000 |  |  |  | N8(Deamidated): 100 |  |  |  |  |  |  |  |  |  | 2 |  | Sequest HT (3) |  | 2 |  | 899.52618 |  | 1.01 |  | 34.9593 |  | 8616 |  | 8616 |  | TRINITY\_DN0\_c1\_g1\_i15.p1; TRINITY\_DN0\_c1\_g1\_i23.p1; TRINITY\_DN0\_c1\_g1\_i11.p1; TRINITY\_DN0\_c1\_g1\_i10.p1 |  | Image |  | Peak List |  |
|  | | | | | | | | | | | | | | | | | | | | | | | | | | | | | | | | | | | | | | | | | | | | | | | | | | | | | |  |
|  | High |  | [R].GVLGTVKnLLIGAGKSAAQ.[S] |  | HCD |  | N8(Deamidated) |  | 2.81 |  | 5.6e-3 |  | 2.1e-1 |  | 0.011 |  | 181.60 |  | 1.000 |  |  |  | N8(Deamidated): 100 |  |  |  |  |  |  |  |  |  | 2 |  | Sequest HT (3) |  | 3 |  | 600.02002 |  | 1.24 |  | 35.0037 |  | 8635 |  | 8635 |  | TRINITY\_DN0\_c1\_g1\_i15.p1; TRINITY\_DN0\_c1\_g1\_i23.p1; TRINITY\_DN0\_c1\_g1\_i11.p1; TRINITY\_DN0\_c1\_g1\_i10.p1 |  | Image |  | Peak List |  |
|  | | | | | | | | | | | | | | | | | | | | | | | | | | | | | | | | | | | | | | | | | | | | | | | | | | | | | |  |
|  | High |  | [R].GVLGTVKnLLIGAGKSAAQS.[V] |  | HCD |  | N8(Deamidated) |  | 3.29 |  | 3.1e-3 |  | 1.1e-1 |  | 0.152 |  | 216.56 |  | 1.000 |  |  |  | N8(Deamidated): 100 |  |  |  |  |  |  |  |  |  | 2 |  | Sequest HT (3) |  | 3 |  | 629.03064 |  | 1.09 |  | 35.0589 |  | 8660 |  | 8660 |  | TRINITY\_DN0\_c1\_g1\_i15.p1; TRINITY\_DN0\_c1\_g1\_i23.p1; TRINITY\_DN0\_c1\_g1\_i11.p1; TRINITY\_DN0\_c1\_g1\_i10.p1 |  | Image |  | Peak List |  |
|  | | | | | | | | | | | | | | | | | | | | | | | | | | | | | | | | | | | | | | | | | | | | | | | | | | | | | |  |
|  | High |  | [R].GVLGTVKnLLIGAGKSAAQS.[V] |  | HCD |  | N8(Deamidated) |  | 4.39 |  | 1.3e-3 |  | 2.3e-5 |  | 2.02 |  | 479.01 |  | 1.000 |  |  |  | N8(Deamidated): 100 |  |  |  |  |  |  |  |  |  | 2 |  | Sequest HT (3) |  | 2 |  | 943.04028 |  | -1.07 |  | 35.0658 |  | 8664 |  | 8664 |  | TRINITY\_DN0\_c1\_g1\_i15.p1; TRINITY\_DN0\_c1\_g1\_i23.p1; TRINITY\_DN0\_c1\_g1\_i11.p1; TRINITY\_DN0\_c1\_g1\_i10.p1 |  | Image |  | Peak List |  |
|  | | | | | | | | | | | | | | | | | | | | | | | | | | | | | | | | | | | | | | | | | | | | | | | | | | | | | |  |
|  | High |  | [R].GVLGTVKnLLIGAGKSAA.[QP] |  | HCD |  | N8(Deamidated) |  | 4.25 |  | 1.3e-3 |  | 2.5e-5 |  | 2 |  | 441.84 |  | 1.000 |  |  |  | N8(Deamidated): 100 |  |  |  |  |  |  |  |  |  | 2 |  | Sequest HT (3) |  | 2 |  | 835.49622 |  | 0.27 |  | 35.0873 |  | 8674 |  | 8674 |  | TRINITY\_DN0\_c1\_g1\_i15.p1; TRINITY\_DN0\_c1\_g1\_i23.p1; TRINITY\_DN0\_c1\_g1\_i11.p1; TRINITY\_DN0\_c1\_g1\_i10.p1 |  | Image |  | Peak List |  |
|  | | | | | | | | | | | | | | | | | | | | | | | | | | | | | | | | | | | | | | | | | | | | | | | | | | | | | |  |
|  | High |  | [R].GVLGTVKnLLIGAGKSAA.[QP] |  | HCD |  | N8(Deamidated) |  | 3.54 |  | 1.3e-3 |  | 1.4e-2 |  | 0.624 |  | 188.03 |  | 1.000 |  |  |  | N8(Deamidated): 100 |  |  |  |  |  |  |  |  |  | 2 |  | Sequest HT (3) |  | 3 |  | 557.33307 |  | -0.03 |  | 35.1010 |  | 8680 |  | 8680 |  | TRINITY\_DN0\_c1\_g1\_i15.p1; TRINITY\_DN0\_c1\_g1\_i23.p1; TRINITY\_DN0\_c1\_g1\_i11.p1; TRINITY\_DN0\_c1\_g1\_i10.p1 |  | Image |  | Peak List |  |
|  | | | | | | | | | | | | | | | | | | | | | | | | | | | | | | | | | | | | | | | | | | | | | | | | | | | | | |  |
|  | High |  | [R].GVLGTVKnLLIGAGKSA.[A] |  | HCD |  | N8(Deamidated) |  | 3.73 |  | 1.3e-3 |  | 5.9e-6 |  | 2.31 |  | 433.98 |  | 1.000 |  |  |  | N8(Deamidated): 100 |  |  |  |  |  |  |  |  |  | 2 |  | Sequest HT (3) |  | 2 |  | 799.97662 |  | -1.01 |  | 35.1096 |  | 8685 |  | 8685 |  | TRINITY\_DN0\_c1\_g1\_i15.p1; TRINITY\_DN0\_c1\_g1\_i23.p1; TRINITY\_DN0\_c1\_g1\_i11.p1; TRINITY\_DN0\_c1\_g1\_i10.p1 |  | Image |  | Peak List |  |
|  | | | | | | | | | | | | | | | | | | | | | | | | | | | | | | | | | | | | | | | | | | | | | | | | | | | | | |  |
|  | High |  | [R].GVLGTVKnLLIGAGKSA.[A] |  | HCD |  | N8(Deamidated) |  | 3.09 |  | 1.3e-3 |  | 6.3e-3 |  | 0.792 |  | 259.57 |  | 1.000 |  |  |  | N8(Deamidated): 100 |  |  |  |  |  |  |  |  |  | 2 |  | Sequest HT (3) |  | 3 |  | 533.65515 |  | 2.07 |  | 35.1437 |  | 8701 |  | 8701 |  | TRINITY\_DN0\_c1\_g1\_i15.p1; TRINITY\_DN0\_c1\_g1\_i23.p1; TRINITY\_DN0\_c1\_g1\_i11.p1; TRINITY\_DN0\_c1\_g1\_i10.p1 |  | Image |  | Peak List |  |
|  | | | | | | | | | | | | | | | | | | | | | | | | | | | | | | | | | | | | | | | | | | | | | | | | | | | | | |  |
|  | High |  | [R].GVLGTVKnLLIGAGKSA.[A] |  | HCD |  | N8(Deamidated) |  | 3.11 |  | 1.3e-3 |  | 4.2e-3 |  | 0.879 |  | 332.93 |  | 1.000 |  |  |  | N8(Deamidated): 100 |  |  |  |  |  |  |  |  |  | 2 |  | Sequest HT (3) |  | 2 |  | 799.97754 |  | 0.14 |  | 35.2533 |  | 8751 |  | 8751 |  | TRINITY\_DN0\_c1\_g1\_i15.p1; TRINITY\_DN0\_c1\_g1\_i23.p1; TRINITY\_DN0\_c1\_g1\_i11.p1; TRINITY\_DN0\_c1\_g1\_i10.p1 |  | Image |  | Peak List |  |
|  | | | | | | | | | | | | | | | | | | | | | | | | | | | | | | | | | | | | | | | | | | | | | | | | | | | | | |  |
|  | High |  | [R].GVLGTVKnLLIGAGKSAA.[QP] |  | HCD |  | N8(Deamidated) |  | 2.14 |  | 3.1e-3 |  | 1.2e-1 |  | 0.142 |  | 163.34 |  | 1.000 |  |  |  | N8(Deamidated): 100 |  |  |  |  |  |  |  |  |  | 2 |  | Sequest HT (3) |  | 3 |  | 557.33313 |  | 0.08 |  | 35.2546 |  | 8752 |  | 8752 |  | TRINITY\_DN0\_c1\_g1\_i15.p1; TRINITY\_DN0\_c1\_g1\_i23.p1; TRINITY\_DN0\_c1\_g1\_i11.p1; TRINITY\_DN0\_c1\_g1\_i10.p1 |  | Image |  | Peak List |  |
|  | | | | | | | | | | | | | | | | | | | | | | | | | | | | | | | | | | | | | | | | | | | | | | | | | | | | | |  |
|  | High |  | [R].GVLGTVKnLLIGAGKSA.[A] |  | HCD |  | N8(Deamidated) |  | 2.46 |  | 3.9e-3 |  | 1.8e-1 |  | 0.05 |  | 170.28 |  | 1.000 |  |  |  | N8(Deamidated): 100 |  |  |  |  |  |  |  |  |  | 2 |  | Sequest HT (3) |  | 3 |  | 533.65466 |  | 1.16 |  | 35.2924 |  | 8770 |  | 8770 |  | TRINITY\_DN0\_c1\_g1\_i15.p1; TRINITY\_DN0\_c1\_g1\_i23.p1; TRINITY\_DN0\_c1\_g1\_i11.p1; TRINITY\_DN0\_c1\_g1\_i10.p1 |  | Image |  | Peak List |  |
|  | | | | | | | | | | | | | | | | | | | | | | | | | | | | | | | | | | | | | | | | | | | | | | | | | | | | | |  |
|  | High |  | [K].SAAQSVLKTLScKLSNDc.[-] |  | HCD |  | C12(Dehydro); C18(Dehydro) |  | 4.40 |  | 1.3e-3 |  | 3.7e-3 |  | 0.906 |  | 350.81 |  | 1.000 |  |  |  | C12(Dehydro): 100; C18(Dehydro): 100 |  |  |  |  |  |  |  | 0.49 |  | 1 |  | Sequest HT (3) |  | 2 |  | 933.45862 |  | 0.21 |  | 35.8588 |  | 9040 |  | 9040 |  | TRINITY\_DN0\_c1\_g1\_i15.p1; TRINITY\_DN0\_c1\_g1\_i23.p1; TRINITY\_DN0\_c1\_g1\_i11.p1; TRINITY\_DN45\_c27\_g1\_i1.p1; TRINITY\_DN2658\_c0\_g2\_i1.p1\_ORF2; TRINITY\_DN0\_c1\_g1\_i10.p1; TRINITY\_DN2658\_c0\_g2\_i1.p1\_ORF1; TRINITY\_DN2658\_c0\_g2\_i1.p1 |  | Image |  | Peak List |  |
|  | | | | | | | | | | | | | | | | | | | | | | | | | | | | | | | | | | | | | | | | | | | | | | | | | | | | | |  |
|  | High |  | [K].SAAQSVLKTLScKLSNDc.[-] |  | HCD |  | C12(Dehydro); C18(Dehydro) |  | 2.79 |  | 3.1e-3 |  | 1.1e-1 |  | 0.155 |  | 206.05 |  | 1.000 |  |  |  | C12(Dehydro): 100; C18(Dehydro): 100 |  |  |  |  |  |  |  | 0.51 |  | 1 |  | Sequest HT (3) |  | 3 |  | 622.64099 |  | -0.61 |  | 35.8729 |  | 9047 |  | 9047 |  | TRINITY\_DN0\_c1\_g1\_i15.p1; TRINITY\_DN0\_c1\_g1\_i23.p1; TRINITY\_DN0\_c1\_g1\_i11.p1; TRINITY\_DN45\_c27\_g1\_i1.p1; TRINITY\_DN2658\_c0\_g2\_i1.p1\_ORF2; TRINITY\_DN0\_c1\_g1\_i10.p1; TRINITY\_DN2658\_c0\_g2\_i1.p1\_ORF1; TRINITY\_DN2658\_c0\_g2\_i1.p1 |  | Image |  | Peak List |  |
|  | | | | | | | | | | | | | | | | | | | | | | | | | | | | | | | | | | | | | | | | | | | | | | | | | | | | | |  |
|  | High |  | [K].SAAQSVLKTLScKLSNDc.[-] |  | HCD |  | C12(Dehydro); C18(Dehydro) |  | 2.92 |  | 1.3e-3 |  | 4.5e-2 |  | 0.363 |  | 216.35 |  | 1.000 |  |  |  | C12(Dehydro): 100; C18(Dehydro): 100 |  |  |  |  |  |  |  | 0.52 |  | 1 |  | Sequest HT (3) |  | 3 |  | 622.64154 |  | 0.27 |  | 36.0172 |  | 9114 |  | 9114 |  | TRINITY\_DN0\_c1\_g1\_i15.p1; TRINITY\_DN0\_c1\_g1\_i23.p1; TRINITY\_DN0\_c1\_g1\_i11.p1; TRINITY\_DN45\_c27\_g1\_i1.p1; TRINITY\_DN2658\_c0\_g2\_i1.p1\_ORF2; TRINITY\_DN0\_c1\_g1\_i10.p1; TRINITY\_DN2658\_c0\_g2\_i1.p1\_ORF1; TRINITY\_DN2658\_c0\_g2\_i1.p1 |  | Image |  | Peak List |  |
|  | | | | | | | | | | | | | | | | | | | | | | | | | | | | | | | | | | | | | | | | | | | | | | | | | | | | | |  |
|  | High |  | [K].SAAQSVLKTLScKLSNDc.[-] |  | HCD |  | C12(Dehydro); C18(Dehydro) |  | 2.87 |  | 1.3e-3 |  | 4.0e-2 |  | 0.385 |  | 182.50 |  | 1.000 |  |  |  | C12(Dehydro): 100; C18(Dehydro): 100 |  |  |  |  |  |  |  | 0.63 |  | 1 |  | Sequest HT (3) |  | 2 |  | 933.45795 |  | -0.50 |  | 36.0198 |  | 9116 |  | 9116 |  | TRINITY\_DN0\_c1\_g1\_i15.p1; TRINITY\_DN0\_c1\_g1\_i23.p1; TRINITY\_DN0\_c1\_g1\_i11.p1; TRINITY\_DN45\_c27\_g1\_i1.p1; TRINITY\_DN2658\_c0\_g2\_i1.p1\_ORF2; TRINITY\_DN0\_c1\_g1\_i10.p1; TRINITY\_DN2658\_c0\_g2\_i1.p1\_ORF1; TRINITY\_DN2658\_c0\_g2\_i1.p1 |  | Image |  | Peak List |  |
|  | | | | | | | | | | | | | | | | | | | | | | | | | | | | | | | | | | | | | | | | | | | | | | | | | | | | | |  |
|  | High |  | [K].SAAQSVLKTLScKLSnDc.[-] |  | HCD |  | C12(Dehydro); N16(Deamidated); C18(Dehydro) |  | 3.98 |  | 1.3e-3 |  | 5.1e-2 |  | 0.332 |  | 248.98 |  | 1.000 |  |  |  | C12(Dehydro): 100; N16(Deamidated): 100; C18(Dehydro): 100 |  |  |  |  |  |  |  | 0.55 |  | 1 |  | Sequest HT (3) |  | 3 |  | 622.96918 |  | -0.32 |  | 36.1850 |  | 9196 |  | 9196 |  | TRINITY\_DN0\_c1\_g1\_i15.p1; TRINITY\_DN0\_c1\_g1\_i23.p1; TRINITY\_DN0\_c1\_g1\_i11.p1; TRINITY\_DN45\_c27\_g1\_i1.p1; TRINITY\_DN2658\_c0\_g2\_i1.p1\_ORF2; TRINITY\_DN0\_c1\_g1\_i10.p1; TRINITY\_DN2658\_c0\_g2\_i1.p1\_ORF1; TRINITY\_DN2658\_c0\_g2\_i1.p1 |  | Image |  | Peak List |  |
|  | | | | | | | | | | | | | | | | | | | | | | | | | | | | | | | | | | | | | | | | | | | | | | | | | | | | | |  |
|  | High |  | [K].SAAQSVLKTLScKLSnDc.[-] |  | HCD |  | C12(Dehydro); N16(Deamidated); C18(Dehydro) |  | 4.33 |  | 1.3e-3 |  | 1.3e-3 |  | 1.13 |  | 325.14 |  | 1.000 |  |  |  | C12(Dehydro): 100; N16(Deamidated): 100; C18(Dehydro): 100 |  |  |  |  |  |  |  | 0.57 |  | 1 |  | Sequest HT (3) |  | 2 |  | 933.95013 |  | -0.31 |  | 36.1949 |  | 9203 |  | 9203 |  | TRINITY\_DN0\_c1\_g1\_i15.p1; TRINITY\_DN0\_c1\_g1\_i23.p1; TRINITY\_DN0\_c1\_g1\_i11.p1; TRINITY\_DN45\_c27\_g1\_i1.p1; TRINITY\_DN2658\_c0\_g2\_i1.p1\_ORF2; TRINITY\_DN0\_c1\_g1\_i10.p1; TRINITY\_DN2658\_c0\_g2\_i1.p1\_ORF1; TRINITY\_DN2658\_c0\_g2\_i1.p1 |  | Image |  | Peak List |  |
|  | | | | | | | | | | | | | | | | | | | | | | | | | | | | | | | | | | | | | | | | | | | | | | | | | | | | | |  |
|  | High |  | [R].GVLGTVKNLLIGA.[GS] |  | HCD |  |  |  | 2.86 |  | 1.3e-3 |  | 6.6e-2 |  | 0.275 |  |  |  |  |  |  |  |  |  |  |  |  |  |  |  | 0.33 |  | 1 |  | Sequest HT (3) |  | 2 |  | 627.89160 |  | -1.65 |  | 36.4079 |  | 9308 |  | 9308 |  | TRINITY\_DN0\_c1\_g1\_i15.p1; TRINITY\_DN0\_c1\_g1\_i23.p1; TRINITY\_DN0\_c1\_g1\_i11.p1; TRINITY\_DN2658\_c0\_g2\_i1.p1\_ORF2; TRINITY\_DN0\_c1\_g1\_i10.p1; TRINITY\_DN2658\_c0\_g2\_i1.p1\_ORF1; TRINITY\_DN2658\_c0\_g2\_i1.p1 |  | Image |  | Peak List |  |
|  | | | | | | | | | | | | | | | | | | | | | | | | | | | | | | | | | | | | | | | | | | | | | | | | | | | | | |  |
|  | High |  | [R].GVLGTVKNLLIGAGKSAAQSVLK.[T] |  | HCD |  |  |  | 6.71 |  | 1.3e-3 |  | 1.8e-5 |  | 2.07 |  |  |  |  |  |  |  |  |  |  |  |  |  |  |  | 0.72 |  | 1 |  | Sequest HT (3) |  | 3 |  | 742.11755 |  | -0.28 |  | 37.3678 |  | 9773 |  | 9773 |  | TRINITY\_DN0\_c1\_g1\_i15.p1; TRINITY\_DN0\_c1\_g1\_i23.p1; TRINITY\_DN0\_c1\_g1\_i11.p1; TRINITY\_DN0\_c1\_g1\_i10.p1 |  | Image |  | Peak List |  |
|  | | | | | | | | | | | | | | | | | | | | | | | | | | | | | | | | | | | | | | | | | | | | | | | | | | | | | |  |
|  | High |  | [R].GVLGTVKNLLIGAGKSAAQSVLK.[T] |  | HCD |  |  |  | 4.46 |  | 1.3e-3 |  | 4.5e-4 |  | 1.37 |  |  |  |  |  |  |  |  |  |  |  |  |  |  |  | 0.59 |  | 1 |  | Sequest HT (3) |  | 4 |  | 556.84009 |  | -0.09 |  | 37.3741 |  | 9778 |  | 9778 |  | TRINITY\_DN0\_c1\_g1\_i15.p1; TRINITY\_DN0\_c1\_g1\_i23.p1; TRINITY\_DN0\_c1\_g1\_i11.p1; TRINITY\_DN0\_c1\_g1\_i10.p1 |  | Image |  | Peak List |  |
|  | | | | | | | | | | | | | | | | | | | | | | | | | | | | | | | | | | | | | | | | | | | | | | | | | | | | | |  |
|  | High |  | [R].GVLGTVKNLLIGAGKSAAQSVLK.[T] |  | HCD |  |  |  | 3.83 |  | 1.3e-3 |  | 1.0e-2 |  | 0.681 |  |  |  |  |  |  |  |  |  |  |  |  |  |  |  | 0.59 |  | 1 |  | Sequest HT (3) |  | 2 |  | 1112.66992 |  | -2.77 |  | 37.4497 |  | 9820 |  | 9820 |  | TRINITY\_DN0\_c1\_g1\_i15.p1; TRINITY\_DN0\_c1\_g1\_i23.p1; TRINITY\_DN0\_c1\_g1\_i11.p1; TRINITY\_DN0\_c1\_g1\_i10.p1 |  | Image |  | Peak List |  |
|  | | | | | | | | | | | | | | | | | | | | | | | | | | | | | | | | | | | | | | | | | | | | | | | | | | | | | |  |
|  | High |  | [R].GVLGTVKnLLIGAG.[K] |  | HCD |  | N8(Deamidated) |  | 2.80 |  | 3.9e-3 |  | 1.5e-1 |  | 0.089 |  | 210.85 |  | 1.000 |  |  |  | N8(Deamidated): 100 |  |  |  |  |  |  |  |  |  | 2 |  | Sequest HT (3) |  | 2 |  | 656.89557 |  | 0.29 |  | 37.4941 |  | 9842 |  | 9842 |  | TRINITY\_DN0\_c1\_g1\_i15.p1; TRINITY\_DN0\_c1\_g1\_i23.p1; TRINITY\_DN0\_c1\_g1\_i11.p1; TRINITY\_DN0\_c1\_g1\_i10.p1 |  | Image |  | Peak List |  |
|  | | | | | | | | | | | | | | | | | | | | | | | | | | | | | | | | | | | | | | | | | | | | | | | | | | | | | |  |
|  | High |  | [R].GVLGTVKnLLIGAG.[K] |  | HCD |  | N8(Deamidated) |  | 2.56 |  | 1.3e-3 |  | 6.7e-2 |  | 0.273 |  | 192.11 |  | 1.000 |  |  |  | N8(Deamidated): 100 |  |  |  |  |  |  |  |  |  | 2 |  | Sequest HT (3) |  | 2 |  | 656.89484 |  | -0.82 |  | 37.6335 |  | 9906 |  | 9906 |  | TRINITY\_DN0\_c1\_g1\_i15.p1; TRINITY\_DN0\_c1\_g1\_i23.p1; TRINITY\_DN0\_c1\_g1\_i11.p1; TRINITY\_DN0\_c1\_g1\_i10.p1 |  | Image |  | Peak List |  |
|  | | | | | | | | | | | | | | | | | | | | | | | | | | | | | | | | | | | | | | | | | | | | | | | | | | | | | |  |
|  | High |  | [ND].LLIGAGKSAAQSVLKTLScKLSNDc.[-] |  | HCD |  | C19(Dehydro); C25(Dehydro) |  | 4.09 |  | 1.3e-3 |  | 2.4e-2 |  | 0.498 |  | 264.63 |  | 1.000 |  |  |  | C19(Dehydro): 100; C25(Dehydro): 100 |  |  |  |  |  |  |  | 0.61 |  | 1 |  | Sequest HT (3) |  | 3 |  | 840.11572 |  | -1.64 |  | 38.1481 |  | 10158 |  | 10158 |  | TRINITY\_DN0\_c1\_g1\_i15.p1; TRINITY\_DN0\_c1\_g1\_i23.p1; TRINITY\_DN0\_c1\_g1\_i11.p1; TRINITY\_DN45\_c27\_g1\_i1.p1; TRINITY\_DN0\_c1\_g1\_i10.p1 |  | Image |  | Peak List |  |
|  | | | | | | | | | | | | | | | | | | | | | | | | | | | | | | | | | | | | | | | | | | | | | | | | | | | | | |  |
|  | High |  | [ND].LLIGAGKSAAQSVLKTLScKLSNDc.[-] |  | HCD |  | C19(Dehydro); C25(Dehydro) |  | 4.88 |  | 1.3e-3 |  | 1.3e-4 |  | 1.64 |  | 486.12 |  | 1.000 |  |  |  | C19(Dehydro): 100; C25(Dehydro): 100 |  |  |  |  |  |  |  | 0.66 |  | 1 |  | Sequest HT (3) |  | 2 |  | 1259.67114 |  | -0.69 |  | 38.1890 |  | 10177 |  | 10177 |  | TRINITY\_DN0\_c1\_g1\_i15.p1; TRINITY\_DN0\_c1\_g1\_i23.p1; TRINITY\_DN0\_c1\_g1\_i11.p1; TRINITY\_DN45\_c27\_g1\_i1.p1; TRINITY\_DN0\_c1\_g1\_i10.p1 |  | Image |  | Peak List |  |
|  | | | | | | | | | | | | | | | | | | | | | | | | | | | | | | | | | | | | | | | | | | | | | | | | | | | | | |  |
|  | High |  | [ND].LLIGAGKSAAQSVLKTLScKLSNDc.[-] |  | HCD |  | C19(Dehydro); C25(Dehydro) |  | 2.95 |  | 3.9e-3 |  | 1.7e-1 |  | 0.065 |  | 131.33 |  | 1.000 |  |  |  | C19(Dehydro): 100; C25(Dehydro): 100 |  |  |  |  |  |  |  | 0.46 |  | 1 |  | Sequest HT (3) |  | 4 |  | 630.33923 |  | -0.66 |  | 38.2519 |  | 10206 |  | 10206 |  | TRINITY\_DN0\_c1\_g1\_i15.p1; TRINITY\_DN0\_c1\_g1\_i23.p1; TRINITY\_DN0\_c1\_g1\_i11.p1; TRINITY\_DN45\_c27\_g1\_i1.p1; TRINITY\_DN0\_c1\_g1\_i10.p1 |  | Image |  | Peak List |  |
|  | | | | | | | | | | | | | | | | | | | | | | | | | | | | | | | | | | | | | | | | | | | | | | | | | | | | | |  |
|  | High |  | [ND].LLIGAGKSAAQSVLKTLScKLSNDc.[-] |  | HCD |  | C19(Dehydro); C25(Dehydro) |  | 4.05 |  | 1.3e-3 |  | 2.7e-2 |  | 0.473 |  | 257.24 |  | 1.000 |  |  |  | C19(Dehydro): 100; C25(Dehydro): 100 |  |  |  |  |  |  |  | 0.56 |  | 1 |  | Sequest HT (3) |  | 3 |  | 840.11639 |  | -0.84 |  | 38.2918 |  | 10226 |  | 10226 |  | TRINITY\_DN0\_c1\_g1\_i15.p1; TRINITY\_DN0\_c1\_g1\_i23.p1; TRINITY\_DN0\_c1\_g1\_i11.p1; TRINITY\_DN45\_c27\_g1\_i1.p1; TRINITY\_DN0\_c1\_g1\_i10.p1 |  | Image |  | Peak List |  |
|  | | | | | | | | | | | | | | | | | | | | | | | | | | | | | | | | | | | | | | | | | | | | | | | | | | | | | |  |
|  | High |  | [ND].LLIGAGKSAAQSVLKTLScKLSnDc.[-] |  | HCD |  | C19(Dehydro); N23(Deamidated); C25(Dehydro) |  | 4.78 |  | 1.3e-3 |  | 4.0e-5 |  | 1.9 |  | 332.04 |  | 1.000 |  |  |  | C19(Dehydro): 100; N23(Deamidated): 100; C25(Dehydro): 100 |  |  |  |  |  |  |  | 0.65 |  | 1 |  | Sequest HT (3) |  | 3 |  | 840.44452 |  | -0.70 |  | 38.4435 |  | 10306 |  | 10306 |  | TRINITY\_DN0\_c1\_g1\_i15.p1; TRINITY\_DN0\_c1\_g1\_i23.p1; TRINITY\_DN0\_c1\_g1\_i11.p1; TRINITY\_DN45\_c27\_g1\_i1.p1; TRINITY\_DN0\_c1\_g1\_i10.p1 |  | Image |  | Peak List |  |
|  | | | | | | | | | | | | | | | | | | | | | | | | | | | | | | | | | | | | | | | | | | | | | | | | | | | | | |  |
|  | High |  | [R].GVLGTVKnLLIGAGKSAAQSVLK.[T] |  | HCD |  | N8(Deamidated) |  | 6.42 |  | 1.3e-3 |  | 1.5e-4 |  | 1.61 |  | 443.14 |  | 1.000 |  |  |  | N8(Deamidated): 100 |  |  |  |  |  |  |  |  |  | 2 |  | Sequest HT (3) |  | 3 |  | 742.44489 |  | -1.19 |  | 38.4473 |  | 10309 |  | 10309 |  | TRINITY\_DN0\_c1\_g1\_i15.p1; TRINITY\_DN0\_c1\_g1\_i23.p1; TRINITY\_DN0\_c1\_g1\_i11.p1; TRINITY\_DN0\_c1\_g1\_i10.p1 |  | Image |  | Peak List |  |
|  | | | | | | | | | | | | | | | | | | | | | | | | | | | | | | | | | | | | | | | | | | | | | | | | | | | | | |  |
|  | High |  | [ND].LLIGAGKSAAQSVLKTLScKLSnDc.[-] |  | HCD |  | C19(Dehydro); N23(Deamidated); C25(Dehydro) |  | 3.16 |  | 1.3e-3 |  | 2.3e-2 |  | 0.512 |  | 166.10 |  | 1.000 |  |  |  | C19(Dehydro): 100; N23(Deamidated): 100; C25(Dehydro): 100 |  |  |  |  |  |  |  | 0.43 |  | 1 |  | Sequest HT (3) |  | 4 |  | 630.58521 |  | -0.71 |  | 38.4726 |  | 10321 |  | 10321 |  | TRINITY\_DN0\_c1\_g1\_i15.p1; TRINITY\_DN0\_c1\_g1\_i23.p1; TRINITY\_DN0\_c1\_g1\_i11.p1; TRINITY\_DN45\_c27\_g1\_i1.p1; TRINITY\_DN0\_c1\_g1\_i10.p1 |  | Image |  | Peak List |  |
|  | | | | | | | | | | | | | | | | | | | | | | | | | | | | | | | | | | | | | | | | | | | | | | | | | | | | | |  |
|  | High |  | [R].GVLGTVKnLLIGAGKSAAQSVLK.[T] |  | HCD |  | N8(Deamidated) |  | 4.76 |  | 1.3e-3 |  | 2.3e-3 |  | 1.01 |  | 286.69 |  | 1.000 |  |  |  | N8(Deamidated): 100 |  |  |  |  |  |  |  |  |  | 2 |  | Sequest HT (3) |  | 4 |  | 557.08563 |  | -0.92 |  | 38.4839 |  | 10325 |  | 10325 |  | TRINITY\_DN0\_c1\_g1\_i15.p1; TRINITY\_DN0\_c1\_g1\_i23.p1; TRINITY\_DN0\_c1\_g1\_i11.p1; TRINITY\_DN0\_c1\_g1\_i10.p1 |  | Image |  | Peak List |  |
|  | | | | | | | | | | | | | | | | | | | | | | | | | | | | | | | | | | | | | | | | | | | | | | | | | | | | | |  |
|  | High |  | [R].GVLGTVKnLLIGAGKSAAQSVLK.[T] |  | HCD |  | N8(Deamidated) |  | 5.77 |  | 1.3e-3 |  | 9.7e-7 |  | 2.7 |  | 508.16 |  | 1.000 |  |  |  | N8(Deamidated): 100 |  |  |  |  |  |  |  |  |  | 2 |  | Sequest HT (3) |  | 2 |  | 1113.16528 |  | 0.25 |  | 38.5336 |  | 10347 |  | 10347 |  | TRINITY\_DN0\_c1\_g1\_i15.p1; TRINITY\_DN0\_c1\_g1\_i23.p1; TRINITY\_DN0\_c1\_g1\_i11.p1; TRINITY\_DN0\_c1\_g1\_i10.p1 |  | Image |  | Peak List |  |
|  | | | | | | | | | | | | | | | | | | | | | | | | | | | | | | | | | | | | | | | | | | | | | | | | | | | | | |  |
|  | High |  | [R].GVLGTVKnLLIGAGKSAAQSVLK.[T] |  | HCD |  | N8(Deamidated) |  | 5.58 |  | 1.3e-3 |  | 1.3e-4 |  | 1.63 |  | 350.19 |  | 1.000 |  |  |  | N8(Deamidated): 100 |  |  |  |  |  |  |  |  |  | 2 |  | Sequest HT (3) |  | 3 |  | 742.44672 |  | 1.28 |  | 38.5887 |  | 10372 |  | 10372 |  | TRINITY\_DN0\_c1\_g1\_i15.p1; TRINITY\_DN0\_c1\_g1\_i23.p1; TRINITY\_DN0\_c1\_g1\_i11.p1; TRINITY\_DN0\_c1\_g1\_i10.p1 |  | Image |  | Peak List |  |
|  | | | | | | | | | | | | | | | | | | | | | | | | | | | | | | | | | | | | | | | | | | | | | | | | | | | | | |  |
|  | High |  | [ND].LLIGAGKSAAQSVLKTLScKLSnDc.[-] |  | HCD |  | C19(Dehydro); N23(Deamidated); C25(Dehydro) |  | 3.34 |  | 1.3e-3 |  | 6.6e-2 |  | 0.275 |  | 231.44 |  | 1.000 |  |  |  | C19(Dehydro): 100; N23(Deamidated): 100; C25(Dehydro): 100 |  |  |  |  |  |  |  | 0.50 |  | 1 |  | Sequest HT (3) |  | 3 |  | 840.44550 |  | 0.46 |  | 38.5924 |  | 10375 |  | 10375 |  | TRINITY\_DN0\_c1\_g1\_i15.p1; TRINITY\_DN0\_c1\_g1\_i23.p1; TRINITY\_DN0\_c1\_g1\_i11.p1; TRINITY\_DN45\_c27\_g1\_i1.p1; TRINITY\_DN0\_c1\_g1\_i10.p1 |  | Image |  | Peak List |  |
|  | | | | | | | | | | | | | | | | | | | | | | | | | | | | | | | | | | | | | | | | | | | | | | | | | | | | | |  |
|  | High |  | [ND].LLIGAGKSAAQSVLKTLScKLSnDc.[-] |  | HCD |  | C19(Dehydro); N23(Deamidated); C25(Dehydro) |  | 3.69 |  | 3.1e-3 |  | 1.2e-1 |  | 0.141 |  | 123.55 |  | 1.000 |  |  |  | C19(Dehydro): 100; N23(Deamidated): 100; C25(Dehydro): 100 |  |  |  |  |  |  |  | 0.49 |  | 1 |  | Sequest HT (3) |  | 4 |  | 630.58655 |  | 1.43 |  | 38.6126 |  | 10384 |  | 10384 |  | TRINITY\_DN0\_c1\_g1\_i15.p1; TRINITY\_DN0\_c1\_g1\_i23.p1; TRINITY\_DN0\_c1\_g1\_i11.p1; TRINITY\_DN45\_c27\_g1\_i1.p1; TRINITY\_DN0\_c1\_g1\_i10.p1 |  | Image |  | Peak List |  |
|  | | | | | | | | | | | | | | | | | | | | | | | | | | | | | | | | | | | | | | | | | | | | | | | | | | | | | |  |
|  | High |  | [G].TVKNLLIGAGKSAAQSVLKTLScKLSNDc.[-] |  | HCD |  | C23(Dehydro); C29(Dehydro) |  | 5.11 |  | 1.3e-3 |  | 1.6e-2 |  | 0.588 |  | 220.11 |  | 1.000 |  |  |  | C23(Dehydro): 100; C29(Dehydro): 100 |  |  |  |  |  |  |  | 0.52 |  | 1 |  | Sequest HT (3) |  | 4 |  | 740.90308 |  | -0.09 |  | 39.1295 |  | 10641 |  | 10641 |  | TRINITY\_DN0\_c1\_g1\_i15.p1; TRINITY\_DN0\_c1\_g1\_i23.p1; TRINITY\_DN0\_c1\_g1\_i11.p1; TRINITY\_DN0\_c1\_g1\_i10.p1 |  | Image |  | Peak List |  |
|  | | | | | | | | | | | | | | | | | | | | | | | | | | | | | | | | | | | | | | | | | | | | | | | | | | | | | |  |
|  | High |  | [G].TVKNLLIGAGKSAAQSVLKTLScKLSNDc.[-] |  | HCD |  | C23(Dehydro); C29(Dehydro) |  | 3.21 |  | 3.1e-3 |  | 9.4e-2 |  | 0.196 |  | 128.10 |  | 1.000 |  |  |  | C23(Dehydro): 100; C29(Dehydro): 100 |  |  |  |  |  |  |  | 0.46 |  | 1 |  | Sequest HT (3) |  | 5 |  | 592.92316 |  | -1.37 |  | 39.2413 |  | 10699 |  | 10699 |  | TRINITY\_DN0\_c1\_g1\_i15.p1; TRINITY\_DN0\_c1\_g1\_i23.p1; TRINITY\_DN0\_c1\_g1\_i11.p1; TRINITY\_DN0\_c1\_g1\_i10.p1 |  | Image |  | Peak List |  |
|  | | | | | | | | | | | | | | | | | | | | | | | | | | | | | | | | | | | | | | | | | | | | | | | | | | | | | |  |
|  | High |  | [G].TVKNLLIGAGKSAAQSVLKTLScKLSnDc.[-] |  | HCD |  | C23(Dehydro); N27(Deamidated); C29(Dehydro) |  | 3.82 |  | 1.3e-3 |  | 1.8e-2 |  | 0.565 |  | 195.15 |  | 1.000 |  | b1+(4): 443.26; b1+(5): 556.35; b1+(6): 669.43; b1+(7): 782.51; y1+(14): 1509.73; y1+(12): 1323.63; y1+(11): 1210.54; y1+(10): 1082.45; y1+(9): 981.40; y1+(8): 868.32; y1+(7): 781.29; b2+(16): 770.45; y2+(23): 1147.08; y2+(22): 1090.54; y2+(20): 1026.51; y2+(18): 933.95 |  | C23(Dehydro): 100; N27(Deamidated): 100; C29(Dehydro): 100 |  |  |  |  |  |  |  | 0.52 |  | 1 |  | Sequest HT (3) |  | 4 |  | 741.14929 |  | 0.20 |  | 39.3649 |  | 10758 |  | 10758 |  | TRINITY\_DN0\_c1\_g1\_i15.p1; TRINITY\_DN0\_c1\_g1\_i23.p1; TRINITY\_DN0\_c1\_g1\_i11.p1; TRINITY\_DN0\_c1\_g1\_i10.p1 |  | Image |  | Peak List |  |
|  | | | | | | | | | | | | | | | | | | | | | | | | | | | | | | | | | | | | | | | | | | | | | | | | | | | | | |  |
|  | High |  | [ND].LLIGAGKSAAQSVLKTLScKLSnDc.[-] |  | HCD |  | C19(Dehydro); N23(Deamidated); C25(Dehydro) |  | 3.01 |  | 1.3e-3 |  | 2.5e-2 |  | 0.493 |  | 245.11 |  | 1.000 |  |  |  | C19(Dehydro): 100; N23(Deamidated): 100; C25(Dehydro): 100 |  |  |  |  |  |  |  | 0.28 |  | 1 |  | Sequest HT (3) |  | 3 |  | 840.44586 |  | 0.90 |  | 39.4619 |  | 10802 |  | 10802 |  | TRINITY\_DN0\_c1\_g1\_i15.p1; TRINITY\_DN0\_c1\_g1\_i23.p1; TRINITY\_DN0\_c1\_g1\_i11.p1; TRINITY\_DN45\_c27\_g1\_i1.p1; TRINITY\_DN0\_c1\_g1\_i10.p1 |  | Image |  | Peak List |  |
|  | | | | | | | | | | | | | | | | | | | | | | | | | | | | | | | | | | | | | | | | | | | | | | | | | | | | | |  |
|  | High |  | [R].GVLGTVKnLLIGAGKSAAQSVL.[K] |  | HCD |  | N8(Deamidated) |  | 4.93 |  | 1.3e-3 |  | 5.3e-4 |  | 1.33 |  | 329.23 |  | 1.000 |  |  |  | N8(Deamidated): 100 |  |  |  |  |  |  |  |  |  | 2 |  | Sequest HT (3) |  | 2 |  | 1049.11829 |  | 0.72 |  | 39.7878 |  | 10970 |  | 10970 |  | TRINITY\_DN0\_c1\_g1\_i15.p1; TRINITY\_DN0\_c1\_g1\_i23.p1; TRINITY\_DN0\_c1\_g1\_i11.p1; TRINITY\_DN0\_c1\_g1\_i10.p1 |  | Image |  | Peak List |  |
|  | | | | | | | | | | | | | | | | | | | | | | | | | | | | | | | | | | | | | | | | | | | | | | | | | | | | | |  |
|  | High |  | [G].TVKnLLIGAGKSAAQSVLKTLScKLSNDc.[-] |  | HCD |  | N4(Deamidated); C23(Dehydro); C29(Dehydro) |  | 5.67 |  | 1.3e-3 |  | 2.7e-3 |  | 0.977 |  | 195.05 |  | 1.000 |  | b1+(4): 444.25; b1+(5): 557.33; b1+(6): 670.41; b1+(7): 783.50; y1+(13): 1421.71; y1+(12): 1322.64; y1+(11): 1209.56; y1+(10): 1081.47; y1+(9): 980.42; y1+(8): 867.33; y1+(7): 780.30; b2+(17): 820.47; y2+(22): 1090.05; y2+(20): 1026.02 |  | N4(Deamidated): 100; C23(Dehydro): 100; C29(Dehydro): 100 |  |  |  |  |  |  |  |  |  | 2 |  | Sequest HT (3) |  | 4 |  | 741.14923 |  | 0.12 |  | 41.4433 |  | 11741 |  | 11741 |  | TRINITY\_DN0\_c1\_g1\_i15.p1; TRINITY\_DN0\_c1\_g1\_i23.p1; TRINITY\_DN0\_c1\_g1\_i11.p1; TRINITY\_DN0\_c1\_g1\_i10.p1 |  | Image |  | Peak List |  |
|  | | | | | | | | | | | | | | | | | | | | | | | | | | | | | | | | | | | | | | | | | | | | | | | | | | | | | |  |
|  | High |  | [G].TVKnLLIGAGKSAAQSVLKTLScKLSNDc.[-] |  | HCD |  | N4(Deamidated); C23(Dehydro); C29(Dehydro) |  | 2.73 |  | 2.4e-3 |  | 8.0e-2 |  | 0.231 |  | 160.76 |  | 1.000 |  | b1+(4): 444.25; b1+(5): 557.33; b1+(6): 670.41; b1+(7): 783.50; y1+(12): 1322.64; y1+(11): 1209.56; y1+(10): 1081.47; y1+(9): 980.42; y1+(8): 867.33; b2+(16): 770.94; y2+(22): 1090.05; y2+(20): 1026.02; y2+(11): 605.28 |  | N4(Deamidated): 100; C23(Dehydro): 100; C29(Dehydro): 100 |  |  |  |  |  |  |  |  |  | 2 |  | Sequest HT (3) |  | 4 |  | 741.14801 |  | -1.53 |  | 41.5902 |  | 11812 |  | 11812 |  | TRINITY\_DN0\_c1\_g1\_i15.p1; TRINITY\_DN0\_c1\_g1\_i23.p1; TRINITY\_DN0\_c1\_g1\_i11.p1; TRINITY\_DN0\_c1\_g1\_i10.p1 |  | Image |  | Peak List |  |
|  | | | | | | | | | | | | | | | | | | | | | | | | | | | | | | | | | | | | | | | | | | | | | | | | | | | | | |  |
|  | High |  | [L].GTVKnLLIGAGKSAAQSVLKTLScKLSNDc.[-] |  | HCD |  | N5(Deamidated); C24(Dehydro); C30(Dehydro) |  | 4.17 |  | 1.3e-3 |  | 5.1e-3 |  | 0.837 |  | 257.11 |  | 1.000 |  | b1+(5): 501.27; b1+(6): 614.35; b1+(7): 727.43; b1+(8): 840.52; b1+(9): 897.54; b1+(10): 968.58; b1+(11): 1025.60; y1+(12): 1322.64; y1+(11): 1209.56; y1+(10): 1081.47; y1+(9): 980.42; y1+(8): 867.33; y1+(7): 780.30; b2+(14): 656.39; b2+(15): 691.90; b2+(19): 905.53; b2+(20): 969.57; y2+(22): 1090.05; y2+(20): 1026.02; y2+(18): 933.46; y2+(11): 605.28 |  | N5(Deamidated): 100; C24(Dehydro): 100; C30(Dehydro): 100 |  |  |  |  |  |  |  |  |  | 2 |  | Sequest HT (3) |  | 4 |  | 755.40430 |  | -0.28 |  | 41.8538 |  | 11933 |  | 11933 |  | TRINITY\_DN0\_c1\_g1\_i15.p1; TRINITY\_DN0\_c1\_g1\_i23.p1; TRINITY\_DN0\_c1\_g1\_i11.p1; TRINITY\_DN0\_c1\_g1\_i10.p1 |  | Image |  | Peak List |  |
|  | | | | | | | | | | | | | | | | | | | | | | | | | | | | | | | | | | | | | | | | | | | | | | | | | | | | | |  |
|  | High |  | [R].GVLGTVKnLLIGAGKSAAQSVLKTL.[S] |  | HCD |  | N8(Deamidated) |  | 4.26 |  | 1.3e-3 |  | 1.2e-2 |  | 0.651 |  | 299.09 |  | 1.000 |  |  |  | N8(Deamidated): 100 |  |  |  |  |  |  |  |  |  | 2 |  | Sequest HT (3) |  | 3 |  | 813.82111 |  | -2.35 |  | 42.0866 |  | 12040 |  | 12040 |  | TRINITY\_DN0\_c1\_g1\_i15.p1; TRINITY\_DN0\_c1\_g1\_i23.p1; TRINITY\_DN0\_c1\_g1\_i11.p1; TRINITY\_DN0\_c1\_g1\_i10.p1 |  | Image |  | Peak List |  |
|  | | | | | | | | | | | | | | | | | | | | | | | | | | | | | | | | | | | | | | | | | | | | | | | | | | | | | |  |
|  | High |  | [R].GVLGTVKNLLIGAGKSAAQSVLKTLScKLSNDc.[-] |  | HCD |  | C27(Dehydro); C33(Dehydro) |  | 5.32 |  | 1.3e-3 |  | 1.4e-3 |  | 1.13 |  | 263.80 |  | 1.000 |  |  |  | C27(Dehydro): 100; C33(Dehydro): 100 |  |  |  |  |  |  |  | 0.61 |  | 1 |  | Sequest HT (3) |  | 4 |  | 822.45160 |  | -0.48 |  | 44.2325 |  | 12910 |  | 12910 |  | TRINITY\_DN0\_c1\_g1\_i15.p1; TRINITY\_DN0\_c1\_g1\_i23.p1; TRINITY\_DN0\_c1\_g1\_i11.p1; TRINITY\_DN0\_c1\_g1\_i10.p1 |  | Image |  | Peak List |  |
|  | | | | | | | | | | | | | | | | | | | | | | | | | | | | | | | | | | | | | | | | | | | | | | | | | | | | | |  |
|  | High |  | [R].GVLGTVKNLLIGAGKSAAQSVLKTLScKLSNDc.[-] |  | HCD |  | C27(Dehydro); C33(Dehydro) |  | 4.74 |  | 1.3e-3 |  | 4.6e-4 |  | 1.36 |  | 347.56 |  | 1.000 |  |  |  | C27(Dehydro): 100; C33(Dehydro): 100 |  |  |  |  |  |  |  | 0.70 |  | 1 |  | Sequest HT (3) |  | 3 |  | 1096.26636 |  | -0.49 |  | 44.2782 |  | 12932 |  | 12932 |  | TRINITY\_DN0\_c1\_g1\_i15.p1; TRINITY\_DN0\_c1\_g1\_i23.p1; TRINITY\_DN0\_c1\_g1\_i11.p1; TRINITY\_DN0\_c1\_g1\_i10.p1 |  | Image |  | Peak List |  |
|  | | | | | | | | | | | | | | | | | | | | | | | | | | | | | | | | | | | | | | | | | | | | | | | | | | | | | |  |
|  | High |  | [R].GVLGTVKNLLIGAGKSAAQSVLKTLScKLSNDc.[-] |  | HCD |  | C27(Dehydro); C33(Dehydro) |  | 4.45 |  | 1.3e-3 |  | 5.8e-3 |  | 0.81 |  | 166.47 |  | 1.000 |  |  |  | C27(Dehydro): 100; C33(Dehydro): 100 |  |  |  |  |  |  |  | 0.60 |  | 1 |  | Sequest HT (3) |  | 5 |  | 658.16150 |  | -2.36 |  | 44.3599 |  | 12969 |  | 12969 |  | TRINITY\_DN0\_c1\_g1\_i15.p1; TRINITY\_DN0\_c1\_g1\_i23.p1; TRINITY\_DN0\_c1\_g1\_i11.p1; TRINITY\_DN0\_c1\_g1\_i10.p1 |  | Image |  | Peak List |  |
|  | | | | | | | | | | | | | | | | | | | | | | | | | | | | | | | | | | | | | | | | | | | | | | | | | | | | | |  |
|  | High |  | [R].GVLGTVKNLLIGAGKSAAQSVLKTLScKLSNDc.[-] |  | HCD |  | C27(Dehydro); C33(Dehydro) |  | 4.71 |  | 1.3e-3 |  | 4.1e-3 |  | 0.886 |  | 283.58 |  | 1.000 |  |  |  | C27(Dehydro): 100; C33(Dehydro): 100 |  |  |  |  |  |  |  | 0.68 |  | 1 |  | Sequest HT (3) |  | 4 |  | 822.45221 |  | 0.26 |  | 44.3785 |  | 12977 |  | 12977 |  | TRINITY\_DN0\_c1\_g1\_i15.p1; TRINITY\_DN0\_c1\_g1\_i23.p1; TRINITY\_DN0\_c1\_g1\_i11.p1; TRINITY\_DN0\_c1\_g1\_i10.p1 |  | Image |  | Peak List |  |
|  | | | | | | | | | | | | | | | | | | | | | | | | | | | | | | | | | | | | | | | | | | | | | | | | | | | | | |  |
|  | High |  | [R].GVLGTVKNLLIGAGKSAAQSVLKTLScKLSNDc.[-] |  | HCD |  | C27(Dehydro); C33(Dehydro) |  | 5.30 |  | 1.3e-3 |  | 1.6e-4 |  | 1.59 |  | 346.00 |  | 1.000 |  |  |  | C27(Dehydro): 100; C33(Dehydro): 100 |  |  |  |  |  |  |  | 0.77 |  | 1 |  | Sequest HT (3) |  | 3 |  | 1096.26636 |  | -0.49 |  | 44.4173 |  | 12994 |  | 12994 |  | TRINITY\_DN0\_c1\_g1\_i15.p1; TRINITY\_DN0\_c1\_g1\_i23.p1; TRINITY\_DN0\_c1\_g1\_i11.p1; TRINITY\_DN0\_c1\_g1\_i10.p1 |  | Image |  | Peak List |  |
|  | | | | | | | | | | | | | | | | | | | | | | | | | | | | | | | | | | | | | | | | | | | | | | | | | | | | | |  |
|  | High |  | [R].GVLGTVKNLLIGAGKSAAQSVLKTLScKLSNDc.[-] |  | HCD |  | C27(Dehydro); C33(Dehydro) |  | 4.46 |  | 2.4e-3 |  | 7.1e-2 |  | 0.258 |  | 169.34 |  | 1.000 |  |  |  | C27(Dehydro): 100; C33(Dehydro): 100 |  |  |  |  |  |  |  | 0.64 |  | 1 |  | Sequest HT (3) |  | 5 |  | 658.16211 |  | -1.43 |  | 44.5099 |  | 13034 |  | 13034 |  | TRINITY\_DN0\_c1\_g1\_i15.p1; TRINITY\_DN0\_c1\_g1\_i23.p1; TRINITY\_DN0\_c1\_g1\_i11.p1; TRINITY\_DN0\_c1\_g1\_i10.p1 |  | Image |  | Peak List |  |
|  | | | | | | | | | | | | | | | | | | | | | | | | | | | | | | | | | | | | | | | | | | | | | | | | | | | | | |  |
|  | High |  | [R].GVLGTVKNLLIGAGKSAAQSVLKTLScKLSNDc.[-] |  | HCD |  | C27(Dehydro); C33(Dehydro) |  | 5.46 |  | 1.3e-3 |  | 1.8e-3 |  | 1.07 |  | 323.38 |  | 1.000 |  |  |  | C27(Dehydro): 100; C33(Dehydro): 100 |  |  |  |  |  |  |  | 0.75 |  | 1 |  | Sequest HT (3) |  | 3 |  | 1096.26648 |  | -0.38 |  | 44.5547 |  | 13052 |  | 13052 |  | TRINITY\_DN0\_c1\_g1\_i15.p1; TRINITY\_DN0\_c1\_g1\_i23.p1; TRINITY\_DN0\_c1\_g1\_i11.p1; TRINITY\_DN0\_c1\_g1\_i10.p1 |  | Image |  | Peak List |  |
|  | | | | | | | | | | | | | | | | | | | | | | | | | | | | | | | | | | | | | | | | | | | | | | | | | | | | | |  |
|  | High |  | [R].GVLGTVKNLLIGAGKSAAQSVLKTLScKLSNDc.[-] |  | HCD |  | C27(Dehydro); C33(Dehydro) |  | 3.94 |  | 1.3e-3 |  | 3.3e-2 |  | 0.432 |  | 178.16 |  | 1.000 |  |  |  | C27(Dehydro): 100; C33(Dehydro): 100 |  |  |  |  |  |  |  | 0.58 |  | 1 |  | Sequest HT (3) |  | 4 |  | 822.45245 |  | 0.56 |  | 44.6537 |  | 13092 |  | 13092 |  | TRINITY\_DN0\_c1\_g1\_i15.p1; TRINITY\_DN0\_c1\_g1\_i23.p1; TRINITY\_DN0\_c1\_g1\_i11.p1; TRINITY\_DN0\_c1\_g1\_i10.p1 |  | Image |  | Peak List |  |
|  | | | | | | | | | | | | | | | | | | | | | | | | | | | | | | | | | | | | | | | | | | | | | | | | | | | | | |  |
|  | High |  | [R].GVLGTVKNLLIGAGKSAAQSVLKTLScKLSnDc.[-] |  | HCD |  | C27(Dehydro); N31(Deamidated); C33(Dehydro) |  | 5.61 |  | 1.3e-3 |  | 8.9e-4 |  | 1.22 |  | 243.83 |  | 1.000 |  | b1+(8): 769.46; b1+(9): 882.54; b1+(10): 995.62; b1+(12): 1165.73; b1+(13): 1236.77; y1+(14): 1509.73; y1+(12): 1323.63; y1+(11): 1210.54; y1+(10): 1082.45; y1+(8): 868.32; y1+(7): 781.29; b2+(9): 441.77; y2+(23): 1147.08; y2+(22): 1090.54; y2+(20): 1026.51; y2+(11): 605.78 |  | C27(Dehydro): 100; N31(Deamidated): 100; C33(Dehydro): 100 |  |  |  |  |  |  |  | 0.63 |  | 1 |  | Sequest HT (3) |  | 4 |  | 822.69928 |  | 1.56 |  | 44.7960 |  | 13152 |  | 13152 |  | TRINITY\_DN0\_c1\_g1\_i15.p1; TRINITY\_DN0\_c1\_g1\_i23.p1; TRINITY\_DN0\_c1\_g1\_i11.p1; TRINITY\_DN0\_c1\_g1\_i10.p1 |  | Image |  | Peak List |  |
|  | | | | | | | | | | | | | | | | | | | | | | | | | | | | | | | | | | | | | | | | | | | | | | | | | | | | | |  |
|  | High |  | [R].GVLGTVKNLLIGAGKSAAQSVLKTLScKLSnDc.[-] |  | HCD |  | C27(Dehydro); N31(Deamidated); C33(Dehydro) |  | 4.48 |  | 1.3e-3 |  | 1.0e-4 |  | 1.69 |  | 290.61 |  | 1.000 |  | b1+(8): 769.46; b1+(9): 882.54; b1+(10): 995.62; b1+(13): 1236.77; y1+(22): 2180.07; y1+(17): 1779.86; y1+(16): 1708.82; y1+(15): 1637.79; y1+(14): 1509.73; y1+(12): 1323.63; y1+(11): 1210.54; y1+(8): 868.32; y1+(7): 781.29; b2+(16): 754.96; b2+(17): 790.48; y2+(24): 1203.62; y2+(22): 1090.54 |  | C27(Dehydro): 100; N31(Deamidated): 100; C33(Dehydro): 100 |  |  |  |  |  |  |  | 0.66 |  | 1 |  | Sequest HT (3) |  | 3 |  | 1096.59509 |  | 0.17 |  | 44.8561 |  | 13179 |  | 13179 |  | TRINITY\_DN0\_c1\_g1\_i15.p1; TRINITY\_DN0\_c1\_g1\_i23.p1; TRINITY\_DN0\_c1\_g1\_i11.p1; TRINITY\_DN0\_c1\_g1\_i10.p1 |  | Image |  | Peak List |  |
|  | | | | | | | | | | | | | | | | | | | | | | | | | | | | | | | | | | | | | | | | | | | | | | | | | | | | | |  |
|  | High |  | [R].GVLGTVKNLLIGAGKSAAQSVLKTLScKLSnDc.[-] |  | HCD |  | C27(Dehydro); N31(Deamidated); C33(Dehydro) |  | 3.87 |  | 1.3e-3 |  | 1.0e-2 |  | 0.69 |  | 154.24 |  | 1.000 |  | b1+(8): 769.46; b1+(9): 882.54; b1+(10): 995.62; y1+(12): 1323.63; y1+(11): 1210.54; y1+(10): 1082.45; y1+(8): 868.32; b2+(9): 441.77; y2+(22): 1090.54; y2+(20): 1026.51; y2+(18): 933.95; y2+(11): 605.78 |  | C27(Dehydro): 100; N31(Deamidated): 100; C33(Dehydro): 100 |  |  |  |  |  |  |  | 0.57 |  | 1 |  | Sequest HT (3) |  | 5 |  | 658.36011 |  | 0.39 |  | 44.9150 |  | 13203 |  | 13203 |  | TRINITY\_DN0\_c1\_g1\_i15.p1; TRINITY\_DN0\_c1\_g1\_i23.p1; TRINITY\_DN0\_c1\_g1\_i11.p1; TRINITY\_DN0\_c1\_g1\_i10.p1 |  | Image |  | Peak List |  |
|  | | | | | | | | | | | | | | | | | | | | | | | | | | | | | | | | | | | | | | | | | | | | | | | | | | | | | |  |
|  | High |  | [R].GVLGTVKNLLIGAGKSAAQSVLKTLScKLSnDc.[-] |  | HCD |  | C27(Dehydro); N31(Deamidated); C33(Dehydro) |  | 4.62 |  | 1.3e-3 |  | 5.0e-3 |  | 0.842 |  | 220.46 |  | 1.000 |  | b1+(8): 769.46; b1+(9): 882.54; b1+(10): 995.62; b1+(11): 1108.71; b1+(13): 1236.77; y1+(14): 1509.73; y1+(12): 1323.63; y1+(11): 1210.54; y1+(10): 1082.45; y1+(9): 981.40; y1+(8): 868.32; b2+(9): 441.77; y2+(22): 1090.54; y2+(11): 605.78 |  | C27(Dehydro): 100; N31(Deamidated): 100; C33(Dehydro): 100 |  |  |  |  |  |  |  | 0.61 |  | 1 |  | Sequest HT (3) |  | 4 |  | 822.69794 |  | -0.07 |  | 44.9432 |  | 13215 |  | 13215 |  | TRINITY\_DN0\_c1\_g1\_i15.p1; TRINITY\_DN0\_c1\_g1\_i23.p1; TRINITY\_DN0\_c1\_g1\_i11.p1; TRINITY\_DN0\_c1\_g1\_i10.p1 |  | Image |  | Peak List |  |
|  | | | | | | | | | | | | | | | | | | | | | | | | | | | | | | | | | | | | | | | | | | | | | | | | | | | | | |  |
|  | High |  | [R].GVLGTVKNLLIGAGKSAAQSVLKTLScKLSnDc.[-] |  | HCD |  | C27(Dehydro); N31(Deamidated); C33(Dehydro) |  | 4.19 |  | 1.3e-3 |  | 1.6e-2 |  | 0.585 |  | 261.68 |  | 1.000 |  | b1+(8): 769.46; b1+(9): 882.54; b1+(10): 995.62; b1+(13): 1236.77; b1+(14): 1293.79; y1+(18): 1866.89; y1+(17): 1779.86; y1+(15): 1637.79; y1+(14): 1509.73; y1+(12): 1323.63; y1+(11): 1210.54; y1+(9): 981.40; y1+(8): 868.32; y1+(7): 781.29; y2+(24): 1203.62 |  | C27(Dehydro): 100; N31(Deamidated): 100; C33(Dehydro): 100 |  |  |  |  |  |  |  | 0.58 |  | 1 |  | Sequest HT (3) |  | 3 |  | 1096.59631 |  | 1.29 |  | 45.0037 |  | 13241 |  | 13241 |  | TRINITY\_DN0\_c1\_g1\_i15.p1; TRINITY\_DN0\_c1\_g1\_i23.p1; TRINITY\_DN0\_c1\_g1\_i11.p1; TRINITY\_DN0\_c1\_g1\_i10.p1 |  | Image |  | Peak List |  |
|  | | | | | | | | | | | | | | | | | | | | | | | | | | | | | | | | | | | | | | | | | | | | | | | | | | | | | |  |
|  | High |  | [R].GVLGTVKNLLIGAGKSAAQSVLKTLScKLSnDc.[-] |  | HCD |  | C27(Dehydro); N31(Deamidated); C33(Dehydro) |  | 3.55 |  | 1.3e-3 |  | 2.1e-2 |  | 0.527 |  | 142.72 |  | 0.000 |  | b1+(9): 883.52; b1+(10): 996.61; y1+(14): 1508.74; y1+(12): 1322.64; y1+(11): 1209.56; y1+(10): 1081.47; y1+(9): 980.42; y1+(8): 867.33; b2+(21): 983.57; y2+(12): 661.83; y2+(11): 605.28 |  | N8(Deamidated): 100; C27(Dehydro): 100; C33(Dehydro): 100 |  |  |  |  |  |  |  | 0.07 |  | 1 |  | Sequest HT (3) |  | 4 |  | 822.69916 |  | 1.41 |  | 46.1304 |  | 13728 |  | 13728 |  | TRINITY\_DN0\_c1\_g1\_i15.p1; TRINITY\_DN0\_c1\_g1\_i23.p1; TRINITY\_DN0\_c1\_g1\_i11.p1; TRINITY\_DN0\_c1\_g1\_i10.p1 |  | Image |  | Peak List |  |
|  | | | | | | | | | | | | | | | | | | | | | | | | | | | | | | | | | | | | | | | | | | | | | | | | | | | | | |  |
|  | High |  | [R].GVLGTVKNLLIGAGKSAAQSVLKTLScKLSnDc.[-] |  | HCD |  | C27(Dehydro); N31(Deamidated); C33(Dehydro) |  | 3.43 |  | 1.3e-3 |  | 1.5e-3 |  | 1.1 |  | 245.90 |  | 1.000 |  | b1+(8): 769.46; b1+(9): 882.54; b1+(10): 995.62; b1+(13): 1236.77; y1+(22): 2180.07; y1+(18): 1866.89; y1+(17): 1779.86; y1+(16): 1708.82; y1+(15): 1637.79; y1+(11): 1210.54; y1+(8): 868.32; b2+(16): 754.96 |  | C27(Dehydro): 100; N31(Deamidated): 100; C33(Dehydro): 100 |  |  |  |  |  |  |  | 0.14 |  | 1 |  | Sequest HT (3) |  | 3 |  | 1096.59326 |  | -1.50 |  | 46.1345 |  | 13730 |  | 13730 |  | TRINITY\_DN0\_c1\_g1\_i15.p1; TRINITY\_DN0\_c1\_g1\_i23.p1; TRINITY\_DN0\_c1\_g1\_i11.p1; TRINITY\_DN0\_c1\_g1\_i10.p1 |  | Image |  | Peak List |  |
|  | | | | | | | | | | | | | | | | | | | | | | | | | | | | | | | | | | | | | | | | | | | | | | | | | | | | | |  |
|  | High |  | [R].GVLGTVKNLLIGAGKSAAQSVLKTLScKLSnDc.[-] |  | HCD |  | C27(Dehydro); N31(Deamidated); C33(Dehydro) |  | 3.41 |  | 1.3e-3 |  | 2.6e-2 |  | 0.478 |  | 109.48 |  | 0.000 |  | b1+(10): 996.61; y1+(12): 1322.64; y1+(11): 1209.56; y1+(10): 1081.47; y1+(9): 980.42; y1+(8): 867.33; b2+(10): 498.81; y2+(11): 605.28 |  | N8(Deamidated): 100; C27(Dehydro): 100; C33(Dehydro): 100 |  |  |  |  |  |  |  | 0.33 |  | 1 |  | Sequest HT (3) |  | 4 |  | 822.69843 |  | 0.52 |  | 46.2751 |  | 13798 |  | 13798 |  | TRINITY\_DN0\_c1\_g1\_i15.p1; TRINITY\_DN0\_c1\_g1\_i23.p1; TRINITY\_DN0\_c1\_g1\_i11.p1; TRINITY\_DN0\_c1\_g1\_i10.p1 |  | Image |  | Peak List |  |
|  | | | | | | | | | | | | | | | | | | | | | | | | | | | | | | | | | | | | | | | | | | | | | | | | | | | | | |  |
|  | High |  | [R].GVLGTVKnLLIGAGKSAAQSVLKTLScKLSNDc.[-] |  | HCD |  | N8(Deamidated); C27(Dehydro); C33(Dehydro) |  | 2.72 |  | 1.3e-3 |  | 1.7e-2 |  | 0.577 |  | 125.27 |  | 0.000 |  | b1+(8): 769.46; b1+(9): 882.54; b1+(10): 995.62; b1+(11): 1108.71; b1+(13): 1236.77; y1+(18): 1866.89; y1+(16): 1708.82; y1+(15): 1637.79; y1+(11): 1210.54; y1+(8): 868.32; y1+(7): 781.29; b2+(9): 441.77; b2+(12): 583.37; b2+(16): 754.96; b2+(17): 790.48; y2+(24): 1203.62 |  | C27(Dehydro): 100; N31(Deamidated): 100; C33(Dehydro): 100 |  |  |  |  |  |  |  |  |  | 2 |  | Sequest HT (3) |  | 3 |  | 1096.59497 |  | 0.06 |  | 46.2764 |  | 13799 |  | 13799 |  | TRINITY\_DN0\_c1\_g1\_i15.p1; TRINITY\_DN0\_c1\_g1\_i23.p1; TRINITY\_DN0\_c1\_g1\_i11.p1; TRINITY\_DN0\_c1\_g1\_i10.p1 |  | Image |  | Peak List |  |
|  | | | | | | | | | | | | | | | | | | | | | | | | | | | | | | | | | | | | | | | | | | | | | | | | | | | | | |  |
|  | High |  | [R].GVLGTVKNLLIGAGKSAAQSVLKTLScKLSnDc.[-] |  | HCD |  | C27(Dehydro); N31(Deamidated); C33(Dehydro) |  | 2.71 |  | 1.3e-3 |  | 4.1e-3 |  | 0.883 |  | 283.44 |  | 1.000 |  | b1+(8): 769.46; b1+(9): 882.54; b1+(10): 995.62; b1+(11): 1108.71; b1+(13): 1236.77; y1+(18): 1866.89; y1+(16): 1708.82; y1+(15): 1637.79; y1+(11): 1210.54; y1+(8): 868.32; y1+(7): 781.29; b2+(9): 441.77; b2+(12): 583.37; b2+(16): 754.96; b2+(17): 790.48; y2+(24): 1203.62 |  | C27(Dehydro): 100; N31(Deamidated): 100; C33(Dehydro): 100 |  |  |  |  |  |  |  |  |  | 3 |  | Sequest HT (3) |  | 3 |  | 1096.59497 |  | 0.06 |  | 46.2764 |  | 13799 |  | 13799 |  | TRINITY\_DN0\_c1\_g1\_i15.p1; TRINITY\_DN0\_c1\_g1\_i23.p1; TRINITY\_DN0\_c1\_g1\_i11.p1; TRINITY\_DN0\_c1\_g1\_i10.p1 |  | Image |  | Peak List |  |
|  | | | | | | | | | | | | | | | | | | | | | | | | | | | | | | | | | | | | | | | | | | | | | | | | | | | | | |  |
|  | High |  | [R].GVLGTVKNLLIGAGKSAAQSVLKTLScKLSnDc.[-] |  | HCD |  | C27(Dehydro); N31(Deamidated); C33(Dehydro) |  | 3.44 |  | 2.4e-3 |  | 7.7e-2 |  | 0.24 |  | 154.95 |  | 1.000 |  | b1+(8): 769.46; b1+(9): 882.54; b1+(10): 995.62; b1+(11): 1108.71; b1+(13): 1236.77; y1+(11): 1210.54; y1+(10): 1082.45; b2+(8): 385.23; b2+(9): 441.77; b2+(10): 498.32; y2+(23): 1147.08; y2+(22): 1090.54 |  | C27(Dehydro): 100; N31(Deamidated): 100; C33(Dehydro): 100 |  |  |  |  |  |  |  | 0.17 |  | 1 |  | Sequest HT (3) |  | 4 |  | 822.69775 |  | -0.30 |  | 46.4149 |  | 13862 |  | 13862 |  | TRINITY\_DN0\_c1\_g1\_i15.p1; TRINITY\_DN0\_c1\_g1\_i23.p1; TRINITY\_DN0\_c1\_g1\_i11.p1; TRINITY\_DN0\_c1\_g1\_i10.p1 |  | Image |  | Peak List |  |
|  | | | | | | | | | | | | | | | | | | | | | | | | | | | | | | | | | | | | | | | | | | | | | | | | | | | | | |  |
|  | High |  | [R].GVLGTVKnLLIGAGKSAAQSVLKTLScKLSNDc.[-] |  | HCD |  | N8(Deamidated); C27(Dehydro); C33(Dehydro) |  | 4.50 |  | 1.3e-3 |  | 1.8e-2 |  | 0.559 |  | 211.58 |  | 1.000 |  | b1+(8): 770.44; b1+(9): 883.52; b1+(10): 996.61; b1+(11): 1109.69; b1+(13): 1237.75; b1+(14): 1294.77; y1+(14): 1508.74; y1+(12): 1322.64; y1+(11): 1209.56; y1+(10): 1081.47; y1+(9): 980.42; y1+(8): 867.33; y2+(22): 1090.05 |  | N8(Deamidated): 100; C27(Dehydro): 100; C33(Dehydro): 100 |  |  |  |  |  |  |  |  |  | 2 |  | Sequest HT (3) |  | 4 |  | 822.69897 |  | 1.19 |  | 46.5894 |  | 13932 |  | 13932 |  | TRINITY\_DN0\_c1\_g1\_i15.p1; TRINITY\_DN0\_c1\_g1\_i23.p1; TRINITY\_DN0\_c1\_g1\_i11.p1; TRINITY\_DN0\_c1\_g1\_i10.p1 |  | Image |  | Peak List |  |
|  | | | | | | | | | | | | | | | | | | | | | | | | | | | | | | | | | | | | | | | | | | | | | | | | | | | | | |  |
|  | High |  | [R].GVLGTVKnLLIGAGKSAAQSVLKTLScKLSNDc.[-] |  | HCD |  | N8(Deamidated); C27(Dehydro); C33(Dehydro) |  | 5.71 |  | 1.3e-3 |  | 5.0e-3 |  | 0.843 |  | 204.54 |  | 1.000 |  | b1+(8): 770.44; b1+(9): 883.52; b1+(10): 996.61; b1+(13): 1237.75; y1+(14): 1508.74; y1+(12): 1322.64; y1+(11): 1209.56; y1+(10): 1081.47; y1+(9): 980.42; y1+(8): 867.33; y1+(7): 780.30; y2+(22): 1090.05; y2+(11): 605.28 |  | N8(Deamidated): 100; C27(Dehydro): 100; C33(Dehydro): 100 |  |  |  |  |  |  |  |  |  | 2 |  | Sequest HT (3) |  | 4 |  | 822.69885 |  | 1.04 |  | 46.7300 |  | 13991 |  | 13991 |  | TRINITY\_DN0\_c1\_g1\_i15.p1; TRINITY\_DN0\_c1\_g1\_i23.p1; TRINITY\_DN0\_c1\_g1\_i11.p1; TRINITY\_DN0\_c1\_g1\_i10.p1 |  | Image |  | Peak List |  |
|  | | | | | | | | | | | | | | | | | | | | | | | | | | | | | | | | | | | | | | | | | | | | | | | | | | | | | |  |
|  | High |  | [R].GVLGTVKnLLIGAGKSAAQSVLKTLScKLSNDc.[-] |  | HCD |  | N8(Deamidated); C27(Dehydro); C33(Dehydro) |  | 5.57 |  | 1.3e-3 |  | 5.7e-5 |  | 1.82 |  | 305.15 |  | 1.000 |  | b1+(8): 770.44; b1+(9): 883.52; b1+(10): 996.61; b1+(13): 1237.75; b1+(14): 1294.77; y1+(18): 1865.91; y1+(16): 1707.84; y1+(15): 1636.80; y1+(14): 1508.74; y1+(13): 1421.71; y1+(12): 1322.64; y1+(11): 1209.56; y1+(9): 980.42; y1+(8): 867.33; y1+(7): 780.30; b2+(16): 755.45; b2+(18): 826.49 |  | N8(Deamidated): 100; C27(Dehydro): 100; C33(Dehydro): 100 |  |  |  |  |  |  |  |  |  | 2 |  | Sequest HT (3) |  | 3 |  | 1096.59412 |  | -0.72 |  | 46.7432 |  | 13997 |  | 13997 |  | TRINITY\_DN0\_c1\_g1\_i15.p1; TRINITY\_DN0\_c1\_g1\_i23.p1; TRINITY\_DN0\_c1\_g1\_i11.p1; TRINITY\_DN0\_c1\_g1\_i10.p1 |  | Image |  | Peak List |  |
|  | | | | | | | | | | | | | | | | | | | | | | | | | | | | | | | | | | | | | | | | | | | | | | | | | | | | | |  |
|  | High |  | [R].GVLGTVKnLLIGAGKSAAQSVLKTLScKLSNDc.[-] |  | HCD |  | N8(Deamidated); C27(Dehydro); C33(Dehydro) |  | 3.38 |  | 1.3e-3 |  | 1.4e-3 |  | 1.13 |  | 376.51 |  | 1.000 |  | b1+(8): 770.44; b1+(9): 883.52; b1+(10): 996.61; b1+(11): 1109.69; b1+(13): 1237.75; b1+(14): 1294.77; b1+(15): 1422.87; b1+(21): 1966.13; b1+(22): 2079.22; b1+(23): 2207.31; y1+(25): 2518.34; y1+(24): 2405.25; y1+(22): 2179.08; y1+(20): 2051.03; y1+(18): 1865.91; y1+(17): 1778.88; y1+(14): 1508.74; y1+(12): 1322.64; y1+(11): 1209.56; y1+(10): 1081.47; y1+(9): 980.42; y1+(8): 867.33; y1+(7): 780.30 |  | N8(Deamidated): 100; C27(Dehydro): 100; C33(Dehydro): 100 |  |  |  |  |  |  |  |  |  | 2 |  | Sequest HT (3) |  | 2 |  | 1644.38684 |  | -1.14 |  | 46.8636 |  | 14049 |  | 14049 |  | TRINITY\_DN0\_c1\_g1\_i15.p1; TRINITY\_DN0\_c1\_g1\_i23.p1; TRINITY\_DN0\_c1\_g1\_i11.p1; TRINITY\_DN0\_c1\_g1\_i10.p1 |  | Image |  | Peak List |  |
|  | | | | | | | | | | | | | | | | | | | | | | | | | | | | | | | | | | | | | | | | | | | | | | | | | | | | | |  |
|  | High |  | [R].GVLGTVKnLLIGAGKSAAQSVLKTLScKLSNDc.[-] |  | HCD |  | N8(Deamidated); C27(Dehydro); C33(Dehydro) |  | 4.56 |  | 1.3e-3 |  | 1.5e-3 |  | 1.11 |  | 256.09 |  | 1.000 |  | b1+(8): 770.44; b1+(9): 883.52; b1+(10): 996.61; b1+(12): 1166.71; b1+(13): 1237.75; b1+(14): 1294.77; y1+(14): 1508.74; y1+(12): 1322.64; y1+(11): 1209.56; y1+(10): 1081.47; y1+(9): 980.42; y1+(8): 867.33; b2+(10): 498.81; y2+(22): 1090.05; y2+(12): 661.83; y2+(11): 605.28 |  | N8(Deamidated): 100; C27(Dehydro): 100; C33(Dehydro): 100 |  |  |  |  |  |  |  |  |  | 2 |  | Sequest HT (3) |  | 4 |  | 822.69775 |  | -0.30 |  | 46.8718 |  | 14052 |  | 14052 |  | TRINITY\_DN0\_c1\_g1\_i15.p1; TRINITY\_DN0\_c1\_g1\_i23.p1; TRINITY\_DN0\_c1\_g1\_i11.p1; TRINITY\_DN0\_c1\_g1\_i10.p1 |  | Image |  | Peak List |  |
|  | | | | | | | | | | | | | | | | | | | | | | | | | | | | | | | | | | | | | | | | | | | | | | | | | | | | | |  |
|  | High |  | [R].GVLGTVKnLLIGAGKSAAQSVLKTLScKLSNDc.[-] |  | HCD |  | N8(Deamidated); C27(Dehydro); C33(Dehydro) |  | 4.85 |  | 1.3e-3 |  | 2.0e-5 |  | 2.05 |  | 336.33 |  | 1.000 |  | b1+(8): 770.44; b1+(9): 883.52; b1+(10): 996.61; b1+(13): 1237.75; y1+(20): 2051.03; y1+(18): 1865.91; y1+(17): 1778.88; y1+(16): 1707.84; y1+(15): 1636.80; y1+(14): 1508.74; y1+(12): 1322.64; y1+(11): 1209.56; y1+(9): 980.42; y1+(8): 867.33; y1+(7): 780.30; b2+(16): 755.45; b2+(17): 790.97 |  | N8(Deamidated): 100; C27(Dehydro): 100; C33(Dehydro): 100 |  |  |  |  |  |  |  |  |  | 2 |  | Sequest HT (3) |  | 3 |  | 1096.59375 |  | -1.05 |  | 46.8865 |  | 14056 |  | 14056 |  | TRINITY\_DN0\_c1\_g1\_i15.p1; TRINITY\_DN0\_c1\_g1\_i23.p1; TRINITY\_DN0\_c1\_g1\_i11.p1; TRINITY\_DN0\_c1\_g1\_i10.p1 |  | Image |  | Peak List |  |
|  | | | | | | | | | | | | | | | | | | | | | | | | | | | | | | | | | | | | | | | | | | | | | | | | | | | | | |  |
|  | High |  | [R].GVLGTVKnLLIGAGKSAAQSVLKTLScKLSNDc.[-] |  | HCD |  | N8(Deamidated); C27(Dehydro); C33(Dehydro) |  | 4.18 |  | 1.3e-3 |  | 4.0e-3 |  | 0.893 |  | 188.58 |  | 1.000 |  | b1+(8): 770.44; b1+(9): 883.52; b1+(10): 996.61; b1+(12): 1166.71; b1+(13): 1237.75; b1+(14): 1294.77; y1+(14): 1508.74; y1+(12): 1322.64; y1+(11): 1209.56; y1+(10): 1081.47; y1+(8): 867.33; y2+(22): 1090.05 |  | N8(Deamidated): 100; C27(Dehydro): 100; C33(Dehydro): 100 |  |  |  |  |  |  |  |  |  | 2 |  | Sequest HT (3) |  | 4 |  | 822.69781 |  | -0.22 |  | 47.0154 |  | 14113 |  | 14113 |  | TRINITY\_DN0\_c1\_g1\_i15.p1; TRINITY\_DN0\_c1\_g1\_i23.p1; TRINITY\_DN0\_c1\_g1\_i11.p1; TRINITY\_DN0\_c1\_g1\_i10.p1 |  | Image |  | Peak List |  |
|  | | | | | | | | | | | | | | | | | | | | | | | | | | | | | | | | | | | | | | | | | | | | | | | | | | | | | |  |
|  | High |  | [R].GVLGTVKnLLIGAGKSAAQSVLKTLScKLSNDc.[-] |  | HCD |  | N8(Deamidated); C27(Dehydro); C33(Dehydro) |  | 3.36 |  | 1.3e-3 |  | 4.3e-4 |  | 1.38 |  | 342.63 |  | 1.000 |  | b1+(8): 770.44; b1+(9): 883.52; b1+(10): 996.61; b1+(11): 1109.69; b1+(13): 1237.75; b1+(15): 1422.87; b1+(23): 2207.31; y1+(23): 2292.17; y1+(22): 2179.08; y1+(20): 2051.03; y1+(18): 1865.91; y1+(16): 1707.84; y1+(14): 1508.74; y1+(13): 1421.71; y1+(12): 1322.64; y1+(11): 1209.56; y1+(10): 1081.47; y1+(9): 980.42; y1+(8): 867.33; y1+(7): 780.30 |  | N8(Deamidated): 100; C27(Dehydro): 100; C33(Dehydro): 100 |  |  |  |  |  |  |  |  |  | 2 |  | Sequest HT (3) |  | 2 |  | 1644.39148 |  | 1.68 |  | 47.0207 |  | 14117 |  | 14117 |  | TRINITY\_DN0\_c1\_g1\_i15.p1; TRINITY\_DN0\_c1\_g1\_i23.p1; TRINITY\_DN0\_c1\_g1\_i11.p1; TRINITY\_DN0\_c1\_g1\_i10.p1 |  | Image |  | Peak List |  |
|  | | | | | | | | | | | | | | | | | | | | | | | | | | | | | | | | | | | | | | | | | | | | | | | | | | | | | |  |
|  | High |  | [R].GVLGTVKnLLIGAGKSAAQSVLKTLScKLSNDc.[-] |  | HCD |  | N8(Deamidated); C27(Dehydro); C33(Dehydro) |  | 5.09 |  | 1.3e-3 |  | 4.0e-4 |  | 1.4 |  | 280.64 |  | 1.000 |  | b1+(8): 770.44; b1+(9): 883.52; b1+(10): 996.61; b1+(13): 1237.75; y1+(22): 2179.08; y1+(18): 1865.91; y1+(15): 1636.80; y1+(13): 1421.71; y1+(12): 1322.64; y1+(11): 1209.56; y1+(9): 980.42; y1+(8): 867.33; y1+(7): 780.30; b2+(16): 755.45 |  | N8(Deamidated): 100; C27(Dehydro): 100; C33(Dehydro): 100 |  |  |  |  |  |  |  |  |  | 2 |  | Sequest HT (3) |  | 3 |  | 1096.59314 |  | -1.61 |  | 47.0310 |  | 14122 |  | 14122 |  | TRINITY\_DN0\_c1\_g1\_i15.p1; TRINITY\_DN0\_c1\_g1\_i23.p1; TRINITY\_DN0\_c1\_g1\_i11.p1; TRINITY\_DN0\_c1\_g1\_i10.p1 |  | Image |  | Peak List |  |
|  | | | | | | | | | | | | | | | | | | | | | | | | | | | | | | | | | | | | | | | | | | | | | | | | | | | | | |  |
|  | High |  | [R].GVLGTVKnLLIGAGKSAAQSVLKTLScKLSNDc.[-] |  | HCD |  | N8(Deamidated); C27(Dehydro); C33(Dehydro) |  | 4.28 |  | 1.3e-3 |  | 4.1e-3 |  | 0.886 |  | 243.83 |  | 1.000 |  | b1+(8): 770.44; b1+(9): 883.52; b1+(10): 996.61; b1+(12): 1166.71; b1+(13): 1237.75; y1+(14): 1508.74; y1+(12): 1322.64; y1+(11): 1209.56; y1+(10): 1081.47; y1+(9): 980.42; y1+(8): 867.33; y2+(22): 1090.05; y2+(20): 1026.02; y2+(18): 933.46; y2+(12): 661.83 |  | N8(Deamidated): 100; C27(Dehydro): 100; C33(Dehydro): 100 |  |  |  |  |  |  |  |  |  | 2 |  | Sequest HT (3) |  | 4 |  | 822.69745 |  | -0.67 |  | 47.1586 |  | 14183 |  | 14183 |  | TRINITY\_DN0\_c1\_g1\_i15.p1; TRINITY\_DN0\_c1\_g1\_i23.p1; TRINITY\_DN0\_c1\_g1\_i11.p1; TRINITY\_DN0\_c1\_g1\_i10.p1 |  | Image |  | Peak List |  |
|  | | | | | | | | | | | | | | | | | | | | | | | | | | | | | | | | | | | | | | | | | | | | | | | | | | | | | |  |
|  | High |  | [R].GVLGTVKnLLIGAGKSAAQSVLKTLScKLSNDc.[-] |  | HCD |  | N8(Deamidated); C27(Dehydro); C33(Dehydro) |  | 4.85 |  | 1.3e-3 |  | 7.4e-5 |  | 1.76 |  | 329.37 |  | 1.000 |  | b1+(8): 770.44; b1+(9): 883.52; b1+(10): 996.61; b1+(13): 1237.75; y1+(18): 1865.91; y1+(17): 1778.88; y1+(15): 1636.80; y1+(14): 1508.74; y1+(13): 1421.71; y1+(12): 1322.64; y1+(11): 1209.56; y1+(9): 980.42; y1+(8): 867.33; y1+(7): 780.30; b2+(16): 755.45; b2+(18): 826.49; y2+(25): 1259.67; y2+(24): 1203.13 |  | N8(Deamidated): 100; C27(Dehydro): 100; C33(Dehydro): 100 |  |  |  |  |  |  |  |  |  | 2 |  | Sequest HT (3) |  | 3 |  | 1096.59363 |  | -1.16 |  | 47.1792 |  | 14193 |  | 14193 |  | TRINITY\_DN0\_c1\_g1\_i15.p1; TRINITY\_DN0\_c1\_g1\_i23.p1; TRINITY\_DN0\_c1\_g1\_i11.p1; TRINITY\_DN0\_c1\_g1\_i10.p1 |  | Image |  | Peak List |  |
|  | | | | | | | | | | | | | | | | | | | | | | | | | | | | | | | | | | | | | | | | | | | | | | | | | | | | | |  |
|  | High |  | [R].GVLGTVKnLLIGAGKSAAQSVLKTLScKLSNDc.[-] |  | HCD |  | N8(Deamidated); C27(Dehydro); C33(Dehydro) |  | 5.38 |  | 1.3e-3 |  | 2.5e-3 |  | 0.996 |  | 261.29 |  | 1.000 |  | b1+(8): 770.44; b1+(9): 883.52; b1+(10): 996.61; b1+(11): 1109.69; b1+(12): 1166.71; b1+(13): 1237.75; y1+(14): 1508.74; y1+(12): 1322.64; y1+(11): 1209.56; y1+(10): 1081.47; y1+(9): 980.42; y1+(8): 867.33; y2+(22): 1090.05; y2+(20): 1026.02; y2+(18): 933.46; y2+(12): 661.83; y2+(11): 605.28 |  | N8(Deamidated): 100; C27(Dehydro): 100; C33(Dehydro): 100 |  |  |  |  |  |  |  |  |  | 2 |  | Sequest HT (3) |  | 4 |  | 822.69855 |  | 0.67 |  | 47.3005 |  | 14243 |  | 14243 |  | TRINITY\_DN0\_c1\_g1\_i15.p1; TRINITY\_DN0\_c1\_g1\_i23.p1; TRINITY\_DN0\_c1\_g1\_i11.p1; TRINITY\_DN0\_c1\_g1\_i10.p1 |  | Image |  | Peak List |  |
|  | | | | | | | | | | | | | | | | | | | | | | | | | | | | | | | | | | | | | | | | | | | | | | | | | | | | | |  |
|  | High |  | [R].GVLGTVKnLLIGAGKSAAQSVLKTLScKLSNDc.[-] |  | HCD |  | N8(Deamidated); C27(Dehydro); C33(Dehydro) |  | 5.07 |  | 1.3e-3 |  | 3.3e-3 |  | 0.935 |  | 261.68 |  | 1.000 |  | b1+(8): 770.44; b1+(9): 883.52; b1+(10): 996.61; b1+(12): 1166.71; b1+(13): 1237.75; y1+(18): 1865.91; y1+(17): 1778.88; y1+(14): 1508.74; y1+(13): 1421.71; y1+(12): 1322.64; y1+(11): 1209.56; y1+(9): 980.42; y1+(8): 867.33; y1+(7): 780.30 |  | N8(Deamidated): 100; C27(Dehydro): 100; C33(Dehydro): 100 |  |  |  |  |  |  |  |  |  | 2 |  | Sequest HT (3) |  | 3 |  | 1096.59424 |  | -0.61 |  | 47.3213 |  | 14253 |  | 14253 |  | TRINITY\_DN0\_c1\_g1\_i15.p1; TRINITY\_DN0\_c1\_g1\_i23.p1; TRINITY\_DN0\_c1\_g1\_i11.p1; TRINITY\_DN0\_c1\_g1\_i10.p1 |  | Image |  | Peak List |  |
|  | | | | | | | | | | | | | | | | | | | | | | | | | | | | | | | | | | | | | | | | | | | | | | | | | | | | | |  |
|  | High |  | [R].GVLGTVKnLLIGAGKSAAQSVLKTLScKLSNDc.[-] |  | HCD |  | N8(Deamidated); C27(Dehydro); C33(Dehydro) |  | 2.67 |  | 3.9e-3 |  | 1.6e-1 |  | 0.072 |  | 52.98 |  | 0.500 |  | b1+(10): 996.61; y1+(11): 1209.56 |  | N8(Deamidated): 50; C27(Dehydro): 100; N31(Deamidated): 50; C33(Dehydro): 100 |  |  |  |  |  |  |  |  |  | 2 |  | Sequest HT (3) |  | 4 |  | 822.69836 |  | 0.45 |  | 47.4355 |  | 14299 |  | 14299 |  | TRINITY\_DN0\_c1\_g1\_i15.p1; TRINITY\_DN0\_c1\_g1\_i23.p1; TRINITY\_DN0\_c1\_g1\_i11.p1; TRINITY\_DN0\_c1\_g1\_i10.p1 |  | Image |  | Peak List |  |
|  | | | | | | | | | | | | | | | | | | | | | | | | | | | | | | | | | | | | | | | | | | | | | | | | | | | | | |  |
|  | High |  | [R].GVLGTVKnLLIGAGKSAAQSVLKTLScKLSNDc.[-] |  | HCD |  | N8(Deamidated); C27(Dehydro); C33(Dehydro) |  | 4.65 |  | 1.3e-3 |  | 7.4e-5 |  | 1.76 |  | 327.70 |  | 1.000 |  | b1+(8): 770.44; b1+(9): 883.52; b1+(10): 996.61; b1+(13): 1237.75; b1+(14): 1294.77; y1+(18): 1865.91; y1+(17): 1778.88; y1+(16): 1707.84; y1+(15): 1636.80; y1+(14): 1508.74; y1+(13): 1421.71; y1+(12): 1322.64; y1+(11): 1209.56; y1+(9): 980.42; y1+(8): 867.33; y1+(7): 780.30; b2+(17): 790.97; y2+(25): 1259.67 |  | N8(Deamidated): 100; C27(Dehydro): 100; C33(Dehydro): 100 |  |  |  |  |  |  |  |  |  | 2 |  | Sequest HT (3) |  | 3 |  | 1096.59473 |  | -0.16 |  | 47.4638 |  | 14312 |  | 14312 |  | TRINITY\_DN0\_c1\_g1\_i15.p1; TRINITY\_DN0\_c1\_g1\_i23.p1; TRINITY\_DN0\_c1\_g1\_i11.p1; TRINITY\_DN0\_c1\_g1\_i10.p1 |  | Image |  | Peak List |  |
|  | | | | | | | | | | | | | | | | | | | | | | | | | | | | | | | | | | | | | | | | | | | | | | | | | | | | | |  |
|  | High |  | [R].GVLGTVKnLLIGAGKSAAQSVLKTLScKLSnDc.[-] |  | HCD |  | N8(Deamidated); C27(Dehydro); N31(Deamidated); C33(Dehydro) |  | 4.96 |  | 1.3e-3 |  | 4.9e-3 |  | 0.848 |  | 274.90 |  | 1.000 |  |  |  | N8(Deamidated): 100; C27(Dehydro): 100; N31(Deamidated): 100; C33(Dehydro): 100 |  |  |  |  |  |  |  |  |  | 2 |  | Sequest HT (3) |  | 4 |  | 822.94580 |  | 2.19 |  | 47.5771 |  | 14357 |  | 14357 |  | TRINITY\_DN0\_c1\_g1\_i15.p1; TRINITY\_DN0\_c1\_g1\_i23.p1; TRINITY\_DN0\_c1\_g1\_i11.p1; TRINITY\_DN0\_c1\_g1\_i10.p1 |  | Image |  | Peak List |  |
|  | | | | | | | | | | | | | | | | | | | | | | | | | | | | | | | | | | | | | | | | | | | | | | | | | | | | | |  |
|  | High |  | [R].GVLGTVKnLLIGAGKSAAQSVLKTLScKLSnDc.[-] |  | HCD |  | N8(Deamidated); C27(Dehydro); N31(Deamidated); C33(Dehydro) |  | 3.03 |  | 1.3e-3 |  | 3.9e-3 |  | 0.899 |  | 222.58 |  | 1.000 |  |  |  | N8(Deamidated): 100; C27(Dehydro): 100; N31(Deamidated): 100; C33(Dehydro): 100 |  |  |  |  |  |  |  |  |  | 2 |  | Sequest HT (3) |  | 3 |  | 1096.92395 |  | 0.95 |  | 47.6021 |  | 14367 |  | 14367 |  | TRINITY\_DN0\_c1\_g1\_i15.p1; TRINITY\_DN0\_c1\_g1\_i23.p1; TRINITY\_DN0\_c1\_g1\_i11.p1; TRINITY\_DN0\_c1\_g1\_i10.p1 |  | Image |  | Peak List |  |
|  | | | | | | | | | | | | | | | | | | | | | | | | | | | | | | | | | | | | | | | | | | | | | | | | | | | | | |  |
|  | High |  | [R].GVLGTVKnLLIGAGKSAAQSVLKTLScKLSnDc.[-] |  | HCD |  | N8(Deamidated); C27(Dehydro); N31(Deamidated); C33(Dehydro) |  | 3.03 |  | 1.3e-3 |  | 5.9e-2 |  | 0.302 |  | 130.53 |  | 1.000 |  |  |  | N8(Deamidated): 100; C27(Dehydro): 100; N31(Deamidated): 100; C33(Dehydro): 100 |  |  |  |  |  |  |  |  |  | 2 |  | Sequest HT (3) |  | 5 |  | 658.55658 |  | -0.12 |  | 47.6302 |  | 14378 |  | 14378 |  | TRINITY\_DN0\_c1\_g1\_i15.p1; TRINITY\_DN0\_c1\_g1\_i23.p1; TRINITY\_DN0\_c1\_g1\_i11.p1; TRINITY\_DN0\_c1\_g1\_i10.p1 |  | Image |  | Peak List |  |
|  | | | | | | | | | | | | | | | | | | | | | | | | | | | | | | | | | | | | | | | | | | | | | | | | | | | | | |  |
|  | High |  | [R].GVLGTVKnLLIGAGKSAAQSVLKTLScKLSnDc.[-] |  | HCD |  | N8(Deamidated); C27(Dehydro); N31(Deamidated); C33(Dehydro) |  | 4.82 |  | 1.3e-3 |  | 1.2e-2 |  | 0.657 |  | 257.64 |  | 1.000 |  |  |  | N8(Deamidated): 100; C27(Dehydro): 100; N31(Deamidated): 100; C33(Dehydro): 100 |  |  |  |  |  |  |  |  |  | 2 |  | Sequest HT (3) |  | 4 |  | 822.94434 |  | 0.41 |  | 47.7156 |  | 14414 |  | 14414 |  | TRINITY\_DN0\_c1\_g1\_i15.p1; TRINITY\_DN0\_c1\_g1\_i23.p1; TRINITY\_DN0\_c1\_g1\_i11.p1; TRINITY\_DN0\_c1\_g1\_i10.p1 |  | Image |  | Peak List |  |
|  | | | | | | | | | | | | | | | | | | | | | | | | | | | | | | | | | | | | | | | | | | | | | | | | | | | | | |  |
|  | High |  | [R].GVLGTVKnLLIGAGKSAAQSVLKTLScKLSnDc.[-] |  | HCD |  | N8(Deamidated); C27(Dehydro); N31(Deamidated); C33(Dehydro) |  | 4.94 |  | 1.3e-3 |  | 2.2e-5 |  | 2.02 |  | 393.47 |  | 1.000 |  |  |  | N8(Deamidated): 100; C27(Dehydro): 100; N31(Deamidated): 100; C33(Dehydro): 100 |  |  |  |  |  |  |  |  |  | 2 |  | Sequest HT (3) |  | 3 |  | 1096.92297 |  | 0.06 |  | 47.7425 |  | 14429 |  | 14429 |  | TRINITY\_DN0\_c1\_g1\_i15.p1; TRINITY\_DN0\_c1\_g1\_i23.p1; TRINITY\_DN0\_c1\_g1\_i11.p1; TRINITY\_DN0\_c1\_g1\_i10.p1 |  | Image |  | Peak List |  |
|  | | | | | | | | | | | | | | | | | | | | | | | | | | | | | | | | | | | | | | | | | | | | | | | | | | | | | |  |
|  | High |  | [R].GVLGTVKnLLIGAGKSAAQSVLKTLScKLSnDc.[-] |  | HCD |  | N8(Deamidated); C27(Dehydro); N31(Deamidated); C33(Dehydro) |  | 3.44 |  | 1.3e-3 |  | 1.8e-2 |  | 0.562 |  | 188.61 |  | 1.000 |  |  |  | N8(Deamidated): 100; C27(Dehydro): 100; N31(Deamidated): 100; C33(Dehydro): 100 |  |  |  |  |  |  |  |  |  | 2 |  | Sequest HT (3) |  | 5 |  | 658.55634 |  | -0.49 |  | 47.7761 |  | 14440 |  | 14440 |  | TRINITY\_DN0\_c1\_g1\_i15.p1; TRINITY\_DN0\_c1\_g1\_i23.p1; TRINITY\_DN0\_c1\_g1\_i11.p1; TRINITY\_DN0\_c1\_g1\_i10.p1 |  | Image |  | Peak List |  |
|  | | | | | | | | | | | | | | | | | | | | | | | | | | | | | | | | | | | | | | | | | | | | | | | | | | | | | |  |
|  | High |  | [R].GVLGTVKnLLIGAGKSAAQSVLKTLScKLSnDc.[-] |  | HCD |  | N8(Deamidated); C27(Dehydro); N31(Deamidated); C33(Dehydro) |  | 4.40 |  | 1.3e-3 |  | 3.0e-4 |  | 1.46 |  | 344.31 |  | 1.000 |  |  |  | N8(Deamidated): 100; C27(Dehydro): 100; N31(Deamidated): 100; C33(Dehydro): 100 |  |  |  |  |  |  |  |  |  | 2 |  | Sequest HT (3) |  | 4 |  | 822.94409 |  | 0.11 |  | 47.8536 |  | 14470 |  | 14470 |  | TRINITY\_DN0\_c1\_g1\_i15.p1; TRINITY\_DN0\_c1\_g1\_i23.p1; TRINITY\_DN0\_c1\_g1\_i11.p1; TRINITY\_DN0\_c1\_g1\_i10.p1 |  | Image |  | Peak List |  |
|  | | | | | | | | | | | | | | | | | | | | | | | | | | | | | | | | | | | | | | | | | | | | | | | | | | | | | |  |
|  | High |  | [R].GVLGTVKnLLIGAGKSAAQSVLKTLScKLSnDc.[-] |  | HCD |  | N8(Deamidated); C27(Dehydro); N31(Deamidated); C33(Dehydro) |  | 5.44 |  | 1.3e-3 |  | 4.5e-5 |  | 1.87 |  | 433.52 |  | 1.000 |  |  |  | N8(Deamidated): 100; C27(Dehydro): 100; N31(Deamidated): 100; C33(Dehydro): 100 |  |  |  |  |  |  |  |  |  | 2 |  | Sequest HT (3) |  | 3 |  | 1096.92273 |  | -0.16 |  | 47.8884 |  | 14488 |  | 14488 |  | TRINITY\_DN0\_c1\_g1\_i15.p1; TRINITY\_DN0\_c1\_g1\_i23.p1; TRINITY\_DN0\_c1\_g1\_i11.p1; TRINITY\_DN0\_c1\_g1\_i10.p1 |  | Image |  | Peak List |  |
|  | | | | | | | | | | | | | | | | | | | | | | | | | | | | | | | | | | | | | | | | | | | | | | | | | | | | | |  |
|  | High |  | [R].GVLGTVKnLLIGAGKSAAQSVLKTLSCKLSNDc.[-] |  | HCD |  | N8(Deamidated); C33(Dehydro) |  | 3.23 |  | 3.1e-3 |  | 1.2e-1 |  | 0.134 |  | 140.09 |  | 0.500 |  |  |  | N8(Deamidated): 100; C27(Dehydro): 50; C33(Dehydro): 50 |  |  |  |  |  |  |  |  |  | 2 |  | Sequest HT (3) |  | 4 |  | 822.94763 |  | -2.82 |  | 48.1349 |  | 14591 |  | 14591 |  | TRINITY\_DN0\_c1\_g1\_i15.p1; TRINITY\_DN0\_c1\_g1\_i23.p1; TRINITY\_DN0\_c1\_g1\_i11.p1; TRINITY\_DN0\_c1\_g1\_i10.p1 |  | Image |  | Peak List |  |
|  | | | | | | | | | | | | | | | | | | | | | | | | | | | | | | | | | | | | | | | | | | | | | | | | | | | | | |  |
|  | High |  | [R].GVLGTVKnLLIGAGKSAAQSVLKTLScKLSNDC.[-] |  | HCD |  | N8(Deamidated); C27(Dehydro) |  | 3.23 |  | 3.1e-3 |  | 1.2e-1 |  | 0.134 |  | 140.09 |  | 0.500 |  |  |  | N8(Deamidated): 100; C27(Dehydro): 50; C33(Dehydro): 50 |  |  |  |  |  |  |  |  |  | 4 |  | Sequest HT (3) |  | 4 |  | 822.94763 |  | -2.82 |  | 48.1349 |  | 14591 |  | 14591 |  | TRINITY\_DN0\_c1\_g1\_i15.p1; TRINITY\_DN0\_c1\_g1\_i23.p1; TRINITY\_DN0\_c1\_g1\_i11.p1; TRINITY\_DN0\_c1\_g1\_i10.p1 |  | Image |  | Peak List |  |
|  | | | | | | | | | | | | | | | | | | | | | | | | | | | | | | | | | | | | | | | | | | | | | | | | | | | | | |  |
|  | High |  | [R].GVLGTVKNLLIGAGKSAAQSVLKTLSCKLSnDc.[-] |  | HCD |  | N31(Deamidated); C33(Dehydro) |  | 3.20 |  | 1.3e-3 |  | 4.4e-2 |  | 0.367 |  | 59.81 |  | 0.000 |  |  |  | N8(Deamidated): 100; C27(Dehydro): 50; C33(Dehydro): 50 |  |  |  |  |  |  |  |  |  | 5 |  | Sequest HT (3) |  | 4 |  | 822.94763 |  | -2.82 |  | 48.1349 |  | 14591 |  | 14591 |  | TRINITY\_DN0\_c1\_g1\_i15.p1; TRINITY\_DN0\_c1\_g1\_i23.p1; TRINITY\_DN0\_c1\_g1\_i11.p1; TRINITY\_DN0\_c1\_g1\_i10.p1 |  | Image |  | Peak List |  |
|  | | | | | | | | | | | | | | | | | | | | | | | | | | | | | | | | | | | | | | | | | | | | | | | | | | | | | |  |
|  | High |  | [R].GVLGTVKNLLIGAGKSAAQSVLKTLScKLSnDC.[-] |  | HCD |  | C27(Dehydro); N31(Deamidated) |  | 3.20 |  | 1.3e-3 |  | 4.4e-2 |  | 0.367 |  |  |  |  |  |  |  |  |  |  |  |  |  |  |  |  |  | 5 |  | Sequest HT (3) |  | 4 |  | 822.94763 |  | -2.82 |  | 48.1349 |  | 14591 |  | 14591 |  | TRINITY\_DN0\_c1\_g1\_i15.p1; TRINITY\_DN0\_c1\_g1\_i23.p1; TRINITY\_DN0\_c1\_g1\_i11.p1; TRINITY\_DN0\_c1\_g1\_i10.p1 |  | Image |  | Peak List |  |
|  | | | | | | | | | | | | | | | | | | | | | | | | | | | | | | | | | | | | | | | | | | | | | | | | | | | | | |  |
|  | High |  | [R].GVLGTVKnLLIGAGKSAAQSVLKTLScKLSnDc.[-] |  | HCD |  | N8(Deamidated); C27(Dehydro); N31(Deamidated); C33(Dehydro) |  | 4.44 |  | 1.3e-3 |  | 1.0e-2 |  | 0.684 |  | 285.62 |  | 1.000 |  |  |  | N8(Deamidated): 100; C27(Dehydro): 100; N31(Deamidated): 100; C33(Dehydro): 100 |  |  |  |  |  |  |  |  |  | 2 |  | Sequest HT (3) |  | 3 |  | 1096.92590 |  | 2.73 |  | 48.1923 |  | 14616 |  | 14616 |  | TRINITY\_DN0\_c1\_g1\_i15.p1; TRINITY\_DN0\_c1\_g1\_i23.p1; TRINITY\_DN0\_c1\_g1\_i11.p1; TRINITY\_DN0\_c1\_g1\_i10.p1 |  | Image |  | Peak List |  |
|  | | | | | | | | | | | | | | | | | | | | | | | | | | | | | | | | | | | | | | | | | | | | | | | | | | | | | |  |
|  | High |  | [R].GVLGTVKnLLIGAGKSAAQSVLKTLScKLSnDc.[-] |  | HCD |  | N8(Deamidated); C27(Dehydro); N31(Deamidated); C33(Dehydro) |  | 3.71 |  | 1.3e-3 |  | 2.5e-3 |  | 0.995 |  | 265.09 |  | 1.000 |  |  |  | N8(Deamidated): 100; C27(Dehydro): 100; N31(Deamidated): 100; C33(Dehydro): 100 |  |  |  |  |  |  |  |  |  | 2 |  | Sequest HT (3) |  | 4 |  | 822.94574 |  | 2.11 |  | 48.3787 |  | 14704 |  | 14704 |  | TRINITY\_DN0\_c1\_g1\_i15.p1; TRINITY\_DN0\_c1\_g1\_i23.p1; TRINITY\_DN0\_c1\_g1\_i11.p1; TRINITY\_DN0\_c1\_g1\_i10.p1 |  | Image |  | Peak List |  |
|  | | | | | | | | | | | | | | | | | | | | | | | | | | | | | | | | | | | | | | | | | | | | | | | | | | | | | |  |
|  | High |  | [R].GVLGTVKnLLIGAGKSAAQSVLKTLScKLSnDc.[-] |  | HCD |  | N8(Deamidated); C27(Dehydro); N31(Deamidated); C33(Dehydro) |  | 2.77 |  | 1.3e-3 |  | 4.0e-2 |  | 0.384 |  | 185.57 |  | 1.000 |  |  |  | N8(Deamidated): 100; C27(Dehydro): 100; N31(Deamidated): 100; C33(Dehydro): 100 |  |  |  |  |  |  |  |  |  | 2 |  | Sequest HT (3) |  | 4 |  | 822.94354 |  | -0.56 |  | 49.8897 |  | 15307 |  | 15307 |  | TRINITY\_DN0\_c1\_g1\_i15.p1; TRINITY\_DN0\_c1\_g1\_i23.p1; TRINITY\_DN0\_c1\_g1\_i11.p1; TRINITY\_DN0\_c1\_g1\_i10.p1 |  | Image |  | Peak List |  |
|  | | | | | | | | | | | | | | | | | | | | | | | | | | | | | | | | | | | | | | | | | | | | | | | | | | | | | |  |
|  | High |  | [R].GVLGTVKnLLIGAGKSAAQSVLKTLScKLSnDc.[-] |  | HCD |  | N8(Deamidated); C27(Dehydro); N31(Deamidated); C33(Dehydro) |  | 3.83 |  | 1.3e-3 |  | 3.1e-3 |  | 0.945 |  | 278.68 |  | 1.000 |  |  |  | N8(Deamidated): 100; C27(Dehydro): 100; N31(Deamidated): 100; C33(Dehydro): 100 |  |  |  |  |  |  |  |  |  | 2 |  | Sequest HT (3) |  | 3 |  | 1096.92285 |  | -0.05 |  | 49.9609 |  | 15335 |  | 15335 |  | TRINITY\_DN0\_c1\_g1\_i15.p1; TRINITY\_DN0\_c1\_g1\_i23.p1; TRINITY\_DN0\_c1\_g1\_i11.p1; TRINITY\_DN0\_c1\_g1\_i10.p1 |  | Image |  | Peak List |  |
|  | | | | | | | | | | | | | | | | | | | | | | | | | | | | | | | | | | | | | | | | | | | | | | | | | | | | | |  |
|  | High |  | [R].GVLGTVKnLLIGAGKSAAQSVLKTLScKLSnDc.[-] |  | HCD |  | N8(Deamidated); C27(Dehydro); N31(Deamidated); C33(Dehydro) |  | 3.17 |  | 1.3e-3 |  | 2.9e-2 |  | 0.46 |  | 213.99 |  | 1.000 |  |  |  | N8(Deamidated): 100; C27(Dehydro): 100; N31(Deamidated): 100; C33(Dehydro): 100 |  |  |  |  |  |  |  |  |  | 2 |  | Sequest HT (3) |  | 4 |  | 822.94318 |  | -1.00 |  | 50.0347 |  | 15364 |  | 15364 |  | TRINITY\_DN0\_c1\_g1\_i15.p1; TRINITY\_DN0\_c1\_g1\_i23.p1; TRINITY\_DN0\_c1\_g1\_i11.p1; TRINITY\_DN0\_c1\_g1\_i10.p1 |  | Image |  | Peak List |  |
|  | | | | | | | | | | | | | | | | | | | | | | | | | | | | | | | | | | | | | | | | | | | | | | | | | | | | | |  |
|  | High |  | [R].GVLGTVKnLLIGAGKSAAQSVLKTLScKLSnDc.[-] |  | HCD |  | N8(Deamidated); C27(Dehydro); N31(Deamidated); C33(Dehydro) |  | 4.34 |  | 1.3e-3 |  | 5.7e-3 |  | 0.813 |  | 242.98 |  | 1.000 |  |  |  | N8(Deamidated): 100; C27(Dehydro): 100; N31(Deamidated): 100; C33(Dehydro): 100 |  |  |  |  |  |  |  |  |  | 2 |  | Sequest HT (3) |  | 3 |  | 1096.92285 |  | -0.05 |  | 50.1023 |  | 15389 |  | 15389 |  | TRINITY\_DN0\_c1\_g1\_i15.p1; TRINITY\_DN0\_c1\_g1\_i23.p1; TRINITY\_DN0\_c1\_g1\_i11.p1; TRINITY\_DN0\_c1\_g1\_i10.p1 |  | Image |  | Peak List |  |
|  | | | | | | | | | | | | | | | | | | | | | | | | | | | | | | | | | | | | | | | | | | | | | | | | | | | | | |  |
|  | High |  | [R].GVLGTVKnLLIGAGKSAAQSVLKTLScKLSnDc.[-] |  | HCD |  | N8(Deamidated); C27(Dehydro); N31(Deamidated); C33(Dehydro) |  | 2.92 |  | 2.4e-3 |  | 7.9e-2 |  | 0.235 |  | 202.56 |  | 1.000 |  |  |  | N8(Deamidated): 100; C27(Dehydro): 100; N31(Deamidated): 100; C33(Dehydro): 100 |  |  |  |  |  |  |  |  |  | 2 |  | Sequest HT (3) |  | 4 |  | 822.94373 |  | -0.34 |  | 50.1827 |  | 15421 |  | 15421 |  | TRINITY\_DN0\_c1\_g1\_i15.p1; TRINITY\_DN0\_c1\_g1\_i23.p1; TRINITY\_DN0\_c1\_g1\_i11.p1; TRINITY\_DN0\_c1\_g1\_i10.p1 |  | Image |  | Peak List |  |
|  | | | | | | | | | | | | | | | | | | | | | | | | | | | | | | | | | | | | | | | | | | | | | | | | | | | | | |  |
|  | High |  | [R].GVLGTVKnLLIGAGKSAAQSVLKTLScKLSnDc.[-] |  | HCD |  | N8(Deamidated); C27(Dehydro); N31(Deamidated); C33(Dehydro) |  | 4.08 |  | 1.3e-3 |  | 7.7e-4 |  | 1.25 |  | 352.26 |  | 1.000 |  |  |  | N8(Deamidated): 100; C27(Dehydro): 100; N31(Deamidated): 100; C33(Dehydro): 100 |  |  |  |  |  |  |  |  |  | 2 |  | Sequest HT (3) |  | 3 |  | 1096.92273 |  | -0.16 |  | 50.2514 |  | 15449 |  | 15449 |  | TRINITY\_DN0\_c1\_g1\_i15.p1; TRINITY\_DN0\_c1\_g1\_i23.p1; TRINITY\_DN0\_c1\_g1\_i11.p1; TRINITY\_DN0\_c1\_g1\_i10.p1 |  | Image |  | Peak List |  |
|  | | | | | | | | | | | | | | | | | | | | | | | | | | | | | | | | | | | | | | | | | | | | | | | | | | | | | |  |
|  | High |  | [R].GVLGTVKnLLIGAGKSAAQSVLKTLScKLSnDc.[-] |  | HCD |  | N8(Deamidated); C27(Dehydro); N31(Deamidated); C33(Dehydro) |  | 2.85 |  | 1.3e-3 |  | 1.1e-2 |  | 0.677 |  | 203.69 |  | 1.000 |  |  |  | N8(Deamidated): 100; C27(Dehydro): 100; N31(Deamidated): 100; C33(Dehydro): 100 |  |  |  |  |  |  |  |  |  | 2 |  | Sequest HT (3) |  | 4 |  | 822.94415 |  | 0.18 |  | 50.3211 |  | 15475 |  | 15475 |  | TRINITY\_DN0\_c1\_g1\_i15.p1; TRINITY\_DN0\_c1\_g1\_i23.p1; TRINITY\_DN0\_c1\_g1\_i11.p1; TRINITY\_DN0\_c1\_g1\_i10.p1 |  | Image |  | Peak List |  |
|  | | | | | | | | | | | | | | | | | | | | | | | | | | | | | | | | | | | | | | | | | | | | | | | | | | | | | |  |
|  | High |  | [R].GVLGTVKnLLIGAGKSAAQSVLKTLScKLSnDc.[-] |  | HCD |  | N8(Deamidated); C27(Dehydro); N31(Deamidated); C33(Dehydro) |  | 3.52 |  | 1.3e-3 |  | 2.9e-3 |  | 0.96 |  | 296.77 |  | 1.000 |  |  |  | N8(Deamidated): 100; C27(Dehydro): 100; N31(Deamidated): 100; C33(Dehydro): 100 |  |  |  |  |  |  |  |  |  | 2 |  | Sequest HT (3) |  | 3 |  | 1096.92273 |  | -0.16 |  | 50.3919 |  | 15505 |  | 15505 |  | TRINITY\_DN0\_c1\_g1\_i15.p1; TRINITY\_DN0\_c1\_g1\_i23.p1; TRINITY\_DN0\_c1\_g1\_i11.p1; TRINITY\_DN0\_c1\_g1\_i10.p1 |  | Image |  | Peak List |  |
|  | | | | | | | | | | | | | | | | | | | | | | | | | | | | | | | | | | | | | | | | | | | | | | | | | | | | | |  |
|  | High |  | [R].GVLGTVKnLLIGAGKSAAQSVLKTLScKLSnDc.[-] |  | HCD |  | N8(Deamidated); C27(Dehydro); N31(Deamidated); C33(Dehydro) |  | 2.56 |  | 3.1e-3 |  | 1.0e-1 |  | 0.182 |  | 146.53 |  | 1.000 |  |  |  | N8(Deamidated): 100; C27(Dehydro): 100; N31(Deamidated): 100; C33(Dehydro): 100 |  |  |  |  |  |  |  |  |  | 2 |  | Sequest HT (3) |  | 4 |  | 822.94385 |  | -0.19 |  | 50.4601 |  | 15531 |  | 15531 |  | TRINITY\_DN0\_c1\_g1\_i15.p1; TRINITY\_DN0\_c1\_g1\_i23.p1; TRINITY\_DN0\_c1\_g1\_i11.p1; TRINITY\_DN0\_c1\_g1\_i10.p1 |  | Image |  | Peak List |  |
|  | | | | | | | | | | | | | | | | | | | | | | | | | | | | | | | | | | | | | | | | | | | | | | | | | | | | | |  |
|  | High |  | [R].GVLGTVKnLLIGAGKSAAQSVLKTLScKLSnDc.[-] |  | HCD |  | N8(Deamidated); C27(Dehydro); N31(Deamidated); C33(Dehydro) |  | 4.30 |  | 1.3e-3 |  | 2.5e-3 |  | 0.995 |  | 322.85 |  | 1.000 |  |  |  | N8(Deamidated): 100; C27(Dehydro): 100; N31(Deamidated): 100; C33(Dehydro): 100 |  |  |  |  |  |  |  |  |  | 2 |  | Sequest HT (3) |  | 3 |  | 1096.92236 |  | -0.50 |  | 50.5401 |  | 15564 |  | 15564 |  | TRINITY\_DN0\_c1\_g1\_i15.p1; TRINITY\_DN0\_c1\_g1\_i23.p1; TRINITY\_DN0\_c1\_g1\_i11.p1; TRINITY\_DN0\_c1\_g1\_i10.p1 |  | Image |  | Peak List |  |
|  | | | | | | | | | | | | | | | | | | | | | | | | | | | | | | | | | | | | | | | | | | | | | | | | | | | | | |  |
|  | High |  | [R].GVLGTVKnLLIGAGKSAAQSVLKTLScKLSnDc.[-] |  | HCD |  | N8(Deamidated); C27(Dehydro); N31(Deamidated); C33(Dehydro) |  | 2.31 |  | 6.5e-3 |  | 2.2e-1 |  | 0 |  | 132.92 |  | 1.000 |  |  |  | N8(Deamidated): 100; C27(Dehydro): 100; N31(Deamidated): 100; C33(Dehydro): 100 |  |  |  |  |  |  |  |  |  | 2 |  | Sequest HT (3) |  | 5 |  | 658.55640 |  | -0.39 |  | 50.5414 |  | 15565 |  | 15565 |  | TRINITY\_DN0\_c1\_g1\_i15.p1; TRINITY\_DN0\_c1\_g1\_i23.p1; TRINITY\_DN0\_c1\_g1\_i11.p1; TRINITY\_DN0\_c1\_g1\_i10.p1 |  | Image |  | Peak List |  |
|  | | | | | | | | | | | | | | | | | | | | | | | | | | | | | | | | | | | | | | | | | | | | | | | | | | | | | |  |
|  | High |  | [R].GVLGTVKnLLIGAGKSAAQSVLKTLScKLSnDc.[-] |  | HCD |  | N8(Deamidated); C27(Dehydro); N31(Deamidated); C33(Dehydro) |  | 2.85 |  | 1.3e-3 |  | 1.7e-2 |  | 0.574 |  | 200.47 |  | 1.000 |  |  |  | N8(Deamidated): 100; C27(Dehydro): 100; N31(Deamidated): 100; C33(Dehydro): 100 |  |  |  |  |  |  |  |  |  | 2 |  | Sequest HT (3) |  | 4 |  | 822.94348 |  | -0.63 |  | 50.6018 |  | 15585 |  | 15585 |  | TRINITY\_DN0\_c1\_g1\_i15.p1; TRINITY\_DN0\_c1\_g1\_i23.p1; TRINITY\_DN0\_c1\_g1\_i11.p1; TRINITY\_DN0\_c1\_g1\_i10.p1 |  | Image |  | Peak List |  |
|  | | | | | | | | | | | | | | | | | | | | | | | | | | | | | | | | | | | | | | | | | | | | | | | | | | | | | |  |
|  | High |  | [R].GVLGTVKnLLIGAGKSAAQSVLKTLScKLSnDc.[-] |  | HCD |  | N8(Deamidated); C27(Dehydro); N31(Deamidated); C33(Dehydro) |  | 3.27 |  | 3.1e-3 |  | 9.7e-2 |  | 0.189 |  | 166.18 |  | 1.000 |  |  |  | N8(Deamidated): 100; C27(Dehydro): 100; N31(Deamidated): 100; C33(Dehydro): 100 |  |  |  |  |  |  |  |  |  | 2 |  | Sequest HT (3) |  | 4 |  | 822.94415 |  | 0.18 |  | 50.7415 |  | 15639 |  | 15639 |  | TRINITY\_DN0\_c1\_g1\_i15.p1; TRINITY\_DN0\_c1\_g1\_i23.p1; TRINITY\_DN0\_c1\_g1\_i11.p1; TRINITY\_DN0\_c1\_g1\_i10.p1 |  | Image |  | Peak List |  |
|  | | | | | | | | | | | | | | | | | | | | | | | | | | | | | | | | | | | | | | | | | | | | | | | | | | | | | |  |
|  | High |  | [R].GVLGTVKnLLIGAGKSAAQSVLKTLScKLSnDc.[-] |  | HCD |  | N8(Deamidated); C27(Dehydro); N31(Deamidated); C33(Dehydro) |  | 4.43 |  | 1.3e-3 |  | 3.2e-3 |  | 0.942 |  | 313.01 |  | 1.000 |  |  |  | N8(Deamidated): 100; C27(Dehydro): 100; N31(Deamidated): 100; C33(Dehydro): 100 |  |  |  |  |  |  |  |  |  | 2 |  | Sequest HT (3) |  | 3 |  | 1096.92456 |  | 1.51 |  | 50.8327 |  | 15672 |  | 15672 |  | TRINITY\_DN0\_c1\_g1\_i15.p1; TRINITY\_DN0\_c1\_g1\_i23.p1; TRINITY\_DN0\_c1\_g1\_i11.p1; TRINITY\_DN0\_c1\_g1\_i10.p1 |  | Image |  | Peak List |  |
|  | | | | | | | | | | | | | | | | | | | | | | | | | | | | | | | | | | | | | | | | | | | | | | | | | | | | | |  |
|  | High |  | [R].GVLGTVKnLLIGAGKSAAQSVLKTLScKLSnDc.[-] |  | HCD |  | N8(Deamidated); C27(Dehydro); N31(Deamidated); C33(Dehydro) |  | 3.82 |  | 1.3e-3 |  | 1.8e-2 |  | 0.567 |  | 203.90 |  | 1.000 |  |  |  | N8(Deamidated): 100; C27(Dehydro): 100; N31(Deamidated): 100; C33(Dehydro): 100 |  |  |  |  |  |  |  |  |  | 2 |  | Sequest HT (3) |  | 4 |  | 822.94373 |  | -0.34 |  | 50.8767 |  | 15689 |  | 15689 |  | TRINITY\_DN0\_c1\_g1\_i15.p1; TRINITY\_DN0\_c1\_g1\_i23.p1; TRINITY\_DN0\_c1\_g1\_i11.p1; TRINITY\_DN0\_c1\_g1\_i10.p1 |  | Image |  | Peak List |  |
|  | | | | | | | | | | | | | | | | | | | | | | | | | | | | | | | | | | | | | | | | | | | | | | | | | | | | | |  |
|  | High |  | [R].GVLGTVKnLLIGAGKSAAQSVLKTLScKLSnDc.[-] |  | HCD |  | N8(Deamidated); C27(Dehydro); N31(Deamidated); C33(Dehydro) |  | 4.89 |  | 1.3e-3 |  | 2.1e-3 |  | 1.04 |  | 322.85 |  | 1.000 |  |  |  | N8(Deamidated): 100; C27(Dehydro): 100; N31(Deamidated): 100; C33(Dehydro): 100 |  |  |  |  |  |  |  |  |  | 2 |  | Sequest HT (3) |  | 3 |  | 1096.92029 |  | -2.39 |  | 50.9735 |  | 15729 |  | 15729 |  | TRINITY\_DN0\_c1\_g1\_i15.p1; TRINITY\_DN0\_c1\_g1\_i23.p1; TRINITY\_DN0\_c1\_g1\_i11.p1; TRINITY\_DN0\_c1\_g1\_i10.p1 |  | Image |  | Peak List |  |
|  | | | | | | | | | | | | | | | | | | | | | | | | | | | | | | | | | | | | | | | | | | | | | | | | | | | | | |  |
|  | High |  | [R].GVLGTVKnLLIGAGKSAAQSVLKTLScKLSnDc.[-] |  | HCD |  | N8(Deamidated); C27(Dehydro); N31(Deamidated); C33(Dehydro) |  | 4.22 |  | 1.3e-3 |  | 1.2e-3 |  | 1.16 |  | 247.30 |  | 1.000 |  |  |  | N8(Deamidated): 100; C27(Dehydro): 100; N31(Deamidated): 100; C33(Dehydro): 100 |  |  |  |  |  |  |  |  |  | 2 |  | Sequest HT (3) |  | 3 |  | 1096.92053 |  | -2.17 |  | 51.1209 |  | 15790 |  | 15790 |  | TRINITY\_DN0\_c1\_g1\_i15.p1; TRINITY\_DN0\_c1\_g1\_i23.p1; TRINITY\_DN0\_c1\_g1\_i11.p1; TRINITY\_DN0\_c1\_g1\_i10.p1 |  | Image |  | Peak List |  |
|  | | | | | | | | | | | | | | | | | | | | | | | | | | | | | | | | | | | | | | | | | | | | | | | | | | | | | |  |
|  | High |  | [R].GVLGTVKnLLIGAGKSAAQSVLKTLScKLSnDc.[-] |  | HCD |  | N8(Deamidated); C27(Dehydro); N31(Deamidated); C33(Dehydro) |  | 3.47 |  | 1.3e-3 |  | 2.2e-2 |  | 0.523 |  | 187.02 |  | 1.000 |  |  |  | N8(Deamidated): 100; C27(Dehydro): 100; N31(Deamidated): 100; C33(Dehydro): 100 |  |  |  |  |  |  |  |  |  | 2 |  | Sequest HT (3) |  | 4 |  | 822.94427 |  | 0.33 |  | 51.2191 |  | 15826 |  | 15826 |  | TRINITY\_DN0\_c1\_g1\_i15.p1; TRINITY\_DN0\_c1\_g1\_i23.p1; TRINITY\_DN0\_c1\_g1\_i11.p1; TRINITY\_DN0\_c1\_g1\_i10.p1 |  | Image |  | Peak List |  |
|  | | | | | | | | | | | | | | | | | | | | | | | | | | | | | | | | | | | | | | | | | | | | | | | | | | | | | |  |
|  | High |  | [R].GVLGTVKnLLIGAGKSAAQSVLKTLScKLSNDc.[-] |  | HCD |  | N8(Deamidated); C27(Dehydro); C33(Dehydro) |  | 2.25 |  | 1.3e-3 |  | 3.7e-2 |  | 0.402 |  | 117.06 |  | 0.978 |  | b1+(9): 883.52; b1+(10): 996.61; y1+(12): 1322.64; y1+(11): 1209.56; y1+(10): 1081.47; y1+(8): 867.33 |  | N8(Deamidated): 97.77; C27(Dehydro): 100; C33(Dehydro): 100 |  |  |  |  |  |  |  |  |  | 2 |  | Sequest HT (3) |  | 4 |  | 822.69843 |  | 0.52 |  | 62.1192 |  | 19514 |  | 19514 |  | TRINITY\_DN0\_c1\_g1\_i15.p1; TRINITY\_DN0\_c1\_g1\_i23.p1; TRINITY\_DN0\_c1\_g1\_i11.p1; TRINITY\_DN0\_c1\_g1\_i10.p1 |  | Image |  | Peak List |  |
|  | | | | | | | | | | | | | | | | | | | | | | | | | | | | | | | | | | | | | | | | | | | | | | | | | | | | | |  |
|  | High |  | [R].GVLGTVKnLLIGAGKSAAQSVLKTLScKLSNDc.[-] |  | HCD |  | N8(Deamidated); C27(Dehydro); C33(Dehydro) |  | 2.81 |  | 3.1e-3 |  | 1.3e-1 |  | 0.123 |  | 149.88 |  | 1.000 |  | b1+(8): 770.44; b1+(9): 883.52; b1+(10): 996.61; b1+(11): 1109.69; b1+(13): 1237.75; y1+(12): 1322.64; y1+(11): 1209.56; y1+(10): 1081.47; y1+(9): 980.42; y1+(8): 867.33; y2+(22): 1090.05 |  | N8(Deamidated): 100; C27(Dehydro): 100; C33(Dehydro): 100 |  |  |  |  |  |  |  |  |  | 2 |  | Sequest HT (3) |  | 4 |  | 822.69977 |  | 2.15 |  | 62.2904 |  | 19566 |  | 19566 |  | TRINITY\_DN0\_c1\_g1\_i15.p1; TRINITY\_DN0\_c1\_g1\_i23.p1; TRINITY\_DN0\_c1\_g1\_i11.p1; TRINITY\_DN0\_c1\_g1\_i10.p1 |  | Image |  | Peak List |  |
|  | | | | | | | | | | | | | | | | | | | | | | | | | | | | | | | | | | | | | | | | | | | | | | | | | | | | | |  |
|  | **TRINITY\_DN96\_c0\_g1\_i2.p1 - TRINITY\_DN96\_c0\_g1\_i2.p1** | | | | | | | | | | | | | | | | | | | | | | | | | | | | | | | | | | | | | | | | | | | | | | | | | | | |  |  |
|  | | | | | | | | | | | | | | | | | | | | | | | | | | | | | | | | | | | | | | | | | | | | | | | | | | | | | |  |
|  | High |  | [R].GIFAILKIATKLIGKTLAKAAGKAGTGLLAcKAAKEc.[-] |  | HCD |  | C31(Dehydro); C37(Dehydro) |  | 5.92 |  | 1.3e-3 |  | 5.9e-4 |  | 1.31 |  | 273.78 |  | 1.000 |  |  |  | C31(Dehydro): 100; C37(Dehydro): 100 |  |  |  |  |  |  |  | 0.73 |  | 1 |  | Sequest HT (3) |  | 5 |  | 729.03308 |  | -0.26 |  | 41.1069 |  | 11595 |  | 11595 |  | TRINITY\_DN96\_c0\_g1\_i2.p1 |  | Image |  | Peak List |  |
|  | | | | | | | | | | | | | | | | | | | | | | | | | | | | | | | | | | | | | | | | | | | | | | | | | | | | | |  |
|  | High |  | [R].GIFAILKIATKLIGKTLAKAAGKAGTGLLAcKAAKEc.[-] |  | HCD |  | C31(Dehydro); C37(Dehydro) |  | 7.91 |  | 1.3e-3 |  | 4.3e-4 |  | 1.38 |  | 254.26 |  | 1.000 |  |  |  | C31(Dehydro): 100; C37(Dehydro): 100 |  |  |  |  |  |  |  | 0.74 |  | 1 |  | Sequest HT (3) |  | 4 |  | 911.03851 |  | -1.38 |  | 41.1184 |  | 11601 |  | 11601 |  | TRINITY\_DN96\_c0\_g1\_i2.p1 |  | Image |  | Peak List |  |
|  | | | | | | | | | | | | | | | | | | | | | | | | | | | | | | | | | | | | | | | | | | | | | | | | | | | | | |  |
|  | High |  | [R].GIFAILKIATKLIGKTLAKAAGKAGTGLLAcKAAKEc.[-] |  | HCD |  | C31(Dehydro); C37(Dehydro) |  | 4.98 |  | 1.3e-3 |  | 2.5e-4 |  | 1.49 |  | 404.01 |  | 1.000 |  |  |  | C31(Dehydro): 100; C37(Dehydro): 100 |  |  |  |  |  |  |  | 0.68 |  | 1 |  | Sequest HT (3) |  | 3 |  | 1214.38611 |  | 1.79 |  | 41.1848 |  | 11629 |  | 11629 |  | TRINITY\_DN96\_c0\_g1\_i2.p1 |  | Image |  | Peak List |  |
|  | | | | | | | | | | | | | | | | | | | | | | | | | | | | | | | | | | | | | | | | | | | | | | | | | | | | | |  |
|  | High |  | [R].GIFAILKIATKLIGKTLAKAAGKAGTGLLAcKAAKEc.[-] |  | HCD |  | C31(Dehydro); C37(Dehydro) |  | 4.43 |  | 1.3e-3 |  | 4.5e-2 |  | 0.361 |  | 144.19 |  | 1.000 |  |  |  | C31(Dehydro): 100; C37(Dehydro): 100 |  |  |  |  |  |  |  | 0.58 |  | 1 |  | Sequest HT (3) |  | 5 |  | 729.03448 |  | 1.66 |  | 41.2498 |  | 11657 |  | 11657 |  | TRINITY\_DN96\_c0\_g1\_i2.p1 |  | Image |  | Peak List |  |
|  | | | | | | | | | | | | | | | | | | | | | | | | | | | | | | | | | | | | | | | | | | | | | | | | | | | | | |  |
|  | High |  | [R].GIFAILKIATKLIGKTLAKAAGKAGTGLLAcKAAKEc.[-] |  | HCD |  | C31(Dehydro); C37(Dehydro) |  | 4.76 |  | 1.3e-3 |  | 2.0e-2 |  | 0.539 |  | 157.66 |  | 1.000 |  |  |  | C31(Dehydro): 100; C37(Dehydro): 100 |  |  |  |  |  |  |  | 0.67 |  | 1 |  | Sequest HT (3) |  | 4 |  | 911.04138 |  | 1.77 |  | 41.2625 |  | 11663 |  | 11663 |  | TRINITY\_DN96\_c0\_g1\_i2.p1 |  | Image |  | Peak List |  |
|  | | | | | | | | | | | | | | | | | | | | | | | | | | | | | | | | | | | | | | | | | | | | | | | | | | | | | |  |
|  | High |  | [R].GIFAILKIATKLIGKTLAKAAGKAG.[TA] |  | HCD |  |  |  | 5.56 |  | 1.3e-3 |  | 2.8e-3 |  | 0.969 |  |  |  |  |  |  |  |  |  |  |  |  |  |  |  | 0.73 |  | 1 |  | Sequest HT (3) |  | 4 |  | 614.39532 |  | 2.47 |  | 41.8308 |  | 11922 |  | 11922 |  | TRINITY\_DN96\_c0\_g1\_i2.p1; TRINITY\_DN96\_c0\_g1\_i1.p1 |  | Image |  | Peak List |  |
|  | | | | | | | | | | | | | | | | | | | | | | | | | | | | | | | | | | | | | | | | | | | | | | | | | | | | | |  |
|  | **TRINITY\_DN0\_c1\_g1\_i20.p1 - TRINITY\_DN0\_c1\_g1\_i20.p1** | | | | | | | | | | | | | | | | | | | | | | | | | | | | | | | | | | | | | | | | | | | | | | | | | | | |  |  |
|  | | | | | | | | | | | | | | | | | | | | | | | | | | | | | | | | | | | | | | | | | | | | | | | | | | | | | |  |
|  | High |  | [R].AVNIPFKVHFRcKAAFc.[-] |  | HCD |  | C12(Dehydro); C17(Dehydro) |  | 2.52 |  | 3.9e-3 |  | 1.7e-1 |  | 0.056 |  | 153.03 |  | 1.000 |  |  |  | C12(Dehydro): 100; C17(Dehydro): 100 |  |  |  |  |  |  |  | 0.49 |  | 1 |  | Sequest HT (3) |  | 5 |  | 390.60641 |  | -0.36 |  | 27.3634 |  | 5594 |  | 5594 |  | TRINITY\_DN603\_c0\_g1\_i1.p1\_ORF1; TRINITY\_DN603\_c0\_g1\_i3.p1\_ORF2; TRINITY\_DN0\_c1\_g1\_i20.p1; TRINITY\_DN603\_c0\_g1\_i1.p1\_ORF3; TRINITY\_DN603\_c0\_g1\_i1.p1\_ORF2 |  | Image |  | Peak List |  |
|  | | | | | | | | | | | | | | | | | | | | | | | | | | | | | | | | | | | | | | | | | | | | | | | | | | | | | |  |
|  | High |  | [R].AVNIPFKVHFRcKAAFc.[-] |  | HCD |  | C12(Dehydro); C17(Dehydro) |  | 2.68 |  | 1.3e-3 |  | 3.5e-2 |  | 0.414 |  | 239.40 |  | 1.000 |  |  |  | C12(Dehydro): 100; C17(Dehydro): 100 |  |  |  |  |  |  |  | 0.45 |  | 1 |  | Sequest HT (3) |  | 3 |  | 650.33911 |  | -0.46 |  | 27.3896 |  | 5604 |  | 5604 |  | TRINITY\_DN603\_c0\_g1\_i1.p1\_ORF1; TRINITY\_DN603\_c0\_g1\_i3.p1\_ORF2; TRINITY\_DN0\_c1\_g1\_i20.p1; TRINITY\_DN603\_c0\_g1\_i1.p1\_ORF3; TRINITY\_DN603\_c0\_g1\_i1.p1\_ORF2 |  | Image |  | Peak List |  |
|  | | | | | | | | | | | | | | | | | | | | | | | | | | | | | | | | | | | | | | | | | | | | | | | | | | | | | |  |
|  | High |  | [R].AVNIPFKVHFRcKAAFc.[-] |  | HCD |  | C12(Dehydro); C17(Dehydro) |  | 1.78 |  | 3.1e-3 |  | 9.0e-2 |  | 0.206 |  | 139.38 |  | 1.000 |  |  |  | C12(Dehydro): 100; C17(Dehydro): 100 |  |  |  |  |  |  |  | 0.33 |  | 1 |  | Sequest HT (3) |  | 4 |  | 488.00647 |  | 0.19 |  | 27.4973 |  | 5643 |  | 5643 |  | TRINITY\_DN603\_c0\_g1\_i1.p1\_ORF1; TRINITY\_DN603\_c0\_g1\_i3.p1\_ORF2; TRINITY\_DN0\_c1\_g1\_i20.p1; TRINITY\_DN603\_c0\_g1\_i1.p1\_ORF3; TRINITY\_DN603\_c0\_g1\_i1.p1\_ORF2 |  | Image |  | Peak List |  |
|  | | | | | | | | | | | | | | | | | | | | | | | | | | | | | | | | | | | | | | | | | | | | | | | | | | | | | |  |
|  | High |  | [R].AVnIPFKVHFRcKAAFc.[-] |  | HCD |  | N3(Deamidated); C12(Dehydro); C17(Dehydro) |  | 2.42 |  | 2.4e-3 |  | 7.0e-2 |  | 0.261 |  | 170.85 |  | 1.000 |  |  |  | N3(Deamidated): 100; C12(Dehydro): 100; C17(Dehydro): 100 |  |  |  |  |  |  |  | 0.42 |  | 1 |  | Sequest HT (3) |  | 4 |  | 488.25211 |  | -0.56 |  | 28.2275 |  | 5898 |  | 5898 |  | TRINITY\_DN603\_c0\_g1\_i1.p1\_ORF1; TRINITY\_DN603\_c0\_g1\_i3.p1\_ORF2; TRINITY\_DN0\_c1\_g1\_i20.p1; TRINITY\_DN603\_c0\_g1\_i1.p1\_ORF3; TRINITY\_DN603\_c0\_g1\_i1.p1\_ORF2 |  | Image |  | Peak List |  |
|  | | | | | | | | | | | | | | | | | | | | | | | | | | | | | | | | | | | | | | | | | | | | | | | | | | | | | |  |
|  | High |  | [R].AVnIPFKVHFRcKAAFc.[-] |  | HCD |  | N3(Deamidated); C12(Dehydro); C17(Dehydro) |  | 1.46 |  | 3.9e-3 |  | 1.6e-1 |  | 0.076 |  | 137.36 |  | 1.000 |  |  |  | N3(Deamidated): 100; C12(Dehydro): 100; C17(Dehydro): 100 |  |  |  |  |  |  |  | 0.24 |  | 1 |  | Sequest HT (3) |  | 3 |  | 650.66748 |  | 0.10 |  | 28.2737 |  | 5915 |  | 5915 |  | TRINITY\_DN603\_c0\_g1\_i1.p1\_ORF1; TRINITY\_DN603\_c0\_g1\_i3.p1\_ORF2; TRINITY\_DN0\_c1\_g1\_i20.p1; TRINITY\_DN603\_c0\_g1\_i1.p1\_ORF3; TRINITY\_DN603\_c0\_g1\_i1.p1\_ORF2 |  | Image |  | Peak List |  |
|  | | | | | | | | | | | | | | | | | | | | | | | | | | | | | | | | | | | | | | | | | | | | | | | | | | | | | |  |
|  | **TRINITY\_DN49\_c0\_g1\_i1.p1\_ORF1 - TRINITY\_DN49\_c0\_g1\_i1.p1\_ORF1** | | | | | | | | | | | | | | | | | | | | | | | | | | | | | | | | | | | | | | | | | | | | | | | | | | | |  |  |
|  | | | | | | | | | | | | | | | | | | | | | | | | | | | | | | | | | | | | | | | | | | | | | | | | | | | | | |  |
|  | High |  | [R].GLFGKILGVGK.[K] |  | HCD |  |  |  | 1.93 |  | 3.1e-3 |  | 1.4e-1 |  | 0.113 |  |  |  |  |  |  |  |  |  |  |  |  |  |  |  | 0.45 |  | 1 |  | Sequest HT (3) |  | 2 |  | 544.84503 |  | 0.13 |  | 28.8754 |  | 6131 |  | 6131 |  | TRINITY\_DN49\_c0\_g1\_i1.p1\_ORF1 |  | Image |  | Peak List |  |
|  | | | | | | | | | | | | | | | | | | | | | | | | | | | | | | | | | | | | | | | | | | | | | | | | | | | | | |  |
|  | High |  | [R].GLFGKILGVGK.[K] |  | HCD |  |  |  | 2.48 |  | 1.3e-3 |  | 2.8e-2 |  | 0.466 |  |  |  |  |  |  |  |  |  |  |  |  |  |  |  | 0.53 |  | 1 |  | Sequest HT (3) |  | 3 |  | 363.56555 |  | -0.50 |  | 28.8902 |  | 6136 |  | 6136 |  | TRINITY\_DN49\_c0\_g1\_i1.p1\_ORF1 |  | Image |  | Peak List |  |
|  | | | | | | | | | | | | | | | | | | | | | | | | | | | | | | | | | | | | | | | | | | | | | | | | | | | | | |  |
|  | High |  | [R].GLFGKILGVGK.[K] |  | HCD |  |  |  | 1.65 |  | 1.3e-3 |  | 1.7e-2 |  | 0.576 |  |  |  |  |  |  |  |  |  |  |  |  |  |  |  | 0.45 |  | 1 |  | Sequest HT (3) |  | 2 |  | 544.84509 |  | 0.25 |  | 29.0144 |  | 6177 |  | 6177 |  | TRINITY\_DN49\_c0\_g1\_i1.p1\_ORF1 |  | Image |  | Peak List |  |
|  | | | | | | | | | | | | | | | | | | | | | | | | | | | | | | | | | | | | | | | | | | | | | | | | | | | | | |  |
|  | High |  | [R].GLFGKILGVGK.[K] |  | HCD |  |  |  | 1.71 |  | 3.1e-3 |  | 1.2e-1 |  | 0.134 |  |  |  |  |  |  |  |  |  |  |  |  |  |  |  | 0.42 |  | 1 |  | Sequest HT (3) |  | 2 |  | 544.84521 |  | 0.47 |  | 29.2027 |  | 6247 |  | 6247 |  | TRINITY\_DN49\_c0\_g1\_i1.p1\_ORF1 |  | Image |  | Peak List |  |
|  | | | | | | | | | | | | | | | | | | | | | | | | | | | | | | | | | | | | | | | | | | | | | | | | | | | | | |  |
|  | High |  | [R].GLFGKILGVGK.[K] |  | HCD |  |  |  | 2.64 |  | 1.3e-3 |  | 1.4e-2 |  | 0.623 |  |  |  |  |  |  |  |  |  |  |  |  |  |  |  | 0.54 |  | 1 |  | Sequest HT (3) |  | 3 |  | 363.56564 |  | -0.24 |  | 29.2701 |  | 6271 |  | 6271 |  | TRINITY\_DN49\_c0\_g1\_i1.p1\_ORF1 |  | Image |  | Peak List |  |
|  | | | | | | | | | | | | | | | | | | | | | | | | | | | | | | | | | | | | | | | | | | | | | | | | | | | | | |  |
|  | High |  | [R].GLFGKILGVGK.[K] |  | HCD |  |  |  | 1.78 |  | 5.6e-3 |  | 2.0e-1 |  | 0.02 |  |  |  |  |  |  |  |  |  |  |  |  |  |  |  | 0.43 |  | 1 |  | Sequest HT (3) |  | 2 |  | 544.84521 |  | 0.47 |  | 29.3666 |  | 6306 |  | 6306 |  | TRINITY\_DN49\_c0\_g1\_i1.p1\_ORF1 |  | Image |  | Peak List |  |
|  | | | | | | | | | | | | | | | | | | | | | | | | | | | | | | | | | | | | | | | | | | | | | | | | | | | | | |  |
|  | High |  | [R].GLFGKILGVGKKVL.[C] |  | HCD |  |  |  | 3.28 |  | 1.3e-3 |  | 5.0e-3 |  | 0.843 |  |  |  |  |  |  |  |  |  |  |  |  |  |  |  | 0.48 |  | 1 |  | Sequest HT (3) |  | 3 |  | 476.98169 |  | 0.30 |  | 31.2664 |  | 7038 |  | 7038 |  | TRINITY\_DN49\_c0\_g1\_i1.p1\_ORF1 |  | Image |  | Peak List |  |
|  | | | | | | | | | | | | | | | | | | | | | | | | | | | | | | | | | | | | | | | | | | | | | | | | | | | | | |  |
|  | High |  | [R].GLFGKILGVGKKVL.[C] |  | HCD |  |  |  | 2.41 |  | 1.3e-3 |  | 6.7e-2 |  | 0.274 |  |  |  |  |  |  |  |  |  |  |  |  |  |  |  | 0.46 |  | 1 |  | Sequest HT (3) |  | 3 |  | 476.98123 |  | -0.66 |  | 31.4117 |  | 7094 |  | 7094 |  | TRINITY\_DN49\_c0\_g1\_i1.p1\_ORF1 |  | Image |  | Peak List |  |
|  | | | | | | | | | | | | | | | | | | | | | | | | | | | | | | | | | | | | | | | | | | | | | | | | | | | | | |  |
|  | High |  | [R].GLFGKILGVGKKVL.[C] |  | HCD |  |  |  | 2.24 |  | 1.3e-3 |  | 2.0e-2 |  | 0.536 |  |  |  |  |  |  |  |  |  |  |  |  |  |  |  | 0.61 |  | 1 |  | Sequest HT (3) |  | 2 |  | 714.96753 |  | -1.61 |  | 31.4194 |  | 7098 |  | 7098 |  | TRINITY\_DN49\_c0\_g1\_i1.p1\_ORF1 |  | Image |  | Peak List |  |
|  | | | | | | | | | | | | | | | | | | | | | | | | | | | | | | | | | | | | | | | | | | | | | | | | | | | | | |  |
|  | High |  | [R].GLFGKILGVGKKVL.[C] |  | HCD |  |  |  | 3.52 |  | 1.3e-3 |  | 1.9e-2 |  | 0.556 |  |  |  |  |  |  |  |  |  |  |  |  |  |  |  | 0.61 |  | 1 |  | Sequest HT (3) |  | 4 |  | 357.98770 |  | -0.77 |  | 31.4714 |  | 7117 |  | 7117 |  | TRINITY\_DN49\_c0\_g1\_i1.p1\_ORF1 |  | Image |  | Peak List |  |
|  | | | | | | | | | | | | | | | | | | | | | | | | | | | | | | | | | | | | | | | | | | | | | | | | | | | | | |  |
|  | High |  | [R].GLFGKILGVGKKVL.[C] |  | HCD |  |  |  | 3.68 |  | 1.3e-3 |  | 9.5e-3 |  | 0.702 |  |  |  |  |  |  |  |  |  |  |  |  |  |  |  | 0.57 |  | 1 |  | Sequest HT (3) |  | 4 |  | 357.98755 |  | -1.20 |  | 31.6532 |  | 7187 |  | 7187 |  | TRINITY\_DN49\_c0\_g1\_i1.p1\_ORF1 |  | Image |  | Peak List |  |
|  | | | | | | | | | | | | | | | | | | | | | | | | | | | | | | | | | | | | | | | | | | | | | | | | | | | | | |  |
|  | High |  | [R].GLFGKILGVGKKVL.[C] |  | HCD |  |  |  | 3.08 |  | 1.3e-3 |  | 7.3e-4 |  | 1.26 |  |  |  |  |  |  |  |  |  |  |  |  |  |  |  | 0.69 |  | 1 |  | Sequest HT (3) |  | 2 |  | 714.96838 |  | -0.41 |  | 31.6781 |  | 7198 |  | 7198 |  | TRINITY\_DN49\_c0\_g1\_i1.p1\_ORF1 |  | Image |  | Peak List |  |
|  | | | | | | | | | | | | | | | | | | | | | | | | | | | | | | | | | | | | | | | | | | | | | | | | | | | | | |  |
|  | High |  | [G].KILGVGKKVLcGLSGMc.[-] |  | HCD |  | C11(Dehydro); C17(Dehydro) |  | 4.38 |  | 1.3e-3 |  | 4.5e-4 |  | 1.36 |  | 386.95 |  | 1.000 |  |  |  | C11(Dehydro): 100; C17(Dehydro): 100 |  |  |  |  |  |  |  | 0.58 |  | 1 |  | Sequest HT (3) |  | 2 |  | 852.47278 |  | 0.81 |  | 32.0237 |  | 7340 |  | 7340 |  | TRINITY\_DN49\_c0\_g1\_i1.p1\_ORF1 |  | Image |  | Peak List |  |
|  | | | | | | | | | | | | | | | | | | | | | | | | | | | | | | | | | | | | | | | | | | | | | | | | | | | | | |  |
|  | High |  | [G].KILGVGKKVLcGLSGMc.[-] |  | HCD |  | C11(Dehydro); C17(Dehydro) |  | 1.93 |  | 3.1e-3 |  | 1.3e-1 |  | 0.131 |  | 226.02 |  | 1.000 |  |  |  | C11(Dehydro): 100; C17(Dehydro): 100 |  |  |  |  |  |  |  | 0.51 |  | 1 |  | Sequest HT (3) |  | 2 |  | 852.47217 |  | 0.09 |  | 32.1735 |  | 7399 |  | 7399 |  | TRINITY\_DN49\_c0\_g1\_i1.p1\_ORF1 |  | Image |  | Peak List |  |
|  | | | | | | | | | | | | | | | | | | | | | | | | | | | | | | | | | | | | | | | | | | | | | | | | | | | | | |  |
|  | High |  | [F].GKILGVGKKVLcGLSGMc.[-] |  | HCD |  | C12(Dehydro); C18(Dehydro) |  | 3.04 |  | 1.3e-3 |  | 8.1e-3 |  | 0.736 |  | 269.64 |  | 1.000 |  |  |  | C12(Dehydro): 100; C18(Dehydro): 100 |  |  |  |  |  |  |  | 0.49 |  | 1 |  | Sequest HT (3) |  | 2 |  | 880.98193 |  | -1.01 |  | 33.5325 |  | 7978 |  | 7978 |  | TRINITY\_DN49\_c0\_g1\_i1.p1\_ORF1 |  | Image |  | Peak List |  |
|  | | | | | | | | | | | | | | | | | | | | | | | | | | | | | | | | | | | | | | | | | | | | | | | | | | | | | |  |
|  | High |  | [R].GLFGKILGVG.[K] |  | HCD |  |  |  | 1.72 |  | 1.3e-3 |  | 3.2e-2 |  | 0.436 |  |  |  |  |  |  |  |  |  |  |  |  |  |  |  | 0.35 |  | 1 |  | Sequest HT (3) |  | 2 |  | 480.79755 |  | 0.14 |  | 34.3030 |  | 8324 |  | 8324 |  | TRINITY\_DN49\_c0\_g1\_i1.p1\_ORF1 |  | Image |  | Peak List |  |
|  | | | | | | | | | | | | | | | | | | | | | | | | | | | | | | | | | | | | | | | | | | | | | | | | | | | | | |  |
|  | High |  | [R].GLFGKILGVGKKVLCGLSGmC.[-] |  | HCD |  | M20(Oxidation) |  | 2.89 |  | 1.3e-3 |  | 8.0e-3 |  | 0.739 |  | 220.37 |  | 1.000 |  |  |  | M20(Oxidation): 100 |  |  |  |  |  |  |  | 0.68 |  | 1 |  | Sequest HT (3) |  | 2 |  | 1048.57642 |  | 1.28 |  | 34.7919 |  | 8546 |  | 8546 |  | TRINITY\_DN49\_c0\_g1\_i1.p1\_ORF1 |  | Image |  | Peak List |  |
|  | | | | | | | | | | | | | | | | | | | | | | | | | | | | | | | | | | | | | | | | | | | | | | | | | | | | | |  |
|  | High |  | [R].GLFGKILGVGKKVLCGLSGmC.[-] |  | HCD |  | M20(Oxidation) |  | 3.46 |  | 3.1e-3 |  | 1.4e-1 |  | 0.107 |  | 168.92 |  | 1.000 |  |  |  | M20(Oxidation): 100 |  |  |  |  |  |  |  | 0.53 |  | 1 |  | Sequest HT (3) |  | 3 |  | 699.38605 |  | 0.34 |  | 34.8963 |  | 8592 |  | 8592 |  | TRINITY\_DN49\_c0\_g1\_i1.p1\_ORF1 |  | Image |  | Peak List |  |
|  | | | | | | | | | | | | | | | | | | | | | | | | | | | | | | | | | | | | | | | | | | | | | | | | | | | | | |  |
|  | High |  | [R].GLFGKILGVGKKVLCGLSGmC.[-] |  | HCD |  | M20(Oxidation) |  | 3.16 |  | 1.3e-3 |  | 5.7e-3 |  | 0.812 |  | 242.93 |  | 1.000 |  |  |  | M20(Oxidation): 100 |  |  |  |  |  |  |  | 0.60 |  | 1 |  | Sequest HT (3) |  | 2 |  | 1048.57446 |  | -0.58 |  | 34.9877 |  | 8629 |  | 8629 |  | TRINITY\_DN49\_c0\_g1\_i1.p1\_ORF1 |  | Image |  | Peak List |  |
|  | | | | | | | | | | | | | | | | | | | | | | | | | | | | | | | | | | | | | | | | | | | | | | | | | | | | | |  |
|  | High |  | [R].GLFGKILGVGKKVLCGLSGmC.[-] |  | HCD |  | M20(Oxidation) |  | 4.55 |  | 1.3e-3 |  | 9.1e-4 |  | 1.21 |  | 323.77 |  | 1.000 |  |  |  | M20(Oxidation): 100 |  |  |  |  |  |  |  | 0.51 |  | 1 |  | Sequest HT (3) |  | 2 |  | 1048.57324 |  | -1.75 |  | 36.3775 |  | 9294 |  | 9294 |  | TRINITY\_DN49\_c0\_g1\_i1.p1\_ORF1 |  | Image |  | Peak List |  |
|  | | | | | | | | | | | | | | | | | | | | | | | | | | | | | | | | | | | | | | | | | | | | | | | | | | | | | |  |
|  | High |  | [R].GLFGKILGVGKKVLCGLSGmC.[-] |  | HCD |  | M20(Oxidation) |  | 2.63 |  | 1.3e-3 |  | 9.1e-3 |  | 0.713 |  | 222.62 |  | 1.000 |  |  |  | M20(Oxidation): 100 |  |  |  |  |  |  |  | 0.49 |  | 1 |  | Sequest HT (3) |  | 2 |  | 1048.57422 |  | -0.82 |  | 36.5195 |  | 9364 |  | 9364 |  | TRINITY\_DN49\_c0\_g1\_i1.p1\_ORF1 |  | Image |  | Peak List |  |
|  | | | | | | | | | | | | | | | | | | | | | | | | | | | | | | | | | | | | | | | | | | | | | | | | | | | | | |  |
|  | High |  | [R].GLFGKILGVGKKVLCGLSGmC.[-] |  | HCD |  | M20(Oxidation) |  | 2.61 |  | 4.7e-3 |  | 2.0e-1 |  | 0.022 |  | 171.10 |  | 1.000 |  |  |  | M20(Oxidation): 100 |  |  |  |  |  |  |  | 0.42 |  | 1 |  | Sequest HT (3) |  | 3 |  | 699.38531 |  | -0.71 |  | 36.5966 |  | 9403 |  | 9403 |  | TRINITY\_DN49\_c0\_g1\_i1.p1\_ORF1 |  | Image |  | Peak List |  |
|  | | | | | | | | | | | | | | | | | | | | | | | | | | | | | | | | | | | | | | | | | | | | | | | | | | | | | |  |
|  | High |  | [R].GLFGKILGVGKKVLCGLSGmC.[-] |  | HCD |  | M20(Oxidation) |  | 4.17 |  | 1.3e-3 |  | 5.3e-4 |  | 1.33 |  | 283.56 |  | 1.000 |  |  |  | M20(Oxidation): 100 |  |  |  |  |  |  |  | 0.61 |  | 1 |  | Sequest HT (3) |  | 2 |  | 1048.57458 |  | -0.47 |  | 36.6684 |  | 9436 |  | 9436 |  | TRINITY\_DN49\_c0\_g1\_i1.p1\_ORF1 |  | Image |  | Peak List |  |
|  | | | | | | | | | | | | | | | | | | | | | | | | | | | | | | | | | | | | | | | | | | | | | | | | | | | | | |  |
|  | High |  | [G].LFGKILGVGKKVLcGLSGmc.[-] |  | HCD |  | C14(Dehydro); M19(Oxidation); C20(Dehydro) |  | 3.49 |  | 1.3e-3 |  | 2.8e-2 |  | 0.462 |  | 251.28 |  | 1.000 |  |  |  | C14(Dehydro): 100; M19(Oxidation): 100; C20(Dehydro): 100 |  |  |  |  |  |  |  | 0.47 |  | 1 |  | Sequest HT (3) |  | 3 |  | 679.70526 |  | -2.22 |  | 37.3268 |  | 9754 |  | 9754 |  | TRINITY\_DN49\_c0\_g1\_i1.p1\_ORF1 |  | Image |  | Peak List |  |
|  | | | | | | | | | | | | | | | | | | | | | | | | | | | | | | | | | | | | | | | | | | | | | | | | | | | | | |  |
|  | High |  | [G].LFGKILGVGKKVLcGLSGmc.[-] |  | HCD |  | C14(Dehydro); M19(Oxidation); C20(Dehydro) |  | 3.55 |  | 1.3e-3 |  | 1.2e-3 |  | 1.15 |  | 474.02 |  | 1.000 |  |  |  | C14(Dehydro): 100; M19(Oxidation): 100; C20(Dehydro): 100 |  |  |  |  |  |  |  | 0.62 |  | 1 |  | Sequest HT (3) |  | 2 |  | 1019.05902 |  | 2.46 |  | 37.3386 |  | 9760 |  | 9760 |  | TRINITY\_DN49\_c0\_g1\_i1.p1\_ORF1 |  | Image |  | Peak List |  |
|  | | | | | | | | | | | | | | | | | | | | | | | | | | | | | | | | | | | | | | | | | | | | | | | | | | | | | |  |
|  | High |  | [G].LFGKILGVGKKVLcGLSGmc.[-] |  | HCD |  | C14(Dehydro); M19(Oxidation); C20(Dehydro) |  | 3.55 |  | 1.3e-3 |  | 8.1e-3 |  | 0.738 |  | 391.80 |  | 1.000 |  |  |  | C14(Dehydro): 100; M19(Oxidation): 100; C20(Dehydro): 100 |  |  |  |  |  |  |  | 0.59 |  | 1 |  | Sequest HT (3) |  | 2 |  | 1019.05469 |  | -1.80 |  | 37.5502 |  | 9872 |  | 9872 |  | TRINITY\_DN49\_c0\_g1\_i1.p1\_ORF1 |  | Image |  | Peak List |  |
|  | | | | | | | | | | | | | | | | | | | | | | | | | | | | | | | | | | | | | | | | | | | | | | | | | | | | | |  |
|  | High |  | [G].LFGKILGVGKKVLcGLSGmc.[-] |  | HCD |  | C14(Dehydro); M19(Oxidation); C20(Dehydro) |  | 2.72 |  | 1.3e-3 |  | 5.7e-2 |  | 0.31 |  | 298.71 |  | 1.000 |  |  |  | C14(Dehydro): 100; M19(Oxidation): 100; C20(Dehydro): 100 |  |  |  |  |  |  |  | 0.29 |  | 1 |  | Sequest HT (3) |  | 2 |  | 1019.05609 |  | -0.42 |  | 38.0150 |  | 10085 |  | 10085 |  | TRINITY\_DN49\_c0\_g1\_i1.p1\_ORF1 |  | Image |  | Peak List |  |
|  | | | | | | | | | | | | | | | | | | | | | | | | | | | | | | | | | | | | | | | | | | | | | | | | | | | | | |  |
|  | High |  | [R].GLFGKILGVGKKVLCGLSGmC.[-] |  | HCD |  | M20(Oxidation) |  | 2.40 |  | 3.1e-3 |  | 8.8e-2 |  | 0.212 |  | 143.79 |  | 1.000 |  |  |  | M20(Oxidation): 100 |  |  |  |  |  |  |  | 0.26 |  | 1 |  | Sequest HT (3) |  | 4 |  | 524.79150 |  | 0.63 |  | 38.1116 |  | 10139 |  | 10139 |  | TRINITY\_DN49\_c0\_g1\_i1.p1\_ORF1 |  | Image |  | Peak List |  |
|  | | | | | | | | | | | | | | | | | | | | | | | | | | | | | | | | | | | | | | | | | | | | | | | | | | | | | |  |
|  | High |  | [R].GLFGKILGVGKKVLCGLSGmC.[-] |  | HCD |  | M20(Oxidation) |  | 3.84 |  | 1.3e-3 |  | 2.2e-3 |  | 1.02 |  | 280.71 |  | 1.000 |  |  |  | M20(Oxidation): 100 |  |  |  |  |  |  |  | 0.46 |  | 1 |  | Sequest HT (3) |  | 2 |  | 1048.57300 |  | -1.98 |  | 38.1437 |  | 10155 |  | 10155 |  | TRINITY\_DN49\_c0\_g1\_i1.p1\_ORF1 |  | Image |  | Peak List |  |
|  | | | | | | | | | | | | | | | | | | | | | | | | | | | | | | | | | | | | | | | | | | | | | | | | | | | | | |  |
|  | High |  | [R].GLFGKILGVGKKVLCGLSGmC.[-] |  | HCD |  | M20(Oxidation) |  | 2.66 |  | 1.3e-3 |  | 1.7e-2 |  | 0.569 |  | 231.52 |  | 1.000 |  |  |  | M20(Oxidation): 100 |  |  |  |  |  |  |  | 0.34 |  | 1 |  | Sequest HT (3) |  | 2 |  | 1048.57300 |  | -1.98 |  | 38.3107 |  | 10237 |  | 10237 |  | TRINITY\_DN49\_c0\_g1\_i1.p1\_ORF1 |  | Image |  | Peak List |  |
|  | | | | | | | | | | | | | | | | | | | | | | | | | | | | | | | | | | | | | | | | | | | | | | | | | | | | | |  |
|  | High |  | [R].GLFGKILGVGKKVLCGLSGmC.[-] |  | HCD |  | M20(Oxidation) |  | 3.25 |  | 1.3e-3 |  | 5.3e-3 |  | 0.829 |  | 245.02 |  | 1.000 |  |  |  | M20(Oxidation): 100 |  |  |  |  |  |  |  | 0.49 |  | 1 |  | Sequest HT (3) |  | 2 |  | 1048.57458 |  | -0.47 |  | 38.4580 |  | 10314 |  | 10314 |  | TRINITY\_DN49\_c0\_g1\_i1.p1\_ORF1 |  | Image |  | Peak List |  |
|  | | | | | | | | | | | | | | | | | | | | | | | | | | | | | | | | | | | | | | | | | | | | | | | | | | | | | |  |
|  | High |  | [R].GLFGKILGVGKKVLCGLSGmC.[-] |  | HCD |  | M20(Oxidation) |  | 3.59 |  | 1.3e-3 |  | 2.3e-4 |  | 1.52 |  | 309.57 |  | 1.000 |  |  |  | M20(Oxidation): 100 |  |  |  |  |  |  |  | 0.52 |  | 1 |  | Sequest HT (3) |  | 2 |  | 1048.57446 |  | -0.58 |  | 38.8428 |  | 10503 |  | 10503 |  | TRINITY\_DN49\_c0\_g1\_i1.p1\_ORF1 |  | Image |  | Peak List |  |
|  | | | | | | | | | | | | | | | | | | | | | | | | | | | | | | | | | | | | | | | | | | | | | | | | | | | | | |  |
|  | High |  | [R].GLFGKILGVGKKVLCGLSGmC.[-] |  | HCD |  | M20(Oxidation) |  | 3.71 |  | 1.3e-3 |  | 4.5e-4 |  | 1.37 |  | 276.08 |  | 1.000 |  |  |  | M20(Oxidation): 100 |  |  |  |  |  |  |  | 0.62 |  | 1 |  | Sequest HT (3) |  | 2 |  | 1048.57544 |  | 0.35 |  | 38.9854 |  | 10568 |  | 10568 |  | TRINITY\_DN49\_c0\_g1\_i1.p1\_ORF1 |  | Image |  | Peak List |  |
|  | | | | | | | | | | | | | | | | | | | | | | | | | | | | | | | | | | | | | | | | | | | | | | | | | | | | | |  |
|  | High |  | [R].GLFGKILGVGKKVLCGLSGmC.[-] |  | HCD |  | M20(Oxidation) |  | 3.71 |  | 1.3e-3 |  | 6.1e-3 |  | 0.798 |  | 281.30 |  | 1.000 |  |  |  | M20(Oxidation): 100 |  |  |  |  |  |  |  | 0.45 |  | 1 |  | Sequest HT (3) |  | 2 |  | 1048.57373 |  | -1.28 |  | 39.2002 |  | 10679 |  | 10679 |  | TRINITY\_DN49\_c0\_g1\_i1.p1\_ORF1 |  | Image |  | Peak List |  |
|  | | | | | | | | | | | | | | | | | | | | | | | | | | | | | | | | | | | | | | | | | | | | | | | | | | | | | |  |
|  | High |  | [R].GLFGKILGVGKKVLCGLSGmC.[-] |  | HCD |  | M20(Oxidation) |  | 3.61 |  | 1.3e-3 |  | 3.4e-3 |  | 0.928 |  | 295.41 |  | 1.000 |  |  |  | M20(Oxidation): 100 |  |  |  |  |  |  |  | 0.66 |  | 1 |  | Sequest HT (3) |  | 2 |  | 1048.57629 |  | 1.16 |  | 39.4092 |  | 10778 |  | 10778 |  | TRINITY\_DN49\_c0\_g1\_i1.p1\_ORF1 |  | Image |  | Peak List |  |
|  | | | | | | | | | | | | | | | | | | | | | | | | | | | | | | | | | | | | | | | | | | | | | | | | | | | | | |  |
|  | High |  | [R].GLFGKILGVGKKVLCGLSGmC.[-] |  | HCD |  | M20(Oxidation) |  | 2.48 |  | 3.9e-3 |  | 1.7e-1 |  | 0.061 |  | 136.56 |  | 1.000 |  |  |  | M20(Oxidation): 100 |  |  |  |  |  |  |  | 0.06 |  | 1 |  | Sequest HT (3) |  | 3 |  | 699.38428 |  | -2.19 |  | 39.9271 |  | 11043 |  | 11043 |  | TRINITY\_DN49\_c0\_g1\_i1.p1\_ORF1 |  | Image |  | Peak List |  |
|  | | | | | | | | | | | | | | | | | | | | | | | | | | | | | | | | | | | | | | | | | | | | | | | | | | | | | |  |
|  | High |  | [R].GLFGKILGVGKKVLCGLSGmC.[-] |  | HCD |  | M20(Oxidation) |  | 3.70 |  | 1.3e-3 |  | 1.4e-3 |  | 1.11 |  | 325.62 |  | 1.000 |  |  |  | M20(Oxidation): 100 |  |  |  |  |  |  |  | 0.59 |  | 1 |  | Sequest HT (3) |  | 2 |  | 1048.57678 |  | 1.63 |  | 39.9871 |  | 11075 |  | 11075 |  | TRINITY\_DN49\_c0\_g1\_i1.p1\_ORF1 |  | Image |  | Peak List |  |
|  | | | | | | | | | | | | | | | | | | | | | | | | | | | | | | | | | | | | | | | | | | | | | | | | | | | | | |  |
|  | High |  | [R].GLFGKILGVGKKVLCGLSGmC.[-] |  | HCD |  | M20(Oxidation) |  | 2.27 |  | 3.1e-3 |  | 1.2e-1 |  | 0.132 |  | 180.28 |  | 1.000 |  |  |  | M20(Oxidation): 100 |  |  |  |  |  |  |  | 0.49 |  | 1 |  | Sequest HT (3) |  | 3 |  | 699.38568 |  | -0.18 |  | 40.0724 |  | 11119 |  | 11119 |  | TRINITY\_DN49\_c0\_g1\_i1.p1\_ORF1 |  | Image |  | Peak List |  |
|  | | | | | | | | | | | | | | | | | | | | | | | | | | | | | | | | | | | | | | | | | | | | | | | | | | | | | |  |
|  | High |  | [G].LFGKILGVGKKVLCGLSGMc.[-] |  | HCD |  | C20(Dehydro) |  | 3.94 |  | 1.3e-3 |  | 2.3e-3 |  | 1.01 |  | 345.94 |  | 0.500 |  |  |  | C14(Dehydro): 50; C20(Dehydro): 50 |  |  |  |  |  |  |  | 0.48 |  | 1 |  | Sequest HT (3) |  | 2 |  | 1011.56012 |  | -2.82 |  | 40.1837 |  | 11175 |  | 11175 |  | TRINITY\_DN49\_c0\_g1\_i1.p1\_ORF1 |  | Image |  | Peak List |  |
|  | | | | | | | | | | | | | | | | | | | | | | | | | | | | | | | | | | | | | | | | | | | | | | | | | | | | | |  |
|  | High |  | [G].LFGKILGVGKKVLcGLSGMC.[-] |  | HCD |  | C14(Dehydro) |  | 3.94 |  | 1.3e-3 |  | 2.3e-3 |  | 1.01 |  | 345.94 |  | 0.500 |  |  |  | C14(Dehydro): 50; C20(Dehydro): 50 |  |  |  |  |  |  |  | 0.48 |  | 1 |  | Sequest HT (3) |  | 2 |  | 1011.56012 |  | -2.82 |  | 40.1837 |  | 11175 |  | 11175 |  | TRINITY\_DN49\_c0\_g1\_i1.p1\_ORF1 |  | Image |  | Peak List |  |
|  | | | | | | | | | | | | | | | | | | | | | | | | | | | | | | | | | | | | | | | | | | | | | | | | | | | | | |  |
|  | High |  | [G].LFGKILGVGKKVLcGLSGMc.[-] |  | HCD |  | C14(Dehydro); C20(Dehydro) |  | 2.79 |  | 3.1e-3 |  | 1.3e-1 |  | 0.117 |  | 200.78 |  | 1.000 |  |  |  | C14(Dehydro): 100; C20(Dehydro): 100 |  |  |  |  |  |  |  | 0.29 |  | 1 |  | Sequest HT (3) |  | 3 |  | 674.37500 |  | -0.20 |  | 40.3144 |  | 11237 |  | 11237 |  | TRINITY\_DN49\_c0\_g1\_i1.p1\_ORF1 |  | Image |  | Peak List |  |
|  | | | | | | | | | | | | | | | | | | | | | | | | | | | | | | | | | | | | | | | | | | | | | | | | | | | | | |  |
|  | High |  | [G].LFGKILGVGKKVLcGLSGMc.[-] |  | HCD |  | C14(Dehydro); C20(Dehydro) |  | 4.03 |  | 1.3e-3 |  | 9.0e-4 |  | 1.22 |  | 406.15 |  | 1.000 |  |  |  | C14(Dehydro): 100; C20(Dehydro): 100 |  |  |  |  |  |  |  | 0.51 |  | 1 |  | Sequest HT (3) |  | 2 |  | 1011.05750 |  | -1.55 |  | 40.3374 |  | 11247 |  | 11247 |  | TRINITY\_DN49\_c0\_g1\_i1.p1\_ORF1 |  | Image |  | Peak List |  |
|  | | | | | | | | | | | | | | | | | | | | | | | | | | | | | | | | | | | | | | | | | | | | | | | | | | | | | |  |
|  | High |  | [G].LFGKILGVGKKVLcGLSGMc.[-] |  | HCD |  | C14(Dehydro); C20(Dehydro) |  | 3.16 |  | 2.4e-3 |  | 8.0e-2 |  | 0.232 |  | 213.21 |  | 1.000 |  |  |  | C14(Dehydro): 100; C20(Dehydro): 100 |  |  |  |  |  |  |  | 0.41 |  | 1 |  | Sequest HT (3) |  | 3 |  | 674.37561 |  | 0.71 |  | 40.7344 |  | 11428 |  | 11428 |  | TRINITY\_DN49\_c0\_g1\_i1.p1\_ORF1 |  | Image |  | Peak List |  |
|  | | | | | | | | | | | | | | | | | | | | | | | | | | | | | | | | | | | | | | | | | | | | | | | | | | | | | |  |
|  | High |  | [G].LFGKILGVGKKVLcGLSGMc.[-] |  | HCD |  | C14(Dehydro); C20(Dehydro) |  | 3.92 |  | 1.3e-3 |  | 3.1e-3 |  | 0.949 |  | 412.42 |  | 1.000 |  |  |  | C14(Dehydro): 100; C20(Dehydro): 100 |  |  |  |  |  |  |  | 0.44 |  | 1 |  | Sequest HT (3) |  | 2 |  | 1011.05743 |  | -1.61 |  | 40.7356 |  | 11429 |  | 11429 |  | TRINITY\_DN49\_c0\_g1\_i1.p1\_ORF1 |  | Image |  | Peak List |  |
|  | | | | | | | | | | | | | | | | | | | | | | | | | | | | | | | | | | | | | | | | | | | | | | | | | | | | | |  |
|  | High |  | [G].LFGKILGVGKKVLcGLSGMc.[-] |  | HCD |  | C14(Dehydro); C20(Dehydro) |  | 3.48 |  | 1.3e-3 |  | 1.3e-2 |  | 0.634 |  | 278.99 |  | 1.000 |  |  |  | C14(Dehydro): 100; C20(Dehydro): 100 |  |  |  |  |  |  |  | 0.34 |  | 1 |  | Sequest HT (3) |  | 2 |  | 1011.05829 |  | -0.76 |  | 40.8777 |  | 11494 |  | 11494 |  | TRINITY\_DN49\_c0\_g1\_i1.p1\_ORF1 |  | Image |  | Peak List |  |
|  | | | | | | | | | | | | | | | | | | | | | | | | | | | | | | | | | | | | | | | | | | | | | | | | | | | | | |  |
|  | High |  | [R].GLFGKILGVGKKVLcGLSGmc.[-] |  | HCD |  | C15(Dehydro); M20(Oxidation); C21(Dehydro) |  | 3.18 |  | 1.3e-3 |  | 3.0e-2 |  | 0.45 |  | 348.06 |  | 1.000 |  |  |  | C15(Dehydro): 100; M20(Oxidation): 100; C21(Dehydro): 100 |  |  |  |  |  |  |  | 0.54 |  | 1 |  | Sequest HT (3) |  | 3 |  | 698.71344 |  | -0.69 |  | 41.4069 |  | 11723 |  | 11723 |  | TRINITY\_DN49\_c0\_g1\_i1.p1\_ORF1 |  | Image |  | Peak List |  |
|  | | | | | | | | | | | | | | | | | | | | | | | | | | | | | | | | | | | | | | | | | | | | | | | | | | | | | |  |
|  | High |  | [R].GLFGKILGVGKKVLcGLSGmc.[-] |  | HCD |  | C15(Dehydro); M20(Oxidation); C21(Dehydro) |  | 3.31 |  | 2.4e-3 |  | 7.8e-2 |  | 0.238 |  | 315.19 |  | 1.000 |  |  |  | C15(Dehydro): 100; M20(Oxidation): 100; C21(Dehydro): 100 |  |  |  |  |  |  |  | 0.46 |  | 1 |  | Sequest HT (3) |  | 2 |  | 1047.56616 |  | -1.04 |  | 41.4345 |  | 11734 |  | 11734 |  | TRINITY\_DN49\_c0\_g1\_i1.p1\_ORF1 |  | Image |  | Peak List |  |
|  | | | | | | | | | | | | | | | | | | | | | | | | | | | | | | | | | | | | | | | | | | | | | | | | | | | | | |  |
|  | High |  | [R].GLFGKILGVGKKVLcGLSGmc.[-] |  | HCD |  | C15(Dehydro); M20(Oxidation); C21(Dehydro) |  | 3.46 |  | 1.3e-3 |  | 8.8e-3 |  | 0.719 |  | 404.81 |  | 1.000 |  |  |  | C15(Dehydro): 100; M20(Oxidation): 100; C21(Dehydro): 100 |  |  |  |  |  |  |  | 0.51 |  | 1 |  | Sequest HT (3) |  | 2 |  | 1047.56604 |  | -1.15 |  | 41.5760 |  | 11804 |  | 11804 |  | TRINITY\_DN49\_c0\_g1\_i1.p1\_ORF1 |  | Image |  | Peak List |  |
|  | | | | | | | | | | | | | | | | | | | | | | | | | | | | | | | | | | | | | | | | | | | | | | | | | | | | | |  |
|  | High |  | [R].GLFGKILGVGKKVLcGLSGmc.[-] |  | HCD |  | C15(Dehydro); M20(Oxidation); C21(Dehydro) |  | 3.93 |  | 1.3e-3 |  | 4.9e-3 |  | 0.848 |  | 432.59 |  | 1.000 |  |  |  | C15(Dehydro): 100; M20(Oxidation): 100; C21(Dehydro): 100 |  |  |  |  |  |  |  | 0.55 |  | 1 |  | Sequest HT (3) |  | 2 |  | 1047.56592 |  | -1.27 |  | 41.7215 |  | 11875 |  | 11875 |  | TRINITY\_DN49\_c0\_g1\_i1.p1\_ORF1 |  | Image |  | Peak List |  |
|  | | | | | | | | | | | | | | | | | | | | | | | | | | | | | | | | | | | | | | | | | | | | | | | | | | | | | |  |
|  | High |  | [R].GLFGKILGVGKKVLcGLSGmc.[-] |  | HCD |  | C15(Dehydro); M20(Oxidation); C21(Dehydro) |  | 2.49 |  | 3.9e-3 |  | 1.6e-1 |  | 0.072 |  | 237.61 |  | 1.000 |  |  |  | C15(Dehydro): 100; M20(Oxidation): 100; C21(Dehydro): 100 |  |  |  |  |  |  |  | 0.49 |  | 1 |  | Sequest HT (3) |  | 3 |  | 698.71375 |  | -0.26 |  | 41.8372 |  | 11924 |  | 11924 |  | TRINITY\_DN49\_c0\_g1\_i1.p1\_ORF1 |  | Image |  | Peak List |  |
|  | | | | | | | | | | | | | | | | | | | | | | | | | | | | | | | | | | | | | | | | | | | | | | | | | | | | | |  |
|  | High |  | [R].GLFGKILGVGKKVLcGLSGmc.[-] |  | HCD |  | C15(Dehydro); M20(Oxidation); C21(Dehydro) |  | 3.24 |  | 1.3e-3 |  | 9.4e-3 |  | 0.705 |  | 374.74 |  | 1.000 |  |  |  | C15(Dehydro): 100; M20(Oxidation): 100; C21(Dehydro): 100 |  |  |  |  |  |  |  | 0.56 |  | 1 |  | Sequest HT (3) |  | 2 |  | 1047.56665 |  | -0.57 |  | 41.9575 |  | 11979 |  | 11979 |  | TRINITY\_DN49\_c0\_g1\_i1.p1\_ORF1 |  | Image |  | Peak List |  |
|  | | | | | | | | | | | | | | | | | | | | | | | | | | | | | | | | | | | | | | | | | | | | | | | | | | | | | |  |
|  | High |  | [R].GLFGKILGVGKKVLcGLSGMc.[-] |  | HCD |  | C15(Dehydro); C21(Dehydro) |  | 4.94 |  | 1.3e-3 |  | 4.4e-4 |  | 1.37 |  | 437.43 |  | 1.000 |  |  |  | C15(Dehydro): 100; C21(Dehydro): 100 |  |  |  |  |  |  |  | 0.56 |  | 1 |  | Sequest HT (3) |  | 2 |  | 1039.56934 |  | -0.44 |  | 45.2887 |  | 13364 |  | 13364 |  | TRINITY\_DN49\_c0\_g1\_i1.p1\_ORF1 |  | Image |  | Peak List |  |
|  | | | | | | | | | | | | | | | | | | | | | | | | | | | | | | | | | | | | | | | | | | | | | | | | | | | | | |  |
|  | High |  | [R].GLFGKILGVGKKVLcGLSGMc.[-] |  | HCD |  | C15(Dehydro); C21(Dehydro) |  | 3.46 |  | 3.1e-3 |  | 1.1e-1 |  | 0.151 |  | 236.43 |  | 1.000 |  |  |  | C15(Dehydro): 100; C21(Dehydro): 100 |  |  |  |  |  |  |  | 0.49 |  | 1 |  | Sequest HT (3) |  | 3 |  | 693.38110 |  | -1.71 |  | 45.3123 |  | 13376 |  | 13376 |  | TRINITY\_DN49\_c0\_g1\_i1.p1\_ORF1 |  | Image |  | Peak List |  |
|  | | | | | | | | | | | | | | | | | | | | | | | | | | | | | | | | | | | | | | | | | | | | | | | | | | | | | |  |
|  | High |  | [R].GLFGKILGVGKKVLcGLSGMc.[-] |  | HCD |  | C15(Dehydro); C21(Dehydro) |  | 4.85 |  | 1.3e-3 |  | 1.6e-4 |  | 1.59 |  | 411.64 |  | 1.000 |  |  |  | C15(Dehydro): 100; C21(Dehydro): 100 |  |  |  |  |  |  |  | 0.55 |  | 1 |  | Sequest HT (3) |  | 2 |  | 1039.56921 |  | -0.56 |  | 45.4359 |  | 13425 |  | 13425 |  | TRINITY\_DN49\_c0\_g1\_i1.p1\_ORF1 |  | Image |  | Peak List |  |
|  | | | | | | | | | | | | | | | | | | | | | | | | | | | | | | | | | | | | | | | | | | | | | | | | | | | | | |  |
|  | High |  | [R].GLFGKILGVGKKVLcGLSGMc.[-] |  | HCD |  | C15(Dehydro); C21(Dehydro) |  | 2.36 |  | 3.1e-3 |  | 8.6e-2 |  | 0.216 |  | 224.69 |  | 1.000 |  |  |  | C15(Dehydro): 100; C21(Dehydro): 100 |  |  |  |  |  |  |  | 0.46 |  | 1 |  | Sequest HT (3) |  | 3 |  | 693.38171 |  | -0.83 |  | 45.4581 |  | 13435 |  | 13435 |  | TRINITY\_DN49\_c0\_g1\_i1.p1\_ORF1 |  | Image |  | Peak List |  |
|  | | | | | | | | | | | | | | | | | | | | | | | | | | | | | | | | | | | | | | | | | | | | | | | | | | | | | |  |
|  | High |  | [R].GLFGKILGVGKKVLcGLSGMc.[-] |  | HCD |  | C15(Dehydro); C21(Dehydro) |  | 4.44 |  | 1.3e-3 |  | 5.6e-4 |  | 1.32 |  | 395.37 |  | 1.000 |  |  |  | C15(Dehydro): 100; C21(Dehydro): 100 |  |  |  |  |  |  |  | 0.53 |  | 1 |  | Sequest HT (3) |  | 2 |  | 1039.56909 |  | -0.67 |  | 45.5721 |  | 13482 |  | 13482 |  | TRINITY\_DN49\_c0\_g1\_i1.p1\_ORF1 |  | Image |  | Peak List |  |
|  | | | | | | | | | | | | | | | | | | | | | | | | | | | | | | | | | | | | | | | | | | | | | | | | | | | | | |  |
|  | High |  | [R].GLFGKILGVGKKVLcGLSGMc.[-] |  | HCD |  | C15(Dehydro); C21(Dehydro) |  | 2.78 |  | 3.9e-3 |  | 1.5e-1 |  | 0.085 |  | 226.19 |  | 1.000 |  |  |  | C15(Dehydro): 100; C21(Dehydro): 100 |  |  |  |  |  |  |  | 0.55 |  | 1 |  | Sequest HT (3) |  | 3 |  | 693.38171 |  | -0.83 |  | 45.5924 |  | 13491 |  | 13491 |  | TRINITY\_DN49\_c0\_g1\_i1.p1\_ORF1 |  | Image |  | Peak List |  |
|  | | | | | | | | | | | | | | | | | | | | | | | | | | | | | | | | | | | | | | | | | | | | | | | | | | | | | |  |
|  | High |  | [R].GLFGKILGVGKKVLcGLSGMc.[-] |  | HCD |  | C15(Dehydro); C21(Dehydro) |  | 4.37 |  | 1.3e-3 |  | 4.4e-3 |  | 0.871 |  | 407.01 |  | 1.000 |  |  |  | C15(Dehydro): 100; C21(Dehydro): 100 |  |  |  |  |  |  |  | 0.46 |  | 1 |  | Sequest HT (3) |  | 2 |  | 1039.56909 |  | -0.67 |  | 45.7073 |  | 13537 |  | 13537 |  | TRINITY\_DN49\_c0\_g1\_i1.p1\_ORF1 |  | Image |  | Peak List |  |
|  | | | | | | | | | | | | | | | | | | | | | | | | | | | | | | | | | | | | | | | | | | | | | | | | | | | | | |  |
|  | High |  | [R].GLFGKILGVGKKVLcGLSGMc.[-] |  | HCD |  | C15(Dehydro); C21(Dehydro) |  | 4.09 |  | 1.3e-3 |  | 3.7e-4 |  | 1.41 |  | 412.22 |  | 1.000 |  |  |  | C15(Dehydro): 100; C21(Dehydro): 100 |  |  |  |  |  |  |  | 0.51 |  | 1 |  | Sequest HT (3) |  | 2 |  | 1039.56921 |  | -0.56 |  | 45.8470 |  | 13603 |  | 13603 |  | TRINITY\_DN49\_c0\_g1\_i1.p1\_ORF1 |  | Image |  | Peak List |  |
|  | | | | | | | | | | | | | | | | | | | | | | | | | | | | | | | | | | | | | | | | | | | | | | | | | | | | | |  |
|  | High |  | [R].GLFGKILGVGKKVLcGLSGMc.[-] |  | HCD |  | C15(Dehydro); C21(Dehydro) |  | 4.26 |  | 1.3e-3 |  | 1.2e-3 |  | 1.15 |  | 399.87 |  | 1.000 |  |  |  | C15(Dehydro): 100; C21(Dehydro): 100 |  |  |  |  |  |  |  | 0.50 |  | 1 |  | Sequest HT (3) |  | 2 |  | 1039.56970 |  | -0.09 |  | 46.0396 |  | 13687 |  | 13687 |  | TRINITY\_DN49\_c0\_g1\_i1.p1\_ORF1 |  | Image |  | Peak List |  |
|  | | | | | | | | | | | | | | | | | | | | | | | | | | | | | | | | | | | | | | | | | | | | | | | | | | | | | |  |
|  | High |  | [R].GLFGKILGVGKKVLcGLSGMc.[-] |  | HCD |  | C15(Dehydro); C21(Dehydro) |  | 2.34 |  | 3.9e-3 |  | 1.8e-1 |  | 0.049 |  | 191.68 |  | 1.000 |  |  |  | C15(Dehydro): 100; C21(Dehydro): 100 |  |  |  |  |  |  |  | 0.51 |  | 1 |  | Sequest HT (3) |  | 3 |  | 693.38214 |  | -0.21 |  | 46.1626 |  | 13743 |  | 13743 |  | TRINITY\_DN49\_c0\_g1\_i1.p1\_ORF1 |  | Image |  | Peak List |  |
|  | | | | | | | | | | | | | | | | | | | | | | | | | | | | | | | | | | | | | | | | | | | | | | | | | | | | | |  |
|  | High |  | [R].GLFGKILGVGKKVLcGLSGMc.[-] |  | HCD |  | C15(Dehydro); C21(Dehydro) |  | 3.95 |  | 1.3e-3 |  | 3.8e-4 |  | 1.41 |  | 435.95 |  | 1.000 |  |  |  | C15(Dehydro): 100; C21(Dehydro): 100 |  |  |  |  |  |  |  | 0.54 |  | 1 |  | Sequest HT (3) |  | 2 |  | 1039.56946 |  | -0.32 |  | 46.1835 |  | 13753 |  | 13753 |  | TRINITY\_DN49\_c0\_g1\_i1.p1\_ORF1 |  | Image |  | Peak List |  |
|  | | | | | | | | | | | | | | | | | | | | | | | | | | | | | | | | | | | | | | | | | | | | | | | | | | | | | |  |
|  | High |  | [R].GLFGKILGVGKKVLcGLSGMc.[-] |  | HCD |  | C15(Dehydro); C21(Dehydro) |  | 2.41 |  | 2.4e-3 |  | 7.5e-2 |  | 0.247 |  | 213.17 |  | 1.000 |  |  |  | C15(Dehydro): 100; C21(Dehydro): 100 |  |  |  |  |  |  |  | 0.45 |  | 1 |  | Sequest HT (3) |  | 3 |  | 693.38184 |  | -0.65 |  | 46.3063 |  | 13811 |  | 13811 |  | TRINITY\_DN49\_c0\_g1\_i1.p1\_ORF1 |  | Image |  | Peak List |  |
|  | | | | | | | | | | | | | | | | | | | | | | | | | | | | | | | | | | | | | | | | | | | | | | | | | | | | | |  |
|  | High |  | [R].GLFGKILGVGKKVLcGLSGMc.[-] |  | HCD |  | C15(Dehydro); C21(Dehydro) |  | 4.10 |  | 1.3e-3 |  | 2.3e-4 |  | 1.51 |  | 410.04 |  | 1.000 |  |  |  | C15(Dehydro): 100; C21(Dehydro): 100 |  |  |  |  |  |  |  | 0.50 |  | 1 |  | Sequest HT (3) |  | 2 |  | 1039.56921 |  | -0.56 |  | 46.3288 |  | 13822 |  | 13822 |  | TRINITY\_DN49\_c0\_g1\_i1.p1\_ORF1 |  | Image |  | Peak List |  |
|  | | | | | | | | | | | | | | | | | | | | | | | | | | | | | | | | | | | | | | | | | | | | | | | | | | | | | |  |
|  | High |  | [R].GLFGKILGVGKKVLcGLSGMc.[-] |  | HCD |  | C15(Dehydro); C21(Dehydro) |  | 2.72 |  | 3.1e-3 |  | 1.3e-1 |  | 0.121 |  | 208.72 |  | 1.000 |  |  |  | C15(Dehydro): 100; C21(Dehydro): 100 |  |  |  |  |  |  |  | 0.55 |  | 1 |  | Sequest HT (3) |  | 3 |  | 693.38123 |  | -1.53 |  | 46.4704 |  | 13884 |  | 13884 |  | TRINITY\_DN49\_c0\_g1\_i1.p1\_ORF1 |  | Image |  | Peak List |  |
|  | | | | | | | | | | | | | | | | | | | | | | | | | | | | | | | | | | | | | | | | | | | | | | | | | | | | | |  |
|  | High |  | [R].GLFGKILGVGKKVLcGLSGMc.[-] |  | HCD |  | C15(Dehydro); C21(Dehydro) |  | 4.27 |  | 1.3e-3 |  | 3.4e-4 |  | 1.43 |  | 399.87 |  | 1.000 |  |  |  | C15(Dehydro): 100; C21(Dehydro): 100 |  |  |  |  |  |  |  | 0.49 |  | 1 |  | Sequest HT (3) |  | 2 |  | 1039.56873 |  | -1.03 |  | 46.4769 |  | 13886 |  | 13886 |  | TRINITY\_DN49\_c0\_g1\_i1.p1\_ORF1 |  | Image |  | Peak List |  |
|  | | | | | | | | | | | | | | | | | | | | | | | | | | | | | | | | | | | | | | | | | | | | | | | | | | | | | |  |
|  | High |  | [R].GLFGKILGVGKKVLcGLSGMc.[-] |  | HCD |  | C15(Dehydro); C21(Dehydro) |  | 4.30 |  | 1.3e-3 |  | 2.6e-5 |  | 1.99 |  | 440.72 |  | 1.000 |  |  |  | C15(Dehydro): 100; C21(Dehydro): 100 |  |  |  |  |  |  |  | 0.53 |  | 1 |  | Sequest HT (3) |  | 2 |  | 1039.56689 |  | -2.79 |  | 46.6220 |  | 13947 |  | 13947 |  | TRINITY\_DN49\_c0\_g1\_i1.p1\_ORF1 |  | Image |  | Peak List |  |
|  | | | | | | | | | | | | | | | | | | | | | | | | | | | | | | | | | | | | | | | | | | | | | | | | | | | | | |  |
|  | High |  | [R].GLFGKILGVGKKVLcGLSGMc.[-] |  | HCD |  | C15(Dehydro); C21(Dehydro) |  | 2.24 |  | 1.3e-3 |  | 3.2e-2 |  | 0.434 |  | 207.45 |  | 1.000 |  |  |  | C15(Dehydro): 100; C21(Dehydro): 100 |  |  |  |  |  |  |  | 0.42 |  | 1 |  | Sequest HT (3) |  | 3 |  | 693.38165 |  | -0.91 |  | 46.7535 |  | 14002 |  | 14002 |  | TRINITY\_DN49\_c0\_g1\_i1.p1\_ORF1 |  | Image |  | Peak List |  |
|  | | | | | | | | | | | | | | | | | | | | | | | | | | | | | | | | | | | | | | | | | | | | | | | | | | | | | |  |
|  | High |  | [R].GLFGKILGVGKKVLcGLSGMc.[-] |  | HCD |  | C15(Dehydro); C21(Dehydro) |  | 4.23 |  | 1.3e-3 |  | 2.6e-4 |  | 1.49 |  | 399.87 |  | 1.000 |  |  |  | C15(Dehydro): 100; C21(Dehydro): 100 |  |  |  |  |  |  |  | 0.52 |  | 1 |  | Sequest HT (3) |  | 2 |  | 1039.56860 |  | -1.14 |  | 46.8267 |  | 14034 |  | 14034 |  | TRINITY\_DN49\_c0\_g1\_i1.p1\_ORF1 |  | Image |  | Peak List |  |
|  | | | | | | | | | | | | | | | | | | | | | | | | | | | | | | | | | | | | | | | | | | | | | | | | | | | | | |  |
|  | High |  | [R].GLFGKILGVGKKVLcGLSGMc.[-] |  | HCD |  | C15(Dehydro); C21(Dehydro) |  | 2.59 |  | 3.9e-3 |  | 1.7e-1 |  | 0.057 |  | 230.32 |  | 1.000 |  |  |  | C15(Dehydro): 100; C21(Dehydro): 100 |  |  |  |  |  |  |  | 0.52 |  | 1 |  | Sequest HT (3) |  | 3 |  | 693.38153 |  | -1.09 |  | 46.9005 |  | 14064 |  | 14064 |  | TRINITY\_DN49\_c0\_g1\_i1.p1\_ORF1 |  | Image |  | Peak List |  |
|  | | | | | | | | | | | | | | | | | | | | | | | | | | | | | | | | | | | | | | | | | | | | | | | | | | | | | |  |
|  | High |  | [R].GLFGKILGVGKKVLcGLSGMc.[-] |  | HCD |  | C15(Dehydro); C21(Dehydro) |  | 4.63 |  | 1.3e-3 |  | 1.2e-4 |  | 1.66 |  | 474.75 |  | 1.000 |  |  |  | C15(Dehydro): 100; C21(Dehydro): 100 |  |  |  |  |  |  |  | 0.48 |  | 1 |  | Sequest HT (3) |  | 2 |  | 1039.56848 |  | -1.26 |  | 46.9835 |  | 14099 |  | 14099 |  | TRINITY\_DN49\_c0\_g1\_i1.p1\_ORF1 |  | Image |  | Peak List |  |
|  | | | | | | | | | | | | | | | | | | | | | | | | | | | | | | | | | | | | | | | | | | | | | | | | | | | | | |  |
|  | High |  | [R].GLFGKILGVGKKVLcGLSGMc.[-] |  | HCD |  | C15(Dehydro); C21(Dehydro) |  | 2.56 |  | 3.9e-3 |  | 1.7e-1 |  | 0.062 |  | 233.76 |  | 1.000 |  |  |  | C15(Dehydro): 100; C21(Dehydro): 100 |  |  |  |  |  |  |  | 0.52 |  | 1 |  | Sequest HT (3) |  | 3 |  | 693.38068 |  | -2.32 |  | 47.1917 |  | 14198 |  | 14198 |  | TRINITY\_DN49\_c0\_g1\_i1.p1\_ORF1 |  | Image |  | Peak List |  |
|  | | | | | | | | | | | | | | | | | | | | | | | | | | | | | | | | | | | | | | | | | | | | | | | | | | | | | |  |
|  | High |  | [R].GLFGKILGVGKKVLcGLSGMc.[-] |  | HCD |  | C15(Dehydro); C21(Dehydro) |  | 2.27 |  | 2.4e-3 |  | 7.2e-2 |  | 0.256 |  | 184.52 |  | 1.000 |  |  |  | C15(Dehydro): 100; C21(Dehydro): 100 |  |  |  |  |  |  |  | 0.33 |  | 1 |  | Sequest HT (3) |  | 3 |  | 693.38043 |  | -2.68 |  | 47.3327 |  | 14259 |  | 14259 |  | TRINITY\_DN49\_c0\_g1\_i1.p1\_ORF1 |  | Image |  | Peak List |  |
|  | | | | | | | | | | | | | | | | | | | | | | | | | | | | | | | | | | | | | | | | | | | | | | | | | | | | | |  |
|  | High |  | [R].GLFGKILGVGKKVLcGLSGMc.[-] |  | HCD |  | C15(Dehydro); C21(Dehydro) |  | 4.09 |  | 1.3e-3 |  | 2.0e-3 |  | 1.04 |  | 380.81 |  | 1.000 |  |  |  | C15(Dehydro): 100; C21(Dehydro): 100 |  |  |  |  |  |  |  | 0.48 |  | 1 |  | Sequest HT (3) |  | 2 |  | 1039.56763 |  | -2.08 |  | 47.4202 |  | 14295 |  | 14295 |  | TRINITY\_DN49\_c0\_g1\_i1.p1\_ORF1 |  | Image |  | Peak List |  |
|  | | | | | | | | | | | | | | | | | | | | | | | | | | | | | | | | | | | | | | | | | | | | | | | | | | | | | |  |
|  | High |  | [R].GLFGKILGVGKKVLcGLSGMc.[-] |  | HCD |  | C15(Dehydro); C21(Dehydro) |  | 2.53 |  | 3.1e-3 |  | 1.3e-1 |  | 0.116 |  | 215.53 |  | 1.000 |  |  |  | C15(Dehydro): 100; C21(Dehydro): 100 |  |  |  |  |  |  |  | 0.34 |  | 1 |  | Sequest HT (3) |  | 3 |  | 693.38074 |  | -2.24 |  | 47.4767 |  | 14317 |  | 14317 |  | TRINITY\_DN49\_c0\_g1\_i1.p1\_ORF1 |  | Image |  | Peak List |  |
|  | | | | | | | | | | | | | | | | | | | | | | | | | | | | | | | | | | | | | | | | | | | | | | | | | | | | | |  |
|  | High |  | [R].GLFGKILGVGKKVLcGLSGMc.[-] |  | HCD |  | C15(Dehydro); C21(Dehydro) |  | 1.82 |  | 3.1e-3 |  | 1.4e-1 |  | 0.105 |  | 164.18 |  | 1.000 |  |  |  | C15(Dehydro): 100; C21(Dehydro): 100 |  |  |  |  |  |  |  | 0.50 |  | 1 |  | Sequest HT (3) |  | 3 |  | 693.38116 |  | -1.62 |  | 47.6289 |  | 14377 |  | 14377 |  | TRINITY\_DN49\_c0\_g1\_i1.p1\_ORF1 |  | Image |  | Peak List |  |
|  | | | | | | | | | | | | | | | | | | | | | | | | | | | | | | | | | | | | | | | | | | | | | | | | | | | | | |  |
|  | High |  | [R].GLFGKILGVGKKVLcGLSGMc.[-] |  | HCD |  | C15(Dehydro); C21(Dehydro) |  | 4.23 |  | 1.3e-3 |  | 5.2e-4 |  | 1.34 |  | 440.72 |  | 1.000 |  |  |  | C15(Dehydro): 100; C21(Dehydro): 100 |  |  |  |  |  |  |  | 0.49 |  | 1 |  | Sequest HT (3) |  | 2 |  | 1039.56885 |  | -0.91 |  | 47.7206 |  | 14418 |  | 14418 |  | TRINITY\_DN49\_c0\_g1\_i1.p1\_ORF1 |  | Image |  | Peak List |  |
|  | | | | | | | | | | | | | | | | | | | | | | | | | | | | | | | | | | | | | | | | | | | | | | | | | | | | | |  |
|  | High |  | [R].GLFGKILGVGKKVLcGLSGMc.[-] |  | HCD |  | C15(Dehydro); C21(Dehydro) |  | 4.43 |  | 1.3e-3 |  | 3.5e-4 |  | 1.42 |  | 438.31 |  | 1.000 |  |  |  | C15(Dehydro): 100; C21(Dehydro): 100 |  |  |  |  |  |  |  | 0.58 |  | 1 |  | Sequest HT (3) |  | 2 |  | 1039.56946 |  | -0.32 |  | 48.0153 |  | 14541 |  | 14541 |  | TRINITY\_DN49\_c0\_g1\_i1.p1\_ORF1 |  | Image |  | Peak List |  |
|  | | | | | | | | | | | | | | | | | | | | | | | | | | | | | | | | | | | | | | | | | | | | | | | | | | | | | |  |
|  | High |  | [R].GLFGKILGVGKKVLcGLSGMc.[-] |  | HCD |  | C15(Dehydro); C21(Dehydro) |  | 3.06 |  | 3.1e-3 |  | 1.3e-1 |  | 0.118 |  | 220.25 |  | 1.000 |  |  |  | C15(Dehydro): 100; C21(Dehydro): 100 |  |  |  |  |  |  |  | 0.48 |  | 1 |  | Sequest HT (3) |  | 3 |  | 693.38135 |  | -1.35 |  | 48.0742 |  | 14565 |  | 14565 |  | TRINITY\_DN49\_c0\_g1\_i1.p1\_ORF1 |  | Image |  | Peak List |  |
|  | | | | | | | | | | | | | | | | | | | | | | | | | | | | | | | | | | | | | | | | | | | | | | | | | | | | | |  |
|  | High |  | [R].GLFGKILGVGKKVLcGLSGMc.[-] |  | HCD |  | C15(Dehydro); C21(Dehydro) |  | 2.43 |  | 1.3e-3 |  | 2.5e-2 |  | 0.494 |  | 305.14 |  | 1.000 |  |  |  | C15(Dehydro): 100; C21(Dehydro): 100 |  |  |  |  |  |  |  | 0.34 |  | 1 |  | Sequest HT (3) |  | 2 |  | 1039.56714 |  | -2.55 |  | 48.1567 |  | 14602 |  | 14602 |  | TRINITY\_DN49\_c0\_g1\_i1.p1\_ORF1 |  | Image |  | Peak List |  |
|  | | | | | | | | | | | | | | | | | | | | | | | | | | | | | | | | | | | | | | | | | | | | | | | | | | | | | |  |
|  | High |  | [R].GLFGKILGVGKKVLcGLSGMc.[-] |  | HCD |  | C15(Dehydro); C21(Dehydro) |  | 2.66 |  | 3.9e-3 |  | 1.5e-1 |  | 0.097 |  | 190.11 |  | 1.000 |  |  |  | C15(Dehydro): 100; C21(Dehydro): 100 |  |  |  |  |  |  |  | 0.50 |  | 1 |  | Sequest HT (3) |  | 3 |  | 693.38165 |  | -0.91 |  | 48.2631 |  | 14650 |  | 14650 |  | TRINITY\_DN49\_c0\_g1\_i1.p1\_ORF1 |  | Image |  | Peak List |  |
|  | | | | | | | | | | | | | | | | | | | | | | | | | | | | | | | | | | | | | | | | | | | | | | | | | | | | | |  |
|  | High |  | [R].GLFGKILGVGKKVLcGLSGMc.[-] |  | HCD |  | C15(Dehydro); C21(Dehydro) |  | 4.09 |  | 1.3e-3 |  | 5.8e-4 |  | 1.31 |  | 395.37 |  | 1.000 |  |  |  | C15(Dehydro): 100; C21(Dehydro): 100 |  |  |  |  |  |  |  | 0.51 |  | 1 |  | Sequest HT (3) |  | 2 |  | 1039.57056 |  | 0.74 |  | 48.6963 |  | 14821 |  | 14821 |  | TRINITY\_DN49\_c0\_g1\_i1.p1\_ORF1 |  | Image |  | Peak List |  |
|  | | | | | | | | | | | | | | | | | | | | | | | | | | | | | | | | | | | | | | | | | | | | | | | | | | | | | |  |
|  | High |  | [R].GLFGKILGVGKKVLcGLSGMc.[-] |  | HCD |  | C15(Dehydro); C21(Dehydro) |  | 3.52 |  | 1.3e-3 |  | 7.8e-4 |  | 1.25 |  | 399.87 |  | 1.000 |  |  |  | C15(Dehydro): 100; C21(Dehydro): 100 |  |  |  |  |  |  |  | 0.58 |  | 1 |  | Sequest HT (3) |  | 2 |  | 1039.56909 |  | -0.67 |  | 48.9967 |  | 14947 |  | 14947 |  | TRINITY\_DN49\_c0\_g1\_i1.p1\_ORF1 |  | Image |  | Peak List |  |
|  | | | | | | | | | | | | | | | | | | | | | | | | | | | | | | | | | | | | | | | | | | | | | | | | | | | | | |  |
|  | High |  | [R].GLFGKILGVGKKVLcGLSGMc.[-] |  | HCD |  | C15(Dehydro); C21(Dehydro) |  | 4.34 |  | 1.3e-3 |  | 9.0e-5 |  | 1.72 |  | 423.83 |  | 1.000 |  |  |  | C15(Dehydro): 100; C21(Dehydro): 100 |  |  |  |  |  |  |  | 0.48 |  | 1 |  | Sequest HT (3) |  | 2 |  | 1039.56958 |  | -0.20 |  | 49.3528 |  | 15089 |  | 15089 |  | TRINITY\_DN49\_c0\_g1\_i1.p1\_ORF1 |  | Image |  | Peak List |  |
|  | | | | | | | | | | | | | | | | | | | | | | | | | | | | | | | | | | | | | | | | | | | | | | | | | | | | | |  |
|  | High |  | [R].GLFGKILGVGKKVLcGLSGMc.[-] |  | HCD |  | C15(Dehydro); C21(Dehydro) |  | 4.31 |  | 1.3e-3 |  | 4.5e-4 |  | 1.37 |  | 419.03 |  | 1.000 |  |  |  | C15(Dehydro): 100; C21(Dehydro): 100 |  |  |  |  |  |  |  | 0.50 |  | 1 |  | Sequest HT (3) |  | 2 |  | 1039.57190 |  | 2.03 |  | 49.5119 |  | 15152 |  | 15152 |  | TRINITY\_DN49\_c0\_g1\_i1.p1\_ORF1 |  | Image |  | Peak List |  |
|  | | | | | | | | | | | | | | | | | | | | | | | | | | | | | | | | | | | | | | | | | | | | | | | | | | | | | |  |
|  | High |  | [R].GLFGKILGVGKKVLcGLSGMc.[-] |  | HCD |  | C15(Dehydro); C21(Dehydro) |  | 4.24 |  | 1.3e-3 |  | 6.3e-4 |  | 1.29 |  | 393.19 |  | 1.000 |  |  |  | C15(Dehydro): 100; C21(Dehydro): 100 |  |  |  |  |  |  |  | 0.47 |  | 1 |  | Sequest HT (3) |  | 2 |  | 1039.56934 |  | -0.44 |  | 49.6714 |  | 15215 |  | 15215 |  | TRINITY\_DN49\_c0\_g1\_i1.p1\_ORF1 |  | Image |  | Peak List |  |
|  | | | | | | | | | | | | | | | | | | | | | | | | | | | | | | | | | | | | | | | | | | | | | | | | | | | | | |  |
|  | High |  | [R].GLFGKILGVGKKVLcGLSGMc.[-] |  | HCD |  | C15(Dehydro); C21(Dehydro) |  | 3.19 |  | 1.3e-3 |  | 5.8e-2 |  | 0.306 |  | 234.64 |  | 1.000 |  |  |  | C15(Dehydro): 100; C21(Dehydro): 100 |  |  |  |  |  |  |  | 0.40 |  | 1 |  | Sequest HT (3) |  | 3 |  | 693.38068 |  | -2.32 |  | 49.8876 |  | 15306 |  | 15306 |  | TRINITY\_DN49\_c0\_g1\_i1.p1\_ORF1 |  | Image |  | Peak List |  |
|  | | | | | | | | | | | | | | | | | | | | | | | | | | | | | | | | | | | | | | | | | | | | | | | | | | | | | |  |
|  | High |  | [R].GLFGKILGVGKKVLcGLSGMc.[-] |  | HCD |  | C15(Dehydro); C21(Dehydro) |  | 3.97 |  | 1.3e-3 |  | 1.6e-3 |  | 1.09 |  | 407.01 |  | 1.000 |  |  |  | C15(Dehydro): 100; C21(Dehydro): 100 |  |  |  |  |  |  |  | 0.53 |  | 1 |  | Sequest HT (3) |  | 2 |  | 1039.56799 |  | -1.73 |  | 50.0657 |  | 15378 |  | 15378 |  | TRINITY\_DN49\_c0\_g1\_i1.p1\_ORF1 |  | Image |  | Peak List |  |
|  | | | | | | | | | | | | | | | | | | | | | | | | | | | | | | | | | | | | | | | | | | | | | | | | | | | | | |  |
|  | High |  | [R].GLFGKILGVGKKVLcGLSGMc.[-] |  | HCD |  | C15(Dehydro); C21(Dehydro) |  | 2.66 |  | 3.1e-3 |  | 8.6e-2 |  | 0.217 |  | 187.87 |  | 1.000 |  |  |  | C15(Dehydro): 100; C21(Dehydro): 100 |  |  |  |  |  |  |  | 0.42 |  | 1 |  | Sequest HT (3) |  | 3 |  | 693.38031 |  | -2.85 |  | 50.0885 |  | 15385 |  | 15385 |  | TRINITY\_DN49\_c0\_g1\_i1.p1\_ORF1 |  | Image |  | Peak List |  |
|  | | | | | | | | | | | | | | | | | | | | | | | | | | | | | | | | | | | | | | | | | | | | | | | | | | | | | |  |
|  | High |  | [R].GLFGKILGVGKKVLcGLSGMc.[-] |  | HCD |  | C15(Dehydro); C21(Dehydro) |  | 4.35 |  | 1.3e-3 |  | 8.0e-4 |  | 1.24 |  | 399.87 |  | 1.000 |  |  |  | C15(Dehydro): 100; C21(Dehydro): 100 |  |  |  |  |  |  |  | 0.48 |  | 1 |  | Sequest HT (3) |  | 2 |  | 1039.56750 |  | -2.20 |  | 50.2437 |  | 15446 |  | 15446 |  | TRINITY\_DN49\_c0\_g1\_i1.p1\_ORF1 |  | Image |  | Peak List |  |
|  | | | | | | | | | | | | | | | | | | | | | | | | | | | | | | | | | | | | | | | | | | | | | | | | | | | | | |  |
|  | High |  | [R].GLFGKILGVGKKVLcGLSGMc.[-] |  | HCD |  | C15(Dehydro); C21(Dehydro) |  | 3.75 |  | 1.3e-3 |  | 9.0e-4 |  | 1.22 |  | 388.96 |  | 1.000 |  |  |  | C15(Dehydro): 100; C21(Dehydro): 100 |  |  |  |  |  |  |  | 0.46 |  | 1 |  | Sequest HT (3) |  | 2 |  | 1039.56824 |  | -1.50 |  | 51.5005 |  | 15941 |  | 15941 |  | TRINITY\_DN49\_c0\_g1\_i1.p1\_ORF1 |  | Image |  | Peak List |  |
|  | | | | | | | | | | | | | | | | | | | | | | | | | | | | | | | | | | | | | | | | | | | | | | | | | | | | | |  |
|  | High |  | [R].GLFGKILGVGKKVLcGLSGMc.[-] |  | HCD |  | C15(Dehydro); C21(Dehydro) |  | 2.67 |  | 3.1e-3 |  | 9.0e-2 |  | 0.206 |  | 183.77 |  | 1.000 |  |  |  | C15(Dehydro): 100; C21(Dehydro): 100 |  |  |  |  |  |  |  | 0.43 |  | 1 |  | Sequest HT (3) |  | 3 |  | 693.38092 |  | -1.97 |  | 52.2173 |  | 16236 |  | 16236 |  | TRINITY\_DN49\_c0\_g1\_i1.p1\_ORF1 |  | Image |  | Peak List |  |
|  | | | | | | | | | | | | | | | | | | | | | | | | | | | | | | | | | | | | | | | | | | | | | | | | | | | | | |  |
|  | High |  | [R].GLFGKILGVGKKVLcGLSGMc.[-] |  | HCD |  | C15(Dehydro); C21(Dehydro) |  | 2.42 |  | 3.1e-3 |  | 1.3e-1 |  | 0.123 |  | 164.97 |  | 1.000 |  |  |  | C15(Dehydro): 100; C21(Dehydro): 100 |  |  |  |  |  |  |  | 0.33 |  | 1 |  | Sequest HT (3) |  | 2 |  | 1039.56726 |  | -2.44 |  | 62.1937 |  | 19538 |  | 19538 |  | TRINITY\_DN49\_c0\_g1\_i1.p1\_ORF1 |  | Image |  | Peak List |  |
|  | | | | | | | | | | | | | | | | | | | | | | | | | | | | | | | | | | | | | | | | | | | | | | | | | | | | | |  |
|  | High |  | [R].GLFGKILGVGKKVLcGLSGMc.[-] |  | HCD |  | C15(Dehydro); C21(Dehydro) |  | 3.35 |  | 3.1e-3 |  | 1.2e-1 |  | 0.146 |  | 243.32 |  | 1.000 |  |  |  | C15(Dehydro): 100; C21(Dehydro): 100 |  |  |  |  |  |  |  | 0.49 |  | 1 |  | Sequest HT (3) |  | 3 |  | 693.38300 |  | 1.02 |  | 62.5155 |  | 19629 |  | 19629 |  | TRINITY\_DN49\_c0\_g1\_i1.p1\_ORF1 |  | Image |  | Peak List |  |
|  | | | | | | | | | | | | | | | | | | | | | | | | | | | | | | | | | | | | | | | | | | | | | | | | | | | | | |  |
|  | High |  | [R].GLFGKILGVGKKVLcGLSGMc.[-] |  | HCD |  | C15(Dehydro); C21(Dehydro) |  | 2.53 |  | 3.9e-3 |  | 1.5e-1 |  | 0.081 |  | 182.39 |  | 1.000 |  |  |  | C15(Dehydro): 100; C21(Dehydro): 100 |  |  |  |  |  |  |  | 0.45 |  | 1 |  | Sequest HT (3) |  | 3 |  | 693.38275 |  | 0.67 |  | 62.6577 |  | 19665 |  | 19665 |  | TRINITY\_DN49\_c0\_g1\_i1.p1\_ORF1 |  | Image |  | Peak List |  |
|  | | | | | | | | | | | | | | | | | | | | | | | | | | | | | | | | | | | | | | | | | | | | | | | | | | | | | |  |
|  | High |  | [R].GLFGKILGVGKKVLcGLSGMc.[-] |  | HCD |  | C15(Dehydro); C21(Dehydro) |  | 3.47 |  | 3.1e-3 |  | 9.6e-2 |  | 0.192 |  | 219.00 |  | 1.000 |  |  |  | C15(Dehydro): 100; C21(Dehydro): 100 |  |  |  |  |  |  |  | 0.46 |  | 1 |  | Sequest HT (3) |  | 3 |  | 693.38208 |  | -0.30 |  | 62.8006 |  | 19701 |  | 19701 |  | TRINITY\_DN49\_c0\_g1\_i1.p1\_ORF1 |  | Image |  | Peak List |  |
|  | | | | | | | | | | | | | | | | | | | | | | | | | | | | | | | | | | | | | | | | | | | | | | | | | | | | | |  |
|  | **TRINITY\_DN96\_c0\_g2\_i2.p1 - TRINITY\_DN96\_c0\_g2\_i2.p1** | | | | | | | | | | | | | | | | | | | | | | | | | | | | | | | | | | | | | | | | | | | | | | | | | | | |  |  |
|  | | | | | | | | | | | | | | | | | | | | | | | | | | | | | | | | | | | | | | | | | | | | | | | | | | | | | |  |
|  | High |  | [R].GIFSILKIA.[T] |  | HCD |  |  |  | 1.96 |  | 1.3e-3 |  | 5.9e-2 |  | 0.3 |  |  |  |  |  |  |  |  |  |  |  |  |  |  |  | 0.30 |  | 1 |  | Sequest HT (3) |  | 2 |  | 481.30704 |  | -1.33 |  | 37.9311 |  | 10043 |  | 10043 |  | TRINITY\_DN96\_c0\_g2\_i2.p1; TRINITY\_DN96\_c0\_g2\_i1.p1 |  | Image |  | Peak List |  |
|  | | | | | | | | | | | | | | | | | | | | | | | | | | | | | | | | | | | | | | | | | | | | | | | | | | | | | |  |
|  | High |  | [R].GIFSILKIATKLIGKTLAKAAGKAGAELAAcKAANQc.[-] |  | HCD |  | C31(Dehydro); C37(Dehydro) |  | 5.81 |  | 1.3e-3 |  | 1.9e-3 |  | 1.05 |  | 209.34 |  | 1.000 |  |  |  | C31(Dehydro): 100; C37(Dehydro): 100 |  |  |  |  |  |  |  | 0.76 |  | 1 |  | Sequest HT (3) |  | 4 |  | 911.27081 |  | 0.46 |  | 41.6678 |  | 11851 |  | 11851 |  | TRINITY\_DN96\_c0\_g2\_i2.p1 |  | Image |  | Peak List |  |
|  | | | | | | | | | | | | | | | | | | | | | | | | | | | | | | | | | | | | | | | | | | | | | | | | | | | | | |  |
|  | High |  | [R].GIFSILKIATKLIGKTLAKAAGKAGAELAAcKAAnQc.[-] |  | HCD |  | C31(Dehydro); N35(Deamidated); C37(Dehydro) |  | 5.64 |  | 1.3e-3 |  | 4.4e-3 |  | 0.87 |  | 207.74 |  | 1.000 |  |  |  | C31(Dehydro): 100; N35(Deamidated): 100; C37(Dehydro): 100 |  |  |  |  |  |  |  | 0.67 |  | 1 |  | Sequest HT (3) |  | 4 |  | 911.51624 |  | -0.18 |  | 41.9664 |  | 11983 |  | 11983 |  | TRINITY\_DN96\_c0\_g2\_i2.p1 |  | Image |  | Peak List |  |
|  | | | | | | | | | | | | | | | | | | | | | | | | | | | | | | | | | | | | | | | | | | | | | | | | | | | | | |  |
|  | **TRINITY\_DN0\_c1\_g1\_i22.p1 - TRINITY\_DN0\_c1\_g1\_i22.p1** | | | | | | | | | | | | | | | | | | | | | | | | | | | | | | | | | | | | | | | | | | | | | | | | | | | |  |  |
|  | | | | | | | | | | | | | | | | | | | | | | | | | | | | | | | | | | | | | | | | | | | | | | | | | | | | | |  |
|  | High |  | [R].GLFTLIKGAAK.[L] |  | HCD |  |  |  | 2.51 |  | 4.7e-3 |  | 2.0e-1 |  | 0.022 |  |  |  |  |  |  |  |  |  |  |  |  |  |  |  | 0.29 |  | 1 |  | Sequest HT (3) |  | 2 |  | 559.84918 |  | -1.89 |  | 27.3090 |  | 5575 |  | 5575 |  | TRINITY\_DN49\_c10\_g1\_i1.p1; TRINITY\_DN0\_c1\_g1\_i22.p1; TRINITY\_DN591\_c1\_g1\_i1.p1; TRINITY\_DN0\_c1\_g1\_i7.p1 |  | Image |  | Peak List |  |
|  | | | | | | | | | | | | | | | | | | | | | | | | | | | | | | | | | | | | | | | | | | | | | | | | | | | | | |  |
|  | High |  | [R].GLFTLIKGAAK.[L] |  | HCD |  |  |  | 2.86 |  | 3.9e-3 |  | 1.5e-1 |  | 0.083 |  |  |  |  |  |  |  |  |  |  |  |  |  |  |  | 0.30 |  | 1 |  | Sequest HT (3) |  | 3 |  | 373.56909 |  | -0.43 |  | 27.3440 |  | 5587 |  | 5587 |  | TRINITY\_DN49\_c10\_g1\_i1.p1; TRINITY\_DN0\_c1\_g1\_i22.p1; TRINITY\_DN591\_c1\_g1\_i1.p1; TRINITY\_DN0\_c1\_g1\_i7.p1 |  | Image |  | Peak List |  |
|  | | | | | | | | | | | | | | | | | | | | | | | | | | | | | | | | | | | | | | | | | | | | | | | | | | | | | |  |
|  | High |  | [R].GLFTLIKGAAK.[L] |  | HCD |  |  |  | 2.22 |  | 2.4e-3 |  | 7.9e-2 |  | 0.235 |  |  |  |  |  |  |  |  |  |  |  |  |  |  |  | 0.33 |  | 1 |  | Sequest HT (3) |  | 2 |  | 559.85022 |  | -0.04 |  | 27.4488 |  | 5626 |  | 5626 |  | TRINITY\_DN49\_c10\_g1\_i1.p1; TRINITY\_DN0\_c1\_g1\_i22.p1; TRINITY\_DN591\_c1\_g1\_i1.p1; TRINITY\_DN0\_c1\_g1\_i7.p1 |  | Image |  | Peak List |  |
|  | | | | | | | | | | | | | | | | | | | | | | | | | | | | | | | | | | | | | | | | | | | | | | | | | | | | | |  |
|  | High |  | [G].AAKLIGKTVAKEAGKTGLELMAcKITNQc.[-] |  | HCD |  | C23(Dehydro); C29(Dehydro) |  | 2.77 |  | 1.3e-3 |  | 3.2e-2 |  | 0.434 |  | 187.05 |  | 1.000 |  |  |  | C23(Dehydro): 100; C29(Dehydro): 100 |  |  |  |  |  |  |  | 0.48 |  | 1 |  | Sequest HT (3) |  | 3 |  | 996.87354 |  | 0.59 |  | 31.1205 |  | 6981 |  | 6981 |  | TRINITY\_DN0\_c1\_g1\_i22.p1; TRINITY\_DN0\_c1\_g1\_i7.p1 |  | Image |  | Peak List |  |
|  | | | | | | | | | | | | | | | | | | | | | | | | | | | | | | | | | | | | | | | | | | | | | | | | | | | | | |  |
|  | High |  | [G].LFTLIKGAAKLIGKTVAKEAGK.[T] |  | HCD |  |  |  | 4.59 |  | 1.3e-3 |  | 4.8e-3 |  | 0.851 |  |  |  |  |  |  |  |  |  |  |  |  |  |  |  | 0.71 |  | 1 |  | Sequest HT (3) |  | 4 |  | 565.10590 |  | 0.51 |  | 31.3166 |  | 7057 |  | 7057 |  | TRINITY\_DN0\_c1\_g1\_i22.p1; TRINITY\_DN0\_c1\_g1\_i7.p1 |  | Image |  | Peak List |  |
|  | | | | | | | | | | | | | | | | | | | | | | | | | | | | | | | | | | | | | | | | | | | | | | | | | | | | | |  |
|  | High |  | [G].KTVAKEAGKTGLELMAcKITnQc.[-] |  | HCD |  | C17(Dehydro); N21(Deamidated); C23(Dehydro) |  | 2.50 |  | 1.3e-3 |  | 2.6e-2 |  | 0.483 |  | 279.59 |  | 1.000 |  |  |  | C17(Dehydro): 100; N21(Deamidated): 100; C23(Dehydro): 100 |  |  |  |  |  |  |  | 0.43 |  | 1 |  | Sequest HT (3) |  | 3 |  | 812.74731 |  | -0.87 |  | 32.2934 |  | 7453 |  | 7453 |  | TRINITY\_DN0\_c1\_g1\_i22.p1; TRINITY\_DN0\_c1\_g1\_i7.p1 |  | Image |  | Peak List |  |
|  | | | | | | | | | | | | | | | | | | | | | | | | | | | | | | | | | | | | | | | | | | | | | | | | | | | | | |  |
|  | High |  | [K].GAAKLIGKTVAKEAGKTGLELMAcKITNQc.[-] |  | HCD |  | C24(Dehydro); C30(Dehydro) |  | 2.77 |  | 1.3e-3 |  | 4.0e-2 |  | 0.386 |  | 146.30 |  | 1.000 |  |  |  | C24(Dehydro): 100; C30(Dehydro): 100 |  |  |  |  |  |  |  | 0.55 |  | 1 |  | Sequest HT (3) |  | 4 |  | 762.16199 |  | 0.12 |  | 32.3698 |  | 7485 |  | 7485 |  | TRINITY\_DN0\_c1\_g1\_i22.p1; TRINITY\_DN0\_c1\_g1\_i7.p1 |  | Image |  | Peak List |  |
|  | | | | | | | | | | | | | | | | | | | | | | | | | | | | | | | | | | | | | | | | | | | | | | | | | | | | | |  |
|  | High |  | [G].LFTLIKGAAKLIGKTVAKEAG.[QK] |  | HCD |  |  |  | 3.15 |  | 1.3e-3 |  | 6.3e-3 |  | 0.791 |  |  |  |  |  |  |  |  |  |  |  |  |  |  |  | 0.59 |  | 1 |  | Sequest HT (3) |  | 3 |  | 710.44080 |  | 1.03 |  | 32.5552 |  | 7565 |  | 7565 |  | TRINITY\_DN0\_c1\_g1\_i22.p1; TRINITY\_DN0\_c1\_g1\_i7.p1 |  | Image |  | Peak List |  |
|  | | | | | | | | | | | | | | | | | | | | | | | | | | | | | | | | | | | | | | | | | | | | | | | | | | | | | |  |
|  | High |  | [K].GAAKLIGKTVAKEAGKTGLELMAcKITnQc.[-] |  | HCD |  | C24(Dehydro); N28(Deamidated); C30(Dehydro) |  | 3.83 |  | 1.3e-3 |  | 3.5e-2 |  | 0.414 |  | 166.89 |  | 1.000 |  |  |  | C24(Dehydro): 100; N28(Deamidated): 100; C30(Dehydro): 100 |  |  |  |  |  |  |  | 0.60 |  | 1 |  | Sequest HT (3) |  | 4 |  | 762.40814 |  | 0.32 |  | 32.7341 |  | 7645 |  | 7645 |  | TRINITY\_DN0\_c1\_g1\_i22.p1; TRINITY\_DN0\_c1\_g1\_i7.p1 |  | Image |  | Peak List |  |
|  | | | | | | | | | | | | | | | | | | | | | | | | | | | | | | | | | | | | | | | | | | | | | | | | | | | | | |  |
|  | High |  | [R].GLFTLIKGAAKL.[LI] |  | HCD |  |  |  | 3.01 |  | 1.3e-3 |  | 6.4e-3 |  | 0.788 |  |  |  |  |  |  |  |  |  |  |  |  |  |  |  | 0.34 |  | 1 |  | Sequest HT (3) |  | 2 |  | 616.39240 |  | 0.20 |  | 33.7237 |  | 8060 |  | 8060 |  | TRINITY\_DN49\_c10\_g1\_i1.p1; TRINITY\_DN0\_c1\_g1\_i22.p1; TRINITY\_DN591\_c1\_g1\_i1.p1; TRINITY\_DN0\_c1\_g1\_i7.p1 |  | Image |  | Peak List |  |
|  | | | | | | | | | | | | | | | | | | | | | | | | | | | | | | | | | | | | | | | | | | | | | | | | | | | | | |  |
|  | High |  | [R].GLFTLIKGAAKL.[LI] |  | HCD |  |  |  | 2.62 |  | 3.1e-3 |  | 1.3e-1 |  | 0.129 |  |  |  |  |  |  |  |  |  |  |  |  |  |  |  | 0.47 |  | 1 |  | Sequest HT (3) |  | 3 |  | 411.26392 |  | -0.06 |  | 33.7336 |  | 8067 |  | 8067 |  | TRINITY\_DN49\_c10\_g1\_i1.p1; TRINITY\_DN0\_c1\_g1\_i22.p1; TRINITY\_DN591\_c1\_g1\_i1.p1; TRINITY\_DN0\_c1\_g1\_i7.p1 |  | Image |  | Peak List |  |
|  | | | | | | | | | | | | | | | | | | | | | | | | | | | | | | | | | | | | | | | | | | | | | | | | | | | | | |  |
|  | High |  | [K].TVAKEAGKTGLELMAcKITNQc.[-] |  | HCD |  | C16(Dehydro); C22(Dehydro) |  | 3.04 |  | 1.3e-3 |  | 6.0e-2 |  | 0.298 |  | 212.25 |  | 1.000 |  |  |  | C16(Dehydro): 100; C22(Dehydro): 100 |  |  |  |  |  |  |  | 0.48 |  | 1 |  | Sequest HT (3) |  | 3 |  | 769.72150 |  | -0.26 |  | 34.1563 |  | 8257 |  | 8257 |  | TRINITY\_DN0\_c1\_g1\_i22.p1; TRINITY\_DN0\_c1\_g1\_i7.p1 |  | Image |  | Peak List |  |
|  | | | | | | | | | | | | | | | | | | | | | | | | | | | | | | | | | | | | | | | | | | | | | | | | | | | | | |  |
|  | High |  | [G].KTGLELMAcKITNQc.[-] |  | HCD |  | C9(Dehydro); C15(Dehydro) |  | 2.62 |  | 1.3e-3 |  | 7.4e-3 |  | 0.756 |  | 212.07 |  | 1.000 |  |  |  | C9(Dehydro): 100; C15(Dehydro): 100 |  |  |  |  |  |  |  | 0.54 |  | 1 |  | Sequest HT (3) |  | 2 |  | 825.90393 |  | -0.37 |  | 35.3409 |  | 8797 |  | 8797 |  | TRINITY\_DN0\_c1\_g1\_i22.p1; TRINITY\_DN591\_c1\_g1\_i1.p1; TRINITY\_DN0\_c1\_g1\_i7.p1 |  | Image |  | Peak List |  |
|  | | | | | | | | | | | | | | | | | | | | | | | | | | | | | | | | | | | | | | | | | | | | | | | | | | | | | |  |
|  | High |  | [A].GKTGLELMAcKITNQc.[-] |  | HCD |  | C10(Dehydro); C16(Dehydro) |  | 3.29 |  | 1.3e-3 |  | 3.0e-2 |  | 0.448 |  | 243.54 |  | 1.000 |  |  |  | C10(Dehydro): 100; C16(Dehydro): 100 |  |  |  |  |  |  |  | 0.57 |  | 1 |  | Sequest HT (3) |  | 2 |  | 854.41400 |  | -1.13 |  | 35.9242 |  | 9076 |  | 9076 |  | TRINITY\_DN0\_c1\_g1\_i22.p1; TRINITY\_DN591\_c1\_g1\_i1.p1; TRINITY\_DN0\_c1\_g1\_i7.p1 |  | Image |  | Peak List |  |
|  | | | | | | | | | | | | | | | | | | | | | | | | | | | | | | | | | | | | | | | | | | | | | | | | | | | | | |  |
|  | High |  | [G].KTGLELMAcKITnQc.[-] |  | HCD |  | C9(Dehydro); N13(Deamidated); C15(Dehydro) |  | 1.85 |  | 1.3e-3 |  | 5.8e-2 |  | 0.306 |  | 115.30 |  | 1.000 |  |  |  | C9(Dehydro): 100; N13(Deamidated): 100; C15(Dehydro): 100 |  |  |  |  |  |  |  | 0.38 |  | 1 |  | Sequest HT (3) |  | 2 |  | 826.39581 |  | -0.52 |  | 35.9931 |  | 9104 |  | 9104 |  | TRINITY\_DN0\_c1\_g1\_i22.p1; TRINITY\_DN591\_c1\_g1\_i1.p1; TRINITY\_DN0\_c1\_g1\_i7.p1 |  | Image |  | Peak List |  |
|  | | | | | | | | | | | | | | | | | | | | | | | | | | | | | | | | | | | | | | | | | | | | | | | | | | | | | |  |
|  | High |  | [R].GLFTLIKGAAKLIGK.[T] |  | HCD |  |  |  | 3.29 |  | 1.3e-3 |  | 7.8e-3 |  | 0.747 |  |  |  |  |  |  |  |  |  |  |  |  |  |  |  | 0.45 |  | 1 |  | Sequest HT (3) |  | 3 |  | 510.66541 |  | 2.55 |  | 36.2787 |  | 9243 |  | 9243 |  | TRINITY\_DN0\_c1\_g1\_i22.p1; TRINITY\_DN591\_c1\_g1\_i1.p1; TRINITY\_DN0\_c1\_g1\_i7.p1 |  | Image |  | Peak List |  |
|  | | | | | | | | | | | | | | | | | | | | | | | | | | | | | | | | | | | | | | | | | | | | | | | | | | | | | |  |
|  | High |  | [R].GLFTLIKGAAKLIGK.[T] |  | HCD |  |  |  | 3.30 |  | 1.3e-3 |  | 2.8e-2 |  | 0.465 |  |  |  |  |  |  |  |  |  |  |  |  |  |  |  | 0.36 |  | 1 |  | Sequest HT (3) |  | 4 |  | 383.24945 |  | -1.17 |  | 36.3338 |  | 9267 |  | 9267 |  | TRINITY\_DN0\_c1\_g1\_i22.p1; TRINITY\_DN591\_c1\_g1\_i1.p1; TRINITY\_DN0\_c1\_g1\_i7.p1 |  | Image |  | Peak List |  |
|  | | | | | | | | | | | | | | | | | | | | | | | | | | | | | | | | | | | | | | | | | | | | | | | | | | | | | |  |
|  | High |  | [R].GLFTLIKGAAKLIGK.[T] |  | HCD |  |  |  | 3.45 |  | 1.3e-3 |  | 1.8e-3 |  | 1.06 |  |  |  |  |  |  |  |  |  |  |  |  |  |  |  | 0.45 |  | 1 |  | Sequest HT (3) |  | 2 |  | 765.49078 |  | -2.27 |  | 36.3720 |  | 9290 |  | 9290 |  | TRINITY\_DN0\_c1\_g1\_i22.p1; TRINITY\_DN591\_c1\_g1\_i1.p1; TRINITY\_DN0\_c1\_g1\_i7.p1 |  | Image |  | Peak List |  |
|  | | | | | | | | | | | | | | | | | | | | | | | | | | | | | | | | | | | | | | | | | | | | | | | | | | | | | |  |
|  | High |  | [R].GLFTLIKGAAKLIGK.[T] |  | HCD |  |  |  | 3.02 |  | 1.3e-3 |  | 6.5e-3 |  | 0.786 |  |  |  |  |  |  |  |  |  |  |  |  |  |  |  | 0.34 |  | 1 |  | Sequest HT (3) |  | 3 |  | 510.66354 |  | -1.10 |  | 36.4235 |  | 9316 |  | 9316 |  | TRINITY\_DN0\_c1\_g1\_i22.p1; TRINITY\_DN591\_c1\_g1\_i1.p1; TRINITY\_DN0\_c1\_g1\_i7.p1 |  | Image |  | Peak List |  |
|  | | | | | | | | | | | | | | | | | | | | | | | | | | | | | | | | | | | | | | | | | | | | | | | | | | | | | |  |
|  | High |  | [R].GLFTLIKGAAKLIGKT.[V] |  | HCD |  |  |  | 3.25 |  | 1.3e-3 |  | 4.3e-2 |  | 0.372 |  |  |  |  |  |  |  |  |  |  |  |  |  |  |  | 0.52 |  | 1 |  | Sequest HT (3) |  | 3 |  | 544.34528 |  | -2.55 |  | 36.4530 |  | 9330 |  | 9330 |  | TRINITY\_DN0\_c1\_g1\_i22.p1; TRINITY\_DN591\_c1\_g1\_i1.p1; TRINITY\_DN0\_c1\_g1\_i7.p1 |  | Image |  | Peak List |  |
|  | | | | | | | | | | | | | | | | | | | | | | | | | | | | | | | | | | | | | | | | | | | | | | | | | | | | | |  |
|  | High |  | [R].GLFTLIKGAAKLIGK.[T] |  | HCD |  |  |  | 3.13 |  | 1.3e-3 |  | 3.0e-2 |  | 0.448 |  |  |  |  |  |  |  |  |  |  |  |  |  |  |  | 0.44 |  | 1 |  | Sequest HT (3) |  | 4 |  | 383.24942 |  | -1.25 |  | 36.4840 |  | 9345 |  | 9345 |  | TRINITY\_DN0\_c1\_g1\_i22.p1; TRINITY\_DN591\_c1\_g1\_i1.p1; TRINITY\_DN0\_c1\_g1\_i7.p1 |  | Image |  | Peak List |  |
|  | | | | | | | | | | | | | | | | | | | | | | | | | | | | | | | | | | | | | | | | | | | | | | | | | | | | | |  |
|  | High |  | [R].GLFTLIKGAAKLIGKT.[V] |  | HCD |  |  |  | 3.29 |  | 1.3e-3 |  | 5.0e-2 |  | 0.339 |  |  |  |  |  |  |  |  |  |  |  |  |  |  |  | 0.64 |  | 1 |  | Sequest HT (3) |  | 4 |  | 408.51123 |  | -1.44 |  | 36.5046 |  | 9357 |  | 9357 |  | TRINITY\_DN0\_c1\_g1\_i22.p1; TRINITY\_DN591\_c1\_g1\_i1.p1; TRINITY\_DN0\_c1\_g1\_i7.p1 |  | Image |  | Peak List |  |
|  | | | | | | | | | | | | | | | | | | | | | | | | | | | | | | | | | | | | | | | | | | | | | | | | | | | | | |  |
|  | High |  | [R].GLFTLIKGAAKLIGKTVAK.[E] |  | HCD |  |  |  | 4.38 |  | 1.3e-3 |  | 1.7e-2 |  | 0.581 |  |  |  |  |  |  |  |  |  |  |  |  |  |  |  | 0.71 |  | 1 |  | Sequest HT (3) |  | 4 |  | 483.06158 |  | -0.74 |  | 36.5516 |  | 9381 |  | 9381 |  | TRINITY\_DN0\_c1\_g1\_i22.p1; TRINITY\_DN0\_c1\_g1\_i7.p1 |  | Image |  | Peak List |  |
|  | | | | | | | | | | | | | | | | | | | | | | | | | | | | | | | | | | | | | | | | | | | | | | | | | | | | | |  |
|  | High |  | [R].GLFTLIKGAAKLIGK.[T] |  | HCD |  |  |  | 1.05 |  | 5.6e-3 |  | 2.0e-1 |  | 0.017 |  |  |  |  |  |  |  |  |  |  |  |  |  |  |  | 0.38 |  | 1 |  | Sequest HT (3) |  | 3 |  | 510.66406 |  | -0.08 |  | 36.5663 |  | 9388 |  | 9388 |  | TRINITY\_DN0\_c1\_g1\_i22.p1; TRINITY\_DN591\_c1\_g1\_i1.p1; TRINITY\_DN0\_c1\_g1\_i7.p1 |  | Image |  | Peak List |  |
|  | | | | | | | | | | | | | | | | | | | | | | | | | | | | | | | | | | | | | | | | | | | | | | | | | | | | | |  |
|  | High |  | [R].GLFTLIKGAAKLIGKTVAK.[E] |  | HCD |  |  |  | 4.58 |  | 1.3e-3 |  | 3.2e-2 |  | 0.436 |  |  |  |  |  |  |  |  |  |  |  |  |  |  |  | 0.62 |  | 1 |  | Sequest HT (3) |  | 5 |  | 386.65057 |  | -1.12 |  | 36.5825 |  | 9398 |  | 9398 |  | TRINITY\_DN0\_c1\_g1\_i22.p1; TRINITY\_DN0\_c1\_g1\_i7.p1 |  | Image |  | Peak List |  |
|  | | | | | | | | | | | | | | | | | | | | | | | | | | | | | | | | | | | | | | | | | | | | | | | | | | | | | |  |
|  | High |  | [R].GLFTLIKGAAKLIGKTVAK.[E] |  | HCD |  |  |  | 4.59 |  | 1.3e-3 |  | 6.4e-5 |  | 1.79 |  |  |  |  |  |  |  |  |  |  |  |  |  |  |  | 0.83 |  | 1 |  | Sequest HT (3) |  | 2 |  | 965.11407 |  | -2.62 |  | 36.6352 |  | 9422 |  | 9422 |  | TRINITY\_DN0\_c1\_g1\_i22.p1; TRINITY\_DN0\_c1\_g1\_i7.p1 |  | Image |  | Peak List |  |
|  | | | | | | | | | | | | | | | | | | | | | | | | | | | | | | | | | | | | | | | | | | | | | | | | | | | | | |  |
|  | High |  | [R].GLFTLIKGAAKLIGKTVAK.[E] |  | HCD |  |  |  | 4.24 |  | 1.3e-3 |  | 7.9e-3 |  | 0.742 |  |  |  |  |  |  |  |  |  |  |  |  |  |  |  | 0.75 |  | 1 |  | Sequest HT (3) |  | 3 |  | 643.74628 |  | -0.86 |  | 36.6659 |  | 9434 |  | 9434 |  | TRINITY\_DN0\_c1\_g1\_i22.p1; TRINITY\_DN0\_c1\_g1\_i7.p1 |  | Image |  | Peak List |  |
|  | | | | | | | | | | | | | | | | | | | | | | | | | | | | | | | | | | | | | | | | | | | | | | | | | | | | | |  |
|  | High |  | [R].GLFTLIKGAAKLIGKTVAK.[E] |  | HCD |  |  |  | 2.76 |  | 1.3e-3 |  | 6.1e-2 |  | 0.294 |  |  |  |  |  |  |  |  |  |  |  |  |  |  |  | 0.62 |  | 1 |  | Sequest HT (3) |  | 4 |  | 483.06128 |  | -1.37 |  | 36.6929 |  | 9447 |  | 9447 |  | TRINITY\_DN0\_c1\_g1\_i22.p1; TRINITY\_DN0\_c1\_g1\_i7.p1 |  | Image |  | Peak List |  |
|  | | | | | | | | | | | | | | | | | | | | | | | | | | | | | | | | | | | | | | | | | | | | | | | | | | | | | |  |
|  | High |  | [R].GLFTLIKGAAKLIGKTVAK.[E] |  | HCD |  |  |  | 4.43 |  | 1.3e-3 |  | 2.0e-2 |  | 0.535 |  |  |  |  |  |  |  |  |  |  |  |  |  |  |  | 0.63 |  | 1 |  | Sequest HT (3) |  | 5 |  | 386.65033 |  | -1.76 |  | 36.7298 |  | 9467 |  | 9467 |  | TRINITY\_DN0\_c1\_g1\_i22.p1; TRINITY\_DN0\_c1\_g1\_i7.p1 |  | Image |  | Peak List |  |
|  | | | | | | | | | | | | | | | | | | | | | | | | | | | | | | | | | | | | | | | | | | | | | | | | | | | | | |  |
|  | High |  | [R].GLFTLIKGAAKLIGKTVAK.[E] |  | HCD |  |  |  | 5.08 |  | 1.3e-3 |  | 2.3e-4 |  | 1.52 |  |  |  |  |  |  |  |  |  |  |  |  |  |  |  | 0.77 |  | 1 |  | Sequest HT (3) |  | 3 |  | 643.74561 |  | -1.90 |  | 36.8039 |  | 9502 |  | 9502 |  | TRINITY\_DN0\_c1\_g1\_i22.p1; TRINITY\_DN0\_c1\_g1\_i7.p1 |  | Image |  | Peak List |  |
|  | | | | | | | | | | | | | | | | | | | | | | | | | | | | | | | | | | | | | | | | | | | | | | | | | | | | | |  |
|  | High |  | [R].GLFTLIKGAAKLIGKTVAK.[E] |  | HCD |  |  |  | 3.99 |  | 1.3e-3 |  | 9.6e-4 |  | 1.2 |  |  |  |  |  |  |  |  |  |  |  |  |  |  |  | 0.70 |  | 1 |  | Sequest HT (3) |  | 4 |  | 483.06140 |  | -1.12 |  | 36.8399 |  | 9517 |  | 9517 |  | TRINITY\_DN0\_c1\_g1\_i22.p1; TRINITY\_DN0\_c1\_g1\_i7.p1 |  | Image |  | Peak List |  |
|  | | | | | | | | | | | | | | | | | | | | | | | | | | | | | | | | | | | | | | | | | | | | | | | | | | | | | |  |
|  | High |  | [R].GLFTLIKGAAKLIGKTVAKEAGK.[T] |  | HCD |  |  |  | 7.99 |  | 1.3e-3 |  | 4.5e-4 |  | 1.36 |  |  |  |  |  |  |  |  |  |  |  |  |  |  |  | 0.60 |  | 1 |  | Sequest HT (3) |  | 4 |  | 579.36249 |  | 2.62 |  | 37.2147 |  | 9696 |  | 9696 |  | TRINITY\_DN0\_c1\_g1\_i22.p1; TRINITY\_DN0\_c1\_g1\_i7.p1 |  | Image |  | Peak List |  |
|  | | | | | | | | | | | | | | | | | | | | | | | | | | | | | | | | | | | | | | | | | | | | | | | | | | | | | |  |
|  | High |  | [R].GLFTLIKGAAKLIGKTVAKEAGK.[T] |  | HCD |  |  |  | 6.85 |  | 1.3e-3 |  | 2.7e-4 |  | 1.48 |  |  |  |  |  |  |  |  |  |  |  |  |  |  |  | 0.70 |  | 1 |  | Sequest HT (3) |  | 5 |  | 463.69095 |  | 1.54 |  | 37.2432 |  | 9709 |  | 9709 |  | TRINITY\_DN0\_c1\_g1\_i22.p1; TRINITY\_DN0\_c1\_g1\_i7.p1 |  | Image |  | Peak List |  |
|  | | | | | | | | | | | | | | | | | | | | | | | | | | | | | | | | | | | | | | | | | | | | | | | | | | | | | |  |
|  | High |  | [R].GLFTLIKGAAKLIGKTVAKEAGK.[T] |  | HCD |  |  |  | 7.68 |  | 1.3e-3 |  | 1.2e-5 |  | 2.16 |  |  |  |  |  |  |  |  |  |  |  |  |  |  |  | 0.66 |  | 1 |  | Sequest HT (3) |  | 3 |  | 772.14392 |  | -2.10 |  | 37.2525 |  | 9716 |  | 9716 |  | TRINITY\_DN0\_c1\_g1\_i22.p1; TRINITY\_DN0\_c1\_g1\_i7.p1 |  | Image |  | Peak List |  |
|  | | | | | | | | | | | | | | | | | | | | | | | | | | | | | | | | | | | | | | | | | | | | | | | | | | | | | |  |
|  | High |  | [R].GLFTLIKGAAKLIGKTVAKEAGK.[T] |  | HCD |  |  |  | 5.80 |  | 1.3e-3 |  | 9.3e-7 |  | 2.71 |  |  |  |  |  |  |  |  |  |  |  |  |  |  |  | 0.64 |  | 1 |  | Sequest HT (3) |  | 2 |  | 1157.71277 |  | -1.64 |  | 37.2981 |  | 9739 |  | 9739 |  | TRINITY\_DN0\_c1\_g1\_i22.p1; TRINITY\_DN0\_c1\_g1\_i7.p1 |  | Image |  | Peak List |  |
|  | | | | | | | | | | | | | | | | | | | | | | | | | | | | | | | | | | | | | | | | | | | | | | | | | | | | | |  |
|  | High |  | [R].GLFTLIKGAAKLIGKTVAKE.[A] |  | HCD |  |  |  | 4.62 |  | 1.3e-3 |  | 6.3e-3 |  | 0.791 |  |  |  |  |  |  |  |  |  |  |  |  |  |  |  | 0.60 |  | 1 |  | Sequest HT (3) |  | 4 |  | 515.32257 |  | -0.03 |  | 37.3345 |  | 9757 |  | 9757 |  | TRINITY\_DN0\_c1\_g1\_i22.p1; TRINITY\_DN0\_c1\_g1\_i7.p1 |  | Image |  | Peak List |  |
|  | | | | | | | | | | | | | | | | | | | | | | | | | | | | | | | | | | | | | | | | | | | | | | | | | | | | | |  |
|  | High |  | [R].GLFTLIKGAAKLIGKTVAKEAGKTG.[L] |  | HCD |  |  |  | 5.76 |  | 1.3e-3 |  | 4.2e-3 |  | 0.882 |  |  |  |  |  |  |  |  |  |  |  |  |  |  |  | 0.59 |  | 1 |  | Sequest HT (3) |  | 5 |  | 495.30423 |  | 0.34 |  | 37.3398 |  | 9761 |  | 9761 |  | TRINITY\_DN0\_c1\_g1\_i22.p1; TRINITY\_DN0\_c1\_g1\_i7.p1 |  | Image |  | Peak List |  |
|  | | | | | | | | | | | | | | | | | | | | | | | | | | | | | | | | | | | | | | | | | | | | | | | | | | | | | |  |
|  | High |  | [R].GLFTLIKGAAKLIGKTVAKE.[A] |  | HCD |  |  |  | 6.34 |  | 1.3e-3 |  | 1.3e-4 |  | 1.65 |  |  |  |  |  |  |  |  |  |  |  |  |  |  |  | 0.76 |  | 1 |  | Sequest HT (3) |  | 3 |  | 686.76074 |  | -0.41 |  | 37.3537 |  | 9768 |  | 9768 |  | TRINITY\_DN0\_c1\_g1\_i22.p1; TRINITY\_DN0\_c1\_g1\_i7.p1 |  | Image |  | Peak List |  |
|  | | | | | | | | | | | | | | | | | | | | | | | | | | | | | | | | | | | | | | | | | | | | | | | | | | | | | |  |
|  | High |  | [R].GLFTLIKGAAKLIGKTVAKEAGK.[T] |  | HCD |  |  |  | 4.96 |  | 1.3e-3 |  | 1.5e-3 |  | 1.1 |  |  |  |  |  |  |  |  |  |  |  |  |  |  |  | 0.58 |  | 1 |  | Sequest HT (3) |  | 4 |  | 579.36084 |  | -0.23 |  | 37.3601 |  | 9770 |  | 9770 |  | TRINITY\_DN0\_c1\_g1\_i22.p1; TRINITY\_DN0\_c1\_g1\_i7.p1 |  | Image |  | Peak List |  |
|  | | | | | | | | | | | | | | | | | | | | | | | | | | | | | | | | | | | | | | | | | | | | | | | | | | | | | |  |
|  | High |  | [R].GLFTLIKGAAKLIGKTVAKE.[A] |  | HCD |  |  |  | 4.20 |  | 3.1e-3 |  | 1.4e-1 |  | 0.103 |  |  |  |  |  |  |  |  |  |  |  |  |  |  |  | 0.45 |  | 1 |  | Sequest HT (3) |  | 5 |  | 412.45950 |  | -0.06 |  | 37.3779 |  | 9781 |  | 9781 |  | TRINITY\_DN0\_c1\_g1\_i22.p1; TRINITY\_DN0\_c1\_g1\_i7.p1 |  | Image |  | Peak List |  |
|  | | | | | | | | | | | | | | | | | | | | | | | | | | | | | | | | | | | | | | | | | | | | | | | | | | | | | |  |
|  | High |  | [R].GLFTLIKGAAKLIGKTVAKEAGK.[T] |  | HCD |  |  |  | 4.08 |  | 1.3e-3 |  | 5.4e-3 |  | 0.827 |  |  |  |  |  |  |  |  |  |  |  |  |  |  |  | 0.59 |  | 1 |  | Sequest HT (3) |  | 5 |  | 463.69000 |  | -0.50 |  | 37.3856 |  | 9784 |  | 9784 |  | TRINITY\_DN0\_c1\_g1\_i22.p1; TRINITY\_DN0\_c1\_g1\_i7.p1 |  | Image |  | Peak List |  |
|  | | | | | | | | | | | | | | | | | | | | | | | | | | | | | | | | | | | | | | | | | | | | | | | | | | | | | |  |
|  | High |  | [R].GLFTLIKGAAKLIGKTVAKEAGKTG.[L] |  | HCD |  |  |  | 5.82 |  | 1.3e-3 |  | 1.4e-3 |  | 1.12 |  |  |  |  |  |  |  |  |  |  |  |  |  |  |  | 0.64 |  | 1 |  | Sequest HT (3) |  | 4 |  | 618.87781 |  | -0.73 |  | 37.3945 |  | 9788 |  | 9788 |  | TRINITY\_DN0\_c1\_g1\_i22.p1; TRINITY\_DN0\_c1\_g1\_i7.p1 |  | Image |  | Peak List |  |
|  | | | | | | | | | | | | | | | | | | | | | | | | | | | | | | | | | | | | | | | | | | | | | | | | | | | | | |  |
|  | High |  | [R].GLFTLIKGAAKLIGKTVAKEAGK.[T] |  | HCD |  |  |  | 5.09 |  | 1.3e-3 |  | 5.1e-4 |  | 1.34 |  |  |  |  |  |  |  |  |  |  |  |  |  |  |  | 0.72 |  | 1 |  | Sequest HT (3) |  | 3 |  | 772.14587 |  | 0.43 |  | 37.3958 |  | 9789 |  | 9789 |  | TRINITY\_DN0\_c1\_g1\_i22.p1; TRINITY\_DN0\_c1\_g1\_i7.p1 |  | Image |  | Peak List |  |
|  | | | | | | | | | | | | | | | | | | | | | | | | | | | | | | | | | | | | | | | | | | | | | | | | | | | | | |  |
|  | High |  | [R].GLFTLIKGAAKLIGKTVAKEAGKT.[G] |  | HCD |  |  |  | 5.91 |  | 1.3e-3 |  | 2.3e-3 |  | 1.01 |  |  |  |  |  |  |  |  |  |  |  |  |  |  |  | 0.53 |  | 1 |  | Sequest HT (3) |  | 4 |  | 604.62292 |  | 0.05 |  | 37.3971 |  | 9790 |  | 9790 |  | TRINITY\_DN0\_c1\_g1\_i22.p1; TRINITY\_DN0\_c1\_g1\_i7.p1 |  | Image |  | Peak List |  |
|  | | | | | | | | | | | | | | | | | | | | | | | | | | | | | | | | | | | | | | | | | | | | | | | | | | | | | |  |
|  | High |  | [R].GLFTLIKGAAKLIGKTVAKE.[A] |  | HCD |  |  |  | 5.21 |  | 1.3e-3 |  | 1.5e-5 |  | 2.11 |  |  |  |  |  |  |  |  |  |  |  |  |  |  |  | 0.77 |  | 1 |  | Sequest HT (3) |  | 2 |  | 1029.63599 |  | -1.86 |  | 37.4021 |  | 9794 |  | 9794 |  | TRINITY\_DN0\_c1\_g1\_i22.p1; TRINITY\_DN0\_c1\_g1\_i7.p1 |  | Image |  | Peak List |  |
|  | | | | | | | | | | | | | | | | | | | | | | | | | | | | | | | | | | | | | | | | | | | | | | | | | | | | | |  |
|  | High |  | [R].GLFTLIKGAAKLIGKTVAKEAGKT.[G] |  | HCD |  |  |  | 6.94 |  | 1.3e-3 |  | 1.4e-4 |  | 1.62 |  |  |  |  |  |  |  |  |  |  |  |  |  |  |  | 0.72 |  | 1 |  | Sequest HT (3) |  | 3 |  | 805.82727 |  | -1.03 |  | 37.4060 |  | 9797 |  | 9797 |  | TRINITY\_DN0\_c1\_g1\_i22.p1; TRINITY\_DN0\_c1\_g1\_i7.p1 |  | Image |  | Peak List |  |
|  | | | | | | | | | | | | | | | | | | | | | | | | | | | | | | | | | | | | | | | | | | | | | | | | | | | | | |  |
|  | High |  | [R].GLFTLIKGAAKLIGKTVAKEAGKTG.[L] |  | HCD |  |  |  | 3.61 |  | 1.3e-3 |  | 1.7e-2 |  | 0.579 |  |  |  |  |  |  |  |  |  |  |  |  |  |  |  | 0.46 |  | 1 |  | Sequest HT (3) |  | 5 |  | 495.30389 |  | -0.34 |  | 37.4850 |  | 9838 |  | 9838 |  | TRINITY\_DN0\_c1\_g1\_i22.p1; TRINITY\_DN0\_c1\_g1\_i7.p1 |  | Image |  | Peak List |  |
|  | | | | | | | | | | | | | | | | | | | | | | | | | | | | | | | | | | | | | | | | | | | | | | | | | | | | | |  |
|  | High |  | [R].GLFTLIKGAAKLIGKTVAKE.[A] |  | HCD |  |  |  | 3.38 |  | 1.3e-3 |  | 2.2e-2 |  | 0.517 |  |  |  |  |  |  |  |  |  |  |  |  |  |  |  | 0.67 |  | 1 |  | Sequest HT (3) |  | 4 |  | 515.32257 |  | -0.03 |  | 37.4862 |  | 9839 |  | 9839 |  | TRINITY\_DN0\_c1\_g1\_i22.p1; TRINITY\_DN0\_c1\_g1\_i7.p1 |  | Image |  | Peak List |  |
|  | | | | | | | | | | | | | | | | | | | | | | | | | | | | | | | | | | | | | | | | | | | | | | | | | | | | | |  |
|  | High |  | [R].GLFTLIKGAAKLIGKTVAKE.[A] |  | HCD |  |  |  | 4.47 |  | 1.3e-3 |  | 2.0e-2 |  | 0.537 |  |  |  |  |  |  |  |  |  |  |  |  |  |  |  | 0.69 |  | 1 |  | Sequest HT (3) |  | 3 |  | 686.76099 |  | -0.06 |  | 37.4953 |  | 9843 |  | 9843 |  | TRINITY\_DN0\_c1\_g1\_i22.p1; TRINITY\_DN0\_c1\_g1\_i7.p1 |  | Image |  | Peak List |  |
|  | | | | | | | | | | | | | | | | | | | | | | | | | | | | | | | | | | | | | | | | | | | | | | | | | | | | | |  |
|  | High |  | [R].GLFTLIKGAAKLIGKTVAKEAGKT.[G] |  | HCD |  |  |  | 2.88 |  | 1.3e-3 |  | 1.1e-2 |  | 0.675 |  |  |  |  |  |  |  |  |  |  |  |  |  |  |  | 0.42 |  | 1 |  | Sequest HT (3) |  | 5 |  | 483.90002 |  | 0.53 |  | 37.5259 |  | 9859 |  | 9859 |  | TRINITY\_DN0\_c1\_g1\_i22.p1; TRINITY\_DN0\_c1\_g1\_i7.p1 |  | Image |  | Peak List |  |
|  | | | | | | | | | | | | | | | | | | | | | | | | | | | | | | | | | | | | | | | | | | | | | | | | | | | | | |  |
|  | High |  | [R].GLFTLIKGAAKLIGKTVAKEAGKTG.[L] |  | HCD |  |  |  | 5.02 |  | 1.3e-3 |  | 2.8e-3 |  | 0.966 |  |  |  |  |  |  |  |  |  |  |  |  |  |  |  | 0.64 |  | 1 |  | Sequest HT (3) |  | 4 |  | 618.87750 |  | -1.22 |  | 37.5400 |  | 9867 |  | 9867 |  | TRINITY\_DN0\_c1\_g1\_i22.p1; TRINITY\_DN0\_c1\_g1\_i7.p1 |  | Image |  | Peak List |  |
|  | | | | | | | | | | | | | | | | | | | | | | | | | | | | | | | | | | | | | | | | | | | | | | | | | | | | | |  |
|  | High |  | [R].GLFTLIKGAAKLIGKTVAKEAGKT.[G] |  | HCD |  |  |  | 3.33 |  | 1.3e-3 |  | 5.4e-3 |  | 0.824 |  |  |  |  |  |  |  |  |  |  |  |  |  |  |  | 0.56 |  | 1 |  | Sequest HT (3) |  | 4 |  | 604.62250 |  | -0.65 |  | 37.5412 |  | 9868 |  | 9868 |  | TRINITY\_DN0\_c1\_g1\_i22.p1; TRINITY\_DN0\_c1\_g1\_i7.p1 |  | Image |  | Peak List |  |
|  | | | | | | | | | | | | | | | | | | | | | | | | | | | | | | | | | | | | | | | | | | | | | | | | | | | | | |  |
|  | High |  | [R].GLFTLIKGAAKLIGKTVAKEA.[G] |  | HCD |  |  |  | 4.25 |  | 1.3e-3 |  | 2.4e-2 |  | 0.496 |  |  |  |  |  |  |  |  |  |  |  |  |  |  |  | 0.55 |  | 1 |  | Sequest HT (3) |  | 4 |  | 533.08173 |  | -0.26 |  | 38.0509 |  | 10101 |  | 10101 |  | TRINITY\_DN0\_c1\_g1\_i22.p1; TRINITY\_DN0\_c1\_g1\_i7.p1 |  | Image |  | Peak List |  |
|  | | | | | | | | | | | | | | | | | | | | | | | | | | | | | | | | | | | | | | | | | | | | | | | | | | | | | |  |
|  | High |  | [R].GLFTLIKGAAKLIGKTVAKEA.[G] |  | HCD |  |  |  | 4.99 |  | 1.3e-3 |  | 5.1e-3 |  | 0.839 |  |  |  |  |  |  |  |  |  |  |  |  |  |  |  | 0.68 |  | 1 |  | Sequest HT (3) |  | 3 |  | 710.43872 |  | -1.89 |  | 38.0665 |  | 10110 |  | 10110 |  | TRINITY\_DN0\_c1\_g1\_i22.p1; TRINITY\_DN0\_c1\_g1\_i7.p1 |  | Image |  | Peak List |  |
|  | | | | | | | | | | | | | | | | | | | | | | | | | | | | | | | | | | | | | | | | | | | | | | | | | | | | | |  |
|  | High |  | [R].GLFTLIKGAAKLIGKTVAKEA.[G] |  | HCD |  |  |  | 5.74 |  | 1.3e-3 |  | 5.5e-6 |  | 2.33 |  |  |  |  |  |  |  |  |  |  |  |  |  |  |  | 0.67 |  | 1 |  | Sequest HT (3) |  | 2 |  | 1065.15540 |  | -1.00 |  | 38.0910 |  | 10126 |  | 10126 |  | TRINITY\_DN0\_c1\_g1\_i22.p1; TRINITY\_DN0\_c1\_g1\_i7.p1 |  | Image |  | Peak List |  |
|  | | | | | | | | | | | | | | | | | | | | | | | | | | | | | | | | | | | | | | | | | | | | | | | | | | | | | |  |
|  | High |  | [R].GLFTLIKGAAKLIGKTVAKEA.[G] |  | HCD |  |  |  | 4.64 |  | 3.1e-3 |  | 9.7e-2 |  | 0.19 |  |  |  |  |  |  |  |  |  |  |  |  |  |  |  | 0.53 |  | 1 |  | Sequest HT (3) |  | 5 |  | 426.66663 |  | -0.76 |  | 38.1000 |  | 10130 |  | 10130 |  | TRINITY\_DN0\_c1\_g1\_i22.p1; TRINITY\_DN0\_c1\_g1\_i7.p1 |  | Image |  | Peak List |  |
|  | | | | | | | | | | | | | | | | | | | | | | | | | | | | | | | | | | | | | | | | | | | | | | | | | | | | | |  |
|  | High |  | [R].GLFTLIKGAAKLIGKTVAKEA.[G] |  | HCD |  |  |  | 2.14 |  | 3.1e-3 |  | 1.4e-1 |  | 0.112 |  |  |  |  |  |  |  |  |  |  |  |  |  |  |  | 0.49 |  | 1 |  | Sequest HT (3) |  | 4 |  | 533.08136 |  | -0.95 |  | 38.2007 |  | 10183 |  | 10183 |  | TRINITY\_DN0\_c1\_g1\_i22.p1; TRINITY\_DN0\_c1\_g1\_i7.p1 |  | Image |  | Peak List |  |
|  | | | | | | | | | | | | | | | | | | | | | | | | | | | | | | | | | | | | | | | | | | | | | | | | | | | | | |  |
|  | High |  | [R].GLFTLIKGAAKLIGKTVAKEA.[G] |  | HCD |  |  |  | 3.01 |  | 3.1e-3 |  | 1.0e-1 |  | 0.183 |  |  |  |  |  |  |  |  |  |  |  |  |  |  |  | 0.63 |  | 1 |  | Sequest HT (3) |  | 3 |  | 710.43921 |  | -1.20 |  | 38.2114 |  | 10188 |  | 10188 |  | TRINITY\_DN0\_c1\_g1\_i22.p1; TRINITY\_DN0\_c1\_g1\_i7.p1 |  | Image |  | Peak List |  |
|  | | | | | | | | | | | | | | | | | | | | | | | | | | | | | | | | | | | | | | | | | | | | | | | | | | | | | |  |
|  | High |  | [R].GLFTLIKGAAKLIG.[K] |  | HCD |  |  |  | 2.22 |  | 6.5e-3 |  | 2.2e-1 |  | 0 |  |  |  |  |  |  |  |  |  |  |  |  |  |  |  | 0.18 |  | 1 |  | Sequest HT (3) |  | 3 |  | 467.96567 |  | -0.25 |  | 38.2690 |  | 10216 |  | 10216 |  | TRINITY\_DN0\_c1\_g1\_i22.p1; TRINITY\_DN591\_c1\_g1\_i1.p1; TRINITY\_DN0\_c1\_g1\_i7.p1 |  | Image |  | Peak List |  |
|  | | | | | | | | | | | | | | | | | | | | | | | | | | | | | | | | | | | | | | | | | | | | | | | | | | | | | |  |
|  | High |  | [R].GLFTLIKGAAKLIGKTVAKEAG.[QK] |  | HCD |  |  |  | 6.06 |  | 1.3e-3 |  | 1.0e-3 |  | 1.19 |  |  |  |  |  |  |  |  |  |  |  |  |  |  |  | 0.61 |  | 1 |  | Sequest HT (3) |  | 3 |  | 729.44696 |  | -0.35 |  | 38.2832 |  | 10223 |  | 10223 |  | TRINITY\_DN0\_c1\_g1\_i22.p1; TRINITY\_DN0\_c1\_g1\_i7.p1 |  | Image |  | Peak List |  |
|  | | | | | | | | | | | | | | | | | | | | | | | | | | | | | | | | | | | | | | | | | | | | | | | | | | | | | |  |
|  | High |  | [R].GLFTLIKGAAKLIG.[K] |  | HCD |  |  |  | 3.02 |  | 1.3e-3 |  | 1.2e-2 |  | 0.646 |  |  |  |  |  |  |  |  |  |  |  |  |  |  |  | 0.49 |  | 1 |  | Sequest HT (3) |  | 2 |  | 701.44470 |  | -0.48 |  | 38.2968 |  | 10230 |  | 10230 |  | TRINITY\_DN0\_c1\_g1\_i22.p1; TRINITY\_DN591\_c1\_g1\_i1.p1; TRINITY\_DN0\_c1\_g1\_i7.p1 |  | Image |  | Peak List |  |
|  | | | | | | | | | | | | | | | | | | | | | | | | | | | | | | | | | | | | | | | | | | | | | | | | | | | | | |  |
|  | High |  | [R].GLFTLIKGAAKLIGKTVAKEAG.[QK] |  | HCD |  |  |  | 3.30 |  | 1.3e-3 |  | 3.7e-2 |  | 0.402 |  |  |  |  |  |  |  |  |  |  |  |  |  |  |  | 0.53 |  | 1 |  | Sequest HT (3) |  | 4 |  | 547.33698 |  | -0.47 |  | 38.3279 |  | 10245 |  | 10245 |  | TRINITY\_DN0\_c1\_g1\_i22.p1; TRINITY\_DN0\_c1\_g1\_i7.p1 |  | Image |  | Peak List |  |
|  | | | | | | | | | | | | | | | | | | | | | | | | | | | | | | | | | | | | | | | | | | | | | | | | | | | | | |  |
|  | High |  | [R].GLFTLIKGAAKLIGKTVAKEAG.[QK] |  | HCD |  |  |  | 3.03 |  | 1.3e-3 |  | 5.0e-2 |  | 0.34 |  |  |  |  |  |  |  |  |  |  |  |  |  |  |  | 0.50 |  | 1 |  | Sequest HT (3) |  | 5 |  | 438.07108 |  | -0.38 |  | 38.3917 |  | 10278 |  | 10278 |  | TRINITY\_DN0\_c1\_g1\_i22.p1; TRINITY\_DN0\_c1\_g1\_i7.p1 |  | Image |  | Peak List |  |
|  | | | | | | | | | | | | | | | | | | | | | | | | | | | | | | | | | | | | | | | | | | | | | | | | | | | | | |  |
|  | High |  | [R].GLFTLIKGAAKLIGKTVAKEAG.[QK] |  | HCD |  |  |  | 4.36 |  | 1.3e-3 |  | 2.8e-4 |  | 1.47 |  |  |  |  |  |  |  |  |  |  |  |  |  |  |  | 0.49 |  | 1 |  | Sequest HT (3) |  | 2 |  | 1093.66577 |  | -1.30 |  | 38.4084 |  | 10288 |  | 10288 |  | TRINITY\_DN0\_c1\_g1\_i22.p1; TRINITY\_DN0\_c1\_g1\_i7.p1 |  | Image |  | Peak List |  |
|  | | | | | | | | | | | | | | | | | | | | | | | | | | | | | | | | | | | | | | | | | | | | | | | | | | | | | |  |
|  | High |  | [R].GLFTLIKGAAKLIG.[K] |  | HCD |  |  |  | 2.00 |  | 1.3e-3 |  | 4.9e-2 |  | 0.342 |  |  |  |  |  |  |  |  |  |  |  |  |  |  |  | 0.30 |  | 1 |  | Sequest HT (3) |  | 2 |  | 701.44458 |  | -0.65 |  | 38.4422 |  | 10305 |  | 10305 |  | TRINITY\_DN0\_c1\_g1\_i22.p1; TRINITY\_DN591\_c1\_g1\_i1.p1; TRINITY\_DN0\_c1\_g1\_i7.p1 |  | Image |  | Peak List |  |
|  | | | | | | | | | | | | | | | | | | | | | | | | | | | | | | | | | | | | | | | | | | | | | | | | | | | | | |  |
|  | High |  | [R].GLFTLIKGAAKLIGKTVAKEAG.[QK] |  | HCD |  |  |  | 2.78 |  | 3.1e-3 |  | 1.3e-1 |  | 0.115 |  |  |  |  |  |  |  |  |  |  |  |  |  |  |  | 0.55 |  | 1 |  | Sequest HT (3) |  | 4 |  | 547.33740 |  | 0.31 |  | 38.4700 |  | 10319 |  | 10319 |  | TRINITY\_DN0\_c1\_g1\_i22.p1; TRINITY\_DN0\_c1\_g1\_i7.p1 |  | Image |  | Peak List |  |
|  | | | | | | | | | | | | | | | | | | | | | | | | | | | | | | | | | | | | | | | | | | | | | | | | | | | | | |  |
|  | High |  | [R].GLFTLIKGAAKLIGKTVAKEAG.[QK] |  | HCD |  |  |  | 2.32 |  | 1.3e-3 |  | 3.1e-2 |  | 0.446 |  |  |  |  |  |  |  |  |  |  |  |  |  |  |  | 0.44 |  | 1 |  | Sequest HT (3) |  | 5 |  | 438.07129 |  | 0.11 |  | 38.5422 |  | 10350 |  | 10350 |  | TRINITY\_DN0\_c1\_g1\_i22.p1; TRINITY\_DN0\_c1\_g1\_i7.p1 |  | Image |  | Peak List |  |
|  | | | | | | | | | | | | | | | | | | | | | | | | | | | | | | | | | | | | | | | | | | | | | | | | | | | | | |  |
|  | High |  | [R].GLFTLIKGAAKLIGKTVAKEAG.[QK] |  | HCD |  |  |  | 5.03 |  | 1.3e-3 |  | 7.5e-6 |  | 2.26 |  |  |  |  |  |  |  |  |  |  |  |  |  |  |  | 0.57 |  | 1 |  | Sequest HT (3) |  | 2 |  | 1093.66699 |  | -0.18 |  | 38.5511 |  | 10354 |  | 10354 |  | TRINITY\_DN0\_c1\_g1\_i22.p1; TRINITY\_DN0\_c1\_g1\_i7.p1 |  | Image |  | Peak List |  |
|  | | | | | | | | | | | | | | | | | | | | | | | | | | | | | | | | | | | | | | | | | | | | | | | | | | | | | |  |
|  | High |  | [R].GLFTLIKGAAKLIGKTVAKEAG.[QK] |  | HCD |  |  |  | 3.70 |  | 1.3e-3 |  | 7.8e-3 |  | 0.745 |  |  |  |  |  |  |  |  |  |  |  |  |  |  |  | 0.53 |  | 1 |  | Sequest HT (3) |  | 3 |  | 729.44818 |  | 1.32 |  | 38.5899 |  | 10373 |  | 10373 |  | TRINITY\_DN0\_c1\_g1\_i22.p1; TRINITY\_DN0\_c1\_g1\_i7.p1 |  | Image |  | Peak List |  |
|  | | | | | | | | | | | | | | | | | | | | | | | | | | | | | | | | | | | | | | | | | | | | | | | | | | | | | |  |
|  | High |  | [R].GLFTLIKGAAKLIGKTVAKEAG.[QK] |  | HCD |  |  |  | 2.83 |  | 2.4e-3 |  | 7.3e-2 |  | 0.253 |  |  |  |  |  |  |  |  |  |  |  |  |  |  |  | 0.56 |  | 1 |  | Sequest HT (3) |  | 4 |  | 547.33881 |  | 2.88 |  | 38.6113 |  | 10383 |  | 10383 |  | TRINITY\_DN0\_c1\_g1\_i22.p1; TRINITY\_DN0\_c1\_g1\_i7.p1 |  | Image |  | Peak List |  |
|  | | | | | | | | | | | | | | | | | | | | | | | | | | | | | | | | | | | | | | | | | | | | | | | | | | | | | |  |
|  | High |  | [R].GLFTLIKGAAKLIGKTVAKEAGKTGLE.[L] |  | HCD |  |  |  | 6.12 |  | 1.3e-3 |  | 7.1e-5 |  | 1.77 |  |  |  |  |  |  |  |  |  |  |  |  |  |  |  | 0.63 |  | 1 |  | Sequest HT (3) |  | 3 |  | 905.54633 |  | 2.42 |  | 38.7055 |  | 10429 |  | 10429 |  | TRINITY\_DN0\_c1\_g1\_i22.p1; TRINITY\_DN0\_c1\_g1\_i7.p1 |  | Image |  | Peak List |  |
|  | | | | | | | | | | | | | | | | | | | | | | | | | | | | | | | | | | | | | | | | | | | | | | | | | | | | | |  |
|  | High |  | [R].GLFTLIKGAAKLIGKTVA.[KT] |  | HCD |  |  |  | 3.83 |  | 2.4e-3 |  | 7.5e-2 |  | 0.248 |  |  |  |  |  |  |  |  |  |  |  |  |  |  |  | 0.38 |  | 1 |  | Sequest HT (3) |  | 4 |  | 451.03934 |  | 2.53 |  | 38.7152 |  | 10433 |  | 10433 |  | TRINITY\_DN0\_c1\_g1\_i22.p1; TRINITY\_DN591\_c1\_g1\_i1.p1; TRINITY\_DN0\_c1\_g1\_i7.p1 |  | Image |  | Peak List |  |
|  | | | | | | | | | | | | | | | | | | | | | | | | | | | | | | | | | | | | | | | | | | | | | | | | | | | | | |  |
|  | High |  | [R].GLFTLIKGAAKLIGKTV.[A] |  | HCD |  |  |  | 3.31 |  | 2.4e-3 |  | 8.0e-2 |  | 0.232 |  |  |  |  |  |  |  |  |  |  |  |  |  |  |  | 0.61 |  | 1 |  | Sequest HT (3) |  | 3 |  | 577.37000 |  | 0.91 |  | 38.8836 |  | 10522 |  | 10522 |  | TRINITY\_DN0\_c1\_g1\_i22.p1; TRINITY\_DN591\_c1\_g1\_i1.p1; TRINITY\_DN0\_c1\_g1\_i7.p1 |  | Image |  | Peak List |  |
|  | | | | | | | | | | | | | | | | | | | | | | | | | | | | | | | | | | | | | | | | | | | | | | | | | | | | | |  |
|  | High |  | [R].GLFTLIKGAAKLIGKTVAKEAGKTGL.[E] |  | HCD |  |  |  | 3.72 |  | 1.3e-3 |  | 1.8e-3 |  | 1.06 |  |  |  |  |  |  |  |  |  |  |  |  |  |  |  | 0.52 |  | 1 |  | Sequest HT (3) |  | 5 |  | 517.92169 |  | 1.58 |  | 38.9713 |  | 10561 |  | 10561 |  | TRINITY\_DN0\_c1\_g1\_i22.p1; TRINITY\_DN0\_c1\_g1\_i7.p1 |  | Image |  | Peak List |  |
|  | | | | | | | | | | | | | | | | | | | | | | | | | | | | | | | | | | | | | | | | | | | | | | | | | | | | | |  |
|  | High |  | [R].GLFTLIKGAAKLIGKTVAKEAGKTGL.[E] |  | HCD |  |  |  | 6.29 |  | 1.3e-3 |  | 3.4e-3 |  | 0.923 |  |  |  |  |  |  |  |  |  |  |  |  |  |  |  | 0.60 |  | 1 |  | Sequest HT (3) |  | 4 |  | 647.15063 |  | 2.10 |  | 38.9931 |  | 10571 |  | 10571 |  | TRINITY\_DN0\_c1\_g1\_i22.p1; TRINITY\_DN0\_c1\_g1\_i7.p1 |  | Image |  | Peak List |  |
|  | | | | | | | | | | | | | | | | | | | | | | | | | | | | | | | | | | | | | | | | | | | | | | | | | | | | | |  |
|  | High |  | [R].GLFTLIKGAAKLIGKTVAKEAGKTGL.[E] |  | HCD |  |  |  | 5.77 |  | 1.3e-3 |  | 3.2e-5 |  | 1.95 |  |  |  |  |  |  |  |  |  |  |  |  |  |  |  | 0.67 |  | 1 |  | Sequest HT (3) |  | 3 |  | 862.53094 |  | 1.16 |  | 39.0459 |  | 10599 |  | 10599 |  | TRINITY\_DN0\_c1\_g1\_i22.p1; TRINITY\_DN0\_c1\_g1\_i7.p1 |  | Image |  | Peak List |  |
|  | | | | | | | | | | | | | | | | | | | | | | | | | | | | | | | | | | | | | | | | | | | | | | | | | | | | | |  |
|  | High |  | [R].GLFTLIKGAAKLIGKTVAKEAGKTGL.[E] |  | HCD |  |  |  | 3.38 |  | 1.3e-3 |  | 7.2e-3 |  | 0.763 |  |  |  |  |  |  |  |  |  |  |  |  |  |  |  | 0.51 |  | 1 |  | Sequest HT (3) |  | 5 |  | 517.92090 |  | 0.05 |  | 39.1193 |  | 10636 |  | 10636 |  | TRINITY\_DN0\_c1\_g1\_i22.p1; TRINITY\_DN0\_c1\_g1\_i7.p1 |  | Image |  | Peak List |  |
|  | | | | | | | | | | | | | | | | | | | | | | | | | | | | | | | | | | | | | | | | | | | | | | | | | | | | | |  |
|  | High |  | [R].GLFTLIKGAAKLIGKTVAKEAGKTGLEL.[M] |  | HCD |  |  |  | 2.48 |  | 3.1e-3 |  | 1.1e-1 |  | 0.152 |  |  |  |  |  |  |  |  |  |  |  |  |  |  |  | 0.41 |  | 1 |  | Sequest HT (3) |  | 5 |  | 566.34698 |  | 1.38 |  | 39.9805 |  | 11070 |  | 11070 |  | TRINITY\_DN0\_c1\_g1\_i22.p1; TRINITY\_DN0\_c1\_g1\_i7.p1 |  | Image |  | Peak List |  |
|  | | | | | | | | | | | | | | | | | | | | | | | | | | | | | | | | | | | | | | | | | | | | | | | | | | | | | |  |
|  | High |  | [R].GLFTLIKGAAKLIGKTVAKEAGKTGLEL.[M] |  | HCD |  |  |  | 5.61 |  | 1.3e-3 |  | 5.7e-4 |  | 1.32 |  |  |  |  |  |  |  |  |  |  |  |  |  |  |  | 0.63 |  | 1 |  | Sequest HT (3) |  | 3 |  | 943.23663 |  | -2.33 |  | 40.0160 |  | 11090 |  | 11090 |  | TRINITY\_DN0\_c1\_g1\_i22.p1; TRINITY\_DN0\_c1\_g1\_i7.p1 |  | Image |  | Peak List |  |
|  | | | | | | | | | | | | | | | | | | | | | | | | | | | | | | | | | | | | | | | | | | | | | | | | | | | | | |  |
|  | High |  | [R].GLFTLIKGAAKLIGKTVAKEAGKTGLELmAcKITNQc.[-] |  | HCD |  | M29(Oxidation); C31(Dehydro); C37(Dehydro) |  | 4.11 |  | 3.1e-3 |  | 1.1e-1 |  | 0.16 |  | 129.92 |  | 1.000 |  |  |  | M29(Oxidation): 100; C31(Dehydro): 100; C37(Dehydro): 100 |  |  |  |  |  |  |  | 0.60 |  | 1 |  | Sequest HT (3) |  | 4 |  | 959.28204 |  | 0.25 |  | 40.1337 |  | 11151 |  | 11151 |  | TRINITY\_DN0\_c1\_g1\_i22.p1; TRINITY\_DN0\_c1\_g1\_i7.p1 |  | Image |  | Peak List |  |
|  | | | | | | | | | | | | | | | | | | | | | | | | | | | | | | | | | | | | | | | | | | | | | | | | | | | | | |  |
|  | High |  | [R].GLFTLIKGAAKLIGKTVAKEAGKTGLELmAcKITNQc.[-] |  | HCD |  | M29(Oxidation); C31(Dehydro); C37(Dehydro) |  | 3.88 |  | 1.3e-3 |  | 6.1e-2 |  | 0.293 |  | 261.82 |  | 1.000 |  |  |  | M29(Oxidation): 100; C31(Dehydro): 100; C37(Dehydro): 100 |  |  |  |  |  |  |  | 0.66 |  | 1 |  | Sequest HT (3) |  | 3 |  | 1278.70410 |  | -1.99 |  | 40.3466 |  | 11251 |  | 11251 |  | TRINITY\_DN0\_c1\_g1\_i22.p1; TRINITY\_DN0\_c1\_g1\_i7.p1 |  | Image |  | Peak List |  |
|  | | | | | | | | | | | | | | | | | | | | | | | | | | | | | | | | | | | | | | | | | | | | | | | | | | | | | |  |
|  | High |  | [R].GLFTLIKGAAKLIGKTVAKEAGKTGLELmAcKITnQc.[-] |  | HCD |  | M29(Oxidation); C31(Dehydro); N35(Deamidated); C37(Dehydro) |  | 3.66 |  | 1.3e-3 |  | 3.6e-2 |  | 0.408 |  | 141.74 |  | 1.000 |  |  |  | M29(Oxidation): 100; C31(Dehydro): 100; N35(Deamidated): 100; C37(Dehydro): 100 |  |  |  |  |  |  |  | 0.58 |  | 1 |  | Sequest HT (3) |  | 4 |  | 959.52930 |  | 1.56 |  | 40.5358 |  | 11335 |  | 11335 |  | TRINITY\_DN0\_c1\_g1\_i22.p1; TRINITY\_DN0\_c1\_g1\_i7.p1 |  | Image |  | Peak List |  |
|  | | | | | | | | | | | | | | | | | | | | | | | | | | | | | | | | | | | | | | | | | | | | | | | | | | | | | |  |
|  | High |  | [R].GLFTLIKGAAKLIGKTVAKEAGKTGLELmAcKITnQc.[-] |  | HCD |  | M29(Oxidation); C31(Dehydro); N35(Deamidated); C37(Dehydro) |  | 3.92 |  | 1.3e-3 |  | 2.1e-2 |  | 0.527 |  | 238.57 |  | 1.000 |  |  |  | M29(Oxidation): 100; C31(Dehydro): 100; N35(Deamidated): 100; C37(Dehydro): 100 |  |  |  |  |  |  |  | 0.48 |  | 1 |  | Sequest HT (3) |  | 3 |  | 1279.03442 |  | -0.17 |  | 40.5723 |  | 11353 |  | 11353 |  | TRINITY\_DN0\_c1\_g1\_i22.p1; TRINITY\_DN0\_c1\_g1\_i7.p1 |  | Image |  | Peak List |  |
|  | | | | | | | | | | | | | | | | | | | | | | | | | | | | | | | | | | | | | | | | | | | | | | | | | | | | | |  |
|  | High |  | [R].GLFTLIKGAAKLIGKTVAKEAGKTGLELM.[A] |  | HCD |  |  |  | 6.23 |  | 2.4e-3 |  | 7.2e-2 |  | 0.255 |  |  |  |  |  |  |  |  |  |  |  |  |  |  |  | 0.56 |  | 1 |  | Sequest HT (3) |  | 4 |  | 740.44287 |  | 2.45 |  | 40.5787 |  | 11358 |  | 11358 |  | TRINITY\_DN0\_c1\_g1\_i22.p1; TRINITY\_DN0\_c1\_g1\_i7.p1 |  | Image |  | Peak List |  |
|  | | | | | | | | | | | | | | | | | | | | | | | | | | | | | | | | | | | | | | | | | | | | | | | | | | | | | |  |
|  | High |  | [R].GLFTLIKGAAKLIGKTVAKEAGKTGLELmAcKITnQc.[-] |  | HCD |  | M29(Oxidation); C31(Dehydro); N35(Deamidated); C37(Dehydro) |  | 2.73 |  | 3.9e-3 |  | 1.5e-1 |  | 0.091 |  | 253.94 |  | 1.000 |  |  |  | M29(Oxidation): 100; C31(Dehydro): 100; N35(Deamidated): 100; C37(Dehydro): 100 |  |  |  |  |  |  |  | 0.41 |  | 1 |  | Sequest HT (3) |  | 3 |  | 1279.03406 |  | -0.46 |  | 40.7204 |  | 11421 |  | 11421 |  | TRINITY\_DN0\_c1\_g1\_i22.p1; TRINITY\_DN0\_c1\_g1\_i7.p1 |  | Image |  | Peak List |  |
|  | | | | | | | | | | | | | | | | | | | | | | | | | | | | | | | | | | | | | | | | | | | | | | | | | | | | | |  |
|  | High |  | [R].GLFTLIKGAAKLIGKTVAKEAGKTGLELM.[A] |  | HCD |  |  |  | 5.14 |  | 4.7e-3 |  | 2.0e-1 |  | 0.025 |  |  |  |  |  |  |  |  |  |  |  |  |  |  |  | 0.53 |  | 1 |  | Sequest HT (3) |  | 4 |  | 740.44305 |  | 2.70 |  | 40.7586 |  | 11441 |  | 11441 |  | TRINITY\_DN0\_c1\_g1\_i22.p1; TRINITY\_DN0\_c1\_g1\_i7.p1 |  | Image |  | Peak List |  |
|  | | | | | | | | | | | | | | | | | | | | | | | | | | | | | | | | | | | | | | | | | | | | | | | | | | | | | |  |
|  | High |  | [R].GLFTLIKGAAKLIGKTVAKEAGKTGLELMAcKITNQc.[-] |  | HCD |  | C31(Dehydro); C37(Dehydro) |  | 6.31 |  | 1.3e-3 |  | 2.8e-2 |  | 0.463 |  | 209.18 |  | 1.000 |  |  |  | C31(Dehydro): 100; C37(Dehydro): 100 |  |  |  |  |  |  |  | 0.70 |  | 1 |  | Sequest HT (3) |  | 4 |  | 955.28192 |  | -1.20 |  | 42.2311 |  | 12104 |  | 12104 |  | TRINITY\_DN0\_c1\_g1\_i22.p1; TRINITY\_DN0\_c1\_g1\_i7.p1 |  | Image |  | Peak List |  |
|  | | | | | | | | | | | | | | | | | | | | | | | | | | | | | | | | | | | | | | | | | | | | | | | | | | | | | |  |
|  | High |  | [R].GLFTLIKGAAKLIGKTVAKEAGKTGLELMAcKITNQc.[-] |  | HCD |  | C31(Dehydro); C37(Dehydro) |  | 5.75 |  | 1.3e-3 |  | 1.6e-3 |  | 1.09 |  | 363.76 |  | 1.000 |  |  |  | C31(Dehydro): 100; C37(Dehydro): 100 |  |  |  |  |  |  |  | 0.73 |  | 1 |  | Sequest HT (3) |  | 3 |  | 1273.37341 |  | -1.25 |  | 42.2655 |  | 12121 |  | 12121 |  | TRINITY\_DN0\_c1\_g1\_i22.p1; TRINITY\_DN0\_c1\_g1\_i7.p1 |  | Image |  | Peak List |  |
|  | | | | | | | | | | | | | | | | | | | | | | | | | | | | | | | | | | | | | | | | | | | | | | | | | | | | | |  |
|  | High |  | [R].GLFTLIKGAAKLIGKTVAKEAGKTGLELMAcKITNQc.[-] |  | HCD |  | C31(Dehydro); C37(Dehydro) |  | 4.98 |  | 1.3e-3 |  | 5.0e-2 |  | 0.338 |  | 203.81 |  | 1.000 |  |  |  | C31(Dehydro): 100; C37(Dehydro): 100 |  |  |  |  |  |  |  | 0.71 |  | 1 |  | Sequest HT (3) |  | 4 |  | 955.28357 |  | 0.52 |  | 42.3719 |  | 12156 |  | 12156 |  | TRINITY\_DN0\_c1\_g1\_i22.p1; TRINITY\_DN0\_c1\_g1\_i7.p1 |  | Image |  | Peak List |  |
|  | | | | | | | | | | | | | | | | | | | | | | | | | | | | | | | | | | | | | | | | | | | | | | | | | | | | | |  |
|  | High |  | [R].GLFTLIKGAAKLIGKTVAKEAGKTGLELMAcKITNQc.[-] |  | HCD |  | C31(Dehydro); C37(Dehydro) |  | 5.00 |  | 1.3e-3 |  | 9.4e-4 |  | 1.21 |  | 377.66 |  | 1.000 |  |  |  | C31(Dehydro): 100; C37(Dehydro): 100 |  |  |  |  |  |  |  | 0.70 |  | 1 |  | Sequest HT (3) |  | 3 |  | 1273.37463 |  | -0.29 |  | 42.4086 |  | 12170 |  | 12170 |  | TRINITY\_DN0\_c1\_g1\_i22.p1; TRINITY\_DN0\_c1\_g1\_i7.p1 |  | Image |  | Peak List |  |
|  | | | | | | | | | | | | | | | | | | | | | | | | | | | | | | | | | | | | | | | | | | | | | | | | | | | | | |  |
|  | High |  | [R].GLFTLIKGAAKLIGKTVAKEAGKTGLELMAcKITNQc.[-] |  | HCD |  | C31(Dehydro); C37(Dehydro) |  | 4.68 |  | 1.3e-3 |  | 2.7e-2 |  | 0.477 |  | 202.99 |  | 1.000 |  |  |  | C31(Dehydro): 100; C37(Dehydro): 100 |  |  |  |  |  |  |  | 0.70 |  | 1 |  | Sequest HT (3) |  | 4 |  | 955.28345 |  | 0.39 |  | 42.5087 |  | 12201 |  | 12201 |  | TRINITY\_DN0\_c1\_g1\_i22.p1; TRINITY\_DN0\_c1\_g1\_i7.p1 |  | Image |  | Peak List |  |
|  | | | | | | | | | | | | | | | | | | | | | | | | | | | | | | | | | | | | | | | | | | | | | | | | | | | | | |  |
|  | High |  | [R].GLFTLIKGAAKLIGKTVAKEAGKTGLELMAcKITNQc.[-] |  | HCD |  | C31(Dehydro); C37(Dehydro) |  | 3.63 |  | 1.3e-3 |  | 1.6e-2 |  | 0.583 |  | 282.37 |  | 1.000 |  |  |  | C31(Dehydro): 100; C37(Dehydro): 100 |  |  |  |  |  |  |  | 0.67 |  | 1 |  | Sequest HT (3) |  | 3 |  | 1273.37488 |  | -0.10 |  | 42.5557 |  | 12220 |  | 12220 |  | TRINITY\_DN0\_c1\_g1\_i22.p1; TRINITY\_DN0\_c1\_g1\_i7.p1 |  | Image |  | Peak List |  |
|  | | | | | | | | | | | | | | | | | | | | | | | | | | | | | | | | | | | | | | | | | | | | | | | | | | | | | |  |
|  | High |  | [R].GLFTLIKGAAKLIGKTVAKEAGKTGLELMAcKITnQc.[-] |  | HCD |  | C31(Dehydro); N35(Deamidated); C37(Dehydro) |  | 5.67 |  | 1.3e-3 |  | 4.8e-2 |  | 0.346 |  | 191.91 |  | 1.000 |  |  |  | C31(Dehydro): 100; N35(Deamidated): 100; C37(Dehydro): 100 |  |  |  |  |  |  |  | 0.63 |  | 1 |  | Sequest HT (3) |  | 4 |  | 955.52917 |  | 0.10 |  | 42.6586 |  | 12262 |  | 12262 |  | TRINITY\_DN0\_c1\_g1\_i22.p1; TRINITY\_DN0\_c1\_g1\_i7.p1 |  | Image |  | Peak List |  |
|  | | | | | | | | | | | | | | | | | | | | | | | | | | | | | | | | | | | | | | | | | | | | | | | | | | | | | |  |
|  | High |  | [R].GLFTLIKGAAKLIGKTVAKEAGKTGLELMAcKITnQc.[-] |  | HCD |  | C31(Dehydro); N35(Deamidated); C37(Dehydro) |  | 5.95 |  | 1.3e-3 |  | 9.3e-4 |  | 1.21 |  | 368.09 |  | 1.000 |  |  |  | C31(Dehydro): 100; N35(Deamidated): 100; C37(Dehydro): 100 |  |  |  |  |  |  |  | 0.72 |  | 1 |  | Sequest HT (3) |  | 3 |  | 1273.70593 |  | 2.30 |  | 42.7164 |  | 12287 |  | 12287 |  | TRINITY\_DN0\_c1\_g1\_i22.p1; TRINITY\_DN0\_c1\_g1\_i7.p1 |  | Image |  | Peak List |  |
|  | | | | | | | | | | | | | | | | | | | | | | | | | | | | | | | | | | | | | | | | | | | | | | | | | | | | | |  |
|  | High |  | [R].GLFTLIKGAAKLIGKTVAKEAGKTGLELMAcKITnQc.[-] |  | HCD |  | C31(Dehydro); N35(Deamidated); C37(Dehydro) |  | 5.84 |  | 1.3e-3 |  | 1.1e-2 |  | 0.663 |  | 219.32 |  | 1.000 |  |  |  | C31(Dehydro): 100; N35(Deamidated): 100; C37(Dehydro): 100 |  |  |  |  |  |  |  | 0.72 |  | 1 |  | Sequest HT (3) |  | 4 |  | 955.52917 |  | 0.10 |  | 42.8051 |  | 12324 |  | 12324 |  | TRINITY\_DN0\_c1\_g1\_i22.p1; TRINITY\_DN0\_c1\_g1\_i7.p1 |  | Image |  | Peak List |  |
|  | | | | | | | | | | | | | | | | | | | | | | | | | | | | | | | | | | | | | | | | | | | | | | | | | | | | | |  |
|  | High |  | [R].GLFTLIKGAAKLIGKTVAKEAGKTGLELMAcKITnQc.[-] |  | HCD |  | C31(Dehydro); N35(Deamidated); C37(Dehydro) |  | 5.93 |  | 1.3e-3 |  | 1.8e-3 |  | 1.06 |  | 395.93 |  | 1.000 |  |  |  | C31(Dehydro): 100; N35(Deamidated): 100; C37(Dehydro): 100 |  |  |  |  |  |  |  | 0.67 |  | 1 |  | Sequest HT (3) |  | 3 |  | 1273.70288 |  | -0.10 |  | 42.8627 |  | 12350 |  | 12350 |  | TRINITY\_DN0\_c1\_g1\_i22.p1; TRINITY\_DN0\_c1\_g1\_i7.p1 |  | Image |  | Peak List |  |
|  | | | | | | | | | | | | | | | | | | | | | | | | | | | | | | | | | | | | | | | | | | | | | | | | | | | | | |  |
|  | High |  | [R].GLFTLIKGAAKLIGKTVAKEAGKTGLELMAcKITnQc.[-] |  | HCD |  | C31(Dehydro); N35(Deamidated); C37(Dehydro) |  | 5.39 |  | 1.3e-3 |  | 4.3e-2 |  | 0.37 |  | 177.14 |  | 1.000 |  |  |  | C31(Dehydro): 100; N35(Deamidated): 100; C37(Dehydro): 100 |  |  |  |  |  |  |  | 0.65 |  | 1 |  | Sequest HT (3) |  | 4 |  | 955.52789 |  | -1.24 |  | 42.9592 |  | 12391 |  | 12391 |  | TRINITY\_DN0\_c1\_g1\_i22.p1; TRINITY\_DN0\_c1\_g1\_i7.p1 |  | Image |  | Peak List |  |
|  | | | | | | | | | | | | | | | | | | | | | | | | | | | | | | | | | | | | | | | | | | | | | | | | | | | | | |  |
|  | High |  | [R].GLFTLIKGAAKLIGKTVAKEAGKTGLELMAcKITnQc.[-] |  | HCD |  | C31(Dehydro); N35(Deamidated); C37(Dehydro) |  | 3.34 |  | 1.3e-3 |  | 3.1e-3 |  | 0.95 |  | 279.73 |  | 1.000 |  |  |  | C31(Dehydro): 100; N35(Deamidated): 100; C37(Dehydro): 100 |  |  |  |  |  |  |  | 0.61 |  | 1 |  | Sequest HT (3) |  | 3 |  | 1273.70300 |  | 0.00 |  | 43.0086 |  | 12414 |  | 12414 |  | TRINITY\_DN0\_c1\_g1\_i22.p1; TRINITY\_DN0\_c1\_g1\_i7.p1 |  | Image |  | Peak List |  |
|  | | | | | | | | | | | | | | | | | | | | | | | | | | | | | | | | | | | | | | | | | | | | | | | | | | | | | |  |
|  | High |  | [R].GLFTLIKGAAKLIGKTVAKEAGKTGLELMAcKITnQc.[-] |  | HCD |  | C31(Dehydro); N35(Deamidated); C37(Dehydro) |  | 4.19 |  | 1.3e-3 |  | 2.6e-2 |  | 0.485 |  | 197.21 |  | 1.000 |  |  |  | C31(Dehydro): 100; N35(Deamidated): 100; C37(Dehydro): 100 |  |  |  |  |  |  |  | 0.65 |  | 1 |  | Sequest HT (3) |  | 4 |  | 955.53082 |  | 1.83 |  | 43.1240 |  | 12458 |  | 12458 |  | TRINITY\_DN0\_c1\_g1\_i22.p1; TRINITY\_DN0\_c1\_g1\_i7.p1 |  | Image |  | Peak List |  |
|  | | | | | | | | | | | | | | | | | | | | | | | | | | | | | | | | | | | | | | | | | | | | | | | | | | | | | |  |
|  | High |  | [R].GLFTLIKGAAKLIGKTVAKEAGKTGLELmAcKITnQc.[-] |  | HCD |  | M29(Oxidation); C31(Dehydro); N35(Deamidated); C37(Dehydro) |  | 3.02 |  | 1.3e-3 |  | 2.5e-2 |  | 0.488 |  | 260.52 |  | 1.000 |  |  |  | M29(Oxidation): 100; C31(Dehydro): 100; N35(Deamidated): 100; C37(Dehydro): 100 |  |  |  |  |  |  |  | 0.41 |  | 1 |  | Sequest HT (3) |  | 3 |  | 1279.03113 |  | -2.75 |  | 43.5679 |  | 12646 |  | 12646 |  | TRINITY\_DN0\_c1\_g1\_i22.p1; TRINITY\_DN0\_c1\_g1\_i7.p1 |  | Image |  | Peak List |  |
|  | | | | | | | | | | | | | | | | | | | | | | | | | | | | | | | | | | | | | | | | | | | | | | | | | | | | | |  |
|  | **TRINITY\_DN440\_c40\_g1\_i1.p1 - TRINITY\_DN440\_c40\_g1\_i1.p1** | | | | | | | | | | | | | | | | | | | | | | | | | | | | | | | | | | | | | | | | | | | | | | | | | | | |  |  |
|  | | | | | | | | | | | | | | | | | | | | | | | | | | | | | | | | | | | | | | | | | | | | | | | | | | | | | |  |
|  | High |  | [SR].GLLDTFKNmALn.[A] |  | HCD |  | M9(Oxidation); N12(Deamidated) |  | 2.89 |  | 1.3e-3 |  | 3.5e-2 |  | 0.419 |  | 301.14 |  | 1.000 |  | b2+-H(4) C O S(10): 522.28; b1+(8): 889.48; b1+(9): 1036.51; b1+(10): 1107.55; y1+(2): 247.13; y1+(1): 134.04; b2+(9): 518.76 |  | M9(Oxidation): 100; N12(Deamidated): 100 |  |  |  |  |  |  |  | 0.02 |  | 1 |  | Sequest HT (3) |  | 2 |  | 677.33942 |  | -0.24 |  | 31.0150 |  | 6934 |  | 6934 |  | TRINITY\_DN113\_c1\_g1\_i1.p1; TRINITY\_DN440\_c40\_g1\_i1.p1; TRINITY\_DN113\_c1\_g1\_i1.p1\_ORF1 |  | Image |  | Peak List |  |
|  | | | | | | | | | | | | | | | | | | | | | | | | | | | | | | | | | | | | | | | | | | | | | | | | | | | | | |  |
|  | High |  | [SR].GLLDTFKnmALN.[A] |  | HCD |  | N8(Deamidated); M9(Oxidation) |  | 2.82 |  | 3.1e-3 |  | 1.2e-1 |  | 0.133 |  | 206.44 |  | 0.000 |  | b2+-H(4) C O S(10): 522.28; b1+(8): 889.48; b1+(9): 1036.51; b1+(10): 1107.55; y1+(2): 247.13; y1+(1): 134.04; b2+(9): 518.76 |  | M9(Oxidation): 100; N12(Deamidated): 100 |  |  |  |  |  |  |  |  |  | 2 |  | Sequest HT (3) |  | 2 |  | 677.33942 |  | -0.24 |  | 31.0150 |  | 6934 |  | 6934 |  | TRINITY\_DN113\_c1\_g1\_i1.p1; TRINITY\_DN440\_c40\_g1\_i1.p1; TRINITY\_DN113\_c1\_g1\_i1.p1\_ORF1 |  | Image |  | Peak List |  |
|  | | | | | | | | | | | | | | | | | | | | | | | | | | | | | | | | | | | | | | | | | | | | | | | | | | | | | |  |
|  | High |  | [SR].GLLDTFKnmALn.[A] |  | HCD |  | N8(Deamidated); M9(Oxidation); N12(Deamidated) |  | 1.51 |  | 2.4e-3 |  | 8.3e-2 |  | 0.225 |  | 222.37 |  | 1.000 |  |  |  | N8(Deamidated): 100; M9(Oxidation): 100; N12(Deamidated): 100 |  |  |  |  |  |  |  | 0.09 |  | 1 |  | Sequest HT (3) |  | 2 |  | 677.83154 |  | -0.06 |  | 31.9057 |  | 7292 |  | 7292 |  | TRINITY\_DN113\_c1\_g1\_i1.p1; TRINITY\_DN440\_c40\_g1\_i1.p1; TRINITY\_DN113\_c1\_g1\_i1.p1\_ORF1 |  | Image |  | Peak List |  |
|  | | | | | | | | | | | | | | | | | | | | | | | | | | | | | | | | | | | | | | | | | | | | | | | | | | | | | |  |
|  | High |  | [A].KSAGVSVLNALScKLSKTc.[-] |  | HCD |  | C13(Dehydro); C19(Dehydro) |  | 4.18 |  | 1.3e-3 |  | 4.3e-2 |  | 0.373 |  | 279.71 |  | 1.000 |  |  |  | C13(Dehydro): 100; C19(Dehydro): 100 |  |  |  |  |  |  |  | 0.55 |  | 1 |  | Sequest HT (3) |  | 3 |  | 636.34113 |  | -0.03 |  | 32.0993 |  | 7371 |  | 7371 |  | TRINITY\_DN440\_c40\_g1\_i1.p1 |  | Image |  | Peak List |  |
|  | | | | | | | | | | | | | | | | | | | | | | | | | | | | | | | | | | | | | | | | | | | | | | | | | | | | | |  |
|  | High |  | [A].KSAGVSVLNALScKLSKTc.[-] |  | HCD |  | C13(Dehydro); C19(Dehydro) |  | 3.92 |  | 1.3e-3 |  | 1.9e-2 |  | 0.548 |  | 242.46 |  | 1.000 |  |  |  | C13(Dehydro): 100; C19(Dehydro): 100 |  |  |  |  |  |  |  | 0.61 |  | 1 |  | Sequest HT (3) |  | 4 |  | 477.50787 |  | 0.41 |  | 32.1645 |  | 7396 |  | 7396 |  | TRINITY\_DN440\_c40\_g1\_i1.p1 |  | Image |  | Peak List |  |
|  | | | | | | | | | | | | | | | | | | | | | | | | | | | | | | | | | | | | | | | | | | | | | | | | | | | | | |  |
|  | High |  | [SR].GLLDTFKNmAL.[N] |  | HCD |  | M9(Oxidation) |  | 1.95 |  | 1.3e-3 |  | 3.7e-2 |  | 0.406 |  | 188.75 |  | 1.000 |  |  |  | M9(Oxidation): 100 |  |  |  |  |  |  |  | 0.35 |  | 1 |  | Sequest HT (3) |  | 2 |  | 619.82611 |  | 0.01 |  | 32.5839 |  | 7578 |  | 7578 |  | TRINITY\_DN113\_c1\_g1\_i1.p1; TRINITY\_DN440\_c40\_g1\_i1.p1; TRINITY\_DN113\_c1\_g1\_i1.p1\_ORF1 |  | Image |  | Peak List |  |
|  | | | | | | | | | | | | | | | | | | | | | | | | | | | | | | | | | | | | | | | | | | | | | | | | | | | | | |  |
|  | High |  | [N].AAKSAGVSVLNALScKLSKTc.[-] |  | HCD |  | C15(Dehydro); C21(Dehydro) |  | 3.00 |  | 1.3e-3 |  | 1.7e-2 |  | 0.573 |  | 224.83 |  | 1.000 |  |  |  | C15(Dehydro): 100; C21(Dehydro): 100 |  |  |  |  |  |  |  | 0.48 |  | 1 |  | Sequest HT (3) |  | 4 |  | 513.02649 |  | 0.50 |  | 32.7014 |  | 7630 |  | 7630 |  | TRINITY\_DN440\_c40\_g1\_i1.p1 |  | Image |  | Peak List |  |
|  | | | | | | | | | | | | | | | | | | | | | | | | | | | | | | | | | | | | | | | | | | | | | | | | | | | | | |  |
|  | High |  | [N].AAKSAGVSVLNALScKLSKTc.[-] |  | HCD |  | C15(Dehydro); C21(Dehydro) |  | 2.51 |  | 1.3e-3 |  | 2.7e-2 |  | 0.472 |  | 191.90 |  | 1.000 |  |  |  | C15(Dehydro): 100; C21(Dehydro): 100 |  |  |  |  |  |  |  | 0.44 |  | 1 |  | Sequest HT (3) |  | 2 |  | 1025.04492 |  | -0.26 |  | 32.7392 |  | 7647 |  | 7647 |  | TRINITY\_DN440\_c40\_g1\_i1.p1 |  | Image |  | Peak List |  |
|  | | | | | | | | | | | | | | | | | | | | | | | | | | | | | | | | | | | | | | | | | | | | | | | | | | | | | |  |
|  | High |  | [N].AAKSAGVSVLNALScKLSKTc.[-] |  | HCD |  | C15(Dehydro); C21(Dehydro) |  | 2.70 |  | 1.3e-3 |  | 3.3e-2 |  | 0.432 |  | 215.48 |  | 1.000 |  |  |  | C15(Dehydro): 100; C21(Dehydro): 100 |  |  |  |  |  |  |  | 0.50 |  | 1 |  | Sequest HT (3) |  | 3 |  | 683.69891 |  | -0.45 |  | 32.7571 |  | 7655 |  | 7655 |  | TRINITY\_DN440\_c40\_g1\_i1.p1 |  | Image |  | Peak List |  |
|  | | | | | | | | | | | | | | | | | | | | | | | | | | | | | | | | | | | | | | | | | | | | | | | | | | | | | |  |
|  | High |  | [L].NAAKSAGVSVLNALScKLSKTc.[-] |  | HCD |  | C16(Dehydro); C22(Dehydro) |  | 3.50 |  | 1.3e-3 |  | 3.3e-3 |  | 0.933 |  | 333.80 |  | 1.000 |  |  |  | C16(Dehydro): 100; C22(Dehydro): 100 |  |  |  |  |  |  |  | 0.53 |  | 1 |  | Sequest HT (3) |  | 3 |  | 721.71283 |  | -0.97 |  | 33.1483 |  | 7820 |  | 7820 |  | TRINITY\_DN440\_c40\_g1\_i1.p1 |  | Image |  | Peak List |  |
|  | | | | | | | | | | | | | | | | | | | | | | | | | | | | | | | | | | | | | | | | | | | | | | | | | | | | | |  |
|  | High |  | [L].NAAKSAGVSVLNALScKLSKTc.[-] |  | HCD |  | C16(Dehydro); C22(Dehydro) |  | 2.47 |  | 1.3e-3 |  | 3.1e-2 |  | 0.441 |  | 219.27 |  | 1.000 |  |  |  | C16(Dehydro): 100; C22(Dehydro): 100 |  |  |  |  |  |  |  | 0.49 |  | 1 |  | Sequest HT (3) |  | 4 |  | 541.53687 |  | -0.19 |  | 33.1499 |  | 7821 |  | 7821 |  | TRINITY\_DN440\_c40\_g1\_i1.p1 |  | Image |  | Peak List |  |
|  | | | | | | | | | | | | | | | | | | | | | | | | | | | | | | | | | | | | | | | | | | | | | | | | | | | | | |  |
|  | High |  | [A].KSAGVSVLnALScKLSKTc.[-] |  | HCD |  | N9(Deamidated); C13(Dehydro); C19(Dehydro) |  | 3.08 |  | 1.3e-3 |  | 2.7e-2 |  | 0.47 |  | 223.06 |  | 1.000 |  |  |  | N9(Deamidated): 100; C13(Dehydro): 100; C19(Dehydro): 100 |  |  |  |  |  |  |  | 0.43 |  | 1 |  | Sequest HT (3) |  | 3 |  | 636.66888 |  | -0.42 |  | 33.8810 |  | 8133 |  | 8133 |  | TRINITY\_DN440\_c40\_g1\_i1.p1 |  | Image |  | Peak List |  |
|  | | | | | | | | | | | | | | | | | | | | | | | | | | | | | | | | | | | | | | | | | | | | | | | | | | | | | |  |
|  | High |  | [A].GVSVLNALScKLSKTc.[-] |  | HCD |  | C10(Dehydro); C16(Dehydro) |  | 2.07 |  | 3.9e-3 |  | 1.6e-1 |  | 0.076 |  | 182.20 |  | 1.000 |  |  |  | C10(Dehydro): 100; C16(Dehydro): 100 |  |  |  |  |  |  |  | 0.20 |  | 1 |  | Sequest HT (3) |  | 3 |  | 540.95319 |  | 0.14 |  | 34.0966 |  | 8231 |  | 8231 |  | TRINITY\_DN440\_c40\_g1\_i1.p1 |  | Image |  | Peak List |  |
|  | | | | | | | | | | | | | | | | | | | | | | | | | | | | | | | | | | | | | | | | | | | | | | | | | | | | | |  |
|  | High |  | [A].GVSVLNALScKLSKTc.[-] |  | HCD |  | C10(Dehydro); C16(Dehydro) |  | 3.30 |  | 1.3e-3 |  | 3.3e-2 |  | 0.427 |  | 268.84 |  | 1.000 |  |  |  | C10(Dehydro): 100; C16(Dehydro): 100 |  |  |  |  |  |  |  | 0.39 |  | 1 |  | Sequest HT (3) |  | 2 |  | 810.92554 |  | -0.60 |  | 34.1014 |  | 8233 |  | 8233 |  | TRINITY\_DN440\_c40\_g1\_i1.p1 |  | Image |  | Peak List |  |
|  | | | | | | | | | | | | | | | | | | | | | | | | | | | | | | | | | | | | | | | | | | | | | | | | | | | | | |  |
|  | High |  | [A].LNAAKSAGVSVLNALScKLSKTc.[-] |  | HCD |  | C17(Dehydro); C23(Dehydro) |  | 5.34 |  | 1.3e-3 |  | 1.2e-3 |  | 1.15 |  | 341.72 |  | 1.000 |  |  |  | C17(Dehydro): 100; C23(Dehydro): 100 |  |  |  |  |  |  |  | 0.57 |  | 1 |  | Sequest HT (3) |  | 3 |  | 759.41010 |  | 2.48 |  | 34.2495 |  | 8301 |  | 8301 |  | TRINITY\_DN440\_c40\_g1\_i1.p1 |  | Image |  | Peak List |  |
|  | | | | | | | | | | | | | | | | | | | | | | | | | | | | | | | | | | | | | | | | | | | | | | | | | | | | | |  |
|  | High |  | [N].AAKSAGVSVLnALScKLSKTc.[-] |  | HCD |  | N11(Deamidated); C15(Dehydro); C21(Dehydro) |  | 2.82 |  | 1.3e-3 |  | 3.8e-2 |  | 0.398 |  | 240.15 |  | 1.000 |  |  |  | N11(Deamidated): 100; C15(Dehydro): 100; C21(Dehydro): 100 |  |  |  |  |  |  |  | 0.43 |  | 1 |  | Sequest HT (3) |  | 3 |  | 684.02753 |  | 0.44 |  | 34.3080 |  | 8326 |  | 8326 |  | TRINITY\_DN440\_c40\_g1\_i1.p1 |  | Image |  | Peak List |  |
|  | | | | | | | | | | | | | | | | | | | | | | | | | | | | | | | | | | | | | | | | | | | | | | | | | | | | | |  |
|  | High |  | [S].AGVSVLNALScKLSKTc.[-] |  | HCD |  | C11(Dehydro); C17(Dehydro) |  | 3.21 |  | 1.3e-3 |  | 4.9e-2 |  | 0.34 |  | 240.26 |  | 1.000 |  |  |  | C11(Dehydro): 100; C17(Dehydro): 100 |  |  |  |  |  |  |  | 0.29 |  | 1 |  | Sequest HT (3) |  | 3 |  | 564.63190 |  | -0.44 |  | 34.7414 |  | 8523 |  | 8523 |  | TRINITY\_DN440\_c40\_g1\_i1.p1 |  | Image |  | Peak List |  |
|  | | | | | | | | | | | | | | | | | | | | | | | | | | | | | | | | | | | | | | | | | | | | | | | | | | | | | |  |
|  | High |  | [S].AGVSVLNALScKLSKTc.[-] |  | HCD |  | C11(Dehydro); C17(Dehydro) |  | 2.39 |  | 2.4e-3 |  | 6.9e-2 |  | 0.267 |  | 207.60 |  | 1.000 |  |  |  | C11(Dehydro): 100; C17(Dehydro): 100 |  |  |  |  |  |  |  | 0.34 |  | 1 |  | Sequest HT (3) |  | 2 |  | 846.44482 |  | 0.29 |  | 34.7802 |  | 8541 |  | 8541 |  | TRINITY\_DN440\_c40\_g1\_i1.p1 |  | Image |  | Peak List |  |
|  | | | | | | | | | | | | | | | | | | | | | | | | | | | | | | | | | | | | | | | | | | | | | | | | | | | | | |  |
|  | High |  | [L].nAAKSAGVSVLNALScKLSKTc.[-] |  | HCD |  | N1(Deamidated); C16(Dehydro); C22(Dehydro) |  | 4.03 |  | 1.3e-3 |  | 4.0e-3 |  | 0.89 |  | 297.95 |  | 1.000 |  | b1+(2): 187.07; b1+(3): 258.11; b1+(6): 544.27; b1+(7): 601.29; b1+(8): 700.36; b1+(9): 787.39; b1+(10): 886.46; b1+(11): 999.55; y1+(12): 1278.65; y1+(11): 1165.57; b2+(9): 394.20; b2+(10): 443.74; b2+(11): 500.28; y2+(11): 583.29 |  | N1(Deamidated): 100; C16(Dehydro): 100; C22(Dehydro): 100 |  |  |  |  |  |  |  | 0.48 |  | 1 |  | Sequest HT (3) |  | 3 |  | 722.04126 |  | -0.38 |  | 34.8634 |  | 8578 |  | 8578 |  | TRINITY\_DN440\_c40\_g1\_i1.p1 |  | Image |  | Peak List |  |
|  | | | | | | | | | | | | | | | | | | | | | | | | | | | | | | | | | | | | | | | | | | | | | | | | | | | | | |  |
|  | High |  | [L].nAAKSAGVSVLNALScKLSKTc.[-] |  | HCD |  | N1(Deamidated); C16(Dehydro); C22(Dehydro) |  | 2.86 |  | 1.3e-3 |  | 4.8e-2 |  | 0.347 |  | 201.49 |  | 1.000 |  | b1+(3): 258.11; b1+(4): 386.20; b1+(7): 601.29; b1+(8): 700.36; b1+(9): 787.39; y1+(12): 1278.65; y1+(11): 1165.57; y2+(14): 732.88; y2+(13): 689.37; y2+(12): 639.83 |  | N1(Deamidated): 100; C16(Dehydro): 100; C22(Dehydro): 100 |  |  |  |  |  |  |  | 0.28 |  | 1 |  | Sequest HT (3) |  | 4 |  | 541.78265 |  | -0.58 |  | 34.8659 |  | 8579 |  | 8579 |  | TRINITY\_DN440\_c40\_g1\_i1.p1 |  | Image |  | Peak List |  |
|  | | | | | | | | | | | | | | | | | | | | | | | | | | | | | | | | | | | | | | | | | | | | | | | | | | | | | |  |
|  | High |  | [S].AGVSVLNALScKLSKTc.[-] |  | HCD |  | C11(Dehydro); C17(Dehydro) |  | 3.02 |  | 1.3e-3 |  | 2.0e-2 |  | 0.544 |  | 231.46 |  | 1.000 |  |  |  | C11(Dehydro): 100; C17(Dehydro): 100 |  |  |  |  |  |  |  | 0.46 |  | 1 |  | Sequest HT (3) |  | 3 |  | 564.63226 |  | 0.21 |  | 34.8902 |  | 8588 |  | 8588 |  | TRINITY\_DN440\_c40\_g1\_i1.p1 |  | Image |  | Peak List |  |
|  | | | | | | | | | | | | | | | | | | | | | | | | | | | | | | | | | | | | | | | | | | | | | | | | | | | | | |  |
|  | High |  | [M].ALNAAKSAGVSVLNALScKLSKTc.[-] |  | HCD |  | C18(Dehydro); C24(Dehydro) |  | 3.83 |  | 1.3e-3 |  | 1.0e-2 |  | 0.691 |  | 268.47 |  | 1.000 |  |  |  | C18(Dehydro): 100; C24(Dehydro): 100 |  |  |  |  |  |  |  | 0.29 |  | 1 |  | Sequest HT (3) |  | 4 |  | 587.56757 |  | 0.52 |  | 35.1213 |  | 8691 |  | 8691 |  | TRINITY\_DN440\_c40\_g1\_i1.p1 |  | Image |  | Peak List |  |
|  | | | | | | | | | | | | | | | | | | | | | | | | | | | | | | | | | | | | | | | | | | | | | | | | | | | | | |  |
|  | High |  | [M].ALNAAKSAGVSVLNALScKLSKTc.[-] |  | HCD |  | C18(Dehydro); C24(Dehydro) |  | 4.28 |  | 1.3e-3 |  | 3.5e-3 |  | 0.921 |  | 342.24 |  | 1.000 |  |  |  | C18(Dehydro): 100; C24(Dehydro): 100 |  |  |  |  |  |  |  | 0.57 |  | 1 |  | Sequest HT (3) |  | 3 |  | 783.08807 |  | 1.05 |  | 35.1465 |  | 8703 |  | 8703 |  | TRINITY\_DN440\_c40\_g1\_i1.p1 |  | Image |  | Peak List |  |
|  | | | | | | | | | | | | | | | | | | | | | | | | | | | | | | | | | | | | | | | | | | | | | | | | | | | | | |  |
|  | High |  | [F].KNMALNAAKSAGVSVLNALScKLSKTc.[-] |  | HCD |  | C21(Dehydro); C27(Dehydro) |  | 4.00 |  | 1.3e-3 |  | 1.3e-2 |  | 0.631 |  | 253.70 |  | 1.000 |  |  |  | C21(Dehydro): 100; C27(Dehydro): 100 |  |  |  |  |  |  |  | 0.48 |  | 1 |  | Sequest HT (3) |  | 4 |  | 680.86151 |  | -0.50 |  | 36.0616 |  | 9138 |  | 9138 |  | TRINITY\_DN440\_c40\_g1\_i1.p1 |  | Image |  | Peak List |  |
|  | | | | | | | | | | | | | | | | | | | | | | | | | | | | | | | | | | | | | | | | | | | | | | | | | | | | | |  |
|  | High |  | [K].SAGVSVLnALScKLSKTc.[-] |  | HCD |  | N8(Deamidated); C12(Dehydro); C18(Dehydro) |  | 2.97 |  | 1.3e-3 |  | 1.3e-2 |  | 0.632 |  | 256.67 |  | 1.000 |  |  |  | N8(Deamidated): 100; C12(Dehydro): 100; C18(Dehydro): 100 |  |  |  |  |  |  |  | 0.39 |  | 1 |  | Sequest HT (3) |  | 3 |  | 593.97083 |  | -0.01 |  | 36.4116 |  | 9311 |  | 9311 |  | TRINITY\_DN440\_c40\_g1\_i1.p1 |  | Image |  | Peak List |  |
|  | | | | | | | | | | | | | | | | | | | | | | | | | | | | | | | | | | | | | | | | | | | | | | | | | | | | | |  |
|  | High |  | [SR].GLLDTFKNMALNAAK.[S] |  | HCD |  |  |  | 3.83 |  | 1.3e-3 |  | 2.9e-3 |  | 0.96 |  |  |  |  |  |  |  |  |  |  |  |  |  |  |  | 0.59 |  | 1 |  | Sequest HT (3) |  | 2 |  | 803.93237 |  | -2.91 |  | 36.9217 |  | 9556 |  | 9556 |  | TRINITY\_DN113\_c1\_g1\_i1.p1; TRINITY\_DN440\_c40\_g1\_i1.p1; TRINITY\_DN113\_c1\_g1\_i1.p1\_ORF1 |  | Image |  | Peak List |  |
|  | | | | | | | | | | | | | | | | | | | | | | | | | | | | | | | | | | | | | | | | | | | | | | | | | | | | | |  |
|  | High |  | [SR].GLLDTFKNMALNAAK.[S] |  | HCD |  |  |  | 2.31 |  | 1.3e-3 |  | 6.1e-4 |  | 1.3 |  |  |  |  |  |  |  |  |  |  |  |  |  |  |  | 0.53 |  | 1 |  | Sequest HT (3) |  | 2 |  | 803.93530 |  | 0.74 |  | 37.0712 |  | 9628 |  | 9628 |  | TRINITY\_DN113\_c1\_g1\_i1.p1; TRINITY\_DN440\_c40\_g1\_i1.p1; TRINITY\_DN113\_c1\_g1\_i1.p1\_ORF1 |  | Image |  | Peak List |  |
|  | | | | | | | | | | | | | | | | | | | | | | | | | | | | | | | | | | | | | | | | | | | | | | | | | | | | | |  |
|  | High |  | [SR].GLLDTFKNMALnAAK.[S] |  | HCD |  | N12(Deamidated) |  | 3.90 |  | 1.3e-3 |  | 1.2e-2 |  | 0.645 |  | 383.40 |  | 1.000 |  | b1+(10): 1091.56; y1+(7): 719.38; y1+(6): 588.34; y1+(5): 517.30; y1+(4): 404.21; b2+(10): 546.28 |  | N12(Deamidated): 100 |  |  |  |  |  |  |  | 0.22 |  | 1 |  | Sequest HT (3) |  | 2 |  | 804.42468 |  | -2.53 |  | 37.3333 |  | 9756 |  | 9756 |  | TRINITY\_DN113\_c1\_g1\_i1.p1; TRINITY\_DN440\_c40\_g1\_i1.p1; TRINITY\_DN113\_c1\_g1\_i1.p1\_ORF1 |  | Image |  | Peak List |  |
|  | | | | | | | | | | | | | | | | | | | | | | | | | | | | | | | | | | | | | | | | | | | | | | | | | | | | | |  |
|  | High |  | [SR].GLLDTFKNMALn.[A] |  | HCD |  | N12(Deamidated) |  | 2.63 |  | 1.3e-3 |  | 1.2e-2 |  | 0.655 |  | 217.89 |  | 1.000 |  | b1+(8): 889.48; b1+(9): 1020.52; b1+(10): 1091.56; y1+(2): 247.13; y1+(1): 134.04 |  | N12(Deamidated): 100 |  |  |  |  |  |  |  | 0.39 |  | 1 |  | Sequest HT (3) |  | 2 |  | 669.34125 |  | -1.30 |  | 37.3792 |  | 9782 |  | 9782 |  | TRINITY\_DN113\_c1\_g1\_i1.p1; TRINITY\_DN440\_c40\_g1\_i1.p1; TRINITY\_DN113\_c1\_g1\_i1.p1\_ORF1 |  | Image |  | Peak List |  |
|  | | | | | | | | | | | | | | | | | | | | | | | | | | | | | | | | | | | | | | | | | | | | | | | | | | | | | |  |
|  | High |  | [SR].GLLDTFKnMALNAAK.[S] |  | HCD |  | N8(Deamidated) |  | 3.97 |  | 1.3e-3 |  | 7.3e-4 |  | 1.26 |  | 371.15 |  | 1.000 |  | b1+(8): 890.46; b1+(10): 1092.54; b1+(11): 1205.62; y1+(7): 718.39; y1+(6): 587.35; y1+(5): 516.31; y1+(4): 403.23 |  | N8(Deamidated): 100 |  |  |  |  |  |  |  | 0.13 |  | 1 |  | Sequest HT (3) |  | 2 |  | 804.42749 |  | 0.96 |  | 37.6159 |  | 9900 |  | 9900 |  | TRINITY\_DN113\_c1\_g1\_i1.p1; TRINITY\_DN440\_c40\_g1\_i1.p1; TRINITY\_DN113\_c1\_g1\_i1.p1\_ORF1 |  | Image |  | Peak List |  |
|  | | | | | | | | | | | | | | | | | | | | | | | | | | | | | | | | | | | | | | | | | | | | | | | | | | | | | |  |
|  | High |  | [SR].GLLDTFKNMALNAAKSAG.[V] |  | HCD |  |  |  | 5.11 |  | 1.3e-3 |  | 9.5e-5 |  | 1.71 |  |  |  |  |  |  |  |  |  |  |  |  |  |  |  | 0.60 |  | 1 |  | Sequest HT (3) |  | 2 |  | 911.47870 |  | -1.44 |  | 38.9764 |  | 10564 |  | 10564 |  | TRINITY\_DN113\_c1\_g1\_i1.p1; TRINITY\_DN440\_c40\_g1\_i1.p1; TRINITY\_DN113\_c1\_g1\_i1.p1\_ORF1 |  | Image |  | Peak List |  |
|  | | | | | | | | | | | | | | | | | | | | | | | | | | | | | | | | | | | | | | | | | | | | | | | | | | | | | |  |
|  | High |  | [R].GLLDTFKNmALNAAKSAGVSVLNALScKLSKTc.[-] |  | HCD |  | M9(Oxidation); C27(Dehydro); C33(Dehydro) |  | 5.83 |  | 1.3e-3 |  | 3.7e-4 |  | 1.41 |  | 341.36 |  | 1.000 |  |  |  | M9(Oxidation): 100; C27(Dehydro): 100; C33(Dehydro): 100 |  |  |  |  |  |  |  | 0.66 |  | 1 |  | Sequest HT (3) |  | 3 |  | 1128.25464 |  | -1.11 |  | 45.6070 |  | 13499 |  | 13499 |  | TRINITY\_DN440\_c40\_g1\_i1.p1 |  | Image |  | Peak List |  |
|  | | | | | | | | | | | | | | | | | | | | | | | | | | | | | | | | | | | | | | | | | | | | | | | | | | | | | |  |
|  | High |  | [R].GLLDTFKNMALNAAKSAGVSVLNALScKLSKTc.[-] |  | HCD |  | C27(Dehydro); C33(Dehydro) |  | 2.31 |  | 1.3e-3 |  | 2.9e-2 |  | 0.457 |  | 131.99 |  | 1.000 |  |  |  | C27(Dehydro): 100; C33(Dehydro): 100 |  |  |  |  |  |  |  | 0.29 |  | 1 |  | Sequest HT (3) |  | 5 |  | 674.15942 |  | 2.91 |  | 53.0659 |  | 16545 |  | 16545 |  | TRINITY\_DN440\_c40\_g1\_i1.p1 |  | Image |  | Peak List |  |
|  | | | | | | | | | | | | | | | | | | | | | | | | | | | | | | | | | | | | | | | | | | | | | | | | | | | | | |  |
|  | High |  | [R].GLLDTFKNMALNAAKSAGVSVLNALScKLSKTc.[-] |  | HCD |  | C27(Dehydro); C33(Dehydro) |  | 5.72 |  | 1.3e-3 |  | 1.0e-4 |  | 1.69 |  | 397.32 |  | 1.000 |  |  |  | C27(Dehydro): 100; C33(Dehydro): 100 |  |  |  |  |  |  |  | 0.48 |  | 1 |  | Sequest HT (3) |  | 3 |  | 1122.92542 |  | 1.03 |  | 53.1228 |  | 16566 |  | 16566 |  | TRINITY\_DN440\_c40\_g1\_i1.p1 |  | Image |  | Peak List |  |
|  | | | | | | | | | | | | | | | | | | | | | | | | | | | | | | | | | | | | | | | | | | | | | | | | | | | | | |  |
|  | High |  | [R].GLLDTFKNMALNAAKSAGVSVLNALScKLSKTc.[-] |  | HCD |  | C27(Dehydro); C33(Dehydro) |  | 4.86 |  | 1.3e-3 |  | 2.5e-3 |  | 0.997 |  | 303.65 |  | 1.000 |  |  |  | C27(Dehydro): 100; C33(Dehydro): 100 |  |  |  |  |  |  |  | 0.57 |  | 1 |  | Sequest HT (3) |  | 4 |  | 842.44568 |  | 0.79 |  | 53.1390 |  | 16571 |  | 16571 |  | TRINITY\_DN440\_c40\_g1\_i1.p1 |  | Image |  | Peak List |  |
|  | | | | | | | | | | | | | | | | | | | | | | | | | | | | | | | | | | | | | | | | | | | | | | | | | | | | | |  |
|  | High |  | [R].GLLDTFKnMALNAAKSAGVSVLNALScKLSKTc.[-] |  | HCD |  | N8(Deamidated); C27(Dehydro); C33(Dehydro) |  | 5.18 |  | 1.3e-3 |  | 1.2e-3 |  | 1.15 |  | 288.88 |  | 1.000 |  | b1+(8): 890.46; b1+(11): 1205.62 |  | N8(Deamidated): 99.96; C27(Dehydro): 100; C33(Dehydro): 100 |  |  |  |  |  |  |  | 0.06 |  | 1 |  | Sequest HT (3) |  | 3 |  | 1123.24976 |  | -2.23 |  | 53.2672 |  | 16614 |  | 16614 |  | TRINITY\_DN440\_c40\_g1\_i1.p1 |  | Image |  | Peak List |  |
|  | | | | | | | | | | | | | | | | | | | | | | | | | | | | | | | | | | | | | | | | | | | | | | | | | | | | | |  |
|  | High |  | [R].GLLDTFKnMALNAAKSAGVSVLNALScKLSKTc.[-] |  | HCD |  | N8(Deamidated); C27(Dehydro); C33(Dehydro) |  | 6.85 |  | 1.3e-3 |  | 1.5e-3 |  | 1.1 |  | 356.19 |  | 1.000 |  | b1+(8): 890.46; b1+(10): 1092.54; b1+(11): 1205.62; b2+(11): 603.32 |  | N8(Deamidated): 100; C27(Dehydro): 100; C33(Dehydro): 100 |  |  |  |  |  |  |  | 0.13 |  | 1 |  | Sequest HT (3) |  | 4 |  | 842.69110 |  | 0.10 |  | 53.2788 |  | 16620 |  | 16620 |  | TRINITY\_DN440\_c40\_g1\_i1.p1 |  | Image |  | Peak List |  |
|  | | | | | | | | | | | | | | | | | | | | | | | | | | | | | | | | | | | | | | | | | | | | | | | | | | | | | |  |
|  | **TRINITY\_DN0\_c1\_g1\_i14.p1,TRINITY\_DN0\_c1\_g1\_i4.p1 - TRINITY\_DN0\_c1\_g1\_i14.p1,TRINITY\_DN0\_c1\_g1\_i4.p1** | | | | | | | | | | | | | | | | | | | | | | | | | | | | | | | | | | | | | | | | | | | | | | | | | | | |  |  |
|  | | | | | | | | | | | | | | | | | | | | | | | | | | | | | | | | | | | | | | | | | | | | | | | | | | | | | |  |
|  | High |  | [A].KSAGVSVLNSLScKLSKTc.[-] |  | HCD |  | C13(Dehydro); C19(Dehydro) |  | 3.46 |  | 1.3e-3 |  | 2.8e-2 |  | 0.465 |  | 263.09 |  | 1.000 |  |  |  | C13(Dehydro): 100; C19(Dehydro): 100 |  |  |  |  |  |  |  | 0.63 |  | 1 |  | Sequest HT (3) |  | 3 |  | 641.67218 |  | -0.94 |  | 29.7343 |  | 6441 |  | 6441 |  | TRINITY\_DN0\_c1\_g1\_i14.p1,TRINITY\_DN0\_c1\_g1\_i4.p1 |  | Image |  | Peak List |  |
|  | | | | | | | | | | | | | | | | | | | | | | | | | | | | | | | | | | | | | | | | | | | | | | | | | | | | | |  |
|  | High |  | [A].KSAGVSVLNSLScKLSKTc.[-] |  | HCD |  | C13(Dehydro); C19(Dehydro) |  | 4.14 |  | 1.3e-3 |  | 3.6e-2 |  | 0.408 |  | 236.79 |  | 1.000 |  |  |  | C13(Dehydro): 100; C19(Dehydro): 100 |  |  |  |  |  |  |  | 0.64 |  | 1 |  | Sequest HT (3) |  | 4 |  | 481.50604 |  | -0.76 |  | 29.7656 |  | 6453 |  | 6453 |  | TRINITY\_DN0\_c1\_g1\_i14.p1,TRINITY\_DN0\_c1\_g1\_i4.p1 |  | Image |  | Peak List |  |
|  | | | | | | | | | | | | | | | | | | | | | | | | | | | | | | | | | | | | | | | | | | | | | | | | | | | | | |  |
|  | High |  | [A].KSAGVSVLNSLScKLSKTc.[-] |  | HCD |  | C13(Dehydro); C19(Dehydro) |  | 1.70 |  | 4.7e-3 |  | 1.9e-1 |  | 0.029 |  | 164.96 |  | 1.000 |  |  |  | C13(Dehydro): 100; C19(Dehydro): 100 |  |  |  |  |  |  |  | 0.52 |  | 1 |  | Sequest HT (3) |  | 2 |  | 962.00537 |  | -0.17 |  | 29.7681 |  | 6454 |  | 6454 |  | TRINITY\_DN0\_c1\_g1\_i14.p1,TRINITY\_DN0\_c1\_g1\_i4.p1 |  | Image |  | Peak List |  |
|  | | | | | | | | | | | | | | | | | | | | | | | | | | | | | | | | | | | | | | | | | | | | | | | | | | | | | |  |
|  | High |  | [A].KSAGVSVLNSLScKLSKTc.[-] |  | HCD |  | C13(Dehydro); C19(Dehydro) |  | 3.52 |  | 1.3e-3 |  | 9.3e-4 |  | 1.21 |  | 351.96 |  | 1.000 |  |  |  | C13(Dehydro): 100; C19(Dehydro): 100 |  |  |  |  |  |  |  | 0.58 |  | 1 |  | Sequest HT (3) |  | 3 |  | 641.67285 |  | 0.11 |  | 29.8563 |  | 6486 |  | 6486 |  | TRINITY\_DN0\_c1\_g1\_i14.p1,TRINITY\_DN0\_c1\_g1\_i4.p1 |  | Image |  | Peak List |  |
|  | | | | | | | | | | | | | | | | | | | | | | | | | | | | | | | | | | | | | | | | | | | | | | | | | | | | | |  |
|  | High |  | [A].KSAGVSVLNSLScKLSKTc.[-] |  | HCD |  | C13(Dehydro); C19(Dehydro) |  | 2.15 |  | 3.9e-3 |  | 1.5e-1 |  | 0.09 |  | 191.33 |  | 1.000 |  |  |  | C13(Dehydro): 100; C19(Dehydro): 100 |  |  |  |  |  |  |  | 0.55 |  | 1 |  | Sequest HT (3) |  | 3 |  | 641.67303 |  | 0.39 |  | 29.8832 |  | 6496 |  | 6496 |  | TRINITY\_DN0\_c1\_g1\_i14.p1,TRINITY\_DN0\_c1\_g1\_i4.p1 |  | Image |  | Peak List |  |
|  | | | | | | | | | | | | | | | | | | | | | | | | | | | | | | | | | | | | | | | | | | | | | | | | | | | | | |  |
|  | High |  | [A].KSAGVSVLNSLScKLSKTc.[-] |  | HCD |  | C13(Dehydro); C19(Dehydro) |  | 3.69 |  | 1.3e-3 |  | 2.7e-2 |  | 0.478 |  | 232.66 |  | 1.000 |  |  |  | C13(Dehydro): 100; C19(Dehydro): 100 |  |  |  |  |  |  |  | 0.66 |  | 1 |  | Sequest HT (3) |  | 4 |  | 481.50616 |  | -0.50 |  | 29.9333 |  | 6518 |  | 6518 |  | TRINITY\_DN0\_c1\_g1\_i14.p1,TRINITY\_DN0\_c1\_g1\_i4.p1 |  | Image |  | Peak List |  |
|  | | | | | | | | | | | | | | | | | | | | | | | | | | | | | | | | | | | | | | | | | | | | | | | | | | | | | |  |
|  | High |  | [A].AKSAGVSVLNSLScKLSKTc.[-] |  | HCD |  | C14(Dehydro); C20(Dehydro) |  | 4.40 |  | 1.3e-3 |  | 8.6e-3 |  | 0.723 |  | 268.60 |  | 1.000 |  |  |  | C14(Dehydro): 100; C20(Dehydro): 100 |  |  |  |  |  |  |  | 0.60 |  | 1 |  | Sequest HT (3) |  | 4 |  | 499.26678 |  | 2.21 |  | 30.1390 |  | 6601 |  | 6601 |  | TRINITY\_DN0\_c1\_g1\_i14.p1,TRINITY\_DN0\_c1\_g1\_i4.p1 |  | Image |  | Peak List |  |
|  | | | | | | | | | | | | | | | | | | | | | | | | | | | | | | | | | | | | | | | | | | | | | | | | | | | | | |  |
|  | High |  | [A].AKSAGVSVLNSLScKLSKTC.[-] |  | HCD |  | C14(Dehydro) |  | 3.56 |  | 1.3e-3 |  | 3.3e-2 |  | 0.427 |  | 217.42 |  | 0.500 |  |  |  | C14(Dehydro): 50; C20(Dehydro): 50 |  |  |  |  |  |  |  | 0.04 |  | 1 |  | Sequest HT (3) |  | 4 |  | 499.51761 |  | -0.06 |  | 30.1390 |  | 6601 |  | 6601 |  | TRINITY\_DN0\_c1\_g1\_i14.p1,TRINITY\_DN0\_c1\_g1\_i4.p1 |  | Image |  | Peak List |  |
|  | | | | | | | | | | | | | | | | | | | | | | | | | | | | | | | | | | | | | | | | | | | | | | | | | | | | | |  |
|  | High |  | [A].AKSAGVSVLNSLSCKLSKTc.[-] |  | HCD |  | C20(Dehydro) |  | 3.40 |  | 1.3e-3 |  | 3.3e-2 |  | 0.433 |  | 217.42 |  | 0.500 |  |  |  | C14(Dehydro): 50; C20(Dehydro): 50 |  |  |  |  |  |  |  |  |  | 2 |  | Sequest HT (3) |  | 4 |  | 499.51761 |  | -0.06 |  | 30.1390 |  | 6601 |  | 6601 |  | TRINITY\_DN0\_c1\_g1\_i14.p1,TRINITY\_DN0\_c1\_g1\_i4.p1 |  | Image |  | Peak List |  |
|  | | | | | | | | | | | | | | | | | | | | | | | | | | | | | | | | | | | | | | | | | | | | | | | | | | | | | |  |
|  | High |  | [A].AKSAGVSVLNSLScKLSKTc.[-] |  | HCD |  | C14(Dehydro); C20(Dehydro) |  | 2.22 |  | 1.3e-3 |  | 1.6e-2 |  | 0.583 |  | 191.80 |  | 1.000 |  |  |  | C14(Dehydro): 100; C20(Dehydro): 100 |  |  |  |  |  |  |  | 0.47 |  | 1 |  | Sequest HT (3) |  | 2 |  | 997.52301 |  | -1.08 |  | 30.1650 |  | 6611 |  | 6611 |  | TRINITY\_DN0\_c1\_g1\_i14.p1,TRINITY\_DN0\_c1\_g1\_i4.p1 |  | Image |  | Peak List |  |
|  | | | | | | | | | | | | | | | | | | | | | | | | | | | | | | | | | | | | | | | | | | | | | | | | | | | | | |  |
|  | High |  | [A].AKSAGVSVLNSLScKLSKTc.[-] |  | HCD |  | C14(Dehydro); C20(Dehydro) |  | 3.97 |  | 1.3e-3 |  | 2.7e-2 |  | 0.471 |  | 253.34 |  | 1.000 |  |  |  | C14(Dehydro): 100; C20(Dehydro): 100 |  |  |  |  |  |  |  | 0.53 |  | 1 |  | Sequest HT (3) |  | 4 |  | 499.26587 |  | 0.37 |  | 30.2783 |  | 6653 |  | 6653 |  | TRINITY\_DN0\_c1\_g1\_i14.p1,TRINITY\_DN0\_c1\_g1\_i4.p1 |  | Image |  | Peak List |  |
|  | | | | | | | | | | | | | | | | | | | | | | | | | | | | | | | | | | | | | | | | | | | | | | | | | | | | | |  |
|  | High |  | [A].AKSAGVSVLNSLScKLSKTc.[-] |  | HCD |  | C14(Dehydro); C20(Dehydro) |  | 3.22 |  | 1.3e-3 |  | 5.9e-4 |  | 1.31 |  | 312.23 |  | 1.000 |  |  |  | C14(Dehydro): 100; C20(Dehydro): 100 |  |  |  |  |  |  |  | 0.56 |  | 1 |  | Sequest HT (3) |  | 2 |  | 997.52374 |  | -0.35 |  | 30.3068 |  | 6664 |  | 6664 |  | TRINITY\_DN0\_c1\_g1\_i14.p1,TRINITY\_DN0\_c1\_g1\_i4.p1 |  | Image |  | Peak List |  |
|  | | | | | | | | | | | | | | | | | | | | | | | | | | | | | | | | | | | | | | | | | | | | | | | | | | | | | |  |
|  | High |  | [N].AAKSAGVSVLNSLScKLSKTc.[-] |  | HCD |  | C15(Dehydro); C21(Dehydro) |  | 4.61 |  | 1.3e-3 |  | 3.7e-3 |  | 0.909 |  | 316.40 |  | 1.000 |  |  |  | C15(Dehydro): 100; C21(Dehydro): 100 |  |  |  |  |  |  |  | 0.67 |  | 1 |  | Sequest HT (3) |  | 3 |  | 689.03113 |  | 0.39 |  | 30.3577 |  | 6684 |  | 6684 |  | TRINITY\_DN0\_c1\_g1\_i14.p1,TRINITY\_DN0\_c1\_g1\_i4.p1 |  | Image |  | Peak List |  |
|  | | | | | | | | | | | | | | | | | | | | | | | | | | | | | | | | | | | | | | | | | | | | | | | | | | | | | |  |
|  | High |  | [N].AAKSAGVSVLNSLScKLSKTc.[-] |  | HCD |  | C15(Dehydro); C21(Dehydro) |  | 3.42 |  | 1.3e-3 |  | 2.4e-2 |  | 0.502 |  | 268.31 |  | 1.000 |  |  |  | C15(Dehydro): 100; C21(Dehydro): 100 |  |  |  |  |  |  |  | 0.61 |  | 1 |  | Sequest HT (3) |  | 4 |  | 517.02557 |  | 1.18 |  | 30.3606 |  | 6686 |  | 6686 |  | TRINITY\_DN0\_c1\_g1\_i14.p1,TRINITY\_DN0\_c1\_g1\_i4.p1 |  | Image |  | Peak List |  |
|  | | | | | | | | | | | | | | | | | | | | | | | | | | | | | | | | | | | | | | | | | | | | | | | | | | | | | |  |
|  | High |  | [N].AAKSAGVSVLNSLScKLSKTc.[-] |  | HCD |  | C15(Dehydro); C21(Dehydro) |  | 3.62 |  | 1.3e-3 |  | 5.5e-4 |  | 1.33 |  | 315.32 |  | 1.000 |  |  |  | C15(Dehydro): 100; C21(Dehydro): 100 |  |  |  |  |  |  |  | 0.65 |  | 1 |  | Sequest HT (3) |  | 2 |  | 1033.04260 |  | -0.04 |  | 30.3652 |  | 6688 |  | 6688 |  | TRINITY\_DN0\_c1\_g1\_i14.p1,TRINITY\_DN0\_c1\_g1\_i4.p1 |  | Image |  | Peak List |  |
|  | | | | | | | | | | | | | | | | | | | | | | | | | | | | | | | | | | | | | | | | | | | | | | | | | | | | | |  |
|  | High |  | [N].AAKSAGVSVLNSLScKLSKTc.[-] |  | HCD |  | C15(Dehydro); C21(Dehydro) |  | 3.76 |  | 1.3e-3 |  | 8.0e-3 |  | 0.739 |  | 262.13 |  | 1.000 |  |  |  | C15(Dehydro): 100; C21(Dehydro): 100 |  |  |  |  |  |  |  | 0.64 |  | 1 |  | Sequest HT (3) |  | 3 |  | 689.03119 |  | 0.48 |  | 30.4985 |  | 6736 |  | 6736 |  | TRINITY\_DN0\_c1\_g1\_i14.p1,TRINITY\_DN0\_c1\_g1\_i4.p1 |  | Image |  | Peak List |  |
|  | | | | | | | | | | | | | | | | | | | | | | | | | | | | | | | | | | | | | | | | | | | | | | | | | | | | | |  |
|  | High |  | [N].AAKSAGVSVLNSLScKLSKTc.[-] |  | HCD |  | C15(Dehydro); C21(Dehydro) |  | 3.07 |  | 1.3e-3 |  | 1.2e-3 |  | 1.16 |  | 279.00 |  | 1.000 |  |  |  | C15(Dehydro): 100; C21(Dehydro): 100 |  |  |  |  |  |  |  | 0.57 |  | 1 |  | Sequest HT (3) |  | 2 |  | 1033.04285 |  | 0.19 |  | 30.5127 |  | 6742 |  | 6742 |  | TRINITY\_DN0\_c1\_g1\_i14.p1,TRINITY\_DN0\_c1\_g1\_i4.p1 |  | Image |  | Peak List |  |
|  | | | | | | | | | | | | | | | | | | | | | | | | | | | | | | | | | | | | | | | | | | | | | | | | | | | | | |  |
|  | High |  | [G].VSVLNSLScKLSKTc.[-] |  | HCD |  | C9(Dehydro); C15(Dehydro) |  | 3.44 |  | 3.9e-3 |  | 1.8e-1 |  | 0.052 |  | 191.39 |  | 1.000 |  |  |  | C9(Dehydro): 100; C15(Dehydro): 100 |  |  |  |  |  |  |  | 0.28 |  | 1 |  | Sequest HT (3) |  | 3 |  | 527.27704 |  | -1.05 |  | 30.6269 |  | 6785 |  | 6785 |  | TRINITY\_DN0\_c1\_g1\_i14.p1,TRINITY\_DN0\_c1\_g1\_i4.p1 |  | Image |  | Peak List |  |
|  | | | | | | | | | | | | | | | | | | | | | | | | | | | | | | | | | | | | | | | | | | | | | | | | | | | | | |  |
|  | High |  | [D].TFKNLALNAAKSAGVSVL.[N] |  | HCD |  |  |  | 2.76 |  | 2.4e-3 |  | 7.0e-2 |  | 0.261 |  |  |  |  |  |  |  |  |  |  |  |  |  |  |  | 0.52 |  | 1 |  | Sequest HT (3) |  | 3 |  | 602.01666 |  | 1.51 |  | 30.6294 |  | 6786 |  | 6786 |  | TRINITY\_DN0\_c1\_g1\_i14.p1,TRINITY\_DN0\_c1\_g1\_i4.p1 |  | Image |  | Peak List |  |
|  | | | | | | | | | | | | | | | | | | | | | | | | | | | | | | | | | | | | | | | | | | | | | | | | | | | | | |  |
|  | High |  | [G].VSVLNSLScKLSKTc.[-] |  | HCD |  | C9(Dehydro); C15(Dehydro) |  | 2.19 |  | 1.3e-3 |  | 5.9e-2 |  | 0.3 |  | 249.98 |  | 1.000 |  |  |  | C9(Dehydro): 100; C15(Dehydro): 100 |  |  |  |  |  |  |  | 0.32 |  | 1 |  | Sequest HT (3) |  | 2 |  | 790.41266 |  | -0.12 |  | 30.6716 |  | 6803 |  | 6803 |  | TRINITY\_DN0\_c1\_g1\_i14.p1,TRINITY\_DN0\_c1\_g1\_i4.p1 |  | Image |  | Peak List |  |
|  | | | | | | | | | | | | | | | | | | | | | | | | | | | | | | | | | | | | | | | | | | | | | | | | | | | | | |  |
|  | High |  | [L].NAAKSAGVSVLNSLScKLSKTc.[-] |  | HCD |  | C16(Dehydro); C22(Dehydro) |  | 2.70 |  | 1.3e-3 |  | 4.8e-2 |  | 0.346 |  | 231.71 |  | 1.000 |  |  |  | C16(Dehydro): 100; C22(Dehydro): 100 |  |  |  |  |  |  |  | 0.41 |  | 1 |  | Sequest HT (3) |  | 4 |  | 545.53613 |  | 0.80 |  | 30.8876 |  | 6885 |  | 6885 |  | TRINITY\_DN0\_c1\_g1\_i14.p1,TRINITY\_DN0\_c1\_g1\_i4.p1 |  | Image |  | Peak List |  |
|  | | | | | | | | | | | | | | | | | | | | | | | | | | | | | | | | | | | | | | | | | | | | | | | | | | | | | |  |
|  | High |  | [L].NAAKSAGVSVLNSLScKLSKTc.[-] |  | HCD |  | C16(Dehydro); C22(Dehydro) |  | 2.01 |  | 1.3e-3 |  | 6.0e-2 |  | 0.296 |  | 202.45 |  | 1.000 |  |  |  | C16(Dehydro): 100; C22(Dehydro): 100 |  |  |  |  |  |  |  | 0.54 |  | 1 |  | Sequest HT (3) |  | 2 |  | 1090.06421 |  | 0.09 |  | 30.9299 |  | 6901 |  | 6901 |  | TRINITY\_DN0\_c1\_g1\_i14.p1,TRINITY\_DN0\_c1\_g1\_i4.p1 |  | Image |  | Peak List |  |
|  | | | | | | | | | | | | | | | | | | | | | | | | | | | | | | | | | | | | | | | | | | | | | | | | | | | | | |  |
|  | High |  | [L].NAAKSAGVSVLNSLScKLSKTc.[-] |  | HCD |  | C16(Dehydro); C22(Dehydro) |  | 4.06 |  | 1.3e-3 |  | 9.2e-4 |  | 1.21 |  | 403.64 |  | 1.000 |  |  |  | C16(Dehydro): 100; C22(Dehydro): 100 |  |  |  |  |  |  |  | 0.62 |  | 1 |  | Sequest HT (3) |  | 3 |  | 727.04498 |  | -0.25 |  | 30.9408 |  | 6906 |  | 6906 |  | TRINITY\_DN0\_c1\_g1\_i14.p1,TRINITY\_DN0\_c1\_g1\_i4.p1 |  | Image |  | Peak List |  |
|  | | | | | | | | | | | | | | | | | | | | | | | | | | | | | | | | | | | | | | | | | | | | | | | | | | | | | |  |
|  | High |  | [L].NAAKSAGVSVLNSLScKLSKTc.[-] |  | HCD |  | C16(Dehydro); C22(Dehydro) |  | 2.39 |  | 1.3e-3 |  | 6.8e-2 |  | 0.27 |  | 180.48 |  | 1.000 |  |  |  | C16(Dehydro): 100; C22(Dehydro): 100 |  |  |  |  |  |  |  | 0.40 |  | 1 |  | Sequest HT (3) |  | 4 |  | 545.53619 |  | 0.92 |  | 31.0592 |  | 6951 |  | 6951 |  | TRINITY\_DN0\_c1\_g1\_i14.p1,TRINITY\_DN0\_c1\_g1\_i4.p1 |  | Image |  | Peak List |  |
|  | | | | | | | | | | | | | | | | | | | | | | | | | | | | | | | | | | | | | | | | | | | | | | | | | | | | | |  |
|  | High |  | [A].KSAGVSVLnSLScKLSKTc.[-] |  | HCD |  | N9(Deamidated); C13(Dehydro); C19(Dehydro) |  | 4.11 |  | 1.3e-3 |  | 2.6e-3 |  | 0.984 |  | 314.97 |  | 1.000 |  |  |  | N9(Deamidated): 100; C13(Dehydro): 100; C19(Dehydro): 100 |  |  |  |  |  |  |  | 0.55 |  | 1 |  | Sequest HT (3) |  | 3 |  | 641.99994 |  | -1.32 |  | 31.3694 |  | 7079 |  | 7079 |  | TRINITY\_DN0\_c1\_g1\_i14.p1,TRINITY\_DN0\_c1\_g1\_i4.p1 |  | Image |  | Peak List |  |
|  | | | | | | | | | | | | | | | | | | | | | | | | | | | | | | | | | | | | | | | | | | | | | | | | | | | | | |  |
|  | High |  | [A].KSAGVSVLnSLScKLSKTc.[-] |  | HCD |  | N9(Deamidated); C13(Dehydro); C19(Dehydro) |  | 2.07 |  | 1.3e-3 |  | 3.9e-4 |  | 1.4 |  | 317.83 |  | 1.000 |  |  |  | N9(Deamidated): 100; C13(Dehydro): 100; C19(Dehydro): 100 |  |  |  |  |  |  |  | 0.48 |  | 1 |  | Sequest HT (3) |  | 2 |  | 962.49799 |  | 0.46 |  | 31.4335 |  | 7103 |  | 7103 |  | TRINITY\_DN0\_c1\_g1\_i14.p1,TRINITY\_DN0\_c1\_g1\_i4.p1 |  | Image |  | Peak List |  |
|  | | | | | | | | | | | | | | | | | | | | | | | | | | | | | | | | | | | | | | | | | | | | | | | | | | | | | |  |
|  | High |  | [A].KSAGVSVLnSLScKLSKTc.[-] |  | HCD |  | N9(Deamidated); C13(Dehydro); C19(Dehydro) |  | 3.77 |  | 1.3e-3 |  | 2.7e-2 |  | 0.472 |  | 238.84 |  | 1.000 |  |  |  | N9(Deamidated): 100; C13(Dehydro): 100; C19(Dehydro): 100 |  |  |  |  |  |  |  | 0.60 |  | 1 |  | Sequest HT (3) |  | 4 |  | 481.75238 |  | -0.06 |  | 31.4933 |  | 7125 |  | 7125 |  | TRINITY\_DN0\_c1\_g1\_i14.p1,TRINITY\_DN0\_c1\_g1\_i4.p1 |  | Image |  | Peak List |  |
|  | | | | | | | | | | | | | | | | | | | | | | | | | | | | | | | | | | | | | | | | | | | | | | | | | | | | | |  |
|  | High |  | [A].KSAGVSVLnSLScKLSKTc.[-] |  | HCD |  | N9(Deamidated); C13(Dehydro); C19(Dehydro) |  | 2.38 |  | 1.3e-3 |  | 4.9e-3 |  | 0.848 |  | 269.21 |  | 1.000 |  |  |  | N9(Deamidated): 100; C13(Dehydro): 100; C19(Dehydro): 100 |  |  |  |  |  |  |  | 0.52 |  | 1 |  | Sequest HT (3) |  | 3 |  | 642.00085 |  | 0.10 |  | 31.5163 |  | 7134 |  | 7134 |  | TRINITY\_DN0\_c1\_g1\_i14.p1,TRINITY\_DN0\_c1\_g1\_i4.p1 |  | Image |  | Peak List |  |
|  | | | | | | | | | | | | | | | | | | | | | | | | | | | | | | | | | | | | | | | | | | | | | | | | | | | | | |  |
|  | High |  | [A].GVSVLNSLScKLSKTc.[-] |  | HCD |  | C10(Dehydro); C16(Dehydro) |  | 3.19 |  | 1.3e-3 |  | 6.3e-2 |  | 0.288 |  | 258.72 |  | 1.000 |  |  |  | C10(Dehydro): 100; C16(Dehydro): 100 |  |  |  |  |  |  |  | 0.34 |  | 1 |  | Sequest HT (3) |  | 2 |  | 818.92401 |  | 0.65 |  | 31.6751 |  | 7196 |  | 7196 |  | TRINITY\_DN0\_c1\_g1\_i14.p1,TRINITY\_DN0\_c1\_g1\_i4.p1 |  | Image |  | Peak List |  |
|  | | | | | | | | | | | | | | | | | | | | | | | | | | | | | | | | | | | | | | | | | | | | | | | | | | | | | |  |
|  | High |  | [R].GLLDTFKNLALNAAKSAG.[V] |  | HCD |  |  |  | 2.22 |  | 4.7e-3 |  | 1.9e-1 |  | 0.032 |  |  |  |  |  |  |  |  |  |  |  |  |  |  |  | 0.50 |  | 1 |  | Sequest HT (3) |  | 3 |  | 602.00342 |  | -0.35 |  | 31.6866 |  | 7202 |  | 7202 |  | TRINITY\_DN0\_c1\_g1\_i14.p1,TRINITY\_DN0\_c1\_g1\_i4.p1 |  | Image |  | Peak List |  |
|  | | | | | | | | | | | | | | | | | | | | | | | | | | | | | | | | | | | | | | | | | | | | | | | | | | | | | |  |
|  | High |  | [A].AKSAGVSVLnSLScKLSKTc.[-] |  | HCD |  | N10(Deamidated); C14(Dehydro); C20(Dehydro) |  | 4.45 |  | 1.3e-3 |  | 2.2e-3 |  | 1.03 |  | 331.91 |  | 1.000 |  |  |  | N10(Deamidated): 100; C14(Dehydro): 100; C20(Dehydro): 100 |  |  |  |  |  |  |  | 0.65 |  | 1 |  | Sequest HT (3) |  | 3 |  | 665.67993 |  | 0.16 |  | 31.7762 |  | 7239 |  | 7239 |  | TRINITY\_DN0\_c1\_g1\_i14.p1,TRINITY\_DN0\_c1\_g1\_i4.p1 |  | Image |  | Peak List |  |
|  | | | | | | | | | | | | | | | | | | | | | | | | | | | | | | | | | | | | | | | | | | | | | | | | | | | | | |  |
|  | High |  | [A].AKSAGVSVLnSLScKLSKTc.[-] |  | HCD |  | N10(Deamidated); C14(Dehydro); C20(Dehydro) |  | 4.17 |  | 1.3e-3 |  | 1.0e-2 |  | 0.689 |  | 232.34 |  | 1.000 |  |  |  | N10(Deamidated): 100; C14(Dehydro): 100; C20(Dehydro): 100 |  |  |  |  |  |  |  | 0.61 |  | 1 |  | Sequest HT (3) |  | 4 |  | 499.51141 |  | -0.55 |  | 31.8139 |  | 7254 |  | 7254 |  | TRINITY\_DN0\_c1\_g1\_i14.p1,TRINITY\_DN0\_c1\_g1\_i4.p1 |  | Image |  | Peak List |  |
|  | | | | | | | | | | | | | | | | | | | | | | | | | | | | | | | | | | | | | | | | | | | | | | | | | | | | | |  |
|  | High |  | [A].AKSAGVSVLnSLScKLSKTc.[-] |  | HCD |  | N10(Deamidated); C14(Dehydro); C20(Dehydro) |  | 1.91 |  | 2.4e-3 |  | 7.4e-2 |  | 0.25 |  | 180.17 |  | 1.000 |  |  |  | N10(Deamidated): 100; C14(Dehydro): 100; C20(Dehydro): 100 |  |  |  |  |  |  |  | 0.49 |  | 1 |  | Sequest HT (3) |  | 2 |  | 998.01422 |  | -1.88 |  | 31.8360 |  | 7263 |  | 7263 |  | TRINITY\_DN0\_c1\_g1\_i14.p1,TRINITY\_DN0\_c1\_g1\_i4.p1 |  | Image |  | Peak List |  |
|  | | | | | | | | | | | | | | | | | | | | | | | | | | | | | | | | | | | | | | | | | | | | | | | | | | | | | |  |
|  | High |  | [N].AAKSAGVSVLNSLScKLSKTc.[-] |  | HCD |  | C15(Dehydro); C21(Dehydro) |  | 2.57 |  | 3.1e-3 |  | 1.3e-1 |  | 0.131 |  | 188.86 |  | 1.000 |  |  |  | C15(Dehydro): 100; C21(Dehydro): 100 |  |  |  |  |  |  |  | 0.51 |  | 1 |  | Sequest HT (3) |  | 4 |  | 517.02399 |  | -1.89 |  | 31.8385 |  | 7264 |  | 7264 |  | TRINITY\_DN0\_c1\_g1\_i14.p1,TRINITY\_DN0\_c1\_g1\_i4.p1 |  | Image |  | Peak List |  |
|  | | | | | | | | | | | | | | | | | | | | | | | | | | | | | | | | | | | | | | | | | | | | | | | | | | | | | |  |
|  | High |  | [A].GVSVLNSLScKLSKTc.[-] |  | HCD |  | C10(Dehydro); C16(Dehydro) |  | 2.30 |  | 3.1e-3 |  | 9.3e-2 |  | 0.2 |  | 167.86 |  | 1.000 |  |  |  | C10(Dehydro): 100; C16(Dehydro): 100 |  |  |  |  |  |  |  | 0.40 |  | 1 |  | Sequest HT (3) |  | 3 |  | 546.28522 |  | 0.86 |  | 31.8463 |  | 7267 |  | 7267 |  | TRINITY\_DN0\_c1\_g1\_i14.p1,TRINITY\_DN0\_c1\_g1\_i4.p1 |  | Image |  | Peak List |  |
|  | | | | | | | | | | | | | | | | | | | | | | | | | | | | | | | | | | | | | | | | | | | | | | | | | | | | | |  |
|  | High |  | [A].GVSVLNSLScKLSKTc.[-] |  | HCD |  | C10(Dehydro); C16(Dehydro) |  | 2.17 |  | 1.3e-3 |  | 5.4e-2 |  | 0.319 |  | 178.14 |  | 1.000 |  |  |  | C10(Dehydro): 100; C16(Dehydro): 100 |  |  |  |  |  |  |  | 0.28 |  | 1 |  | Sequest HT (3) |  | 2 |  | 818.92401 |  | 0.65 |  | 31.8501 |  | 7270 |  | 7270 |  | TRINITY\_DN0\_c1\_g1\_i14.p1,TRINITY\_DN0\_c1\_g1\_i4.p1 |  | Image |  | Peak List |  |
|  | | | | | | | | | | | | | | | | | | | | | | | | | | | | | | | | | | | | | | | | | | | | | | | | | | | | | |  |
|  | High |  | [N].AAKSAGVSVLnSLScKLSKTc.[-] |  | HCD |  | N11(Deamidated); C15(Dehydro); C21(Dehydro) |  | 4.34 |  | 1.3e-3 |  | 9.8e-4 |  | 1.2 |  | 387.28 |  | 1.000 |  |  |  | N11(Deamidated): 100; C15(Dehydro): 100; C21(Dehydro): 100 |  |  |  |  |  |  |  | 0.62 |  | 1 |  | Sequest HT (3) |  | 3 |  | 689.35901 |  | 0.21 |  | 31.9440 |  | 7307 |  | 7307 |  | TRINITY\_DN0\_c1\_g1\_i14.p1,TRINITY\_DN0\_c1\_g1\_i4.p1 |  | Image |  | Peak List |  |
|  | | | | | | | | | | | | | | | | | | | | | | | | | | | | | | | | | | | | | | | | | | | | | | | | | | | | | |  |
|  | High |  | [N].AAKSAGVSVLnSLScKLSKTc.[-] |  | HCD |  | N11(Deamidated); C15(Dehydro); C21(Dehydro) |  | 4.24 |  | 1.3e-3 |  | 1.7e-4 |  | 1.58 |  | 333.67 |  | 1.000 |  |  |  | N11(Deamidated): 100; C15(Dehydro): 100; C21(Dehydro): 100 |  |  |  |  |  |  |  | 0.71 |  | 1 |  | Sequest HT (3) |  | 2 |  | 1033.53479 |  | 0.13 |  | 31.9696 |  | 7318 |  | 7318 |  | TRINITY\_DN0\_c1\_g1\_i14.p1,TRINITY\_DN0\_c1\_g1\_i4.p1 |  | Image |  | Peak List |  |
|  | | | | | | | | | | | | | | | | | | | | | | | | | | | | | | | | | | | | | | | | | | | | | | | | | | | | | |  |
|  | High |  | [N].AAKSAGVSVLnSLScKLSKTc.[-] |  | HCD |  | N11(Deamidated); C15(Dehydro); C21(Dehydro) |  | 4.12 |  | 1.3e-3 |  | 8.4e-3 |  | 0.73 |  | 243.32 |  | 1.000 |  |  |  | N11(Deamidated): 100; C15(Dehydro): 100; C21(Dehydro): 100 |  |  |  |  |  |  |  | 0.62 |  | 1 |  | Sequest HT (3) |  | 4 |  | 517.27148 |  | 1.00 |  | 31.9889 |  | 7325 |  | 7325 |  | TRINITY\_DN0\_c1\_g1\_i14.p1,TRINITY\_DN0\_c1\_g1\_i4.p1 |  | Image |  | Peak List |  |
|  | | | | | | | | | | | | | | | | | | | | | | | | | | | | | | | | | | | | | | | | | | | | | | | | | | | | | |  |
|  | High |  | [A].LNAAKSAGVSVLNSLScKLSKTc.[-] |  | HCD |  | C17(Dehydro); C23(Dehydro) |  | 5.88 |  | 1.3e-3 |  | 7.4e-4 |  | 1.26 |  | 354.60 |  | 1.000 |  |  |  | C17(Dehydro): 100; C23(Dehydro): 100 |  |  |  |  |  |  |  | 0.60 |  | 1 |  | Sequest HT (3) |  | 3 |  | 764.74030 |  | 0.58 |  | 31.9996 |  | 7330 |  | 7330 |  | TRINITY\_DN0\_c1\_g1\_i14.p1,TRINITY\_DN0\_c1\_g1\_i4.p1 |  | Image |  | Peak List |  |
|  | | | | | | | | | | | | | | | | | | | | | | | | | | | | | | | | | | | | | | | | | | | | | | | | | | | | | |  |
|  | High |  | [A].LNAAKSAGVSVLNSLScKLSKTc.[-] |  | HCD |  | C17(Dehydro); C23(Dehydro) |  | 4.13 |  | 1.3e-3 |  | 4.3e-2 |  | 0.372 |  | 265.10 |  | 1.000 |  |  |  | C17(Dehydro): 100; C23(Dehydro): 100 |  |  |  |  |  |  |  | 0.56 |  | 1 |  | Sequest HT (3) |  | 4 |  | 573.80731 |  | 1.05 |  | 32.0012 |  | 7331 |  | 7331 |  | TRINITY\_DN0\_c1\_g1\_i14.p1,TRINITY\_DN0\_c1\_g1\_i4.p1 |  | Image |  | Peak List |  |
|  | | | | | | | | | | | | | | | | | | | | | | | | | | | | | | | | | | | | | | | | | | | | | | | | | | | | | |  |
|  | High |  | [A].LNAAKSAGVSVLNSLScKLSKTc.[-] |  | HCD |  | C17(Dehydro); C23(Dehydro) |  | 3.28 |  | 1.3e-3 |  | 1.9e-3 |  | 1.06 |  | 265.20 |  | 1.000 |  |  |  | C17(Dehydro): 100; C23(Dehydro): 100 |  |  |  |  |  |  |  | 0.64 |  | 1 |  | Sequest HT (3) |  | 2 |  | 1146.60791 |  | 1.54 |  | 32.0657 |  | 7357 |  | 7357 |  | TRINITY\_DN0\_c1\_g1\_i14.p1,TRINITY\_DN0\_c1\_g1\_i4.p1 |  | Image |  | Peak List |  |
|  | | | | | | | | | | | | | | | | | | | | | | | | | | | | | | | | | | | | | | | | | | | | | | | | | | | | | |  |
|  | High |  | [A].LNAAKSAGVSVLNSLScKLSKTc.[-] |  | HCD |  | C17(Dehydro); C23(Dehydro) |  | 2.78 |  | 3.9e-3 |  | 1.7e-1 |  | 0.061 |  | 188.23 |  | 1.000 |  |  |  | C17(Dehydro): 100; C23(Dehydro): 100 |  |  |  |  |  |  |  | 0.56 |  | 1 |  | Sequest HT (3) |  | 3 |  | 764.74005 |  | 0.26 |  | 32.1748 |  | 7400 |  | 7400 |  | TRINITY\_DN0\_c1\_g1\_i14.p1,TRINITY\_DN0\_c1\_g1\_i4.p1 |  | Image |  | Peak List |  |
|  | | | | | | | | | | | | | | | | | | | | | | | | | | | | | | | | | | | | | | | | | | | | | | | | | | | | | |  |
|  | High |  | [A].LNAAKSAGVSVLNSLScKLSKTc.[-] |  | HCD |  | C17(Dehydro); C23(Dehydro) |  | 2.27 |  | 3.1e-3 |  | 9.4e-2 |  | 0.195 |  | 167.79 |  | 1.000 |  |  |  | C17(Dehydro): 100; C23(Dehydro): 100 |  |  |  |  |  |  |  | 0.48 |  | 1 |  | Sequest HT (3) |  | 4 |  | 573.80707 |  | 0.62 |  | 32.1761 |  | 7401 |  | 7401 |  | TRINITY\_DN0\_c1\_g1\_i14.p1,TRINITY\_DN0\_c1\_g1\_i4.p1 |  | Image |  | Peak List |  |
|  | | | | | | | | | | | | | | | | | | | | | | | | | | | | | | | | | | | | | | | | | | | | | | | | | | | | | |  |
|  | High |  | [S].AGVSVLNSLScKLSKTc.[-] |  | HCD |  | C11(Dehydro); C17(Dehydro) |  | 3.15 |  | 1.3e-3 |  | 4.0e-2 |  | 0.385 |  | 263.36 |  | 1.000 |  |  |  | C11(Dehydro): 100; C17(Dehydro): 100 |  |  |  |  |  |  |  | 0.37 |  | 1 |  | Sequest HT (3) |  | 3 |  | 569.96515 |  | 2.40 |  | 32.4492 |  | 7519 |  | 7519 |  | TRINITY\_DN0\_c1\_g1\_i14.p1,TRINITY\_DN0\_c1\_g1\_i4.p1 |  | Image |  | Peak List |  |
|  | | | | | | | | | | | | | | | | | | | | | | | | | | | | | | | | | | | | | | | | | | | | | | | | | | | | | |  |
|  | High |  | [L].nAAKSAGVSVLNSLScKLSKTc.[-] |  | HCD |  | N1(Deamidated); C16(Dehydro); C22(Dehydro) |  | 1.33 |  | 1.3e-3 |  | 4.6e-2 |  | 0.356 |  | 203.89 |  | 1.000 |  | b1+(2): 187.07; b1+(3): 258.11; b1+(5): 473.24; b1+(6): 544.27; y1+(18): 1794.91; y1+(17): 1707.88; y1+(16): 1636.84; y1+(14): 1480.75; y1+(12): 1294.65; y1+(11): 1181.57; y2+(19): 962.01 |  | N1(Deamidated): 100; C16(Dehydro): 100; C22(Dehydro): 100 |  |  |  |  |  |  |  | 0.30 |  | 1 |  | Sequest HT (3) |  | 2 |  | 1090.55652 |  | 0.37 |  | 32.5941 |  | 7582 |  | 7582 |  | TRINITY\_DN0\_c1\_g1\_i14.p1,TRINITY\_DN0\_c1\_g1\_i4.p1 |  | Image |  | Peak List |  |
|  | | | | | | | | | | | | | | | | | | | | | | | | | | | | | | | | | | | | | | | | | | | | | | | | | | | | | |  |
|  | High |  | [L].nAAKSAGVSVLNSLScKLSKTc.[-] |  | HCD |  | N1(Deamidated); C16(Dehydro); C22(Dehydro) |  | 3.23 |  | 1.3e-3 |  | 4.0e-2 |  | 0.386 |  | 184.05 |  | 1.000 |  | b1+(2): 187.07; b1+(3): 258.11; b1+(7): 601.29; b1+(8): 700.36; y1+(12): 1294.65; y1+(11): 1181.57; y2+(14): 740.88; y2+(13): 697.36; y2+(12): 647.83 |  | N1(Deamidated): 100; C16(Dehydro): 100; C22(Dehydro): 100 |  |  |  |  |  |  |  | 0.44 |  | 1 |  | Sequest HT (3) |  | 4 |  | 545.78247 |  | 1.42 |  | 32.6082 |  | 7589 |  | 7589 |  | TRINITY\_DN0\_c1\_g1\_i14.p1,TRINITY\_DN0\_c1\_g1\_i4.p1 |  | Image |  | Peak List |  |
|  | | | | | | | | | | | | | | | | | | | | | | | | | | | | | | | | | | | | | | | | | | | | | | | | | | | | | |  |
|  | High |  | [G].VSVLnSLScKLSKTc.[-] |  | HCD |  | N5(Deamidated); C9(Dehydro); C15(Dehydro) |  | 1.72 |  | 2.4e-3 |  | 7.7e-2 |  | 0.242 |  | 165.20 |  | 1.000 |  |  |  | N5(Deamidated): 100; C9(Dehydro): 100; C15(Dehydro): 100 |  |  |  |  |  |  |  | 0.14 |  | 1 |  | Sequest HT (3) |  | 3 |  | 527.60553 |  | -0.13 |  | 32.7926 |  | 7671 |  | 7671 |  | TRINITY\_DN0\_c1\_g1\_i14.p1,TRINITY\_DN0\_c1\_g1\_i4.p1 |  | Image |  | Peak List |  |
|  | | | | | | | | | | | | | | | | | | | | | | | | | | | | | | | | | | | | | | | | | | | | | | | | | | | | | |  |
|  | High |  | [K].SAGVSVLNSLScKLSKTc.[-] |  | HCD |  | C12(Dehydro); C18(Dehydro) |  | 3.96 |  | 1.3e-3 |  | 2.3e-2 |  | 0.514 |  | 270.00 |  | 1.000 |  |  |  | C12(Dehydro): 100; C18(Dehydro): 100 |  |  |  |  |  |  |  | 0.41 |  | 1 |  | Sequest HT (3) |  | 3 |  | 598.97388 |  | -0.98 |  | 32.8330 |  | 7687 |  | 7687 |  | TRINITY\_DN0\_c1\_g1\_i14.p1,TRINITY\_DN0\_c1\_g1\_i4.p1 |  | Image |  | Peak List |  |
|  | | | | | | | | | | | | | | | | | | | | | | | | | | | | | | | | | | | | | | | | | | | | | | | | | | | | | |  |
|  | High |  | [K].SAGVSVLNSLScKLSKTc.[-] |  | HCD |  | C12(Dehydro); C18(Dehydro) |  | 2.56 |  | 1.3e-3 |  | 1.4e-2 |  | 0.62 |  | 269.95 |  | 1.000 |  |  |  | C12(Dehydro): 100; C18(Dehydro): 100 |  |  |  |  |  |  |  | 0.46 |  | 1 |  | Sequest HT (3) |  | 2 |  | 897.95679 |  | -1.41 |  | 32.8356 |  | 7689 |  | 7689 |  | TRINITY\_DN0\_c1\_g1\_i14.p1,TRINITY\_DN0\_c1\_g1\_i4.p1 |  | Image |  | Peak List |  |
|  | | | | | | | | | | | | | | | | | | | | | | | | | | | | | | | | | | | | | | | | | | | | | | | | | | | | | |  |
|  | High |  | [G].VSVLnSLScKLSKTc.[-] |  | HCD |  | N5(Deamidated); C9(Dehydro); C15(Dehydro) |  | 2.40 |  | 3.9e-3 |  | 1.6e-1 |  | 0.079 |  | 193.27 |  | 1.000 |  |  |  | N5(Deamidated): 100; C9(Dehydro): 100; C15(Dehydro): 100 |  |  |  |  |  |  |  | 0.22 |  | 1 |  | Sequest HT (3) |  | 3 |  | 527.60529 |  | -0.59 |  | 32.8825 |  | 7707 |  | 7707 |  | TRINITY\_DN0\_c1\_g1\_i14.p1,TRINITY\_DN0\_c1\_g1\_i4.p1 |  | Image |  | Peak List |  |
|  | | | | | | | | | | | | | | | | | | | | | | | | | | | | | | | | | | | | | | | | | | | | | | | | | | | | | |  |
|  | High |  | [K].SAGVSVLNSLScKLSKTc.[-] |  | HCD |  | C12(Dehydro); C18(Dehydro) |  | 2.42 |  | 1.3e-3 |  | 2.3e-2 |  | 0.509 |  | 214.06 |  | 1.000 |  |  |  | C12(Dehydro): 100; C18(Dehydro): 100 |  |  |  |  |  |  |  | 0.50 |  | 1 |  | Sequest HT (3) |  | 2 |  | 897.95807 |  | 0.02 |  | 32.9807 |  | 7748 |  | 7748 |  | TRINITY\_DN0\_c1\_g1\_i14.p1,TRINITY\_DN0\_c1\_g1\_i4.p1 |  | Image |  | Peak List |  |
|  | | | | | | | | | | | | | | | | | | | | | | | | | | | | | | | | | | | | | | | | | | | | | | | | | | | | | |  |
|  | High |  | [L].ALNAAKSAGVSVLNSLScKLSKTc.[-] |  | HCD |  | C18(Dehydro); C24(Dehydro) |  | 5.51 |  | 1.3e-3 |  | 1.5e-2 |  | 0.599 |  | 287.10 |  | 1.000 |  |  |  | C18(Dehydro): 100; C24(Dehydro): 100 |  |  |  |  |  |  |  | 0.63 |  | 1 |  | Sequest HT (3) |  | 4 |  | 591.56671 |  | 1.22 |  | 32.9895 |  | 7753 |  | 7753 |  | TRINITY\_DN0\_c1\_g1\_i14.p1,TRINITY\_DN0\_c1\_g1\_i4.p1 |  | Image |  | Peak List |  |
|  | | | | | | | | | | | | | | | | | | | | | | | | | | | | | | | | | | | | | | | | | | | | | | | | | | | | | |  |
|  | High |  | [L].ALNAAKSAGVSVLNSLScKLSKTc.[-] |  | HCD |  | C18(Dehydro); C24(Dehydro) |  | 5.86 |  | 1.3e-3 |  | 6.8e-4 |  | 1.28 |  | 382.56 |  | 1.000 |  |  |  | C18(Dehydro): 100; C24(Dehydro): 100 |  |  |  |  |  |  |  | 0.69 |  | 1 |  | Sequest HT (3) |  | 3 |  | 788.41943 |  | 0.69 |  | 33.0141 |  | 7762 |  | 7762 |  | TRINITY\_DN0\_c1\_g1\_i14.p1,TRINITY\_DN0\_c1\_g1\_i4.p1 |  | Image |  | Peak List |  |
|  | | | | | | | | | | | | | | | | | | | | | | | | | | | | | | | | | | | | | | | | | | | | | | | | | | | | | |  |
|  | High |  | [L].ALNAAKSAGVSVLNSLScKLSKTc.[-] |  | HCD |  | C18(Dehydro); C24(Dehydro) |  | 3.53 |  | 1.3e-3 |  | 1.8e-4 |  | 1.57 |  | 386.36 |  | 1.000 |  |  |  | C18(Dehydro): 100; C24(Dehydro): 100 |  |  |  |  |  |  |  | 0.67 |  | 1 |  | Sequest HT (3) |  | 2 |  | 1182.12561 |  | 0.77 |  | 33.0340 |  | 7771 |  | 7771 |  | TRINITY\_DN0\_c1\_g1\_i14.p1,TRINITY\_DN0\_c1\_g1\_i4.p1 |  | Image |  | Peak List |  |
|  | | | | | | | | | | | | | | | | | | | | | | | | | | | | | | | | | | | | | | | | | | | | | | | | | | | | | |  |
|  | High |  | [A].LnAAKSAGVSVLNSLScKLSKTc.[-] |  | HCD |  | N2(Deamidated); C17(Dehydro); C23(Dehydro) |  | 4.82 |  | 1.3e-3 |  | 1.1e-3 |  | 1.17 |  | 321.95 |  | 1.000 |  | b1+(2): 229.12; b1+(3): 300.16; b1+(4): 371.19; b1+(5): 499.29; b1+(7): 657.36; b1+(8): 714.38; b1+(9): 813.45; b1+(10): 900.48; b1+(11): 999.55; b1+(12): 1112.63; y1+(14): 1480.75; y1+(12): 1294.65; y1+(11): 1181.57; b2+(10): 450.74; y2+(12): 647.83; y2+(11): 591.29 |  | N2(Deamidated): 100; C17(Dehydro): 100; C23(Dehydro): 100 |  |  |  |  |  |  |  | 0.59 |  | 1 |  | Sequest HT (3) |  | 3 |  | 765.06818 |  | 0.41 |  | 33.0623 |  | 7784 |  | 7784 |  | TRINITY\_DN0\_c1\_g1\_i14.p1,TRINITY\_DN0\_c1\_g1\_i4.p1 |  | Image |  | Peak List |  |
|  | | | | | | | | | | | | | | | | | | | | | | | | | | | | | | | | | | | | | | | | | | | | | | | | | | | | | |  |
|  | High |  | [A].LnAAKSAGVSVLNSLScKLSKTc.[-] |  | HCD |  | N2(Deamidated); C17(Dehydro); C23(Dehydro) |  | 4.59 |  | 1.3e-3 |  | 7.4e-3 |  | 0.756 |  | 249.12 |  | 1.000 |  | b1+(2): 229.12; b1+(3): 300.16; b1+(4): 371.19; b1+(8): 714.38; b1+(9): 813.45; b1+(10): 900.48; b1+(11): 999.55; y1+(12): 1294.65; y1+(11): 1181.57; y2+(14): 740.88; y2+(13): 697.36; y2+(12): 647.83; y2+(11): 591.29 |  | N2(Deamidated): 100; C17(Dehydro): 100; C23(Dehydro): 100 |  |  |  |  |  |  |  | 0.31 |  | 1 |  | Sequest HT (3) |  | 4 |  | 574.05292 |  | 0.35 |  | 33.0648 |  | 7785 |  | 7785 |  | TRINITY\_DN0\_c1\_g1\_i14.p1,TRINITY\_DN0\_c1\_g1\_i4.p1 |  | Image |  | Peak List |  |
|  | | | | | | | | | | | | | | | | | | | | | | | | | | | | | | | | | | | | | | | | | | | | | | | | | | | | | |  |
|  | High |  | [L].ALNAAKSAGVSVLNSLScKLSKTc.[-] |  | HCD |  | C18(Dehydro); C24(Dehydro) |  | 2.28 |  | 1.3e-3 |  | 5.8e-2 |  | 0.306 |  | 157.76 |  | 1.000 |  |  |  | C18(Dehydro): 100; C24(Dehydro): 100 |  |  |  |  |  |  |  | 0.39 |  | 1 |  | Sequest HT (3) |  | 4 |  | 591.56598 |  | -0.02 |  | 33.1433 |  | 7817 |  | 7817 |  | TRINITY\_DN0\_c1\_g1\_i14.p1,TRINITY\_DN0\_c1\_g1\_i4.p1 |  | Image |  | Peak List |  |
|  | | | | | | | | | | | | | | | | | | | | | | | | | | | | | | | | | | | | | | | | | | | | | | | | | | | | | |  |
|  | High |  | [A].LNAAKSAGVSVLnSLScKLSKTc.[-] |  | HCD |  | N13(Deamidated); C17(Dehydro); C23(Dehydro) |  | 3.19 |  | 1.3e-3 |  | 4.1e-2 |  | 0.382 |  | 226.33 |  | 1.000 |  | b1+(2): 228.13; b1+(3): 299.17; b1+(8): 713.39; b1+(9): 812.46; b1+(10): 899.49; y1+(14): 1481.73; y1+(12): 1295.63; y1+(11): 1182.55; y2+(14): 741.37; y2+(13): 697.85; y2+(12): 648.32 |  | N13(Deamidated): 100; C17(Dehydro): 100; C23(Dehydro): 100 |  |  |  |  |  |  |  | 0.18 |  | 1 |  | Sequest HT (3) |  | 4 |  | 574.05341 |  | 1.21 |  | 33.2202 |  | 7852 |  | 7852 |  | TRINITY\_DN0\_c1\_g1\_i14.p1,TRINITY\_DN0\_c1\_g1\_i4.p1 |  | Image |  | Peak List |  |
|  | | | | | | | | | | | | | | | | | | | | | | | | | | | | | | | | | | | | | | | | | | | | | | | | | | | | | |  |
|  | High |  | [A].LNAAKSAGVSVLnSLScKLSKTc.[-] |  | HCD |  | N13(Deamidated); C17(Dehydro); C23(Dehydro) |  | 5.40 |  | 1.3e-3 |  | 2.3e-3 |  | 1.01 |  | 287.38 |  | 1.000 |  | b1+(2): 228.13; b1+(3): 299.17; b1+(4): 370.21; b1+(6): 585.34; b1+(7): 656.37; b1+(8): 713.39; b1+(9): 812.46; b1+(10): 899.49; b1+(11): 998.56; y1+(14): 1481.73; y1+(13): 1394.70; y1+(12): 1295.63; y1+(11): 1182.55; b2+(10): 450.25; y2+(12): 648.32; y2+(11): 591.78 |  | N13(Deamidated): 100; C17(Dehydro): 100; C23(Dehydro): 100 |  |  |  |  |  |  |  | 0.54 |  | 1 |  | Sequest HT (3) |  | 3 |  | 765.06873 |  | 1.13 |  | 33.2221 |  | 7853 |  | 7853 |  | TRINITY\_DN0\_c1\_g1\_i14.p1,TRINITY\_DN0\_c1\_g1\_i4.p1 |  | Image |  | Peak List |  |
|  | | | | | | | | | | | | | | | | | | | | | | | | | | | | | | | | | | | | | | | | | | | | | | | | | | | | | |  |
|  | High |  | [A].LNAAKSAGVSVLnSLScKLSKTc.[-] |  | HCD |  | N13(Deamidated); C17(Dehydro); C23(Dehydro) |  | 3.07 |  | 1.3e-3 |  | 8.6e-3 |  | 0.725 |  | 274.92 |  | 1.000 |  | b1+(2): 228.13; b1+(3): 299.17; b1+(4): 370.21; b1+(6): 585.34; b1+(7): 656.37; b1+(9): 812.46; b1+(10): 899.49; b1+(11): 998.56; y1+(14): 1481.73; y1+(12): 1295.63; y1+(11): 1182.55; b2+(10): 450.25; y2+(11): 591.78 |  | N13(Deamidated): 100; C17(Dehydro): 100; C23(Dehydro): 100 |  |  |  |  |  |  |  | 0.46 |  | 1 |  | Sequest HT (3) |  | 3 |  | 765.06775 |  | -0.15 |  | 33.2783 |  | 7877 |  | 7877 |  | TRINITY\_DN0\_c1\_g1\_i14.p1,TRINITY\_DN0\_c1\_g1\_i4.p1 |  | Image |  | Peak List |  |
|  | | | | | | | | | | | | | | | | | | | | | | | | | | | | | | | | | | | | | | | | | | | | | | | | | | | | | |  |
|  | High |  | [A].GVSVLnSLScKLSKTc.[-] |  | HCD |  | N6(Deamidated); C10(Dehydro); C16(Dehydro) |  | 2.93 |  | 1.3e-3 |  | 3.8e-2 |  | 0.398 |  | 233.33 |  | 1.000 |  |  |  | N6(Deamidated): 100; C10(Dehydro): 100; C16(Dehydro): 100 |  |  |  |  |  |  |  | 0.34 |  | 1 |  | Sequest HT (3) |  | 2 |  | 819.41455 |  | -1.15 |  | 33.2837 |  | 7880 |  | 7880 |  | TRINITY\_DN0\_c1\_g1\_i14.p1,TRINITY\_DN0\_c1\_g1\_i4.p1 |  | Image |  | Peak List |  |
|  | | | | | | | | | | | | | | | | | | | | | | | | | | | | | | | | | | | | | | | | | | | | | | | | | | | | | |  |
|  | High |  | [A].GVSVLnSLScKLSKTc.[-] |  | HCD |  | N6(Deamidated); C10(Dehydro); C16(Dehydro) |  | 3.32 |  | 3.1e-3 |  | 9.1e-2 |  | 0.203 |  | 219.55 |  | 1.000 |  |  |  | N6(Deamidated): 100; C10(Dehydro): 100; C16(Dehydro): 100 |  |  |  |  |  |  |  | 0.48 |  | 1 |  | Sequest HT (3) |  | 3 |  | 546.61334 |  | 1.08 |  | 33.3152 |  | 7893 |  | 7893 |  | TRINITY\_DN0\_c1\_g1\_i14.p1,TRINITY\_DN0\_c1\_g1\_i4.p1 |  | Image |  | Peak List |  |
|  | | | | | | | | | | | | | | | | | | | | | | | | | | | | | | | | | | | | | | | | | | | | | | | | | | | | | |  |
|  | High |  | [S].AGVSVLnSLScKLSKTc.[-] |  | HCD |  | N7(Deamidated); C11(Dehydro); C17(Dehydro) |  | 2.44 |  | 1.3e-3 |  | 6.9e-2 |  | 0.267 |  | 182.18 |  | 1.000 |  |  |  | N7(Deamidated): 100; C11(Dehydro): 100; C17(Dehydro): 100 |  |  |  |  |  |  |  | 0.24 |  | 1 |  | Sequest HT (3) |  | 3 |  | 570.29224 |  | 0.78 |  | 33.7249 |  | 8061 |  | 8061 |  | TRINITY\_DN0\_c1\_g1\_i14.p1,TRINITY\_DN0\_c1\_g1\_i4.p1 |  | Image |  | Peak List |  |
|  | | | | | | | | | | | | | | | | | | | | | | | | | | | | | | | | | | | | | | | | | | | | | | | | | | | | | |  |
|  | High |  | [S].AGVSVLnSLScKLSKTc.[-] |  | HCD |  | N7(Deamidated); C11(Dehydro); C17(Dehydro) |  | 2.68 |  | 1.3e-3 |  | 1.4e-2 |  | 0.62 |  | 272.13 |  | 1.000 |  |  |  | N7(Deamidated): 100; C11(Dehydro): 100; C17(Dehydro): 100 |  |  |  |  |  |  |  | 0.25 |  | 1 |  | Sequest HT (3) |  | 2 |  | 854.93384 |  | -0.25 |  | 33.7374 |  | 8069 |  | 8069 |  | TRINITY\_DN0\_c1\_g1\_i14.p1,TRINITY\_DN0\_c1\_g1\_i4.p1 |  | Image |  | Peak List |  |
|  | | | | | | | | | | | | | | | | | | | | | | | | | | | | | | | | | | | | | | | | | | | | | | | | | | | | | |  |
|  | High |  | [L].nAAKSAGVSVLnSLScKLSKTc.[-] |  | HCD |  | N1(Deamidated); N12(Deamidated); C16(Dehydro); C22(Dehydro) |  | 5.36 |  | 1.3e-3 |  | 1.3e-3 |  | 1.13 |  | 423.62 |  | 1.000 |  |  |  | N1(Deamidated): 100; N12(Deamidated): 100; C16(Dehydro): 100; C22(Dehydro): 100 |  |  |  |  |  |  |  | 0.54 |  | 1 |  | Sequest HT (3) |  | 3 |  | 727.70099 |  | -0.26 |  | 33.8687 |  | 8128 |  | 8128 |  | TRINITY\_DN0\_c1\_g1\_i14.p1,TRINITY\_DN0\_c1\_g1\_i4.p1 |  | Image |  | Peak List |  |
|  | | | | | | | | | | | | | | | | | | | | | | | | | | | | | | | | | | | | | | | | | | | | | | | | | | | | | |  |
|  | High |  | [K].SAGVSVLnSLScKLSKTc.[-] |  | HCD |  | N8(Deamidated); C12(Dehydro); C18(Dehydro) |  | 4.19 |  | 1.3e-3 |  | 1.3e-2 |  | 0.636 |  | 267.13 |  | 1.000 |  |  |  | N8(Deamidated): 100; C12(Dehydro): 100; C18(Dehydro): 100 |  |  |  |  |  |  |  | 0.54 |  | 1 |  | Sequest HT (3) |  | 3 |  | 599.30249 |  | 0.04 |  | 34.1470 |  | 8251 |  | 8251 |  | TRINITY\_DN0\_c1\_g1\_i14.p1,TRINITY\_DN0\_c1\_g1\_i4.p1 |  | Image |  | Peak List |  |
|  | | | | | | | | | | | | | | | | | | | | | | | | | | | | | | | | | | | | | | | | | | | | | | | | | | | | | |  |
|  | High |  | [K].SAGVSVLnSLScKLSKTc.[-] |  | HCD |  | N8(Deamidated); C12(Dehydro); C18(Dehydro) |  | 3.02 |  | 1.3e-3 |  | 1.7e-2 |  | 0.574 |  | 281.62 |  | 1.000 |  |  |  | N8(Deamidated): 100; C12(Dehydro): 100; C18(Dehydro): 100 |  |  |  |  |  |  |  | 0.34 |  | 1 |  | Sequest HT (3) |  | 2 |  | 898.45007 |  | 0.01 |  | 34.1484 |  | 8252 |  | 8252 |  | TRINITY\_DN0\_c1\_g1\_i14.p1,TRINITY\_DN0\_c1\_g1\_i4.p1 |  | Image |  | Peak List |  |
|  | | | | | | | | | | | | | | | | | | | | | | | | | | | | | | | | | | | | | | | | | | | | | | | | | | | | | |  |
|  | High |  | [L].ALNAAKSAGVSVLnSLScKLSKTc.[-] |  | HCD |  | N14(Deamidated); C18(Dehydro); C24(Dehydro) |  | 3.57 |  | 1.3e-3 |  | 2.6e-2 |  | 0.478 |  | 200.03 |  | 1.000 |  | b1+(3): 299.17; b1+(4): 370.21; b1+(5): 441.25; b1+(7): 656.37; b1+(9): 784.43; b1+(10): 883.50; b1+(11): 970.53; y1+(12): 1295.63; y1+(11): 1182.55; y2+(12): 648.32 |  | N14(Deamidated): 100; C18(Dehydro): 100; C24(Dehydro): 100 |  |  |  |  |  |  |  | 0.45 |  | 1 |  | Sequest HT (3) |  | 3 |  | 788.74774 |  | 1.07 |  | 34.1944 |  | 8273 |  | 8273 |  | TRINITY\_DN0\_c1\_g1\_i14.p1,TRINITY\_DN0\_c1\_g1\_i4.p1 |  | Image |  | Peak List |  |
|  | | | | | | | | | | | | | | | | | | | | | | | | | | | | | | | | | | | | | | | | | | | | | | | | | | | | | |  |
|  | High |  | [L].ALNAAKSAGVSVLnSLScKLSKTc.[-] |  | HCD |  | N14(Deamidated); C18(Dehydro); C24(Dehydro) |  | 4.94 |  | 1.3e-3 |  | 8.6e-4 |  | 1.23 |  | 348.77 |  | 1.000 |  | b1+(3): 299.17; b1+(4): 370.21; b1+(5): 441.25; b1+(6): 569.34; b1+(7): 656.37; b1+(8): 727.41; b1+(9): 784.43; b1+(10): 883.50; b1+(11): 970.53; b1+(12): 1069.60; y1+(17): 1708.86; y1+(14): 1481.73; y1+(12): 1295.63; y1+(11): 1182.55; b2+(10): 442.25; b2+(11): 485.77; y2+(12): 648.32 |  | N14(Deamidated): 100; C18(Dehydro): 100; C24(Dehydro): 100 |  |  |  |  |  |  |  | 0.60 |  | 1 |  | Sequest HT (3) |  | 3 |  | 788.74774 |  | 1.07 |  | 34.2001 |  | 8277 |  | 8277 |  | TRINITY\_DN0\_c1\_g1\_i14.p1,TRINITY\_DN0\_c1\_g1\_i4.p1 |  | Image |  | Peak List |  |
|  | | | | | | | | | | | | | | | | | | | | | | | | | | | | | | | | | | | | | | | | | | | | | | | | | | | | | |  |
|  | High |  | [L].ALNAAKSAGVSVLnSLScKLSKTc.[-] |  | HCD |  | N14(Deamidated); C18(Dehydro); C24(Dehydro) |  | 3.41 |  | 1.3e-3 |  | 3.2e-2 |  | 0.439 |  | 221.18 |  | 1.000 |  | b1+(3): 299.17; b1+(4): 370.21; b1+(5): 441.25; b1+(7): 656.37; b1+(9): 784.43; y1+(14): 1481.73; y1+(12): 1295.63; y1+(11): 1182.55; y2+(14): 741.37; y2+(12): 648.32 |  | N14(Deamidated): 100; C18(Dehydro): 100; C24(Dehydro): 100 |  |  |  |  |  |  |  | 0.35 |  | 1 |  | Sequest HT (3) |  | 4 |  | 591.81311 |  | 1.89 |  | 34.2014 |  | 8278 |  | 8278 |  | TRINITY\_DN0\_c1\_g1\_i14.p1,TRINITY\_DN0\_c1\_g1\_i4.p1 |  | Image |  | Peak List |  |
|  | | | | | | | | | | | | | | | | | | | | | | | | | | | | | | | | | | | | | | | | | | | | | | | | | | | | | |  |
|  | High |  | [K].SAGVSVLnSLScKLSKTc.[-] |  | HCD |  | N8(Deamidated); C12(Dehydro); C18(Dehydro) |  | 2.53 |  | 2.4e-3 |  | 8.0e-2 |  | 0.233 |  | 192.81 |  | 1.000 |  |  |  | N8(Deamidated): 100; C12(Dehydro): 100; C18(Dehydro): 100 |  |  |  |  |  |  |  | 0.50 |  | 1 |  | Sequest HT (3) |  | 3 |  | 599.30310 |  | 1.06 |  | 34.3320 |  | 8335 |  | 8335 |  | TRINITY\_DN0\_c1\_g1\_i14.p1,TRINITY\_DN0\_c1\_g1\_i4.p1 |  | Image |  | Peak List |  |
|  | | | | | | | | | | | | | | | | | | | | | | | | | | | | | | | | | | | | | | | | | | | | | | | | | | | | | |  |
|  | High |  | [L].ALnAAKSAGVSVLNSLScKLSKTc.[-] |  | HCD |  | N3(Deamidated); C18(Dehydro); C24(Dehydro) |  | 4.72 |  | 1.3e-3 |  | 4.4e-3 |  | 0.869 |  | 227.07 |  | 1.000 |  | b1+(3): 300.16; b1+(4): 371.19; b1+(5): 442.23; b1+(9): 785.42; y1+(14): 1480.75; y1+(12): 1294.65; y1+(11): 1181.57; b2+(11): 486.26; y2+(18): 897.96; y2+(14): 740.88; y2+(12): 647.83 |  | N3(Deamidated): 100; C18(Dehydro): 100; C24(Dehydro): 100 |  |  |  |  |  |  |  | 0.40 |  | 1 |  | Sequest HT (3) |  | 4 |  | 591.81262 |  | 1.06 |  | 34.4481 |  | 8389 |  | 8389 |  | TRINITY\_DN0\_c1\_g1\_i14.p1,TRINITY\_DN0\_c1\_g1\_i4.p1 |  | Image |  | Peak List |  |
|  | | | | | | | | | | | | | | | | | | | | | | | | | | | | | | | | | | | | | | | | | | | | | | | | | | | | | |  |
|  | High |  | [L].ALnAAKSAGVSVLNSLScKLSKTc.[-] |  | HCD |  | N3(Deamidated); C18(Dehydro); C24(Dehydro) |  | 5.44 |  | 1.3e-3 |  | 1.9e-4 |  | 1.56 |  | 369.77 |  | 1.000 |  | b1+(3): 300.16; b1+(4): 371.19; b1+(5): 442.23; b1+(6): 570.32; b1+(7): 657.36; b1+(8): 728.39; b1+(9): 785.42; b1+(10): 884.48; b1+(11): 971.52; b1+(12): 1070.58; y1+(14): 1480.75; y1+(13): 1393.72; y1+(12): 1294.65; y1+(11): 1181.57; b2+(11): 486.26; y2+(17): 854.44; y2+(14): 740.88; y2+(12): 647.83; y2+(11): 591.29 |  | N3(Deamidated): 100; C18(Dehydro): 100; C24(Dehydro): 100 |  |  |  |  |  |  |  | 0.60 |  | 1 |  | Sequest HT (3) |  | 3 |  | 788.74683 |  | -0.09 |  | 34.4782 |  | 8402 |  | 8402 |  | TRINITY\_DN0\_c1\_g1\_i14.p1,TRINITY\_DN0\_c1\_g1\_i4.p1 |  | Image |  | Peak List |  |
|  | | | | | | | | | | | | | | | | | | | | | | | | | | | | | | | | | | | | | | | | | | | | | | | | | | | | | |  |
|  | High |  | [L].ALnAAKSAGVSVLNSLScKLSKTc.[-] |  | HCD |  | N3(Deamidated); C18(Dehydro); C24(Dehydro) |  | 2.36 |  | 3.1e-3 |  | 1.0e-1 |  | 0.181 |  | 149.63 |  | 1.000 |  | b1+(3): 300.16; b1+(4): 371.19; b1+(7): 657.36; b1+(9): 785.42; y1+(12): 1294.65; y1+(11): 1181.57; y2+(14): 740.88; y2+(12): 647.83 |  | N3(Deamidated): 100; C18(Dehydro): 100; C24(Dehydro): 100 |  |  |  |  |  |  |  | 0.36 |  | 1 |  | Sequest HT (3) |  | 4 |  | 591.81134 |  | -1.10 |  | 34.5901 |  | 8454 |  | 8454 |  | TRINITY\_DN0\_c1\_g1\_i14.p1,TRINITY\_DN0\_c1\_g1\_i4.p1 |  | Image |  | Peak List |  |
|  | | | | | | | | | | | | | | | | | | | | | | | | | | | | | | | | | | | | | | | | | | | | | | | | | | | | | |  |
|  | High |  | [F].KNLALNAAKSAGVSVLNSLScKLSKTc.[-] |  | HCD |  | C21(Dehydro); C27(Dehydro) |  | 4.35 |  | 1.3e-3 |  | 1.6e-2 |  | 0.59 |  | 241.21 |  | 1.000 |  |  |  | C21(Dehydro): 100; C27(Dehydro): 100 |  |  |  |  |  |  |  | 0.61 |  | 1 |  | Sequest HT (3) |  | 4 |  | 680.37048 |  | -1.46 |  | 34.6303 |  | 8473 |  | 8473 |  | TRINITY\_DN0\_c1\_g1\_i14.p1,TRINITY\_DN0\_c1\_g1\_i4.p1 |  | Image |  | Peak List |  |
|  | | | | | | | | | | | | | | | | | | | | | | | | | | | | | | | | | | | | | | | | | | | | | | | | | | | | | |  |
|  | High |  | [F].KNLALNAAKSAGVSVLNSLScKLSKTc.[-] |  | HCD |  | C21(Dehydro); C27(Dehydro) |  | 5.32 |  | 1.3e-3 |  | 1.6e-4 |  | 1.59 |  | 456.19 |  | 1.000 |  |  |  | C21(Dehydro): 100; C27(Dehydro): 100 |  |  |  |  |  |  |  | 0.57 |  | 1 |  | Sequest HT (3) |  | 3 |  | 906.82446 |  | -1.93 |  | 34.6349 |  | 8476 |  | 8476 |  | TRINITY\_DN0\_c1\_g1\_i14.p1,TRINITY\_DN0\_c1\_g1\_i4.p1 |  | Image |  | Peak List |  |
|  | | | | | | | | | | | | | | | | | | | | | | | | | | | | | | | | | | | | | | | | | | | | | | | | | | | | | |  |
|  | High |  | [F].KNLALNAAKSAGVSVLNSLScKLSKTc.[-] |  | HCD |  | C21(Dehydro); C27(Dehydro) |  | 3.89 |  | 1.3e-3 |  | 3.4e-2 |  | 0.423 |  | 203.94 |  | 1.000 |  |  |  | C21(Dehydro): 100; C27(Dehydro): 100 |  |  |  |  |  |  |  | 0.52 |  | 1 |  | Sequest HT (3) |  | 5 |  | 544.49731 |  | -2.43 |  | 34.6439 |  | 8479 |  | 8479 |  | TRINITY\_DN0\_c1\_g1\_i14.p1,TRINITY\_DN0\_c1\_g1\_i4.p1 |  | Image |  | Peak List |  |
|  | | | | | | | | | | | | | | | | | | | | | | | | | | | | | | | | | | | | | | | | | | | | | | | | | | | | | |  |
|  | High |  | [F].KNLALNAAKSAGVSVLNSLScKLSKTc.[-] |  | HCD |  | C21(Dehydro); C27(Dehydro) |  | 2.85 |  | 2.4e-3 |  | 7.4e-2 |  | 0.252 |  | 195.99 |  | 1.000 |  |  |  | C21(Dehydro): 100; C27(Dehydro): 100 |  |  |  |  |  |  |  | 0.59 |  | 1 |  | Sequest HT (3) |  | 4 |  | 680.37109 |  | -0.56 |  | 34.8036 |  | 8550 |  | 8550 |  | TRINITY\_DN0\_c1\_g1\_i14.p1,TRINITY\_DN0\_c1\_g1\_i4.p1 |  | Image |  | Peak List |  |
|  | | | | | | | | | | | | | | | | | | | | | | | | | | | | | | | | | | | | | | | | | | | | | | | | | | | | | |  |
|  | High |  | [F].KNLALNAAKSAGVSVLNSLScKLSKTc.[-] |  | HCD |  | C21(Dehydro); C27(Dehydro) |  | 2.73 |  | 1.3e-3 |  | 2.3e-2 |  | 0.513 |  | 258.49 |  | 1.000 |  |  |  | C21(Dehydro): 100; C27(Dehydro): 100 |  |  |  |  |  |  |  | 0.61 |  | 1 |  | Sequest HT (3) |  | 3 |  | 906.82501 |  | -1.32 |  | 34.8061 |  | 8552 |  | 8552 |  | TRINITY\_DN0\_c1\_g1\_i14.p1,TRINITY\_DN0\_c1\_g1\_i4.p1 |  | Image |  | Peak List |  |
|  | | | | | | | | | | | | | | | | | | | | | | | | | | | | | | | | | | | | | | | | | | | | | | | | | | | | | |  |
|  | High |  | [F].KNLALNAAKSAGVSVLNSLScKLSKTc.[-] |  | HCD |  | C21(Dehydro); C27(Dehydro) |  | 4.01 |  | 1.3e-3 |  | 2.1e-2 |  | 0.531 |  | 241.21 |  | 1.000 |  |  |  | C21(Dehydro): 100; C27(Dehydro): 100 |  |  |  |  |  |  |  | 0.43 |  | 1 |  | Sequest HT (3) |  | 5 |  | 544.49872 |  | 0.15 |  | 34.8276 |  | 8562 |  | 8562 |  | TRINITY\_DN0\_c1\_g1\_i14.p1,TRINITY\_DN0\_c1\_g1\_i4.p1 |  | Image |  | Peak List |  |
|  | | | | | | | | | | | | | | | | | | | | | | | | | | | | | | | | | | | | | | | | | | | | | | | | | | | | | |  |
|  | High |  | [D].TFKNLALNAAKSAGVSVLNSLScKLSKTc.[-] |  | HCD |  | C23(Dehydro); C29(Dehydro) |  | 2.96 |  | 3.9e-3 |  | 1.6e-1 |  | 0.069 |  | 129.67 |  | 1.000 |  |  |  | C23(Dehydro): 100; C29(Dehydro): 100 |  |  |  |  |  |  |  | 0.50 |  | 1 |  | Sequest HT (3) |  | 3 |  | 989.53021 |  | -1.38 |  | 35.0682 |  | 8665 |  | 8665 |  | TRINITY\_DN0\_c1\_g1\_i14.p1,TRINITY\_DN0\_c1\_g1\_i4.p1 |  | Image |  | Peak List |  |
|  | | | | | | | | | | | | | | | | | | | | | | | | | | | | | | | | | | | | | | | | | | | | | | | | | | | | | |  |
|  | High |  | [D].TFKNLALNAAKSAGVSVLnSLScKLSKTc.[-] |  | HCD |  | N19(Deamidated); C23(Dehydro); C29(Dehydro) |  | 3.63 |  | 3.1e-3 |  | 1.3e-1 |  | 0.118 |  | 136.17 |  | 1.000 |  | b1+(10): 1044.58; y1+(12): 1295.63; y1+(11): 1182.55; b2+(14): 694.39; y2+(19): 962.50 |  | N19(Deamidated): 100; C23(Dehydro): 100; C29(Dehydro): 100 |  |  |  |  |  |  |  | 0.28 |  | 1 |  | Sequest HT (3) |  | 5 |  | 594.31970 |  | 1.76 |  | 35.5127 |  | 8877 |  | 8877 |  | TRINITY\_DN0\_c1\_g1\_i14.p1,TRINITY\_DN0\_c1\_g1\_i4.p1 |  | Image |  | Peak List |  |
|  | | | | | | | | | | | | | | | | | | | | | | | | | | | | | | | | | | | | | | | | | | | | | | | | | | | | | |  |
|  | High |  | [N].LALNAAKSAGVSVLNSLScKLSKTc.[-] |  | HCD |  | C19(Dehydro); C25(Dehydro) |  | 3.95 |  | 1.3e-3 |  | 2.3e-3 |  | 1.01 |  | 303.47 |  | 1.000 |  |  |  | C19(Dehydro): 100; C25(Dehydro): 100 |  |  |  |  |  |  |  | 0.58 |  | 1 |  | Sequest HT (3) |  | 3 |  | 826.11292 |  | -0.81 |  | 35.9931 |  | 9104 |  | 9104 |  | TRINITY\_DN0\_c1\_g1\_i14.p1,TRINITY\_DN0\_c1\_g1\_i4.p1 |  | Image |  | Peak List |  |
|  | | | | | | | | | | | | | | | | | | | | | | | | | | | | | | | | | | | | | | | | | | | | | | | | | | | | | |  |
|  | High |  | [N].LALNAAKSAGVSVLNSLScKLSKTc.[-] |  | HCD |  | C19(Dehydro); C25(Dehydro) |  | 4.05 |  | 1.3e-3 |  | 4.7e-2 |  | 0.35 |  | 152.28 |  | 1.000 |  |  |  | C19(Dehydro): 100; C25(Dehydro): 100 |  |  |  |  |  |  |  | 0.55 |  | 1 |  | Sequest HT (3) |  | 4 |  | 619.83765 |  | 1.04 |  | 35.9944 |  | 9105 |  | 9105 |  | TRINITY\_DN0\_c1\_g1\_i14.p1,TRINITY\_DN0\_c1\_g1\_i4.p1 |  | Image |  | Peak List |  |
|  | | | | | | | | | | | | | | | | | | | | | | | | | | | | | | | | | | | | | | | | | | | | | | | | | | | | | |  |
|  | High |  | [N].LALNAAKSAGVSVLNSLScKLSKTc.[-] |  | HCD |  | C19(Dehydro); C25(Dehydro) |  | 3.18 |  | 3.1e-3 |  | 1.1e-1 |  | 0.164 |  | 132.72 |  | 1.000 |  |  |  | C19(Dehydro): 100; C25(Dehydro): 100 |  |  |  |  |  |  |  | 0.56 |  | 1 |  | Sequest HT (3) |  | 4 |  | 619.83716 |  | 0.25 |  | 36.1433 |  | 9174 |  | 9174 |  | TRINITY\_DN0\_c1\_g1\_i14.p1,TRINITY\_DN0\_c1\_g1\_i4.p1 |  | Image |  | Peak List |  |
|  | | | | | | | | | | | | | | | | | | | | | | | | | | | | | | | | | | | | | | | | | | | | | | | | | | | | | |  |
|  | High |  | [N].LALNAAKSAGVSVLNSLScKLSKTc.[-] |  | HCD |  | C19(Dehydro); C25(Dehydro) |  | 3.68 |  | 1.3e-3 |  | 6.0e-3 |  | 0.802 |  | 277.12 |  | 1.000 |  |  |  | C19(Dehydro): 100; C25(Dehydro): 100 |  |  |  |  |  |  |  | 0.67 |  | 1 |  | Sequest HT (3) |  | 3 |  | 826.11292 |  | -0.81 |  | 36.1445 |  | 9175 |  | 9175 |  | TRINITY\_DN0\_c1\_g1\_i14.p1,TRINITY\_DN0\_c1\_g1\_i4.p1 |  | Image |  | Peak List |  |
|  | | | | | | | | | | | | | | | | | | | | | | | | | | | | | | | | | | | | | | | | | | | | | | | | | | | | | |  |
|  | High |  | [R].GLLDTFKnL.[A] |  | HCD |  | N8(Deamidated) |  | 1.87 |  | 1.3e-3 |  | 2.1e-2 |  | 0.527 |  | 195.46 |  | 1.000 |  |  |  | N8(Deamidated): 100 |  |  |  |  |  |  |  | 0.24 |  | 1 |  | Sequest HT (3) |  | 2 |  | 511.28177 |  | -0.17 |  | 36.5663 |  | 9388 |  | 9388 |  | TRINITY\_DN24401\_c0\_g1\_i2.p14; TRINITY\_DN6490\_c1\_g1\_i1.p1\_ORF1; TRINITY\_DN0\_c1\_g1\_i14.p1,TRINITY\_DN0\_c1\_g1\_i4.p1; TRINITY\_DN6490\_c1\_g1\_i1.p1 |  | Image |  | Peak List |  |
|  | | | | | | | | | | | | | | | | | | | | | | | | | | | | | | | | | | | | | | | | | | | | | | | | | | | | | |  |
|  | High |  | [R].GLLDTFKnL.[A] |  | HCD |  | N8(Deamidated) |  | 1.25 |  | 3.1e-3 |  | 1.4e-1 |  | 0.103 |  | 144.10 |  | 1.000 |  |  |  | N8(Deamidated): 100 |  |  |  |  |  |  |  | 0.04 |  | 1 |  | Sequest HT (3) |  | 2 |  | 511.28177 |  | -0.17 |  | 36.5688 |  | 9390 |  | 9390 |  | TRINITY\_DN24401\_c0\_g1\_i2.p14; TRINITY\_DN6490\_c1\_g1\_i1.p1\_ORF1; TRINITY\_DN0\_c1\_g1\_i14.p1,TRINITY\_DN0\_c1\_g1\_i4.p1; TRINITY\_DN6490\_c1\_g1\_i1.p1 |  | Image |  | Peak List |  |
|  | | | | | | | | | | | | | | | | | | | | | | | | | | | | | | | | | | | | | | | | | | | | | | | | | | | | | |  |
|  | High |  | [T].FKNLALNAAKSAGVSVLNSLScKLSKTc.[-] |  | HCD |  | C22(Dehydro); C28(Dehydro) |  | 4.55 |  | 1.3e-3 |  | 1.7e-2 |  | 0.573 |  | 258.72 |  | 1.000 |  |  |  | C22(Dehydro): 100; C28(Dehydro): 100 |  |  |  |  |  |  |  | 0.45 |  | 1 |  | Sequest HT (3) |  | 5 |  | 573.91266 |  | 0.59 |  | 36.9059 |  | 9548 |  | 9548 |  | TRINITY\_DN0\_c1\_g1\_i14.p1,TRINITY\_DN0\_c1\_g1\_i4.p1 |  | Image |  | Peak List |  |
|  | | | | | | | | | | | | | | | | | | | | | | | | | | | | | | | | | | | | | | | | | | | | | | | | | | | | | |  |
|  | High |  | [T].FKNLALNAAKSAGVSVLNSLScKLSKTc.[-] |  | HCD |  | C22(Dehydro); C28(Dehydro) |  | 5.12 |  | 1.3e-3 |  | 2.1e-4 |  | 1.53 |  | 455.60 |  | 1.000 |  |  |  | C22(Dehydro): 100; C28(Dehydro): 100 |  |  |  |  |  |  |  | 0.67 |  | 1 |  | Sequest HT (3) |  | 3 |  | 955.84778 |  | -1.30 |  | 36.9236 |  | 9557 |  | 9557 |  | TRINITY\_DN0\_c1\_g1\_i14.p1,TRINITY\_DN0\_c1\_g1\_i4.p1 |  | Image |  | Peak List |  |
|  | | | | | | | | | | | | | | | | | | | | | | | | | | | | | | | | | | | | | | | | | | | | | | | | | | | | | |  |
|  | High |  | [D].TFKNLALNAAKSAGVSVLNSLScKLSKTc.[-] |  | HCD |  | C23(Dehydro); C29(Dehydro) |  | 6.20 |  | 1.3e-3 |  | 9.2e-4 |  | 1.21 |  | 313.38 |  | 1.000 |  |  |  | C23(Dehydro): 100; C29(Dehydro): 100 |  |  |  |  |  |  |  | 0.66 |  | 1 |  | Sequest HT (3) |  | 4 |  | 742.40198 |  | 1.99 |  | 37.5247 |  | 9858 |  | 9858 |  | TRINITY\_DN0\_c1\_g1\_i14.p1,TRINITY\_DN0\_c1\_g1\_i4.p1 |  | Image |  | Peak List |  |
|  | | | | | | | | | | | | | | | | | | | | | | | | | | | | | | | | | | | | | | | | | | | | | | | | | | | | | |  |
|  | High |  | [D].TFKNLALNAAKSAGVSVLNSLScKLSKTc.[-] |  | HCD |  | C23(Dehydro); C29(Dehydro) |  | 6.34 |  | 1.3e-3 |  | 7.8e-4 |  | 1.25 |  | 356.69 |  | 1.000 |  |  |  | C23(Dehydro): 100; C29(Dehydro): 100 |  |  |  |  |  |  |  | 0.71 |  | 1 |  | Sequest HT (3) |  | 3 |  | 989.53156 |  | -0.02 |  | 37.5272 |  | 9860 |  | 9860 |  | TRINITY\_DN0\_c1\_g1\_i14.p1,TRINITY\_DN0\_c1\_g1\_i4.p1 |  | Image |  | Peak List |  |
|  | | | | | | | | | | | | | | | | | | | | | | | | | | | | | | | | | | | | | | | | | | | | | | | | | | | | | |  |
|  | High |  | [D].TFKNLALNAAKSAGVSVLNSLScKLSKTc.[-] |  | HCD |  | C23(Dehydro); C29(Dehydro) |  | 6.40 |  | 1.3e-3 |  | 4.7e-3 |  | 0.856 |  | 237.34 |  | 1.000 |  |  |  | C23(Dehydro): 100; C29(Dehydro): 100 |  |  |  |  |  |  |  | 0.67 |  | 1 |  | Sequest HT (3) |  | 5 |  | 594.12140 |  | -0.77 |  | 37.5489 |  | 9871 |  | 9871 |  | TRINITY\_DN0\_c1\_g1\_i14.p1,TRINITY\_DN0\_c1\_g1\_i4.p1 |  | Image |  | Peak List |  |
|  | | | | | | | | | | | | | | | | | | | | | | | | | | | | | | | | | | | | | | | | | | | | | | | | | | | | | |  |
|  | High |  | [D].TFKNLALNAAKSAGVSVLNSLScKLSKTc.[-] |  | HCD |  | C23(Dehydro); C29(Dehydro) |  | 4.73 |  | 1.3e-3 |  | 8.8e-3 |  | 0.719 |  | 253.61 |  | 1.000 |  |  |  | C23(Dehydro): 100; C29(Dehydro): 100 |  |  |  |  |  |  |  | 0.70 |  | 1 |  | Sequest HT (3) |  | 4 |  | 742.40118 |  | 0.92 |  | 37.6638 |  | 9919 |  | 9919 |  | TRINITY\_DN0\_c1\_g1\_i14.p1,TRINITY\_DN0\_c1\_g1\_i4.p1 |  | Image |  | Peak List |  |
|  | | | | | | | | | | | | | | | | | | | | | | | | | | | | | | | | | | | | | | | | | | | | | | | | | | | | | |  |
|  | High |  | [D].TFKNLALNAAKSAGVSVLNSLScKLSKTc.[-] |  | HCD |  | C23(Dehydro); C29(Dehydro) |  | 4.93 |  | 1.3e-3 |  | 5.8e-4 |  | 1.31 |  | 355.92 |  | 1.000 |  |  |  | C23(Dehydro): 100; C29(Dehydro): 100 |  |  |  |  |  |  |  | 0.71 |  | 1 |  | Sequest HT (3) |  | 3 |  | 989.52991 |  | -1.69 |  | 37.6715 |  | 9922 |  | 9922 |  | TRINITY\_DN0\_c1\_g1\_i14.p1,TRINITY\_DN0\_c1\_g1\_i4.p1 |  | Image |  | Peak List |  |
|  | | | | | | | | | | | | | | | | | | | | | | | | | | | | | | | | | | | | | | | | | | | | | | | | | | | | | |  |
|  | High |  | [D].TFKNLALNAAKSAGVSVLNSLScKLSKTc.[-] |  | HCD |  | C23(Dehydro); C29(Dehydro) |  | 5.30 |  | 1.3e-3 |  | 9.7e-3 |  | 0.699 |  | 254.86 |  | 1.000 |  |  |  | C23(Dehydro): 100; C29(Dehydro): 100 |  |  |  |  |  |  |  | 0.68 |  | 1 |  | Sequest HT (3) |  | 5 |  | 594.12170 |  | -0.26 |  | 37.6882 |  | 9928 |  | 9928 |  | TRINITY\_DN0\_c1\_g1\_i14.p1,TRINITY\_DN0\_c1\_g1\_i4.p1 |  | Image |  | Peak List |  |
|  | | | | | | | | | | | | | | | | | | | | | | | | | | | | | | | | | | | | | | | | | | | | | | | | | | | | | |  |
|  | High |  | [K].NLALNAAKSAGVSVLNSLScKLSKTc.[-] |  | HCD |  | C20(Dehydro); C26(Dehydro) |  | 7.00 |  | 1.3e-3 |  | 4.7e-5 |  | 1.86 |  | 468.15 |  | 1.000 |  |  |  | C20(Dehydro): 100; C26(Dehydro): 100 |  |  |  |  |  |  |  | 0.69 |  | 1 |  | Sequest HT (3) |  | 3 |  | 864.12817 |  | 0.33 |  | 37.7910 |  | 9976 |  | 9976 |  | TRINITY\_DN0\_c1\_g1\_i14.p1,TRINITY\_DN0\_c1\_g1\_i4.p1 |  | Image |  | Peak List |  |
|  | | | | | | | | | | | | | | | | | | | | | | | | | | | | | | | | | | | | | | | | | | | | | | | | | | | | | |  |
|  | High |  | [D].TFKNLALNAAKSAGVSVLNSLScKLSKTc.[-] |  | HCD |  | C23(Dehydro); C29(Dehydro) |  | 2.91 |  | 2.4e-3 |  | 8.4e-2 |  | 0.221 |  | 190.93 |  | 1.000 |  |  |  | C23(Dehydro): 100; C29(Dehydro): 100 |  |  |  |  |  |  |  | 0.60 |  | 1 |  | Sequest HT (3) |  | 4 |  | 742.40228 |  | 2.40 |  | 37.8048 |  | 9980 |  | 9980 |  | TRINITY\_DN0\_c1\_g1\_i14.p1,TRINITY\_DN0\_c1\_g1\_i4.p1 |  | Image |  | Peak List |  |
|  | | | | | | | | | | | | | | | | | | | | | | | | | | | | | | | | | | | | | | | | | | | | | | | | | | | | | |  |
|  | High |  | [K].NLALNAAKSAGVSVLNSLScKLSKTc.[-] |  | HCD |  | C20(Dehydro); C26(Dehydro) |  | 5.40 |  | 1.3e-3 |  | 5.1e-3 |  | 0.839 |  | 264.24 |  | 1.000 |  |  |  | C20(Dehydro): 100; C26(Dehydro): 100 |  |  |  |  |  |  |  | 0.55 |  | 1 |  | Sequest HT (3) |  | 4 |  | 648.34729 |  | -0.69 |  | 37.8111 |  | 9985 |  | 9985 |  | TRINITY\_DN0\_c1\_g1\_i14.p1,TRINITY\_DN0\_c1\_g1\_i4.p1 |  | Image |  | Peak List |  |
|  | | | | | | | | | | | | | | | | | | | | | | | | | | | | | | | | | | | | | | | | | | | | | | | | | | | | | |  |
|  | High |  | [D].TFKNLALNAAKSAGVSVLNSLScKLSKTc.[-] |  | HCD |  | C23(Dehydro); C29(Dehydro) |  | 3.35 |  | 1.3e-3 |  | 2.7e-3 |  | 0.977 |  | 293.65 |  | 1.000 |  |  |  | C23(Dehydro): 100; C29(Dehydro): 100 |  |  |  |  |  |  |  | 0.64 |  | 1 |  | Sequest HT (3) |  | 3 |  | 989.53040 |  | -1.19 |  | 37.8222 |  | 9990 |  | 9990 |  | TRINITY\_DN0\_c1\_g1\_i14.p1,TRINITY\_DN0\_c1\_g1\_i4.p1 |  | Image |  | Peak List |  |
|  | | | | | | | | | | | | | | | | | | | | | | | | | | | | | | | | | | | | | | | | | | | | | | | | | | | | | |  |
|  | High |  | [R].GLLDTFKNLALNAAK.[S] |  | HCD |  |  |  | 5.37 |  | 1.3e-3 |  | 4.9e-5 |  | 1.85 |  |  |  |  |  |  |  |  |  |  |  |  |  |  |  | 0.72 |  | 1 |  | Sequest HT (3) |  | 2 |  | 794.95612 |  | -0.48 |  | 37.8609 |  | 10011 |  | 10011 |  | TRINITY\_DN24401\_c0\_g1\_i2.p14; TRINITY\_DN0\_c1\_g1\_i14.p1,TRINITY\_DN0\_c1\_g1\_i4.p1 |  | Image |  | Peak List |  |
|  | | | | | | | | | | | | | | | | | | | | | | | | | | | | | | | | | | | | | | | | | | | | | | | | | | | | | |  |
|  | High |  | [R].GLLDTFKNLALNAAK.[S] |  | HCD |  |  |  | 3.95 |  | 1.3e-3 |  | 6.5e-3 |  | 0.786 |  |  |  |  |  |  |  |  |  |  |  |  |  |  |  | 0.59 |  | 1 |  | Sequest HT (3) |  | 3 |  | 530.30646 |  | -0.57 |  | 37.8901 |  | 10025 |  | 10025 |  | TRINITY\_DN24401\_c0\_g1\_i2.p14; TRINITY\_DN0\_c1\_g1\_i14.p1,TRINITY\_DN0\_c1\_g1\_i4.p1 |  | Image |  | Peak List |  |
|  | | | | | | | | | | | | | | | | | | | | | | | | | | | | | | | | | | | | | | | | | | | | | | | | | | | | | |  |
|  | High |  | [R].GLLDTFKNLALN.[A] |  | HCD |  |  |  | 2.85 |  | 1.3e-3 |  | 2.6e-2 |  | 0.481 |  |  |  |  |  |  |  |  |  |  |  |  |  |  |  | 0.44 |  | 1 |  | Sequest HT (3) |  | 2 |  | 659.87183 |  | -0.12 |  | 37.9326 |  | 10044 |  | 10044 |  | TRINITY\_DN24401\_c0\_g1\_i2.p14; TRINITY\_DN6490\_c1\_g1\_i1.p1\_ORF1; TRINITY\_DN0\_c1\_g1\_i14.p1,TRINITY\_DN0\_c1\_g1\_i4.p1; TRINITY\_DN6490\_c1\_g1\_i1.p1 |  | Image |  | Peak List |  |
|  | | | | | | | | | | | | | | | | | | | | | | | | | | | | | | | | | | | | | | | | | | | | | | | | | | | | | |  |
|  | High |  | [K].NLALNAAKSAGVSVLNSLScKLSKTc.[-] |  | HCD |  | C20(Dehydro); C26(Dehydro) |  | 4.29 |  | 1.3e-3 |  | 2.2e-3 |  | 1.03 |  | 382.60 |  | 1.000 |  |  |  | C20(Dehydro): 100; C26(Dehydro): 100 |  |  |  |  |  |  |  | 0.69 |  | 1 |  | Sequest HT (3) |  | 3 |  | 864.12689 |  | -1.16 |  | 37.9467 |  | 10051 |  | 10051 |  | TRINITY\_DN0\_c1\_g1\_i14.p1,TRINITY\_DN0\_c1\_g1\_i4.p1 |  | Image |  | Peak List |  |
|  | | | | | | | | | | | | | | | | | | | | | | | | | | | | | | | | | | | | | | | | | | | | | | | | | | | | | |  |
|  | High |  | [R].GLLDTFKNLALNAAK.[S] |  | HCD |  |  |  | 3.64 |  | 1.3e-3 |  | 1.4e-4 |  | 1.61 |  |  |  |  |  |  |  |  |  |  |  |  |  |  |  | 0.63 |  | 1 |  | Sequest HT (3) |  | 2 |  | 794.95593 |  | -0.71 |  | 38.0070 |  | 10079 |  | 10079 |  | TRINITY\_DN24401\_c0\_g1\_i2.p14; TRINITY\_DN0\_c1\_g1\_i14.p1,TRINITY\_DN0\_c1\_g1\_i4.p1 |  | Image |  | Peak List |  |
|  | | | | | | | | | | | | | | | | | | | | | | | | | | | | | | | | | | | | | | | | | | | | | | | | | | | | | |  |
|  | High |  | [R].GLLDTFKNLALNAAK.[S] |  | HCD |  |  |  | 3.12 |  | 1.3e-3 |  | 6.2e-3 |  | 0.795 |  |  |  |  |  |  |  |  |  |  |  |  |  |  |  | 0.60 |  | 1 |  | Sequest HT (3) |  | 3 |  | 530.30664 |  | -0.22 |  | 38.0303 |  | 10091 |  | 10091 |  | TRINITY\_DN24401\_c0\_g1\_i2.p14; TRINITY\_DN0\_c1\_g1\_i14.p1,TRINITY\_DN0\_c1\_g1\_i4.p1 |  | Image |  | Peak List |  |
|  | | | | | | | | | | | | | | | | | | | | | | | | | | | | | | | | | | | | | | | | | | | | | | | | | | | | | |  |
|  | High |  | [D].TFKNLALNAAKSAGVSVLnSLScKLSKTc.[-] |  | HCD |  | N19(Deamidated); C23(Dehydro); C29(Dehydro) |  | 5.16 |  | 1.3e-3 |  | 1.4e-3 |  | 1.12 |  | 236.79 |  | 1.000 |  | b1+(10): 1044.58; y1+(14): 1481.73; y1+(13): 1394.70; y1+(12): 1295.63; y1+(11): 1182.55; b2+(15): 743.92; y2+(19): 962.50; y2+(18): 898.45; y2+(12): 648.32; y2+(11): 591.78 |  | N19(Deamidated): 100; C23(Dehydro): 100; C29(Dehydro): 100 |  |  |  |  |  |  |  | 0.44 |  | 1 |  | Sequest HT (3) |  | 4 |  | 742.64594 |  | -0.77 |  | 38.0844 |  | 10121 |  | 10121 |  | TRINITY\_DN0\_c1\_g1\_i14.p1,TRINITY\_DN0\_c1\_g1\_i4.p1 |  | Image |  | Peak List |  |
|  | | | | | | | | | | | | | | | | | | | | | | | | | | | | | | | | | | | | | | | | | | | | | | | | | | | | | |  |
|  | High |  | [D].TFKNLALNAAKSAGVSVLnSLScKLSKTc.[-] |  | HCD |  | N19(Deamidated); C23(Dehydro); C29(Dehydro) |  | 5.06 |  | 1.3e-3 |  | 6.5e-3 |  | 0.786 |  | 232.85 |  | 1.000 |  | b1+(8): 902.51; b1+(10): 1044.58; y1+(12): 1295.63; y1+(11): 1182.55; y2+(19): 962.50; y2+(14): 741.37; y2+(12): 648.32; y2+(11): 591.78 |  | N19(Deamidated): 100; C23(Dehydro): 100; C29(Dehydro): 100 |  |  |  |  |  |  |  | 0.31 |  | 1 |  | Sequest HT (3) |  | 5 |  | 594.31799 |  | -1.12 |  | 38.1506 |  | 10160 |  | 10160 |  | TRINITY\_DN0\_c1\_g1\_i14.p1,TRINITY\_DN0\_c1\_g1\_i4.p1 |  | Image |  | Peak List |  |
|  | | | | | | | | | | | | | | | | | | | | | | | | | | | | | | | | | | | | | | | | | | | | | | | | | | | | | |  |
|  | High |  | [D].TFKNLALNAAKSAGVSVLnSLScKLSKTc.[-] |  | HCD |  | N19(Deamidated); C23(Dehydro); C29(Dehydro) |  | 4.56 |  | 1.3e-3 |  | 5.9e-4 |  | 1.31 |  | 337.31 |  | 1.000 |  | b1+(10): 1044.58; y1+(18): 1795.89; y1+(17): 1708.86; y1+(16): 1637.82; y1+(15): 1580.80; y1+(14): 1481.73; y1+(13): 1394.70; y1+(12): 1295.63; y1+(11): 1182.55; b2+(12): 630.36; b2+(14): 694.39; y2+(12): 648.32 |  | N19(Deamidated): 100; C23(Dehydro): 100; C29(Dehydro): 100 |  |  |  |  |  |  |  | 0.37 |  | 1 |  | Sequest HT (3) |  | 3 |  | 989.85797 |  | -1.63 |  | 38.1784 |  | 10172 |  | 10172 |  | TRINITY\_DN0\_c1\_g1\_i14.p1,TRINITY\_DN0\_c1\_g1\_i4.p1 |  | Image |  | Peak List |  |
|  | | | | | | | | | | | | | | | | | | | | | | | | | | | | | | | | | | | | | | | | | | | | | | | | | | | | | |  |
|  | High |  | [D].TFKNLALNAAKSAGVSVLnSLScKLSKTc.[-] |  | HCD |  | N19(Deamidated); C23(Dehydro); C29(Dehydro) |  | 5.19 |  | 1.3e-3 |  | 2.1e-3 |  | 1.03 |  | 361.87 |  | 1.000 |  | b1+(9): 973.55; b1+(10): 1044.58; y1+(14): 1481.73; y1+(12): 1295.63; y1+(11): 1182.55; b2+(12): 630.36; b2+(14): 694.39; b2+(15): 743.92; b2+(16): 787.44; b2+(17): 836.97; y2+(19): 962.50; y2+(18): 898.45; y2+(12): 648.32; y2+(11): 591.78 |  | N19(Deamidated): 100; C23(Dehydro): 100; C29(Dehydro): 100 |  |  |  |  |  |  |  | 0.37 |  | 1 |  | Sequest HT (3) |  | 4 |  | 742.64581 |  | -0.93 |  | 38.2277 |  | 10195 |  | 10195 |  | TRINITY\_DN0\_c1\_g1\_i14.p1,TRINITY\_DN0\_c1\_g1\_i4.p1 |  | Image |  | Peak List |  |
|  | | | | | | | | | | | | | | | | | | | | | | | | | | | | | | | | | | | | | | | | | | | | | | | | | | | | | |  |
|  | High |  | [D].TFKNLALNAAKSAGVSVLnSLScKLSKTc.[-] |  | HCD |  | N19(Deamidated); C23(Dehydro); C29(Dehydro) |  | 4.04 |  | 1.3e-3 |  | 4.4e-3 |  | 0.872 |  | 268.48 |  | 1.000 |  | b1+(10): 1044.58; y1+(14): 1481.73; y1+(12): 1295.63; y1+(11): 1182.55; y2+(19): 962.50; y2+(18): 898.45; y2+(14): 741.37; y2+(12): 648.32; y2+(11): 591.78 |  | N19(Deamidated): 100; C23(Dehydro): 100; C29(Dehydro): 100 |  |  |  |  |  |  |  | 0.32 |  | 1 |  | Sequest HT (3) |  | 5 |  | 594.31787 |  | -1.33 |  | 38.2943 |  | 10228 |  | 10228 |  | TRINITY\_DN0\_c1\_g1\_i14.p1,TRINITY\_DN0\_c1\_g1\_i4.p1 |  | Image |  | Peak List |  |
|  | | | | | | | | | | | | | | | | | | | | | | | | | | | | | | | | | | | | | | | | | | | | | | | | | | | | | |  |
|  | High |  | [R].GLLDTFKNLALNAAKS.[A] |  | HCD |  |  |  | 4.62 |  | 1.3e-3 |  | 2.1e-3 |  | 1.03 |  |  |  |  |  |  |  |  |  |  |  |  |  |  |  | 0.49 |  | 1 |  | Sequest HT (3) |  | 3 |  | 559.31677 |  | -1.18 |  | 38.3006 |  | 10233 |  | 10233 |  | TRINITY\_DN24401\_c0\_g1\_i2.p14; TRINITY\_DN0\_c1\_g1\_i14.p1,TRINITY\_DN0\_c1\_g1\_i4.p1 |  | Image |  | Peak List |  |
|  | | | | | | | | | | | | | | | | | | | | | | | | | | | | | | | | | | | | | | | | | | | | | | | | | | | | | |  |
|  | High |  | [D].TFKNLALNAAKSAGVSVLnSLScKLSKTc.[-] |  | HCD |  | N19(Deamidated); C23(Dehydro); C29(Dehydro) |  | 4.52 |  | 1.3e-3 |  | 8.4e-4 |  | 1.23 |  | 357.97 |  | 1.000 |  | b1+(9): 973.55; b1+(10): 1044.58; y1+(18): 1795.89; y1+(17): 1708.86; y1+(14): 1481.73; y1+(13): 1394.70; y1+(12): 1295.63; y1+(11): 1182.55; b2+(12): 630.36; b2+(15): 743.92; b2+(16): 787.44; b2+(17): 836.97 |  | N19(Deamidated): 100; C23(Dehydro): 100; C29(Dehydro): 100 |  |  |  |  |  |  |  | 0.37 |  | 1 |  | Sequest HT (3) |  | 3 |  | 989.85944 |  | -0.15 |  | 38.3292 |  | 10246 |  | 10246 |  | TRINITY\_DN0\_c1\_g1\_i14.p1,TRINITY\_DN0\_c1\_g1\_i4.p1 |  | Image |  | Peak List |  |
|  | | | | | | | | | | | | | | | | | | | | | | | | | | | | | | | | | | | | | | | | | | | | | | | | | | | | | |  |
|  | High |  | [R].GLLDTFKNLALNAAKS.[A] |  | HCD |  |  |  | 4.27 |  | 1.3e-3 |  | 5.5e-4 |  | 1.32 |  |  |  |  |  |  |  |  |  |  |  |  |  |  |  | 0.57 |  | 1 |  | Sequest HT (3) |  | 2 |  | 838.47180 |  | -0.85 |  | 38.3891 |  | 10276 |  | 10276 |  | TRINITY\_DN24401\_c0\_g1\_i2.p14; TRINITY\_DN0\_c1\_g1\_i14.p1,TRINITY\_DN0\_c1\_g1\_i4.p1 |  | Image |  | Peak List |  |
|  | | | | | | | | | | | | | | | | | | | | | | | | | | | | | | | | | | | | | | | | | | | | | | | | | | | | | |  |
|  | High |  | [R].GLLDTFKNLALNAAKS.[A] |  | HCD |  |  |  | 2.46 |  | 3.9e-3 |  | 1.5e-1 |  | 0.084 |  |  |  |  |  |  |  |  |  |  |  |  |  |  |  | 0.45 |  | 1 |  | Sequest HT (3) |  | 3 |  | 559.31732 |  | -0.20 |  | 38.5099 |  | 10338 |  | 10338 |  | TRINITY\_DN24401\_c0\_g1\_i2.p14; TRINITY\_DN0\_c1\_g1\_i14.p1,TRINITY\_DN0\_c1\_g1\_i4.p1 |  | Image |  | Peak List |  |
|  | | | | | | | | | | | | | | | | | | | | | | | | | | | | | | | | | | | | | | | | | | | | | | | | | | | | | |  |
|  | High |  | [R].GLLDTFKNLALnAAK.[S] |  | HCD |  | N12(Deamidated) |  | 3.79 |  | 1.3e-3 |  | 6.0e-3 |  | 0.801 |  | 277.68 |  | 1.000 |  | b1+(8): 889.48; y1+(7): 701.42; y1+(6): 588.34; y1+(5): 517.30; y1+(4): 404.21 |  | N12(Deamidated): 100 |  |  |  |  |  |  |  | 0.32 |  | 1 |  | Sequest HT (3) |  | 3 |  | 530.63586 |  | 2.08 |  | 38.6010 |  | 10378 |  | 10378 |  | TRINITY\_DN24401\_c0\_g1\_i2.p14; TRINITY\_DN0\_c1\_g1\_i14.p1,TRINITY\_DN0\_c1\_g1\_i4.p1 |  | Image |  | Peak List |  |
|  | | | | | | | | | | | | | | | | | | | | | | | | | | | | | | | | | | | | | | | | | | | | | | | | | | | | | |  |
|  | High |  | [R].GLLDTFKNLALnAAK.[S] |  | HCD |  | N12(Deamidated) |  | 4.11 |  | 1.3e-3 |  | 6.4e-5 |  | 1.79 |  | 451.37 |  | 1.000 |  | b1+(8): 889.48; b1+(9): 1002.56; b1+(10): 1073.60; y1+(7): 701.42; y1+(6): 588.34; y1+(5): 517.30; y1+(4): 404.21 |  | N12(Deamidated): 100 |  |  |  |  |  |  |  | 0.33 |  | 1 |  | Sequest HT (3) |  | 2 |  | 795.45013 |  | 2.05 |  | 38.6190 |  | 10386 |  | 10386 |  | TRINITY\_DN24401\_c0\_g1\_i2.p14; TRINITY\_DN0\_c1\_g1\_i14.p1,TRINITY\_DN0\_c1\_g1\_i4.p1 |  | Image |  | Peak List |  |
|  | | | | | | | | | | | | | | | | | | | | | | | | | | | | | | | | | | | | | | | | | | | | | | | | | | | | | |  |
|  | High |  | [R].GLLDTFKNLALnAAK.[S] |  | HCD |  | N12(Deamidated) |  | 3.73 |  | 1.3e-3 |  | 1.1e-2 |  | 0.672 |  | 237.20 |  | 0.000 |  | b1+(8): 890.46; y1+(7): 700.44; y1+(6): 587.35; y1+(5): 516.31; y1+(4): 403.23 |  | N8(Deamidated): 99.99 |  |  |  |  |  |  |  | 0.01 |  | 1 |  | Sequest HT (3) |  | 3 |  | 530.63574 |  | 1.85 |  | 38.7452 |  | 10449 |  | 10449 |  | TRINITY\_DN24401\_c0\_g1\_i2.p14; TRINITY\_DN0\_c1\_g1\_i14.p1,TRINITY\_DN0\_c1\_g1\_i4.p1 |  | Image |  | Peak List |  |
|  | | | | | | | | | | | | | | | | | | | | | | | | | | | | | | | | | | | | | | | | | | | | | | | | | | | | | |  |
|  | High |  | [R].GLLDTFKnLALNAAK.[S] |  | HCD |  | N8(Deamidated) |  | 3.68 |  | 1.3e-3 |  | 6.8e-3 |  | 0.776 |  | 278.53 |  | 1.000 |  | b1+(8): 890.46; y1+(7): 700.44; y1+(6): 587.35; y1+(5): 516.31; y1+(4): 403.23 |  | N8(Deamidated): 99.99 |  |  |  |  |  |  |  |  |  | 2 |  | Sequest HT (3) |  | 3 |  | 530.63574 |  | 1.85 |  | 38.7452 |  | 10449 |  | 10449 |  | TRINITY\_DN24401\_c0\_g1\_i2.p14; TRINITY\_DN0\_c1\_g1\_i14.p1,TRINITY\_DN0\_c1\_g1\_i4.p1 |  | Image |  | Peak List |  |
|  | | | | | | | | | | | | | | | | | | | | | | | | | | | | | | | | | | | | | | | | | | | | | | | | | | | | | |  |
|  | High |  | [R].GLLDTFKnLALNAAK.[S] |  | HCD |  | N8(Deamidated) |  | 4.30 |  | 1.3e-3 |  | 7.8e-5 |  | 1.75 |  | 421.61 |  | 1.000 |  | b1+(8): 890.46; b1+(10): 1074.58; b1+(11): 1187.67; y1+(7): 700.44; y1+(6): 587.35; y1+(5): 516.31; y1+(4): 403.23 |  | N8(Deamidated): 100 |  |  |  |  |  |  |  | 0.17 |  | 1 |  | Sequest HT (3) |  | 2 |  | 795.44891 |  | 0.51 |  | 38.7709 |  | 10462 |  | 10462 |  | TRINITY\_DN24401\_c0\_g1\_i2.p14; TRINITY\_DN0\_c1\_g1\_i14.p1,TRINITY\_DN0\_c1\_g1\_i4.p1 |  | Image |  | Peak List |  |
|  | | | | | | | | | | | | | | | | | | | | | | | | | | | | | | | | | | | | | | | | | | | | | | | | | | | | | |  |
|  | High |  | [D].TFKNLALnAAKSAGVSVLNSLScKLSKTc.[-] |  | HCD |  | N8(Deamidated); C23(Dehydro); C29(Dehydro) |  | 5.37 |  | 1.3e-3 |  | 1.0e-2 |  | 0.685 |  | 270.68 |  | 1.000 |  | b1+(4): 491.26; b1+(5): 604.35; b1+(6): 675.38; y2+(22): 1090.56 |  | N8(Deamidated): 100; C23(Dehydro): 100; C29(Dehydro): 100 |  |  |  |  |  |  |  | 0.18 |  | 1 |  | Sequest HT (3) |  | 4 |  | 742.64716 |  | 0.88 |  | 38.8400 |  | 10501 |  | 10501 |  | TRINITY\_DN0\_c1\_g1\_i14.p1,TRINITY\_DN0\_c1\_g1\_i4.p1 |  | Image |  | Peak List |  |
|  | | | | | | | | | | | | | | | | | | | | | | | | | | | | | | | | | | | | | | | | | | | | | | | | | | | | | |  |
|  | High |  | [D].TFKNLALnAAKSAGVSVLNSLScKLSKTc.[-] |  | HCD |  | N8(Deamidated); C23(Dehydro); C29(Dehydro) |  | 4.40 |  | 1.3e-3 |  | 8.8e-4 |  | 1.22 |  | 318.92 |  | 1.000 |  | b1+(4): 491.26; b1+(5): 604.35; b1+(6): 675.38; b1+(7): 788.47; y2+(23): 1147.10; y2+(22): 1090.56 |  | N8(Deamidated): 100; C23(Dehydro): 100; C29(Dehydro): 100 |  |  |  |  |  |  |  | 0.08 |  | 1 |  | Sequest HT (3) |  | 3 |  | 989.85938 |  | -0.21 |  | 38.8587 |  | 10509 |  | 10509 |  | TRINITY\_DN0\_c1\_g1\_i14.p1,TRINITY\_DN0\_c1\_g1\_i4.p1 |  | Image |  | Peak List |  |
|  | | | | | | | | | | | | | | | | | | | | | | | | | | | | | | | | | | | | | | | | | | | | | | | | | | | | | |  |
|  | High |  | [K].nLALNAAKSAGVSVLNSLScKLSKTc.[-] |  | HCD |  | N1(Deamidated); C20(Dehydro); C26(Dehydro) |  | 5.62 |  | 1.3e-3 |  | 4.9e-4 |  | 1.35 |  | 276.77 |  | 0.987 |  | b1+(2): 229.12; b1+(3): 300.16; b1+(4): 413.24 |  | N1(Deamidated): 98.68; C20(Dehydro): 100; C26(Dehydro): 100 |  |  |  |  |  |  |  | 0.15 |  | 1 |  | Sequest HT (3) |  | 3 |  | 864.45776 |  | 2.16 |  | 38.9162 |  | 10536 |  | 10536 |  | TRINITY\_DN0\_c1\_g1\_i14.p1,TRINITY\_DN0\_c1\_g1\_i4.p1 |  | Image |  | Peak List |  |
|  | | | | | | | | | | | | | | | | | | | | | | | | | | | | | | | | | | | | | | | | | | | | | | | | | | | | | |  |
|  | High |  | [D].TFKNLALnAAKSAGVSVLNSLScKLSKTc.[-] |  | HCD |  | N8(Deamidated); C23(Dehydro); C29(Dehydro) |  | 4.30 |  | 2.4e-3 |  | 7.8e-2 |  | 0.239 |  | 186.23 |  | 1.000 |  | b1+(4): 491.26; b1+(5): 604.35; b1+(6): 675.38; b1+(7): 788.47 |  | N8(Deamidated): 100; C23(Dehydro): 100; C29(Dehydro): 100 |  |  |  |  |  |  |  | 0.08 |  | 1 |  | Sequest HT (3) |  | 5 |  | 594.31903 |  | 0.63 |  | 38.9673 |  | 10558 |  | 10558 |  | TRINITY\_DN0\_c1\_g1\_i14.p1,TRINITY\_DN0\_c1\_g1\_i4.p1 |  | Image |  | Peak List |  |
|  | | | | | | | | | | | | | | | | | | | | | | | | | | | | | | | | | | | | | | | | | | | | | | | | | | | | | |  |
|  | High |  | [D].TFKNLALnAAKSAGVSVLNSLScKLSKTc.[-] |  | HCD |  | N8(Deamidated); C23(Dehydro); C29(Dehydro) |  | 3.94 |  | 1.3e-3 |  | 4.7e-3 |  | 0.857 |  | 264.50 |  | 1.000 |  | b1+(4): 491.26; b1+(5): 604.35; b1+(6): 675.38 |  | N8(Deamidated): 100; C23(Dehydro): 100; C29(Dehydro): 100 |  |  |  |  |  |  |  | 0.16 |  | 1 |  | Sequest HT (3) |  | 4 |  | 742.64758 |  | 1.45 |  | 38.9828 |  | 10566 |  | 10566 |  | TRINITY\_DN0\_c1\_g1\_i14.p1,TRINITY\_DN0\_c1\_g1\_i4.p1 |  | Image |  | Peak List |  |
|  | | | | | | | | | | | | | | | | | | | | | | | | | | | | | | | | | | | | | | | | | | | | | | | | | | | | | |  |
|  | High |  | [R].GLLDTFKnLALNAAKS.[A] |  | HCD |  | N8(Deamidated) |  | 4.22 |  | 1.3e-3 |  | 4.6e-3 |  | 0.86 |  | 331.67 |  | 1.000 |  | b1+(8): 890.46; b1+(9): 1003.55; b1+(10): 1074.58; b1+(11): 1187.67; y1+(5): 490.26 |  | N8(Deamidated): 100 |  |  |  |  |  |  |  | 0.18 |  | 1 |  | Sequest HT (3) |  | 2 |  | 838.96613 |  | 1.92 |  | 39.1283 |  | 10640 |  | 10640 |  | TRINITY\_DN24401\_c0\_g1\_i2.p14; TRINITY\_DN0\_c1\_g1\_i14.p1,TRINITY\_DN0\_c1\_g1\_i4.p1 |  | Image |  | Peak List |  |
|  | | | | | | | | | | | | | | | | | | | | | | | | | | | | | | | | | | | | | | | | | | | | | | | | | | | | | |  |
|  | High |  | [R].GLLDTFKnLALnAAK.[S] |  | HCD |  | N8(Deamidated); N12(Deamidated) |  | 3.87 |  | 1.3e-3 |  | 5.9e-3 |  | 0.807 |  | 285.12 |  | 1.000 |  |  |  | N8(Deamidated): 100; N12(Deamidated): 100 |  |  |  |  |  |  |  | 0.62 |  | 1 |  | Sequest HT (3) |  | 3 |  | 530.96295 |  | 0.35 |  | 39.2914 |  | 10722 |  | 10722 |  | TRINITY\_DN24401\_c0\_g1\_i2.p14; TRINITY\_DN0\_c1\_g1\_i14.p1,TRINITY\_DN0\_c1\_g1\_i4.p1 |  | Image |  | Peak List |  |
|  | | | | | | | | | | | | | | | | | | | | | | | | | | | | | | | | | | | | | | | | | | | | | | | | | | | | | |  |
|  | High |  | [D].TFKNLALnAAKSAGVSVLnSLScKLSKTc.[-] |  | HCD |  | N8(Deamidated); N19(Deamidated); C23(Dehydro); C29(Dehydro) |  | 2.66 |  | 3.9e-3 |  | 1.7e-1 |  | 0.063 |  | 120.08 |  | 0.999 |  | b1+(4): 491.26; b1+(5): 604.35 |  | N8(Deamidated): 99.86; N19(Deamidated): 100; C23(Dehydro): 100; C29(Dehydro): 100 |  |  |  |  |  |  |  | 0.36 |  | 1 |  | Sequest HT (3) |  | 4 |  | 742.89136 |  | -1.55 |  | 39.2952 |  | 10725 |  | 10725 |  | TRINITY\_DN0\_c1\_g1\_i14.p1,TRINITY\_DN0\_c1\_g1\_i4.p1 |  | Image |  | Peak List |  |
|  | | | | | | | | | | | | | | | | | | | | | | | | | | | | | | | | | | | | | | | | | | | | | | | | | | | | | |  |
|  | High |  | [R].GLLDTFKNLALNAAKSAG.[V] |  | HCD |  |  |  | 5.91 |  | 1.3e-3 |  | 8.4e-5 |  | 1.73 |  |  |  |  |  |  |  |  |  |  |  |  |  |  |  | 0.55 |  | 1 |  | Sequest HT (3) |  | 2 |  | 902.50287 |  | 1.18 |  | 39.7067 |  | 10926 |  | 10926 |  | TRINITY\_DN0\_c1\_g1\_i14.p1,TRINITY\_DN0\_c1\_g1\_i4.p1 |  | Image |  | Peak List |  |
|  | | | | | | | | | | | | | | | | | | | | | | | | | | | | | | | | | | | | | | | | | | | | | | | | | | | | | |  |
|  | High |  | [R].GLLDTFKNLALNAAKSAG.[V] |  | HCD |  |  |  | 4.29 |  | 1.3e-3 |  | 4.9e-3 |  | 0.848 |  |  |  |  |  |  |  |  |  |  |  |  |  |  |  | 0.50 |  | 1 |  | Sequest HT (3) |  | 3 |  | 602.00476 |  | 1.89 |  | 39.7094 |  | 10928 |  | 10928 |  | TRINITY\_DN0\_c1\_g1\_i14.p1,TRINITY\_DN0\_c1\_g1\_i4.p1 |  | Image |  | Peak List |  |
|  | | | | | | | | | | | | | | | | | | | | | | | | | | | | | | | | | | | | | | | | | | | | | | | | | | | | | |  |
|  | High |  | [D].TFKnLALNAAKSAGVSVLNSLScKLSKTc.[-] |  | HCD |  | N4(Deamidated); C23(Dehydro); C29(Dehydro) |  | 6.89 |  | 1.3e-3 |  | 7.6e-4 |  | 1.25 |  | 270.68 |  | 1.000 |  | b1+(4): 492.25; b1+(5): 605.33; b1+(6): 676.37; b1+(7): 789.45 |  | N4(Deamidated): 100; C23(Dehydro): 100; C29(Dehydro): 100 |  |  |  |  |  |  |  | 0.14 |  | 1 |  | Sequest HT (3) |  | 4 |  | 742.64722 |  | 0.96 |  | 39.7815 |  | 10965 |  | 10965 |  | TRINITY\_DN0\_c1\_g1\_i14.p1,TRINITY\_DN0\_c1\_g1\_i4.p1 |  | Image |  | Peak List |  |
|  | | | | | | | | | | | | | | | | | | | | | | | | | | | | | | | | | | | | | | | | | | | | | | | | | | | | | |  |
|  | High |  | [D].TFKnLALNAAKSAGVSVLNSLScKLSKTc.[-] |  | HCD |  | N4(Deamidated); C23(Dehydro); C29(Dehydro) |  | 6.21 |  | 1.3e-3 |  | 1.8e-6 |  | 2.57 |  | 339.48 |  | 1.000 |  | b1+(4): 492.25; b1+(5): 605.33; b1+(6): 676.37; b1+(7): 789.45; y2+(24): 1182.12 |  | N4(Deamidated): 100; C23(Dehydro): 100; C29(Dehydro): 100 |  |  |  |  |  |  |  | 0.17 |  | 1 |  | Sequest HT (3) |  | 3 |  | 989.85962 |  | 0.04 |  | 39.7840 |  | 10967 |  | 10967 |  | TRINITY\_DN0\_c1\_g1\_i14.p1,TRINITY\_DN0\_c1\_g1\_i4.p1 |  | Image |  | Peak List |  |
|  | | | | | | | | | | | | | | | | | | | | | | | | | | | | | | | | | | | | | | | | | | | | | | | | | | | | | |  |
|  | High |  | [D].TFKnLALNAAKSAGVSVLNSLScKLSKTc.[-] |  | HCD |  | N4(Deamidated); C23(Dehydro); C29(Dehydro) |  | 5.37 |  | 1.3e-3 |  | 1.1e-2 |  | 0.667 |  | 204.89 |  | 1.000 |  | b1+(4): 492.25; b1+(5): 605.33; b1+(6): 676.37; b1+(7): 789.45 |  | N4(Deamidated): 100; C23(Dehydro): 100; C29(Dehydro): 100 |  |  |  |  |  |  |  | 0.17 |  | 1 |  | Sequest HT (3) |  | 5 |  | 594.31824 |  | -0.71 |  | 39.7903 |  | 10972 |  | 10972 |  | TRINITY\_DN0\_c1\_g1\_i14.p1,TRINITY\_DN0\_c1\_g1\_i4.p1 |  | Image |  | Peak List |  |
|  | | | | | | | | | | | | | | | | | | | | | | | | | | | | | | | | | | | | | | | | | | | | | | | | | | | | | |  |
|  | High |  | [R].GLLDTFKNLALNAAKSAG.[V] |  | HCD |  |  |  | 1.77 |  | 3.1e-3 |  | 1.2e-1 |  | 0.145 |  |  |  |  |  |  |  |  |  |  |  |  |  |  |  | 0.39 |  | 1 |  | Sequest HT (3) |  | 2 |  | 902.50220 |  | 0.44 |  | 39.8456 |  | 10999 |  | 10999 |  | TRINITY\_DN0\_c1\_g1\_i14.p1,TRINITY\_DN0\_c1\_g1\_i4.p1 |  | Image |  | Peak List |  |
|  | | | | | | | | | | | | | | | | | | | | | | | | | | | | | | | | | | | | | | | | | | | | | | | | | | | | | |  |
|  | High |  | [R].GLLDTFKNLALNAAKSAG.[V] |  | HCD |  |  |  | 3.28 |  | 1.3e-3 |  | 2.3e-2 |  | 0.513 |  |  |  |  |  |  |  |  |  |  |  |  |  |  |  | 0.59 |  | 1 |  | Sequest HT (3) |  | 3 |  | 602.00403 |  | 0.67 |  | 39.8646 |  | 11011 |  | 11011 |  | TRINITY\_DN0\_c1\_g1\_i14.p1,TRINITY\_DN0\_c1\_g1\_i4.p1 |  | Image |  | Peak List |  |
|  | | | | | | | | | | | | | | | | | | | | | | | | | | | | | | | | | | | | | | | | | | | | | | | | | | | | | |  |
|  | High |  | [R].GLLDTFKNLAL.[N] |  | HCD |  |  |  | 2.51 |  | 1.3e-3 |  | 2.8e-3 |  | 0.967 |  |  |  |  |  |  |  |  |  |  |  |  |  |  |  | 0.41 |  | 1 |  | Sequest HT (3) |  | 2 |  | 602.85077 |  | 0.55 |  | 39.8646 |  | 11011 |  | 11011 |  | TRINITY\_DN24401\_c0\_g1\_i2.p14; TRINITY\_DN6490\_c1\_g1\_i1.p1\_ORF1; TRINITY\_DN0\_c1\_g1\_i14.p1,TRINITY\_DN0\_c1\_g1\_i4.p1; TRINITY\_DN6490\_c1\_g1\_i1.p1 |  | Image |  | Peak List |  |
|  | | | | | | | | | | | | | | | | | | | | | | | | | | | | | | | | | | | | | | | | | | | | | | | | | | | | | |  |
|  | High |  | [R].GLLDTFKNLAL.[N] |  | HCD |  |  |  | 2.43 |  | 1.3e-3 |  | 5.2e-2 |  | 0.331 |  |  |  |  |  |  |  |  |  |  |  |  |  |  |  | 0.38 |  | 1 |  | Sequest HT (3) |  | 2 |  | 602.85089 |  | 0.75 |  | 39.8761 |  | 11017 |  | 11017 |  | TRINITY\_DN24401\_c0\_g1\_i2.p14; TRINITY\_DN6490\_c1\_g1\_i1.p1\_ORF1; TRINITY\_DN0\_c1\_g1\_i14.p1,TRINITY\_DN0\_c1\_g1\_i4.p1; TRINITY\_DN6490\_c1\_g1\_i1.p1 |  | Image |  | Peak List |  |
|  | | | | | | | | | | | | | | | | | | | | | | | | | | | | | | | | | | | | | | | | | | | | | | | | | | | | | |  |
|  | High |  | [L].DTFKNLALNAAKSAGVSVLNSLScKLSKTc.[-] |  | HCD |  | C24(Dehydro); C30(Dehydro) |  | 5.19 |  | 1.3e-3 |  | 1.2e-3 |  | 1.15 |  | 336.83 |  | 1.000 |  |  |  | C24(Dehydro): 100; C30(Dehydro): 100 |  |  |  |  |  |  |  | 0.57 |  | 1 |  | Sequest HT (3) |  | 4 |  | 771.15845 |  | 1.57 |  | 39.9208 |  | 11038 |  | 11038 |  | TRINITY\_DN0\_c1\_g1\_i14.p1,TRINITY\_DN0\_c1\_g1\_i4.p1 |  | Image |  | Peak List |  |
|  | | | | | | | | | | | | | | | | | | | | | | | | | | | | | | | | | | | | | | | | | | | | | | | | | | | | | |  |
|  | High |  | [R].GLLDTFKNLALNAA.[KT] |  | HCD |  |  |  | 2.72 |  | 2.4e-3 |  | 7.1e-2 |  | 0.261 |  |  |  |  |  |  |  |  |  |  |  |  |  |  |  | 0.50 |  | 1 |  | Sequest HT (3) |  | 2 |  | 730.90887 |  | -0.19 |  | 39.9246 |  | 11041 |  | 11041 |  | TRINITY\_DN24401\_c0\_g1\_i2.p14; TRINITY\_DN6490\_c1\_g1\_i1.p1\_ORF1; TRINITY\_DN0\_c1\_g1\_i14.p1,TRINITY\_DN0\_c1\_g1\_i4.p1; TRINITY\_DN6490\_c1\_g1\_i1.p1 |  | Image |  | Peak List |  |
|  | | | | | | | | | | | | | | | | | | | | | | | | | | | | | | | | | | | | | | | | | | | | | | | | | | | | | |  |
|  | High |  | [L].DTFKNLALNAAKSAGVSVLNSLScKLSKTc.[-] |  | HCD |  | C24(Dehydro); C30(Dehydro) |  | 5.15 |  | 1.3e-3 |  | 4.0e-5 |  | 1.9 |  | 424.89 |  | 1.000 |  |  |  | C24(Dehydro): 100; C30(Dehydro): 100 |  |  |  |  |  |  |  | 0.64 |  | 1 |  | Sequest HT (3) |  | 3 |  | 1027.87354 |  | -0.35 |  | 39.9284 |  | 11044 |  | 11044 |  | TRINITY\_DN0\_c1\_g1\_i14.p1,TRINITY\_DN0\_c1\_g1\_i4.p1 |  | Image |  | Peak List |  |
|  | | | | | | | | | | | | | | | | | | | | | | | | | | | | | | | | | | | | | | | | | | | | | | | | | | | | | |  |
|  | High |  | [R].GLLDTFKNLALNA.[A] |  | HCD |  |  |  | 2.24 |  | 1.3e-3 |  | 6.6e-2 |  | 0.277 |  |  |  |  |  |  |  |  |  |  |  |  |  |  |  | 0.30 |  | 1 |  | Sequest HT (3) |  | 2 |  | 695.39081 |  | 0.50 |  | 39.9548 |  | 11058 |  | 11058 |  | TRINITY\_DN24401\_c0\_g1\_i2.p14; TRINITY\_DN6490\_c1\_g1\_i1.p1\_ORF1; TRINITY\_DN0\_c1\_g1\_i14.p1,TRINITY\_DN0\_c1\_g1\_i4.p1; TRINITY\_DN6490\_c1\_g1\_i1.p1 |  | Image |  | Peak List |  |
|  | | | | | | | | | | | | | | | | | | | | | | | | | | | | | | | | | | | | | | | | | | | | | | | | | | | | | |  |
|  | High |  | [L].LDTFKNLALNAAKSAGVSVLNSLScKLSKTc.[-] |  | HCD |  | C25(Dehydro); C31(Dehydro) |  | 4.29 |  | 1.3e-3 |  | 3.6e-4 |  | 1.41 |  | 327.50 |  | 1.000 |  |  |  | C25(Dehydro): 100; C31(Dehydro): 100 |  |  |  |  |  |  |  | 0.64 |  | 1 |  | Sequest HT (3) |  | 3 |  | 1065.57019 |  | 1.51 |  | 39.9680 |  | 11065 |  | 11065 |  | TRINITY\_DN0\_c1\_g1\_i14.p1,TRINITY\_DN0\_c1\_g1\_i4.p1 |  | Image |  | Peak List |  |
|  | | | | | | | | | | | | | | | | | | | | | | | | | | | | | | | | | | | | | | | | | | | | | | | | | | | | | |  |
|  | High |  | [R].GLLDTFKNLALNAAKSA.[VG] |  | HCD |  |  |  | 5.21 |  | 1.3e-3 |  | 1.1e-3 |  | 1.17 |  |  |  |  |  |  |  |  |  |  |  |  |  |  |  | 0.66 |  | 1 |  | Sequest HT (3) |  | 2 |  | 873.99109 |  | 0.02 |  | 40.0712 |  | 11118 |  | 11118 |  | TRINITY\_DN24401\_c0\_g1\_i2.p14; TRINITY\_DN0\_c1\_g1\_i14.p1,TRINITY\_DN0\_c1\_g1\_i4.p1 |  | Image |  | Peak List |  |
|  | | | | | | | | | | | | | | | | | | | | | | | | | | | | | | | | | | | | | | | | | | | | | | | | | | | | | |  |
|  | High |  | [R].GLLDTFKNLALNAAKSA.[VG] |  | HCD |  |  |  | 4.34 |  | 1.3e-3 |  | 4.0e-3 |  | 0.893 |  |  |  |  |  |  |  |  |  |  |  |  |  |  |  | 0.57 |  | 1 |  | Sequest HT (3) |  | 3 |  | 582.99664 |  | 0.30 |  | 40.0750 |  | 11121 |  | 11121 |  | TRINITY\_DN24401\_c0\_g1\_i2.p14; TRINITY\_DN0\_c1\_g1\_i14.p1,TRINITY\_DN0\_c1\_g1\_i4.p1 |  | Image |  | Peak List |  |
|  | | | | | | | | | | | | | | | | | | | | | | | | | | | | | | | | | | | | | | | | | | | | | | | | | | | | | |  |
|  | High |  | [R].GLLDTFKNLALNAAKSA.[VG] |  | HCD |  |  |  | 3.40 |  | 1.3e-3 |  | 5.8e-3 |  | 0.81 |  |  |  |  |  |  |  |  |  |  |  |  |  |  |  | 0.63 |  | 1 |  | Sequest HT (3) |  | 2 |  | 873.99023 |  | -0.95 |  | 40.2093 |  | 11186 |  | 11186 |  | TRINITY\_DN24401\_c0\_g1\_i2.p14; TRINITY\_DN0\_c1\_g1\_i14.p1,TRINITY\_DN0\_c1\_g1\_i4.p1 |  | Image |  | Peak List |  |
|  | | | | | | | | | | | | | | | | | | | | | | | | | | | | | | | | | | | | | | | | | | | | | | | | | | | | | |  |
|  | High |  | [R].GLLDTFKNLALNAAKSA.[VG] |  | HCD |  |  |  | 2.72 |  | 1.3e-3 |  | 2.3e-2 |  | 0.507 |  |  |  |  |  |  |  |  |  |  |  |  |  |  |  | 0.55 |  | 1 |  | Sequest HT (3) |  | 3 |  | 582.99762 |  | 1.97 |  | 40.2334 |  | 11197 |  | 11197 |  | TRINITY\_DN24401\_c0\_g1\_i2.p14; TRINITY\_DN0\_c1\_g1\_i14.p1,TRINITY\_DN0\_c1\_g1\_i4.p1 |  | Image |  | Peak List |  |
|  | | | | | | | | | | | | | | | | | | | | | | | | | | | | | | | | | | | | | | | | | | | | | | | | | | | | | |  |
|  | High |  | [R].GLLDTFKnLALNAAKSAG.[V] |  | HCD |  | N8(Deamidated) |  | 5.20 |  | 1.3e-3 |  | 8.4e-5 |  | 1.73 |  | 433.18 |  | 1.000 |  | b1+(8): 890.46; b1+(9): 1003.55; b1+(10): 1074.58; b1+(11): 1187.67; y1+(10): 915.53; y1+(9): 802.44; y1+(8): 731.40; y1+(7): 618.32 |  | N8(Deamidated): 100 |  |  |  |  |  |  |  | 0.37 |  | 1 |  | Sequest HT (3) |  | 2 |  | 902.99445 |  | 0.71 |  | 40.2787 |  | 11219 |  | 11219 |  | TRINITY\_DN0\_c1\_g1\_i14.p1,TRINITY\_DN0\_c1\_g1\_i4.p1 |  | Image |  | Peak List |  |
|  | | | | | | | | | | | | | | | | | | | | | | | | | | | | | | | | | | | | | | | | | | | | | | | | | | | | | |  |
|  | High |  | [R].GLLDTFKnLALNAAKSAG.[V] |  | HCD |  | N8(Deamidated) |  | 3.25 |  | 5.6e-3 |  | 2.0e-1 |  | 0.015 |  | 194.68 |  | 1.000 |  | b1+(8): 890.46; b1+(9): 1003.55; b1+(10): 1074.58; y1+(9): 802.44; y1+(8): 731.40; y1+(7): 618.32 |  | N8(Deamidated): 100 |  |  |  |  |  |  |  | 0.45 |  | 1 |  | Sequest HT (3) |  | 3 |  | 602.33337 |  | 2.90 |  | 40.2914 |  | 11226 |  | 11226 |  | TRINITY\_DN0\_c1\_g1\_i14.p1,TRINITY\_DN0\_c1\_g1\_i4.p1 |  | Image |  | Peak List |  |
|  | | | | | | | | | | | | | | | | | | | | | | | | | | | | | | | | | | | | | | | | | | | | | | | | | | | | | |  |
|  | High |  | [R].GLLDTFKNLALnAAKSAGVSVLNSLScKLSKTc.[-] |  | HCD |  | N12(Deamidated); C27(Dehydro); C33(Dehydro) |  | 3.72 |  | 3.9e-3 |  | 1.6e-1 |  | 0.071 |  | 135.45 |  | 0.927 |  | b1+(10): 1073.60 |  | N12(Deamidated): 92.75; C27(Dehydro): 100; C33(Dehydro): 100 |  |  |  |  |  |  |  | 0.07 |  | 1 |  | Sequest HT (3) |  | 4 |  | 842.20099 |  | 0.42 |  | 40.4174 |  | 11281 |  | 11281 |  | TRINITY\_DN0\_c1\_g1\_i14.p1,TRINITY\_DN0\_c1\_g1\_i4.p1 |  | Image |  | Peak List |  |
|  | | | | | | | | | | | | | | | | | | | | | | | | | | | | | | | | | | | | | | | | | | | | | | | | | | | | | |  |
|  | High |  | [R].GLLDTFKnLALNAAKSAG.[V] |  | HCD |  | N8(Deamidated) |  | 4.33 |  | 1.3e-3 |  | 7.7e-4 |  | 1.25 |  | 417.92 |  | 1.000 |  | b1+(8): 890.46; b1+(9): 1003.55; b1+(10): 1074.58; b1+(11): 1187.67; y1+(9): 802.44; y1+(7): 618.32; b2+(11): 594.34 |  | N8(Deamidated): 100 |  |  |  |  |  |  |  | 0.37 |  | 1 |  | Sequest HT (3) |  | 2 |  | 902.99286 |  | -1.05 |  | 40.4250 |  | 11284 |  | 11284 |  | TRINITY\_DN0\_c1\_g1\_i14.p1,TRINITY\_DN0\_c1\_g1\_i4.p1 |  | Image |  | Peak List |  |
|  | | | | | | | | | | | | | | | | | | | | | | | | | | | | | | | | | | | | | | | | | | | | | | | | | | | | | |  |
|  | High |  | [R].GLLDTFKnLALNAAKSAG.[V] |  | HCD |  | N8(Deamidated) |  | 3.11 |  | 3.1e-3 |  | 1.1e-1 |  | 0.168 |  | 166.66 |  | 1.000 |  | b1+(8): 890.46; b1+(9): 1003.55; b1+(10): 1074.58; y1+(8): 731.40; y1+(7): 618.32 |  | N8(Deamidated): 100 |  |  |  |  |  |  |  | 0.50 |  | 1 |  | Sequest HT (3) |  | 3 |  | 602.33234 |  | 1.17 |  | 40.4406 |  | 11292 |  | 11292 |  | TRINITY\_DN0\_c1\_g1\_i14.p1,TRINITY\_DN0\_c1\_g1\_i4.p1 |  | Image |  | Peak List |  |
|  | | | | | | | | | | | | | | | | | | | | | | | | | | | | | | | | | | | | | | | | | | | | | | | | | | | | | |  |
|  | High |  | [D].TFKnLALnAAKSAGVSVLNSLScKLSKTc.[-] |  | HCD |  | N4(Deamidated); N8(Deamidated); C23(Dehydro); C29(Dehydro) |  | 5.65 |  | 1.3e-3 |  | 1.7e-3 |  | 1.08 |  | 272.65 |  | 1.000 |  | b1+(8): 904.48; b1+(9): 975.51; b1+(10): 1046.55; y1+(14): 1480.75; y1+(13): 1393.72; y1+(12): 1294.65; y1+(11): 1181.57; b2+(15): 744.91; b2+(16): 788.42; y2+(12): 647.83; y2+(11): 591.29 |  | N4(Deamidated): 100; N8(Deamidated): 100; C23(Dehydro): 100; C29(Dehydro): 100 |  |  |  |  |  |  |  | 0.45 |  | 1 |  | Sequest HT (3) |  | 4 |  | 742.89233 |  | -0.24 |  | 40.4858 |  | 11313 |  | 11313 |  | TRINITY\_DN0\_c1\_g1\_i14.p1,TRINITY\_DN0\_c1\_g1\_i4.p1 |  | Image |  | Peak List |  |
|  | | | | | | | | | | | | | | | | | | | | | | | | | | | | | | | | | | | | | | | | | | | | | | | | | | | | | |  |
|  | High |  | [R].GLLDTFKNLALnAAKSAG.[V] |  | HCD |  | N12(Deamidated) |  | 3.66 |  | 1.3e-3 |  | 4.0e-4 |  | 1.39 |  | 398.87 |  | 1.000 |  | b1+(8): 889.48; b1+(9): 1002.56; b1+(10): 1073.60; b1+(11): 1186.68; y1+(9): 803.43; y1+(8): 732.39; y1+(7): 619.30; b2+(10): 537.30 |  | N12(Deamidated): 100 |  |  |  |  |  |  |  | 0.34 |  | 1 |  | Sequest HT (3) |  | 2 |  | 902.99323 |  | -0.65 |  | 40.5711 |  | 11352 |  | 11352 |  | TRINITY\_DN0\_c1\_g1\_i14.p1,TRINITY\_DN0\_c1\_g1\_i4.p1 |  | Image |  | Peak List |  |
|  | | | | | | | | | | | | | | | | | | | | | | | | | | | | | | | | | | | | | | | | | | | | | | | | | | | | | |  |
|  | High |  | [R].GLLDTFKNLALnAAKSAG.[V] |  | HCD |  | N12(Deamidated) |  | 3.96 |  | 4.7e-3 |  | 1.9e-1 |  | 0.036 |  | 144.64 |  | 1.000 |  | b1+(8): 889.48; b1+(9): 1002.56; b1+(10): 1073.60; y1+(9): 803.43; y1+(8): 732.39; y1+(7): 619.30 |  | N12(Deamidated): 100 |  |  |  |  |  |  |  | 0.48 |  | 1 |  | Sequest HT (3) |  | 3 |  | 602.33203 |  | 0.67 |  | 40.5877 |  | 11362 |  | 11362 |  | TRINITY\_DN0\_c1\_g1\_i14.p1,TRINITY\_DN0\_c1\_g1\_i4.p1 |  | Image |  | Peak List |  |
|  | | | | | | | | | | | | | | | | | | | | | | | | | | | | | | | | | | | | | | | | | | | | | | | | | | | | | |  |
|  | High |  | [R].GLLDTFKNLALnAAKSAG.[V] |  | HCD |  | N12(Deamidated) |  | 3.82 |  | 1.3e-3 |  | 1.7e-2 |  | 0.58 |  | 163.16 |  | 1.000 |  | b1+(8): 889.48; b1+(9): 1002.56; b1+(10): 1073.60; y1+(9): 803.43; y1+(8): 732.39; y1+(7): 619.30 |  | N12(Deamidated): 100 |  |  |  |  |  |  |  | 0.41 |  | 1 |  | Sequest HT (3) |  | 3 |  | 602.33203 |  | 0.67 |  | 40.5914 |  | 11365 |  | 11365 |  | TRINITY\_DN0\_c1\_g1\_i14.p1,TRINITY\_DN0\_c1\_g1\_i4.p1 |  | Image |  | Peak List |  |
|  | | | | | | | | | | | | | | | | | | | | | | | | | | | | | | | | | | | | | | | | | | | | | | | | | | | | | |  |
|  | High |  | [R].GLLDTFKnLALNAAKSA.[VG] |  | HCD |  | N8(Deamidated) |  | 4.06 |  | 1.3e-3 |  | 7.7e-3 |  | 0.749 |  | 245.62 |  | 1.000 |  | b1+(8): 890.46; b1+(9): 1003.55; b1+(10): 1074.58; y1+(6): 561.30 |  | N8(Deamidated): 100 |  |  |  |  |  |  |  | 0.39 |  | 1 |  | Sequest HT (3) |  | 3 |  | 583.32617 |  | 2.91 |  | 40.8042 |  | 11460 |  | 11460 |  | TRINITY\_DN24401\_c0\_g1\_i2.p14; TRINITY\_DN0\_c1\_g1\_i14.p1,TRINITY\_DN0\_c1\_g1\_i4.p1 |  | Image |  | Peak List |  |
|  | | | | | | | | | | | | | | | | | | | | | | | | | | | | | | | | | | | | | | | | | | | | | | | | | | | | | |  |
|  | High |  | [R].GLLDTFKnLAL.[N] |  | HCD |  | N8(Deamidated) |  | 2.80 |  | 1.3e-3 |  | 3.5e-2 |  | 0.416 |  | 209.64 |  | 1.000 |  |  |  | N8(Deamidated): 100 |  |  |  |  |  |  |  | 0.24 |  | 1 |  | Sequest HT (3) |  | 2 |  | 603.34412 |  | 2.77 |  | 40.8442 |  | 11481 |  | 11481 |  | TRINITY\_DN24401\_c0\_g1\_i2.p14; TRINITY\_DN6490\_c1\_g1\_i1.p1\_ORF1; TRINITY\_DN0\_c1\_g1\_i14.p1,TRINITY\_DN0\_c1\_g1\_i4.p1; TRINITY\_DN6490\_c1\_g1\_i1.p1 |  | Image |  | Peak List |  |
|  | | | | | | | | | | | | | | | | | | | | | | | | | | | | | | | | | | | | | | | | | | | | | | | | | | | | | |  |
|  | High |  | [R].GLLDTFKNLALnAAKSA.[VG] |  | HCD |  | N12(Deamidated) |  | 3.05 |  | 1.3e-3 |  | 4.2e-2 |  | 0.375 |  | 205.45 |  | 0.987 |  | b1+(8): 889.48; b1+(9): 1002.56; b1+(10): 1073.60; y1+(7): 675.37; y1+(6): 562.28 |  | N12(Deamidated): 98.69 |  |  |  |  |  |  |  | 0.09 |  | 1 |  | Sequest HT (3) |  | 3 |  | 583.32550 |  | 1.76 |  | 40.9474 |  | 11524 |  | 11524 |  | TRINITY\_DN24401\_c0\_g1\_i2.p14; TRINITY\_DN0\_c1\_g1\_i14.p1,TRINITY\_DN0\_c1\_g1\_i4.p1 |  | Image |  | Peak List |  |
|  | | | | | | | | | | | | | | | | | | | | | | | | | | | | | | | | | | | | | | | | | | | | | | | | | | | | | |  |
|  | High |  | [R].GLLDTFKnLALnAAKSAG.[V] |  | HCD |  | N8(Deamidated); N12(Deamidated) |  | 4.66 |  | 1.3e-3 |  | 2.1e-4 |  | 1.53 |  | 441.84 |  | 1.000 |  |  |  | N8(Deamidated): 100; N12(Deamidated): 100 |  |  |  |  |  |  |  | 0.54 |  | 1 |  | Sequest HT (3) |  | 2 |  | 903.48358 |  | -2.47 |  | 41.1319 |  | 11608 |  | 11608 |  | TRINITY\_DN0\_c1\_g1\_i14.p1,TRINITY\_DN0\_c1\_g1\_i4.p1 |  | Image |  | Peak List |  |
|  | | | | | | | | | | | | | | | | | | | | | | | | | | | | | | | | | | | | | | | | | | | | | | | | | | | | | |  |
|  | High |  | [G].LLDTFKNLALNAAKSAGVSVLNSLScKLSKTc.[-] |  | HCD |  | C26(Dehydro); C32(Dehydro) |  | 5.91 |  | 1.3e-3 |  | 1.0e-3 |  | 1.19 |  | 351.53 |  | 1.000 |  |  |  | C26(Dehydro): 100; C32(Dehydro): 100 |  |  |  |  |  |  |  | 0.65 |  | 1 |  | Sequest HT (3) |  | 4 |  | 827.69757 |  | -2.05 |  | 42.2323 |  | 12105 |  | 12105 |  | TRINITY\_DN0\_c1\_g1\_i14.p1,TRINITY\_DN0\_c1\_g1\_i4.p1 |  | Image |  | Peak List |  |
|  | | | | | | | | | | | | | | | | | | | | | | | | | | | | | | | | | | | | | | | | | | | | | | | | | | | | | |  |
|  | High |  | [G].LLDTFKNLALNAAKSAGVSVLNSLScKLSKTc.[-] |  | HCD |  | C26(Dehydro); C32(Dehydro) |  | 6.74 |  | 1.3e-3 |  | 8.2e-5 |  | 1.74 |  | 412.40 |  | 1.000 |  |  |  | C26(Dehydro): 100; C32(Dehydro): 100 |  |  |  |  |  |  |  | 0.72 |  | 1 |  | Sequest HT (3) |  | 3 |  | 1103.26453 |  | 1.14 |  | 42.2348 |  | 12107 |  | 12107 |  | TRINITY\_DN0\_c1\_g1\_i14.p1,TRINITY\_DN0\_c1\_g1\_i4.p1 |  | Image |  | Peak List |  |
|  | | | | | | | | | | | | | | | | | | | | | | | | | | | | | | | | | | | | | | | | | | | | | | | | | | | | | |  |
|  | High |  | [R].GLLDTFKNLALNAAKSAGVSVLNSLScKLSKTc.[-] |  | HCD |  | C27(Dehydro); C33(Dehydro) |  | 4.81 |  | 3.9e-3 |  | 1.8e-1 |  | 0.049 |  | 200.66 |  | 1.000 |  |  |  | C27(Dehydro): 100; C33(Dehydro): 100 |  |  |  |  |  |  |  | 0.56 |  | 1 |  | Sequest HT (3) |  | 4 |  | 841.95679 |  | 2.56 |  | 43.8131 |  | 12742 |  | 12742 |  | TRINITY\_DN0\_c1\_g1\_i14.p1,TRINITY\_DN0\_c1\_g1\_i4.p1 |  | Image |  | Peak List |  |
|  | | | | | | | | | | | | | | | | | | | | | | | | | | | | | | | | | | | | | | | | | | | | | | | | | | | | | |  |
|  | High |  | [R].GLLDTFKNLALNAAKSAGVSVLNSLScKLSKTc.[-] |  | HCD |  | C27(Dehydro); C33(Dehydro) |  | 4.11 |  | 1.3e-3 |  | 2.0e-3 |  | 1.04 |  | 223.02 |  | 1.000 |  |  |  | C27(Dehydro): 100; C33(Dehydro): 100 |  |  |  |  |  |  |  | 0.58 |  | 1 |  | Sequest HT (3) |  | 3 |  | 1122.27087 |  | 0.40 |  | 43.8221 |  | 12746 |  | 12746 |  | TRINITY\_DN0\_c1\_g1\_i14.p1,TRINITY\_DN0\_c1\_g1\_i4.p1 |  | Image |  | Peak List |  |
|  | | | | | | | | | | | | | | | | | | | | | | | | | | | | | | | | | | | | | | | | | | | | | | | | | | | | | |  |
|  | High |  | [R].GLLDTFKNLALNAAKSAGVSVLNSLScKLSKTc.[-] |  | HCD |  | C27(Dehydro); C33(Dehydro) |  | 4.02 |  | 1.3e-3 |  | 3.0e-2 |  | 0.452 |  | 267.86 |  | 1.000 |  |  |  | C27(Dehydro): 100; C33(Dehydro): 100 |  |  |  |  |  |  |  | 0.56 |  | 1 |  | Sequest HT (3) |  | 4 |  | 841.95703 |  | 2.85 |  | 43.9497 |  | 12798 |  | 12798 |  | TRINITY\_DN0\_c1\_g1\_i14.p1,TRINITY\_DN0\_c1\_g1\_i4.p1 |  | Image |  | Peak List |  |
|  | | | | | | | | | | | | | | | | | | | | | | | | | | | | | | | | | | | | | | | | | | | | | | | | | | | | | |  |
|  | High |  | [R].GLLDTFKNLALNAAKSAGVSVLNSLScKLSKTc.[-] |  | HCD |  | C27(Dehydro); C33(Dehydro) |  | 6.61 |  | 1.3e-3 |  | 6.1e-3 |  | 0.798 |  | 322.79 |  | 1.000 |  |  |  | C27(Dehydro): 100; C33(Dehydro): 100 |  |  |  |  |  |  |  | 0.68 |  | 1 |  | Sequest HT (3) |  | 4 |  | 841.95587 |  | 1.47 |  | 48.8601 |  | 14891 |  | 14891 |  | TRINITY\_DN0\_c1\_g1\_i14.p1,TRINITY\_DN0\_c1\_g1\_i4.p1 |  | Image |  | Peak List |  |
|  | | | | | | | | | | | | | | | | | | | | | | | | | | | | | | | | | | | | | | | | | | | | | | | | | | | | | |  |
|  | High |  | [R].GLLDTFKNLALNAAKSAGVSVLNSLScKLSKTc.[-] |  | HCD |  | C27(Dehydro); C33(Dehydro) |  | 3.29 |  | 3.1e-3 |  | 1.4e-1 |  | 0.112 |  | 182.65 |  | 1.000 |  |  |  | C27(Dehydro): 100; C33(Dehydro): 100 |  |  |  |  |  |  |  | 0.57 |  | 1 |  | Sequest HT (3) |  | 5 |  | 673.76318 |  | -2.94 |  | 48.9847 |  | 14942 |  | 14942 |  | TRINITY\_DN0\_c1\_g1\_i14.p1,TRINITY\_DN0\_c1\_g1\_i4.p1 |  | Image |  | Peak List |  |
|  | | | | | | | | | | | | | | | | | | | | | | | | | | | | | | | | | | | | | | | | | | | | | | | | | | | | | |  |
|  | High |  | [R].GLLDTFKNLALNAAKSAGVSVLNSLScKLSKTc.[-] |  | HCD |  | C27(Dehydro); C33(Dehydro) |  | 7.26 |  | 1.3e-3 |  | 2.0e-3 |  | 1.04 |  | 347.53 |  | 1.000 |  |  |  | C27(Dehydro): 100; C33(Dehydro): 100 |  |  |  |  |  |  |  | 0.69 |  | 1 |  | Sequest HT (3) |  | 4 |  | 841.95490 |  | 0.31 |  | 49.0044 |  | 14950 |  | 14950 |  | TRINITY\_DN0\_c1\_g1\_i14.p1,TRINITY\_DN0\_c1\_g1\_i4.p1 |  | Image |  | Peak List |  |
|  | | | | | | | | | | | | | | | | | | | | | | | | | | | | | | | | | | | | | | | | | | | | | | | | | | | | | |  |
|  | High |  | [R].GLLDTFKNLALNAAKSAGVSVLNSLScKLSKTc.[-] |  | HCD |  | C27(Dehydro); C33(Dehydro) |  | 7.37 |  | 1.3e-3 |  | 8.6e-6 |  | 2.23 |  | 449.54 |  | 1.000 |  |  |  | C27(Dehydro): 100; C33(Dehydro): 100 |  |  |  |  |  |  |  | 0.74 |  | 1 |  | Sequest HT (3) |  | 3 |  | 1122.26953 |  | -0.79 |  | 49.0278 |  | 14961 |  | 14961 |  | TRINITY\_DN0\_c1\_g1\_i14.p1,TRINITY\_DN0\_c1\_g1\_i4.p1 |  | Image |  | Peak List |  |
|  | | | | | | | | | | | | | | | | | | | | | | | | | | | | | | | | | | | | | | | | | | | | | | | | | | | | | |  |
|  | High |  | [R].GLLDTFKNLALNAAKSAGVSVLNSLScKLSKTc.[-] |  | HCD |  | C27(Dehydro); C33(Dehydro) |  | 3.83 |  | 1.3e-3 |  | 3.8e-5 |  | 1.9 |  | 448.31 |  | 1.000 |  |  |  | C27(Dehydro): 100; C33(Dehydro): 100 |  |  |  |  |  |  |  | 0.68 |  | 1 |  | Sequest HT (3) |  | 2 |  | 1682.90039 |  | -0.95 |  | 49.0725 |  | 14978 |  | 14978 |  | TRINITY\_DN0\_c1\_g1\_i14.p1,TRINITY\_DN0\_c1\_g1\_i4.p1 |  | Image |  | Peak List |  |
|  | | | | | | | | | | | | | | | | | | | | | | | | | | | | | | | | | | | | | | | | | | | | | | | | | | | | | |  |
|  | High |  | [R].GLLDTFKNLALNAAKSAGVSVLNSLScKLSKTc.[-] |  | HCD |  | C27(Dehydro); C33(Dehydro) |  | 3.78 |  | 3.9e-3 |  | 1.5e-1 |  | 0.083 |  | 160.39 |  | 1.000 |  |  |  | C27(Dehydro): 100; C33(Dehydro): 100 |  |  |  |  |  |  |  | 0.53 |  | 1 |  | Sequest HT (3) |  | 5 |  | 673.76471 |  | -0.67 |  | 49.1455 |  | 15005 |  | 15005 |  | TRINITY\_DN0\_c1\_g1\_i14.p1,TRINITY\_DN0\_c1\_g1\_i4.p1 |  | Image |  | Peak List |  |
|  | | | | | | | | | | | | | | | | | | | | | | | | | | | | | | | | | | | | | | | | | | | | | | | | | | | | | |  |
|  | High |  | [R].GLLDTFKNLALNAAKSAGVSVLNSLScKLSKTc.[-] |  | HCD |  | C27(Dehydro); C33(Dehydro) |  | 7.46 |  | 1.3e-3 |  | 1.2e-3 |  | 1.16 |  | 319.17 |  | 1.000 |  |  |  | C27(Dehydro): 100; C33(Dehydro): 100 |  |  |  |  |  |  |  | 0.71 |  | 1 |  | Sequest HT (3) |  | 4 |  | 841.95551 |  | 1.04 |  | 49.1564 |  | 15010 |  | 15010 |  | TRINITY\_DN0\_c1\_g1\_i14.p1,TRINITY\_DN0\_c1\_g1\_i4.p1 |  | Image |  | Peak List |  |
|  | | | | | | | | | | | | | | | | | | | | | | | | | | | | | | | | | | | | | | | | | | | | | | | | | | | | | |  |
|  | High |  | [R].GLLDTFKNLALNAAKSAGVSVLNSLScKLSKTc.[-] |  | HCD |  | C27(Dehydro); C33(Dehydro) |  | 6.04 |  | 1.3e-3 |  | 5.1e-6 |  | 2.34 |  | 453.17 |  | 1.000 |  |  |  | C27(Dehydro): 100; C33(Dehydro): 100 |  |  |  |  |  |  |  | 0.74 |  | 1 |  | Sequest HT (3) |  | 3 |  | 1122.27002 |  | -0.36 |  | 49.1691 |  | 15017 |  | 15017 |  | TRINITY\_DN0\_c1\_g1\_i14.p1,TRINITY\_DN0\_c1\_g1\_i4.p1 |  | Image |  | Peak List |  |
|  | | | | | | | | | | | | | | | | | | | | | | | | | | | | | | | | | | | | | | | | | | | | | | | | | | | | | |  |
|  | High |  | [R].GLLDTFKNLALNAAKSAGVSVLNSLScKLSKTc.[-] |  | HCD |  | C27(Dehydro); C33(Dehydro) |  | 4.04 |  | 1.3e-3 |  | 1.1e-2 |  | 0.675 |  | 221.99 |  | 1.000 |  |  |  | C27(Dehydro): 100; C33(Dehydro): 100 |  |  |  |  |  |  |  | 0.54 |  | 1 |  | Sequest HT (3) |  | 5 |  | 673.76410 |  | -1.58 |  | 49.2862 |  | 15062 |  | 15062 |  | TRINITY\_DN0\_c1\_g1\_i14.p1,TRINITY\_DN0\_c1\_g1\_i4.p1 |  | Image |  | Peak List |  |
|  | | | | | | | | | | | | | | | | | | | | | | | | | | | | | | | | | | | | | | | | | | | | | | | | | | | | | |  |
|  | High |  | [R].GLLDTFKNLALNAAKSAGVSVLNSLScKLSKTc.[-] |  | HCD |  | C27(Dehydro); C33(Dehydro) |  | 6.65 |  | 1.3e-3 |  | 3.1e-3 |  | 0.949 |  | 365.42 |  | 1.000 |  |  |  | C27(Dehydro): 100; C33(Dehydro): 100 |  |  |  |  |  |  |  | 0.70 |  | 1 |  | Sequest HT (3) |  | 4 |  | 841.95538 |  | 0.89 |  | 49.2939 |  | 15065 |  | 15065 |  | TRINITY\_DN0\_c1\_g1\_i14.p1,TRINITY\_DN0\_c1\_g1\_i4.p1 |  | Image |  | Peak List |  |
|  | | | | | | | | | | | | | | | | | | | | | | | | | | | | | | | | | | | | | | | | | | | | | | | | | | | | | |  |
|  | High |  | [R].GLLDTFKNLALNAAKSAGVSVLNSLScKLSKTc.[-] |  | HCD |  | C27(Dehydro); C33(Dehydro) |  | 6.71 |  | 1.3e-3 |  | 5.5e-5 |  | 1.82 |  | 439.24 |  | 1.000 |  |  |  | C27(Dehydro): 100; C33(Dehydro): 100 |  |  |  |  |  |  |  | 0.68 |  | 1 |  | Sequest HT (3) |  | 3 |  | 1122.26917 |  | -1.12 |  | 49.3140 |  | 15073 |  | 15073 |  | TRINITY\_DN0\_c1\_g1\_i14.p1,TRINITY\_DN0\_c1\_g1\_i4.p1 |  | Image |  | Peak List |  |
|  | | | | | | | | | | | | | | | | | | | | | | | | | | | | | | | | | | | | | | | | | | | | | | | | | | | | | |  |
|  | High |  | [R].GLLDTFKNLALNAAKSAGVSVLNSLScKLSKTc.[-] |  | HCD |  | C27(Dehydro); C33(Dehydro) |  | 4.92 |  | 1.3e-3 |  | 2.6e-2 |  | 0.478 |  | 209.28 |  | 1.000 |  |  |  | C27(Dehydro): 100; C33(Dehydro): 100 |  |  |  |  |  |  |  | 0.62 |  | 1 |  | Sequest HT (3) |  | 5 |  | 673.76434 |  | -1.22 |  | 49.4283 |  | 15117 |  | 15117 |  | TRINITY\_DN0\_c1\_g1\_i14.p1,TRINITY\_DN0\_c1\_g1\_i4.p1 |  | Image |  | Peak List |  |
|  | | | | | | | | | | | | | | | | | | | | | | | | | | | | | | | | | | | | | | | | | | | | | | | | | | | | | |  |
|  | High |  | [R].GLLDTFKNLALNAAKSAGVSVLNSLScKLSKTc.[-] |  | HCD |  | C27(Dehydro); C33(Dehydro) |  | 3.88 |  | 1.3e-3 |  | 5.3e-2 |  | 0.326 |  | 162.06 |  | 1.000 |  |  |  | C27(Dehydro): 100; C33(Dehydro): 100 |  |  |  |  |  |  |  | 0.62 |  | 1 |  | Sequest HT (3) |  | 4 |  | 841.95587 |  | 1.47 |  | 49.4423 |  | 15123 |  | 15123 |  | TRINITY\_DN0\_c1\_g1\_i14.p1,TRINITY\_DN0\_c1\_g1\_i4.p1 |  | Image |  | Peak List |  |
|  | | | | | | | | | | | | | | | | | | | | | | | | | | | | | | | | | | | | | | | | | | | | | | | | | | | | | |  |
|  | High |  | [R].GLLDTFKNLALNAAKSAGVSVLNSLScKLSKTc.[-] |  | HCD |  | C27(Dehydro); C33(Dehydro) |  | 6.16 |  | 1.3e-3 |  | 1.6e-5 |  | 2.1 |  | 543.14 |  | 1.000 |  |  |  | C27(Dehydro): 100; C33(Dehydro): 100 |  |  |  |  |  |  |  | 0.65 |  | 1 |  | Sequest HT (3) |  | 3 |  | 1122.26892 |  | -1.34 |  | 49.4524 |  | 15126 |  | 15126 |  | TRINITY\_DN0\_c1\_g1\_i14.p1,TRINITY\_DN0\_c1\_g1\_i4.p1 |  | Image |  | Peak List |  |
|  | | | | | | | | | | | | | | | | | | | | | | | | | | | | | | | | | | | | | | | | | | | | | | | | | | | | | |  |
|  | High |  | [R].GLLDTFKNLALNAAKSAGVSVLNSLScKLSKTc.[-] |  | HCD |  | C27(Dehydro); C33(Dehydro) |  | 3.69 |  | 1.3e-3 |  | 1.4e-3 |  | 1.12 |  | 373.68 |  | 1.000 |  |  |  | C27(Dehydro): 100; C33(Dehydro): 100 |  |  |  |  |  |  |  | 0.72 |  | 1 |  | Sequest HT (3) |  | 2 |  | 1682.90137 |  | -0.37 |  | 49.5618 |  | 15173 |  | 15173 |  | TRINITY\_DN0\_c1\_g1\_i14.p1,TRINITY\_DN0\_c1\_g1\_i4.p1 |  | Image |  | Peak List |  |
|  | | | | | | | | | | | | | | | | | | | | | | | | | | | | | | | | | | | | | | | | | | | | | | | | | | | | | |  |
|  | High |  | [R].GLLDTFKNLALNAAKSAGVSVLNSLScKLSKTc.[-] |  | HCD |  | C27(Dehydro); C33(Dehydro) |  | 3.74 |  | 1.3e-3 |  | 8.0e-3 |  | 0.74 |  | 234.98 |  | 1.000 |  |  |  | C27(Dehydro): 100; C33(Dehydro): 100 |  |  |  |  |  |  |  | 0.56 |  | 1 |  | Sequest HT (3) |  | 5 |  | 673.76465 |  | -0.76 |  | 49.5714 |  | 15177 |  | 15177 |  | TRINITY\_DN0\_c1\_g1\_i14.p1,TRINITY\_DN0\_c1\_g1\_i4.p1 |  | Image |  | Peak List |  |
|  | | | | | | | | | | | | | | | | | | | | | | | | | | | | | | | | | | | | | | | | | | | | | | | | | | | | | |  |
|  | High |  | [R].GLLDTFKNLALNAAKSAGVSVLNSLScKLSKTc.[-] |  | HCD |  | C27(Dehydro); C33(Dehydro) |  | 6.43 |  | 1.3e-3 |  | 2.2e-3 |  | 1.02 |  | 392.77 |  | 1.000 |  |  |  | C27(Dehydro): 100; C33(Dehydro): 100 |  |  |  |  |  |  |  | 0.70 |  | 1 |  | Sequest HT (3) |  | 4 |  | 841.95618 |  | 1.83 |  | 49.5782 |  | 15179 |  | 15179 |  | TRINITY\_DN0\_c1\_g1\_i14.p1,TRINITY\_DN0\_c1\_g1\_i4.p1 |  | Image |  | Peak List |  |
|  | | | | | | | | | | | | | | | | | | | | | | | | | | | | | | | | | | | | | | | | | | | | | | | | | | | | | |  |
|  | High |  | [R].GLLDTFKNLALNAAKSAGVSVLNSLScKLSKTc.[-] |  | HCD |  | C27(Dehydro); C33(Dehydro) |  | 6.28 |  | 1.3e-3 |  | 5.2e-6 |  | 2.34 |  | 430.75 |  | 1.000 |  |  |  | C27(Dehydro): 100; C33(Dehydro): 100 |  |  |  |  |  |  |  | 0.72 |  | 1 |  | Sequest HT (3) |  | 3 |  | 1122.27014 |  | -0.25 |  | 49.5880 |  | 15183 |  | 15183 |  | TRINITY\_DN0\_c1\_g1\_i14.p1,TRINITY\_DN0\_c1\_g1\_i4.p1 |  | Image |  | Peak List |  |
|  | | | | | | | | | | | | | | | | | | | | | | | | | | | | | | | | | | | | | | | | | | | | | | | | | | | | | |  |
|  | High |  | [R].GLLDTFKNLALNAAKSAGVSVLNSLScKLSKTc.[-] |  | HCD |  | C27(Dehydro); C33(Dehydro) |  | 7.01 |  | 1.3e-3 |  | 2.2e-3 |  | 1.02 |  | 347.28 |  | 1.000 |  |  |  | C27(Dehydro): 100; C33(Dehydro): 100 |  |  |  |  |  |  |  | 0.68 |  | 1 |  | Sequest HT (3) |  | 4 |  | 841.95587 |  | 1.47 |  | 49.7134 |  | 15234 |  | 15234 |  | TRINITY\_DN0\_c1\_g1\_i14.p1,TRINITY\_DN0\_c1\_g1\_i4.p1 |  | Image |  | Peak List |  |
|  | | | | | | | | | | | | | | | | | | | | | | | | | | | | | | | | | | | | | | | | | | | | | | | | | | | | | |  |
|  | High |  | [R].GLLDTFKNLALNAAKSAGVSVLNSLScKLSKTc.[-] |  | HCD |  | C27(Dehydro); C33(Dehydro) |  | 3.51 |  | 3.1e-3 |  | 1.3e-1 |  | 0.12 |  | 174.41 |  | 1.000 |  |  |  | C27(Dehydro): 100; C33(Dehydro): 100 |  |  |  |  |  |  |  | 0.44 |  | 1 |  | Sequest HT (3) |  | 5 |  | 673.76453 |  | -0.95 |  | 49.7159 |  | 15236 |  | 15236 |  | TRINITY\_DN0\_c1\_g1\_i14.p1,TRINITY\_DN0\_c1\_g1\_i4.p1 |  | Image |  | Peak List |  |
|  | | | | | | | | | | | | | | | | | | | | | | | | | | | | | | | | | | | | | | | | | | | | | | | | | | | | | |  |
|  | High |  | [R].GLLDTFKNLALNAAKSAGVSVLNSLScKLSKTc.[-] |  | HCD |  | C27(Dehydro); C33(Dehydro) |  | 6.36 |  | 1.3e-3 |  | 3.4e-5 |  | 1.93 |  | 438.38 |  | 1.000 |  |  |  | C27(Dehydro): 100; C33(Dehydro): 100 |  |  |  |  |  |  |  | 0.69 |  | 1 |  | Sequest HT (3) |  | 3 |  | 1122.26941 |  | -0.90 |  | 49.7249 |  | 15240 |  | 15240 |  | TRINITY\_DN0\_c1\_g1\_i14.p1,TRINITY\_DN0\_c1\_g1\_i4.p1 |  | Image |  | Peak List |  |
|  | | | | | | | | | | | | | | | | | | | | | | | | | | | | | | | | | | | | | | | | | | | | | | | | | | | | | |  |
|  | High |  | [R].GLLDTFKNLALNAAKSAGVSVLNSLScKLSKTc.[-] |  | HCD |  | C27(Dehydro); C33(Dehydro) |  | 5.59 |  | 1.3e-3 |  | 5.9e-5 |  | 1.81 |  | 427.51 |  | 1.000 |  |  |  | C27(Dehydro): 100; C33(Dehydro): 100 |  |  |  |  |  |  |  | 0.70 |  | 1 |  | Sequest HT (3) |  | 3 |  | 1122.26929 |  | -1.01 |  | 49.8650 |  | 15295 |  | 15295 |  | TRINITY\_DN0\_c1\_g1\_i14.p1,TRINITY\_DN0\_c1\_g1\_i4.p1 |  | Image |  | Peak List |  |
|  | | | | | | | | | | | | | | | | | | | | | | | | | | | | | | | | | | | | | | | | | | | | | | | | | | | | | |  |
|  | High |  | [R].GLLDTFKNLALNAAKSAGVSVLNSLScKLSKTc.[-] |  | HCD |  | C27(Dehydro); C33(Dehydro) |  | 3.65 |  | 3.9e-3 |  | 1.7e-1 |  | 0.055 |  | 166.18 |  | 1.000 |  |  |  | C27(Dehydro): 100; C33(Dehydro): 100 |  |  |  |  |  |  |  | 0.47 |  | 1 |  | Sequest HT (3) |  | 5 |  | 673.76422 |  | -1.40 |  | 49.8675 |  | 15297 |  | 15297 |  | TRINITY\_DN0\_c1\_g1\_i14.p1,TRINITY\_DN0\_c1\_g1\_i4.p1 |  | Image |  | Peak List |  |
|  | | | | | | | | | | | | | | | | | | | | | | | | | | | | | | | | | | | | | | | | | | | | | | | | | | | | | |  |
|  | High |  | [R].GLLDTFKNLALNAAKSAGVSVLNSLScKLSKTc.[-] |  | HCD |  | C27(Dehydro); C33(Dehydro) |  | 6.36 |  | 1.3e-3 |  | 1.1e-4 |  | 1.67 |  | 339.71 |  | 1.000 |  |  |  | C27(Dehydro): 100; C33(Dehydro): 100 |  |  |  |  |  |  |  | 0.72 |  | 1 |  | Sequest HT (3) |  | 4 |  | 841.95593 |  | 1.54 |  | 49.9963 |  | 15349 |  | 15349 |  | TRINITY\_DN0\_c1\_g1\_i14.p1,TRINITY\_DN0\_c1\_g1\_i4.p1 |  | Image |  | Peak List |  |
|  | | | | | | | | | | | | | | | | | | | | | | | | | | | | | | | | | | | | | | | | | | | | | | | | | | | | | |  |
|  | High |  | [R].GLLDTFKNLALNAAKSAGVSVLNSLScKLSKTc.[-] |  | HCD |  | C27(Dehydro); C33(Dehydro) |  | 6.84 |  | 1.3e-3 |  | 7.6e-6 |  | 2.26 |  | 477.13 |  | 1.000 |  |  |  | C27(Dehydro): 100; C33(Dehydro): 100 |  |  |  |  |  |  |  | 0.69 |  | 1 |  | Sequest HT (3) |  | 3 |  | 1122.26929 |  | -1.01 |  | 50.0040 |  | 15352 |  | 15352 |  | TRINITY\_DN0\_c1\_g1\_i14.p1,TRINITY\_DN0\_c1\_g1\_i4.p1 |  | Image |  | Peak List |  |
|  | | | | | | | | | | | | | | | | | | | | | | | | | | | | | | | | | | | | | | | | | | | | | | | | | | | | | |  |
|  | High |  | [R].GLLDTFKNLALNAAKSAGVSVLNSLScKLSKTc.[-] |  | HCD |  | C27(Dehydro); C33(Dehydro) |  | 3.93 |  | 1.3e-3 |  | 2.0e-2 |  | 0.535 |  | 239.69 |  | 1.000 |  |  |  | C27(Dehydro): 100; C33(Dehydro): 100 |  |  |  |  |  |  |  | 0.56 |  | 1 |  | Sequest HT (3) |  | 5 |  | 673.76483 |  | -0.49 |  | 50.0168 |  | 15356 |  | 15356 |  | TRINITY\_DN0\_c1\_g1\_i14.p1,TRINITY\_DN0\_c1\_g1\_i4.p1 |  | Image |  | Peak List |  |
|  | | | | | | | | | | | | | | | | | | | | | | | | | | | | | | | | | | | | | | | | | | | | | | | | | | | | | |  |
|  | High |  | [R].GLLDTFKNLALNAAKSAGVSVLNSLScKLSKTc.[-] |  | HCD |  | C27(Dehydro); C33(Dehydro) |  | 7.49 |  | 1.3e-3 |  | 7.3e-4 |  | 1.26 |  | 360.93 |  | 1.000 |  |  |  | C27(Dehydro): 100; C33(Dehydro): 100 |  |  |  |  |  |  |  | 0.70 |  | 1 |  | Sequest HT (3) |  | 4 |  | 841.95551 |  | 1.04 |  | 50.1381 |  | 15403 |  | 15403 |  | TRINITY\_DN0\_c1\_g1\_i14.p1,TRINITY\_DN0\_c1\_g1\_i4.p1 |  | Image |  | Peak List |  |
|  | | | | | | | | | | | | | | | | | | | | | | | | | | | | | | | | | | | | | | | | | | | | | | | | | | | | | |  |
|  | High |  | [R].GLLDTFKNLALNAAKSAGVSVLNSLScKLSKTc.[-] |  | HCD |  | C27(Dehydro); C33(Dehydro) |  | 5.40 |  | 1.3e-3 |  | 1.3e-4 |  | 1.63 |  | 459.23 |  | 1.000 |  |  |  | C27(Dehydro): 100; C33(Dehydro): 100 |  |  |  |  |  |  |  | 0.71 |  | 1 |  | Sequest HT (3) |  | 3 |  | 1122.26953 |  | -0.79 |  | 50.1486 |  | 15407 |  | 15407 |  | TRINITY\_DN0\_c1\_g1\_i14.p1,TRINITY\_DN0\_c1\_g1\_i4.p1 |  | Image |  | Peak List |  |
|  | | | | | | | | | | | | | | | | | | | | | | | | | | | | | | | | | | | | | | | | | | | | | | | | | | | | | |  |
|  | High |  | [R].GLLDTFKNLALNAAKSAGVSVLNSLScKLSKTc.[-] |  | HCD |  | C27(Dehydro); C33(Dehydro) |  | 4.03 |  | 1.3e-3 |  | 2.7e-2 |  | 0.475 |  | 191.81 |  | 1.000 |  |  |  | C27(Dehydro): 100; C33(Dehydro): 100 |  |  |  |  |  |  |  | 0.61 |  | 1 |  | Sequest HT (3) |  | 5 |  | 673.76459 |  | -0.85 |  | 50.1620 |  | 15412 |  | 15412 |  | TRINITY\_DN0\_c1\_g1\_i14.p1,TRINITY\_DN0\_c1\_g1\_i4.p1 |  | Image |  | Peak List |  |
|  | | | | | | | | | | | | | | | | | | | | | | | | | | | | | | | | | | | | | | | | | | | | | | | | | | | | | |  |
|  | High |  | [R].GLLDTFKNLALNAAKSAGVSVLNSLScKLSKTc.[-] |  | HCD |  | C27(Dehydro); C33(Dehydro) |  | 7.63 |  | 1.3e-3 |  | 1.6e-4 |  | 1.59 |  | 449.32 |  | 1.000 |  |  |  | C27(Dehydro): 100; C33(Dehydro): 100 |  |  |  |  |  |  |  | 0.75 |  | 1 |  | Sequest HT (3) |  | 4 |  | 841.95465 |  | 0.02 |  | 50.2761 |  | 15457 |  | 15457 |  | TRINITY\_DN0\_c1\_g1\_i14.p1,TRINITY\_DN0\_c1\_g1\_i4.p1 |  | Image |  | Peak List |  |
|  | | | | | | | | | | | | | | | | | | | | | | | | | | | | | | | | | | | | | | | | | | | | | | | | | | | | | |  |
|  | High |  | [R].GLLDTFKNLALNAAKSAGVSVLNSLScKLSKTc.[-] |  | HCD |  | C27(Dehydro); C33(Dehydro) |  | 6.46 |  | 1.3e-3 |  | 8.8e-6 |  | 2.22 |  | 496.24 |  | 1.000 |  |  |  | C27(Dehydro): 100; C33(Dehydro): 100 |  |  |  |  |  |  |  | 0.70 |  | 1 |  | Sequest HT (3) |  | 3 |  | 1122.26868 |  | -1.55 |  | 50.2863 |  | 15462 |  | 15462 |  | TRINITY\_DN0\_c1\_g1\_i14.p1,TRINITY\_DN0\_c1\_g1\_i4.p1 |  | Image |  | Peak List |  |
|  | | | | | | | | | | | | | | | | | | | | | | | | | | | | | | | | | | | | | | | | | | | | | | | | | | | | | |  |
|  | High |  | [R].GLLDTFKNLALNAAKSAGVSVLNSLScKLSKTc.[-] |  | HCD |  | C27(Dehydro); C33(Dehydro) |  | 4.18 |  | 3.1e-3 |  | 8.8e-2 |  | 0.212 |  | 208.35 |  | 1.000 |  |  |  | C27(Dehydro): 100; C33(Dehydro): 100 |  |  |  |  |  |  |  | 0.54 |  | 1 |  | Sequest HT (3) |  | 5 |  | 673.76453 |  | -0.95 |  | 50.3048 |  | 15469 |  | 15469 |  | TRINITY\_DN0\_c1\_g1\_i14.p1,TRINITY\_DN0\_c1\_g1\_i4.p1 |  | Image |  | Peak List |  |
|  | | | | | | | | | | | | | | | | | | | | | | | | | | | | | | | | | | | | | | | | | | | | | | | | | | | | | |  |
|  | High |  | [R].GLLDTFKNLALNAAKSAGVSVLNSLScKLSKTc.[-] |  | HCD |  | C27(Dehydro); C33(Dehydro) |  | 5.67 |  | 1.3e-3 |  | 4.7e-3 |  | 0.855 |  | 295.19 |  | 1.000 |  |  |  | C27(Dehydro): 100; C33(Dehydro): 100 |  |  |  |  |  |  |  | 0.74 |  | 1 |  | Sequest HT (3) |  | 4 |  | 841.95435 |  | -0.34 |  | 50.4140 |  | 15512 |  | 15512 |  | TRINITY\_DN0\_c1\_g1\_i14.p1,TRINITY\_DN0\_c1\_g1\_i4.p1 |  | Image |  | Peak List |  |
|  | | | | | | | | | | | | | | | | | | | | | | | | | | | | | | | | | | | | | | | | | | | | | | | | | | | | | |  |
|  | High |  | [R].GLLDTFKNLALNAAKSAGVSVLNSLScKLSKTc.[-] |  | HCD |  | C27(Dehydro); C33(Dehydro) |  | 5.50 |  | 1.3e-3 |  | 5.9e-5 |  | 1.81 |  | 417.63 |  | 1.000 |  |  |  | C27(Dehydro): 100; C33(Dehydro): 100 |  |  |  |  |  |  |  | 0.67 |  | 1 |  | Sequest HT (3) |  | 3 |  | 1122.26868 |  | -1.55 |  | 50.4255 |  | 15518 |  | 15518 |  | TRINITY\_DN0\_c1\_g1\_i14.p1,TRINITY\_DN0\_c1\_g1\_i4.p1 |  | Image |  | Peak List |  |
|  | | | | | | | | | | | | | | | | | | | | | | | | | | | | | | | | | | | | | | | | | | | | | | | | | | | | | |  |
|  | High |  | [R].GLLDTFKNLALNAAKSAGVSVLNSLScKLSKTc.[-] |  | HCD |  | C27(Dehydro); C33(Dehydro) |  | 5.64 |  | 1.3e-3 |  | 1.8e-2 |  | 0.565 |  | 274.26 |  | 1.000 |  |  |  | C27(Dehydro): 100; C33(Dehydro): 100 |  |  |  |  |  |  |  | 0.67 |  | 1 |  | Sequest HT (3) |  | 4 |  | 841.95361 |  | -1.21 |  | 50.6509 |  | 15604 |  | 15604 |  | TRINITY\_DN0\_c1\_g1\_i14.p1,TRINITY\_DN0\_c1\_g1\_i4.p1 |  | Image |  | Peak List |  |
|  | | | | | | | | | | | | | | | | | | | | | | | | | | | | | | | | | | | | | | | | | | | | | | | | | | | | | |  |
|  | High |  | [R].GLLDTFKNLALNAAKSAGVSVLNSLScKLSKTc.[-] |  | HCD |  | C27(Dehydro); C33(Dehydro) |  | 5.71 |  | 1.3e-3 |  | 6.7e-4 |  | 1.28 |  | 316.46 |  | 1.000 |  |  |  | C27(Dehydro): 100; C33(Dehydro): 100 |  |  |  |  |  |  |  | 0.66 |  | 1 |  | Sequest HT (3) |  | 4 |  | 841.95490 |  | 0.31 |  | 50.7998 |  | 15661 |  | 15661 |  | TRINITY\_DN0\_c1\_g1\_i14.p1,TRINITY\_DN0\_c1\_g1\_i4.p1 |  | Image |  | Peak List |  |
|  | | | | | | | | | | | | | | | | | | | | | | | | | | | | | | | | | | | | | | | | | | | | | | | | | | | | | |  |
|  | High |  | [R].GLLDTFKNLALnAAKSAGVSVLNSLScKLSKTc.[-] |  | HCD |  | N12(Deamidated); C27(Dehydro); C33(Dehydro) |  | 6.26 |  | 1.3e-3 |  | 2.4e-3 |  | 0.998 |  | 310.26 |  | 1.000 |  | b1+(8): 889.48; b1+(9): 1002.56; b1+(10): 1073.60; b1+(11): 1186.68; b2+(10): 537.30 |  | N12(Deamidated): 100; C27(Dehydro): 100; C33(Dehydro): 100 |  |  |  |  |  |  |  | 0.22 |  | 1 |  | Sequest HT (3) |  | 4 |  | 842.20160 |  | 1.14 |  | 50.9441 |  | 15717 |  | 15717 |  | TRINITY\_DN0\_c1\_g1\_i14.p1,TRINITY\_DN0\_c1\_g1\_i4.p1 |  | Image |  | Peak List |  |
|  | | | | | | | | | | | | | | | | | | | | | | | | | | | | | | | | | | | | | | | | | | | | | | | | | | | | | |  |
|  | High |  | [R].GLLDTFKNLALnAAKSAGVSVLNSLScKLSKTc.[-] |  | HCD |  | N12(Deamidated); C27(Dehydro); C33(Dehydro) |  | 6.86 |  | 1.3e-3 |  | 3.9e-6 |  | 2.4 |  | 344.31 |  | 1.000 |  | b1+(8): 889.48; b1+(9): 1002.56; b1+(10): 1073.60; b1+(11): 1186.68; b2+(10): 537.30 |  | N12(Deamidated): 100; C27(Dehydro): 100; C33(Dehydro): 100 |  |  |  |  |  |  |  | 0.14 |  | 1 |  | Sequest HT (3) |  | 3 |  | 1122.59900 |  | 0.51 |  | 50.9812 |  | 15732 |  | 15732 |  | TRINITY\_DN0\_c1\_g1\_i14.p1,TRINITY\_DN0\_c1\_g1\_i4.p1 |  | Image |  | Peak List |  |
|  | | | | | | | | | | | | | | | | | | | | | | | | | | | | | | | | | | | | | | | | | | | | | | | | | | | | | |  |
|  | High |  | [R].GLLDTFKNLALnAAKSAGVSVLNSLScKLSKTc.[-] |  | HCD |  | N12(Deamidated); C27(Dehydro); C33(Dehydro) |  | 3.58 |  | 4.7e-3 |  | 1.9e-1 |  | 0.029 |  | 148.50 |  | 0.963 |  | y1+(12): 1294.65; y1+(11): 1181.57; y2+(15): 790.41; y2+(12): 647.83; y2+(11): 591.29 |  | N12(Deamidated): 96.31; C27(Dehydro): 100; C33(Dehydro): 100 |  |  |  |  |  |  |  | 0.14 |  | 1 |  | Sequest HT (3) |  | 5 |  | 673.96063 |  | -1.98 |  | 51.0330 |  | 15754 |  | 15754 |  | TRINITY\_DN0\_c1\_g1\_i14.p1,TRINITY\_DN0\_c1\_g1\_i4.p1 |  | Image |  | Peak List |  |
|  | | | | | | | | | | | | | | | | | | | | | | | | | | | | | | | | | | | | | | | | | | | | | | | | | | | | | |  |
|  | High |  | [R].GLLDTFKNLALnAAKSAGVSVLNSLScKLSKTc.[-] |  | HCD |  | N12(Deamidated); C27(Dehydro); C33(Dehydro) |  | 6.67 |  | 1.3e-3 |  | 3.0e-3 |  | 0.954 |  | 328.47 |  | 1.000 |  | b1+(12): 1301.71; b1+(13): 1372.75; b1+(14): 1443.78; y1+(14): 1480.75; y1+(12): 1294.65; y1+(11): 1181.57; b2+(12): 651.36; b2+(13): 686.88; b2+(20): 987.04 |  | N12(Deamidated): 100; C27(Dehydro): 100; C33(Dehydro): 100 |  |  |  |  |  |  |  | 0.15 |  | 1 |  | Sequest HT (3) |  | 4 |  | 842.20148 |  | 1.00 |  | 51.0881 |  | 15774 |  | 15774 |  | TRINITY\_DN0\_c1\_g1\_i14.p1,TRINITY\_DN0\_c1\_g1\_i4.p1 |  | Image |  | Peak List |  |
|  | | | | | | | | | | | | | | | | | | | | | | | | | | | | | | | | | | | | | | | | | | | | | | | | | | | | | |  |
|  | High |  | [R].GLLDTFKNLALnAAKSAGVSVLNSLScKLSKTc.[-] |  | HCD |  | N12(Deamidated); C27(Dehydro); C33(Dehydro) |  | 6.36 |  | 1.3e-3 |  | 7.3e-5 |  | 1.76 |  | 311.73 |  | 0.500 |  | b1+(14): 1443.78; y1+(18): 1794.91; y1+(17): 1707.88; y1+(16): 1636.84; y1+(14): 1480.75; y1+(11): 1181.57; b2+(12): 651.36; b2+(13): 686.88; b2+(16): 829.96; b2+(17): 865.48 |  | N12(Deamidated): 50; N23(Deamidated): 50; C27(Dehydro): 100; C33(Dehydro): 100 |  |  |  |  |  |  |  | 0.09 |  | 1 |  | Sequest HT (3) |  | 3 |  | 1122.59766 |  | -0.69 |  | 51.1159 |  | 15786 |  | 15786 |  | TRINITY\_DN0\_c1\_g1\_i14.p1,TRINITY\_DN0\_c1\_g1\_i4.p1 |  | Image |  | Peak List |  |
|  | | | | | | | | | | | | | | | | | | | | | | | | | | | | | | | | | | | | | | | | | | | | | | | | | | | | | |  |
|  | High |  | [R].GLLDTFKNLALnAAKSAGVSVLNSLScKLSKTc.[-] |  | HCD |  | N12(Deamidated); C27(Dehydro); C33(Dehydro) |  | 4.49 |  | 1.3e-3 |  | 8.6e-3 |  | 0.723 |  | 190.17 |  | 0.500 |  | y1+(12): 1294.65; y1+(11): 1181.57; y2+(15): 790.41; y2+(12): 647.83; y2+(11): 591.29 |  | N12(Deamidated): 50; N23(Deamidated): 50; C27(Dehydro): 100; C33(Dehydro): 100 |  |  |  |  |  |  |  | 0.07 |  | 1 |  | Sequest HT (3) |  | 5 |  | 673.96124 |  | -1.07 |  | 51.1855 |  | 15814 |  | 15814 |  | TRINITY\_DN0\_c1\_g1\_i14.p1,TRINITY\_DN0\_c1\_g1\_i4.p1 |  | Image |  | Peak List |  |
|  | | | | | | | | | | | | | | | | | | | | | | | | | | | | | | | | | | | | | | | | | | | | | | | | | | | | | |  |
|  | High |  | [R].GLLDTFKNLALnAAKSAGVSVLNSLScKLSKTc.[-] |  | HCD |  | N12(Deamidated); C27(Dehydro); C33(Dehydro) |  | 6.47 |  | 1.3e-3 |  | 1.5e-2 |  | 0.597 |  | 296.61 |  | 1.000 |  | b1+(12): 1301.71; b1+(13): 1372.75; b1+(14): 1443.78; y1+(12): 1294.65; y1+(11): 1181.57; b2+(12): 651.36; b2+(13): 686.88; b2+(18): 893.99; b2+(20): 987.04 |  | N12(Deamidated): 100; C27(Dehydro): 100; C33(Dehydro): 100 |  |  |  |  |  |  |  | 0.18 |  | 1 |  | Sequest HT (3) |  | 4 |  | 842.20190 |  | 1.50 |  | 51.2298 |  | 15831 |  | 15831 |  | TRINITY\_DN0\_c1\_g1\_i14.p1,TRINITY\_DN0\_c1\_g1\_i4.p1 |  | Image |  | Peak List |  |
|  | | | | | | | | | | | | | | | | | | | | | | | | | | | | | | | | | | | | | | | | | | | | | | | | | | | | | |  |
|  | High |  | [R].GLLDTFKNLALnAAKSAGVSVLNSLScKLSKTc.[-] |  | HCD |  | N12(Deamidated); C27(Dehydro); C33(Dehydro) |  | 6.47 |  | 1.3e-3 |  | 1.2e-4 |  | 1.65 |  | 297.09 |  | 0.983 |  | b1+(14): 1443.78; y1+(19): 1923.00; y1+(18): 1794.91; y1+(17): 1707.88; y1+(16): 1636.84; y1+(14): 1480.75; y1+(11): 1181.57; b2+(12): 651.36; b2+(13): 686.88; b2+(16): 829.96 |  | N12(Deamidated): 98.32; C27(Dehydro): 100; C33(Dehydro): 100 |  |  |  |  |  |  |  | 0.13 |  | 1 |  | Sequest HT (3) |  | 3 |  | 1122.59753 |  | -0.79 |  | 51.2571 |  | 15841 |  | 15841 |  | TRINITY\_DN0\_c1\_g1\_i14.p1,TRINITY\_DN0\_c1\_g1\_i4.p1 |  | Image |  | Peak List |  |
|  | | | | | | | | | | | | | | | | | | | | | | | | | | | | | | | | | | | | | | | | | | | | | | | | | | | | | |  |
|  | High |  | [R].GLLDTFKNLALnAAKSAGVSVLNSLScKLSKTc.[-] |  | HCD |  | N12(Deamidated); C27(Dehydro); C33(Dehydro) |  | 4.34 |  | 1.3e-3 |  | 8.7e-3 |  | 0.722 |  | 209.59 |  | 0.494 |  | y1+(12): 1294.65; y1+(11): 1181.57; y2+(15): 790.41; y2+(14): 740.88; y2+(12): 647.83; y2+(11): 591.29 |  | N12(Deamidated): 49.42; N23(Deamidated): 49.42; C27(Dehydro): 100; C33(Dehydro): 100 |  |  |  |  |  |  |  | 0.10 |  | 1 |  | Sequest HT (3) |  | 5 |  | 673.96155 |  | -0.62 |  | 51.3290 |  | 15870 |  | 15870 |  | TRINITY\_DN0\_c1\_g1\_i14.p1,TRINITY\_DN0\_c1\_g1\_i4.p1 |  | Image |  | Peak List |  |
|  | | | | | | | | | | | | | | | | | | | | | | | | | | | | | | | | | | | | | | | | | | | | | | | | | | | | | |  |
|  | High |  | [R].GLLDTFKNLALnAAKSAGVSVLNSLScKLSKTc.[-] |  | HCD |  | N12(Deamidated); C27(Dehydro); C33(Dehydro) |  | 4.11 |  | 1.3e-3 |  | 8.1e-4 |  | 1.24 |  | 292.56 |  | 1.000 |  | b1+(8): 889.48; b1+(9): 1002.56; b1+(10): 1073.60 |  | N12(Deamidated): 99.98; C27(Dehydro): 100; C33(Dehydro): 100 |  |  |  |  |  |  |  | 0.09 |  | 1 |  | Sequest HT (3) |  | 2 |  | 1683.39490 |  | 0.53 |  | 51.3670 |  | 15885 |  | 15885 |  | TRINITY\_DN0\_c1\_g1\_i14.p1,TRINITY\_DN0\_c1\_g1\_i4.p1 |  | Image |  | Peak List |  |
|  | | | | | | | | | | | | | | | | | | | | | | | | | | | | | | | | | | | | | | | | | | | | | | | | | | | | | |  |
|  | High |  | [R].GLLDTFKNLALnAAKSAGVSVLNSLScKLSKTc.[-] |  | HCD |  | N12(Deamidated); C27(Dehydro); C33(Dehydro) |  | 6.82 |  | 1.3e-3 |  | 1.6e-3 |  | 1.08 |  | 300.62 |  | 1.000 |  | b1+(12): 1301.71; b1+(13): 1372.75; b1+(14): 1443.78; y1+(14): 1480.75; y1+(12): 1294.65; y1+(11): 1181.57; b2+(12): 651.36; b2+(13): 686.88; b2+(20): 987.04 |  | N12(Deamidated): 100; C27(Dehydro): 100; C33(Dehydro): 100 |  |  |  |  |  |  |  | 0.21 |  | 1 |  | Sequest HT (3) |  | 4 |  | 842.20221 |  | 1.87 |  | 51.3734 |  | 15887 |  | 15887 |  | TRINITY\_DN0\_c1\_g1\_i14.p1,TRINITY\_DN0\_c1\_g1\_i4.p1 |  | Image |  | Peak List |  |
|  | | | | | | | | | | | | | | | | | | | | | | | | | | | | | | | | | | | | | | | | | | | | | | | | | | | | | |  |
|  | High |  | [R].GLLDTFKNLALnAAKSAGVSVLNSLScKLSKTc.[-] |  | HCD |  | N12(Deamidated); C27(Dehydro); C33(Dehydro) |  | 6.47 |  | 1.3e-3 |  | 2.1e-5 |  | 2.04 |  | 336.04 |  | 0.999 |  | b1+(8): 889.48; b1+(9): 1002.56; b1+(11): 1186.68 |  | N12(Deamidated): 99.94; C27(Dehydro): 100; C33(Dehydro): 100 |  |  |  |  |  |  |  | 0.04 |  | 1 |  | Sequest HT (3) |  | 3 |  | 1122.59705 |  | -1.23 |  | 51.3933 |  | 15896 |  | 15896 |  | TRINITY\_DN0\_c1\_g1\_i14.p1,TRINITY\_DN0\_c1\_g1\_i4.p1 |  | Image |  | Peak List |  |
|  | | | | | | | | | | | | | | | | | | | | | | | | | | | | | | | | | | | | | | | | | | | | | | | | | | | | | |  |
|  | High |  | [R].GLLDTFKNLALNAAKSAGVSVLnSLScKLSKTc.[-] |  | HCD |  | N23(Deamidated); C27(Dehydro); C33(Dehydro) |  | 6.23 |  | 1.3e-3 |  | 1.7e-3 |  | 1.08 |  | 300.80 |  | 0.000 |  | b1+(8): 889.48; b1+(9): 1002.56; b1+(11): 1186.68 |  | N12(Deamidated): 99.94; C27(Dehydro): 100; C33(Dehydro): 100 |  |  |  |  |  |  |  |  |  | 2 |  | Sequest HT (3) |  | 3 |  | 1122.59705 |  | -1.23 |  | 51.3933 |  | 15896 |  | 15896 |  | TRINITY\_DN0\_c1\_g1\_i14.p1,TRINITY\_DN0\_c1\_g1\_i4.p1 |  | Image |  | Peak List |  |
|  | | | | | | | | | | | | | | | | | | | | | | | | | | | | | | | | | | | | | | | | | | | | | | | | | | | | | |  |
|  | High |  | [R].GLLDTFKNLALnAAKSAGVSVLNSLScKLSKTc.[-] |  | HCD |  | N12(Deamidated); C27(Dehydro); C33(Dehydro) |  | 5.03 |  | 1.3e-3 |  | 8.3e-3 |  | 0.73 |  | 196.62 |  | 0.972 |  | y1+(14): 1480.75; y1+(12): 1294.65; y1+(11): 1181.57; y2+(15): 790.41; y2+(14): 740.88; y2+(12): 647.83 |  | N12(Deamidated): 97.17; C27(Dehydro): 100; C33(Dehydro): 100 |  |  |  |  |  |  |  | 0.19 |  | 1 |  | Sequest HT (3) |  | 5 |  | 673.96112 |  | -1.26 |  | 51.4691 |  | 15927 |  | 15927 |  | TRINITY\_DN0\_c1\_g1\_i14.p1,TRINITY\_DN0\_c1\_g1\_i4.p1 |  | Image |  | Peak List |  |
|  | | | | | | | | | | | | | | | | | | | | | | | | | | | | | | | | | | | | | | | | | | | | | | | | | | | | | |  |
|  | High |  | [R].GLLDTFKNLALnAAKSAGVSVLNSLScKLSKTc.[-] |  | HCD |  | N12(Deamidated); C27(Dehydro); C33(Dehydro) |  | 6.74 |  | 1.3e-3 |  | 2.3e-2 |  | 0.512 |  | 277.60 |  | 1.000 |  | b1+(13): 1372.75; b1+(14): 1443.78; y1+(14): 1480.75; y1+(11): 1181.57; b2+(12): 651.36; b2+(20): 987.04; b2+(21): 1036.57 |  | N12(Deamidated): 99.96; C27(Dehydro): 100; C33(Dehydro): 100 |  |  |  |  |  |  |  | 0.13 |  | 1 |  | Sequest HT (3) |  | 4 |  | 842.20172 |  | 1.29 |  | 51.5142 |  | 15945 |  | 15945 |  | TRINITY\_DN0\_c1\_g1\_i14.p1,TRINITY\_DN0\_c1\_g1\_i4.p1 |  | Image |  | Peak List |  |
|  | | | | | | | | | | | | | | | | | | | | | | | | | | | | | | | | | | | | | | | | | | | | | | | | | | | | | |  |
|  | High |  | [R].GLLDTFKNLALnAAKSAGVSVLNSLScKLSKTc.[-] |  | HCD |  | N12(Deamidated); C27(Dehydro); C33(Dehydro) |  | 5.37 |  | 1.3e-3 |  | 1.6e-5 |  | 2.1 |  | 327.87 |  | 0.984 |  | b1+(14): 1443.78; y1+(18): 1794.91; y1+(17): 1707.88; y1+(16): 1636.84; y1+(14): 1480.75; y1+(11): 1181.57; b2+(12): 651.36; b2+(13): 686.88; b2+(16): 829.96 |  | N12(Deamidated): 98.41; C27(Dehydro): 100; C33(Dehydro): 100 |  |  |  |  |  |  |  | 0.03 |  | 1 |  | Sequest HT (3) |  | 3 |  | 1122.59741 |  | -0.90 |  | 51.5324 |  | 15952 |  | 15952 |  | TRINITY\_DN0\_c1\_g1\_i14.p1,TRINITY\_DN0\_c1\_g1\_i4.p1 |  | Image |  | Peak List |  |
|  | | | | | | | | | | | | | | | | | | | | | | | | | | | | | | | | | | | | | | | | | | | | | | | | | | | | | |  |
|  | High |  | [R].GLLDTFKNLALNAAKSAGVSVLnSLScKLSKTc.[-] |  | HCD |  | N23(Deamidated); C27(Dehydro); C33(Dehydro) |  | 5.20 |  | 1.3e-3 |  | 1.5e-3 |  | 1.1 |  | 309.96 |  | 0.016 |  | b1+(14): 1443.78; y1+(18): 1794.91; y1+(17): 1707.88; y1+(16): 1636.84; y1+(14): 1480.75; y1+(11): 1181.57; b2+(12): 651.36; b2+(13): 686.88; b2+(16): 829.96 |  | N12(Deamidated): 98.41; C27(Dehydro): 100; C33(Dehydro): 100 |  |  |  |  |  |  |  |  |  | 2 |  | Sequest HT (3) |  | 3 |  | 1122.59741 |  | -0.90 |  | 51.5324 |  | 15952 |  | 15952 |  | TRINITY\_DN0\_c1\_g1\_i14.p1,TRINITY\_DN0\_c1\_g1\_i4.p1 |  | Image |  | Peak List |  |
|  | | | | | | | | | | | | | | | | | | | | | | | | | | | | | | | | | | | | | | | | | | | | | | | | | | | | | |  |
|  | High |  | [R].GLLDTFKNLALnAAKSAGVSVLNSLScKLSKTc.[-] |  | HCD |  | N12(Deamidated); C27(Dehydro); C33(Dehydro) |  | 3.58 |  | 1.3e-3 |  | 5.3e-2 |  | 0.327 |  | 135.51 |  | 0.000 |  | y1+(14): 1481.73; y1+(12): 1295.63; y1+(11): 1182.55; y2+(18): 898.45; y2+(12): 648.32 |  | N23(Deamidated): 100; C27(Dehydro): 100; C33(Dehydro): 100 |  |  |  |  |  |  |  | 0.07 |  | 1 |  | Sequest HT (3) |  | 5 |  | 673.96112 |  | -1.26 |  | 51.6133 |  | 15985 |  | 15985 |  | TRINITY\_DN0\_c1\_g1\_i14.p1,TRINITY\_DN0\_c1\_g1\_i4.p1 |  | Image |  | Peak List |  |
|  | | | | | | | | | | | | | | | | | | | | | | | | | | | | | | | | | | | | | | | | | | | | | | | | | | | | | |  |
|  | High |  | [R].GLLDTFKNLALnAAKSAGVSVLNSLScKLSKTc.[-] |  | HCD |  | N12(Deamidated); C27(Dehydro); C33(Dehydro) |  | 6.92 |  | 1.3e-3 |  | 4.6e-3 |  | 0.858 |  | 233.19 |  | 0.000 |  | b1+(13): 1371.76; b1+(14): 1442.80; y1+(14): 1481.73; y1+(13): 1394.70; y1+(12): 1295.63; y1+(11): 1182.55; y2+(18): 898.45; y2+(12): 648.32 |  | N23(Deamidated): 100; C27(Dehydro): 100; C33(Dehydro): 100 |  |  |  |  |  |  |  | 0.14 |  | 1 |  | Sequest HT (3) |  | 4 |  | 842.20154 |  | 1.07 |  | 51.6498 |  | 16000 |  | 16000 |  | TRINITY\_DN0\_c1\_g1\_i14.p1,TRINITY\_DN0\_c1\_g1\_i4.p1 |  | Image |  | Peak List |  |
|  | | | | | | | | | | | | | | | | | | | | | | | | | | | | | | | | | | | | | | | | | | | | | | | | | | | | | |  |
|  | High |  | [R].GLLDTFKNLALnAAKSAGVSVLNSLScKLSKTc.[-] |  | HCD |  | N12(Deamidated); C27(Dehydro); C33(Dehydro) |  | 5.46 |  | 1.3e-3 |  | 6.7e-5 |  | 1.78 |  | 263.80 |  | 0.000 |  | y1+(19): 1923.99; y1+(18): 1795.89; y1+(17): 1708.86; y1+(16): 1637.82; y1+(15): 1580.80; y1+(14): 1481.73; y1+(13): 1394.70; y1+(12): 1295.63; y2+(21): 1033.53; y2+(18): 898.45; y2+(12): 648.32 |  | N23(Deamidated): 100; C27(Dehydro): 100; C33(Dehydro): 100 |  |  |  |  |  |  |  | 0.00 |  | 1 |  | Sequest HT (3) |  | 3 |  | 1122.59802 |  | -0.36 |  | 51.6694 |  | 16008 |  | 16008 |  | TRINITY\_DN0\_c1\_g1\_i14.p1,TRINITY\_DN0\_c1\_g1\_i4.p1 |  | Image |  | Peak List |  |
|  | | | | | | | | | | | | | | | | | | | | | | | | | | | | | | | | | | | | | | | | | | | | | | | | | | | | | |  |
|  | High |  | [R].GLLDTFKNLALNAAKSAGVSVLnSLScKLSKTc.[-] |  | HCD |  | N23(Deamidated); C27(Dehydro); C33(Dehydro) |  | 5.44 |  | 1.3e-3 |  | 3.8e-4 |  | 1.4 |  | 315.25 |  | 1.000 |  | y1+(19): 1923.99; y1+(18): 1795.89; y1+(17): 1708.86; y1+(16): 1637.82; y1+(15): 1580.80; y1+(14): 1481.73; y1+(13): 1394.70; y1+(12): 1295.63; y2+(21): 1033.53; y2+(18): 898.45; y2+(12): 648.32 |  | N23(Deamidated): 100; C27(Dehydro): 100; C33(Dehydro): 100 |  |  |  |  |  |  |  |  |  | 2 |  | Sequest HT (3) |  | 3 |  | 1122.59802 |  | -0.36 |  | 51.6694 |  | 16008 |  | 16008 |  | TRINITY\_DN0\_c1\_g1\_i14.p1,TRINITY\_DN0\_c1\_g1\_i4.p1 |  | Image |  | Peak List |  |
|  | | | | | | | | | | | | | | | | | | | | | | | | | | | | | | | | | | | | | | | | | | | | | | | | | | | | | |  |
|  | High |  | [R].GLLDTFKNLALNAAKSAGVSVLnSLScKLSKTc.[-] |  | HCD |  | N23(Deamidated); C27(Dehydro); C33(Dehydro) |  | 4.10 |  | 1.3e-3 |  | 3.0e-3 |  | 0.951 |  | 237.13 |  | 0.000 |  | b1+(12): 1301.71; b1+(13): 1372.75; b1+(14): 1443.78; b1+(15): 1571.88; b1+(19): 1886.04; y1+(16): 1636.84; y1+(12): 1294.65; y1+(11): 1181.57 |  | N12(Deamidated): 99.98; C27(Dehydro): 100; C33(Dehydro): 100 |  |  |  |  |  |  |  | 0.11 |  | 1 |  | Sequest HT (3) |  | 2 |  | 1683.39819 |  | 2.49 |  | 51.6736 |  | 16010 |  | 16010 |  | TRINITY\_DN0\_c1\_g1\_i14.p1,TRINITY\_DN0\_c1\_g1\_i4.p1 |  | Image |  | Peak List |  |
|  | | | | | | | | | | | | | | | | | | | | | | | | | | | | | | | | | | | | | | | | | | | | | | | | | | | | | |  |
|  | High |  | [R].GLLDTFKNLALNAAKSAGVSVLnSLScKLSKTc.[-] |  | HCD |  | N23(Deamidated); C27(Dehydro); C33(Dehydro) |  | 3.08 |  | 1.3e-3 |  | 2.2e-2 |  | 0.514 |  | 175.10 |  | 1.000 |  | y1+(12): 1295.63; y1+(11): 1182.55; y2+(19): 962.50; y2+(18): 898.45; y2+(14): 741.37; y2+(12): 648.32 |  | N23(Deamidated): 100; C27(Dehydro): 100; C33(Dehydro): 100 |  |  |  |  |  |  |  | 0.02 |  | 1 |  | Sequest HT (3) |  | 5 |  | 673.96161 |  | -0.53 |  | 51.7560 |  | 16042 |  | 16042 |  | TRINITY\_DN0\_c1\_g1\_i14.p1,TRINITY\_DN0\_c1\_g1\_i4.p1 |  | Image |  | Peak List |  |
|  | | | | | | | | | | | | | | | | | | | | | | | | | | | | | | | | | | | | | | | | | | | | | | | | | | | | | |  |
|  | High |  | [R].GLLDTFKNLALnAAKSAGVSVLNSLScKLSKTc.[-] |  | HCD |  | N12(Deamidated); C27(Dehydro); C33(Dehydro) |  | 3.03 |  | 2.4e-3 |  | 8.1e-2 |  | 0.229 |  | 117.92 |  | 0.000 |  | y1+(12): 1295.63; y1+(11): 1182.55; y2+(19): 962.50; y2+(18): 898.45; y2+(14): 741.37; y2+(12): 648.32 |  | N23(Deamidated): 100; C27(Dehydro): 100; C33(Dehydro): 100 |  |  |  |  |  |  |  |  |  | 2 |  | Sequest HT (3) |  | 5 |  | 673.96161 |  | -0.53 |  | 51.7560 |  | 16042 |  | 16042 |  | TRINITY\_DN0\_c1\_g1\_i14.p1,TRINITY\_DN0\_c1\_g1\_i4.p1 |  | Image |  | Peak List |  |
|  | | | | | | | | | | | | | | | | | | | | | | | | | | | | | | | | | | | | | | | | | | | | | | | | | | | | | |  |
|  | High |  | [R].GLLDTFKNLALnAAKSAGVSVLNSLScKLSKTc.[-] |  | HCD |  | N12(Deamidated); C27(Dehydro); C33(Dehydro) |  | 6.04 |  | 1.3e-3 |  | 1.1e-2 |  | 0.676 |  | 252.11 |  | 0.023 |  | y1+(16): 1637.82; y1+(14): 1481.73; y1+(12): 1295.63; y1+(11): 1182.55; y2+(19): 962.50; y2+(18): 898.45; y2+(12): 648.32; y2+(11): 591.78 |  | N23(Deamidated): 97.73; C27(Dehydro): 100; C33(Dehydro): 100 |  |  |  |  |  |  |  | 0.10 |  | 1 |  | Sequest HT (3) |  | 4 |  | 842.20178 |  | 1.36 |  | 51.7899 |  | 16058 |  | 16058 |  | TRINITY\_DN0\_c1\_g1\_i14.p1,TRINITY\_DN0\_c1\_g1\_i4.p1 |  | Image |  | Peak List |  |
|  | | | | | | | | | | | | | | | | | | | | | | | | | | | | | | | | | | | | | | | | | | | | | | | | | | | | | |  |
|  | High |  | [R].GLLDTFKNLALNAAKSAGVSVLnSLScKLSKTc.[-] |  | HCD |  | N23(Deamidated); C27(Dehydro); C33(Dehydro) |  | 6.36 |  | 1.3e-3 |  | 1.2e-3 |  | 1.16 |  | 293.83 |  | 0.980 |  | y1+(18): 1795.89; y1+(17): 1708.86; y1+(16): 1637.82; y1+(15): 1580.80; y1+(14): 1481.73; y1+(12): 1295.63; b2+(13): 686.39; y2+(21): 1033.53; y2+(20): 998.02; y2+(19): 962.50; y2+(12): 648.32 |  | N23(Deamidated): 97.99; C27(Dehydro): 100; C33(Dehydro): 100 |  |  |  |  |  |  |  | 0.14 |  | 1 |  | Sequest HT (3) |  | 3 |  | 1122.59705 |  | -1.23 |  | 51.8126 |  | 16067 |  | 16067 |  | TRINITY\_DN0\_c1\_g1\_i14.p1,TRINITY\_DN0\_c1\_g1\_i4.p1 |  | Image |  | Peak List |  |
|  | | | | | | | | | | | | | | | | | | | | | | | | | | | | | | | | | | | | | | | | | | | | | | | | | | | | | |  |
|  | High |  | [R].GLLDTFKNLALnAAKSAGVSVLNSLScKLSKTc.[-] |  | HCD |  | N12(Deamidated); C27(Dehydro); C33(Dehydro) |  | 3.41 |  | 1.3e-3 |  | 1.6e-2 |  | 0.594 |  | 145.40 |  | 0.492 |  | b1+(10): 1074.58 |  | N8(Deamidated): 49.2; N12(Deamidated): 49.2; C27(Dehydro): 100; C33(Dehydro): 100 |  |  |  |  |  |  |  | 0.08 |  | 1 |  | Sequest HT (3) |  | 5 |  | 673.96161 |  | -0.53 |  | 51.9005 |  | 16100 |  | 16100 |  | TRINITY\_DN0\_c1\_g1\_i14.p1,TRINITY\_DN0\_c1\_g1\_i4.p1 |  | Image |  | Peak List |  |
|  | | | | | | | | | | | | | | | | | | | | | | | | | | | | | | | | | | | | | | | | | | | | | | | | | | | | | |  |
|  | High |  | [R].GLLDTFKnLALNAAKSAGVSVLNSLScKLSKTc.[-] |  | HCD |  | N8(Deamidated); C27(Dehydro); C33(Dehydro) |  | 6.62 |  | 1.3e-3 |  | 2.1e-3 |  | 1.03 |  | 293.09 |  | 1.000 |  | b1+(8): 890.46; b1+(9): 1003.55; b1+(10): 1074.58; b1+(11): 1187.67 |  | N8(Deamidated): 100; C27(Dehydro): 100; C33(Dehydro): 100 |  |  |  |  |  |  |  | 0.10 |  | 1 |  | Sequest HT (3) |  | 4 |  | 842.20099 |  | 0.42 |  | 51.9352 |  | 16118 |  | 16118 |  | TRINITY\_DN0\_c1\_g1\_i14.p1,TRINITY\_DN0\_c1\_g1\_i4.p1 |  | Image |  | Peak List |  |
|  | | | | | | | | | | | | | | | | | | | | | | | | | | | | | | | | | | | | | | | | | | | | | | | | | | | | | |  |
|  | High |  | [R].GLLDTFKNLALnAAKSAGVSVLNSLScKLSKTc.[-] |  | HCD |  | N12(Deamidated); C27(Dehydro); C33(Dehydro) |  | 6.54 |  | 1.3e-3 |  | 1.6e-5 |  | 2.1 |  | 330.37 |  | 0.000 |  | b1+(8): 890.46; b1+(9): 1003.55; b1+(10): 1074.58; b1+(11): 1187.67 |  | N8(Deamidated): 100; C27(Dehydro): 100; C33(Dehydro): 100 |  |  |  |  |  |  |  | 0.01 |  | 1 |  | Sequest HT (3) |  | 3 |  | 1122.59790 |  | -0.47 |  | 51.9522 |  | 16124 |  | 16124 |  | TRINITY\_DN0\_c1\_g1\_i14.p1,TRINITY\_DN0\_c1\_g1\_i4.p1 |  | Image |  | Peak List |  |
|  | | | | | | | | | | | | | | | | | | | | | | | | | | | | | | | | | | | | | | | | | | | | | | | | | | | | | |  |
|  | High |  | [R].GLLDTFKnLALNAAKSAGVSVLNSLScKLSKTc.[-] |  | HCD |  | N8(Deamidated); C27(Dehydro); C33(Dehydro) |  | 6.50 |  | 1.3e-3 |  | 1.5e-5 |  | 2.1 |  | 402.41 |  | 1.000 |  | b1+(8): 890.46; b1+(9): 1003.55; b1+(10): 1074.58; b1+(11): 1187.67 |  | N8(Deamidated): 100; C27(Dehydro): 100; C33(Dehydro): 100 |  |  |  |  |  |  |  |  |  | 2 |  | Sequest HT (3) |  | 3 |  | 1122.59790 |  | -0.47 |  | 51.9522 |  | 16124 |  | 16124 |  | TRINITY\_DN0\_c1\_g1\_i14.p1,TRINITY\_DN0\_c1\_g1\_i4.p1 |  | Image |  | Peak List |  |
|  | | | | | | | | | | | | | | | | | | | | | | | | | | | | | | | | | | | | | | | | | | | | | | | | | | | | | |  |
|  | High |  | [R].GLLDTFKnLALNAAKSAGVSVLNSLScKLSKTc.[-] |  | HCD |  | N8(Deamidated); C27(Dehydro); C33(Dehydro) |  | 4.11 |  | 1.3e-3 |  | 1.1e-3 |  | 1.18 |  | 264.01 |  | 0.500 |  | b1+(8): 890.46; b1+(9): 1003.55; b1+(10): 1074.58; b1+(11): 1187.67 |  | N8(Deamidated): 50; N12(Deamidated): 50; C27(Dehydro): 100; C33(Dehydro): 100 |  |  |  |  |  |  |  | 0.16 |  | 1 |  | Sequest HT (3) |  | 2 |  | 1683.38977 |  | -2.51 |  | 51.9847 |  | 16138 |  | 16138 |  | TRINITY\_DN0\_c1\_g1\_i14.p1,TRINITY\_DN0\_c1\_g1\_i4.p1 |  | Image |  | Peak List |  |
|  | | | | | | | | | | | | | | | | | | | | | | | | | | | | | | | | | | | | | | | | | | | | | | | | | | | | | |  |
|  | High |  | [R].GLLDTFKnLALNAAKSAGVSVLNSLScKLSKTc.[-] |  | HCD |  | N8(Deamidated); C27(Dehydro); C33(Dehydro) |  | 3.56 |  | 2.4e-3 |  | 7.5e-2 |  | 0.247 |  | 143.86 |  | 0.999 |  | b1+(9): 1003.55; b1+(10): 1074.58 |  | N8(Deamidated): 99.88; C27(Dehydro): 100; C33(Dehydro): 100 |  |  |  |  |  |  |  | 0.09 |  | 1 |  | Sequest HT (3) |  | 5 |  | 673.96191 |  | -0.08 |  | 52.0496 |  | 16167 |  | 16167 |  | TRINITY\_DN0\_c1\_g1\_i14.p1,TRINITY\_DN0\_c1\_g1\_i4.p1 |  | Image |  | Peak List |  |
|  | | | | | | | | | | | | | | | | | | | | | | | | | | | | | | | | | | | | | | | | | | | | | | | | | | | | | |  |
|  | High |  | [R].GLLDTFKnLALNAAKSAGVSVLNSLScKLSKTc.[-] |  | HCD |  | N8(Deamidated); C27(Dehydro); C33(Dehydro) |  | 7.15 |  | 1.3e-3 |  | 4.1e-3 |  | 0.883 |  | 297.18 |  | 1.000 |  | b1+(8): 890.46; b1+(9): 1003.55; b1+(10): 1074.58; b1+(11): 1187.67; b2+(10): 537.80 |  | N8(Deamidated): 100; C27(Dehydro): 100; C33(Dehydro): 100 |  |  |  |  |  |  |  | 0.14 |  | 1 |  | Sequest HT (3) |  | 4 |  | 842.20117 |  | 0.63 |  | 52.0767 |  | 16178 |  | 16178 |  | TRINITY\_DN0\_c1\_g1\_i14.p1,TRINITY\_DN0\_c1\_g1\_i4.p1 |  | Image |  | Peak List |  |
|  | | | | | | | | | | | | | | | | | | | | | | | | | | | | | | | | | | | | | | | | | | | | | | | | | | | | | |  |
|  | High |  | [R].GLLDTFKnLALNAAKSAGVSVLNSLScKLSKTc.[-] |  | HCD |  | N8(Deamidated); C27(Dehydro); C33(Dehydro) |  | 6.18 |  | 1.3e-3 |  | 3.4e-5 |  | 1.93 |  | 371.13 |  | 1.000 |  | b1+(8): 890.46; b1+(9): 1003.55; b1+(10): 1074.58; b1+(11): 1187.67; b2+(9): 502.28 |  | N8(Deamidated): 100; C27(Dehydro): 100; C33(Dehydro): 100 |  |  |  |  |  |  |  | 0.11 |  | 1 |  | Sequest HT (3) |  | 3 |  | 1122.59717 |  | -1.12 |  | 52.0923 |  | 16183 |  | 16183 |  | TRINITY\_DN0\_c1\_g1\_i14.p1,TRINITY\_DN0\_c1\_g1\_i4.p1 |  | Image |  | Peak List |  |
|  | | | | | | | | | | | | | | | | | | | | | | | | | | | | | | | | | | | | | | | | | | | | | | | | | | | | | |  |
|  | High |  | [R].GLLDTFKnLALNAAKSAGVSVLNSLScKLSKTc.[-] |  | HCD |  | N8(Deamidated); C27(Dehydro); C33(Dehydro) |  | 4.11 |  | 1.3e-3 |  | 2.1e-2 |  | 0.524 |  | 155.46 |  | 0.999 |  | b1+(8): 890.46; b1+(10): 1074.58 |  | N8(Deamidated): 99.92; C27(Dehydro): 100; C33(Dehydro): 100 |  |  |  |  |  |  |  | 0.13 |  | 1 |  | Sequest HT (3) |  | 5 |  | 673.96228 |  | 0.47 |  | 52.1983 |  | 16229 |  | 16229 |  | TRINITY\_DN0\_c1\_g1\_i14.p1,TRINITY\_DN0\_c1\_g1\_i4.p1 |  | Image |  | Peak List |  |
|  | | | | | | | | | | | | | | | | | | | | | | | | | | | | | | | | | | | | | | | | | | | | | | | | | | | | | |  |
|  | High |  | [R].GLLDTFKnLALNAAKSAGVSVLNSLScKLSKTc.[-] |  | HCD |  | N8(Deamidated); C27(Dehydro); C33(Dehydro) |  | 7.03 |  | 1.3e-3 |  | 2.6e-3 |  | 0.989 |  | 320.19 |  | 1.000 |  | b1+(8): 890.46; b1+(9): 1003.55; b1+(10): 1074.58; b1+(11): 1187.67; b2+(10): 537.80 |  | N8(Deamidated): 100; C27(Dehydro): 100; C33(Dehydro): 100 |  |  |  |  |  |  |  | 0.12 |  | 1 |  | Sequest HT (3) |  | 4 |  | 842.20203 |  | 1.65 |  | 52.2146 |  | 16235 |  | 16235 |  | TRINITY\_DN0\_c1\_g1\_i14.p1,TRINITY\_DN0\_c1\_g1\_i4.p1 |  | Image |  | Peak List |  |
|  | | | | | | | | | | | | | | | | | | | | | | | | | | | | | | | | | | | | | | | | | | | | | | | | | | | | | |  |
|  | High |  | [R].GLLDTFKnLALNAAKSAGVSVLNSLScKLSKTc.[-] |  | HCD |  | N8(Deamidated); C27(Dehydro); C33(Dehydro) |  | 6.72 |  | 1.3e-3 |  | 1.0e-5 |  | 2.19 |  | 399.53 |  | 1.000 |  | b1+(8): 890.46; b1+(9): 1003.55; b1+(10): 1074.58; b1+(11): 1187.67 |  | N8(Deamidated): 100; C27(Dehydro): 100; C33(Dehydro): 100 |  |  |  |  |  |  |  | 0.12 |  | 1 |  | Sequest HT (3) |  | 3 |  | 1122.59680 |  | -1.45 |  | 52.2335 |  | 16241 |  | 16241 |  | TRINITY\_DN0\_c1\_g1\_i14.p1,TRINITY\_DN0\_c1\_g1\_i4.p1 |  | Image |  | Peak List |  |
|  | | | | | | | | | | | | | | | | | | | | | | | | | | | | | | | | | | | | | | | | | | | | | | | | | | | | | |  |
|  | High |  | [R].GLLDTFKnLALNAAKSAGVSVLNSLScKLSKTc.[-] |  | HCD |  | N8(Deamidated); C27(Dehydro); C33(Dehydro) |  | 3.87 |  | 1.3e-3 |  | 4.6e-3 |  | 0.862 |  | 232.68 |  | 1.000 |  | b1+(8): 890.46; b1+(9): 1003.55; b1+(10): 1074.58 |  | N8(Deamidated): 100; C27(Dehydro): 100; C33(Dehydro): 100 |  |  |  |  |  |  |  | 0.16 |  | 1 |  | Sequest HT (3) |  | 5 |  | 673.96234 |  | 0.56 |  | 52.3427 |  | 16284 |  | 16284 |  | TRINITY\_DN0\_c1\_g1\_i14.p1,TRINITY\_DN0\_c1\_g1\_i4.p1 |  | Image |  | Peak List |  |
|  | | | | | | | | | | | | | | | | | | | | | | | | | | | | | | | | | | | | | | | | | | | | | | | | | | | | | |  |
|  | High |  | [R].GLLDTFKnLALNAAKSAGVSVLNSLScKLSKTc.[-] |  | HCD |  | N8(Deamidated); C27(Dehydro); C33(Dehydro) |  | 6.92 |  | 1.3e-3 |  | 1.1e-5 |  | 2.18 |  | 401.65 |  | 1.000 |  | b1+(8): 890.46; b1+(9): 1003.55; b1+(10): 1074.58; b1+(11): 1187.67; b2+(11): 594.34 |  | N8(Deamidated): 100; C27(Dehydro): 100; C33(Dehydro): 100 |  |  |  |  |  |  |  | 0.10 |  | 1 |  | Sequest HT (3) |  | 3 |  | 1122.59753 |  | -0.79 |  | 52.3766 |  | 16295 |  | 16295 |  | TRINITY\_DN0\_c1\_g1\_i14.p1,TRINITY\_DN0\_c1\_g1\_i4.p1 |  | Image |  | Peak List |  |
|  | | | | | | | | | | | | | | | | | | | | | | | | | | | | | | | | | | | | | | | | | | | | | | | | | | | | | |  |
|  | High |  | [R].GLLDTFKnLALNAAKSAGVSVLNSLScKLSKTc.[-] |  | HCD |  | N8(Deamidated); C27(Dehydro); C33(Dehydro) |  | 3.42 |  | 1.3e-3 |  | 6.5e-2 |  | 0.278 |  | 123.81 |  | 0.998 |  | b1+(8): 890.46; b1+(10): 1074.58 |  | N8(Deamidated): 99.81; C27(Dehydro): 100; C33(Dehydro): 100 |  |  |  |  |  |  |  | 0.13 |  | 1 |  | Sequest HT (3) |  | 5 |  | 673.96191 |  | -0.08 |  | 52.4874 |  | 16337 |  | 16337 |  | TRINITY\_DN0\_c1\_g1\_i14.p1,TRINITY\_DN0\_c1\_g1\_i4.p1 |  | Image |  | Peak List |  |
|  | | | | | | | | | | | | | | | | | | | | | | | | | | | | | | | | | | | | | | | | | | | | | | | | | | | | | |  |
|  | High |  | [R].GLLDTFKnLALNAAKSAGVSVLNSLScKLSKTc.[-] |  | HCD |  | N8(Deamidated); C27(Dehydro); C33(Dehydro) |  | 6.41 |  | 1.3e-3 |  | 2.3e-3 |  | 1.01 |  | 328.47 |  | 1.000 |  | b1+(8): 890.46; b1+(9): 1003.55; b1+(10): 1074.58; b1+(11): 1187.67; b2+(10): 537.80 |  | N8(Deamidated): 100; C27(Dehydro): 100; C33(Dehydro): 100 |  |  |  |  |  |  |  | 0.11 |  | 1 |  | Sequest HT (3) |  | 4 |  | 842.20233 |  | 2.01 |  | 52.4966 |  | 16341 |  | 16341 |  | TRINITY\_DN0\_c1\_g1\_i14.p1,TRINITY\_DN0\_c1\_g1\_i4.p1 |  | Image |  | Peak List |  |
|  | | | | | | | | | | | | | | | | | | | | | | | | | | | | | | | | | | | | | | | | | | | | | | | | | | | | | |  |
|  | High |  | [R].GLLDTFKnLALNAAKSAGVSVLNSLScKLSKTc.[-] |  | HCD |  | N8(Deamidated); C27(Dehydro); C33(Dehydro) |  | 4.09 |  | 1.3e-3 |  | 1.6e-2 |  | 0.593 |  | 249.28 |  | 1.000 |  | b1+(8): 890.46; b1+(9): 1003.55; b1+(11): 1187.67; b2+(10): 537.80 |  | N8(Deamidated): 100; C27(Dehydro): 100; C33(Dehydro): 100 |  |  |  |  |  |  |  | 0.09 |  | 1 |  | Sequest HT (3) |  | 3 |  | 1122.59839 |  | -0.03 |  | 52.5161 |  | 16349 |  | 16349 |  | TRINITY\_DN0\_c1\_g1\_i14.p1,TRINITY\_DN0\_c1\_g1\_i4.p1 |  | Image |  | Peak List |  |
|  | | | | | | | | | | | | | | | | | | | | | | | | | | | | | | | | | | | | | | | | | | | | | | | | | | | | | |  |
|  | High |  | [R].GLLDTFKnLALNAAKSAGVSVLNSLScKLSKTc.[-] |  | HCD |  | N8(Deamidated); C27(Dehydro); C33(Dehydro) |  | 5.78 |  | 1.3e-3 |  | 6.4e-3 |  | 0.788 |  | 301.88 |  | 1.000 |  | b1+(8): 890.46; b1+(9): 1003.55; b1+(10): 1074.58; b1+(11): 1187.67 |  | N8(Deamidated): 100; C27(Dehydro): 100; C33(Dehydro): 100 |  |  |  |  |  |  |  | 0.18 |  | 1 |  | Sequest HT (3) |  | 4 |  | 842.20178 |  | 1.36 |  | 52.6374 |  | 16391 |  | 16391 |  | TRINITY\_DN0\_c1\_g1\_i14.p1,TRINITY\_DN0\_c1\_g1\_i4.p1 |  | Image |  | Peak List |  |
|  | | | | | | | | | | | | | | | | | | | | | | | | | | | | | | | | | | | | | | | | | | | | | | | | | | | | | |  |
|  | High |  | [R].GLLDTFKnLALNAAKSAGVSVLNSLScKLSKTc.[-] |  | HCD |  | N8(Deamidated); C27(Dehydro); C33(Dehydro) |  | 3.58 |  | 1.3e-3 |  | 3.6e-2 |  | 0.408 |  | 164.89 |  | 0.999 |  | b1+(8): 890.46; b1+(10): 1074.58 |  | N8(Deamidated): 99.92; C27(Dehydro): 100; C33(Dehydro): 100 |  |  |  |  |  |  |  | 0.18 |  | 1 |  | Sequest HT (3) |  | 5 |  | 673.96210 |  | 0.19 |  | 52.6399 |  | 16392 |  | 16392 |  | TRINITY\_DN0\_c1\_g1\_i14.p1,TRINITY\_DN0\_c1\_g1\_i4.p1 |  | Image |  | Peak List |  |
|  | | | | | | | | | | | | | | | | | | | | | | | | | | | | | | | | | | | | | | | | | | | | | | | | | | | | | |  |
|  | High |  | [R].GLLDTFKnLALNAAKSAGVSVLNSLScKLSKTc.[-] |  | HCD |  | N8(Deamidated); C27(Dehydro); C33(Dehydro) |  | 6.13 |  | 1.3e-3 |  | 2.9e-5 |  | 1.96 |  | 417.63 |  | 1.000 |  | b1+(8): 890.46; b1+(9): 1003.55; b1+(10): 1074.58; b1+(11): 1187.67; y2+(25): 1238.67; y2+(24): 1182.12 |  | N8(Deamidated): 100; C27(Dehydro): 100; C33(Dehydro): 100 |  |  |  |  |  |  |  | 0.14 |  | 1 |  | Sequest HT (3) |  | 3 |  | 1122.59778 |  | -0.58 |  | 52.6565 |  | 16398 |  | 16398 |  | TRINITY\_DN0\_c1\_g1\_i14.p1,TRINITY\_DN0\_c1\_g1\_i4.p1 |  | Image |  | Peak List |  |
|  | | | | | | | | | | | | | | | | | | | | | | | | | | | | | | | | | | | | | | | | | | | | | | | | | | | | | |  |
|  | High |  | [R].GLLDTFKnLALnAAKSAGVSVLNSLScKLSKTc.[-] |  | HCD |  | N8(Deamidated); N12(Deamidated); C27(Dehydro); C33(Dehydro) |  | 7.13 |  | 1.3e-3 |  | 3.6e-6 |  | 2.42 |  | 322.69 |  | 0.500 |  | b1+(12): 1302.69; b1+(13): 1373.73; b1+(14): 1444.77; y1+(19): 1923.00; y1+(17): 1707.88; y1+(16): 1636.84; y1+(14): 1480.75; y1+(13): 1393.72; y1+(12): 1294.65; y1+(11): 1181.57; b2+(16): 830.45 |  | N8(Deamidated): 100; N12(Deamidated): 50; N23(Deamidated): 50; C27(Dehydro): 100; C33(Dehydro): 100 |  |  |  |  |  |  |  | 0.04 |  | 1 |  | Sequest HT (3) |  | 3 |  | 1122.92871 |  | 2.03 |  | 52.8439 |  | 16464 |  | 16464 |  | TRINITY\_DN0\_c1\_g1\_i14.p1,TRINITY\_DN0\_c1\_g1\_i4.p1 |  | Image |  | Peak List |  |
|  | | | | | | | | | | | | | | | | | | | | | | | | | | | | | | | | | | | | | | | | | | | | | | | | | | | | | |  |
|  | High |  | [R].GLLDTFKNLALnAAKSAGVSVLnSLScKLSKTc.[-] |  | HCD |  | N12(Deamidated); N23(Deamidated); C27(Dehydro); C33(Dehydro) |  | 6.85 |  | 1.3e-3 |  | 1.5e-4 |  | 1.6 |  | 270.29 |  | 0.000 |  | b1+(12): 1302.69; b1+(13): 1373.73; b1+(14): 1444.77; y1+(19): 1923.00; y1+(17): 1707.88; y1+(16): 1636.84; y1+(14): 1480.75; y1+(13): 1393.72; y1+(12): 1294.65; y1+(11): 1181.57; b2+(16): 830.45 |  | N8(Deamidated): 100; N12(Deamidated): 50; N23(Deamidated): 50; C27(Dehydro): 100; C33(Dehydro): 100 |  |  |  |  |  |  |  |  |  | 2 |  | Sequest HT (3) |  | 3 |  | 1122.92871 |  | 2.03 |  | 52.8439 |  | 16464 |  | 16464 |  | TRINITY\_DN0\_c1\_g1\_i14.p1,TRINITY\_DN0\_c1\_g1\_i4.p1 |  | Image |  | Peak List |  |
|  | | | | | | | | | | | | | | | | | | | | | | | | | | | | | | | | | | | | | | | | | | | | | | | | | | | | | |  |
|  | High |  | [R].GLLDTFKnLALnAAKSAGVSVLNSLScKLSKTc.[-] |  | HCD |  | N8(Deamidated); N12(Deamidated); C27(Dehydro); C33(Dehydro) |  | 6.37 |  | 1.3e-3 |  | 5.9e-4 |  | 1.31 |  | 304.44 |  | 1.000 |  | b1+(12): 1302.69; b1+(13): 1373.73; b1+(14): 1444.77; y1+(18): 1794.91; y1+(12): 1294.65; y1+(11): 1181.57; b2+(12): 651.85; b2+(13): 687.37; b2+(20): 987.53 |  | N8(Deamidated): 100; N12(Deamidated): 100; C27(Dehydro): 100; C33(Dehydro): 100 |  |  |  |  |  |  |  | 0.15 |  | 1 |  | Sequest HT (3) |  | 4 |  | 842.44781 |  | 1.39 |  | 52.8577 |  | 16470 |  | 16470 |  | TRINITY\_DN0\_c1\_g1\_i14.p1,TRINITY\_DN0\_c1\_g1\_i4.p1 |  | Image |  | Peak List |  |
|  | | | | | | | | | | | | | | | | | | | | | | | | | | | | | | | | | | | | | | | | | | | | | | | | | | | | | |  |
|  | High |  | [R].GLLDTFKnLALnAAKSAGVSVLNSLScKLSKTc.[-] |  | HCD |  | N8(Deamidated); N12(Deamidated); C27(Dehydro); C33(Dehydro) |  | 5.60 |  | 1.3e-3 |  | 1.1e-6 |  | 2.67 |  | 501.78 |  | 1.000 |  | b1+(12): 1302.69; b1+(13): 1373.73; b1+(14): 1444.77; b1+(15): 1572.86; b1+(19): 1887.02; b1+(21): 2073.12; b1+(22): 2186.21; y1+(21): 2065.08; y1+(20): 1994.04; y1+(19): 1923.00; y1+(18): 1794.91; y1+(16): 1636.84; y1+(12): 1294.65 |  | N8(Deamidated): 100; N12(Deamidated): 100; C27(Dehydro): 100; C33(Dehydro): 100 |  |  |  |  |  |  |  | 0.37 |  | 1 |  | Sequest HT (3) |  | 2 |  | 1683.88831 |  | 1.36 |  | 52.8961 |  | 16486 |  | 16486 |  | TRINITY\_DN0\_c1\_g1\_i14.p1,TRINITY\_DN0\_c1\_g1\_i4.p1 |  | Image |  | Peak List |  |
|  | | | | | | | | | | | | | | | | | | | | | | | | | | | | | | | | | | | | | | | | | | | | | | | | | | | | | |  |
|  | High |  | [R].GLLDTFKnLALnAAKSAGVSVLNSLScKLSKTc.[-] |  | HCD |  | N8(Deamidated); N12(Deamidated); C27(Dehydro); C33(Dehydro) |  | 4.03 |  | 1.3e-3 |  | 3.5e-2 |  | 0.415 |  | 175.94 |  | 0.000 |  | b1+(8): 890.46; b1+(9): 1003.55; b1+(10): 1074.58 |  | N8(Deamidated): 100; N23(Deamidated): 100; C27(Dehydro): 100; C33(Dehydro): 100 |  |  |  |  |  |  |  | 0.11 |  | 1 |  | Sequest HT (3) |  | 5 |  | 674.15863 |  | -0.21 |  | 52.8973 |  | 16487 |  | 16487 |  | TRINITY\_DN0\_c1\_g1\_i14.p1,TRINITY\_DN0\_c1\_g1\_i4.p1 |  | Image |  | Peak List |  |
|  | | | | | | | | | | | | | | | | | | | | | | | | | | | | | | | | | | | | | | | | | | | | | | | | | | | | | |  |
|  | High |  | [R].GLLDTFKnLALnAAKSAGVSVLNSLScKLSKTc.[-] |  | HCD |  | N8(Deamidated); N12(Deamidated); C27(Dehydro); C33(Dehydro) |  | 5.59 |  | 1.3e-3 |  | 2.0e-5 |  | 2.04 |  | 349.26 |  | 1.000 |  | b1+(12): 1302.69; b1+(14): 1444.77; y1+(16): 1636.84; y1+(15): 1579.82; y1+(14): 1480.75; y1+(12): 1294.65; y1+(11): 1181.57; b2+(12): 651.85; b2+(13): 687.37; b2+(16): 830.45; b2+(17): 865.97; b2+(19): 944.01 |  | N8(Deamidated): 100; N12(Deamidated): 100; C27(Dehydro): 100; C33(Dehydro): 100 |  |  |  |  |  |  |  | 0.07 |  | 1 |  | Sequest HT (3) |  | 3 |  | 1122.92651 |  | 0.07 |  | 52.9886 |  | 16521 |  | 16521 |  | TRINITY\_DN0\_c1\_g1\_i14.p1,TRINITY\_DN0\_c1\_g1\_i4.p1 |  | Image |  | Peak List |  |
|  | | | | | | | | | | | | | | | | | | | | | | | | | | | | | | | | | | | | | | | | | | | | | | | | | | | | | |  |
|  | High |  | [R].GLLDTFKnLALnAAKSAGVSVLNSLScKLSKTc.[-] |  | HCD |  | N8(Deamidated); N12(Deamidated); C27(Dehydro); C33(Dehydro) |  | 5.30 |  | 1.3e-3 |  | 2.1e-3 |  | 1.03 |  | 268.81 |  | 0.999 |  | b1+(12): 1302.69; b1+(13): 1373.73; b1+(14): 1444.77; y1+(14): 1480.75; y1+(12): 1294.65; y1+(11): 1181.57; b2+(13): 687.37; b2+(20): 987.53 |  | N8(Deamidated): 100; N12(Deamidated): 99.94; C27(Dehydro): 100; C33(Dehydro): 100 |  |  |  |  |  |  |  | 0.17 |  | 1 |  | Sequest HT (3) |  | 4 |  | 842.44739 |  | 0.89 |  | 52.9999 |  | 16526 |  | 16526 |  | TRINITY\_DN0\_c1\_g1\_i14.p1,TRINITY\_DN0\_c1\_g1\_i4.p1 |  | Image |  | Peak List |  |
|  | | | | | | | | | | | | | | | | | | | | | | | | | | | | | | | | | | | | | | | | | | | | | | | | | | | | | |  |
|  | High |  | [R].GLLDTFKnLALnAAKSAGVSVLNSLScKLSKTc.[-] |  | HCD |  | N8(Deamidated); N12(Deamidated); C27(Dehydro); C33(Dehydro) |  | 2.71 |  | 2.4e-3 |  | 8.2e-2 |  | 0.227 |  | 204.38 |  | 0.000 |  | b1+(8): 890.46; b1+(9): 1003.55; b1+(10): 1074.58; b1+(11): 1187.67 |  | N8(Deamidated): 100; N23(Deamidated): 100; C27(Dehydro): 100; C33(Dehydro): 100 |  |  |  |  |  |  |  | 0.18 |  | 1 |  | Sequest HT (3) |  | 2 |  | 1683.88477 |  | -0.74 |  | 53.0388 |  | 16539 |  | 16539 |  | TRINITY\_DN0\_c1\_g1\_i14.p1,TRINITY\_DN0\_c1\_g1\_i4.p1 |  | Image |  | Peak List |  |
|  | | | | | | | | | | | | | | | | | | | | | | | | | | | | | | | | | | | | | | | | | | | | | | | | | | | | | |  |
|  | High |  | [R].GLLDTFKNLALNAAKSAGVSVLNSLScKLSKTc.[-] |  | HCD |  | C27(Dehydro); C33(Dehydro) |  | 2.16 |  | 2.4e-3 |  | 8.1e-2 |  | 0.23 |  | 153.02 |  | 1.000 |  |  |  | C27(Dehydro): 100; C33(Dehydro): 100 |  |  |  |  |  |  |  | 0.32 |  | 1 |  | Sequest HT (3) |  | 4 |  | 841.95398 |  | -0.78 |  | 59.4995 |  | 18659 |  | 18659 |  | TRINITY\_DN0\_c1\_g1\_i14.p1,TRINITY\_DN0\_c1\_g1\_i4.p1 |  | Image |  | Peak List |  |
|  | | | | | | | | | | | | | | | | | | | | | | | | | | | | | | | | | | | | | | | | | | | | | | | | | | | | | |  |
|  | High |  | [R].GLLDTFKNLALNAAKSAGVSVLNSLScKLSKTc.[-] |  | HCD |  | C27(Dehydro); C33(Dehydro) |  | 1.99 |  | 4.7e-3 |  | 2.0e-1 |  | 0.025 |  | 114.90 |  | 1.000 |  |  |  | C27(Dehydro): 100; C33(Dehydro): 100 |  |  |  |  |  |  |  | 0.46 |  | 1 |  | Sequest HT (3) |  | 4 |  | 841.95404 |  | -0.71 |  | 61.0344 |  | 19171 |  | 19171 |  | TRINITY\_DN0\_c1\_g1\_i14.p1,TRINITY\_DN0\_c1\_g1\_i4.p1 |  | Image |  | Peak List |  |
|  | | | | | | | | | | | | | | | | | | | | | | | | | | | | | | | | | | | | | | | | | | | | | | | | | | | | | |  |
|  | **TRINITY\_DN0\_c1\_g1\_i24.p1 - TRINITY\_DN0\_c1\_g1\_i24.p1** | | | | | | | | | | | | | | | | | | | | | | | | | | | | | | | | | | | | | | | | | | | | | | | | | | | |  |  |
|  | | | | | | | | | | | | | | | | | | | | | | | | | | | | | | | | | | | | | | | | | | | | | | | | | | | | | |  |
|  | High |  | [L].AANFLPKLFcKITKKc.[-] |  | HCD |  | C10(Dehydro); C16(Dehydro) |  | 2.35 |  | 1.3e-3 |  | 5.3e-2 |  | 0.323 |  | 227.32 |  | 1.000 |  |  |  | C10(Dehydro): 100; C16(Dehydro): 100 |  |  |  |  |  |  |  | 0.37 |  | 1 |  | Sequest HT (3) |  | 3 |  | 608.34009 |  | -0.72 |  | 35.4671 |  | 8857 |  | 8857 |  | TRINITY\_DN0\_c1\_g1\_i24.p1\_ORF1; TRINITY\_DN0\_c1\_g1\_i24.p1; TRINITY\_DN0\_c1\_g1\_i6.p1 |  | Image |  | Peak List |  |
|  | | | | | | | | | | | | | | | | | | | | | | | | | | | | | | | | | | | | | | | | | | | | | | | | | | | | | |  |
|  | High |  | [G].LAANFLPKLFcKITKKc.[-] |  | HCD |  | C11(Dehydro); C17(Dehydro) |  | 3.31 |  | 3.1e-3 |  | 9.4e-2 |  | 0.196 |  | 230.66 |  | 1.000 |  |  |  | C11(Dehydro): 100; C17(Dehydro): 100 |  |  |  |  |  |  |  | 0.53 |  | 1 |  | Sequest HT (3) |  | 4 |  | 484.77902 |  | 1.64 |  | 37.2445 |  | 9710 |  | 9710 |  | TRINITY\_DN0\_c1\_g1\_i24.p1\_ORF1; TRINITY\_DN0\_c1\_g1\_i24.p1; TRINITY\_DN0\_c1\_g1\_i6.p1 |  | Image |  | Peak List |  |
|  | | | | | | | | | | | | | | | | | | | | | | | | | | | | | | | | | | | | | | | | | | | | | | | | | | | | | |  |
|  | High |  | [G].LAANFLPKLFcKITKKc.[-] |  | HCD |  | C11(Dehydro); C17(Dehydro) |  | 3.80 |  | 1.3e-3 |  | 2.2e-2 |  | 0.516 |  | 239.45 |  | 1.000 |  |  |  | C11(Dehydro): 100; C17(Dehydro): 100 |  |  |  |  |  |  |  | 0.51 |  | 1 |  | Sequest HT (3) |  | 3 |  | 646.03534 |  | 0.19 |  | 37.2740 |  | 9726 |  | 9726 |  | TRINITY\_DN0\_c1\_g1\_i24.p1\_ORF1; TRINITY\_DN0\_c1\_g1\_i24.p1; TRINITY\_DN0\_c1\_g1\_i6.p1 |  | Image |  | Peak List |  |
|  | | | | | | | | | | | | | | | | | | | | | | | | | | | | | | | | | | | | | | | | | | | | | | | | | | | | | |  |
|  | High |  | [G].LAANFLPKLFcKITKKc.[-] |  | HCD |  | C11(Dehydro); C17(Dehydro) |  | 2.65 |  | 3.1e-3 |  | 1.4e-1 |  | 0.105 |  | 200.60 |  | 1.000 |  |  |  | C11(Dehydro): 100; C17(Dehydro): 100 |  |  |  |  |  |  |  | 0.40 |  | 1 |  | Sequest HT (3) |  | 4 |  | 484.77802 |  | -0.45 |  | 37.3881 |  | 9786 |  | 9786 |  | TRINITY\_DN0\_c1\_g1\_i24.p1\_ORF1; TRINITY\_DN0\_c1\_g1\_i24.p1; TRINITY\_DN0\_c1\_g1\_i6.p1 |  | Image |  | Peak List |  |
|  | | | | | | | | | | | | | | | | | | | | | | | | | | | | | | | | | | | | | | | | | | | | | | | | | | | | | |  |
|  | High |  | [G].LAANFLPKLFcKITKKc.[-] |  | HCD |  | C11(Dehydro); C17(Dehydro) |  | 3.07 |  | 1.3e-3 |  | 3.6e-2 |  | 0.413 |  | 249.01 |  | 1.000 |  |  |  | C11(Dehydro): 100; C17(Dehydro): 100 |  |  |  |  |  |  |  | 0.41 |  | 1 |  | Sequest HT (3) |  | 3 |  | 646.03430 |  | -1.42 |  | 37.4150 |  | 9801 |  | 9801 |  | TRINITY\_DN0\_c1\_g1\_i24.p1\_ORF1; TRINITY\_DN0\_c1\_g1\_i24.p1; TRINITY\_DN0\_c1\_g1\_i6.p1 |  | Image |  | Peak List |  |
|  | | | | | | | | | | | | | | | | | | | | | | | | | | | | | | | | | | | | | | | | | | | | | | | | | | | | | |  |
|  | High |  | [A].GLAANFLPKLFcKITKKc.[-] |  | HCD |  | C12(Dehydro); C18(Dehydro) |  | 1.95 |  | 3.1e-3 |  | 1.4e-1 |  | 0.098 |  | 189.83 |  | 1.000 |  |  |  | C12(Dehydro): 100; C18(Dehydro): 100 |  |  |  |  |  |  |  | 0.37 |  | 1 |  | Sequest HT (3) |  | 4 |  | 499.03281 |  | -1.59 |  | 38.1544 |  | 10163 |  | 10163 |  | TRINITY\_DN0\_c1\_g1\_i24.p1\_ORF1; TRINITY\_DN0\_c1\_g1\_i24.p1; TRINITY\_DN0\_c1\_g1\_i6.p1 |  | Image |  | Peak List |  |
|  | | | | | | | | | | | | | | | | | | | | | | | | | | | | | | | | | | | | | | | | | | | | | | | | | | | | | |  |
|  | High |  | [A].GLAANFLPKLFcKITKKc.[-] |  | HCD |  | C12(Dehydro); C18(Dehydro) |  | 3.27 |  | 1.3e-3 |  | 5.4e-2 |  | 0.319 |  | 224.68 |  | 1.000 |  |  |  | C12(Dehydro): 100; C18(Dehydro): 100 |  |  |  |  |  |  |  | 0.52 |  | 1 |  | Sequest HT (3) |  | 3 |  | 665.04205 |  | -0.48 |  | 38.1915 |  | 10179 |  | 10179 |  | TRINITY\_DN0\_c1\_g1\_i24.p1\_ORF1; TRINITY\_DN0\_c1\_g1\_i24.p1; TRINITY\_DN0\_c1\_g1\_i6.p1 |  | Image |  | Peak List |  |
|  | | | | | | | | | | | | | | | | | | | | | | | | | | | | | | | | | | | | | | | | | | | | | | | | | | | | | |  |
|  | High |  | [A].GLAANFLPKLFcKITKKc.[-] |  | HCD |  | C12(Dehydro); C18(Dehydro) |  | 2.92 |  | 3.1e-3 |  | 1.1e-1 |  | 0.156 |  | 240.03 |  | 1.000 |  |  |  | C12(Dehydro): 100; C18(Dehydro): 100 |  |  |  |  |  |  |  | 0.57 |  | 1 |  | Sequest HT (3) |  | 2 |  | 997.06134 |  | 1.43 |  | 38.2815 |  | 10222 |  | 10222 |  | TRINITY\_DN0\_c1\_g1\_i24.p1\_ORF1; TRINITY\_DN0\_c1\_g1\_i24.p1; TRINITY\_DN0\_c1\_g1\_i6.p1 |  | Image |  | Peak List |  |
|  | | | | | | | | | | | | | | | | | | | | | | | | | | | | | | | | | | | | | | | | | | | | | | | | | | | | | |  |
|  | High |  | [L].AGLAANFLPKLFcKITKKc.[-] |  | HCD |  | C13(Dehydro); C19(Dehydro) |  | 3.56 |  | 1.3e-3 |  | 3.3e-2 |  | 0.432 |  | 200.21 |  | 1.000 |  |  |  | C13(Dehydro): 100; C19(Dehydro): 100 |  |  |  |  |  |  |  | 0.48 |  | 1 |  | Sequest HT (3) |  | 4 |  | 516.79242 |  | -0.88 |  | 38.3152 |  | 10240 |  | 10240 |  | TRINITY\_DN0\_c1\_g1\_i24.p1\_ORF1; TRINITY\_DN0\_c1\_g1\_i24.p1; TRINITY\_DN0\_c1\_g1\_i6.p1 |  | Image |  | Peak List |  |
|  | | | | | | | | | | | | | | | | | | | | | | | | | | | | | | | | | | | | | | | | | | | | | | | | | | | | | |  |
|  | High |  | [A].GLAANFLPKLFcKITKKc.[-] |  | HCD |  | C12(Dehydro); C18(Dehydro) |  | 3.07 |  | 1.3e-3 |  | 4.7e-2 |  | 0.352 |  | 240.90 |  | 1.000 |  |  |  | C12(Dehydro): 100; C18(Dehydro): 100 |  |  |  |  |  |  |  | 0.54 |  | 1 |  | Sequest HT (3) |  | 3 |  | 665.04266 |  | 0.44 |  | 38.3408 |  | 10252 |  | 10252 |  | TRINITY\_DN0\_c1\_g1\_i24.p1\_ORF1; TRINITY\_DN0\_c1\_g1\_i24.p1; TRINITY\_DN0\_c1\_g1\_i6.p1 |  | Image |  | Peak List |  |
|  | | | | | | | | | | | | | | | | | | | | | | | | | | | | | | | | | | | | | | | | | | | | | | | | | | | | | |  |
|  | High |  | [L].AGLAANFLPKLFcKITKKc.[-] |  | HCD |  | C13(Dehydro); C19(Dehydro) |  | 3.14 |  | 3.1e-3 |  | 1.3e-1 |  | 0.116 |  | 192.98 |  | 1.000 |  |  |  | C13(Dehydro): 100; C19(Dehydro): 100 |  |  |  |  |  |  |  | 0.39 |  | 1 |  | Sequest HT (3) |  | 3 |  | 688.72076 |  | -0.94 |  | 38.3477 |  | 10257 |  | 10257 |  | TRINITY\_DN0\_c1\_g1\_i24.p1\_ORF1; TRINITY\_DN0\_c1\_g1\_i24.p1; TRINITY\_DN0\_c1\_g1\_i6.p1 |  | Image |  | Peak List |  |
|  | | | | | | | | | | | | | | | | | | | | | | | | | | | | | | | | | | | | | | | | | | | | | | | | | | | | | |  |
|  | High |  | [L].AGLAANFLPKLFcKITKKc.[-] |  | HCD |  | C13(Dehydro); C19(Dehydro) |  | 3.39 |  | 3.1e-3 |  | 1.1e-1 |  | 0.152 |  | 216.93 |  | 1.000 |  |  |  | C13(Dehydro): 100; C19(Dehydro): 100 |  |  |  |  |  |  |  | 0.45 |  | 1 |  | Sequest HT (3) |  | 3 |  | 688.72278 |  | 1.99 |  | 38.6024 |  | 10379 |  | 10379 |  | TRINITY\_DN0\_c1\_g1\_i24.p1\_ORF1; TRINITY\_DN0\_c1\_g1\_i24.p1; TRINITY\_DN0\_c1\_g1\_i6.p1 |  | Image |  | Peak List |  |
|  | | | | | | | | | | | | | | | | | | | | | | | | | | | | | | | | | | | | | | | | | | | | | | | | | | | | | |  |
|  | High |  | [L].AGLAANFLPKLFcKITKKc.[-] |  | HCD |  | C13(Dehydro); C19(Dehydro) |  | 2.81 |  | 4.7e-3 |  | 1.9e-1 |  | 0.039 |  | 160.15 |  | 1.000 |  |  |  | C13(Dehydro): 100; C19(Dehydro): 100 |  |  |  |  |  |  |  | 0.33 |  | 1 |  | Sequest HT (3) |  | 4 |  | 516.79327 |  | 0.77 |  | 38.6036 |  | 10380 |  | 10380 |  | TRINITY\_DN0\_c1\_g1\_i24.p1\_ORF1; TRINITY\_DN0\_c1\_g1\_i24.p1; TRINITY\_DN0\_c1\_g1\_i6.p1 |  | Image |  | Peak List |  |
|  | | | | | | | | | | | | | | | | | | | | | | | | | | | | | | | | | | | | | | | | | | | | | | | | | | | | | |  |
|  | High |  | [G].LAAnFLPKLFcKITKKc.[-] |  | HCD |  | N4(Deamidated); C11(Dehydro); C17(Dehydro) |  | 3.01 |  | 1.3e-3 |  | 2.3e-2 |  | 0.505 |  | 217.85 |  | 1.000 |  |  |  | N4(Deamidated): 100; C11(Dehydro): 100; C17(Dehydro): 100 |  |  |  |  |  |  |  | 0.48 |  | 1 |  | Sequest HT (3) |  | 4 |  | 485.02484 |  | 1.25 |  | 39.0421 |  | 10596 |  | 10596 |  | TRINITY\_DN0\_c1\_g1\_i24.p1\_ORF1; TRINITY\_DN0\_c1\_g1\_i24.p1; TRINITY\_DN0\_c1\_g1\_i6.p1 |  | Image |  | Peak List |  |
|  | | | | | | | | | | | | | | | | | | | | | | | | | | | | | | | | | | | | | | | | | | | | | | | | | | | | | |  |
|  | High |  | [P].LLAGLAANFLPKLFcKITKKc.[-] |  | HCD |  | C15(Dehydro); C21(Dehydro) |  | 4.87 |  | 1.3e-3 |  | 1.1e-2 |  | 0.672 |  | 310.29 |  | 1.000 |  |  |  | C15(Dehydro): 100; C21(Dehydro): 100 |  |  |  |  |  |  |  | 0.50 |  | 1 |  | Sequest HT (3) |  | 3 |  | 764.11261 |  | 2.39 |  | 41.0038 |  | 11547 |  | 11547 |  | TRINITY\_DN0\_c1\_g1\_i24.p1\_ORF1; TRINITY\_DN0\_c1\_g1\_i24.p1; TRINITY\_DN0\_c1\_g1\_i6.p1 |  | Image |  | Peak List |  |
|  | | | | | | | | | | | | | | | | | | | | | | | | | | | | | | | | | | | | | | | | | | | | | | | | | | | | | |  |
|  | High |  | [P].LLAGLAANFLPKLFcKITKKc.[-] |  | HCD |  | C15(Dehydro); C21(Dehydro) |  | 4.00 |  | 1.3e-3 |  | 3.1e-2 |  | 0.444 |  | 210.34 |  | 1.000 |  |  |  | C15(Dehydro): 100; C21(Dehydro): 100 |  |  |  |  |  |  |  | 0.66 |  | 1 |  | Sequest HT (3) |  | 5 |  | 458.86902 |  | -0.79 |  | 41.0687 |  | 11578 |  | 11578 |  | TRINITY\_DN0\_c1\_g1\_i24.p1\_ORF1; TRINITY\_DN0\_c1\_g1\_i24.p1; TRINITY\_DN0\_c1\_g1\_i6.p1 |  | Image |  | Peak List |  |
|  | | | | | | | | | | | | | | | | | | | | | | | | | | | | | | | | | | | | | | | | | | | | | | | | | | | | | |  |
|  | High |  | [P].LLAGLAANFLPKLFcKITKKc.[-] |  | HCD |  | C15(Dehydro); C21(Dehydro) |  | 4.18 |  | 1.3e-3 |  | 3.0e-3 |  | 0.954 |  | 365.60 |  | 1.000 |  |  |  | C15(Dehydro): 100; C21(Dehydro): 100 |  |  |  |  |  |  |  | 0.64 |  | 1 |  | Sequest HT (3) |  | 2 |  | 1145.66333 |  | 0.69 |  | 41.1171 |  | 11600 |  | 11600 |  | TRINITY\_DN0\_c1\_g1\_i24.p1\_ORF1; TRINITY\_DN0\_c1\_g1\_i24.p1; TRINITY\_DN0\_c1\_g1\_i6.p1 |  | Image |  | Peak List |  |
|  | | | | | | | | | | | | | | | | | | | | | | | | | | | | | | | | | | | | | | | | | | | | | | | | | | | | | |  |
|  | High |  | [P].LLAGLAANFLPKLFcKITKKc.[-] |  | HCD |  | C15(Dehydro); C21(Dehydro) |  | 3.33 |  | 3.1e-3 |  | 1.1e-1 |  | 0.151 |  | 233.99 |  | 1.000 |  |  |  | C15(Dehydro): 100; C21(Dehydro): 100 |  |  |  |  |  |  |  | 0.60 |  | 1 |  | Sequest HT (3) |  | 3 |  | 764.11139 |  | 0.79 |  | 41.1445 |  | 11611 |  | 11611 |  | TRINITY\_DN0\_c1\_g1\_i24.p1\_ORF1; TRINITY\_DN0\_c1\_g1\_i24.p1; TRINITY\_DN0\_c1\_g1\_i6.p1 |  | Image |  | Peak List |  |
|  | | | | | | | | | | | | | | | | | | | | | | | | | | | | | | | | | | | | | | | | | | | | | | | | | | | | | |  |
|  | High |  | [P].LLAGLAAnFLPKLFcKITKKc.[-] |  | HCD |  | N8(Deamidated); C15(Dehydro); C21(Dehydro) |  | 3.33 |  | 3.1e-3 |  | 9.3e-2 |  | 0.198 |  | 239.21 |  | 1.000 |  |  |  | N8(Deamidated): 100; C15(Dehydro): 100; C21(Dehydro): 100 |  |  |  |  |  |  |  | 0.51 |  | 1 |  | Sequest HT (3) |  | 4 |  | 573.58099 |  | 0.14 |  | 41.3880 |  | 11715 |  | 11715 |  | TRINITY\_DN0\_c1\_g1\_i24.p1\_ORF1; TRINITY\_DN0\_c1\_g1\_i24.p1; TRINITY\_DN0\_c1\_g1\_i6.p1 |  | Image |  | Peak List |  |
|  | | | | | | | | | | | | | | | | | | | | | | | | | | | | | | | | | | | | | | | | | | | | | | | | | | | | | |  |
|  | High |  | [P].LLAGLAAnFLPKLFcKITKKc.[-] |  | HCD |  | N8(Deamidated); C15(Dehydro); C21(Dehydro) |  | 3.54 |  | 1.3e-3 |  | 3.8e-2 |  | 0.398 |  | 218.15 |  | 1.000 |  |  |  | N8(Deamidated): 100; C15(Dehydro): 100; C21(Dehydro): 100 |  |  |  |  |  |  |  | 0.56 |  | 1 |  | Sequest HT (3) |  | 4 |  | 573.58057 |  | -0.60 |  | 41.5336 |  | 11785 |  | 11785 |  | TRINITY\_DN0\_c1\_g1\_i24.p1\_ORF1; TRINITY\_DN0\_c1\_g1\_i24.p1; TRINITY\_DN0\_c1\_g1\_i6.p1 |  | Image |  | Peak List |  |
|  | | | | | | | | | | | | | | | | | | | | | | | | | | | | | | | | | | | | | | | | | | | | | | | | | | | | | |  |
|  | High |  | [L].PLLAGLAANFLPKLFcKITKKc.[-] |  | HCD |  | C16(Dehydro); C22(Dehydro) |  | 3.90 |  | 1.3e-3 |  | 6.1e-3 |  | 0.798 |  | 304.41 |  | 1.000 |  |  |  | C16(Dehydro): 100; C22(Dehydro): 100 |  |  |  |  |  |  |  | 0.63 |  | 1 |  | Sequest HT (3) |  | 4 |  | 597.59863 |  | 0.90 |  | 42.1372 |  | 12065 |  | 12065 |  | TRINITY\_DN0\_c1\_g1\_i24.p1\_ORF1; TRINITY\_DN0\_c1\_g1\_i24.p1; TRINITY\_DN0\_c1\_g1\_i6.p1 |  | Image |  | Peak List |  |
|  | | | | | | | | | | | | | | | | | | | | | | | | | | | | | | | | | | | | | | | | | | | | | | | | | | | | | |  |
|  | High |  | [F].LPLLAGLAANFLPKLFcKITKKc.[-] |  | HCD |  | C17(Dehydro); C23(Dehydro) |  | 3.98 |  | 1.3e-3 |  | 5.5e-3 |  | 0.82 |  | 291.70 |  | 1.000 |  |  |  | C17(Dehydro): 100; C23(Dehydro): 100 |  |  |  |  |  |  |  | 0.67 |  | 1 |  | Sequest HT (3) |  | 4 |  | 625.87006 |  | 1.51 |  | 44.5779 |  | 13063 |  | 13063 |  | TRINITY\_DN0\_c1\_g1\_i24.p1\_ORF1; TRINITY\_DN0\_c1\_g1\_i24.p1; TRINITY\_DN0\_c1\_g1\_i6.p1 |  | Image |  | Peak List |  |
|  | | | | | | | | | | | | | | | | | | | | | | | | | | | | | | | | | | | | | | | | | | | | | | | | | | | | | |  |
|  | High |  | [F].LPLLAGLAANFLPKLFcKITKKc.[-] |  | HCD |  | C17(Dehydro); C23(Dehydro) |  | 3.89 |  | 1.3e-3 |  | 1.5e-2 |  | 0.597 |  | 293.71 |  | 1.000 |  |  |  | C17(Dehydro): 100; C23(Dehydro): 100 |  |  |  |  |  |  |  | 0.58 |  | 1 |  | Sequest HT (3) |  | 4 |  | 625.86884 |  | -0.45 |  | 44.7421 |  | 13132 |  | 13132 |  | TRINITY\_DN0\_c1\_g1\_i24.p1\_ORF1; TRINITY\_DN0\_c1\_g1\_i24.p1; TRINITY\_DN0\_c1\_g1\_i6.p1 |  | Image |  | Peak List |  |
|  | | | | | | | | | | | | | | | | | | | | | | | | | | | | | | | | | | | | | | | | | | | | | | | | | | | | | |  |
|  | High |  | [R].FLPLLAGLAANFLPKLFcKITKKc.[-] |  | HCD |  | C18(Dehydro); C24(Dehydro) |  | 2.51 |  | 1.3e-3 |  | 6.8e-2 |  | 0.269 |  | 210.64 |  | 1.000 |  |  |  | C18(Dehydro): 100; C24(Dehydro): 100 |  |  |  |  |  |  |  | 0.46 |  | 1 |  | Sequest HT (3) |  | 5 |  | 530.31018 |  | -0.47 |  | 48.2988 |  | 14668 |  | 14668 |  | TRINITY\_DN0\_c1\_g1\_i24.p1\_ORF1; TRINITY\_DN0\_c1\_g1\_i24.p1; TRINITY\_DN0\_c1\_g1\_i6.p1 |  | Image |  | Peak List |  |
|  | | | | | | | | | | | | | | | | | | | | | | | | | | | | | | | | | | | | | | | | | | | | | | | | | | | | | |  |
|  | High |  | [R].FLPLLAGLAANFLPKLFcKITKKc.[-] |  | HCD |  | C18(Dehydro); C24(Dehydro) |  | 1.95 |  | 3.9e-3 |  | 1.5e-1 |  | 0.082 |  | 188.75 |  | 1.000 |  |  |  | C18(Dehydro): 100; C24(Dehydro): 100 |  |  |  |  |  |  |  | 0.39 |  | 1 |  | Sequest HT (3) |  | 5 |  | 530.30994 |  | -0.93 |  | 48.4465 |  | 14729 |  | 14729 |  | TRINITY\_DN0\_c1\_g1\_i24.p1\_ORF1; TRINITY\_DN0\_c1\_g1\_i24.p1; TRINITY\_DN0\_c1\_g1\_i6.p1 |  | Image |  | Peak List |  |
|  | | | | | | | | | | | | | | | | | | | | | | | | | | | | | | | | | | | | | | | | | | | | | | | | | | | | | |  |
|  | High |  | [R].FLPLLAGLAANFLPKLFcKITKKc.[-] |  | HCD |  | C18(Dehydro); C24(Dehydro) |  | 3.55 |  | 5.6e-3 |  | 2.1e-1 |  | 0.009 |  | 217.93 |  | 1.000 |  |  |  | C18(Dehydro): 100; C24(Dehydro): 100 |  |  |  |  |  |  |  | 0.44 |  | 1 |  | Sequest HT (3) |  | 4 |  | 662.63635 |  | 0.20 |  | 48.4841 |  | 14744 |  | 14744 |  | TRINITY\_DN0\_c1\_g1\_i24.p1\_ORF1; TRINITY\_DN0\_c1\_g1\_i24.p1; TRINITY\_DN0\_c1\_g1\_i6.p1 |  | Image |  | Peak List |  |
|  | | | | | | | | | | | | | | | | | | | | | | | | | | | | | | | | | | | | | | | | | | | | | | | | | | | | | |  |
|  | High |  | [R].FLPLLAGLAANFLPKLFcKITKKc.[-] |  | HCD |  | C18(Dehydro); C24(Dehydro) |  | 4.47 |  | 1.3e-3 |  | 1.7e-3 |  | 1.08 |  | 438.12 |  | 1.000 |  |  |  | C18(Dehydro): 100; C24(Dehydro): 100 |  |  |  |  |  |  |  | 0.66 |  | 1 |  | Sequest HT (3) |  | 2 |  | 1324.26147 |  | -2.78 |  | 48.5863 |  | 14778 |  | 14778 |  | TRINITY\_DN0\_c1\_g1\_i24.p1\_ORF1; TRINITY\_DN0\_c1\_g1\_i24.p1; TRINITY\_DN0\_c1\_g1\_i6.p1 |  | Image |  | Peak List |  |
|  | | | | | | | | | | | | | | | | | | | | | | | | | | | | | | | | | | | | | | | | | | | | | | | | | | | | | |  |
|  | High |  | [R].FLPLLAGLAANFLPKLFcKITKKc.[-] |  | HCD |  | C18(Dehydro); C24(Dehydro) |  | 4.18 |  | 3.1e-3 |  | 1.2e-1 |  | 0.142 |  | 266.89 |  | 1.000 |  |  |  | C18(Dehydro): 100; C24(Dehydro): 100 |  |  |  |  |  |  |  | 0.56 |  | 1 |  | Sequest HT (3) |  | 3 |  | 883.17694 |  | -2.56 |  | 48.7191 |  | 14829 |  | 14829 |  | TRINITY\_DN0\_c1\_g1\_i24.p1\_ORF1; TRINITY\_DN0\_c1\_g1\_i24.p1; TRINITY\_DN0\_c1\_g1\_i6.p1 |  | Image |  | Peak List |  |
|  | | | | | | | | | | | | | | | | | | | | | | | | | | | | | | | | | | | | | | | | | | | | | | | | | | | | | |  |
|  | High |  | [R].FLPLLAGLAANFLPKLFcKITKKc.[-] |  | HCD |  | C18(Dehydro); C24(Dehydro) |  | 4.58 |  | 1.3e-3 |  | 7.4e-4 |  | 1.26 |  | 429.65 |  | 1.000 |  |  |  | C18(Dehydro): 100; C24(Dehydro): 100 |  |  |  |  |  |  |  | 0.74 |  | 1 |  | Sequest HT (3) |  | 2 |  | 1324.26257 |  | -1.95 |  | 48.7293 |  | 14834 |  | 14834 |  | TRINITY\_DN0\_c1\_g1\_i24.p1\_ORF1; TRINITY\_DN0\_c1\_g1\_i24.p1; TRINITY\_DN0\_c1\_g1\_i6.p1 |  | Image |  | Peak List |  |
|  | | | | | | | | | | | | | | | | | | | | | | | | | | | | | | | | | | | | | | | | | | | | | | | | | | | | | |  |
|  | High |  | [R].FLPLLAGLAANFLPKLFcKITKKc.[-] |  | HCD |  | C18(Dehydro); C24(Dehydro) |  | 3.37 |  | 3.1e-3 |  | 9.9e-2 |  | 0.184 |  | 225.12 |  | 1.000 |  |  |  | C18(Dehydro): 100; C24(Dehydro): 100 |  |  |  |  |  |  |  | 0.45 |  | 1 |  | Sequest HT (3) |  | 4 |  | 662.63647 |  | 0.39 |  | 48.7638 |  | 14849 |  | 14849 |  | TRINITY\_DN0\_c1\_g1\_i24.p1\_ORF1; TRINITY\_DN0\_c1\_g1\_i24.p1; TRINITY\_DN0\_c1\_g1\_i6.p1 |  | Image |  | Peak List |  |
|  | | | | | | | | | | | | | | | | | | | | | | | | | | | | | | | | | | | | | | | | | | | | | | | | | | | | | |  |
|  | High |  | [R].FLPLLAGLAANFLPKLFcKITKKc.[-] |  | HCD |  | C18(Dehydro); C24(Dehydro) |  | 5.09 |  | 3.1e-3 |  | 1.1e-1 |  | 0.162 |  | 321.52 |  | 1.000 |  |  |  | C18(Dehydro): 100; C24(Dehydro): 100 |  |  |  |  |  |  |  | 0.65 |  | 1 |  | Sequest HT (3) |  | 3 |  | 883.17731 |  | -2.14 |  | 48.8574 |  | 14890 |  | 14890 |  | TRINITY\_DN0\_c1\_g1\_i24.p1\_ORF1; TRINITY\_DN0\_c1\_g1\_i24.p1; TRINITY\_DN0\_c1\_g1\_i6.p1 |  | Image |  | Peak List |  |
|  | | | | | | | | | | | | | | | | | | | | | | | | | | | | | | | | | | | | | | | | | | | | | | | | | | | | | |  |
|  | High |  | [R].FLPLLAGLAANFLPKLFcKITKKc.[-] |  | HCD |  | C18(Dehydro); C24(Dehydro) |  | 4.37 |  | 1.3e-3 |  | 4.7e-4 |  | 1.36 |  | 435.94 |  | 1.000 |  |  |  | C18(Dehydro): 100; C24(Dehydro): 100 |  |  |  |  |  |  |  | 0.69 |  | 1 |  | Sequest HT (3) |  | 2 |  | 1324.26221 |  | -2.23 |  | 48.8703 |  | 14896 |  | 14896 |  | TRINITY\_DN0\_c1\_g1\_i24.p1\_ORF1; TRINITY\_DN0\_c1\_g1\_i24.p1; TRINITY\_DN0\_c1\_g1\_i6.p1 |  | Image |  | Peak List |  |
|  | | | | | | | | | | | | | | | | | | | | | | | | | | | | | | | | | | | | | | | | | | | | | | | | | | | | | |  |
|  | High |  | [R].FLPLLAGLAANFLPKLFcKITKKc.[-] |  | HCD |  | C18(Dehydro); C24(Dehydro) |  | 2.73 |  | 1.3e-3 |  | 5.0e-2 |  | 0.337 |  | 210.77 |  | 1.000 |  |  |  | C18(Dehydro): 100; C24(Dehydro): 100 |  |  |  |  |  |  |  | 0.55 |  | 1 |  | Sequest HT (3) |  | 5 |  | 530.30981 |  | -1.16 |  | 48.8783 |  | 14899 |  | 14899 |  | TRINITY\_DN0\_c1\_g1\_i24.p1\_ORF1; TRINITY\_DN0\_c1\_g1\_i24.p1; TRINITY\_DN0\_c1\_g1\_i6.p1 |  | Image |  | Peak List |  |
|  | | | | | | | | | | | | | | | | | | | | | | | | | | | | | | | | | | | | | | | | | | | | | | | | | | | | | |  |
|  | High |  | [R].FLPLLAGLAANFLPKLFcKITKKc.[-] |  | HCD |  | C18(Dehydro); C24(Dehydro) |  | 3.10 |  | 3.1e-3 |  | 1.2e-1 |  | 0.142 |  | 218.75 |  | 1.000 |  |  |  | C18(Dehydro): 100; C24(Dehydro): 100 |  |  |  |  |  |  |  | 0.48 |  | 1 |  | Sequest HT (3) |  | 4 |  | 662.63599 |  | -0.35 |  | 48.9030 |  | 14908 |  | 14908 |  | TRINITY\_DN0\_c1\_g1\_i24.p1\_ORF1; TRINITY\_DN0\_c1\_g1\_i24.p1; TRINITY\_DN0\_c1\_g1\_i6.p1 |  | Image |  | Peak List |  |
|  | | | | | | | | | | | | | | | | | | | | | | | | | | | | | | | | | | | | | | | | | | | | | | | | | | | | | |  |
|  | High |  | [R].FLPLLAGLAANFLPKLFcKITKKc.[-] |  | HCD |  | C18(Dehydro); C24(Dehydro) |  | 3.15 |  | 1.3e-3 |  | 2.7e-2 |  | 0.474 |  | 331.86 |  | 1.000 |  |  |  | C18(Dehydro): 100; C24(Dehydro): 100 |  |  |  |  |  |  |  | 0.45 |  | 1 |  | Sequest HT (3) |  | 3 |  | 883.17755 |  | -1.87 |  | 49.0057 |  | 14951 |  | 14951 |  | TRINITY\_DN0\_c1\_g1\_i24.p1\_ORF1; TRINITY\_DN0\_c1\_g1\_i24.p1; TRINITY\_DN0\_c1\_g1\_i6.p1 |  | Image |  | Peak List |  |
|  | | | | | | | | | | | | | | | | | | | | | | | | | | | | | | | | | | | | | | | | | | | | | | | | | | | | | |  |
|  | High |  | [R].FLPLLAGLAANFLPKLFcKITKKc.[-] |  | HCD |  | C18(Dehydro); C24(Dehydro) |  | 4.17 |  | 1.3e-3 |  | 5.6e-4 |  | 1.32 |  | 433.80 |  | 1.000 |  |  |  | C18(Dehydro): 100; C24(Dehydro): 100 |  |  |  |  |  |  |  | 0.60 |  | 1 |  | Sequest HT (3) |  | 2 |  | 1324.26428 |  | -0.66 |  | 49.0146 |  | 14955 |  | 14955 |  | TRINITY\_DN0\_c1\_g1\_i24.p1\_ORF1; TRINITY\_DN0\_c1\_g1\_i24.p1; TRINITY\_DN0\_c1\_g1\_i6.p1 |  | Image |  | Peak List |  |
|  | | | | | | | | | | | | | | | | | | | | | | | | | | | | | | | | | | | | | | | | | | | | | | | | | | | | | |  |
|  | High |  | [R].FLPLLAGLAANFLPKLFcKITKKc.[-] |  | HCD |  | C18(Dehydro); C24(Dehydro) |  | 2.95 |  | 1.3e-3 |  | 4.5e-2 |  | 0.361 |  | 210.77 |  | 1.000 |  |  |  | C18(Dehydro): 100; C24(Dehydro): 100 |  |  |  |  |  |  |  | 0.63 |  | 1 |  | Sequest HT (3) |  | 5 |  | 530.31006 |  | -0.70 |  | 49.0159 |  | 14956 |  | 14956 |  | TRINITY\_DN0\_c1\_g1\_i24.p1\_ORF1; TRINITY\_DN0\_c1\_g1\_i24.p1; TRINITY\_DN0\_c1\_g1\_i6.p1 |  | Image |  | Peak List |  |
|  | | | | | | | | | | | | | | | | | | | | | | | | | | | | | | | | | | | | | | | | | | | | | | | | | | | | | |  |
|  | High |  | [R].FLPLLAGLAANFLPKLFcKITKKc.[-] |  | HCD |  | C18(Dehydro); C24(Dehydro) |  | 3.34 |  | 1.3e-3 |  | 1.6e-2 |  | 0.589 |  | 287.10 |  | 1.000 |  |  |  | C18(Dehydro): 100; C24(Dehydro): 100 |  |  |  |  |  |  |  | 0.50 |  | 1 |  | Sequest HT (3) |  | 3 |  | 883.17755 |  | -1.87 |  | 49.1576 |  | 15011 |  | 15011 |  | TRINITY\_DN0\_c1\_g1\_i24.p1\_ORF1; TRINITY\_DN0\_c1\_g1\_i24.p1; TRINITY\_DN0\_c1\_g1\_i6.p1 |  | Image |  | Peak List |  |
|  | | | | | | | | | | | | | | | | | | | | | | | | | | | | | | | | | | | | | | | | | | | | | | | | | | | | | |  |
|  | High |  | [R].FLPLLAGLAANFLPKLFcKITKKc.[-] |  | HCD |  | C18(Dehydro); C24(Dehydro) |  | 4.73 |  | 1.3e-3 |  | 2.4e-3 |  | 1 |  | 446.84 |  | 1.000 |  |  |  | C18(Dehydro): 100; C24(Dehydro): 100 |  |  |  |  |  |  |  | 0.63 |  | 1 |  | Sequest HT (3) |  | 2 |  | 1324.26123 |  | -2.97 |  | 49.1589 |  | 15012 |  | 15012 |  | TRINITY\_DN0\_c1\_g1\_i24.p1\_ORF1; TRINITY\_DN0\_c1\_g1\_i24.p1; TRINITY\_DN0\_c1\_g1\_i6.p1 |  | Image |  | Peak List |  |
|  | | | | | | | | | | | | | | | | | | | | | | | | | | | | | | | | | | | | | | | | | | | | | | | | | | | | | |  |
|  | High |  | [R].FLPLLAGLAANFLPKLFcKITKKc.[-] |  | HCD |  | C18(Dehydro); C24(Dehydro) |  | 2.61 |  | 3.1e-3 |  | 1.0e-1 |  | 0.179 |  | 181.12 |  | 1.000 |  |  |  | C18(Dehydro): 100; C24(Dehydro): 100 |  |  |  |  |  |  |  | 0.55 |  | 1 |  | Sequest HT (3) |  | 5 |  | 530.30939 |  | -1.97 |  | 49.1614 |  | 15014 |  | 15014 |  | TRINITY\_DN0\_c1\_g1\_i24.p1\_ORF1; TRINITY\_DN0\_c1\_g1\_i24.p1; TRINITY\_DN0\_c1\_g1\_i6.p1 |  | Image |  | Peak List |  |
|  | | | | | | | | | | | | | | | | | | | | | | | | | | | | | | | | | | | | | | | | | | | | | | | | | | | | | |  |
|  | High |  | [R].FLPLLAGLAANFLPKLFcKITKKc.[-] |  | HCD |  | C18(Dehydro); C24(Dehydro) |  | 2.55 |  | 1.3e-3 |  | 6.2e-2 |  | 0.29 |  | 218.54 |  | 1.000 |  |  |  | C18(Dehydro): 100; C24(Dehydro): 100 |  |  |  |  |  |  |  | 0.55 |  | 1 |  | Sequest HT (3) |  | 4 |  | 662.63574 |  | -0.72 |  | 49.1830 |  | 15024 |  | 15024 |  | TRINITY\_DN0\_c1\_g1\_i24.p1\_ORF1; TRINITY\_DN0\_c1\_g1\_i24.p1; TRINITY\_DN0\_c1\_g1\_i6.p1 |  | Image |  | Peak List |  |
|  | | | | | | | | | | | | | | | | | | | | | | | | | | | | | | | | | | | | | | | | | | | | | | | | | | | | | |  |
|  | High |  | [R].FLPLLAGLAAnFLPKLFcKITKKc.[-] |  | HCD |  | N11(Deamidated); C18(Dehydro); C24(Dehydro) |  | 3.68 |  | 1.3e-3 |  | 2.4e-2 |  | 0.501 |  | 263.29 |  | 1.000 |  |  |  | N11(Deamidated): 100; C18(Dehydro): 100; C24(Dehydro): 100 |  |  |  |  |  |  |  | 0.54 |  | 1 |  | Sequest HT (3) |  | 4 |  | 662.88361 |  | 2.09 |  | 49.6245 |  | 15197 |  | 15197 |  | TRINITY\_DN0\_c1\_g1\_i24.p1\_ORF1; TRINITY\_DN0\_c1\_g1\_i24.p1; TRINITY\_DN0\_c1\_g1\_i6.p1 |  | Image |  | Peak List |  |
|  | | | | | | | | | | | | | | | | | | | | | | | | | | | | | | | | | | | | | | | | | | | | | | | | | | | | | |  |
|  | High |  | [R].FLPLLAGLAAnFLPKLFcKITKKc.[-] |  | HCD |  | N11(Deamidated); C18(Dehydro); C24(Dehydro) |  | 4.64 |  | 1.3e-3 |  | 5.4e-3 |  | 0.826 |  | 314.22 |  | 1.000 |  |  |  | N11(Deamidated): 100; C18(Dehydro): 100; C24(Dehydro): 100 |  |  |  |  |  |  |  | 0.58 |  | 1 |  | Sequest HT (3) |  | 3 |  | 883.50781 |  | 0.69 |  | 49.7057 |  | 15231 |  | 15231 |  | TRINITY\_DN0\_c1\_g1\_i24.p1\_ORF1; TRINITY\_DN0\_c1\_g1\_i24.p1; TRINITY\_DN0\_c1\_g1\_i6.p1 |  | Image |  | Peak List |  |
|  | | | | | | | | | | | | | | | | | | | | | | | | | | | | | | | | | | | | | | | | | | | | | | | | | | | | | |  |
|  | High |  | [R].FLPLLAGLAAnFLPKLFcKITKKc.[-] |  | HCD |  | N11(Deamidated); C18(Dehydro); C24(Dehydro) |  | 4.54 |  | 1.3e-3 |  | 1.4e-2 |  | 0.614 |  | 290.53 |  | 1.000 |  |  |  | N11(Deamidated): 100; C18(Dehydro): 100; C24(Dehydro): 100 |  |  |  |  |  |  |  | 0.54 |  | 1 |  | Sequest HT (3) |  | 4 |  | 662.88293 |  | 1.08 |  | 49.7703 |  | 15257 |  | 15257 |  | TRINITY\_DN0\_c1\_g1\_i24.p1\_ORF1; TRINITY\_DN0\_c1\_g1\_i24.p1; TRINITY\_DN0\_c1\_g1\_i6.p1 |  | Image |  | Peak List |  |
|  | | | | | | | | | | | | | | | | | | | | | | | | | | | | | | | | | | | | | | | | | | | | | | | | | | | | | |  |
|  | High |  | [R].FLPLLAGLAAnFLPKLFcKITKKc.[-] |  | HCD |  | N11(Deamidated); C18(Dehydro); C24(Dehydro) |  | 4.08 |  | 1.3e-3 |  | 1.3e-2 |  | 0.64 |  | 303.73 |  | 1.000 |  |  |  | N11(Deamidated): 100; C18(Dehydro): 100; C24(Dehydro): 100 |  |  |  |  |  |  |  | 0.55 |  | 1 |  | Sequest HT (3) |  | 4 |  | 662.88269 |  | 0.71 |  | 49.9350 |  | 15322 |  | 15322 |  | TRINITY\_DN0\_c1\_g1\_i24.p1\_ORF1; TRINITY\_DN0\_c1\_g1\_i24.p1; TRINITY\_DN0\_c1\_g1\_i6.p1 |  | Image |  | Peak List |  |
|  | | | | | | | | | | | | | | | | | | | | | | | | | | | | | | | | | | | | | | | | | | | | | | | | | | | | | |  |
|  | High |  | [R].FLPLLAGLAAnFLPKLFcKITKKc.[-] |  | HCD |  | N11(Deamidated); C18(Dehydro); C24(Dehydro) |  | 4.47 |  | 1.3e-3 |  | 3.9e-3 |  | 0.894 |  | 278.67 |  | 1.000 |  |  |  | N11(Deamidated): 100; C18(Dehydro): 100; C24(Dehydro): 100 |  |  |  |  |  |  |  | 0.62 |  | 1 |  | Sequest HT (3) |  | 4 |  | 662.88202 |  | -0.31 |  | 50.0796 |  | 15382 |  | 15382 |  | TRINITY\_DN0\_c1\_g1\_i24.p1\_ORF1; TRINITY\_DN0\_c1\_g1\_i24.p1; TRINITY\_DN0\_c1\_g1\_i6.p1 |  | Image |  | Peak List |  |
|  | | | | | | | | | | | | | | | | | | | | | | | | | | | | | | | | | | | | | | | | | | | | | | | | | | | | | |  |
|  | High |  | [R].FLPLLAGLAAnFLPKLFcKITKKC.[-] |  | HCD |  | N11(Deamidated); C18(Dehydro) |  | 2.65 |  | 1.3e-3 |  | 3.3e-2 |  | 0.427 |  | 248.63 |  | 0.500 |  |  |  | N11(Deamidated): 100; C18(Dehydro): 50; C24(Dehydro): 50 |  |  |  |  |  |  |  | 0.01 |  | 1 |  | Sequest HT (3) |  | 4 |  | 663.13300 |  | -1.78 |  | 50.0796 |  | 15382 |  | 15382 |  | TRINITY\_DN0\_c1\_g1\_i24.p1\_ORF1; TRINITY\_DN0\_c1\_g1\_i24.p1; TRINITY\_DN0\_c1\_g1\_i6.p1 |  | Image |  | Peak List |  |
|  | | | | | | | | | | | | | | | | | | | | | | | | | | | | | | | | | | | | | | | | | | | | | | | | | | | | | |  |
|  | High |  | [R].FLPLLAGLAAnFLPKLFCKITKKc.[-] |  | HCD |  | N11(Deamidated); C24(Dehydro) |  | 2.63 |  | 1.3e-3 |  | 3.3e-2 |  | 0.429 |  | 248.63 |  | 0.500 |  |  |  | N11(Deamidated): 100; C18(Dehydro): 50; C24(Dehydro): 50 |  |  |  |  |  |  |  |  |  | 2 |  | Sequest HT (3) |  | 4 |  | 663.13300 |  | -1.78 |  | 50.0796 |  | 15382 |  | 15382 |  | TRINITY\_DN0\_c1\_g1\_i24.p1\_ORF1; TRINITY\_DN0\_c1\_g1\_i24.p1; TRINITY\_DN0\_c1\_g1\_i6.p1 |  | Image |  | Peak List |  |
|  | | | | | | | | | | | | | | | | | | | | | | | | | | | | | | | | | | | | | | | | | | | | | | | | | | | | | |  |
|  | High |  | [R].FLPLLAGLAAnFLPKLFcKITKKc.[-] |  | HCD |  | N11(Deamidated); C18(Dehydro); C24(Dehydro) |  | 4.51 |  | 1.3e-3 |  | 2.6e-3 |  | 0.985 |  | 323.51 |  | 1.000 |  |  |  | N11(Deamidated): 100; C18(Dehydro): 100; C24(Dehydro): 100 |  |  |  |  |  |  |  | 0.64 |  | 1 |  | Sequest HT (3) |  | 3 |  | 883.50757 |  | 0.41 |  | 50.1979 |  | 15430 |  | 15430 |  | TRINITY\_DN0\_c1\_g1\_i24.p1\_ORF1; TRINITY\_DN0\_c1\_g1\_i24.p1; TRINITY\_DN0\_c1\_g1\_i6.p1 |  | Image |  | Peak List |  |
|  | | | | | | | | | | | | | | | | | | | | | | | | | | | | | | | | | | | | | | | | | | | | | | | | | | | | | |  |
|  | High |  | [R].FLPLLAGLAAnFLPKLFcKITKKc.[-] |  | HCD |  | N11(Deamidated); C18(Dehydro); C24(Dehydro) |  | 4.03 |  | 1.3e-3 |  | 6.5e-3 |  | 0.784 |  | 300.84 |  | 1.000 |  |  |  | N11(Deamidated): 100; C18(Dehydro): 100; C24(Dehydro): 100 |  |  |  |  |  |  |  | 0.59 |  | 1 |  | Sequest HT (3) |  | 4 |  | 662.88208 |  | -0.21 |  | 50.2195 |  | 15436 |  | 15436 |  | TRINITY\_DN0\_c1\_g1\_i24.p1\_ORF1; TRINITY\_DN0\_c1\_g1\_i24.p1; TRINITY\_DN0\_c1\_g1\_i6.p1 |  | Image |  | Peak List |  |
|  | | | | | | | | | | | | | | | | | | | | | | | | | | | | | | | | | | | | | | | | | | | | | | | | | | | | | |  |
|  | High |  | [R].FLPLLAGLAAnFLPKLFcKITKKc.[-] |  | HCD |  | N11(Deamidated); C18(Dehydro); C24(Dehydro) |  | 4.26 |  | 1.3e-3 |  | 6.6e-3 |  | 0.782 |  | 260.42 |  | 1.000 |  |  |  | N11(Deamidated): 100; C18(Dehydro): 100; C24(Dehydro): 100 |  |  |  |  |  |  |  | 0.64 |  | 1 |  | Sequest HT (3) |  | 4 |  | 662.88269 |  | 0.71 |  | 50.3623 |  | 15493 |  | 15493 |  | TRINITY\_DN0\_c1\_g1\_i24.p1\_ORF1; TRINITY\_DN0\_c1\_g1\_i24.p1; TRINITY\_DN0\_c1\_g1\_i6.p1 |  | Image |  | Peak List |  |
|  | | | | | | | | | | | | | | | | | | | | | | | | | | | | | | | | | | | | | | | | | | | | | | | | | | | | | |  |
|  | High |  | [R].FLPLLAGLAAnFLPKLFcKITKKc.[-] |  | HCD |  | N11(Deamidated); C18(Dehydro); C24(Dehydro) |  | 5.19 |  | 1.3e-3 |  | 1.5e-3 |  | 1.11 |  | 425.65 |  | 1.000 |  |  |  | N11(Deamidated): 100; C18(Dehydro): 100; C24(Dehydro): 100 |  |  |  |  |  |  |  | 0.74 |  | 1 |  | Sequest HT (3) |  | 2 |  | 1324.75525 |  | -1.45 |  | 50.4644 |  | 15533 |  | 15533 |  | TRINITY\_DN0\_c1\_g1\_i24.p1\_ORF1; TRINITY\_DN0\_c1\_g1\_i24.p1; TRINITY\_DN0\_c1\_g1\_i6.p1 |  | Image |  | Peak List |  |
|  | | | | | | | | | | | | | | | | | | | | | | | | | | | | | | | | | | | | | | | | | | | | | | | | | | | | | |  |
|  | High |  | [R].FLPLLAGLAAnFLPKLFcKITKKc.[-] |  | HCD |  | N11(Deamidated); C18(Dehydro); C24(Dehydro) |  | 3.88 |  | 1.3e-3 |  | 1.1e-2 |  | 0.666 |  | 237.10 |  | 1.000 |  |  |  | N11(Deamidated): 100; C18(Dehydro): 100; C24(Dehydro): 100 |  |  |  |  |  |  |  | 0.57 |  | 1 |  | Sequest HT (3) |  | 4 |  | 662.88269 |  | 0.71 |  | 50.5041 |  | 15549 |  | 15549 |  | TRINITY\_DN0\_c1\_g1\_i24.p1\_ORF1; TRINITY\_DN0\_c1\_g1\_i24.p1; TRINITY\_DN0\_c1\_g1\_i6.p1 |  | Image |  | Peak List |  |
|  | | | | | | | | | | | | | | | | | | | | | | | | | | | | | | | | | | | | | | | | | | | | | | | | | | | | | |  |
|  | High |  | [R].FLPLLAGLAAnFLPKLFcKITKKc.[-] |  | HCD |  | N11(Deamidated); C18(Dehydro); C24(Dehydro) |  | 2.94 |  | 1.3e-3 |  | 1.3e-2 |  | 0.632 |  | 268.47 |  | 1.000 |  |  |  | N11(Deamidated): 100; C18(Dehydro): 100; C24(Dehydro): 100 |  |  |  |  |  |  |  | 0.53 |  | 1 |  | Sequest HT (3) |  | 5 |  | 530.50684 |  | -0.75 |  | 50.5539 |  | 15568 |  | 15568 |  | TRINITY\_DN0\_c1\_g1\_i24.p1\_ORF1; TRINITY\_DN0\_c1\_g1\_i24.p1; TRINITY\_DN0\_c1\_g1\_i6.p1 |  | Image |  | Peak List |  |
|  | | | | | | | | | | | | | | | | | | | | | | | | | | | | | | | | | | | | | | | | | | | | | | | | | | | | | |  |
|  | High |  | [R].FLPLLAGLAAnFLPKLFcKITKKc.[-] |  | HCD |  | N11(Deamidated); C18(Dehydro); C24(Dehydro) |  | 5.44 |  | 1.3e-3 |  | 2.6e-4 |  | 1.48 |  | 500.88 |  | 1.000 |  |  |  | N11(Deamidated): 100; C18(Dehydro): 100; C24(Dehydro): 100 |  |  |  |  |  |  |  | 0.74 |  | 1 |  | Sequest HT (3) |  | 2 |  | 1324.75842 |  | 0.95 |  | 50.6062 |  | 15587 |  | 15587 |  | TRINITY\_DN0\_c1\_g1\_i24.p1\_ORF1; TRINITY\_DN0\_c1\_g1\_i24.p1; TRINITY\_DN0\_c1\_g1\_i6.p1 |  | Image |  | Peak List |  |
|  | | | | | | | | | | | | | | | | | | | | | | | | | | | | | | | | | | | | | | | | | | | | | | | | | | | | | |  |
|  | High |  | [R].FLPLLAGLAAnFLPKLFcKITKKc.[-] |  | HCD |  | N11(Deamidated); C18(Dehydro); C24(Dehydro) |  | 3.43 |  | 3.1e-3 |  | 1.0e-1 |  | 0.178 |  | 256.42 |  | 1.000 |  |  |  | N11(Deamidated): 100; C18(Dehydro): 100; C24(Dehydro): 100 |  |  |  |  |  |  |  | 0.52 |  | 1 |  | Sequest HT (3) |  | 4 |  | 662.88281 |  | 0.89 |  | 50.6475 |  | 15602 |  | 15602 |  | TRINITY\_DN0\_c1\_g1\_i24.p1\_ORF1; TRINITY\_DN0\_c1\_g1\_i24.p1; TRINITY\_DN0\_c1\_g1\_i6.p1 |  | Image |  | Peak List |  |
|  | | | | | | | | | | | | | | | | | | | | | | | | | | | | | | | | | | | | | | | | | | | | | | | | | | | | | |  |
|  | High |  | [R].FLPLLAGLAAnFLPKLFcKITKKc.[-] |  | HCD |  | N11(Deamidated); C18(Dehydro); C24(Dehydro) |  | 3.85 |  | 1.3e-3 |  | 1.0e-2 |  | 0.684 |  | 349.20 |  | 1.000 |  |  |  | N11(Deamidated): 100; C18(Dehydro): 100; C24(Dehydro): 100 |  |  |  |  |  |  |  | 0.55 |  | 1 |  | Sequest HT (3) |  | 3 |  | 883.50604 |  | -1.31 |  | 50.6496 |  | 15603 |  | 15603 |  | TRINITY\_DN0\_c1\_g1\_i24.p1\_ORF1; TRINITY\_DN0\_c1\_g1\_i24.p1; TRINITY\_DN0\_c1\_g1\_i6.p1 |  | Image |  | Peak List |  |
|  | | | | | | | | | | | | | | | | | | | | | | | | | | | | | | | | | | | | | | | | | | | | | | | | | | | | | |  |
|  | High |  | [R].FLPLLAGLAAnFLPKLFcKITKKc.[-] |  | HCD |  | N11(Deamidated); C18(Dehydro); C24(Dehydro) |  | 2.98 |  | 4.7e-3 |  | 2.0e-1 |  | 0.024 |  | 208.59 |  | 1.000 |  |  |  | N11(Deamidated): 100; C18(Dehydro): 100; C24(Dehydro): 100 |  |  |  |  |  |  |  | 0.51 |  | 1 |  | Sequest HT (3) |  | 5 |  | 530.50714 |  | -0.17 |  | 50.6920 |  | 15620 |  | 15620 |  | TRINITY\_DN0\_c1\_g1\_i24.p1\_ORF1; TRINITY\_DN0\_c1\_g1\_i24.p1; TRINITY\_DN0\_c1\_g1\_i6.p1 |  | Image |  | Peak List |  |
|  | | | | | | | | | | | | | | | | | | | | | | | | | | | | | | | | | | | | | | | | | | | | | | | | | | | | | |  |
|  | High |  | [R].FLPLLAGLAAnFLPKLFcKITKKc.[-] |  | HCD |  | N11(Deamidated); C18(Dehydro); C24(Dehydro) |  | 4.98 |  | 1.3e-3 |  | 6.5e-3 |  | 0.784 |  | 385.78 |  | 1.000 |  |  |  | N11(Deamidated): 100; C18(Dehydro): 100; C24(Dehydro): 100 |  |  |  |  |  |  |  | 0.73 |  | 1 |  | Sequest HT (3) |  | 2 |  | 1324.75708 |  | -0.06 |  | 50.7769 |  | 15651 |  | 15651 |  | TRINITY\_DN0\_c1\_g1\_i24.p1\_ORF1; TRINITY\_DN0\_c1\_g1\_i24.p1; TRINITY\_DN0\_c1\_g1\_i6.p1 |  | Image |  | Peak List |  |
|  | | | | | | | | | | | | | | | | | | | | | | | | | | | | | | | | | | | | | | | | | | | | | | | | | | | | | |  |
|  | High |  | [R].FLPLLAGLAAnFLPKLFcKITKKc.[-] |  | HCD |  | N11(Deamidated); C18(Dehydro); C24(Dehydro) |  | 3.17 |  | 2.4e-3 |  | 7.6e-2 |  | 0.246 |  | 268.47 |  | 1.000 |  |  |  | N11(Deamidated): 100; C18(Dehydro): 100; C24(Dehydro): 100 |  |  |  |  |  |  |  | 0.54 |  | 1 |  | Sequest HT (3) |  | 4 |  | 662.88226 |  | 0.06 |  | 50.7889 |  | 15657 |  | 15657 |  | TRINITY\_DN0\_c1\_g1\_i24.p1\_ORF1; TRINITY\_DN0\_c1\_g1\_i24.p1; TRINITY\_DN0\_c1\_g1\_i6.p1 |  | Image |  | Peak List |  |
|  | | | | | | | | | | | | | | | | | | | | | | | | | | | | | | | | | | | | | | | | | | | | | | | | | | | | | |  |
|  | High |  | [R].FLPLLAGLAAnFLPKLFcKITKKc.[-] |  | HCD |  | N11(Deamidated); C18(Dehydro); C24(Dehydro) |  | 2.65 |  | 1.3e-3 |  | 5.5e-2 |  | 0.315 |  | 202.48 |  | 1.000 |  |  |  | N11(Deamidated): 100; C18(Dehydro): 100; C24(Dehydro): 100 |  |  |  |  |  |  |  | 0.54 |  | 1 |  | Sequest HT (3) |  | 5 |  | 530.50708 |  | -0.29 |  | 50.8818 |  | 15693 |  | 15693 |  | TRINITY\_DN0\_c1\_g1\_i24.p1\_ORF1; TRINITY\_DN0\_c1\_g1\_i24.p1; TRINITY\_DN0\_c1\_g1\_i6.p1 |  | Image |  | Peak List |  |
|  | | | | | | | | | | | | | | | | | | | | | | | | | | | | | | | | | | | | | | | | | | | | | | | | | | | | | |  |
|  | High |  | [R].FLPLLAGLAAnFLPKLFcKITKKc.[-] |  | HCD |  | N11(Deamidated); C18(Dehydro); C24(Dehydro) |  | 4.61 |  | 1.3e-3 |  | 1.5e-4 |  | 1.61 |  | 466.16 |  | 1.000 |  |  |  | N11(Deamidated): 100; C18(Dehydro): 100; C24(Dehydro): 100 |  |  |  |  |  |  |  | 0.74 |  | 1 |  | Sequest HT (3) |  | 2 |  | 1324.76099 |  | 2.88 |  | 50.9122 |  | 15703 |  | 15703 |  | TRINITY\_DN0\_c1\_g1\_i24.p1\_ORF1; TRINITY\_DN0\_c1\_g1\_i24.p1; TRINITY\_DN0\_c1\_g1\_i6.p1 |  | Image |  | Peak List |  |
|  | | | | | | | | | | | | | | | | | | | | | | | | | | | | | | | | | | | | | | | | | | | | | | | | | | | | | |  |
|  | High |  | [R].FLPLLAGLAAnFLPKLFcKITKKc.[-] |  | HCD |  | N11(Deamidated); C18(Dehydro); C24(Dehydro) |  | 3.82 |  | 1.3e-3 |  | 2.5e-2 |  | 0.495 |  | 258.08 |  | 1.000 |  |  |  | N11(Deamidated): 100; C18(Dehydro): 100; C24(Dehydro): 100 |  |  |  |  |  |  |  | 0.56 |  | 1 |  | Sequest HT (3) |  | 4 |  | 662.88220 |  | -0.03 |  | 50.9243 |  | 15709 |  | 15709 |  | TRINITY\_DN0\_c1\_g1\_i24.p1\_ORF1; TRINITY\_DN0\_c1\_g1\_i24.p1; TRINITY\_DN0\_c1\_g1\_i6.p1 |  | Image |  | Peak List |  |
|  | | | | | | | | | | | | | | | | | | | | | | | | | | | | | | | | | | | | | | | | | | | | | | | | | | | | | |  |
|  | High |  | [R].FLPLLAGLAAnFLPKLFcKITKKc.[-] |  | HCD |  | N11(Deamidated); C18(Dehydro); C24(Dehydro) |  | 4.74 |  | 1.3e-3 |  | 2.3e-3 |  | 1.01 |  | 333.12 |  | 1.000 |  |  |  | N11(Deamidated): 100; C18(Dehydro): 100; C24(Dehydro): 100 |  |  |  |  |  |  |  | 0.64 |  | 1 |  | Sequest HT (3) |  | 3 |  | 883.50867 |  | 1.66 |  | 50.9748 |  | 15730 |  | 15730 |  | TRINITY\_DN0\_c1\_g1\_i24.p1\_ORF1; TRINITY\_DN0\_c1\_g1\_i24.p1; TRINITY\_DN0\_c1\_g1\_i6.p1 |  | Image |  | Peak List |  |
|  | | | | | | | | | | | | | | | | | | | | | | | | | | | | | | | | | | | | | | | | | | | | | | | | | | | | | |  |
|  | High |  | [R].FLPLLAGLAAnFLPKLFcKITKKc.[-] |  | HCD |  | N11(Deamidated); C18(Dehydro); C24(Dehydro) |  | 3.25 |  | 1.3e-3 |  | 5.3e-2 |  | 0.326 |  | 226.81 |  | 1.000 |  |  |  | N11(Deamidated): 100; C18(Dehydro): 100; C24(Dehydro): 100 |  |  |  |  |  |  |  | 0.62 |  | 1 |  | Sequest HT (3) |  | 5 |  | 530.50696 |  | -0.52 |  | 51.0305 |  | 15752 |  | 15752 |  | TRINITY\_DN0\_c1\_g1\_i24.p1\_ORF1; TRINITY\_DN0\_c1\_g1\_i24.p1; TRINITY\_DN0\_c1\_g1\_i6.p1 |  | Image |  | Peak List |  |
|  | | | | | | | | | | | | | | | | | | | | | | | | | | | | | | | | | | | | | | | | | | | | | | | | | | | | | |  |
|  | High |  | [R].FLPLLAGLAAnFLPKLFcKITKKc.[-] |  | HCD |  | N11(Deamidated); C18(Dehydro); C24(Dehydro) |  | 5.18 |  | 1.3e-3 |  | 1.2e-4 |  | 1.66 |  | 421.78 |  | 1.000 |  |  |  | N11(Deamidated): 100; C18(Dehydro): 100; C24(Dehydro): 100 |  |  |  |  |  |  |  | 0.75 |  | 1 |  | Sequest HT (3) |  | 2 |  | 1324.75684 |  | -0.25 |  | 51.0506 |  | 15760 |  | 15760 |  | TRINITY\_DN0\_c1\_g1\_i24.p1\_ORF1; TRINITY\_DN0\_c1\_g1\_i24.p1; TRINITY\_DN0\_c1\_g1\_i6.p1 |  | Image |  | Peak List |  |
|  | | | | | | | | | | | | | | | | | | | | | | | | | | | | | | | | | | | | | | | | | | | | | | | | | | | | | |  |
|  | High |  | [R].FLPLLAGLAAnFLPKLFcKITKKc.[-] |  | HCD |  | N11(Deamidated); C18(Dehydro); C24(Dehydro) |  | 3.95 |  | 1.3e-3 |  | 8.7e-3 |  | 0.721 |  | 258.08 |  | 1.000 |  |  |  | N11(Deamidated): 100; C18(Dehydro): 100; C24(Dehydro): 100 |  |  |  |  |  |  |  | 0.60 |  | 1 |  | Sequest HT (3) |  | 4 |  | 662.88208 |  | -0.21 |  | 51.0618 |  | 15764 |  | 15764 |  | TRINITY\_DN0\_c1\_g1\_i24.p1\_ORF1; TRINITY\_DN0\_c1\_g1\_i24.p1; TRINITY\_DN0\_c1\_g1\_i6.p1 |  | Image |  | Peak List |  |
|  | | | | | | | | | | | | | | | | | | | | | | | | | | | | | | | | | | | | | | | | | | | | | | | | | | | | | |  |
|  | High |  | [R].FLPLLAGLAAnFLPKLFcKITKKc.[-] |  | HCD |  | N11(Deamidated); C18(Dehydro); C24(Dehydro) |  | 3.96 |  | 1.3e-3 |  | 5.6e-3 |  | 0.818 |  | 314.65 |  | 1.000 |  |  |  | N11(Deamidated): 100; C18(Dehydro): 100; C24(Dehydro): 100 |  |  |  |  |  |  |  | 0.61 |  | 1 |  | Sequest HT (3) |  | 3 |  | 883.50659 |  | -0.69 |  | 51.1171 |  | 15787 |  | 15787 |  | TRINITY\_DN0\_c1\_g1\_i24.p1\_ORF1; TRINITY\_DN0\_c1\_g1\_i24.p1; TRINITY\_DN0\_c1\_g1\_i6.p1 |  | Image |  | Peak List |  |
|  | | | | | | | | | | | | | | | | | | | | | | | | | | | | | | | | | | | | | | | | | | | | | | | | | | | | | |  |
|  | High |  | [R].FLPLLAGLAAnFLPKLFcKITKKc.[-] |  | HCD |  | N11(Deamidated); C18(Dehydro); C24(Dehydro) |  | 3.18 |  | 3.1e-3 |  | 1.4e-1 |  | 0.102 |  | 222.39 |  | 1.000 |  |  |  | N11(Deamidated): 100; C18(Dehydro): 100; C24(Dehydro): 100 |  |  |  |  |  |  |  | 0.56 |  | 1 |  | Sequest HT (3) |  | 5 |  | 530.50659 |  | -1.21 |  | 51.1758 |  | 15810 |  | 15810 |  | TRINITY\_DN0\_c1\_g1\_i24.p1\_ORF1; TRINITY\_DN0\_c1\_g1\_i24.p1; TRINITY\_DN0\_c1\_g1\_i6.p1 |  | Image |  | Peak List |  |
|  | | | | | | | | | | | | | | | | | | | | | | | | | | | | | | | | | | | | | | | | | | | | | | | | | | | | | |  |
|  | High |  | [R].FLPLLAGLAAnFLPKLFcKITKKc.[-] |  | HCD |  | N11(Deamidated); C18(Dehydro); C24(Dehydro) |  | 5.72 |  | 1.3e-3 |  | 6.2e-4 |  | 1.3 |  | 474.80 |  | 1.000 |  |  |  | N11(Deamidated): 100; C18(Dehydro): 100; C24(Dehydro): 100 |  |  |  |  |  |  |  | 0.70 |  | 1 |  | Sequest HT (3) |  | 2 |  | 1324.75415 |  | -2.28 |  | 51.1946 |  | 15818 |  | 15818 |  | TRINITY\_DN0\_c1\_g1\_i24.p1\_ORF1; TRINITY\_DN0\_c1\_g1\_i24.p1; TRINITY\_DN0\_c1\_g1\_i6.p1 |  | Image |  | Peak List |  |
|  | | | | | | | | | | | | | | | | | | | | | | | | | | | | | | | | | | | | | | | | | | | | | | | | | | | | | |  |
|  | High |  | [R].FLPLLAGLAAnFLPKLFcKITKKc.[-] |  | HCD |  | N11(Deamidated); C18(Dehydro); C24(Dehydro) |  | 3.62 |  | 1.3e-3 |  | 4.3e-2 |  | 0.37 |  | 247.97 |  | 1.000 |  |  |  | N11(Deamidated): 100; C18(Dehydro): 100; C24(Dehydro): 100 |  |  |  |  |  |  |  | 0.52 |  | 1 |  | Sequest HT (3) |  | 4 |  | 662.88165 |  | -0.86 |  | 51.2044 |  | 15822 |  | 15822 |  | TRINITY\_DN0\_c1\_g1\_i24.p1\_ORF1; TRINITY\_DN0\_c1\_g1\_i24.p1; TRINITY\_DN0\_c1\_g1\_i6.p1 |  | Image |  | Peak List |  |
|  | | | | | | | | | | | | | | | | | | | | | | | | | | | | | | | | | | | | | | | | | | | | | | | | | | | | | |  |
|  | High |  | [R].FLPLLAGLAAnFLPKLFcKITKKc.[-] |  | HCD |  | N11(Deamidated); C18(Dehydro); C24(Dehydro) |  | 4.75 |  | 1.3e-3 |  | 9.9e-4 |  | 1.2 |  | 375.08 |  | 1.000 |  |  |  | N11(Deamidated): 100; C18(Dehydro): 100; C24(Dehydro): 100 |  |  |  |  |  |  |  | 0.63 |  | 1 |  | Sequest HT (3) |  | 3 |  | 883.50586 |  | -1.52 |  | 51.2583 |  | 15842 |  | 15842 |  | TRINITY\_DN0\_c1\_g1\_i24.p1\_ORF1; TRINITY\_DN0\_c1\_g1\_i24.p1; TRINITY\_DN0\_c1\_g1\_i6.p1 |  | Image |  | Peak List |  |
|  | | | | | | | | | | | | | | | | | | | | | | | | | | | | | | | | | | | | | | | | | | | | | | | | | | | | | |  |
|  | High |  | [R].FLPLLAGLAAnFLPKLFcKITKKc.[-] |  | HCD |  | N11(Deamidated); C18(Dehydro); C24(Dehydro) |  | 3.14 |  | 1.3e-3 |  | 2.6e-2 |  | 0.48 |  | 206.49 |  | 1.000 |  |  |  | N11(Deamidated): 100; C18(Dehydro): 100; C24(Dehydro): 100 |  |  |  |  |  |  |  | 0.57 |  | 1 |  | Sequest HT (3) |  | 5 |  | 530.50635 |  | -1.67 |  | 51.3213 |  | 15867 |  | 15867 |  | TRINITY\_DN0\_c1\_g1\_i24.p1\_ORF1; TRINITY\_DN0\_c1\_g1\_i24.p1; TRINITY\_DN0\_c1\_g1\_i6.p1 |  | Image |  | Peak List |  |
|  | | | | | | | | | | | | | | | | | | | | | | | | | | | | | | | | | | | | | | | | | | | | | | | | | | | | | |  |
|  | High |  | [R].FLPLLAGLAAnFLPKLFcKITKKc.[-] |  | HCD |  | N11(Deamidated); C18(Dehydro); C24(Dehydro) |  | 3.88 |  | 1.3e-3 |  | 8.0e-4 |  | 1.24 |  | 438.32 |  | 1.000 |  |  |  | N11(Deamidated): 100; C18(Dehydro): 100; C24(Dehydro): 100 |  |  |  |  |  |  |  | 0.65 |  | 1 |  | Sequest HT (3) |  | 2 |  | 1324.75891 |  | 1.32 |  | 51.3405 |  | 15876 |  | 15876 |  | TRINITY\_DN0\_c1\_g1\_i24.p1\_ORF1; TRINITY\_DN0\_c1\_g1\_i24.p1; TRINITY\_DN0\_c1\_g1\_i6.p1 |  | Image |  | Peak List |  |
|  | | | | | | | | | | | | | | | | | | | | | | | | | | | | | | | | | | | | | | | | | | | | | | | | | | | | | |  |
|  | High |  | [R].FLPLLAGLAAnFLPKLFcKITKKc.[-] |  | HCD |  | N11(Deamidated); C18(Dehydro); C24(Dehydro) |  | 2.78 |  | 2.4e-3 |  | 8.5e-2 |  | 0.219 |  | 248.44 |  | 1.000 |  |  |  | N11(Deamidated): 100; C18(Dehydro): 100; C24(Dehydro): 100 |  |  |  |  |  |  |  | 0.32 |  | 1 |  | Sequest HT (3) |  | 4 |  | 662.88159 |  | -0.95 |  | 51.3469 |  | 15878 |  | 15878 |  | TRINITY\_DN0\_c1\_g1\_i24.p1\_ORF1; TRINITY\_DN0\_c1\_g1\_i24.p1; TRINITY\_DN0\_c1\_g1\_i6.p1 |  | Image |  | Peak List |  |
|  | | | | | | | | | | | | | | | | | | | | | | | | | | | | | | | | | | | | | | | | | | | | | | | | | | | | | |  |
|  | High |  | [R].FLPLLAGLAAnFLPKLFcKITKKc.[-] |  | HCD |  | N11(Deamidated); C18(Dehydro); C24(Dehydro) |  | 4.73 |  | 1.3e-3 |  | 6.0e-3 |  | 0.802 |  | 353.64 |  | 1.000 |  |  |  | N11(Deamidated): 100; C18(Dehydro): 100; C24(Dehydro): 100 |  |  |  |  |  |  |  | 0.65 |  | 1 |  | Sequest HT (3) |  | 3 |  | 883.50671 |  | -0.55 |  | 51.4060 |  | 15903 |  | 15903 |  | TRINITY\_DN0\_c1\_g1\_i24.p1\_ORF1; TRINITY\_DN0\_c1\_g1\_i24.p1; TRINITY\_DN0\_c1\_g1\_i6.p1 |  | Image |  | Peak List |  |
|  | | | | | | | | | | | | | | | | | | | | | | | | | | | | | | | | | | | | | | | | | | | | | | | | | | | | | |  |
|  | High |  | [R].FLPLLAGLAAnFLPKLFcKITKKc.[-] |  | HCD |  | N11(Deamidated); C18(Dehydro); C24(Dehydro) |  | 3.17 |  | 3.1e-3 |  | 1.0e-1 |  | 0.176 |  | 237.10 |  | 1.000 |  |  |  | N11(Deamidated): 100; C18(Dehydro): 100; C24(Dehydro): 100 |  |  |  |  |  |  |  | 0.49 |  | 1 |  | Sequest HT (3) |  | 4 |  | 662.88171 |  | -0.77 |  | 51.4970 |  | 15939 |  | 15939 |  | TRINITY\_DN0\_c1\_g1\_i24.p1\_ORF1; TRINITY\_DN0\_c1\_g1\_i24.p1; TRINITY\_DN0\_c1\_g1\_i6.p1 |  | Image |  | Peak List |  |
|  | | | | | | | | | | | | | | | | | | | | | | | | | | | | | | | | | | | | | | | | | | | | | | | | | | | | | |  |
|  | High |  | [R].FLPLLAGLAANFLPKLFcKITKKc.[-] |  | HCD |  | C18(Dehydro); C24(Dehydro) |  | 3.37 |  | 2.4e-3 |  | 7.7e-2 |  | 0.241 |  | 224.88 |  | 1.000 |  |  |  | C18(Dehydro): 100; C24(Dehydro): 100 |  |  |  |  |  |  |  | 0.41 |  | 1 |  | Sequest HT (3) |  | 4 |  | 662.63617 |  | -0.07 |  | 52.9647 |  | 16513 |  | 16513 |  | TRINITY\_DN0\_c1\_g1\_i24.p1\_ORF1; TRINITY\_DN0\_c1\_g1\_i24.p1; TRINITY\_DN0\_c1\_g1\_i6.p1 |  | Image |  | Peak List |  |
|  | | | | | | | | | | | | | | | | | | | | | | | | | | | | | | | | | | | | | | | | | | | | | | | | | | | | | |  |
|  | High |  | [R].FLPLLAGLAANFLPKLFcKITKKc.[-] |  | HCD |  | C18(Dehydro); C24(Dehydro) |  | 3.06 |  | 3.9e-3 |  | 1.6e-1 |  | 0.072 |  | 207.01 |  | 1.000 |  |  |  | C18(Dehydro): 100; C24(Dehydro): 100 |  |  |  |  |  |  |  | 0.49 |  | 1 |  | Sequest HT (3) |  | 4 |  | 662.63470 |  | -2.29 |  | 53.1051 |  | 16560 |  | 16560 |  | TRINITY\_DN0\_c1\_g1\_i24.p1\_ORF1; TRINITY\_DN0\_c1\_g1\_i24.p1; TRINITY\_DN0\_c1\_g1\_i6.p1 |  | Image |  | Peak List |  |
|  | | | | | | | | | | | | | | | | | | | | | | | | | | | | | | | | | | | | | | | | | | | | | | | | | | | | | |  |
|  | High |  | [R].FLPLLAGLAANFLPKLFcKITKKc.[-] |  | HCD |  | C18(Dehydro); C24(Dehydro) |  | 1.54 |  | 6.5e-3 |  | 2.1e-1 |  | 0.006 |  | 134.25 |  | 1.000 |  |  |  | C18(Dehydro): 100; C24(Dehydro): 100 |  |  |  |  |  |  |  | 0.32 |  | 1 |  | Sequest HT (3) |  | 5 |  | 530.31012 |  | -0.58 |  | 59.2993 |  | 18591 |  | 18591 |  | TRINITY\_DN0\_c1\_g1\_i24.p1\_ORF1; TRINITY\_DN0\_c1\_g1\_i24.p1; TRINITY\_DN0\_c1\_g1\_i6.p1 |  | Image |  | Peak List |  |
|  | | | | | | | | | | | | | | | | | | | | | | | | | | | | | | | | | | | | | | | | | | | | | | | | | | | | | |  |
|  | **TRINITY\_DN0\_c1\_g1\_i16.p1 - TRINITY\_DN0\_c1\_g1\_i16.p1** | | | | | | | | | | | | | | | | | | | | | | | | | | | | | | | | | | | | | | | | | | | | | | | | | | | |  |  |
|  | | | | | | | | | | | | | | | | | | | | | | | | | | | | | | | | | | | | | | | | | | | | | | | | | | | | | |  |
|  | High |  | [R].GLFSKFAGKGIKD.[L] |  | HCD |  |  |  | 2.62 |  | 1.3e-3 |  | 5.7e-2 |  | 0.31 |  |  |  |  |  |  |  |  |  |  |  |  |  |  |  | 0.00 |  | 1 |  | Sequest HT (3) |  | 3 |  | 456.59424 |  | 0.00 |  | 21.4968 |  | 4038 |  | 4038 |  | TRINITY\_DN0\_c1\_g1\_i16.p1; TRINITY\_DN0\_c1\_g1\_i16.p1\_ORF1; TRINITY\_DN81\_c0\_g1\_i1.p1\_ORF2; TRINITY\_DN12814\_c1\_g1\_i1.p1; TRINITY\_DN45\_c31\_g1\_i1.p1; TRINITY\_DN0\_c1\_g1\_i18.p1 |  | Image |  | Peak List |  |
|  | | | | | | | | | | | | | | | | | | | | | | | | | | | | | | | | | | | | | | | | | | | | | | | | | | | | | |  |
|  | High |  | [R].GLFSKFAGK.[GV] |  | HCD |  |  |  | 1.47 |  | 2.4e-3 |  | 8.5e-2 |  | 0.218 |  |  |  |  |  |  |  |  |  |  |  |  |  |  |  | 0.37 |  | 1 |  | Sequest HT (3) |  | 2 |  | 477.77396 |  | -0.10 |  | 21.7911 |  | 4105 |  | 4105 |  | TRINITY\_DN8697\_c0\_g1\_i1.p1; TRINITY\_DN259\_c0\_g1\_i1.p1\_ORF1; TRINITY\_DN21\_c2\_g1\_i1.p1; TRINITY\_DN12856\_c2\_g1\_i1.p1\_ORF1; TRINITY\_DN8697\_c0\_g1\_i2.p1\_ORF1; TRINITY\_DN750\_c3\_g1\_i1.p1; TRINITY\_DN12856\_c2\_g1\_i1.p1; TRINITY\_DN0\_c1\_g1\_i16.p1; TRINITY\_DN8697\_c0\_g1\_i1.p1\_ORF1; TRINITY\_DN0\_c1\_g1\_i16.p1\_ORF1; TRINITY\_DN81\_c0\_g1\_i1.p1\_ORF2; TRINITY\_DN12814\_c1\_g1\_i1.p1; TRINITY\_DN45\_c31\_g1\_i1.p1; TRINITY\_DN0\_c1\_g1\_i18.p1; TRINITY\_DN8697\_c0\_g1\_i2.p1 |  | Image |  | Peak List |  |
|  | | | | | | | | | | | | | | | | | | | | | | | | | | | | | | | | | | | | | | | | | | | | | | | | | | | | | |  |
|  | High |  | [G].MDVIRTGIDVAGcKIKGEc.[-] |  | HCD |  | C13(Dehydro); C19(Dehydro) |  | 3.32 |  | 1.3e-3 |  | 2.2e-2 |  | 0.519 |  | 249.76 |  | 1.000 |  |  |  | C13(Dehydro): 100; C19(Dehydro): 100 |  |  |  |  |  |  |  | 0.46 |  | 1 |  | Sequest HT (3) |  | 3 |  | 669.33502 |  | 1.87 |  | 29.7293 |  | 6439 |  | 6439 |  | TRINITY\_DN0\_c1\_g1\_i16.p1; TRINITY\_DN0\_c1\_g1\_i16.p1\_ORF1; TRINITY\_DN81\_c0\_g1\_i1.p1\_ORF2; TRINITY\_DN836\_c5\_g1\_i1.p1; TRINITY\_DN0\_c1\_g1\_i18.p1 |  | Image |  | Peak List |  |
|  | | | | | | | | | | | | | | | | | | | | | | | | | | | | | | | | | | | | | | | | | | | | | | | | | | | | | |  |
|  | High |  | [G].MDVIRTGIDVAGcKIKGEc.[-] |  | HCD |  | C13(Dehydro); C19(Dehydro) |  | 2.79 |  | 1.3e-3 |  | 6.3e-2 |  | 0.287 |  | 188.93 |  | 1.000 |  |  |  | C13(Dehydro): 100; C19(Dehydro): 100 |  |  |  |  |  |  |  | 0.49 |  | 1 |  | Sequest HT (3) |  | 3 |  | 669.33368 |  | -0.14 |  | 29.8867 |  | 6498 |  | 6498 |  | TRINITY\_DN0\_c1\_g1\_i16.p1; TRINITY\_DN0\_c1\_g1\_i16.p1\_ORF1; TRINITY\_DN81\_c0\_g1\_i1.p1\_ORF2; TRINITY\_DN836\_c5\_g1\_i1.p1; TRINITY\_DN0\_c1\_g1\_i18.p1 |  | Image |  | Peak List |  |
|  | | | | | | | | | | | | | | | | | | | | | | | | | | | | | | | | | | | | | | | | | | | | | | | | | | | | | |  |
|  | High |  | [D].LIFKGVKHIGKEVGmDVIRTGIDVAGcKIKGEc.[-] |  | HCD |  | M15(Oxidation); C27(Dehydro); C33(Dehydro) |  | 3.79 |  | 1.3e-3 |  | 4.9e-2 |  | 0.343 |  | 64.57 |  | 1.000 |  |  |  | M15(Oxidation): 100; C27(Dehydro): 100; C33(Dehydro): 100 |  |  |  |  |  |  |  | 0.55 |  | 1 |  | Sequest HT (3) |  | 5 |  | 706.38336 |  | -0.62 |  | 31.0200 |  | 6936 |  | 6936 |  | TRINITY\_DN0\_c1\_g1\_i16.p1; TRINITY\_DN0\_c1\_g1\_i16.p1\_ORF1; TRINITY\_DN81\_c0\_g1\_i1.p1\_ORF2; TRINITY\_DN0\_c1\_g1\_i18.p1 |  | Image |  | Peak List |  |
|  | | | | | | | | | | | | | | | | | | | | | | | | | | | | | | | | | | | | | | | | | | | | | | | | | | | | | |  |
|  | High |  | [R].GLFSKFAGKGIKDLIFKGVK.[H] |  | HCD |  |  |  | 4.81 |  | 1.3e-3 |  | 2.7e-3 |  | 0.975 |  |  |  |  |  |  |  |  |  |  |  |  |  |  |  | 0.09 |  | 1 |  | Sequest HT (3) |  | 3 |  | 718.43292 |  | -0.14 |  | 31.0324 |  | 6941 |  | 6941 |  | TRINITY\_DN0\_c1\_g1\_i16.p1; TRINITY\_DN0\_c1\_g1\_i16.p1\_ORF1; TRINITY\_DN81\_c0\_g1\_i1.p1\_ORF2; TRINITY\_DN0\_c1\_g1\_i18.p1 |  | Image |  | Peak List |  |
|  | | | | | | | | | | | | | | | | | | | | | | | | | | | | | | | | | | | | | | | | | | | | | | | | | | | | | |  |
|  | High |  | [R].GLFSKFAGKGIKDLIFKGVK.[H] |  | HCD |  |  |  | 3.53 |  | 1.3e-3 |  | 5.8e-3 |  | 0.809 |  |  |  |  |  |  |  |  |  |  |  |  |  |  |  | 0.12 |  | 1 |  | Sequest HT (3) |  | 5 |  | 431.46320 |  | 1.10 |  | 31.0676 |  | 6957 |  | 6957 |  | TRINITY\_DN0\_c1\_g1\_i16.p1; TRINITY\_DN0\_c1\_g1\_i16.p1\_ORF1; TRINITY\_DN81\_c0\_g1\_i1.p1\_ORF2; TRINITY\_DN0\_c1\_g1\_i18.p1 |  | Image |  | Peak List |  |
|  | | | | | | | | | | | | | | | | | | | | | | | | | | | | | | | | | | | | | | | | | | | | | | | | | | | | | |  |
|  | High |  | [R].GLFSKFAGKGIKDLIFKGVKHIG.[K] |  | HCD |  |  |  | 3.49 |  | 1.3e-3 |  | 3.7e-2 |  | 0.402 |  |  |  |  |  |  |  |  |  |  |  |  |  |  |  | 0.10 |  | 1 |  | Sequest HT (3) |  | 5 |  | 492.89606 |  | 0.91 |  | 31.2328 |  | 7024 |  | 7024 |  | TRINITY\_DN0\_c1\_g1\_i16.p1; TRINITY\_DN0\_c1\_g1\_i16.p1\_ORF1; TRINITY\_DN81\_c0\_g1\_i1.p1\_ORF2; TRINITY\_DN0\_c1\_g1\_i18.p1 |  | Image |  | Peak List |  |
|  | | | | | | | | | | | | | | | | | | | | | | | | | | | | | | | | | | | | | | | | | | | | | | | | | | | | | |  |
|  | High |  | [R].GLFSKFAGKGIKDLIFKGVKHIG.[K] |  | HCD |  |  |  | 3.95 |  | 1.3e-3 |  | 6.4e-2 |  | 0.282 |  |  |  |  |  |  |  |  |  |  |  |  |  |  |  | 0.06 |  | 1 |  | Sequest HT (3) |  | 4 |  | 615.86761 |  | -0.13 |  | 31.2596 |  | 7035 |  | 7035 |  | TRINITY\_DN0\_c1\_g1\_i16.p1; TRINITY\_DN0\_c1\_g1\_i16.p1\_ORF1; TRINITY\_DN81\_c0\_g1\_i1.p1\_ORF2; TRINITY\_DN0\_c1\_g1\_i18.p1 |  | Image |  | Peak List |  |
|  | | | | | | | | | | | | | | | | | | | | | | | | | | | | | | | | | | | | | | | | | | | | | | | | | | | | | |  |
|  | High |  | [R].GLFSKFAGKGIKDLIFK.[G] |  | HCD |  |  |  | 2.84 |  | 1.3e-3 |  | 1.5e-2 |  | 0.599 |  |  |  |  |  |  |  |  |  |  |  |  |  |  |  | 0.17 |  | 1 |  | Sequest HT (3) |  | 4 |  | 468.03030 |  | -0.15 |  | 31.8684 |  | 7278 |  | 7278 |  | TRINITY\_DN0\_c1\_g1\_i16.p1; TRINITY\_DN0\_c1\_g1\_i16.p1\_ORF1; TRINITY\_DN81\_c0\_g1\_i1.p1\_ORF2; TRINITY\_DN0\_c1\_g1\_i18.p1 |  | Image |  | Peak List |  |
|  | | | | | | | | | | | | | | | | | | | | | | | | | | | | | | | | | | | | | | | | | | | | | | | | | | | | | |  |
|  | High |  | [R].GLFSKFAGKGIKDLIFKG.[V] |  | HCD |  |  |  | 4.28 |  | 1.3e-3 |  | 1.3e-3 |  | 1.13 |  |  |  |  |  |  |  |  |  |  |  |  |  |  |  | 0.21 |  | 1 |  | Sequest HT (3) |  | 3 |  | 642.71222 |  | 0.51 |  | 31.9594 |  | 7314 |  | 7314 |  | TRINITY\_DN0\_c1\_g1\_i16.p1; TRINITY\_DN0\_c1\_g1\_i16.p1\_ORF1; TRINITY\_DN81\_c0\_g1\_i1.p1\_ORF2; TRINITY\_DN0\_c1\_g1\_i18.p1 |  | Image |  | Peak List |  |
|  | | | | | | | | | | | | | | | | | | | | | | | | | | | | | | | | | | | | | | | | | | | | | | | | | | | | | |  |
|  | High |  | [R].GLFSKFAGKGIKDLIFKG.[V] |  | HCD |  |  |  | 3.83 |  | 1.3e-3 |  | 2.6e-2 |  | 0.481 |  |  |  |  |  |  |  |  |  |  |  |  |  |  |  | 0.16 |  | 1 |  | Sequest HT (3) |  | 4 |  | 482.28589 |  | 0.31 |  | 31.9645 |  | 7316 |  | 7316 |  | TRINITY\_DN0\_c1\_g1\_i16.p1; TRINITY\_DN0\_c1\_g1\_i16.p1\_ORF1; TRINITY\_DN81\_c0\_g1\_i1.p1\_ORF2; TRINITY\_DN0\_c1\_g1\_i18.p1 |  | Image |  | Peak List |  |
|  | | | | | | | | | | | | | | | | | | | | | | | | | | | | | | | | | | | | | | | | | | | | | | | | | | | | | |  |
|  | High |  | [D].LIFKGVKHIGKEVGMDVIRTGIDVAGcKIKGEc.[-] |  | HCD |  | C27(Dehydro); C33(Dehydro) |  | 3.45 |  | 1.3e-3 |  | 1.4e-2 |  | 0.614 |  | 134.17 |  | 1.000 |  |  |  | C27(Dehydro): 100; C33(Dehydro): 100 |  |  |  |  |  |  |  | 0.54 |  | 1 |  | Sequest HT (3) |  | 5 |  | 703.18469 |  | -0.18 |  | 32.7971 |  | 7673 |  | 7673 |  | TRINITY\_DN0\_c1\_g1\_i16.p1; TRINITY\_DN0\_c1\_g1\_i16.p1\_ORF1; TRINITY\_DN81\_c0\_g1\_i1.p1\_ORF2; TRINITY\_DN0\_c1\_g1\_i18.p1 |  | Image |  | Peak List |  |
|  | | | | | | | | | | | | | | | | | | | | | | | | | | | | | | | | | | | | | | | | | | | | | | | | | | | | | |  |
|  | High |  | [D].LIFKGVKHIGKEVGMDVIRTGIDVAGcKIKGEc.[-] |  | HCD |  | C27(Dehydro); C33(Dehydro) |  | 1.33 |  | 3.9e-3 |  | 1.8e-1 |  | 0.044 |  | 88.98 |  | 1.000 |  |  |  | C27(Dehydro): 100; C33(Dehydro): 100 |  |  |  |  |  |  |  | 0.42 |  | 1 |  | Sequest HT (3) |  | 4 |  | 878.73071 |  | 1.72 |  | 32.8087 |  | 7678 |  | 7678 |  | TRINITY\_DN0\_c1\_g1\_i16.p1; TRINITY\_DN0\_c1\_g1\_i16.p1\_ORF1; TRINITY\_DN81\_c0\_g1\_i1.p1\_ORF2; TRINITY\_DN0\_c1\_g1\_i18.p1 |  | Image |  | Peak List |  |
|  | | | | | | | | | | | | | | | | | | | | | | | | | | | | | | | | | | | | | | | | | | | | | | | | | | | | | |  |
|  | High |  | [D].LIFKGVKHIGKEVGMDVIRTGIDVAGcKIKGEc.[-] |  | HCD |  | C27(Dehydro); C33(Dehydro) |  | 3.33 |  | 3.1e-3 |  | 9.2e-2 |  | 0.202 |  | 102.98 |  | 1.000 |  |  |  | C27(Dehydro): 100; C33(Dehydro): 100 |  |  |  |  |  |  |  | 0.60 |  | 1 |  | Sequest HT (3) |  | 5 |  | 703.18591 |  | 1.56 |  | 32.9369 |  | 7728 |  | 7728 |  | TRINITY\_DN0\_c1\_g1\_i16.p1; TRINITY\_DN0\_c1\_g1\_i16.p1\_ORF1; TRINITY\_DN81\_c0\_g1\_i1.p1\_ORF2; TRINITY\_DN0\_c1\_g1\_i18.p1 |  | Image |  | Peak List |  |
|  | | | | | | | | | | | | | | | | | | | | | | | | | | | | | | | | | | | | | | | | | | | | | | | | | | | | | |  |
|  | High |  | [D].LIFKGVKHIGKEVGMDVIRTGIDVAGcKIKGEc.[-] |  | HCD |  | C27(Dehydro); C33(Dehydro) |  | 2.88 |  | 1.3e-3 |  | 4.8e-2 |  | 0.345 |  | 98.54 |  | 1.000 |  |  |  | C27(Dehydro): 100; C33(Dehydro): 100 |  |  |  |  |  |  |  | 0.66 |  | 1 |  | Sequest HT (3) |  | 4 |  | 878.72919 |  | -0.02 |  | 32.9647 |  | 7741 |  | 7741 |  | TRINITY\_DN0\_c1\_g1\_i16.p1; TRINITY\_DN0\_c1\_g1\_i16.p1\_ORF1; TRINITY\_DN81\_c0\_g1\_i1.p1\_ORF2; TRINITY\_DN0\_c1\_g1\_i18.p1 |  | Image |  | Peak List |  |
|  | | | | | | | | | | | | | | | | | | | | | | | | | | | | | | | | | | | | | | | | | | | | | | | | | | | | | |  |
|  | High |  | [R].GLFSKFAGKGIKDLIFKGVKHIGKEVGMD.[V] |  | HCD |  |  |  | 2.74 |  | 6.5e-3 |  | 2.1e-1 |  | 0.004 |  |  |  |  |  |  |  |  |  |  |  |  |  |  |  | 0.35 |  | 1 |  | Sequest HT (3) |  | 5 |  | 624.75531 |  | 1.17 |  | 34.2440 |  | 8298 |  | 8298 |  | TRINITY\_DN0\_c1\_g1\_i16.p1; TRINITY\_DN0\_c1\_g1\_i16.p1\_ORF1; TRINITY\_DN81\_c0\_g1\_i1.p1\_ORF2; TRINITY\_DN0\_c1\_g1\_i18.p1 |  | Image |  | Peak List |  |
|  | | | | | | | | | | | | | | | | | | | | | | | | | | | | | | | | | | | | | | | | | | | | | | | | | | | | | |  |
|  | High |  | [R].GLFSKFAGKGIKDLIFKGVKHIGKEVGMD.[V] |  | HCD |  |  |  | 6.09 |  | 1.3e-3 |  | 8.5e-4 |  | 1.23 |  |  |  |  |  |  |  |  |  |  |  |  |  |  |  | 0.66 |  | 1 |  | Sequest HT (3) |  | 4 |  | 780.69086 |  | -0.71 |  | 34.2902 |  | 8318 |  | 8318 |  | TRINITY\_DN0\_c1\_g1\_i16.p1; TRINITY\_DN0\_c1\_g1\_i16.p1\_ORF1; TRINITY\_DN81\_c0\_g1\_i1.p1\_ORF2; TRINITY\_DN0\_c1\_g1\_i18.p1 |  | Image |  | Peak List |  |
|  | | | | | | | | | | | | | | | | | | | | | | | | | | | | | | | | | | | | | | | | | | | | | | | | | | | | | |  |
|  | High |  | [R].GLFSKFAGKGIKDLIFKGVKHIGKEVGMD.[V] |  | HCD |  |  |  | 3.08 |  | 1.3e-3 |  | 4.1e-4 |  | 1.39 |  |  |  |  |  |  |  |  |  |  |  |  |  |  |  | 0.59 |  | 1 |  | Sequest HT (3) |  | 3 |  | 1040.58679 |  | 0.65 |  | 34.3004 |  | 8323 |  | 8323 |  | TRINITY\_DN0\_c1\_g1\_i16.p1; TRINITY\_DN0\_c1\_g1\_i16.p1\_ORF1; TRINITY\_DN81\_c0\_g1\_i1.p1\_ORF2; TRINITY\_DN0\_c1\_g1\_i18.p1 |  | Image |  | Peak List |  |
|  | | | | | | | | | | | | | | | | | | | | | | | | | | | | | | | | | | | | | | | | | | | | | | | | | | | | | |  |
|  | High |  | [R].GLFSKFAGKGIKDLIFKGVKHIGKEVGMDVIRTGID.[V] |  | HCD |  |  |  | 4.40 |  | 1.3e-3 |  | 4.9e-3 |  | 0.845 |  |  |  |  |  |  |  |  |  |  |  |  |  |  |  | 0.62 |  | 1 |  | Sequest HT (3) |  | 5 |  | 775.64008 |  | -1.62 |  | 35.8025 |  | 9014 |  | 9014 |  | TRINITY\_DN0\_c1\_g1\_i16.p1; TRINITY\_DN0\_c1\_g1\_i16.p1\_ORF1; TRINITY\_DN81\_c0\_g1\_i1.p1\_ORF2; TRINITY\_DN0\_c1\_g1\_i18.p1 |  | Image |  | Peak List |  |
|  | | | | | | | | | | | | | | | | | | | | | | | | | | | | | | | | | | | | | | | | | | | | | | | | | | | | | |  |
|  | High |  | [R].GLFSKFAGKGIKDLIFKGVKHIGKEVGMDVIRTGIDVAGcKIKGEc.[-] |  | HCD |  | C40(Dehydro); C46(Dehydro) |  | 3.71 |  | 1.3e-3 |  | 5.3e-2 |  | 0.326 |  | 96.01 |  | 1.000 |  |  |  | C40(Dehydro): 100; C46(Dehydro): 100 |  |  |  |  |  |  |  | 0.64 |  | 1 |  | Sequest HT (3) |  | 5 |  | 972.93506 |  | 0.18 |  | 38.0497 |  | 10100 |  | 10100 |  | TRINITY\_DN0\_c1\_g1\_i16.p1; TRINITY\_DN0\_c1\_g1\_i16.p1\_ORF1; TRINITY\_DN81\_c0\_g1\_i1.p1\_ORF2; TRINITY\_DN0\_c1\_g1\_i18.p1 |  | Image |  | Peak List |  |
|  | | | | | | | | | | | | | | | | | | | | | | | | | | | | | | | | | | | | | | | | | | | | | | | | | | | | | |  |
|  | **TRINITY\_DN5345\_c0\_g1\_i2.p1 - TRINITY\_DN5345\_c0\_g1\_i2.p1** | | | | | | | | | | | | | | | | | | | | | | | | | | | | | | | | | | | | | | | | | | | | | | | | | | | |  |  |
|  | | | | | | | | | | | | | | | | | | | | | | | | | | | | | | | | | | | | | | | | | | | | | | | | | | | | | |  |
|  | High |  | [R].VIPFVASVAAEmmQHVYcAASKKc.[-] |  | HCD |  | M12(Oxidation); M13(Oxidation); C18(Dehydro); C24(Dehydro) |  | 2.07 |  | 1.3e-3 |  | 5.8e-2 |  | 0.303 |  | 197.95 |  | 1.000 |  |  |  | M12(Oxidation): 100; M13(Oxidation): 100; C18(Dehydro): 100; C24(Dehydro): 100 |  |  |  |  |  |  |  | 0.39 |  | 1 |  | Sequest HT (3) |  | 3 |  | 871.74945 |  | 0.22 |  | 31.9569 |  | 7313 |  | 7313 |  | TRINITY\_DN5345\_c0\_g1\_i1.p1; TRINITY\_DN5345\_c0\_g1\_i2.p1\_ORF1; TRINITY\_DN5345\_c0\_g1\_i2.p1; TRINITY\_DN0\_c3\_g1\_i1.p2 |  | Image |  | Peak List |  |
|  | | | | | | | | | | | | | | | | | | | | | | | | | | | | | | | | | | | | | | | | | | | | | | | | | | | | | |  |
|  | High |  | [R].VIPFVASVAAEmmQHVYcAASKKc.[-] |  | HCD |  | M12(Oxidation); M13(Oxidation); C18(Dehydro); C24(Dehydro) |  | 3.95 |  | 1.3e-3 |  | 6.8e-2 |  | 0.268 |  | 262.77 |  | 1.000 |  |  |  | M12(Oxidation): 100; M13(Oxidation): 100; C18(Dehydro): 100; C24(Dehydro): 100 |  |  |  |  |  |  |  | 0.63 |  | 1 |  | Sequest HT (3) |  | 4 |  | 654.06360 |  | -0.25 |  | 32.0403 |  | 7346 |  | 7346 |  | TRINITY\_DN5345\_c0\_g1\_i1.p1; TRINITY\_DN5345\_c0\_g1\_i2.p1\_ORF1; TRINITY\_DN5345\_c0\_g1\_i2.p1; TRINITY\_DN0\_c3\_g1\_i1.p2 |  | Image |  | Peak List |  |
|  | | | | | | | | | | | | | | | | | | | | | | | | | | | | | | | | | | | | | | | | | | | | | | | | | | | | | |  |
|  | High |  | [R].VIPFVASVAAEmmQHVYcAASKKc.[-] |  | HCD |  | M12(Oxidation); M13(Oxidation); C18(Dehydro); C24(Dehydro) |  | 3.87 |  | 1.3e-3 |  | 1.1e-2 |  | 0.664 |  | 293.62 |  | 1.000 |  |  |  | M12(Oxidation): 100; M13(Oxidation): 100; C18(Dehydro): 100; C24(Dehydro): 100 |  |  |  |  |  |  |  | 0.59 |  | 1 |  | Sequest HT (3) |  | 4 |  | 654.06445 |  | 1.06 |  | 32.2208 |  | 7421 |  | 7421 |  | TRINITY\_DN5345\_c0\_g1\_i1.p1; TRINITY\_DN5345\_c0\_g1\_i2.p1\_ORF1; TRINITY\_DN5345\_c0\_g1\_i2.p1; TRINITY\_DN0\_c3\_g1\_i1.p2 |  | Image |  | Peak List |  |
|  | | | | | | | | | | | | | | | | | | | | | | | | | | | | | | | | | | | | | | | | | | | | | | | | | | | | | |  |
|  | High |  | [R].VIPFVASVAAEmmQHVYcAASKKc.[-] |  | HCD |  | M12(Oxidation); M13(Oxidation); C18(Dehydro); C24(Dehydro) |  | 4.06 |  | 3.1e-3 |  | 9.6e-2 |  | 0.192 |  | 249.90 |  | 1.000 |  |  |  | M12(Oxidation): 100; M13(Oxidation): 100; C18(Dehydro): 100; C24(Dehydro): 100 |  |  |  |  |  |  |  | 0.60 |  | 1 |  | Sequest HT (3) |  | 4 |  | 654.06342 |  | -0.53 |  | 32.6950 |  | 7627 |  | 7627 |  | TRINITY\_DN5345\_c0\_g1\_i1.p1; TRINITY\_DN5345\_c0\_g1\_i2.p1\_ORF1; TRINITY\_DN5345\_c0\_g1\_i2.p1; TRINITY\_DN0\_c3\_g1\_i1.p2 |  | Image |  | Peak List |  |
|  | | | | | | | | | | | | | | | | | | | | | | | | | | | | | | | | | | | | | | | | | | | | | | | | | | | | | |  |
|  | High |  | [R].VIPFVASVAAEmmQHVYcAASKKc.[-] |  | HCD |  | M12(Oxidation); M13(Oxidation); C18(Dehydro); C24(Dehydro) |  | 3.70 |  | 1.3e-3 |  | 1.8e-2 |  | 0.562 |  | 255.78 |  | 1.000 |  |  |  | M12(Oxidation): 100; M13(Oxidation): 100; C18(Dehydro): 100; C24(Dehydro): 100 |  |  |  |  |  |  |  | 0.61 |  | 1 |  | Sequest HT (3) |  | 3 |  | 871.74982 |  | 0.64 |  | 32.7052 |  | 7632 |  | 7632 |  | TRINITY\_DN5345\_c0\_g1\_i1.p1; TRINITY\_DN5345\_c0\_g1\_i2.p1\_ORF1; TRINITY\_DN5345\_c0\_g1\_i2.p1; TRINITY\_DN0\_c3\_g1\_i1.p2 |  | Image |  | Peak List |  |
|  | | | | | | | | | | | | | | | | | | | | | | | | | | | | | | | | | | | | | | | | | | | | | | | | | | | | | |  |
|  | High |  | [R].VIPFVASVAAEmmQHVYcAASKKc.[-] |  | HCD |  | M12(Oxidation); M13(Oxidation); C18(Dehydro); C24(Dehydro) |  | 2.39 |  | 5.6e-3 |  | 2.1e-1 |  | 0.014 |  | 143.72 |  | 1.000 |  |  |  | M12(Oxidation): 100; M13(Oxidation): 100; C18(Dehydro): 100; C24(Dehydro): 100 |  |  |  |  |  |  |  | 0.44 |  | 1 |  | Sequest HT (3) |  | 4 |  | 654.06335 |  | -0.62 |  | 32.8317 |  | 7686 |  | 7686 |  | TRINITY\_DN5345\_c0\_g1\_i1.p1; TRINITY\_DN5345\_c0\_g1\_i2.p1\_ORF1; TRINITY\_DN5345\_c0\_g1\_i2.p1; TRINITY\_DN0\_c3\_g1\_i1.p2 |  | Image |  | Peak List |  |
|  | | | | | | | | | | | | | | | | | | | | | | | | | | | | | | | | | | | | | | | | | | | | | | | | | | | | | |  |
|  | High |  | [R].VIPFVASVAAEmmQHVYcAASKKc.[-] |  | HCD |  | M12(Oxidation); M13(Oxidation); C18(Dehydro); C24(Dehydro) |  | 2.63 |  | 1.3e-3 |  | 1.1e-2 |  | 0.664 |  | 305.37 |  | 1.000 |  |  |  | M12(Oxidation): 100; M13(Oxidation): 100; C18(Dehydro): 100; C24(Dehydro): 100 |  |  |  |  |  |  |  | 0.44 |  | 1 |  | Sequest HT (3) |  | 3 |  | 871.74963 |  | 0.43 |  | 32.9151 |  | 7720 |  | 7720 |  | TRINITY\_DN5345\_c0\_g1\_i1.p1; TRINITY\_DN5345\_c0\_g1\_i2.p1\_ORF1; TRINITY\_DN5345\_c0\_g1\_i2.p1; TRINITY\_DN0\_c3\_g1\_i1.p2 |  | Image |  | Peak List |  |
|  | | | | | | | | | | | | | | | | | | | | | | | | | | | | | | | | | | | | | | | | | | | | | | | | | | | | | |  |
|  | High |  | [R].VIPFVASVAAEmMQHVYcAASKKc.[-] |  | HCD |  | M12(Oxidation); C18(Dehydro); C24(Dehydro) |  | 4.28 |  | 1.3e-3 |  | 1.9e-2 |  | 0.555 |  | 296.00 |  | 0.999 |  | y1+-H(4) C O S(16): 1720.76; y1+-H(4) C O S(15): 1649.72; y1+-H(4) C O S(14): 1578.69; b2+-H(4) C O S(12): 584.32; y2+-H(4) C O S(22): 1161.05; y1+(12): 1366.61 |  | M12(Oxidation): 99.95; C18(Dehydro): 100; C24(Dehydro): 100 |  |  |  |  |  |  |  | 0.06 |  | 1 |  | Sequest HT (3) |  | 3 |  | 866.41718 |  | -0.51 |  | 39.2427 |  | 10700 |  | 10700 |  | TRINITY\_DN5345\_c0\_g1\_i1.p1; TRINITY\_DN5345\_c0\_g1\_i2.p1\_ORF1; TRINITY\_DN5345\_c0\_g1\_i2.p1; TRINITY\_DN0\_c3\_g1\_i1.p2 |  | Image |  | Peak List |  |
|  | | | | | | | | | | | | | | | | | | | | | | | | | | | | | | | | | | | | | | | | | | | | | | | | | | | | | |  |
|  | High |  | [R].VIPFVASVAAEmMQHVYcAASKKc.[-] |  | HCD |  | M12(Oxidation); C18(Dehydro); C24(Dehydro) |  | 3.77 |  | 1.3e-3 |  | 1.8e-2 |  | 0.557 |  | 309.87 |  | 1.000 |  | y1+-H(4) C O S(16): 1720.76; y1+-H(4) C O S(15): 1649.72; y1+-H(4) C O S(14): 1578.69; y1+-H(4) C O S(13): 1449.64; b2+-H(4) C O S(12): 584.32; y2+-H(4) C O S(22): 1161.05; y2+-H(4) C O S(19): 989.45; y2+-H(4) C O S(15): 825.37; y1+(12): 1366.61 |  | M12(Oxidation): 99.96; C18(Dehydro): 100; C24(Dehydro): 100 |  |  |  |  |  |  |  | 0.09 |  | 1 |  | Sequest HT (3) |  | 3 |  | 866.41711 |  | -0.58 |  | 39.3807 |  | 10763 |  | 10763 |  | TRINITY\_DN5345\_c0\_g1\_i1.p1; TRINITY\_DN5345\_c0\_g1\_i2.p1\_ORF1; TRINITY\_DN5345\_c0\_g1\_i2.p1; TRINITY\_DN0\_c3\_g1\_i1.p2 |  | Image |  | Peak List |  |
|  | | | | | | | | | | | | | | | | | | | | | | | | | | | | | | | | | | | | | | | | | | | | | | | | | | | | | |  |
|  | High |  | [R].VIPFVASVAAEMmQHVYcAASKKc.[-] |  | HCD |  | M13(Oxidation); C18(Dehydro); C24(Dehydro) |  | 3.98 |  | 1.3e-3 |  | 2.6e-2 |  | 0.483 |  | 260.26 |  | 0.024 |  | y1+-H(4) C O S(16): 1720.76; y1+-H(4) C O S(15): 1649.72; y1+-H(4) C O S(14): 1578.69; y1+-H(4) C O S(13): 1449.64; b2+-H(4) C O S(12): 584.32; y2+-H(4) C O S(22): 1161.05; y2+-H(4) C O S(19): 989.45 |  | M12(Oxidation): 97.64; C18(Dehydro): 100; C24(Dehydro): 100 |  |  |  |  |  |  |  | 0.61 |  | 1 |  | Sequest HT (3) |  | 3 |  | 866.41772 |  | 0.12 |  | 39.6551 |  | 10898 |  | 10898 |  | TRINITY\_DN5345\_c0\_g1\_i1.p1; TRINITY\_DN5345\_c0\_g1\_i2.p1\_ORF1; TRINITY\_DN5345\_c0\_g1\_i2.p1; TRINITY\_DN0\_c3\_g1\_i1.p2 |  | Image |  | Peak List |  |
|  | | | | | | | | | | | | | | | | | | | | | | | | | | | | | | | | | | | | | | | | | | | | | | | | | | | | | |  |
|  | High |  | [R].VIPFVASVAAEmMQHVYcAASKKc.[-] |  | HCD |  | M12(Oxidation); C18(Dehydro); C24(Dehydro) |  | 3.98 |  | 1.3e-3 |  | 2.6e-2 |  | 0.483 |  | 276.42 |  | 0.976 |  | y1+-H(4) C O S(16): 1720.76; y1+-H(4) C O S(15): 1649.72; y1+-H(4) C O S(14): 1578.69; y1+-H(4) C O S(13): 1449.64; b2+-H(4) C O S(12): 584.32; y2+-H(4) C O S(22): 1161.05; y2+-H(4) C O S(19): 989.45 |  | M12(Oxidation): 97.64; C18(Dehydro): 100; C24(Dehydro): 100 |  |  |  |  |  |  |  | 0.61 |  | 1 |  | Sequest HT (3) |  | 3 |  | 866.41772 |  | 0.12 |  | 39.6551 |  | 10898 |  | 10898 |  | TRINITY\_DN5345\_c0\_g1\_i1.p1; TRINITY\_DN5345\_c0\_g1\_i2.p1\_ORF1; TRINITY\_DN5345\_c0\_g1\_i2.p1; TRINITY\_DN0\_c3\_g1\_i1.p2 |  | Image |  | Peak List |  |
|  | | | | | | | | | | | | | | | | | | | | | | | | | | | | | | | | | | | | | | | | | | | | | | | | | | | | | |  |
|  | High |  | [R].VIPFVASVAAEmMQHVYcAASKKc.[-] |  | HCD |  | M12(Oxidation); C18(Dehydro); C24(Dehydro) |  | 4.14 |  | 1.3e-3 |  | 2.7e-2 |  | 0.475 |  | 309.87 |  | 1.000 |  | y1+-H(4) C O S(15): 1649.72; y1+-H(4) C O S(14): 1578.69; b2+-H(4) C O S(12): 584.32; y2+-H(4) C O S(19): 989.45; y2+-H(4) C O S(16): 860.88; y2+-H(4) C O S(15): 825.37; y2+-H(4) C O S(14): 789.85; y1+(12): 1366.61; y2+(12): 683.81 |  | M12(Oxidation): 100; C18(Dehydro): 100; C24(Dehydro): 100 |  |  |  |  |  |  |  | 0.03 |  | 1 |  | Sequest HT (3) |  | 4 |  | 650.06555 |  | 0.80 |  | 39.7286 |  | 10937 |  | 10937 |  | TRINITY\_DN5345\_c0\_g1\_i1.p1; TRINITY\_DN5345\_c0\_g1\_i2.p1\_ORF1; TRINITY\_DN5345\_c0\_g1\_i2.p1; TRINITY\_DN0\_c3\_g1\_i1.p2 |  | Image |  | Peak List |  |
|  | | | | | | | | | | | | | | | | | | | | | | | | | | | | | | | | | | | | | | | | | | | | | | | | | | | | | |  |
|  | High |  | [R].VIPFVASVAAEMmQHVYcAASKKc.[-] |  | HCD |  | M13(Oxidation); C18(Dehydro); C24(Dehydro) |  | 4.01 |  | 1.3e-3 |  | 3.4e-2 |  | 0.42 |  | 259.20 |  | 0.000 |  | y1+-H(4) C O S(15): 1649.72; y1+-H(4) C O S(14): 1578.69; b2+-H(4) C O S(12): 584.32; y2+-H(4) C O S(19): 989.45; y2+-H(4) C O S(16): 860.88; y2+-H(4) C O S(15): 825.37; y2+-H(4) C O S(14): 789.85; y1+(12): 1366.61; y2+(12): 683.81 |  | M12(Oxidation): 100; C18(Dehydro): 100; C24(Dehydro): 100 |  |  |  |  |  |  |  |  |  | 2 |  | Sequest HT (3) |  | 4 |  | 650.06555 |  | 0.80 |  | 39.7286 |  | 10937 |  | 10937 |  | TRINITY\_DN5345\_c0\_g1\_i1.p1; TRINITY\_DN5345\_c0\_g1\_i2.p1\_ORF1; TRINITY\_DN5345\_c0\_g1\_i2.p1; TRINITY\_DN0\_c3\_g1\_i1.p2 |  | Image |  | Peak List |  |
|  | | | | | | | | | | | | | | | | | | | | | | | | | | | | | | | | | | | | | | | | | | | | | | | | | | | | | |  |
|  | High |  | [R].VIPFVASVAAEmMQHVYcAASKKc.[-] |  | HCD |  | M12(Oxidation); C18(Dehydro); C24(Dehydro) |  | 4.28 |  | 1.3e-3 |  | 1.0e-2 |  | 0.691 |  | 343.23 |  | 1.000 |  | y1+-H(4) C O S(16): 1720.76; y1+-H(4) C O S(15): 1649.72; y1+-H(4) C O S(14): 1578.69; y1+-H(4) C O S(13): 1449.64; b2+-H(4) C O S(12): 584.32; y2+-H(4) C O S(22): 1161.05; y2+-H(4) C O S(19): 989.45; y2+-H(4) C O S(16): 860.88; y1+(12): 1366.61 |  | M12(Oxidation): 99.96; C18(Dehydro): 100; C24(Dehydro): 100 |  |  |  |  |  |  |  | 0.67 |  | 1 |  | Sequest HT (3) |  | 3 |  | 866.41785 |  | 0.26 |  | 39.8072 |  | 10979 |  | 10979 |  | TRINITY\_DN5345\_c0\_g1\_i1.p1; TRINITY\_DN5345\_c0\_g1\_i2.p1\_ORF1; TRINITY\_DN5345\_c0\_g1\_i2.p1; TRINITY\_DN0\_c3\_g1\_i1.p2 |  | Image |  | Peak List |  |
|  | | | | | | | | | | | | | | | | | | | | | | | | | | | | | | | | | | | | | | | | | | | | | | | | | | | | | |  |
|  | High |  | [R].VIPFVASVAAEMmQHVYcAASKKc.[-] |  | HCD |  | M13(Oxidation); C18(Dehydro); C24(Dehydro) |  | 4.28 |  | 1.3e-3 |  | 1.0e-2 |  | 0.691 |  | 309.41 |  | 0.000 |  | y1+-H(4) C O S(16): 1720.76; y1+-H(4) C O S(15): 1649.72; y1+-H(4) C O S(14): 1578.69; y1+-H(4) C O S(13): 1449.64; b2+-H(4) C O S(12): 584.32; y2+-H(4) C O S(22): 1161.05; y2+-H(4) C O S(19): 989.45; y2+-H(4) C O S(16): 860.88; y1+(12): 1366.61 |  | M12(Oxidation): 99.96; C18(Dehydro): 100; C24(Dehydro): 100 |  |  |  |  |  |  |  | 0.67 |  | 1 |  | Sequest HT (3) |  | 3 |  | 866.41785 |  | 0.26 |  | 39.8072 |  | 10979 |  | 10979 |  | TRINITY\_DN5345\_c0\_g1\_i1.p1; TRINITY\_DN5345\_c0\_g1\_i2.p1\_ORF1; TRINITY\_DN5345\_c0\_g1\_i2.p1; TRINITY\_DN0\_c3\_g1\_i1.p2 |  | Image |  | Peak List |  |
|  | | | | | | | | | | | | | | | | | | | | | | | | | | | | | | | | | | | | | | | | | | | | | | | | | | | | | |  |
|  | High |  | [R].VIPFVASVAAEMmQHVYcAASKKc.[-] |  | HCD |  | M13(Oxidation); C18(Dehydro); C24(Dehydro) |  | 3.76 |  | 3.1e-3 |  | 1.1e-1 |  | 0.155 |  | 236.92 |  | 0.999 |  | y1+-H(4) C O S(15): 1649.72; y1+-H(4) C O S(14): 1578.69; y1+-H(4) C O S(12): 1318.60; y2+-H(4) C O S(15): 825.37; y1+(12): 1382.60; y2+(12): 691.80 |  | M13(Oxidation): 99.93; C18(Dehydro): 100; C24(Dehydro): 100 |  |  |  |  |  |  |  | 0.05 |  | 1 |  | Sequest HT (3) |  | 4 |  | 650.06573 |  | 1.08 |  | 39.8786 |  | 11019 |  | 11019 |  | TRINITY\_DN5345\_c0\_g1\_i1.p1; TRINITY\_DN5345\_c0\_g1\_i2.p1\_ORF1; TRINITY\_DN5345\_c0\_g1\_i2.p1; TRINITY\_DN0\_c3\_g1\_i1.p2 |  | Image |  | Peak List |  |
|  | | | | | | | | | | | | | | | | | | | | | | | | | | | | | | | | | | | | | | | | | | | | | | | | | | | | | |  |
|  | High |  | [R].VIPFVASVAAEmMQHVYcAASKKc.[-] |  | HCD |  | M12(Oxidation); C18(Dehydro); C24(Dehydro) |  | 3.58 |  | 6.5e-3 |  | 2.2e-1 |  | 0.001 |  | 205.43 |  | 0.001 |  | y1+-H(4) C O S(15): 1649.72; y1+-H(4) C O S(14): 1578.69; y1+-H(4) C O S(12): 1318.60; y2+-H(4) C O S(15): 825.37; y1+(12): 1382.60; y2+(12): 691.80 |  | M13(Oxidation): 99.93; C18(Dehydro): 100; C24(Dehydro): 100 |  |  |  |  |  |  |  |  |  | 2 |  | Sequest HT (3) |  | 4 |  | 650.06573 |  | 1.08 |  | 39.8786 |  | 11019 |  | 11019 |  | TRINITY\_DN5345\_c0\_g1\_i1.p1; TRINITY\_DN5345\_c0\_g1\_i2.p1\_ORF1; TRINITY\_DN5345\_c0\_g1\_i2.p1; TRINITY\_DN0\_c3\_g1\_i1.p2 |  | Image |  | Peak List |  |
|  | | | | | | | | | | | | | | | | | | | | | | | | | | | | | | | | | | | | | | | | | | | | | | | | | | | | | |  |
|  | High |  | [R].VIPFVASVAAEMmQHVYcAASKKc.[-] |  | HCD |  | M13(Oxidation); C18(Dehydro); C24(Dehydro) |  | 3.45 |  | 1.3e-3 |  | 1.3e-2 |  | 0.637 |  | 215.69 |  | 0.500 |  | y1+-H(4) C O S(16): 1720.76; y1+-H(4) C O S(15): 1649.72; y1+-H(4) C O S(13): 1449.64; b2+-H(4) C O S(12): 584.32; y2+-H(4) C O S(16): 860.88; y2+-H(4) C O S(15): 825.37 |  | M12(Oxidation): 50; M13(Oxidation): 50; C18(Dehydro): 100; C24(Dehydro): 100 |  |  |  |  |  |  |  | 0.06 |  | 1 |  | Sequest HT (3) |  | 3 |  | 866.41705 |  | -0.65 |  | 39.9573 |  | 11060 |  | 11060 |  | TRINITY\_DN5345\_c0\_g1\_i1.p1; TRINITY\_DN5345\_c0\_g1\_i2.p1\_ORF1; TRINITY\_DN5345\_c0\_g1\_i2.p1; TRINITY\_DN0\_c3\_g1\_i1.p2 |  | Image |  | Peak List |  |
|  | | | | | | | | | | | | | | | | | | | | | | | | | | | | | | | | | | | | | | | | | | | | | | | | | | | | | |  |
|  | High |  | [R].VIPFVASVAAEMMQHVYcAASKKc.[-] |  | HCD |  | C18(Dehydro); C24(Dehydro) |  | 4.54 |  | 1.3e-3 |  | 1.0e-2 |  | 0.689 |  | 278.67 |  | 1.000 |  |  |  | C18(Dehydro): 100; C24(Dehydro): 100 |  |  |  |  |  |  |  | 0.65 |  | 1 |  | Sequest HT (3) |  | 4 |  | 646.06628 |  | -0.03 |  | 44.1280 |  | 12871 |  | 12871 |  | TRINITY\_DN5345\_c0\_g1\_i1.p1; TRINITY\_DN5345\_c0\_g1\_i2.p1\_ORF1; TRINITY\_DN5345\_c0\_g1\_i2.p1; TRINITY\_DN0\_c3\_g1\_i1.p2 |  | Image |  | Peak List |  |
|  | | | | | | | | | | | | | | | | | | | | | | | | | | | | | | | | | | | | | | | | | | | | | | | | | | | | | |  |
|  | High |  | [R].VIPFVASVAAEMMQHVYcAASKKc.[-] |  | HCD |  | C18(Dehydro); C24(Dehydro) |  | 4.93 |  | 1.3e-3 |  | 1.4e-2 |  | 0.621 |  | 331.59 |  | 1.000 |  |  |  | C18(Dehydro): 100; C24(Dehydro): 100 |  |  |  |  |  |  |  | 0.67 |  | 1 |  | Sequest HT (3) |  | 3 |  | 861.08594 |  | -0.05 |  | 44.1443 |  | 12877 |  | 12877 |  | TRINITY\_DN5345\_c0\_g1\_i1.p1; TRINITY\_DN5345\_c0\_g1\_i2.p1\_ORF1; TRINITY\_DN5345\_c0\_g1\_i2.p1; TRINITY\_DN0\_c3\_g1\_i1.p2 |  | Image |  | Peak List |  |
|  | | | | | | | | | | | | | | | | | | | | | | | | | | | | | | | | | | | | | | | | | | | | | | | | | | | | | |  |
|  | High |  | [R].VIPFVASVAAEMMQHVYcAASKKc.[-] |  | HCD |  | C18(Dehydro); C24(Dehydro) |  | 4.12 |  | 1.3e-3 |  | 2.3e-4 |  | 1.52 |  | 426.32 |  | 1.000 |  |  |  | C18(Dehydro): 100; C24(Dehydro): 100 |  |  |  |  |  |  |  | 0.66 |  | 1 |  | Sequest HT (3) |  | 2 |  | 1291.12366 |  | -1.30 |  | 44.1572 |  | 12883 |  | 12883 |  | TRINITY\_DN5345\_c0\_g1\_i1.p1; TRINITY\_DN5345\_c0\_g1\_i2.p1\_ORF1; TRINITY\_DN5345\_c0\_g1\_i2.p1; TRINITY\_DN0\_c3\_g1\_i1.p2 |  | Image |  | Peak List |  |
|  | | | | | | | | | | | | | | | | | | | | | | | | | | | | | | | | | | | | | | | | | | | | | | | | | | | | | |  |
|  | High |  | [R].VIPFVASVAAEMMQHVYcAASKKc.[-] |  | HCD |  | C18(Dehydro); C24(Dehydro) |  | 4.23 |  | 1.3e-3 |  | 8.1e-3 |  | 0.737 |  | 271.73 |  | 1.000 |  |  |  | C18(Dehydro): 100; C24(Dehydro): 100 |  |  |  |  |  |  |  | 0.59 |  | 1 |  | Sequest HT (3) |  | 4 |  | 646.06641 |  | 0.16 |  | 44.2739 |  | 12929 |  | 12929 |  | TRINITY\_DN5345\_c0\_g1\_i1.p1; TRINITY\_DN5345\_c0\_g1\_i2.p1\_ORF1; TRINITY\_DN5345\_c0\_g1\_i2.p1; TRINITY\_DN0\_c3\_g1\_i1.p2 |  | Image |  | Peak List |  |
|  | | | | | | | | | | | | | | | | | | | | | | | | | | | | | | | | | | | | | | | | | | | | | | | | | | | | | |  |
|  | **TRINITY\_DN6\_c2\_g1\_i1.p1 - TRINITY\_DN6\_c2\_g1\_i1.p1** | | | | | | | | | | | | | | | | | | | | | | | | | | | | | | | | | | | | | | | | | | | | | | | | | | | |  |  |
|  | | | | | | | | | | | | | | | | | | | | | | | | | | | | | | | | | | | | | | | | | | | | | | | | | | | | | |  |
|  | High |  | [N].VLTNVAGTVKDKIKcKFTGGc.[-] |  | HCD |  | C15(Dehydro); C21(Dehydro) |  | 3.10 |  | 1.3e-3 |  | 2.3e-2 |  | 0.508 |  | 221.13 |  | 1.000 |  |  |  | C15(Dehydro): 100; C21(Dehydro): 100 |  |  |  |  |  |  |  | 0.50 |  | 1 |  | Sequest HT (3) |  | 4 |  | 545.79498 |  | 0.69 |  | 26.7653 |  | 5388 |  | 5388 |  | TRINITY\_DN6\_c2\_g1\_i1.p1 |  | Image |  | Peak List |  |
|  | | | | | | | | | | | | | | | | | | | | | | | | | | | | | | | | | | | | | | | | | | | | | | | | | | | | | |  |
|  | High |  | [R].GLMNTVLNVLTNVAGTVKDKIKcKFTGGc.[-] |  | HCD |  | C23(Dehydro); C29(Dehydro) |  | 4.29 |  | 1.3e-3 |  | 1.1e-3 |  | 1.17 |  | 311.77 |  | 1.000 |  |  |  | C23(Dehydro): 100; C29(Dehydro): 100 |  |  |  |  |  |  |  | 0.43 |  | 1 |  | Sequest HT (3) |  | 4 |  | 756.40222 |  | -0.52 |  | 48.5648 |  | 14771 |  | 14771 |  | TRINITY\_DN6\_c2\_g1\_i1.p1 |  | Image |  | Peak List |  |
|  | | | | | | | | | | | | | | | | | | | | | | | | | | | | | | | | | | | | | | | | | | | | | | | | | | | | | |  |
|  | High |  | [R].GLMNTVLNVLTNVAGTVKDKIKcKFTGGc.[-] |  | HCD |  | C23(Dehydro); C29(Dehydro) |  | 5.34 |  | 1.3e-3 |  | 1.5e-3 |  | 1.11 |  | 306.15 |  | 1.000 |  |  |  | C23(Dehydro): 100; C29(Dehydro): 100 |  |  |  |  |  |  |  | 0.59 |  | 1 |  | Sequest HT (3) |  | 3 |  | 1008.19904 |  | -2.01 |  | 48.6252 |  | 14794 |  | 14794 |  | TRINITY\_DN6\_c2\_g1\_i1.p1 |  | Image |  | Peak List |  |
|  | | | | | | | | | | | | | | | | | | | | | | | | | | | | | | | | | | | | | | | | | | | | | | | | | | | | | |  |
|  | High |  | [R].GLMNTVLNVLTNVAGTVKDKIKcKFTGGc.[-] |  | HCD |  | C23(Dehydro); C29(Dehydro) |  | 4.41 |  | 1.3e-3 |  | 3.3e-4 |  | 1.44 |  | 316.66 |  | 1.000 |  |  |  | C23(Dehydro): 100; C29(Dehydro): 100 |  |  |  |  |  |  |  | 0.54 |  | 1 |  | Sequest HT (3) |  | 4 |  | 756.40118 |  | -1.89 |  | 48.7089 |  | 14824 |  | 14824 |  | TRINITY\_DN6\_c2\_g1\_i1.p1 |  | Image |  | Peak List |  |
|  | | | | | | | | | | | | | | | | | | | | | | | | | | | | | | | | | | | | | | | | | | | | | | | | | | | | | |  |
|  | High |  | [R].GLMNTVLNVLTNVAGTVKDKIKcKFTGGc.[-] |  | HCD |  | C23(Dehydro); C29(Dehydro) |  | 5.71 |  | 1.3e-3 |  | 9.1e-4 |  | 1.21 |  | 372.43 |  | 1.000 |  |  |  | C23(Dehydro): 100; C29(Dehydro): 100 |  |  |  |  |  |  |  | 0.60 |  | 1 |  | Sequest HT (3) |  | 3 |  | 1008.19855 |  | -2.49 |  | 48.7817 |  | 14857 |  | 14857 |  | TRINITY\_DN6\_c2\_g1\_i1.p1 |  | Image |  | Peak List |  |
|  | | | | | | | | | | | | | | | | | | | | | | | | | | | | | | | | | | | | | | | | | | | | | | | | | | | | | |  |
|  | High |  | [R].GLMNTVLNVLTNVAGTVKDKIKcKFTGGc.[-] |  | HCD |  | C23(Dehydro); C29(Dehydro) |  | 3.85 |  | 1.3e-3 |  | 3.1e-2 |  | 0.445 |  | 256.14 |  | 1.000 |  |  |  | C23(Dehydro): 100; C29(Dehydro): 100 |  |  |  |  |  |  |  | 0.40 |  | 1 |  | Sequest HT (3) |  | 4 |  | 756.40192 |  | -0.92 |  | 48.8613 |  | 14892 |  | 14892 |  | TRINITY\_DN6\_c2\_g1\_i1.p1 |  | Image |  | Peak List |  |
|  | | | | | | | | | | | | | | | | | | | | | | | | | | | | | | | | | | | | | | | | | | | | | | | | | | | | | |  |
|  | High |  | [R].GLMNTVLNVLTnVAGTVKDKIKcKFTGGc.[-] |  | HCD |  | N12(Deamidated); C23(Dehydro); C29(Dehydro) |  | 4.46 |  | 1.3e-3 |  | 4.6e-3 |  | 0.862 |  | 264.99 |  | 1.000 |  | y2+(21): 1091.07; y2+(20): 1041.54; y2+(19): 985.00; y2+(18): 934.47 |  | N12(Deamidated): 100; C23(Dehydro): 100; C29(Dehydro): 100 |  |  |  |  |  |  |  | 0.16 |  | 1 |  | Sequest HT (3) |  | 4 |  | 756.64746 |  | -1.53 |  | 51.7811 |  | 16055 |  | 16055 |  | TRINITY\_DN6\_c2\_g1\_i1.p1 |  | Image |  | Peak List |  |
|  | | | | | | | | | | | | | | | | | | | | | | | | | | | | | | | | | | | | | | | | | | | | | | | | | | | | | |  |
|  | High |  | [R].GLMNTVLNVLTnVAGTVKDKIKcKFTGGc.[-] |  | HCD |  | N12(Deamidated); C23(Dehydro); C29(Dehydro) |  | 4.90 |  | 1.3e-3 |  | 2.0e-3 |  | 1.05 |  | 329.36 |  | 1.000 |  | y1+(19): 1968.99; y2+(21): 1091.07; y2+(20): 1041.54; y2+(19): 985.00; y2+(18): 934.47 |  | N12(Deamidated): 100; C23(Dehydro): 100; C29(Dehydro): 100 |  |  |  |  |  |  |  | 0.16 |  | 1 |  | Sequest HT (3) |  | 4 |  | 756.64941 |  | 1.05 |  | 51.9547 |  | 16126 |  | 16126 |  | TRINITY\_DN6\_c2\_g1\_i1.p1 |  | Image |  | Peak List |  |
|  | | | | | | | | | | | | | | | | | | | | | | | | | | | | | | | | | | | | | | | | | | | | | | | | | | | | | |  |
|  | High |  | [R].GLMNTVLNVLTnVAGTVKDKIKcKFTGGc.[-] |  | HCD |  | N12(Deamidated); C23(Dehydro); C29(Dehydro) |  | 5.92 |  | 1.3e-3 |  | 2.6e-3 |  | 0.981 |  | 345.08 |  | 1.000 |  | y1+(19): 1968.99; y1+(18): 1867.94; y2+(21): 1091.07; y2+(20): 1041.54; y2+(19): 985.00; y2+(18): 934.47 |  | N12(Deamidated): 100; C23(Dehydro): 100; C29(Dehydro): 100 |  |  |  |  |  |  |  | 0.22 |  | 1 |  | Sequest HT (3) |  | 3 |  | 1008.52606 |  | -2.98 |  | 52.0141 |  | 16152 |  | 16152 |  | TRINITY\_DN6\_c2\_g1\_i1.p1 |  | Image |  | Peak List |  |
|  | | | | | | | | | | | | | | | | | | | | | | | | | | | | | | | | | | | | | | | | | | | | | | | | | | | | | |  |
|  | High |  | [R].GLMNTVLNVLTnVAGTVKDKIKcKFTGGc.[-] |  | HCD |  | N12(Deamidated); C23(Dehydro); C29(Dehydro) |  | 5.85 |  | 1.3e-3 |  | 1.3e-3 |  | 1.13 |  | 365.09 |  | 1.000 |  | y1+(19): 1968.99; y2+(21): 1091.07; y2+(20): 1041.54; y2+(19): 985.00; y2+(18): 934.47 |  | N12(Deamidated): 100; C23(Dehydro): 100; C29(Dehydro): 100 |  |  |  |  |  |  |  | 0.16 |  | 1 |  | Sequest HT (3) |  | 3 |  | 1008.52765 |  | -1.41 |  | 52.1881 |  | 16224 |  | 16224 |  | TRINITY\_DN6\_c2\_g1\_i1.p1 |  | Image |  | Peak List |  |
|  | | | | | | | | | | | | | | | | | | | | | | | | | | | | | | | | | | | | | | | | | | | | | | | | | | | | | |  |
|  | High |  | [R].GLMNTVLNVLTnVAGTVKDKIKcKFTGGc.[-] |  | HCD |  | N12(Deamidated); C23(Dehydro); C29(Dehydro) |  | 5.43 |  | 1.3e-3 |  | 6.1e-4 |  | 1.3 |  | 354.55 |  | 1.000 |  | y2+(21): 1091.07; y2+(20): 1041.54; y2+(19): 985.00; y2+(18): 934.47 |  | N12(Deamidated): 100; C23(Dehydro): 100; C29(Dehydro): 100 |  |  |  |  |  |  |  | 0.04 |  | 1 |  | Sequest HT (3) |  | 4 |  | 756.64801 |  | -0.80 |  | 52.2857 |  | 16260 |  | 16260 |  | TRINITY\_DN6\_c2\_g1\_i1.p1 |  | Image |  | Peak List |  |
|  | | | | | | | | | | | | | | | | | | | | | | | | | | | | | | | | | | | | | | | | | | | | | | | | | | | | | |  |
|  | High |  | [R].GLMNTVLnVLTNVAGTVKDKIKcKFTGGc.[-] |  | HCD |  | N8(Deamidated); C23(Dehydro); C29(Dehydro) |  | 5.21 |  | 1.3e-3 |  | 7.0e-4 |  | 1.27 |  | 281.71 |  | 0.000 |  | y2+(21): 1091.07; y2+(20): 1041.54; y2+(19): 985.00; y2+(18): 934.47 |  | N12(Deamidated): 100; C23(Dehydro): 100; C29(Dehydro): 100 |  |  |  |  |  |  |  |  |  | 2 |  | Sequest HT (3) |  | 4 |  | 756.64801 |  | -0.80 |  | 52.2857 |  | 16260 |  | 16260 |  | TRINITY\_DN6\_c2\_g1\_i1.p1 |  | Image |  | Peak List |  |
|  | | | | | | | | | | | | | | | | | | | | | | | | | | | | | | | | | | | | | | | | | | | | | | | | | | | | | |  |
|  | High |  | [R].GLMNTVLNVLTnVAGTVKDKIKcKFTGGc.[-] |  | HCD |  | N12(Deamidated); C23(Dehydro); C29(Dehydro) |  | 6.04 |  | 1.3e-3 |  | 3.7e-4 |  | 1.41 |  | 409.54 |  | 1.000 |  | y1+(20): 2082.07; y1+(19): 1968.99; y1+(18): 1867.94; y2+(21): 1091.07; y2+(20): 1041.54; y2+(19): 985.00; y2+(18): 934.47 |  | N12(Deamidated): 100; C23(Dehydro): 100; C29(Dehydro): 100 |  |  |  |  |  |  |  | 0.17 |  | 1 |  | Sequest HT (3) |  | 3 |  | 1008.52863 |  | -0.44 |  | 52.3351 |  | 16281 |  | 16281 |  | TRINITY\_DN6\_c2\_g1\_i1.p1 |  | Image |  | Peak List |  |
|  | | | | | | | | | | | | | | | | | | | | | | | | | | | | | | | | | | | | | | | | | | | | | | | | | | | | | |  |
|  | High |  | [R].GLMNTVLNVLTnVAGTVKDKIKcKFTGGc.[-] |  | HCD |  | N12(Deamidated); C23(Dehydro); C29(Dehydro) |  | 5.14 |  | 1.3e-3 |  | 2.2e-3 |  | 1.02 |  | 307.55 |  | 1.000 |  | y2+(21): 1091.07; y2+(20): 1041.54; y2+(19): 985.00; y2+(18): 934.47 |  | N12(Deamidated): 100; C23(Dehydro): 100; C29(Dehydro): 100 |  |  |  |  |  |  |  | 0.01 |  | 1 |  | Sequest HT (3) |  | 4 |  | 756.65082 |  | 2.91 |  | 52.4381 |  | 16317 |  | 16317 |  | TRINITY\_DN6\_c2\_g1\_i1.p1 |  | Image |  | Peak List |  |
|  | | | | | | | | | | | | | | | | | | | | | | | | | | | | | | | | | | | | | | | | | | | | | | | | | | | | | |  |
|  | High |  | [R].GLMNTVLnVLTNVAGTVKDKIKcKFTGGc.[-] |  | HCD |  | N8(Deamidated); C23(Dehydro); C29(Dehydro) |  | 5.10 |  | 1.3e-3 |  | 1.5e-3 |  | 1.11 |  | 238.42 |  | 0.000 |  | y2+(21): 1091.07; y2+(20): 1041.54; y2+(19): 985.00; y2+(18): 934.47 |  | N12(Deamidated): 100; C23(Dehydro): 100; C29(Dehydro): 100 |  |  |  |  |  |  |  |  |  | 2 |  | Sequest HT (3) |  | 4 |  | 756.65082 |  | 2.91 |  | 52.4381 |  | 16317 |  | 16317 |  | TRINITY\_DN6\_c2\_g1\_i1.p1 |  | Image |  | Peak List |  |
|  | | | | | | | | | | | | | | | | | | | | | | | | | | | | | | | | | | | | | | | | | | | | | | | | | | | | | |  |
|  | High |  | [R].GLMNTVLNVLTnVAGTVKDKIKcKFTGGc.[-] |  | HCD |  | N12(Deamidated); C23(Dehydro); C29(Dehydro) |  | 4.40 |  | 1.3e-3 |  | 1.0e-3 |  | 1.19 |  | 236.87 |  | 1.000 |  | y2+(21): 1091.07; y2+(19): 985.00; y2+(18): 934.47 |  | N12(Deamidated): 100; C23(Dehydro): 100; C29(Dehydro): 100 |  |  |  |  |  |  |  | 0.03 |  | 1 |  | Sequest HT (3) |  | 5 |  | 605.52002 |  | -0.55 |  | 52.5027 |  | 16345 |  | 16345 |  | TRINITY\_DN6\_c2\_g1\_i1.p1 |  | Image |  | Peak List |  |
|  | | | | | | | | | | | | | | | | | | | | | | | | | | | | | | | | | | | | | | | | | | | | | | | | | | | | | |  |
|  | High |  | [R].GLMNTVLnVLTNVAGTVKDKIKcKFTGGc.[-] |  | HCD |  | N8(Deamidated); C23(Dehydro); C29(Dehydro) |  | 4.27 |  | 1.3e-3 |  | 2.2e-3 |  | 1.02 |  | 186.13 |  | 0.000 |  | y2+(21): 1091.07; y2+(19): 985.00; y2+(18): 934.47 |  | N12(Deamidated): 100; C23(Dehydro): 100; C29(Dehydro): 100 |  |  |  |  |  |  |  |  |  | 2 |  | Sequest HT (3) |  | 5 |  | 605.52002 |  | -0.55 |  | 52.5027 |  | 16345 |  | 16345 |  | TRINITY\_DN6\_c2\_g1\_i1.p1 |  | Image |  | Peak List |  |
|  | | | | | | | | | | | | | | | | | | | | | | | | | | | | | | | | | | | | | | | | | | | | | | | | | | | | | |  |
|  | High |  | [R].GLMNTVLnVLTNVAGTVKDKIKcKFTGGc.[-] |  | HCD |  | N8(Deamidated); C23(Dehydro); C29(Dehydro) |  | 4.47 |  | 1.3e-3 |  | 3.7e-3 |  | 0.909 |  | 229.03 |  | 0.000 |  | y1+(19): 1968.99; y2+(21): 1091.07; y2+(20): 1041.54; y2+(19): 985.00; y2+(18): 934.47 |  | N12(Deamidated): 100; C23(Dehydro): 100; C29(Dehydro): 100 |  |  |  |  |  |  |  | 0.02 |  | 1 |  | Sequest HT (3) |  | 4 |  | 756.64777 |  | -1.13 |  | 52.5881 |  | 16375 |  | 16375 |  | TRINITY\_DN6\_c2\_g1\_i1.p1 |  | Image |  | Peak List |  |
|  | | | | | | | | | | | | | | | | | | | | | | | | | | | | | | | | | | | | | | | | | | | | | | | | | | | | | |  |
|  | High |  | [R].GLMNTVLNVLTnVAGTVKDKIKcKFTGGc.[-] |  | HCD |  | N12(Deamidated); C23(Dehydro); C29(Dehydro) |  | 4.38 |  | 1.3e-3 |  | 3.3e-3 |  | 0.932 |  | 320.59 |  | 1.000 |  | y1+(19): 1968.99; y2+(21): 1091.07; y2+(20): 1041.54; y2+(19): 985.00; y2+(18): 934.47 |  | N12(Deamidated): 100; C23(Dehydro): 100; C29(Dehydro): 100 |  |  |  |  |  |  |  |  |  | 2 |  | Sequest HT (3) |  | 4 |  | 756.64777 |  | -1.13 |  | 52.5881 |  | 16375 |  | 16375 |  | TRINITY\_DN6\_c2\_g1\_i1.p1 |  | Image |  | Peak List |  |
|  | | | | | | | | | | | | | | | | | | | | | | | | | | | | | | | | | | | | | | | | | | | | | | | | | | | | | |  |
|  | High |  | [R].GLMNTVLnVLTNVAGTVKDKIKcKFTGGc.[-] |  | HCD |  | N8(Deamidated); C23(Dehydro); C29(Dehydro) |  | 4.88 |  | 1.3e-3 |  | 3.0e-3 |  | 0.955 |  | 342.02 |  | 0.000 |  | y1+(20): 2082.07; y1+(19): 1968.99; y2+(21): 1091.07; y2+(20): 1041.54; y2+(19): 985.00; y2+(18): 934.47 |  | N12(Deamidated): 99.98; C23(Dehydro): 100; C29(Dehydro): 100 |  |  |  |  |  |  |  | 0.03 |  | 1 |  | Sequest HT (3) |  | 3 |  | 1008.52637 |  | -2.68 |  | 52.6923 |  | 16412 |  | 16412 |  | TRINITY\_DN6\_c2\_g1\_i1.p1 |  | Image |  | Peak List |  |
|  | | | | | | | | | | | | | | | | | | | | | | | | | | | | | | | | | | | | | | | | | | | | | | | | | | | | | |  |
|  | High |  | [R].GLMNTVLNVLTnVAGTVKDKIKcKFTGGc.[-] |  | HCD |  | N12(Deamidated); C23(Dehydro); C29(Dehydro) |  | 4.72 |  | 1.3e-3 |  | 3.1e-3 |  | 0.943 |  | 378.77 |  | 1.000 |  | y1+(20): 2082.07; y1+(19): 1968.99; y2+(21): 1091.07; y2+(20): 1041.54; y2+(19): 985.00; y2+(18): 934.47 |  | N12(Deamidated): 99.98; C23(Dehydro): 100; C29(Dehydro): 100 |  |  |  |  |  |  |  |  |  | 2 |  | Sequest HT (3) |  | 3 |  | 1008.52637 |  | -2.68 |  | 52.6923 |  | 16412 |  | 16412 |  | TRINITY\_DN6\_c2\_g1\_i1.p1 |  | Image |  | Peak List |  |
|  | | | | | | | | | | | | | | | | | | | | | | | | | | | | | | | | | | | | | | | | | | | | | | | | | | | | | |  |
|  | High |  | [R].GLMNTVLnVLTNVAGTVKDKIKcKFTGGc.[-] |  | HCD |  | N8(Deamidated); C23(Dehydro); C29(Dehydro) |  | 4.94 |  | 1.3e-3 |  | 4.2e-3 |  | 0.879 |  | 222.88 |  | 0.000 |  | y2+(21): 1091.07; y2+(20): 1041.54; y2+(19): 985.00; y2+(18): 934.47 |  | N12(Deamidated): 99.97; C23(Dehydro): 100; C29(Dehydro): 100 |  |  |  |  |  |  |  | 0.04 |  | 1 |  | Sequest HT (3) |  | 4 |  | 756.64673 |  | -2.50 |  | 52.7457 |  | 16430 |  | 16430 |  | TRINITY\_DN6\_c2\_g1\_i1.p1 |  | Image |  | Peak List |  |
|  | | | | | | | | | | | | | | | | | | | | | | | | | | | | | | | | | | | | | | | | | | | | | | | | | | | | | |  |
|  | High |  | [R].GLMNTVLNVLTnVAGTVKDKIKcKFTGGc.[-] |  | HCD |  | N12(Deamidated); C23(Dehydro); C29(Dehydro) |  | 4.72 |  | 1.3e-3 |  | 4.9e-3 |  | 0.848 |  | 257.96 |  | 1.000 |  | y2+(21): 1091.07; y2+(20): 1041.54; y2+(19): 985.00; y2+(18): 934.47 |  | N12(Deamidated): 99.97; C23(Dehydro): 100; C29(Dehydro): 100 |  |  |  |  |  |  |  |  |  | 2 |  | Sequest HT (3) |  | 4 |  | 756.64673 |  | -2.50 |  | 52.7457 |  | 16430 |  | 16430 |  | TRINITY\_DN6\_c2\_g1\_i1.p1 |  | Image |  | Peak List |  |
|  | | | | | | | | | | | | | | | | | | | | | | | | | | | | | | | | | | | | | | | | | | | | | | | | | | | | | |  |
|  | **TRINITY\_DN10285\_c0\_g1\_i1.p1 - TRINITY\_DN10285\_c0\_g1\_i1.p1** | | | | | | | | | | | | | | | | | | | | | | | | | | | | | | | | | | | | | | | | | | | | | | | | | | | |  |  |
|  | | | | | | | | | | | | | | | | | | | | | | | | | | | | | | | | | | | | | | | | | | | | | | | | | | | | | |  |
|  | High |  | [N].VAVTLLDNLKcKITKAc.[-] |  | HCD |  | C11(Dehydro); C17(Dehydro) |  | 4.05 |  | 1.3e-3 |  | 5.7e-2 |  | 0.31 |  | 268.55 |  | 1.000 |  |  |  | C11(Dehydro): 100; C17(Dehydro): 100 |  |  |  |  |  |  |  | 0.46 |  | 1 |  | Sequest HT (3) |  | 3 |  | 611.01117 |  | 0.53 |  | 34.4442 |  | 8387 |  | 8387 |  | TRINITY\_DN6\_c27\_g1\_i1.p1\_ORF2; TRINITY\_DN10285\_c0\_g1\_i1.p1\_ORF1; TRINITY\_DN6\_c27\_g1\_i1.p1\_ORF1; TRINITY\_DN10285\_c0\_g1\_i1.p1 |  | Image |  | Peak List |  |
|  | | | | | | | | | | | | | | | | | | | | | | | | | | | | | | | | | | | | | | | | | | | | | | | | | | | | | |  |
|  | High |  | [N].VAVTLLDNLKcKITKAc.[-] |  | HCD |  | C11(Dehydro); C17(Dehydro) |  | 3.51 |  | 2.4e-3 |  | 7.0e-2 |  | 0.264 |  | 229.59 |  | 1.000 |  |  |  | C11(Dehydro): 100; C17(Dehydro): 100 |  |  |  |  |  |  |  | 0.38 |  | 1 |  | Sequest HT (3) |  | 4 |  | 458.51038 |  | 0.92 |  | 34.4885 |  | 8408 |  | 8408 |  | TRINITY\_DN6\_c27\_g1\_i1.p1\_ORF2; TRINITY\_DN10285\_c0\_g1\_i1.p1\_ORF1; TRINITY\_DN6\_c27\_g1\_i1.p1\_ORF1; TRINITY\_DN10285\_c0\_g1\_i1.p1 |  | Image |  | Peak List |  |
|  | | | | | | | | | | | | | | | | | | | | | | | | | | | | | | | | | | | | | | | | | | | | | | | | | | | | | |  |
|  | High |  | [D].TAKNVAKNVAVTLLDNLKcKITKAc.[-] |  | HCD |  | C19(Dehydro); C25(Dehydro) |  | 6.76 |  | 3.1e-3 |  | 1.2e-1 |  | 0.133 |  | 260.24 |  | 1.000 |  |  |  | C19(Dehydro): 100; C25(Dehydro): 100 |  |  |  |  |  |  |  | 0.54 |  | 1 |  | Sequest HT (3) |  | 5 |  | 532.30188 |  | -1.43 |  | 36.5327 |  | 9371 |  | 9371 |  | TRINITY\_DN6\_c27\_g1\_i1.p1\_ORF2; TRINITY\_DN10285\_c0\_g1\_i1.p1\_ORF1; TRINITY\_DN6\_c27\_g1\_i1.p1\_ORF1; TRINITY\_DN10285\_c0\_g1\_i1.p1 |  | Image |  | Peak List |  |
|  | | | | | | | | | | | | | | | | | | | | | | | | | | | | | | | | | | | | | | | | | | | | | | | | | | | | | |  |
|  | High |  | [D].TAKNVAKNVAVTLLDNLKcKITKAc.[-] |  | HCD |  | C19(Dehydro); C25(Dehydro) |  | 5.79 |  | 1.3e-3 |  | 1.9e-5 |  | 2.05 |  | 427.78 |  | 1.000 |  |  |  | C19(Dehydro): 100; C25(Dehydro): 100 |  |  |  |  |  |  |  | 0.58 |  | 1 |  | Sequest HT (3) |  | 3 |  | 886.49835 |  | -1.35 |  | 36.5715 |  | 9392 |  | 9392 |  | TRINITY\_DN6\_c27\_g1\_i1.p1\_ORF2; TRINITY\_DN10285\_c0\_g1\_i1.p1\_ORF1; TRINITY\_DN6\_c27\_g1\_i1.p1\_ORF1; TRINITY\_DN10285\_c0\_g1\_i1.p1 |  | Image |  | Peak List |  |
|  | | | | | | | | | | | | | | | | | | | | | | | | | | | | | | | | | | | | | | | | | | | | | | | | | | | | | |  |
|  | High |  | [A].KNVAVTLLDNLKcKITKAc.[-] |  | HCD |  | C13(Dehydro); C19(Dehydro) |  | 3.21 |  | 1.3e-3 |  | 4.9e-2 |  | 0.343 |  | 222.97 |  | 1.000 |  |  |  | C13(Dehydro): 100; C19(Dehydro): 100 |  |  |  |  |  |  |  | 0.41 |  | 1 |  | Sequest HT (3) |  | 4 |  | 519.04498 |  | 1.07 |  | 36.6273 |  | 9418 |  | 9418 |  | TRINITY\_DN6\_c27\_g1\_i1.p1\_ORF2; TRINITY\_DN10285\_c0\_g1\_i1.p1\_ORF1; TRINITY\_DN6\_c27\_g1\_i1.p1\_ORF1; TRINITY\_DN10285\_c0\_g1\_i1.p1 |  | Image |  | Peak List |  |
|  | | | | | | | | | | | | | | | | | | | | | | | | | | | | | | | | | | | | | | | | | | | | | | | | | | | | | |  |
|  | High |  | [D].TAKNVAKNVAVTLLDNLKcKITKAc.[-] |  | HCD |  | C19(Dehydro); C25(Dehydro) |  | 4.32 |  | 1.3e-3 |  | 2.6e-2 |  | 0.478 |  | 270.66 |  | 1.000 |  |  |  | C19(Dehydro): 100; C25(Dehydro): 100 |  |  |  |  |  |  |  | 0.53 |  | 1 |  | Sequest HT (3) |  | 4 |  | 665.12598 |  | -0.76 |  | 36.6570 |  | 9430 |  | 9430 |  | TRINITY\_DN6\_c27\_g1\_i1.p1\_ORF2; TRINITY\_DN10285\_c0\_g1\_i1.p1\_ORF1; TRINITY\_DN6\_c27\_g1\_i1.p1\_ORF1; TRINITY\_DN10285\_c0\_g1\_i1.p1 |  | Image |  | Peak List |  |
|  | | | | | | | | | | | | | | | | | | | | | | | | | | | | | | | | | | | | | | | | | | | | | | | | | | | | | |  |
|  | High |  | [A].KNVAVTLLDNLKcKITKAC.[-] |  | HCD |  | C13(Dehydro) |  | 4.32 |  | 2.4e-3 |  | 8.1e-2 |  | 0.231 |  | 266.65 |  | 0.500 |  |  |  | C13(Dehydro): 50; C19(Dehydro): 50 |  |  |  |  |  |  |  | 0.01 |  | 1 |  | Sequest HT (3) |  | 3 |  | 692.05737 |  | -2.96 |  | 36.6815 |  | 9443 |  | 9443 |  | TRINITY\_DN6\_c27\_g1\_i1.p1\_ORF2; TRINITY\_DN10285\_c0\_g1\_i1.p1\_ORF1; TRINITY\_DN6\_c27\_g1\_i1.p1\_ORF1; TRINITY\_DN10285\_c0\_g1\_i1.p1 |  | Image |  | Peak List |  |
|  | | | | | | | | | | | | | | | | | | | | | | | | | | | | | | | | | | | | | | | | | | | | | | | | | | | | | |  |
|  | High |  | [A].KNVAVTLLDNLKCKITKAc.[-] |  | HCD |  | C19(Dehydro) |  | 4.27 |  | 2.4e-3 |  | 7.9e-2 |  | 0.236 |  | 266.65 |  | 0.500 |  |  |  | C13(Dehydro): 50; C19(Dehydro): 50 |  |  |  |  |  |  |  |  |  | 2 |  | Sequest HT (3) |  | 3 |  | 692.05737 |  | -2.96 |  | 36.6815 |  | 9443 |  | 9443 |  | TRINITY\_DN6\_c27\_g1\_i1.p1\_ORF2; TRINITY\_DN10285\_c0\_g1\_i1.p1\_ORF1; TRINITY\_DN6\_c27\_g1\_i1.p1\_ORF1; TRINITY\_DN10285\_c0\_g1\_i1.p1 |  | Image |  | Peak List |  |
|  | | | | | | | | | | | | | | | | | | | | | | | | | | | | | | | | | | | | | | | | | | | | | | | | | | | | | |  |
|  | High |  | [D].TAKNVAKNVAVTLLDNLKcKITKAc.[-] |  | HCD |  | C19(Dehydro); C25(Dehydro) |  | 3.33 |  | 1.3e-3 |  | 5.5e-2 |  | 0.317 |  | 182.63 |  | 1.000 |  |  |  | C19(Dehydro): 100; C25(Dehydro): 100 |  |  |  |  |  |  |  | 0.43 |  | 1 |  | Sequest HT (3) |  | 5 |  | 532.30200 |  | -1.20 |  | 36.6954 |  | 9449 |  | 9449 |  | TRINITY\_DN6\_c27\_g1\_i1.p1\_ORF2; TRINITY\_DN10285\_c0\_g1\_i1.p1\_ORF1; TRINITY\_DN6\_c27\_g1\_i1.p1\_ORF1; TRINITY\_DN10285\_c0\_g1\_i1.p1 |  | Image |  | Peak List |  |
|  | | | | | | | | | | | | | | | | | | | | | | | | | | | | | | | | | | | | | | | | | | | | | | | | | | | | | |  |
|  | High |  | [D].TAKNVAKNVAVTLLDNLKcKITKAc.[-] |  | HCD |  | C19(Dehydro); C25(Dehydro) |  | 4.51 |  | 1.3e-3 |  | 2.0e-3 |  | 1.04 |  | 398.89 |  | 1.000 |  |  |  | C19(Dehydro): 100; C25(Dehydro): 100 |  |  |  |  |  |  |  | 0.65 |  | 1 |  | Sequest HT (3) |  | 3 |  | 886.49835 |  | -1.35 |  | 36.7181 |  | 9461 |  | 9461 |  | TRINITY\_DN6\_c27\_g1\_i1.p1\_ORF2; TRINITY\_DN10285\_c0\_g1\_i1.p1\_ORF1; TRINITY\_DN6\_c27\_g1\_i1.p1\_ORF1; TRINITY\_DN10285\_c0\_g1\_i1.p1 |  | Image |  | Peak List |  |
|  | | | | | | | | | | | | | | | | | | | | | | | | | | | | | | | | | | | | | | | | | | | | | | | | | | | | | |  |
|  | High |  | [D].TAKNVAKNVAVTLLDNLKcKITKAc.[-] |  | HCD |  | C19(Dehydro); C25(Dehydro) |  | 6.26 |  | 1.3e-3 |  | 2.6e-3 |  | 0.985 |  | 342.34 |  | 1.000 |  |  |  | C19(Dehydro): 100; C25(Dehydro): 100 |  |  |  |  |  |  |  | 0.58 |  | 1 |  | Sequest HT (3) |  | 4 |  | 665.12683 |  | 0.53 |  | 36.8062 |  | 9503 |  | 9503 |  | TRINITY\_DN6\_c27\_g1\_i1.p1\_ORF2; TRINITY\_DN10285\_c0\_g1\_i1.p1\_ORF1; TRINITY\_DN6\_c27\_g1\_i1.p1\_ORF1; TRINITY\_DN10285\_c0\_g1\_i1.p1 |  | Image |  | Peak List |  |
|  | | | | | | | | | | | | | | | | | | | | | | | | | | | | | | | | | | | | | | | | | | | | | | | | | | | | | |  |
|  | High |  | [D].TAKNVAKnVAVTLLDNLKcKITKAc.[-] |  | HCD |  | N8(Deamidated); C19(Dehydro); C25(Dehydro) |  | 5.48 |  | 3.1e-3 |  | 1.3e-1 |  | 0.114 |  | 236.52 |  | 1.000 |  | b1+(4): 415.23; b1+(5): 514.30; b1+(6): 585.34 |  | N8(Deamidated): 100; C19(Dehydro): 100; C25(Dehydro): 100 |  |  |  |  |  |  |  | 0.11 |  | 1 |  | Sequest HT (3) |  | 5 |  | 532.49945 |  | 0.02 |  | 37.4755 |  | 9834 |  | 9834 |  | TRINITY\_DN6\_c27\_g1\_i1.p1\_ORF2; TRINITY\_DN10285\_c0\_g1\_i1.p1\_ORF1; TRINITY\_DN6\_c27\_g1\_i1.p1\_ORF1; TRINITY\_DN10285\_c0\_g1\_i1.p1 |  | Image |  | Peak List |  |
|  | | | | | | | | | | | | | | | | | | | | | | | | | | | | | | | | | | | | | | | | | | | | | | | | | | | | | |  |
|  | High |  | [D].TAKNVAKNVAVTLLDnLKcKITKAc.[-] |  | HCD |  | N16(Deamidated); C19(Dehydro); C25(Dehydro) |  | 4.62 |  | 1.3e-3 |  | 1.0e-2 |  | 0.685 |  | 227.89 |  | 1.000 |  | b1+(9): 926.54; y1+(12): 1348.70; y1+(11): 1235.61; y1+(10): 1120.59; b2+(8): 414.24; b2+(9): 463.77; b2+(10): 499.29; y2+(14): 781.92; y2+(13): 731.39; y2+(12): 674.85; y2+(11): 618.31 |  | N16(Deamidated): 100; C19(Dehydro): 100; C25(Dehydro): 100 |  |  |  |  |  |  |  | 0.41 |  | 1 |  | Sequest HT (3) |  | 5 |  | 532.50012 |  | 1.28 |  | 37.7711 |  | 9965 |  | 9965 |  | TRINITY\_DN6\_c27\_g1\_i1.p1\_ORF2; TRINITY\_DN10285\_c0\_g1\_i1.p1\_ORF1; TRINITY\_DN6\_c27\_g1\_i1.p1\_ORF1; TRINITY\_DN10285\_c0\_g1\_i1.p1 |  | Image |  | Peak List |  |
|  | | | | | | | | | | | | | | | | | | | | | | | | | | | | | | | | | | | | | | | | | | | | | | | | | | | | | |  |
|  | High |  | [R].GFmDTAKNVAKNVAVTLLDNLKcKITKAc.[-] |  | HCD |  | M3(Oxidation); C23(Dehydro); C29(Dehydro) |  | 5.73 |  | 1.3e-3 |  | 1.0e-2 |  | 0.686 |  | 228.24 |  | 1.000 |  |  |  | M3(Oxidation): 100; C23(Dehydro): 100; C29(Dehydro): 100 |  |  |  |  |  |  |  | 0.53 |  | 1 |  | Sequest HT (3) |  | 5 |  | 625.53436 |  | 2.05 |  | 40.7191 |  | 11420 |  | 11420 |  | TRINITY\_DN6\_c27\_g1\_i1.p1\_ORF2; TRINITY\_DN10285\_c0\_g1\_i1.p1\_ORF1; TRINITY\_DN6\_c27\_g1\_i1.p1\_ORF1; TRINITY\_DN10285\_c0\_g1\_i1.p1 |  | Image |  | Peak List |  |
|  | | | | | | | | | | | | | | | | | | | | | | | | | | | | | | | | | | | | | | | | | | | | | | | | | | | | | |  |
|  | High |  | [R].GFmDTAKNVAKNVAVTLLDNLKcKITKAc.[-] |  | HCD |  | M3(Oxidation); C23(Dehydro); C29(Dehydro) |  | 4.83 |  | 1.3e-3 |  | 2.6e-3 |  | 0.986 |  | 366.54 |  | 1.000 |  |  |  | M3(Oxidation): 100; C23(Dehydro): 100; C29(Dehydro): 100 |  |  |  |  |  |  |  | 0.61 |  | 1 |  | Sequest HT (3) |  | 3 |  | 1041.88599 |  | 2.27 |  | 40.7574 |  | 11440 |  | 11440 |  | TRINITY\_DN6\_c27\_g1\_i1.p1\_ORF2; TRINITY\_DN10285\_c0\_g1\_i1.p1\_ORF1; TRINITY\_DN6\_c27\_g1\_i1.p1\_ORF1; TRINITY\_DN10285\_c0\_g1\_i1.p1 |  | Image |  | Peak List |  |
|  | | | | | | | | | | | | | | | | | | | | | | | | | | | | | | | | | | | | | | | | | | | | | | | | | | | | | |  |
|  | High |  | [R].GFmDTAKNVAKNVAVTLLDNLKcKITKAc.[-] |  | HCD |  | M3(Oxidation); C23(Dehydro); C29(Dehydro) |  | 5.54 |  | 1.3e-3 |  | 7.7e-3 |  | 0.749 |  | 337.16 |  | 1.000 |  |  |  | M3(Oxidation): 100; C23(Dehydro): 100; C29(Dehydro): 100 |  |  |  |  |  |  |  | 0.63 |  | 1 |  | Sequest HT (3) |  | 4 |  | 781.66608 |  | 1.97 |  | 40.8055 |  | 11461 |  | 11461 |  | TRINITY\_DN6\_c27\_g1\_i1.p1\_ORF2; TRINITY\_DN10285\_c0\_g1\_i1.p1\_ORF1; TRINITY\_DN6\_c27\_g1\_i1.p1\_ORF1; TRINITY\_DN10285\_c0\_g1\_i1.p1 |  | Image |  | Peak List |  |
|  | | | | | | | | | | | | | | | | | | | | | | | | | | | | | | | | | | | | | | | | | | | | | | | | | | | | | |  |
|  | High |  | [R].GFmDTAKNVAKNVAVTLLDNLKcKITKAc.[-] |  | HCD |  | M3(Oxidation); C23(Dehydro); C29(Dehydro) |  | 4.49 |  | 1.3e-3 |  | 6.4e-2 |  | 0.283 |  | 216.42 |  | 1.000 |  |  |  | M3(Oxidation): 100; C23(Dehydro): 100; C29(Dehydro): 100 |  |  |  |  |  |  |  | 0.51 |  | 1 |  | Sequest HT (3) |  | 5 |  | 625.53455 |  | 2.34 |  | 40.8597 |  | 11486 |  | 11486 |  | TRINITY\_DN6\_c27\_g1\_i1.p1\_ORF2; TRINITY\_DN10285\_c0\_g1\_i1.p1\_ORF1; TRINITY\_DN6\_c27\_g1\_i1.p1\_ORF1; TRINITY\_DN10285\_c0\_g1\_i1.p1 |  | Image |  | Peak List |  |
|  | | | | | | | | | | | | | | | | | | | | | | | | | | | | | | | | | | | | | | | | | | | | | | | | | | | | | |  |
|  | High |  | [R].GFmDTAKNVAKNVAVTLLDNLKcKITKAc.[-] |  | HCD |  | M3(Oxidation); C23(Dehydro); C29(Dehydro) |  | 5.07 |  | 1.3e-3 |  | 5.4e-4 |  | 1.33 |  | 348.96 |  | 1.000 |  |  |  | M3(Oxidation): 100; C23(Dehydro): 100; C29(Dehydro): 100 |  |  |  |  |  |  |  | 0.68 |  | 1 |  | Sequest HT (3) |  | 3 |  | 1041.88562 |  | 1.92 |  | 40.9056 |  | 11509 |  | 11509 |  | TRINITY\_DN6\_c27\_g1\_i1.p1\_ORF2; TRINITY\_DN10285\_c0\_g1\_i1.p1\_ORF1; TRINITY\_DN6\_c27\_g1\_i1.p1\_ORF1; TRINITY\_DN10285\_c0\_g1\_i1.p1 |  | Image |  | Peak List |  |
|  | | | | | | | | | | | | | | | | | | | | | | | | | | | | | | | | | | | | | | | | | | | | | | | | | | | | | |  |
|  | High |  | [R].GFmDTAKnVAKNVAVTLLDNLKcKITKAc.[-] |  | HCD |  | M3(Oxidation); N8(Deamidated); C23(Dehydro); C29(Dehydro) |  | 5.33 |  | 1.3e-3 |  | 3.4e-3 |  | 0.926 |  | 175.48 |  | 0.999 |  | b1+-H(4) C O S(13): 1329.68; b1+(8): 882.37; b1+(9): 981.43; b1+(10): 1052.47; y1+(12): 1347.71; y1+(11): 1234.63; y1+(10): 1119.60; y2+(18): 973.03; y2+(16): 866.48; y2+(14): 781.43; y2+(12): 674.36; y2+(11): 617.82; y2+(10): 560.30 |  | M3(Oxidation): 100; N8(Deamidated): 99.87; C23(Dehydro): 100; C29(Dehydro): 100 |  |  |  |  |  |  |  | 0.11 |  | 1 |  | Sequest HT (3) |  | 5 |  | 625.73151 |  | 2.59 |  | 41.1608 |  | 11617 |  | 11617 |  | TRINITY\_DN6\_c27\_g1\_i1.p1\_ORF2; TRINITY\_DN10285\_c0\_g1\_i1.p1\_ORF1; TRINITY\_DN6\_c27\_g1\_i1.p1\_ORF1; TRINITY\_DN10285\_c0\_g1\_i1.p1 |  | Image |  | Peak List |  |
|  | | | | | | | | | | | | | | | | | | | | | | | | | | | | | | | | | | | | | | | | | | | | | | | | | | | | | |  |
|  | High |  | [R].GFmDTAKnVAKNVAVTLLDNLKcKITKAc.[-] |  | HCD |  | M3(Oxidation); N8(Deamidated); C23(Dehydro); C29(Dehydro) |  | 4.44 |  | 1.3e-3 |  | 3.7e-3 |  | 0.907 |  | 359.76 |  | 1.000 |  | b1+-H(4) C O S(8): 818.37; b1+(8): 882.37; b1+(10): 1052.47 |  | M3(Oxidation): 100; N8(Deamidated): 100; C23(Dehydro): 100; C29(Dehydro): 100 |  |  |  |  |  |  |  | 0.06 |  | 1 |  | Sequest HT (3) |  | 3 |  | 1042.21033 |  | -1.25 |  | 41.1659 |  | 11621 |  | 11621 |  | TRINITY\_DN6\_c27\_g1\_i1.p1\_ORF2; TRINITY\_DN10285\_c0\_g1\_i1.p1\_ORF1; TRINITY\_DN6\_c27\_g1\_i1.p1\_ORF1; TRINITY\_DN10285\_c0\_g1\_i1.p1 |  | Image |  | Peak List |  |
|  | | | | | | | | | | | | | | | | | | | | | | | | | | | | | | | | | | | | | | | | | | | | | | | | | | | | | |  |
|  | High |  | [R].GFmDTAKNVAKNVAVTLLDnLKcKITKAc.[-] |  | HCD |  | M3(Oxidation); N20(Deamidated); C23(Dehydro); C29(Dehydro) |  | 4.51 |  | 1.3e-3 |  | 1.0e-3 |  | 1.18 |  | 235.67 |  | 1.000 |  | y1+(12): 1348.70; y1+(11): 1235.61; b2+(15): 781.90; y2+(16): 866.97; y2+(15): 831.45; y2+(14): 781.92; y2+(13): 731.39; y2+(12): 674.85; y2+(11): 618.31; y2+(10): 560.80 |  | M3(Oxidation): 100; N20(Deamidated): 100; C23(Dehydro): 100; C29(Dehydro): 100 |  |  |  |  |  |  |  | 0.21 |  | 1 |  | Sequest HT (3) |  | 5 |  | 625.73065 |  | 1.23 |  | 41.3121 |  | 11684 |  | 11684 |  | TRINITY\_DN6\_c27\_g1\_i1.p1\_ORF2; TRINITY\_DN10285\_c0\_g1\_i1.p1\_ORF1; TRINITY\_DN6\_c27\_g1\_i1.p1\_ORF1; TRINITY\_DN10285\_c0\_g1\_i1.p1 |  | Image |  | Peak List |  |
|  | | | | | | | | | | | | | | | | | | | | | | | | | | | | | | | | | | | | | | | | | | | | | | | | | | | | | |  |
|  | High |  | [R].GFMDTAKNVAKNVAVTLLDNLKcKITKAc.[-] |  | HCD |  | C23(Dehydro); C29(Dehydro) |  | 5.00 |  | 1.3e-3 |  | 1.1e-2 |  | 0.674 |  | 250.90 |  | 1.000 |  |  |  | C23(Dehydro): 100; C29(Dehydro): 100 |  |  |  |  |  |  |  | 0.43 |  | 1 |  | Sequest HT (3) |  | 5 |  | 622.33356 |  | -0.87 |  | 43.5691 |  | 12647 |  | 12647 |  | TRINITY\_DN6\_c27\_g1\_i1.p1\_ORF2; TRINITY\_DN10285\_c0\_g1\_i1.p1\_ORF1; TRINITY\_DN6\_c27\_g1\_i1.p1\_ORF1; TRINITY\_DN10285\_c0\_g1\_i1.p1 |  | Image |  | Peak List |  |
|  | | | | | | | | | | | | | | | | | | | | | | | | | | | | | | | | | | | | | | | | | | | | | | | | | | | | | |  |
|  | High |  | [R].GFMDTAKNVAKNVAVTLLDNLKcKITKAc.[-] |  | HCD |  | C23(Dehydro); C29(Dehydro) |  | 5.42 |  | 1.3e-3 |  | 8.1e-5 |  | 1.74 |  | 391.02 |  | 1.000 |  |  |  | C23(Dehydro): 100; C29(Dehydro): 100 |  |  |  |  |  |  |  | 0.53 |  | 1 |  | Sequest HT (3) |  | 3 |  | 1036.55444 |  | 2.38 |  | 43.5704 |  | 12648 |  | 12648 |  | TRINITY\_DN6\_c27\_g1\_i1.p1\_ORF2; TRINITY\_DN10285\_c0\_g1\_i1.p1\_ORF1; TRINITY\_DN6\_c27\_g1\_i1.p1\_ORF1; TRINITY\_DN10285\_c0\_g1\_i1.p1 |  | Image |  | Peak List |  |
|  | | | | | | | | | | | | | | | | | | | | | | | | | | | | | | | | | | | | | | | | | | | | | | | | | | | | | |  |
|  | High |  | [R].GFMDTAKNVAKNVAVTLLDNLKcKITKAc.[-] |  | HCD |  | C23(Dehydro); C29(Dehydro) |  | 5.62 |  | 1.3e-3 |  | 4.9e-6 |  | 2.35 |  | 440.82 |  | 1.000 |  |  |  | C23(Dehydro): 100; C29(Dehydro): 100 |  |  |  |  |  |  |  | 0.58 |  | 1 |  | Sequest HT (3) |  | 3 |  | 1036.55176 |  | -0.22 |  | 43.7108 |  | 12699 |  | 12699 |  | TRINITY\_DN6\_c27\_g1\_i1.p1\_ORF2; TRINITY\_DN10285\_c0\_g1\_i1.p1\_ORF1; TRINITY\_DN6\_c27\_g1\_i1.p1\_ORF1; TRINITY\_DN10285\_c0\_g1\_i1.p1 |  | Image |  | Peak List |  |
|  | | | | | | | | | | | | | | | | | | | | | | | | | | | | | | | | | | | | | | | | | | | | | | | | | | | | | |  |
|  | High |  | [R].GFMDTAKNVAKNVAVTLLDNLKcKITKAc.[-] |  | HCD |  | C23(Dehydro); C29(Dehydro) |  | 5.04 |  | 1.3e-3 |  | 3.3e-2 |  | 0.433 |  | 236.52 |  | 1.000 |  |  |  | C23(Dehydro): 100; C29(Dehydro): 100 |  |  |  |  |  |  |  | 0.49 |  | 1 |  | Sequest HT (3) |  | 5 |  | 622.33411 |  | 0.01 |  | 43.7133 |  | 12701 |  | 12701 |  | TRINITY\_DN6\_c27\_g1\_i1.p1\_ORF2; TRINITY\_DN10285\_c0\_g1\_i1.p1\_ORF1; TRINITY\_DN6\_c27\_g1\_i1.p1\_ORF1; TRINITY\_DN10285\_c0\_g1\_i1.p1 |  | Image |  | Peak List |  |
|  | | | | | | | | | | | | | | | | | | | | | | | | | | | | | | | | | | | | | | | | | | | | | | | | | | | | | |  |
|  | High |  | [R].GFMDTAKNVAKNVAVTLLDNLKcKITKAc.[-] |  | HCD |  | C23(Dehydro); C29(Dehydro) |  | 4.59 |  | 1.3e-3 |  | 4.4e-3 |  | 0.87 |  | 280.55 |  | 1.000 |  |  |  | C23(Dehydro): 100; C29(Dehydro): 100 |  |  |  |  |  |  |  | 0.62 |  | 1 |  | Sequest HT (3) |  | 4 |  | 777.66760 |  | 2.31 |  | 43.7767 |  | 12727 |  | 12727 |  | TRINITY\_DN6\_c27\_g1\_i1.p1\_ORF2; TRINITY\_DN10285\_c0\_g1\_i1.p1\_ORF1; TRINITY\_DN6\_c27\_g1\_i1.p1\_ORF1; TRINITY\_DN10285\_c0\_g1\_i1.p1 |  | Image |  | Peak List |  |
|  | | | | | | | | | | | | | | | | | | | | | | | | | | | | | | | | | | | | | | | | | | | | | | | | | | | | | |  |
|  | High |  | [R].GFMDTAKnVAKNVAVTLLDNLKcKITKAc.[-] |  | HCD |  | N8(Deamidated); C23(Dehydro); C29(Dehydro) |  | 6.26 |  | 1.3e-3 |  | 5.7e-5 |  | 1.82 |  | 325.98 |  | 0.982 |  | b1+(8): 866.37; b1+(10): 1036.48 |  | N8(Deamidated): 98.21; C23(Dehydro): 100; C29(Dehydro): 100 |  |  |  |  |  |  |  | 0.01 |  | 1 |  | Sequest HT (3) |  | 3 |  | 1036.88062 |  | 0.61 |  | 44.2794 |  | 12933 |  | 12933 |  | TRINITY\_DN6\_c27\_g1\_i1.p1\_ORF2; TRINITY\_DN10285\_c0\_g1\_i1.p1\_ORF1; TRINITY\_DN6\_c27\_g1\_i1.p1\_ORF1; TRINITY\_DN10285\_c0\_g1\_i1.p1 |  | Image |  | Peak List |  |
|  | | | | | | | | | | | | | | | | | | | | | | | | | | | | | | | | | | | | | | | | | | | | | | | | | | | | | |  |
|  | High |  | [R].GFMDTAKNVAKnVAVTLLDNLKcKITKAc.[-] |  | HCD |  | N12(Deamidated); C23(Dehydro); C29(Dehydro) |  | 6.19 |  | 1.3e-3 |  | 9.0e-5 |  | 1.72 |  | 308.59 |  | 0.018 |  | b1+(8): 866.37; b1+(10): 1036.48 |  | N8(Deamidated): 98.21; C23(Dehydro): 100; C29(Dehydro): 100 |  |  |  |  |  |  |  |  |  | 2 |  | Sequest HT (3) |  | 3 |  | 1036.88062 |  | 0.61 |  | 44.2794 |  | 12933 |  | 12933 |  | TRINITY\_DN6\_c27\_g1\_i1.p1\_ORF2; TRINITY\_DN10285\_c0\_g1\_i1.p1\_ORF1; TRINITY\_DN6\_c27\_g1\_i1.p1\_ORF1; TRINITY\_DN10285\_c0\_g1\_i1.p1 |  | Image |  | Peak List |  |
|  | | | | | | | | | | | | | | | | | | | | | | | | | | | | | | | | | | | | | | | | | | | | | | | | | | | | | |  |
|  | High |  | [R].GFMDTAKnVAKNVAVTLLDNLKcKITKAc.[-] |  | HCD |  | N8(Deamidated); C23(Dehydro); C29(Dehydro) |  | 4.10 |  | 2.4e-3 |  | 7.0e-2 |  | 0.264 |  | 139.06 |  | 0.999 |  | b1+(9): 965.44; b1+(10): 1036.48 |  | N8(Deamidated): 99.9; C23(Dehydro): 100; C29(Dehydro): 100 |  |  |  |  |  |  |  | 0.12 |  | 1 |  | Sequest HT (3) |  | 5 |  | 622.53058 |  | -0.52 |  | 44.3434 |  | 12959 |  | 12959 |  | TRINITY\_DN6\_c27\_g1\_i1.p1\_ORF2; TRINITY\_DN10285\_c0\_g1\_i1.p1\_ORF1; TRINITY\_DN6\_c27\_g1\_i1.p1\_ORF1; TRINITY\_DN10285\_c0\_g1\_i1.p1 |  | Image |  | Peak List |  |
|  | | | | | | | | | | | | | | | | | | | | | | | | | | | | | | | | | | | | | | | | | | | | | | | | | | | | | |  |
|  | High |  | [R].GFMDTAKnVAKNVAVTLLDNLKcKITKAc.[-] |  | HCD |  | N8(Deamidated); C23(Dehydro); C29(Dehydro) |  | 5.15 |  | 1.3e-3 |  | 4.1e-3 |  | 0.884 |  | 259.48 |  | 1.000 |  | b1+(9): 965.44; b1+(10): 1036.48; y2+(20): 1072.60 |  | N8(Deamidated): 99.96; C23(Dehydro): 100; C29(Dehydro): 100 |  |  |  |  |  |  |  | 0.17 |  | 1 |  | Sequest HT (3) |  | 4 |  | 777.91248 |  | 0.86 |  | 44.3683 |  | 12972 |  | 12972 |  | TRINITY\_DN6\_c27\_g1\_i1.p1\_ORF2; TRINITY\_DN10285\_c0\_g1\_i1.p1\_ORF1; TRINITY\_DN6\_c27\_g1\_i1.p1\_ORF1; TRINITY\_DN10285\_c0\_g1\_i1.p1 |  | Image |  | Peak List |  |
|  | | | | | | | | | | | | | | | | | | | | | | | | | | | | | | | | | | | | | | | | | | | | | | | | | | | | | |  |
|  | High |  | [R].GFMDTAKnVAKNVAVTLLDNLKcKITKAc.[-] |  | HCD |  | N8(Deamidated); C23(Dehydro); C29(Dehydro) |  | 5.75 |  | 1.3e-3 |  | 3.3e-4 |  | 1.43 |  | 260.54 |  | 0.978 |  | b1+(10): 1036.48; b1+(11): 1164.57 |  | N8(Deamidated): 97.85; C23(Dehydro): 100; C29(Dehydro): 100 |  |  |  |  |  |  |  | 0.07 |  | 1 |  | Sequest HT (3) |  | 4 |  | 777.91180 |  | -0.01 |  | 44.3836 |  | 12981 |  | 12981 |  | TRINITY\_DN6\_c27\_g1\_i1.p1\_ORF2; TRINITY\_DN10285\_c0\_g1\_i1.p1\_ORF1; TRINITY\_DN6\_c27\_g1\_i1.p1\_ORF1; TRINITY\_DN10285\_c0\_g1\_i1.p1 |  | Image |  | Peak List |  |
|  | | | | | | | | | | | | | | | | | | | | | | | | | | | | | | | | | | | | | | | | | | | | | | | | | | | | | |  |
|  | High |  | [R].GFMDTAKNVAKnVAVTLLDNLKcKITKAc.[-] |  | HCD |  | N12(Deamidated); C23(Dehydro); C29(Dehydro) |  | 5.80 |  | 1.3e-3 |  | 1.0e-3 |  | 1.19 |  | 370.19 |  | 0.000 |  | b1+(8): 866.37; b1+(10): 1036.48; b1+(11): 1164.57 |  | N8(Deamidated): 99.98; C23(Dehydro): 100; C29(Dehydro): 100 |  |  |  |  |  |  |  | 0.01 |  | 1 |  | Sequest HT (3) |  | 3 |  | 1036.87830 |  | -1.63 |  | 44.4198 |  | 12996 |  | 12996 |  | TRINITY\_DN6\_c27\_g1\_i1.p1\_ORF2; TRINITY\_DN10285\_c0\_g1\_i1.p1\_ORF1; TRINITY\_DN6\_c27\_g1\_i1.p1\_ORF1; TRINITY\_DN10285\_c0\_g1\_i1.p1 |  | Image |  | Peak List |  |
|  | | | | | | | | | | | | | | | | | | | | | | | | | | | | | | | | | | | | | | | | | | | | | | | | | | | | | |  |
|  | High |  | [R].GFMDTAKnVAKNVAVTLLDNLKcKITKAc.[-] |  | HCD |  | N8(Deamidated); C23(Dehydro); C29(Dehydro) |  | 5.77 |  | 1.3e-3 |  | 6.4e-4 |  | 1.29 |  | 407.12 |  | 1.000 |  | b1+(8): 866.37; b1+(10): 1036.48; b1+(11): 1164.57 |  | N8(Deamidated): 99.98; C23(Dehydro): 100; C29(Dehydro): 100 |  |  |  |  |  |  |  |  |  | 2 |  | Sequest HT (3) |  | 3 |  | 1036.87830 |  | -1.63 |  | 44.4198 |  | 12996 |  | 12996 |  | TRINITY\_DN6\_c27\_g1\_i1.p1\_ORF2; TRINITY\_DN10285\_c0\_g1\_i1.p1\_ORF1; TRINITY\_DN6\_c27\_g1\_i1.p1\_ORF1; TRINITY\_DN10285\_c0\_g1\_i1.p1 |  | Image |  | Peak List |  |
|  | | | | | | | | | | | | | | | | | | | | | | | | | | | | | | | | | | | | | | | | | | | | | | | | | | | | | |  |
|  | High |  | [R].GFMDTAKNVAKNVAVTLLDnLKcKITKAc.[-] |  | HCD |  | N20(Deamidated); C23(Dehydro); C29(Dehydro) |  | 5.61 |  | 1.3e-3 |  | 3.3e-4 |  | 1.44 |  | 289.01 |  | 1.000 |  | y1+(14): 1562.83; y1+(13): 1461.78; y1+(12): 1348.70; y1+(11): 1235.61; y1+(10): 1120.59; b2+(12): 639.32; b2+(13): 688.85; b2+(15): 773.91; y2+(16): 866.97; y2+(13): 731.39; y2+(12): 674.85; y2+(10): 560.80 |  | N20(Deamidated): 100; C23(Dehydro): 100; C29(Dehydro): 100 |  |  |  |  |  |  |  | 0.40 |  | 1 |  | Sequest HT (3) |  | 4 |  | 777.91119 |  | -0.79 |  | 44.5888 |  | 13068 |  | 13068 |  | TRINITY\_DN6\_c27\_g1\_i1.p1\_ORF2; TRINITY\_DN10285\_c0\_g1\_i1.p1\_ORF1; TRINITY\_DN6\_c27\_g1\_i1.p1\_ORF1; TRINITY\_DN10285\_c0\_g1\_i1.p1 |  | Image |  | Peak List |  |
|  | | | | | | | | | | | | | | | | | | | | | | | | | | | | | | | | | | | | | | | | | | | | | | | | | | | | | |  |
|  | High |  | [R].GFMDTAKNVAKNVAVTLLDnLKcKITKAc.[-] |  | HCD |  | N20(Deamidated); C23(Dehydro); C29(Dehydro) |  | 4.60 |  | 1.3e-3 |  | 3.4e-4 |  | 1.43 |  | 333.04 |  | 1.000 |  | b1+(14): 1447.74; y1+(16): 1732.93; y1+(15): 1661.90; y1+(14): 1562.83; y1+(13): 1461.78; y1+(12): 1348.70; y1+(11): 1235.61; y1+(10): 1120.59; b2+(12): 639.32; b2+(13): 688.85; b2+(14): 724.37; y2+(16): 866.97; y2+(15): 831.45; y2+(14): 781.92; y2+(12): 674.85 |  | N20(Deamidated): 100; C23(Dehydro): 100; C29(Dehydro): 100 |  |  |  |  |  |  |  | 0.44 |  | 1 |  | Sequest HT (3) |  | 3 |  | 1036.87854 |  | -1.40 |  | 44.6736 |  | 13101 |  | 13101 |  | TRINITY\_DN6\_c27\_g1\_i1.p1\_ORF2; TRINITY\_DN10285\_c0\_g1\_i1.p1\_ORF1; TRINITY\_DN6\_c27\_g1\_i1.p1\_ORF1; TRINITY\_DN10285\_c0\_g1\_i1.p1 |  | Image |  | Peak List |  |
|  | | | | | | | | | | | | | | | | | | | | | | | | | | | | | | | | | | | | | | | | | | | | | | | | | | | | | |  |
|  | **TRINITY\_DN2658\_c0\_g2\_i1.p1 - TRINITY\_DN2658\_c0\_g2\_i1.p1** | | | | | | | | | | | | | | | | | | | | | | | | | | | | | | | | | | | | | | | | | | | | | | | | | | | |  |  |
|  | | | | | | | | | | | | | | | | | | | | | | | | | | | | | | | | | | | | | | | | | | | | | | | | | | | | | |  |
|  | High |  | [GS].KSAAQSVLKTLScKLSNDc.[-] |  | HCD |  | C13(Dehydro); C19(Dehydro) |  | 3.00 |  | 1.3e-3 |  | 4.8e-2 |  | 0.347 |  | 189.80 |  | 1.000 |  |  |  | C13(Dehydro): 100; C19(Dehydro): 100 |  |  |  |  |  |  |  | 0.60 |  | 1 |  | Sequest HT (3) |  | 3 |  | 665.33936 |  | -0.51 |  | 32.3240 |  | 7465 |  | 7465 |  | TRINITY\_DN0\_c1\_g1\_i15.p1; TRINITY\_DN0\_c1\_g1\_i23.p1; TRINITY\_DN0\_c1\_g1\_i11.p1; TRINITY\_DN45\_c27\_g1\_i1.p1; TRINITY\_DN2658\_c0\_g2\_i1.p1\_ORF2; TRINITY\_DN0\_c1\_g1\_i10.p1; TRINITY\_DN2658\_c0\_g2\_i1.p1\_ORF1; TRINITY\_DN2658\_c0\_g2\_i1.p1 |  | Image |  | Peak List |  |
|  | | | | | | | | | | | | | | | | | | | | | | | | | | | | | | | | | | | | | | | | | | | | | | | | | | | | | |  |
|  | High |  | [GS].KSAAQSVLKTLScKLSNDc.[-] |  | HCD |  | C13(Dehydro); C19(Dehydro) |  | 4.38 |  | 1.3e-3 |  | 4.1e-2 |  | 0.382 |  | 219.12 |  | 1.000 |  |  |  | C13(Dehydro): 100; C19(Dehydro): 100 |  |  |  |  |  |  |  | 0.58 |  | 1 |  | Sequest HT (3) |  | 4 |  | 499.25656 |  | -0.05 |  | 32.3418 |  | 7474 |  | 7474 |  | TRINITY\_DN0\_c1\_g1\_i15.p1; TRINITY\_DN0\_c1\_g1\_i23.p1; TRINITY\_DN0\_c1\_g1\_i11.p1; TRINITY\_DN45\_c27\_g1\_i1.p1; TRINITY\_DN2658\_c0\_g2\_i1.p1\_ORF2; TRINITY\_DN0\_c1\_g1\_i10.p1; TRINITY\_DN2658\_c0\_g2\_i1.p1\_ORF1; TRINITY\_DN2658\_c0\_g2\_i1.p1 |  | Image |  | Peak List |  |
|  | | | | | | | | | | | | | | | | | | | | | | | | | | | | | | | | | | | | | | | | | | | | | | | | | | | | | |  |
|  | High |  | [GS].KSAAQSVLKTLScKLSNDc.[-] |  | HCD |  | C13(Dehydro); C19(Dehydro) |  | 1.73 |  | 3.1e-3 |  | 1.1e-1 |  | 0.15 |  | 202.39 |  | 1.000 |  |  |  | C13(Dehydro): 100; C19(Dehydro): 100 |  |  |  |  |  |  |  | 0.46 |  | 1 |  | Sequest HT (3) |  | 2 |  | 997.50824 |  | 2.35 |  | 32.3444 |  | 7475 |  | 7475 |  | TRINITY\_DN0\_c1\_g1\_i15.p1; TRINITY\_DN0\_c1\_g1\_i23.p1; TRINITY\_DN0\_c1\_g1\_i11.p1; TRINITY\_DN45\_c27\_g1\_i1.p1; TRINITY\_DN2658\_c0\_g2\_i1.p1\_ORF2; TRINITY\_DN0\_c1\_g1\_i10.p1; TRINITY\_DN2658\_c0\_g2\_i1.p1\_ORF1; TRINITY\_DN2658\_c0\_g2\_i1.p1 |  | Image |  | Peak List |  |
|  | | | | | | | | | | | | | | | | | | | | | | | | | | | | | | | | | | | | | | | | | | | | | | | | | | | | | |  |
|  | High |  | [GS].KSAAQSVLKTLScKLSNDc.[-] |  | HCD |  | C13(Dehydro); C19(Dehydro) |  | 2.27 |  | 1.3e-3 |  | 4.4e-3 |  | 0.871 |  | 314.72 |  | 1.000 |  |  |  | C13(Dehydro): 100; C19(Dehydro): 100 |  |  |  |  |  |  |  | 0.54 |  | 1 |  | Sequest HT (3) |  | 2 |  | 997.50623 |  | 0.33 |  | 32.4897 |  | 7536 |  | 7536 |  | TRINITY\_DN0\_c1\_g1\_i15.p1; TRINITY\_DN0\_c1\_g1\_i23.p1; TRINITY\_DN0\_c1\_g1\_i11.p1; TRINITY\_DN45\_c27\_g1\_i1.p1; TRINITY\_DN2658\_c0\_g2\_i1.p1\_ORF2; TRINITY\_DN0\_c1\_g1\_i10.p1; TRINITY\_DN2658\_c0\_g2\_i1.p1\_ORF1; TRINITY\_DN2658\_c0\_g2\_i1.p1 |  | Image |  | Peak List |  |
|  | | | | | | | | | | | | | | | | | | | | | | | | | | | | | | | | | | | | | | | | | | | | | | | | | | | | | |  |
|  | High |  | [G].ASKSAAQSVLKTLScKLSNDc.[-] |  | HCD |  | C15(Dehydro); C21(Dehydro) |  | 1.92 |  | 1.3e-3 |  | 4.3e-2 |  | 0.371 |  | 134.23 |  | 1.000 |  |  |  | C15(Dehydro): 100; C21(Dehydro): 100 |  |  |  |  |  |  |  | 0.48 |  | 1 |  | Sequest HT (3) |  | 2 |  | 1076.54077 |  | 0.28 |  | 32.6164 |  | 7593 |  | 7593 |  | TRINITY\_DN2658\_c0\_g2\_i1.p1\_ORF2; TRINITY\_DN2658\_c0\_g2\_i1.p1\_ORF1; TRINITY\_DN2658\_c0\_g2\_i1.p1 |  | Image |  | Peak List |  |
|  | | | | | | | | | | | | | | | | | | | | | | | | | | | | | | | | | | | | | | | | | | | | | | | | | | | | | |  |
|  | High |  | [GS].KSAAQSVLKTLScKLSnDc.[-] |  | HCD |  | C13(Dehydro); N17(Deamidated); C19(Dehydro) |  | 2.66 |  | 3.1e-3 |  | 1.1e-1 |  | 0.162 |  | 164.04 |  | 1.000 |  |  |  | C13(Dehydro): 100; N17(Deamidated): 100; C19(Dehydro): 100 |  |  |  |  |  |  |  | 0.58 |  | 1 |  | Sequest HT (3) |  | 3 |  | 665.66742 |  | -0.42 |  | 32.6438 |  | 7606 |  | 7606 |  | TRINITY\_DN0\_c1\_g1\_i15.p1; TRINITY\_DN0\_c1\_g1\_i23.p1; TRINITY\_DN0\_c1\_g1\_i11.p1; TRINITY\_DN45\_c27\_g1\_i1.p1; TRINITY\_DN2658\_c0\_g2\_i1.p1\_ORF2; TRINITY\_DN0\_c1\_g1\_i10.p1; TRINITY\_DN2658\_c0\_g2\_i1.p1\_ORF1; TRINITY\_DN2658\_c0\_g2\_i1.p1 |  | Image |  | Peak List |  |
|  | | | | | | | | | | | | | | | | | | | | | | | | | | | | | | | | | | | | | | | | | | | | | | | | | | | | | |  |
|  | High |  | [GS].KSAAQSVLKTLScKLSnDc.[-] |  | HCD |  | C13(Dehydro); N17(Deamidated); C19(Dehydro) |  | 2.84 |  | 1.3e-3 |  | 2.6e-2 |  | 0.48 |  | 247.07 |  | 1.000 |  |  |  | C13(Dehydro): 100; N17(Deamidated): 100; C19(Dehydro): 100 |  |  |  |  |  |  |  | 0.48 |  | 1 |  | Sequest HT (3) |  | 2 |  | 997.99811 |  | 0.20 |  | 32.6558 |  | 7612 |  | 7612 |  | TRINITY\_DN0\_c1\_g1\_i15.p1; TRINITY\_DN0\_c1\_g1\_i23.p1; TRINITY\_DN0\_c1\_g1\_i11.p1; TRINITY\_DN45\_c27\_g1\_i1.p1; TRINITY\_DN2658\_c0\_g2\_i1.p1\_ORF2; TRINITY\_DN0\_c1\_g1\_i10.p1; TRINITY\_DN2658\_c0\_g2\_i1.p1\_ORF1; TRINITY\_DN2658\_c0\_g2\_i1.p1 |  | Image |  | Peak List |  |
|  | | | | | | | | | | | | | | | | | | | | | | | | | | | | | | | | | | | | | | | | | | | | | | | | | | | | | |  |
|  | High |  | [GS].KSAAQSVLKTLScKLSnDc.[-] |  | HCD |  | C13(Dehydro); N17(Deamidated); C19(Dehydro) |  | 2.77 |  | 1.3e-3 |  | 4.1e-2 |  | 0.383 |  | 217.58 |  | 1.000 |  |  |  | C13(Dehydro): 100; N17(Deamidated): 100; C19(Dehydro): 100 |  |  |  |  |  |  |  | 0.61 |  | 1 |  | Sequest HT (3) |  | 4 |  | 499.50272 |  | 0.25 |  | 32.6665 |  | 7616 |  | 7616 |  | TRINITY\_DN0\_c1\_g1\_i15.p1; TRINITY\_DN0\_c1\_g1\_i23.p1; TRINITY\_DN0\_c1\_g1\_i11.p1; TRINITY\_DN45\_c27\_g1\_i1.p1; TRINITY\_DN2658\_c0\_g2\_i1.p1\_ORF2; TRINITY\_DN0\_c1\_g1\_i10.p1; TRINITY\_DN2658\_c0\_g2\_i1.p1\_ORF1; TRINITY\_DN2658\_c0\_g2\_i1.p1 |  | Image |  | Peak List |  |
|  | | | | | | | | | | | | | | | | | | | | | | | | | | | | | | | | | | | | | | | | | | | | | | | | | | | | | |  |
|  | High |  | [A].AQSVLKTLScKLSnDc.[-] |  | HCD |  | C10(Dehydro); N14(Deamidated); C16(Dehydro) |  | 2.69 |  | 2.4e-3 |  | 7.1e-2 |  | 0.26 |  | 200.69 |  | 1.000 |  |  |  | C10(Dehydro): 100; N14(Deamidated): 100; C16(Dehydro): 100 |  |  |  |  |  |  |  | 0.43 |  | 1 |  | Sequest HT (3) |  | 2 |  | 854.91431 |  | -1.81 |  | 32.8138 |  | 7680 |  | 7680 |  | TRINITY\_DN0\_c1\_g1\_i15.p1; TRINITY\_DN0\_c1\_g1\_i23.p1; TRINITY\_DN0\_c1\_g1\_i11.p1; TRINITY\_DN45\_c27\_g1\_i1.p1; TRINITY\_DN2658\_c0\_g2\_i1.p1\_ORF2; TRINITY\_DN0\_c1\_g1\_i10.p1; TRINITY\_DN2658\_c0\_g2\_i1.p1\_ORF1; TRINITY\_DN2658\_c0\_g2\_i1.p1 |  | Image |  | Peak List |  |
|  | | | | | | | | | | | | | | | | | | | | | | | | | | | | | | | | | | | | | | | | | | | | | | | | | | | | | |  |
|  | High |  | [S].AAQSVLKTLScKLSNDc.[-] |  | HCD |  | C11(Dehydro); C17(Dehydro) |  | 3.29 |  | 1.3e-3 |  | 8.3e-3 |  | 0.731 |  | 248.78 |  | 1.000 |  |  |  | C11(Dehydro): 100; C17(Dehydro): 100 |  |  |  |  |  |  |  | 0.56 |  | 1 |  | Sequest HT (3) |  | 2 |  | 889.94220 |  | -0.23 |  | 33.0598 |  | 7783 |  | 7783 |  | TRINITY\_DN0\_c1\_g1\_i15.p1; TRINITY\_DN0\_c1\_g1\_i23.p1; TRINITY\_DN0\_c1\_g1\_i11.p1; TRINITY\_DN45\_c27\_g1\_i1.p1; TRINITY\_DN2658\_c0\_g2\_i1.p1\_ORF2; TRINITY\_DN0\_c1\_g1\_i10.p1; TRINITY\_DN2658\_c0\_g2\_i1.p1\_ORF1; TRINITY\_DN2658\_c0\_g2\_i1.p1 |  | Image |  | Peak List |  |
|  | | | | | | | | | | | | | | | | | | | | | | | | | | | | | | | | | | | | | | | | | | | | | | | | | | | | | |  |
|  | High |  | [R].GVLGTVKNLLIGASKSAAQ.[S] |  | HCD |  |  |  | 2.85 |  | 1.3e-3 |  | 1.8e-2 |  | 0.567 |  |  |  |  |  |  |  |  |  |  |  |  |  |  |  | 0.58 |  | 1 |  | Sequest HT (3) |  | 2 |  | 914.03851 |  | -0.04 |  | 33.5986 |  | 8006 |  | 8006 |  | TRINITY\_DN2658\_c0\_g2\_i1.p1\_ORF2; TRINITY\_DN2658\_c0\_g2\_i1.p1\_ORF1; TRINITY\_DN2658\_c0\_g2\_i1.p1 |  | Image |  | Peak List |  |
|  | | | | | | | | | | | | | | | | | | | | | | | | | | | | | | | | | | | | | | | | | | | | | | | | | | | | | |  |
|  | High |  | [R].GVLGTVKnLLIGASKSAAQ.[S] |  | HCD |  | N8(Deamidated) |  | 3.31 |  | 1.3e-3 |  | 7.5e-3 |  | 0.755 |  | 220.37 |  | 1.000 |  |  |  | N8(Deamidated): 100 |  |  |  |  |  |  |  | 0.58 |  | 1 |  | Sequest HT (3) |  | 2 |  | 914.52917 |  | -1.51 |  | 34.8205 |  | 8559 |  | 8559 |  | TRINITY\_DN2658\_c0\_g2\_i1.p1\_ORF2; TRINITY\_DN2658\_c0\_g2\_i1.p1\_ORF1; TRINITY\_DN2658\_c0\_g2\_i1.p1 |  | Image |  | Peak List |  |
|  | | | | | | | | | | | | | | | | | | | | | | | | | | | | | | | | | | | | | | | | | | | | | | | | | | | | | |  |
|  | High |  | [K].SAAQSVLKTLScKLSNDc.[-] |  | HCD |  | C12(Dehydro); C18(Dehydro) |  | 4.40 |  | 1.3e-3 |  | 3.7e-3 |  | 0.906 |  | 350.81 |  | 1.000 |  |  |  | C12(Dehydro): 100; C18(Dehydro): 100 |  |  |  |  |  |  |  | 0.49 |  | 1 |  | Sequest HT (3) |  | 2 |  | 933.45862 |  | 0.21 |  | 35.8588 |  | 9040 |  | 9040 |  | TRINITY\_DN0\_c1\_g1\_i15.p1; TRINITY\_DN0\_c1\_g1\_i23.p1; TRINITY\_DN0\_c1\_g1\_i11.p1; TRINITY\_DN45\_c27\_g1\_i1.p1; TRINITY\_DN2658\_c0\_g2\_i1.p1\_ORF2; TRINITY\_DN0\_c1\_g1\_i10.p1; TRINITY\_DN2658\_c0\_g2\_i1.p1\_ORF1; TRINITY\_DN2658\_c0\_g2\_i1.p1 |  | Image |  | Peak List |  |
|  | | | | | | | | | | | | | | | | | | | | | | | | | | | | | | | | | | | | | | | | | | | | | | | | | | | | | |  |
|  | High |  | [K].SAAQSVLKTLScKLSNDc.[-] |  | HCD |  | C12(Dehydro); C18(Dehydro) |  | 2.79 |  | 3.1e-3 |  | 1.1e-1 |  | 0.155 |  | 206.05 |  | 1.000 |  |  |  | C12(Dehydro): 100; C18(Dehydro): 100 |  |  |  |  |  |  |  | 0.51 |  | 1 |  | Sequest HT (3) |  | 3 |  | 622.64099 |  | -0.61 |  | 35.8729 |  | 9047 |  | 9047 |  | TRINITY\_DN0\_c1\_g1\_i15.p1; TRINITY\_DN0\_c1\_g1\_i23.p1; TRINITY\_DN0\_c1\_g1\_i11.p1; TRINITY\_DN45\_c27\_g1\_i1.p1; TRINITY\_DN2658\_c0\_g2\_i1.p1\_ORF2; TRINITY\_DN0\_c1\_g1\_i10.p1; TRINITY\_DN2658\_c0\_g2\_i1.p1\_ORF1; TRINITY\_DN2658\_c0\_g2\_i1.p1 |  | Image |  | Peak List |  |
|  | | | | | | | | | | | | | | | | | | | | | | | | | | | | | | | | | | | | | | | | | | | | | | | | | | | | | |  |
|  | High |  | [K].SAAQSVLKTLScKLSNDc.[-] |  | HCD |  | C12(Dehydro); C18(Dehydro) |  | 2.92 |  | 1.3e-3 |  | 4.5e-2 |  | 0.363 |  | 216.35 |  | 1.000 |  |  |  | C12(Dehydro): 100; C18(Dehydro): 100 |  |  |  |  |  |  |  | 0.52 |  | 1 |  | Sequest HT (3) |  | 3 |  | 622.64154 |  | 0.27 |  | 36.0172 |  | 9114 |  | 9114 |  | TRINITY\_DN0\_c1\_g1\_i15.p1; TRINITY\_DN0\_c1\_g1\_i23.p1; TRINITY\_DN0\_c1\_g1\_i11.p1; TRINITY\_DN45\_c27\_g1\_i1.p1; TRINITY\_DN2658\_c0\_g2\_i1.p1\_ORF2; TRINITY\_DN0\_c1\_g1\_i10.p1; TRINITY\_DN2658\_c0\_g2\_i1.p1\_ORF1; TRINITY\_DN2658\_c0\_g2\_i1.p1 |  | Image |  | Peak List |  |
|  | | | | | | | | | | | | | | | | | | | | | | | | | | | | | | | | | | | | | | | | | | | | | | | | | | | | | |  |
|  | High |  | [K].SAAQSVLKTLScKLSNDc.[-] |  | HCD |  | C12(Dehydro); C18(Dehydro) |  | 2.87 |  | 1.3e-3 |  | 4.0e-2 |  | 0.385 |  | 182.50 |  | 1.000 |  |  |  | C12(Dehydro): 100; C18(Dehydro): 100 |  |  |  |  |  |  |  | 0.63 |  | 1 |  | Sequest HT (3) |  | 2 |  | 933.45795 |  | -0.50 |  | 36.0198 |  | 9116 |  | 9116 |  | TRINITY\_DN0\_c1\_g1\_i15.p1; TRINITY\_DN0\_c1\_g1\_i23.p1; TRINITY\_DN0\_c1\_g1\_i11.p1; TRINITY\_DN45\_c27\_g1\_i1.p1; TRINITY\_DN2658\_c0\_g2\_i1.p1\_ORF2; TRINITY\_DN0\_c1\_g1\_i10.p1; TRINITY\_DN2658\_c0\_g2\_i1.p1\_ORF1; TRINITY\_DN2658\_c0\_g2\_i1.p1 |  | Image |  | Peak List |  |
|  | | | | | | | | | | | | | | | | | | | | | | | | | | | | | | | | | | | | | | | | | | | | | | | | | | | | | |  |
|  | High |  | [K].SAAQSVLKTLScKLSnDc.[-] |  | HCD |  | C12(Dehydro); N16(Deamidated); C18(Dehydro) |  | 3.98 |  | 1.3e-3 |  | 5.1e-2 |  | 0.332 |  | 248.98 |  | 1.000 |  |  |  | C12(Dehydro): 100; N16(Deamidated): 100; C18(Dehydro): 100 |  |  |  |  |  |  |  | 0.55 |  | 1 |  | Sequest HT (3) |  | 3 |  | 622.96918 |  | -0.32 |  | 36.1850 |  | 9196 |  | 9196 |  | TRINITY\_DN0\_c1\_g1\_i15.p1; TRINITY\_DN0\_c1\_g1\_i23.p1; TRINITY\_DN0\_c1\_g1\_i11.p1; TRINITY\_DN45\_c27\_g1\_i1.p1; TRINITY\_DN2658\_c0\_g2\_i1.p1\_ORF2; TRINITY\_DN0\_c1\_g1\_i10.p1; TRINITY\_DN2658\_c0\_g2\_i1.p1\_ORF1; TRINITY\_DN2658\_c0\_g2\_i1.p1 |  | Image |  | Peak List |  |
|  | | | | | | | | | | | | | | | | | | | | | | | | | | | | | | | | | | | | | | | | | | | | | | | | | | | | | |  |
|  | High |  | [K].SAAQSVLKTLScKLSnDc.[-] |  | HCD |  | C12(Dehydro); N16(Deamidated); C18(Dehydro) |  | 4.33 |  | 1.3e-3 |  | 1.3e-3 |  | 1.13 |  | 325.14 |  | 1.000 |  |  |  | C12(Dehydro): 100; N16(Deamidated): 100; C18(Dehydro): 100 |  |  |  |  |  |  |  | 0.57 |  | 1 |  | Sequest HT (3) |  | 2 |  | 933.95013 |  | -0.31 |  | 36.1949 |  | 9203 |  | 9203 |  | TRINITY\_DN0\_c1\_g1\_i15.p1; TRINITY\_DN0\_c1\_g1\_i23.p1; TRINITY\_DN0\_c1\_g1\_i11.p1; TRINITY\_DN45\_c27\_g1\_i1.p1; TRINITY\_DN2658\_c0\_g2\_i1.p1\_ORF2; TRINITY\_DN0\_c1\_g1\_i10.p1; TRINITY\_DN2658\_c0\_g2\_i1.p1\_ORF1; TRINITY\_DN2658\_c0\_g2\_i1.p1 |  | Image |  | Peak List |  |
|  | | | | | | | | | | | | | | | | | | | | | | | | | | | | | | | | | | | | | | | | | | | | | | | | | | | | | |  |
|  | High |  | [R].GVLGTVKNLLIGA.[GS] |  | HCD |  |  |  | 2.86 |  | 1.3e-3 |  | 6.6e-2 |  | 0.275 |  |  |  |  |  |  |  |  |  |  |  |  |  |  |  | 0.33 |  | 1 |  | Sequest HT (3) |  | 2 |  | 627.89160 |  | -1.65 |  | 36.4079 |  | 9308 |  | 9308 |  | TRINITY\_DN0\_c1\_g1\_i15.p1; TRINITY\_DN0\_c1\_g1\_i23.p1; TRINITY\_DN0\_c1\_g1\_i11.p1; TRINITY\_DN2658\_c0\_g2\_i1.p1\_ORF2; TRINITY\_DN0\_c1\_g1\_i10.p1; TRINITY\_DN2658\_c0\_g2\_i1.p1\_ORF1; TRINITY\_DN2658\_c0\_g2\_i1.p1 |  | Image |  | Peak List |  |
|  | | | | | | | | | | | | | | | | | | | | | | | | | | | | | | | | | | | | | | | | | | | | | | | | | | | | | |  |
|  | High |  | [R].GVLGTVKNLLIGASKSAAQSVLK.[T] |  | HCD |  |  |  | 3.73 |  | 1.3e-3 |  | 4.8e-2 |  | 0.346 |  |  |  |  |  |  |  |  |  |  |  |  |  |  |  | 0.40 |  | 1 |  | Sequest HT (3) |  | 3 |  | 752.12042 |  | -1.14 |  | 37.3083 |  | 9744 |  | 9744 |  | TRINITY\_DN2658\_c0\_g2\_i1.p1\_ORF2; TRINITY\_DN2658\_c0\_g2\_i1.p1\_ORF1; TRINITY\_DN2658\_c0\_g2\_i1.p1 |  | Image |  | Peak List |  |
|  | | | | | | | | | | | | | | | | | | | | | | | | | | | | | | | | | | | | | | | | | | | | | | | | | | | | | |  |
|  | High |  | [N].LLIGASKSAAQSVLKTLScKLSNDc.[-] |  | HCD |  | C19(Dehydro); C25(Dehydro) |  | 1.92 |  | 1.3e-3 |  | 6.2e-2 |  | 0.291 |  | 161.15 |  | 1.000 |  |  |  | C19(Dehydro): 100; C25(Dehydro): 100 |  |  |  |  |  |  |  | 0.60 |  | 1 |  | Sequest HT (3) |  | 2 |  | 1274.67578 |  | -1.19 |  | 38.0897 |  | 10125 |  | 10125 |  | TRINITY\_DN2658\_c0\_g2\_i1.p1\_ORF2; TRINITY\_DN2658\_c0\_g2\_i1.p1\_ORF1; TRINITY\_DN2658\_c0\_g2\_i1.p1 |  | Image |  | Peak List |  |
|  | | | | | | | | | | | | | | | | | | | | | | | | | | | | | | | | | | | | | | | | | | | | | | | | | | | | | |  |
|  | High |  | [G].TVKnLLIGASKSAAQSVLKTLScKLSNDc.[-] |  | HCD |  | N4(Deamidated); C23(Dehydro); C29(Dehydro) |  | 3.93 |  | 1.3e-3 |  | 2.3e-2 |  | 0.506 |  | 179.88 |  | 1.000 |  | b1+(4): 444.25; b1+(5): 557.33; b1+(6): 670.41; b1+(7): 783.50; y1+(14): 1508.74; y1+(13): 1421.71; y1+(12): 1322.64; y1+(11): 1209.56; y1+(10): 1081.47; y1+(9): 980.42; y1+(8): 867.33; y1+(7): 780.30; y2+(18): 933.46 |  | N4(Deamidated): 100; C23(Dehydro): 100; C29(Dehydro): 100 |  |  |  |  |  |  |  | 0.53 |  | 1 |  | Sequest HT (3) |  | 4 |  | 748.65210 |  | 0.42 |  | 41.3461 |  | 11697 |  | 11697 |  | TRINITY\_DN2658\_c0\_g2\_i1.p1\_ORF2; TRINITY\_DN2658\_c0\_g2\_i1.p1\_ORF1; TRINITY\_DN2658\_c0\_g2\_i1.p1 |  | Image |  | Peak List |  |
|  | | | | | | | | | | | | | | | | | | | | | | | | | | | | | | | | | | | | | | | | | | | | | | | | | | | | | |  |
|  | High |  | [R].GVLGTVKNLLIGASKSAAQSVLKTLScKLSNDc.[-] |  | HCD |  | C27(Dehydro); C33(Dehydro) |  | 3.05 |  | 3.1e-3 |  | 1.1e-1 |  | 0.168 |  | 190.40 |  | 1.000 |  |  |  | C27(Dehydro): 100; C33(Dehydro): 100 |  |  |  |  |  |  |  | 0.65 |  | 1 |  | Sequest HT (3) |  | 3 |  | 1106.26965 |  | -0.69 |  | 44.1711 |  | 12887 |  | 12887 |  | TRINITY\_DN2658\_c0\_g2\_i1.p1\_ORF2; TRINITY\_DN2658\_c0\_g2\_i1.p1\_ORF1; TRINITY\_DN2658\_c0\_g2\_i1.p1 |  | Image |  | Peak List |  |
|  | | | | | | | | | | | | | | | | | | | | | | | | | | | | | | | | | | | | | | | | | | | | | | | | | | | | | |  |
|  | High |  | [R].GVLGTVKNLLIGASKSAAQSVLKTLScKLSNDc.[-] |  | HCD |  | C27(Dehydro); C33(Dehydro) |  | 3.48 |  | 1.3e-3 |  | 6.6e-2 |  | 0.275 |  | 238.28 |  | 1.000 |  |  |  | C27(Dehydro): 100; C33(Dehydro): 100 |  |  |  |  |  |  |  | 0.64 |  | 1 |  | Sequest HT (3) |  | 3 |  | 1106.26990 |  | -0.47 |  | 44.4728 |  | 13017 |  | 13017 |  | TRINITY\_DN2658\_c0\_g2\_i1.p1\_ORF2; TRINITY\_DN2658\_c0\_g2\_i1.p1\_ORF1; TRINITY\_DN2658\_c0\_g2\_i1.p1 |  | Image |  | Peak List |  |
|  | | | | | | | | | | | | | | | | | | | | | | | | | | | | | | | | | | | | | | | | | | | | | | | | | | | | | |  |
|  | High |  | [R].GVLGTVKNLLIGASKSAAQSVLKTLScKLSNDc.[-] |  | HCD |  | C27(Dehydro); C33(Dehydro) |  | 4.23 |  | 1.3e-3 |  | 6.0e-2 |  | 0.298 |  | 211.17 |  | 1.000 |  |  |  | C27(Dehydro): 100; C33(Dehydro): 100 |  |  |  |  |  |  |  | 0.54 |  | 1 |  | Sequest HT (3) |  | 4 |  | 829.95483 |  | 0.24 |  | 44.5733 |  | 13060 |  | 13060 |  | TRINITY\_DN2658\_c0\_g2\_i1.p1\_ORF2; TRINITY\_DN2658\_c0\_g2\_i1.p1\_ORF1; TRINITY\_DN2658\_c0\_g2\_i1.p1 |  | Image |  | Peak List |  |
|  | | | | | | | | | | | | | | | | | | | | | | | | | | | | | | | | | | | | | | | | | | | | | | | | | | | | | |  |
|  | High |  | [R].GVLGTVKNLLIGASKSAAQSVLKTLScKLSNDc.[-] |  | HCD |  | C27(Dehydro); C33(Dehydro) |  | 2.82 |  | 3.1e-3 |  | 8.9e-2 |  | 0.208 |  | 205.65 |  | 1.000 |  |  |  | C27(Dehydro): 100; C33(Dehydro): 100 |  |  |  |  |  |  |  | 0.60 |  | 1 |  | Sequest HT (3) |  | 3 |  | 1106.26953 |  | -0.80 |  | 44.6146 |  | 13079 |  | 13079 |  | TRINITY\_DN2658\_c0\_g2\_i1.p1\_ORF2; TRINITY\_DN2658\_c0\_g2\_i1.p1\_ORF1; TRINITY\_DN2658\_c0\_g2\_i1.p1 |  | Image |  | Peak List |  |
|  | | | | | | | | | | | | | | | | | | | | | | | | | | | | | | | | | | | | | | | | | | | | | | | | | | | | | |  |
|  | High |  | [R].GVLGTVKNLLIGASKSAAQSVLKTLScKLSNDc.[-] |  | HCD |  | C27(Dehydro); C33(Dehydro) |  | 3.49 |  | 1.3e-3 |  | 6.0e-2 |  | 0.298 |  | 158.97 |  | 1.000 |  |  |  | C27(Dehydro): 100; C33(Dehydro): 100 |  |  |  |  |  |  |  | 0.54 |  | 1 |  | Sequest HT (3) |  | 4 |  | 829.95581 |  | 1.42 |  | 44.7125 |  | 13119 |  | 13119 |  | TRINITY\_DN2658\_c0\_g2\_i1.p1\_ORF2; TRINITY\_DN2658\_c0\_g2\_i1.p1\_ORF1; TRINITY\_DN2658\_c0\_g2\_i1.p1 |  | Image |  | Peak List |  |
|  | | | | | | | | | | | | | | | | | | | | | | | | | | | | | | | | | | | | | | | | | | | | | | | | | | | | | |  |
|  | High |  | [R].GVLGTVKnLLIGASKSAAQSVLKTLScKLSNDc.[-] |  | HCD |  | N8(Deamidated); C27(Dehydro); C33(Dehydro) |  | 3.17 |  | 1.3e-3 |  | 1.9e-2 |  | 0.548 |  | 171.10 |  | 1.000 |  | b1+(8): 770.44; b1+(9): 883.52; b1+(10): 996.61; y1+(14): 1508.74; y1+(12): 1322.64; y1+(11): 1209.56; y1+(8): 867.33; y1+(7): 780.30; b2+(16): 770.46 |  | N8(Deamidated): 100; C27(Dehydro): 100; C33(Dehydro): 100 |  |  |  |  |  |  |  | 0.23 |  | 1 |  | Sequest HT (3) |  | 3 |  | 1106.59973 |  | 1.18 |  | 46.3309 |  | 13823 |  | 13823 |  | TRINITY\_DN2658\_c0\_g2\_i1.p1\_ORF2; TRINITY\_DN2658\_c0\_g2\_i1.p1\_ORF1; TRINITY\_DN2658\_c0\_g2\_i1.p1 |  | Image |  | Peak List |  |
|  | | | | | | | | | | | | | | | | | | | | | | | | | | | | | | | | | | | | | | | | | | | | | | | | | | | | | |  |
|  | High |  | [R].GVLGTVKnLLIGASKSAAQSVLKTLScKLSNDc.[-] |  | HCD |  | N8(Deamidated); C27(Dehydro); C33(Dehydro) |  | 4.20 |  | 2.4e-3 |  | 8.1e-2 |  | 0.23 |  | 204.26 |  | 1.000 |  | b1+(8): 770.44; b1+(9): 883.52; b1+(10): 996.61; b1+(11): 1109.69; b1+(12): 1166.71; b1+(13): 1237.75; y1+(12): 1322.64; y1+(11): 1209.56; y1+(10): 1081.47; y1+(9): 980.42; y1+(8): 867.33; b2+(9): 442.27; b2+(16): 770.46 |  | N8(Deamidated): 100; C27(Dehydro): 100; C33(Dehydro): 100 |  |  |  |  |  |  |  | 0.47 |  | 1 |  | Sequest HT (3) |  | 4 |  | 830.20203 |  | 1.67 |  | 46.3757 |  | 13846 |  | 13846 |  | TRINITY\_DN2658\_c0\_g2\_i1.p1\_ORF2; TRINITY\_DN2658\_c0\_g2\_i1.p1\_ORF1; TRINITY\_DN2658\_c0\_g2\_i1.p1 |  | Image |  | Peak List |  |
|  | | | | | | | | | | | | | | | | | | | | | | | | | | | | | | | | | | | | | | | | | | | | | | | | | | | | | |  |
|  | High |  | [R].GVLGTVKnLLIGASKSAAQSVLKTLScKLSNDc.[-] |  | HCD |  | N8(Deamidated); C27(Dehydro); C33(Dehydro) |  | 3.79 |  | 1.3e-3 |  | 5.8e-2 |  | 0.304 |  | 226.16 |  | 1.000 |  | b1+(8): 770.44; b1+(9): 883.52; b1+(10): 996.61; b1+(13): 1237.75; y1+(18): 1865.91; y1+(17): 1778.88; y1+(16): 1707.84; y1+(14): 1508.74; y1+(12): 1322.64; y1+(11): 1209.56; y1+(8): 867.33; y1+(7): 780.30; b2+(18): 841.50 |  | N8(Deamidated): 100; C27(Dehydro): 100; C33(Dehydro): 100 |  |  |  |  |  |  |  | 0.53 |  | 1 |  | Sequest HT (3) |  | 3 |  | 1106.59814 |  | -0.25 |  | 46.4692 |  | 13883 |  | 13883 |  | TRINITY\_DN2658\_c0\_g2\_i1.p1\_ORF2; TRINITY\_DN2658\_c0\_g2\_i1.p1\_ORF1; TRINITY\_DN2658\_c0\_g2\_i1.p1 |  | Image |  | Peak List |  |
|  | | | | | | | | | | | | | | | | | | | | | | | | | | | | | | | | | | | | | | | | | | | | | | | | | | | | | |  |
|  | High |  | [R].GVLGTVKnLLIGASKSAAQSVLKTLScKLSNDc.[-] |  | HCD |  | N8(Deamidated); C27(Dehydro); C33(Dehydro) |  | 4.22 |  | 1.3e-3 |  | 1.3e-2 |  | 0.633 |  | 211.79 |  | 1.000 |  | b1+(8): 770.44; b1+(9): 883.52; b1+(10): 996.61; b1+(12): 1166.71; b1+(13): 1237.75; y1+(18): 1865.91; y1+(16): 1707.84; y1+(14): 1508.74; y1+(12): 1322.64; y1+(11): 1209.56; y1+(8): 867.33; y1+(7): 780.30 |  | N8(Deamidated): 100; C27(Dehydro): 100; C33(Dehydro): 100 |  |  |  |  |  |  |  | 0.67 |  | 1 |  | Sequest HT (3) |  | 3 |  | 1106.59875 |  | 0.30 |  | 46.6098 |  | 13941 |  | 13941 |  | TRINITY\_DN2658\_c0\_g2\_i1.p1\_ORF2; TRINITY\_DN2658\_c0\_g2\_i1.p1\_ORF1; TRINITY\_DN2658\_c0\_g2\_i1.p1 |  | Image |  | Peak List |  |
|  | | | | | | | | | | | | | | | | | | | | | | | | | | | | | | | | | | | | | | | | | | | | | | | | | | | | | |  |
|  | High |  | [R].GVLGTVKnLLIGASKSAAQSVLKTLScKLSNDc.[-] |  | HCD |  | N8(Deamidated); C27(Dehydro); C33(Dehydro) |  | 3.95 |  | 3.1e-3 |  | 1.3e-1 |  | 0.123 |  | 200.47 |  | 1.000 |  | b1+(8): 770.44; b1+(9): 883.52; b1+(10): 996.61; b1+(11): 1109.69; b1+(12): 1166.71; b1+(13): 1237.75; y1+(12): 1322.64; y1+(11): 1209.56; y1+(10): 1081.47; y1+(9): 980.42; y1+(8): 867.33; y1+(7): 780.30; b2+(16): 770.46; y2+(12): 661.83 |  | N8(Deamidated): 100; C27(Dehydro): 100; C33(Dehydro): 100 |  |  |  |  |  |  |  | 0.66 |  | 1 |  | Sequest HT (3) |  | 4 |  | 830.20294 |  | 2.78 |  | 46.6598 |  | 13962 |  | 13962 |  | TRINITY\_DN2658\_c0\_g2\_i1.p1\_ORF2; TRINITY\_DN2658\_c0\_g2\_i1.p1\_ORF1; TRINITY\_DN2658\_c0\_g2\_i1.p1 |  | Image |  | Peak List |  |
|  | | | | | | | | | | | | | | | | | | | | | | | | | | | | | | | | | | | | | | | | | | | | | | | | | | | | | |  |
|  | High |  | [R].GVLGTVKnLLIGASKSAAQSVLKTLScKLSNDc.[-] |  | HCD |  | N8(Deamidated); C27(Dehydro); C33(Dehydro) |  | 3.49 |  | 3.1e-3 |  | 1.4e-1 |  | 0.103 |  | 230.02 |  | 1.000 |  | b1+(8): 770.44; b1+(9): 883.52; b1+(10): 996.61; b1+(12): 1166.71; b1+(13): 1237.75; y1+(18): 1865.91; y1+(17): 1778.88; y1+(16): 1707.84; y1+(14): 1508.74; y1+(13): 1421.71; y1+(11): 1209.56; y1+(8): 867.33; y1+(7): 780.30; b2+(16): 770.46 |  | N8(Deamidated): 100; C27(Dehydro): 100; C33(Dehydro): 100 |  |  |  |  |  |  |  | 0.53 |  | 1 |  | Sequest HT (3) |  | 3 |  | 1106.59839 |  | -0.03 |  | 46.7522 |  | 14001 |  | 14001 |  | TRINITY\_DN2658\_c0\_g2\_i1.p1\_ORF2; TRINITY\_DN2658\_c0\_g2\_i1.p1\_ORF1; TRINITY\_DN2658\_c0\_g2\_i1.p1 |  | Image |  | Peak List |  |
|  | | | | | | | | | | | | | | | | | | | | | | | | | | | | | | | | | | | | | | | | | | | | | | | | | | | | | |  |
|  | High |  | [R].GVLGTVKnLLIGASKSAAQSVLKTLScKLSNDc.[-] |  | HCD |  | N8(Deamidated); C27(Dehydro); C33(Dehydro) |  | 3.38 |  | 3.1e-3 |  | 1.2e-1 |  | 0.15 |  | 210.82 |  | 1.000 |  | b1+(8): 770.44; b1+(9): 883.52; b1+(10): 996.61; b1+(11): 1109.69; b1+(12): 1166.71; b1+(13): 1237.75; y1+(12): 1322.64; y1+(11): 1209.56; y1+(10): 1081.47; y1+(8): 867.33; b2+(10): 498.81; b2+(11): 555.35; b2+(16): 770.46; b2+(21): 998.58 |  | N8(Deamidated): 100; C27(Dehydro): 100; C33(Dehydro): 100 |  |  |  |  |  |  |  | 1.00 |  | 1 |  | Sequest HT (3) |  | 4 |  | 830.20074 |  | 0.13 |  | 46.7982 |  | 14020 |  | 14020 |  | TRINITY\_DN2658\_c0\_g2\_i1.p1\_ORF2; TRINITY\_DN2658\_c0\_g2\_i1.p1\_ORF1; TRINITY\_DN2658\_c0\_g2\_i1.p1 |  | Image |  | Peak List |  |
|  | | | | | | | | | | | | | | | | | | | | | | | | | | | | | | | | | | | | | | | | | | | | | | | | | | | | | |  |
|  | High |  | [R].GVLGTVKnLLIGASKSAAQSVLKTLScKLSNDc.[-] |  | HCD |  | N8(Deamidated); C27(Dehydro); C33(Dehydro) |  | 3.73 |  | 1.3e-3 |  | 4.0e-2 |  | 0.388 |  | 268.39 |  | 1.000 |  | b1+(8): 770.44; b1+(9): 883.52; b1+(10): 996.61; b1+(11): 1109.69; b1+(13): 1237.75; y1+(18): 1865.91; y1+(16): 1707.84; y1+(14): 1508.74; y1+(13): 1421.71; y1+(12): 1322.64; y1+(11): 1209.56; y1+(9): 980.42; y1+(8): 867.33; y1+(7): 780.30; b2+(16): 770.46; b2+(18): 841.50 |  | N8(Deamidated): 100; C27(Dehydro): 100; C33(Dehydro): 100 |  |  |  |  |  |  |  | 0.62 |  | 1 |  | Sequest HT (3) |  | 3 |  | 1106.59741 |  | -0.92 |  | 46.8890 |  | 14058 |  | 14058 |  | TRINITY\_DN2658\_c0\_g2\_i1.p1\_ORF2; TRINITY\_DN2658\_c0\_g2\_i1.p1\_ORF1; TRINITY\_DN2658\_c0\_g2\_i1.p1 |  | Image |  | Peak List |  |
|  | | | | | | | | | | | | | | | | | | | | | | | | | | | | | | | | | | | | | | | | | | | | | | | | | | | | | |  |
|  | High |  | [R].GVLGTVKnLLIGASKSAAQSVLKTLScKLSNDc.[-] |  | HCD |  | N8(Deamidated); C27(Dehydro); C33(Dehydro) |  | 3.72 |  | 3.1e-3 |  | 1.2e-1 |  | 0.132 |  | 250.01 |  | 1.000 |  | b1+(8): 770.44; b1+(9): 883.52; b1+(10): 996.61; b1+(11): 1109.69; b1+(12): 1166.71; b1+(13): 1237.75; y1+(18): 1865.91; y1+(14): 1508.74; y1+(13): 1421.71; y1+(12): 1322.64; y1+(11): 1209.56; y1+(9): 980.42; y1+(8): 867.33; y1+(7): 780.30; b2+(18): 841.50 |  | N8(Deamidated): 100; C27(Dehydro): 100; C33(Dehydro): 100 |  |  |  |  |  |  |  | 0.58 |  | 1 |  | Sequest HT (3) |  | 3 |  | 1106.59705 |  | -1.25 |  | 47.0297 |  | 14121 |  | 14121 |  | TRINITY\_DN2658\_c0\_g2\_i1.p1\_ORF2; TRINITY\_DN2658\_c0\_g2\_i1.p1\_ORF1; TRINITY\_DN2658\_c0\_g2\_i1.p1 |  | Image |  | Peak List |  |
|  | | | | | | | | | | | | | | | | | | | | | | | | | | | | | | | | | | | | | | | | | | | | | | | | | | | | | |  |
|  | High |  | [R].GVLGTVKnLLIGASKSAAQSVLKTLScKLSNDc.[-] |  | HCD |  | N8(Deamidated); C27(Dehydro); C33(Dehydro) |  | 3.45 |  | 3.1e-3 |  | 1.1e-1 |  | 0.15 |  | 194.05 |  | 1.000 |  | b1+(8): 770.44; b1+(9): 883.52; b1+(10): 996.61; b1+(11): 1109.69; b1+(13): 1237.75; y1+(14): 1508.74; y1+(12): 1322.64; y1+(11): 1209.56; y1+(9): 980.42; y1+(8): 867.33; y1+(7): 780.30; b2+(18): 841.50 |  | N8(Deamidated): 100; C27(Dehydro): 100; C33(Dehydro): 100 |  |  |  |  |  |  |  | 0.54 |  | 1 |  | Sequest HT (3) |  | 3 |  | 1106.59705 |  | -1.25 |  | 47.1701 |  | 14189 |  | 14189 |  | TRINITY\_DN2658\_c0\_g2\_i1.p1\_ORF2; TRINITY\_DN2658\_c0\_g2\_i1.p1\_ORF1; TRINITY\_DN2658\_c0\_g2\_i1.p1 |  | Image |  | Peak List |  |
|  | | | | | | | | | | | | | | | | | | | | | | | | | | | | | | | | | | | | | | | | | | | | | | | | | | | | | |  |
|  | High |  | [R].GVLGTVKnLLIGASKSAAQSVLKTLScKLSNDc.[-] |  | HCD |  | N8(Deamidated); C27(Dehydro); C33(Dehydro) |  | 4.04 |  | 1.3e-3 |  | 3.0e-2 |  | 0.454 |  | 250.01 |  | 1.000 |  | b1+(8): 770.44; b1+(9): 883.52; b1+(10): 996.61; b1+(11): 1109.69; b1+(12): 1166.71; b1+(13): 1237.75; y1+(14): 1508.74; y1+(13): 1421.71; y1+(12): 1322.64; y1+(11): 1209.56; y1+(9): 980.42; y1+(8): 867.33; y1+(7): 780.30; b2+(16): 770.46; b2+(21): 998.58 |  | N8(Deamidated): 100; C27(Dehydro): 100; C33(Dehydro): 100 |  |  |  |  |  |  |  | 0.57 |  | 1 |  | Sequest HT (3) |  | 3 |  | 1106.59827 |  | -0.14 |  | 47.3123 |  | 14249 |  | 14249 |  | TRINITY\_DN2658\_c0\_g2\_i1.p1\_ORF2; TRINITY\_DN2658\_c0\_g2\_i1.p1\_ORF1; TRINITY\_DN2658\_c0\_g2\_i1.p1 |  | Image |  | Peak List |  |
|  | | | | | | | | | | | | | | | | | | | | | | | | | | | | | | | | | | | | | | | | | | | | | | | | | | | | | |  |
|  | High |  | [R].GVLGTVKnLLIGASKSAAQSVLKTLScKLSnDc.[-] |  | HCD |  | N8(Deamidated); C27(Dehydro); N31(Deamidated); C33(Dehydro) |  | 2.84 |  | 1.3e-3 |  | 3.4e-2 |  | 0.422 |  | 224.01 |  | 1.000 |  |  |  | N8(Deamidated): 100; C27(Dehydro): 100; N31(Deamidated): 100; C33(Dehydro): 100 |  |  |  |  |  |  |  | 0.50 |  | 1 |  | Sequest HT (3) |  | 3 |  | 1106.92578 |  | -0.59 |  | 50.3140 |  | 15473 |  | 15473 |  | TRINITY\_DN2658\_c0\_g2\_i1.p1\_ORF2; TRINITY\_DN2658\_c0\_g2\_i1.p1\_ORF1; TRINITY\_DN2658\_c0\_g2\_i1.p1 |  | Image |  | Peak List |  |
|  | | | | | | | | | | | | | | | | | | | | | | | | | | | | | | | | | | | | | | | | | | | | | | | | | | | | | |  |
|  | High |  | [R].GVLGTVKnLLIGASKSAAQSVLKTLScKLSNDc.[-] |  | HCD |  | N8(Deamidated); C27(Dehydro); C33(Dehydro) |  | 4.06 |  | 1.3e-3 |  | 1.1e-2 |  | 0.67 |  | 258.04 |  | 1.000 |  | b1+(8): 770.44; b1+(9): 883.52; b1+(10): 996.61; b1+(11): 1109.69; b1+(12): 1166.71; b1+(13): 1237.75; y1+(14): 1508.74; y1+(12): 1322.64; y1+(11): 1209.56; y1+(9): 980.42; y1+(8): 867.33; y1+(7): 780.30; y2+(23): 1161.59 |  | N8(Deamidated): 100; C27(Dehydro): 100; C33(Dehydro): 100 |  |  |  |  |  |  |  | 0.54 |  | 1 |  | Sequest HT (3) |  | 3 |  | 1106.59888 |  | 0.41 |  | 51.6723 |  | 16009 |  | 16009 |  | TRINITY\_DN2658\_c0\_g2\_i1.p1\_ORF2; TRINITY\_DN2658\_c0\_g2\_i1.p1\_ORF1; TRINITY\_DN2658\_c0\_g2\_i1.p1 |  | Image |  | Peak List |  |
|  | | | | | | | | | | | | | | | | | | | | | | | | | | | | | | | | | | | | | | | | | | | | | | | | | | | | | |  |
|  | High |  | [R].GVLGTVKnLLIGASKSAAQSVLKTLScKLSNDc.[-] |  | HCD |  | N8(Deamidated); C27(Dehydro); C33(Dehydro) |  | 3.31 |  | 3.1e-3 |  | 1.4e-1 |  | 0.101 |  | 137.21 |  | 0.028 |  | y1+(14): 1509.73; y1+(12): 1323.63; y1+(11): 1210.54; y1+(10): 1082.45; b2+(12): 583.37; y2+(23): 1162.09; y2+(22): 1105.54; y2+(20): 1041.51; y2+(11): 605.78 |  | C27(Dehydro): 100; N31(Deamidated): 97.23; C33(Dehydro): 100 |  |  |  |  |  |  |  | 0.01 |  | 1 |  | Sequest HT (3) |  | 4 |  | 830.20093 |  | 0.35 |  | 52.8870 |  | 16482 |  | 16482 |  | TRINITY\_DN2658\_c0\_g2\_i1.p1\_ORF2; TRINITY\_DN2658\_c0\_g2\_i1.p1\_ORF1; TRINITY\_DN2658\_c0\_g2\_i1.p1 |  | Image |  | Peak List |  |
|  | | | | | | | | | | | | | | | | | | | | | | | | | | | | | | | | | | | | | | | | | | | | | | | | | | | | | |  |
|  | High |  | [R].GVLGTVKNLLIGASKSAAQSVLKTLScKLSnDc.[-] |  | HCD |  | C27(Dehydro); N31(Deamidated); C33(Dehydro) |  | 3.28 |  | 1.3e-3 |  | 4.0e-2 |  | 0.385 |  | 152.65 |  | 0.972 |  | y1+(14): 1509.73; y1+(12): 1323.63; y1+(11): 1210.54; y1+(10): 1082.45; b2+(12): 583.37; y2+(23): 1162.09; y2+(22): 1105.54; y2+(20): 1041.51; y2+(11): 605.78 |  | C27(Dehydro): 100; N31(Deamidated): 97.23; C33(Dehydro): 100 |  |  |  |  |  |  |  |  |  | 2 |  | Sequest HT (3) |  | 4 |  | 830.20093 |  | 0.35 |  | 52.8870 |  | 16482 |  | 16482 |  | TRINITY\_DN2658\_c0\_g2\_i1.p1\_ORF2; TRINITY\_DN2658\_c0\_g2\_i1.p1\_ORF1; TRINITY\_DN2658\_c0\_g2\_i1.p1 |  | Image |  | Peak List |  |
|  | | | | | | | | | | | | | | | | | | | | | | | | | | | | | | | | | | | | | | | | | | | | | | | | | | | | | |  |
|  | High |  | [R].GVLGTVKnLLIGASKSAAQSVLKTLScKLSNDc.[-] |  | HCD |  | N8(Deamidated); C27(Dehydro); C33(Dehydro) |  | 3.57 |  | 3.1e-3 |  | 8.7e-2 |  | 0.215 |  | 154.24 |  | 1.000 |  | b1+(8): 770.44; b1+(9): 883.52; b1+(10): 996.61; b1+(11): 1109.69; b1+(13): 1237.75; y1+(11): 1209.56; y1+(10): 1081.47; y1+(8): 867.33; b2+(9): 442.27; b2+(10): 498.81 |  | N8(Deamidated): 100; C27(Dehydro): 100; C33(Dehydro): 100 |  |  |  |  |  |  |  | 0.52 |  | 1 |  | Sequest HT (3) |  | 4 |  | 830.20154 |  | 1.08 |  | 62.2878 |  | 19565 |  | 19565 |  | TRINITY\_DN2658\_c0\_g2\_i1.p1\_ORF2; TRINITY\_DN2658\_c0\_g2\_i1.p1\_ORF1; TRINITY\_DN2658\_c0\_g2\_i1.p1 |  | Image |  | Peak List |  |
|  | | | | | | | | | | | | | | | | | | | | | | | | | | | | | | | | | | | | | | | | | | | | | | | | | | | | | |  |
|  | **TRINITY\_DN0\_c1\_g1\_i17.p1 - TRINITY\_DN0\_c1\_g1\_i17.p1** | | | | | | | | | | | | | | | | | | | | | | | | | | | | | | | | | | | | | | | | | | | | | | | | | | | |  |  |
|  | | | | | | | | | | | | | | | | | | | | | | | | | | | | | | | | | | | | | | | | | | | | | | | | | | | | | |  |
|  | High |  | [R].GLLSGILGAGK.[HN] |  | HCD |  |  |  | 2.70 |  | 1.3e-3 |  | 7.8e-3 |  | 0.744 |  |  |  |  |  |  |  |  |  |  |  |  |  |  |  | 0.38 |  | 1 |  | Sequest HT (3) |  | 2 |  | 493.30603 |  | 0.74 |  | 32.6730 |  | 7618 |  | 7618 |  | TRINITY\_DN4414\_c6\_g1\_i1.p1\_ORF2; TRINITY\_DN4414\_c6\_g1\_i1.p1; TRINITY\_DN4414\_c6\_g1\_i1.p1\_ORF1; TRINITY\_DN0\_c1\_g1\_i17.p1\_ORF1; TRINITY\_DN77\_c0\_g1\_i1.p1\_ORF2; TRINITY\_DN39\_c0\_g1\_i2.p3; TRINITY\_DN0\_c1\_g1\_i2.p1; TRINITY\_DN0\_c1\_g1\_i3.p1; TRINITY\_DN1399\_c0\_g1\_i1.p1\_ORF1; TRINITY\_DN0\_c1\_g1\_i17.p1; TRINITY\_DN77\_c0\_g1\_i1.p1\_ORF1 |  | Image |  | Peak List |  |
|  | | | | | | | | | | | | | | | | | | | | | | | | | | | | | | | | | | | | | | | | | | | | | | | | | | | | | |  |
|  | High |  | [R].GLLSGILGAGK.[HN] |  | HCD |  |  |  | 1.53 |  | 1.3e-3 |  | 3.0e-2 |  | 0.45 |  |  |  |  |  |  |  |  |  |  |  |  |  |  |  | 0.36 |  | 1 |  | Sequest HT (3) |  | 2 |  | 493.30582 |  | 0.30 |  | 32.8253 |  | 7684 |  | 7684 |  | TRINITY\_DN4414\_c6\_g1\_i1.p1\_ORF2; TRINITY\_DN4414\_c6\_g1\_i1.p1; TRINITY\_DN4414\_c6\_g1\_i1.p1\_ORF1; TRINITY\_DN0\_c1\_g1\_i17.p1\_ORF1; TRINITY\_DN77\_c0\_g1\_i1.p1\_ORF2; TRINITY\_DN39\_c0\_g1\_i2.p3; TRINITY\_DN0\_c1\_g1\_i2.p1; TRINITY\_DN0\_c1\_g1\_i3.p1; TRINITY\_DN1399\_c0\_g1\_i1.p1\_ORF1; TRINITY\_DN0\_c1\_g1\_i17.p1; TRINITY\_DN77\_c0\_g1\_i1.p1\_ORF1 |  | Image |  | Peak List |  |
|  | | | | | | | | | | | | | | | | | | | | | | | | | | | | | | | | | | | | | | | | | | | | | | | | | | | | | |  |
|  | High |  | [R].GLLSGILGAGKNIVcGLSGLc.[-] |  | HCD |  | C15(Dehydro); C21(Dehydro) |  | 3.85 |  | 1.3e-3 |  | 8.9e-3 |  | 0.716 |  | 344.08 |  | 1.000 |  |  |  | C15(Dehydro): 100; C21(Dehydro): 100 |  |  |  |  |  |  |  | 0.57 |  | 1 |  | Sequest HT (3) |  | 2 |  | 972.02844 |  | 2.24 |  | 53.8416 |  | 16852 |  | 16852 |  | TRINITY\_DN0\_c1\_g1\_i17.p1\_ORF1; TRINITY\_DN0\_c1\_g1\_i2.p1; TRINITY\_DN0\_c1\_g1\_i3.p1; TRINITY\_DN0\_c1\_g1\_i17.p1 |  | Image |  | Peak List |  |
|  | | | | | | | | | | | | | | | | | | | | | | | | | | | | | | | | | | | | | | | | | | | | | | | | | | | | | |  |
|  | **TRINITY\_DN77\_c0\_g1\_i1.p1\_ORF1 - TRINITY\_DN77\_c0\_g1\_i1.p1\_ORF1** | | | | | | | | | | | | | | | | | | | | | | | | | | | | | | | | | | | | | | | | | | | | | | | | | | | |  |  |
|  | | | | | | | | | | | | | | | | | | | | | | | | | | | | | | | | | | | | | | | | | | | | | | | | | | | | | |  |
|  | High |  | [G].ILGAGKHIVcGLSGLc.[-] |  | HCD |  | C10(Dehydro); C16(Dehydro) |  | 2.21 |  | 2.4e-3 |  | 8.5e-2 |  | 0.218 |  | 213.96 |  | 1.000 |  |  |  | C10(Dehydro): 100; C16(Dehydro): 100 |  |  |  |  |  |  |  | 0.29 |  | 1 |  | Sequest HT (3) |  | 2 |  | 769.91284 |  | 0.16 |  | 31.6763 |  | 7197 |  | 7197 |  | TRINITY\_DN9643\_c0\_g1\_i4.p5\_ORF1; TRINITY\_DN77\_c0\_g1\_i1.p1\_ORF2; TRINITY\_DN1399\_c0\_g1\_i1.p1\_ORF1; TRINITY\_DN77\_c0\_g1\_i1.p1\_ORF1; TRINITY\_DN9643\_c0\_g1\_i4.p5 |  | Image |  | Peak List |  |
|  | | | | | | | | | | | | | | | | | | | | | | | | | | | | | | | | | | | | | | | | | | | | | | | | | | | | | |  |
|  | High |  | [R].GLLSGILGAGK.[HN] |  | HCD |  |  |  | 2.70 |  | 1.3e-3 |  | 7.8e-3 |  | 0.744 |  |  |  |  |  |  |  |  |  |  |  |  |  |  |  | 0.38 |  | 1 |  | Sequest HT (3) |  | 2 |  | 493.30603 |  | 0.74 |  | 32.6730 |  | 7618 |  | 7618 |  | TRINITY\_DN4414\_c6\_g1\_i1.p1\_ORF2; TRINITY\_DN4414\_c6\_g1\_i1.p1; TRINITY\_DN4414\_c6\_g1\_i1.p1\_ORF1; TRINITY\_DN0\_c1\_g1\_i17.p1\_ORF1; TRINITY\_DN77\_c0\_g1\_i1.p1\_ORF2; TRINITY\_DN39\_c0\_g1\_i2.p3; TRINITY\_DN0\_c1\_g1\_i2.p1; TRINITY\_DN0\_c1\_g1\_i3.p1; TRINITY\_DN1399\_c0\_g1\_i1.p1\_ORF1; TRINITY\_DN0\_c1\_g1\_i17.p1; TRINITY\_DN77\_c0\_g1\_i1.p1\_ORF1 |  | Image |  | Peak List |  |
|  | | | | | | | | | | | | | | | | | | | | | | | | | | | | | | | | | | | | | | | | | | | | | | | | | | | | | |  |
|  | High |  | [R].GLLSGILGAGK.[HN] |  | HCD |  |  |  | 1.53 |  | 1.3e-3 |  | 3.0e-2 |  | 0.45 |  |  |  |  |  |  |  |  |  |  |  |  |  |  |  | 0.36 |  | 1 |  | Sequest HT (3) |  | 2 |  | 493.30582 |  | 0.30 |  | 32.8253 |  | 7684 |  | 7684 |  | TRINITY\_DN4414\_c6\_g1\_i1.p1\_ORF2; TRINITY\_DN4414\_c6\_g1\_i1.p1; TRINITY\_DN4414\_c6\_g1\_i1.p1\_ORF1; TRINITY\_DN0\_c1\_g1\_i17.p1\_ORF1; TRINITY\_DN77\_c0\_g1\_i1.p1\_ORF2; TRINITY\_DN39\_c0\_g1\_i2.p3; TRINITY\_DN0\_c1\_g1\_i2.p1; TRINITY\_DN0\_c1\_g1\_i3.p1; TRINITY\_DN1399\_c0\_g1\_i1.p1\_ORF1; TRINITY\_DN0\_c1\_g1\_i17.p1; TRINITY\_DN77\_c0\_g1\_i1.p1\_ORF1 |  | Image |  | Peak List |  |
|  | | | | | | | | | | | | | | | | | | | | | | | | | | | | | | | | | | | | | | | | | | | | | | | | | | | | | |  |
|  | High |  | [L].SGILGAGKHIVcGLSGLc.[-] |  | HCD |  | C12(Dehydro); C18(Dehydro) |  | 2.69 |  | 1.3e-3 |  | 1.9e-2 |  | 0.552 |  | 274.58 |  | 1.000 |  |  |  | C12(Dehydro): 100; C18(Dehydro): 100 |  |  |  |  |  |  |  | 0.25 |  | 1 |  | Sequest HT (3) |  | 2 |  | 841.93842 |  | -1.25 |  | 37.3071 |  | 9743 |  | 9743 |  | TRINITY\_DN9643\_c0\_g1\_i4.p5\_ORF1; TRINITY\_DN77\_c0\_g1\_i1.p1\_ORF2; TRINITY\_DN1399\_c0\_g1\_i1.p1\_ORF1; TRINITY\_DN77\_c0\_g1\_i1.p1\_ORF1; TRINITY\_DN9643\_c0\_g1\_i4.p5 |  | Image |  | Peak List |  |
|  | | | | | | | | | | | | | | | | | | | | | | | | | | | | | | | | | | | | | | | | | | | | | | | | | | | | | |  |
|  | High |  | [L].SGILGAGKHIVcGLSGLc.[-] |  | HCD |  | C12(Dehydro); C18(Dehydro) |  | 2.37 |  | 6.5e-3 |  | 2.1e-1 |  | 0.006 |  | 192.32 |  | 1.000 |  |  |  | C12(Dehydro): 100; C18(Dehydro): 100 |  |  |  |  |  |  |  | 0.05 |  | 1 |  | Sequest HT (3) |  | 3 |  | 561.62872 |  | -0.02 |  | 37.3096 |  | 9745 |  | 9745 |  | TRINITY\_DN9643\_c0\_g1\_i4.p5\_ORF1; TRINITY\_DN77\_c0\_g1\_i1.p1\_ORF2; TRINITY\_DN1399\_c0\_g1\_i1.p1\_ORF1; TRINITY\_DN77\_c0\_g1\_i1.p1\_ORF1; TRINITY\_DN9643\_c0\_g1\_i4.p5 |  | Image |  | Peak List |  |
|  | | | | | | | | | | | | | | | | | | | | | | | | | | | | | | | | | | | | | | | | | | | | | | | | | | | | | |  |
|  | High |  | [R].GLLSGILGAGKHIVcGLSGLc.[-] |  | HCD |  | C15(Dehydro); C21(Dehydro) |  | 3.52 |  | 1.3e-3 |  | 1.5e-2 |  | 0.605 |  | 265.04 |  | 1.000 |  |  |  | C15(Dehydro): 100; C21(Dehydro): 100 |  |  |  |  |  |  |  | 0.11 |  | 1 |  | Sequest HT (3) |  | 2 |  | 983.53290 |  | -1.39 |  | 51.2710 |  | 15849 |  | 15849 |  | TRINITY\_DN77\_c0\_g1\_i1.p1\_ORF2; TRINITY\_DN1399\_c0\_g1\_i1.p1\_ORF1; TRINITY\_DN77\_c0\_g1\_i1.p1\_ORF1 |  | Image |  | Peak List |  |
|  | | | | | | | | | | | | | | | | | | | | | | | | | | | | | | | | | | | | | | | | | | | | | | | | | | | | | |  |
|  | High |  | [R].GLLSGILGAGKHIVcGLSGLc.[-] |  | HCD |  | C15(Dehydro); C21(Dehydro) |  | 3.82 |  | 1.3e-3 |  | 1.6e-3 |  | 1.09 |  | 397.83 |  | 1.000 |  |  |  | C15(Dehydro): 100; C21(Dehydro): 100 |  |  |  |  |  |  |  | 0.10 |  | 1 |  | Sequest HT (3) |  | 2 |  | 983.53333 |  | -0.95 |  | 51.4137 |  | 15906 |  | 15906 |  | TRINITY\_DN77\_c0\_g1\_i1.p1\_ORF2; TRINITY\_DN1399\_c0\_g1\_i1.p1\_ORF1; TRINITY\_DN77\_c0\_g1\_i1.p1\_ORF1 |  | Image |  | Peak List |  |
|  | | | | | | | | | | | | | | | | | | | | | | | | | | | | | | | | | | | | | | | | | | | | | | | | | | | | | |  |
|  | High |  | [R].GLLSGILGAGKHIVcGLSGLc.[-] |  | HCD |  | C15(Dehydro); C21(Dehydro) |  | 3.53 |  | 1.3e-3 |  | 9.3e-4 |  | 1.21 |  | 393.62 |  | 1.000 |  |  |  | C15(Dehydro): 100; C21(Dehydro): 100 |  |  |  |  |  |  |  | 0.13 |  | 1 |  | Sequest HT (3) |  | 2 |  | 983.53271 |  | -1.57 |  | 51.5559 |  | 15960 |  | 15960 |  | TRINITY\_DN77\_c0\_g1\_i1.p1\_ORF2; TRINITY\_DN1399\_c0\_g1\_i1.p1\_ORF1; TRINITY\_DN77\_c0\_g1\_i1.p1\_ORF1 |  | Image |  | Peak List |  |
|  | | | | | | | | | | | | | | | | | | | | | | | | | | | | | | | | | | | | | | | | | | | | | | | | | | | | | |  |
|  | High |  | [R].GLLSGILGAGKHIVcGLSGLc.[-] |  | HCD |  | C15(Dehydro); C21(Dehydro) |  | 3.27 |  | 1.3e-3 |  | 8.5e-3 |  | 0.727 |  | 273.54 |  | 1.000 |  |  |  | C15(Dehydro): 100; C21(Dehydro): 100 |  |  |  |  |  |  |  | 0.15 |  | 1 |  | Sequest HT (3) |  | 2 |  | 983.53320 |  | -1.08 |  | 51.6988 |  | 16019 |  | 16019 |  | TRINITY\_DN77\_c0\_g1\_i1.p1\_ORF2; TRINITY\_DN1399\_c0\_g1\_i1.p1\_ORF1; TRINITY\_DN77\_c0\_g1\_i1.p1\_ORF1 |  | Image |  | Peak List |  |
|  | | | | | | | | | | | | | | | | | | | | | | | | | | | | | | | | | | | | | | | | | | | | | | | | | | | | | |  |
|  | High |  | [R].GLLSGILGAGKHIVcGLSGLc.[-] |  | HCD |  | C15(Dehydro); C21(Dehydro) |  | 2.06 |  | 3.1e-3 |  | 1.2e-1 |  | 0.139 |  | 166.69 |  | 1.000 |  |  |  | C15(Dehydro): 100; C21(Dehydro): 100 |  |  |  |  |  |  |  | 0.14 |  | 1 |  | Sequest HT (3) |  | 3 |  | 656.02502 |  | -0.37 |  | 52.4746 |  | 16332 |  | 16332 |  | TRINITY\_DN77\_c0\_g1\_i1.p1\_ORF2; TRINITY\_DN1399\_c0\_g1\_i1.p1\_ORF1; TRINITY\_DN77\_c0\_g1\_i1.p1\_ORF1 |  | Image |  | Peak List |  |
|  | | | | | | | | | | | | | | | | | | | | | | | | | | | | | | | | | | | | | | | | | | | | | | | | | | | | | |  |
|  | High |  | [R].GLLSGILGAGKHIVcGLSGLc.[-] |  | HCD |  | C15(Dehydro); C21(Dehydro) |  | 2.63 |  | 3.1e-3 |  | 9.4e-2 |  | 0.196 |  | 203.30 |  | 1.000 |  |  |  | C15(Dehydro): 100; C21(Dehydro): 100 |  |  |  |  |  |  |  | 0.10 |  | 1 |  | Sequest HT (3) |  | 3 |  | 656.02600 |  | 1.12 |  | 52.7547 |  | 16433 |  | 16433 |  | TRINITY\_DN77\_c0\_g1\_i1.p1\_ORF2; TRINITY\_DN1399\_c0\_g1\_i1.p1\_ORF1; TRINITY\_DN77\_c0\_g1\_i1.p1\_ORF1 |  | Image |  | Peak List |  |
|  | | | | | | | | | | | | | | | | | | | | | | | | | | | | | | | | | | | | | | | | | | | | | | | | | | | | | |  |
|  | High |  | [R].GLLSGILGAGKHIVcGLSGLc.[-] |  | HCD |  | C15(Dehydro); C21(Dehydro) |  | 3.64 |  | 1.3e-3 |  | 2.0e-3 |  | 1.05 |  | 379.00 |  | 1.000 |  |  |  | C15(Dehydro): 100; C21(Dehydro): 100 |  |  |  |  |  |  |  | 0.14 |  | 1 |  | Sequest HT (3) |  | 2 |  | 983.53461 |  | 0.35 |  | 52.7568 |  | 16434 |  | 16434 |  | TRINITY\_DN77\_c0\_g1\_i1.p1\_ORF2; TRINITY\_DN1399\_c0\_g1\_i1.p1\_ORF1; TRINITY\_DN77\_c0\_g1\_i1.p1\_ORF1 |  | Image |  | Peak List |  |
|  | | | | | | | | | | | | | | | | | | | | | | | | | | | | | | | | | | | | | | | | | | | | | | | | | | | | | |  |
|  | High |  | [R].GLLSGILGAGKHIVcGLSGLc.[-] |  | HCD |  | C15(Dehydro); C21(Dehydro) |  | 3.08 |  | 1.3e-3 |  | 1.6e-2 |  | 0.593 |  | 302.34 |  | 1.000 |  |  |  | C15(Dehydro): 100; C21(Dehydro): 100 |  |  |  |  |  |  |  | 0.15 |  | 1 |  | Sequest HT (3) |  | 2 |  | 983.53418 |  | -0.08 |  | 52.9057 |  | 16490 |  | 16490 |  | TRINITY\_DN77\_c0\_g1\_i1.p1\_ORF2; TRINITY\_DN1399\_c0\_g1\_i1.p1\_ORF1; TRINITY\_DN77\_c0\_g1\_i1.p1\_ORF1 |  | Image |  | Peak List |  |
|  | | | | | | | | | | | | | | | | | | | | | | | | | | | | | | | | | | | | | | | | | | | | | | | | | | | | | |  |
|  | High |  | [R].GLLSGILGAGKHIVcGLSGLc.[-] |  | HCD |  | C15(Dehydro); C21(Dehydro) |  | 3.33 |  | 1.3e-3 |  | 6.7e-3 |  | 0.778 |  | 337.87 |  | 1.000 |  |  |  | C15(Dehydro): 100; C21(Dehydro): 100 |  |  |  |  |  |  |  | 0.12 |  | 1 |  | Sequest HT (3) |  | 2 |  | 983.53424 |  | -0.02 |  | 53.0465 |  | 16541 |  | 16541 |  | TRINITY\_DN77\_c0\_g1\_i1.p1\_ORF2; TRINITY\_DN1399\_c0\_g1\_i1.p1\_ORF1; TRINITY\_DN77\_c0\_g1\_i1.p1\_ORF1 |  | Image |  | Peak List |  |
|  | | | | | | | | | | | | | | | | | | | | | | | | | | | | | | | | | | | | | | | | | | | | | | | | | | | | | |  |
|  | High |  | [R].GLLSGILGAGKHIVcGLSGLc.[-] |  | HCD |  | C15(Dehydro); C21(Dehydro) |  | 1.60 |  | 3.1e-3 |  | 1.3e-1 |  | 0.123 |  | 172.19 |  | 1.000 |  |  |  | C15(Dehydro): 100; C21(Dehydro): 100 |  |  |  |  |  |  |  | 0.09 |  | 1 |  | Sequest HT (3) |  | 2 |  | 983.53583 |  | 1.59 |  | 54.8330 |  | 17152 |  | 17152 |  | TRINITY\_DN77\_c0\_g1\_i1.p1\_ORF2; TRINITY\_DN1399\_c0\_g1\_i1.p1\_ORF1; TRINITY\_DN77\_c0\_g1\_i1.p1\_ORF1 |  | Image |  | Peak List |  |
|  | | | | | | | | | | | | | | | | | | | | | | | | | | | | | | | | | | | | | | | | | | | | | | | | | | | | | |  |
|  | High |  | [R].GLLSGILGAGKHIVcGLSGLc.[-] |  | HCD |  | C15(Dehydro); C21(Dehydro) |  | 2.51 |  | 1.3e-3 |  | 2.3e-2 |  | 0.513 |  | 234.68 |  | 1.000 |  |  |  | C15(Dehydro): 100; C21(Dehydro): 100 |  |  |  |  |  |  |  | 0.11 |  | 1 |  | Sequest HT (3) |  | 2 |  | 983.53595 |  | 1.72 |  | 62.2126 |  | 19543 |  | 19543 |  | TRINITY\_DN77\_c0\_g1\_i1.p1\_ORF2; TRINITY\_DN1399\_c0\_g1\_i1.p1\_ORF1; TRINITY\_DN77\_c0\_g1\_i1.p1\_ORF1 |  | Image |  | Peak List |  |
|  | | | | | | | | | | | | | | | | | | | | | | | | | | | | | | | | | | | | | | | | | | | | | | | | | | | | | |  |

  
Top
  

### Search Summary

Result name: Search\_file
  
Description: Processing workflow for precursor-based quantification, using SequestHT with Multi Peptide Search and Percolator validation. Specify the FASTA database, labels used, and any additional modifications.
  
Workflow based on template: PWF\_QE\_Precursor\_Quan\_and\_LFQ\_MPS\_SequestHT\_Percolator
  
Creation date: 6/12/2022 12:17:29 PM
  
Created with Discoverer version: 2.5.0.400
  
  
------------------------------------------------------------------
  
The workflow tree:
  
------------------------------------------------------------------
  
  
    |-(6) Spectrum Files
  
        |-(5) Minora Feature Detector
  
        |-(1) Spectrum Selector
  
            |-(2) Precursor Detector
  
                |-(3) Sequest HT
  
                    |-(4) Percolator
  
                        |-(7) IMP-ptmRS
  
Result name: Search\_file
  
Description: Processing workflow for precursor-based quantification, using SequestHT with Multi Peptide Search and Percolator validation. Specify the FASTA database, labels used, and any additional modifications.
  
Workflow based on template: PWF\_QE\_Precursor\_Quan\_and\_LFQ\_MPS\_SequestHT\_Percolator
  
Creation date: 6/12/2022 12:17:29 PM
  
------------------------------------------------------------------
  
The workflow tree:
  
------------------------------------------------------------------
  
  
    |-(6) Spectrum Files
  
        |-(5) Minora Feature Detector
  
        |-(1) Spectrum Selector
  
            |-(2) Precursor Detector
  
                |-(3) Sequest HT
  
                    |-(4) Percolator
  
                        |-(7) IMP-ptmRS
  
------------------------------------------------------------------
  
Processing node 6: Spectrum Files
  
------------------------------------------------------------------
  
Input Data:
  
- File Name(s): Z:\����\Wuzhipan.raw
  
  
------------------------------------------------------------------
  
Processing node 5: Minora Feature Detector
  
------------------------------------------------------------------
  
1. Peak & Feature Detection:
  
- Min. Trace Length: 5
  
- S/N Threshold: 1
  
- Max. ��RT of Isotope Pattern Multiplets [min]: 0.2
  
  
2. Feature to ID Linking:
  
- PSM Confidence At Least: High
  
  
------------------------------------------------------------------
  
Processing node 1: Spectrum Selector
  
------------------------------------------------------------------
  
1. General Settings:
  
- Precursor Selection: Use MS1 Precursor
  
- Use Isotope Pattern in Precursor Reevaluation: True
  
- Provide Profile Spectra: Automatic
  
  
2. Spectrum Properties Filter:
  
- Lower RT Limit: 0
  
- Upper RT Limit: 0
  
- First Scan: 0
  
- Last Scan: 0
  
- Lowest Charge State: 1
  
- Highest Charge State: 0
  
- Min. Precursor Mass: 350 Da
  
- Max. Precursor Mass: 6000 Da
  
- Total Intensity Threshold: 0
  
- Minimum Peak Count: 1
  
  
3. Scan Event Filters:
  
- Mass Analyzer: Is FTMS
  
- MS Order: Is Not MS1
  
- Activation Type: Is HCD
  
- Min. Collision Energy: 0
  
- Max. Collision Energy: 1000
  
- Scan Type: Is Full
  
- Polarity Mode: Is +
  
  
4. Peak Filters:
  
- S/N Threshold (FT-only): 1.5
  
  
5. Replacements for Unrecognized Properties:
  
- Unrecognized Charge Replacements: Automatic
  
- Unrecognized Mass Analyzer Replacements: ITMS
  
- Unrecognized MS Order Replacements: MS2
  
- Unrecognized Activation Type Replacements: CID
  
- Unrecognized Polarity Replacements: +
  
- Unrecognized MS Resolution@200 Replacements: 60000
  
- Unrecognized MSn Resolution@200 Replacements: 30000
  
  
6. Precursor Pattern Extraction:
  
- Precursor Clipping Range Before: 2.5 Da
  
- Precursor Clipping Range After: 5.5 Da
  
  
------------------------------------------------------------------
  
Processing node 2: Precursor Detector
  
------------------------------------------------------------------
  
1. General:
  
- S/N Threshold: 1.5
  
- S/N Threshold for most abundant peak: 1.5
  
  
------------------------------------------------------------------
  
Processing node 3: Sequest HT
  
------------------------------------------------------------------
  
1. Input Data:
  
- Protein Database: Search\_file.fasta
  
- Enzyme Name: No-Enzyme (Unspecific)
  
- Max. Missed Cleavage Sites: 2
  
- Min. Peptide Length: 6
  
- Max. Peptide Length: 144
  
- Max. Number of Peptides Reported: 10
  
  
2. Tolerances:
  
- Precursor Mass Tolerance: 3 ppm
  
- Fragment Mass Tolerance: 0.02 Da
  
- Use Average Precursor Mass: False
  
- Use Average Fragment Mass: False
  
  
3. Spectrum Matching:
  
- Use Neutral Loss a Ions: True
  
- Use Neutral Loss b Ions: True
  
- Use Neutral Loss y Ions: True
  
- Use Flanking Ions: True
  
- Weight of a Ions: 0
  
- Weight of b Ions: 1
  
- Weight of c Ions: 0
  
- Weight of x Ions: 0
  
- Weight of y Ions: 1
  
- Weight of z Ions: 0
  
  
4. Dynamic Modifications:
  
- Max. Equal Modifications Per Peptide: 2
  
- Max. Dynamic Modifications Per Peptide: 4
  
- 1. Dynamic Modification: Oxidation / +15.995 Da (M)
  
- 2. Dynamic Modification: Deamidated / +0.984 Da (N)
  
- 3. Dynamic Modification: Dehydro / -1.008 Da (C)
  
  
6. Dynamic Modifications (protein terminus):
  
- 1. N-Terminal Modification: Acetyl / +42.011 Da (N-Terminus)
  
- 2. N-Terminal Modification: Met-loss / -131.040 Da (M)
  
- 3. N-Terminal Modification: Met-loss+Acetyl / -89.030 Da (M)
  
  
------------------------------------------------------------------
  
Processing node 4: Percolator
  
------------------------------------------------------------------
  
1. Target/Decoy Strategy:
  
- Target/Decoy Selection: Separate
  
- Validation based on: q-Value
  
  
2. Input Data:
  
- Maximum Delta Cn: 0.05
  
- Maximum Rank: 0
  
  
3. FDR Targets:
  
- Target FDR (Strict): 0.01
  
- Target FDR (Relaxed): 0.05
  
  
------------------------------------------------------------------
  
Processing node 7: IMP-ptmRS
  
------------------------------------------------------------------
  
1. Scoring:
  
- PhosphoRS Mode: False
  
- Report Only PTMs: False
  
- Use Diagnostic Ions: True
  
- Use Fragment Mass Tolerance of Search Node: True
  
- Fragment Mass Tolerance: 0.5 Da
  
- Consider neutral loss peaks for CID, HCD and EThcD: Automatic
  
- Maximum Peak Depth: 8
  
- Use a mass accuracy correction: True
  
  
2. Performance:
  
- Maximum Number of Position Isoforms: 25
  
- Maximum PTMs per peptide: 10
  
================================================================================
  
  
Processing details:
  
  
06/12/2022 05:08 PM Job Execution: Processing D:\OG\_skin\Search\_file.msf
  
06/12/2022 05:08 PM (1) Spectrum Selector: Profile spectra are not sent.
  
06/12/2022 05:08 PM (1) Spectrum Selector: Reading from file 1 of 1 F1: Z:\����\Wuzhipan.raw (20137 spectra total)
  
06/12/2022 05:08 PM (3) Sequest HT: Sequence Database: Search\_file.fasta
  
06/12/2022 05:09 PM (1) Spectrum Selector: Sent 13335 spectra from file F1.
  
06/12/2022 05:09 PM (1) Spectrum Selector: Sent 13335 spectra from 1 files (processing time: 31 s).
  
06/12/2022 05:09 PM (1) Spectrum Selector: -- Total execution of Spectrum Selector (1) took 1 min 16 s --
  
06/12/2022 05:09 PM (2) Precursor Detector: -- Total execution of Precursor Detector (2) took 15.7 s --
  
06/12/2022 05:09 PM (3) Sequest HT: Storing spectra took 27.8 s.
  
06/12/2022 05:09 PM (3) Sequest HT: An index for the FASTA database is not going to be created automatically, because the cleavage specificity isn't full enzymatic.   
You can manually create this index with the FASTA index manager.
  
06/12/2022 05:09 PM (3) Sequest HT: Start Sequest HT target search for 13335 spectra (23708 precursors)...
  
06/12/2022 05:10 PM (3) Sequest HT: Ise (2.0.0.24, x64) started at 6/12/2022 5:10:00 PM on MASS-PC (x64) [8 CPUs] running Microsoft Windows NT 6.1.7601 Service Pack 1 (64bit) [.NET: 4.0.30319.42000]
  
06/12/2022 05:10 PM (3) Sequest HT: Workload level: #parallel tasks: 8
  
06/12/2022 05:10 PM (3) Sequest HT: Workload level: #spectra loaded and processed at once: 8000
  
06/12/2022 05:10 PM (3) Sequest HT: In-memory search is performed
  
06/12/2022 07:39 PM (3) Sequest HT: 88 spectra were not used for search.
  
06/12/2022 07:39 PM (3) Sequest HT: Average search time per spectrum was 380.7 ms.
  
06/12/2022 07:39 PM (3) Sequest HT: Performing target search took 2 h 29 min.
  
06/12/2022 07:40 PM (3) Sequest HT: Stored 42583 PSMs for 23620 spectra
  
06/12/2022 07:40 PM (3) Sequest HT: Discarded 61113 peptide(s) that did not match the conditions for protein terminal modifications.
  
06/12/2022 07:40 PM (3) Sequest HT: Reading search results took 4.3 s.
  
06/12/2022 07:40 PM (3) Sequest HT: Saving results took 3.6 s.
  
06/12/2022 07:40 PM (3) Sequest HT: Saving proteins took 4.9 s.
  
06/12/2022 07:40 PM (3) Sequest HT: Reading results took 13.1 s.
  
06/12/2022 07:40 PM (3) Sequest HT: An index for the FASTA database is not going to be created automatically, because the cleavage specificity isn't full enzymatic.   
You can manually create this index with the FASTA index manager.
  
06/12/2022 07:40 PM (3) Sequest HT: Start Sequest HT decoy search for 13335 spectra (23708 precursors)...
  
06/12/2022 07:40 PM (3) Sequest HT: Ise (2.0.0.24, x64) started at 6/12/2022 7:40:08 PM on MASS-PC (x64) [8 CPUs] running Microsoft Windows NT 6.1.7601 Service Pack 1 (64bit) [.NET: 4.0.30319.42000]
  
06/12/2022 07:40 PM (3) Sequest HT: Workload level: #parallel tasks: 8
  
06/12/2022 07:40 PM (3) Sequest HT: Workload level: #spectra loaded and processed at once: 8000
  
06/12/2022 07:40 PM (3) Sequest HT: In-memory search is performed
  
06/12/2022 10:08 PM (3) Sequest HT: 88 spectra were not used for search.
  
06/12/2022 10:08 PM (3) Sequest HT: Average search time per spectrum was 376.4 ms.
  
06/12/2022 10:08 PM (3) Sequest HT: Performing decoy search took 2 h 28 min.
  
06/12/2022 10:08 PM (3) Sequest HT: Stored 85643 decoy PSMs for 23620 spectra
  
06/12/2022 10:08 PM (3) Sequest HT: Discarded 63590 peptide(s) that did not match the conditions for protein terminal modifications.
  
06/12/2022 10:08 PM (3) Sequest HT: Reading search results took 4.1 s.
  
06/12/2022 10:08 PM (3) Sequest HT: Saving results took 3.4 s.
  
06/12/2022 10:08 PM (3) Sequest HT: Saving proteins took 4.8 s.
  
06/12/2022 10:08 PM (3) Sequest HT: Reading results took 12.5 s.
  
06/12/2022 10:08 PM (3) Sequest HT: Finalizing search results...
  
06/12/2022 10:08 PM (3) Sequest HT: -- Total search time was 4 h 58 min --
  
06/12/2022 10:10 PM (4) Percolator: Creating percolator input file with results from Sequest HT (3) took 1 min 50 s.
  
06/12/2022 10:10 PM (4) Percolator: The input file contains 39501 PSMs, 39804 decoy PSMs and 32 features.
  
06/12/2022 10:10 PM (4) Percolator: Generated percolator input for separate mode with mix max algorithm
  
06/12/2022 10:10 PM (4) Percolator: Percolator version 3.05.0, Build Date May 18 2020 02:35:01
  
06/12/2022 10:10 PM (4) Percolator: Copyright (c) 2006-9 University of Washington. All rights reserved.
  
06/12/2022 10:10 PM (4) Percolator: Written by Lukas Käll (lukall@u.washington.edu) in the
  
06/12/2022 10:10 PM (4) Percolator: Department of Genome Sciences at the University of Washington.
  
06/12/2022 10:10 PM (4) Percolator: Issued command:
  
06/12/2022 10:10 PM (4) Percolator: C:\Program Files\Thermo\Proteome Discoverer 2.5\Tools\Percolator\win\percolator.exe --num-threads 6 -s -X C:\ProgramData\Thermo\Proteome Discoverer 2.5\Scratch\Job44\Percol(4)\output3.xml -N 0 -Z -I auto C:\ProgramData\Thermo\Proteome Discoverer 2.5\Scratch\Job44\Percol(4)\input3.tab
  
06/12/2022 10:10 PM (4) Percolator: Started Sun Jun 12 22:10:48 2022
  
06/12/2022 10:10 PM (4) Percolator: Hyperparameters: selectionFdr=0.01, Cpos=0, Cneg=0, maxNiter=10
  
06/12/2022 10:10 PM (4) Percolator: Reading tab-delimited input from datafile C:\ProgramData\Thermo\Proteome Discoverer 2.5\Scratch\Job44\Percol(4)\input3.tab
  
06/12/2022 10:10 PM (4) Percolator: Features:
  
06/12/2022 10:10 PM (4) Percolator: XCorr Delta Cn From Second PSM Binomial Score Isolation Interference [%] MH+ [Da] Delta Mass [Da] Delta Mass [ppm] Absolute Delta Mass [Da] Absolute Delta Mass [ppm] Peptide Length Is z=1 Is z=2 Is z=3 Is z=4 Is z=5 Is z>5 # Missed Cleavages Log Peptides Matched Log Total Intensity Fraction Matched Intensity [%] Fragment Coverage Series A, B, C [%] Fragment Coverage Series X, Y, Z [%] Log Matched Fragment Series Intensities A, B, C Log Matched Fragment Series Intensities X, Y, Z Longest Sequence Series A, B, C Longest Sequence Series X, Y, Z IQR Fragment Delta Mass [Da] IQR Fragment Delta Mass [ppm] Mean Fragment Delta Mass [Da] Mean Fragment Delta Mass [ppm] Mean Absolute Fragment Delta Mass [Da] Mean Absolute Fragment Delta Mass [ppm]
  
06/12/2022 10:10 PM (4) Percolator: Found 79305 PSMs
  
06/12/2022 10:10 PM (4) Percolator: Separate target and decoy search inputs detected, using mix-max method.
  
06/12/2022 10:10 PM (4) Percolator: Train/test set contains 39501 positives and 39804 negatives, size ratio=0.992388 and pi0=1
  
06/12/2022 10:10 PM (4) Percolator: Selecting Cpos by cross-validation.
  
06/12/2022 10:10 PM (4) Percolator: Selecting Cneg by cross-validation.
  
06/12/2022 10:10 PM (4) Percolator: Split 1: Selected feature 3 as initial direction. Could separate 566 training set positives with q<0.01 in that direction.
  
06/12/2022 10:10 PM (4) Percolator: Split 2: Selected feature 3 as initial direction. Could separate 629 training set positives with q<0.01 in that direction.
  
06/12/2022 10:10 PM (4) Percolator: Split 3: Selected feature 3 as initial direction. Could separate 539 training set positives with q<0.01 in that direction.
  
06/12/2022 10:10 PM (4) Percolator: Found 793 test set positives with q<0.01 in initial direction
  
06/12/2022 10:10 PM (4) Percolator: Reading in data and feature calculation took 2.587 cpu seconds or 2 seconds wall clock time.
  
06/12/2022 10:10 PM (4) Percolator: ---Training with Cpos selected by cross validation, Cneg selected by cross validation, initial\_fdr=0.01, fdr=0.01
  
06/12/2022 10:10 PM (4) Percolator: Iteration 1: Estimated 1194 PSMs with q<0.01
  
06/12/2022 10:10 PM (4) Percolator: Iteration 2: Estimated 1206 PSMs with q<0.01
  
06/12/2022 10:10 PM (4) Percolator: Iteration 3: Estimated 1232 PSMs with q<0.01
  
06/12/2022 10:10 PM (4) Percolator: Iteration 4: Estimated 1238 PSMs with q<0.01
  
06/12/2022 10:10 PM (4) Percolator: Iteration 5: Estimated 1243 PSMs with q<0.01
  
06/12/2022 10:10 PM (4) Percolator: Iteration 6: Estimated 1239 PSMs with q<0.01
  
06/12/2022 10:10 PM (4) Percolator: Iteration 7: Estimated 1241 PSMs with q<0.01
  
06/12/2022 10:10 PM (4) Percolator: Iteration 8: Estimated 1239 PSMs with q<0.01
  
06/12/2022 10:10 PM (4) Percolator: Iteration 9: Estimated 1244 PSMs with q<0.01
  
06/12/2022 10:10 PM (4) Percolator: Iteration 10: Estimated 1244 PSMs with q<0.01
  
06/12/2022 10:10 PM (4) Percolator: Learned normalized SVM weights for the 3 cross-validation splits:
  
06/12/2022 10:10 PM (4) Percolator: Split1 Split2 Split3 FeatureName
  
06/12/2022 10:10 PM (4) Percolator: -1.0750 -0.4192 -0.8600 XCorr
  
06/12/2022 10:10 PM (4) Percolator: 0.2035 0.2688 0.6351 Delta Cn From Second PSM
  
06/12/2022 10:10 PM (4) Percolator: 2.8173 1.8742 3.1576 Binomial Score
  
06/12/2022 10:10 PM (4) Percolator: 0.2843 0.3912 0.1959 Isolation Interference [%]
  
06/12/2022 10:10 PM (4) Percolator: -2.4447 -2.7830 -3.5464 MH+ [Da]
  
06/12/2022 10:10 PM (4) Percolator: 0.0325 0.4030 0.8580 Delta Mass [Da]
  
06/12/2022 10:10 PM (4) Percolator: 0.4488 -0.7258 -1.0373 Delta Mass [ppm]
  
06/12/2022 10:10 PM (4) Percolator: -0.8409 1.3454 -2.0875 Absolute Delta Mass [Da]
  
06/12/2022 10:10 PM (4) Percolator: 0.1402 -1.5835 1.4140 Absolute Delta Mass [ppm]
  
06/12/2022 10:10 PM (4) Percolator: 4.1077 3.2871 6.3325 Peptide Length
  
06/12/2022 10:10 PM (4) Percolator: -0.6336 -0.1158 -0.6970 Is z=1
  
06/12/2022 10:10 PM (4) Percolator: -0.5332 -0.1256 -0.3783 Is z=2
  
06/12/2022 10:10 PM (4) Percolator: -0.0994 -0.1298 -0.0798 Is z=3
  
06/12/2022 10:10 PM (4) Percolator: 0.4398 0.1491 0.7513 Is z=4
  
06/12/2022 10:10 PM (4) Percolator: 0.7683 0.3803 0.5057 Is z=5
  
06/12/2022 10:10 PM (4) Percolator: 0.0368 -0.3494 -0.9587 Is z>5
  
06/12/2022 10:10 PM (4) Percolator: 0.0000 0.0000 0.0000 # Missed Cleavages
  
06/12/2022 10:10 PM (4) Percolator: 0.0735 0.1530 0.9670 Log Peptides Matched
  
06/12/2022 10:10 PM (4) Percolator: -0.6802 -0.7404 -2.4397 Log Total Intensity
  
06/12/2022 10:10 PM (4) Percolator: 0.7963 0.5459 0.7769 Fraction Matched Intensity [%]
  
06/12/2022 10:10 PM (4) Percolator: 0.3671 0.3389 0.6283 Fragment Coverage Series A, B, C [%]
  
06/12/2022 10:10 PM (4) Percolator: 0.4341 -0.0282 1.1330 Fragment Coverage Series X, Y, Z [%]
  
06/12/2022 10:10 PM (4) Percolator: -0.6573 -0.4172 -1.1616 Log Matched Fragment Series Intensities A, B, C
  
06/12/2022 10:10 PM (4) Percolator: 0.7180 0.9815 1.3943 Log Matched Fragment Series Intensities X, Y, Z
  
06/12/2022 10:10 PM (4) Percolator: 0.1215 0.2000 0.7661 Longest Sequence Series A, B, C
  
06/12/2022 10:10 PM (4) Percolator: 0.5315 0.7450 0.5176 Longest Sequence Series X, Y, Z
  
06/12/2022 10:10 PM (4) Percolator: 1.6107 1.1587 0.2170 IQR Fragment Delta Mass [Da]
  
06/12/2022 10:10 PM (4) Percolator: -1.6927 -0.6761 -0.0172 IQR Fragment Delta Mass [ppm]
  
06/12/2022 10:10 PM (4) Percolator: -1.0496 -0.7332 -1.2215 Mean Fragment Delta Mass [Da]
  
06/12/2022 10:10 PM (4) Percolator: 0.8937 0.7233 -0.1026 Mean Fragment Delta Mass [ppm]
  
06/12/2022 10:10 PM (4) Percolator: -3.1904 -1.8440 -2.7840 Mean Absolute Fragment Delta Mass [Da]
  
06/12/2022 10:10 PM (4) Percolator: -0.4012 -2.3496 -1.4859 Mean Absolute Fragment Delta Mass [ppm]
  
06/12/2022 10:10 PM (4) Percolator: -10.8850 -9.2962 -15.2669 m0
  
06/12/2022 10:10 PM (4) Percolator: Found 1045 test set PSMs with q<0.01.
  
06/12/2022 10:11 PM (4) Percolator: Tossing out "redundant" PSMs keeping only the best scoring PSM for each unique peptide.
  
06/12/2022 10:11 PM (4) Percolator: Selecting pi\_0=0.993665
  
06/12/2022 10:11 PM (4) Percolator: Calculating q values.
  
06/12/2022 10:11 PM (4) Percolator: New pi\_0 estimate on final list yields 152 target peptides with q<0.01.
  
06/12/2022 10:11 PM (4) Percolator: Calculating posterior error probabilities (PEPs).
  
06/12/2022 10:11 PM (4) Percolator: Processing took 16.93 cpu seconds or 13 seconds wall clock time.
  
06/12/2022 10:11 PM (4) Percolator: PSMId score q-value posterior\_error\_prob peptide proteinIds
  
06/12/2022 10:11 PM (4) Percolator: 1131/9 high confident target/decoy peptides were found for Sequest HT (3).
  
06/12/2022 10:11 PM (4) Percolator: 226/59 medium confident target/decoy peptides were found for Sequest HT (3).
  
06/12/2022 10:11 PM (4) Percolator: -- Total execution of Percolator (64Bit) for Sequest HT (3) took 2 min 18 s --
  
06/12/2022 10:11 PM (5) Minora Feature Detector: Start detecting lcmspeaks...
  
06/12/2022 10:11 PM (5) Minora Feature Detector: Running 1 jobs with 1 jobs in parallel.
  
06/12/2022 10:11 PM (5) Minora Feature Detector: Start F1 with 'FTMS + p NSI Full ms [350.00-1800.00]'
  
06/12/2022 10:14 PM (5) Minora Feature Detector: Detecting LcmsPeaks took 2 min 59 s
  
06/12/2022 10:14 PM (5) Minora Feature Detector: Start processing F1: 'Wuzhipan.raw'.
  
06/12/2022 10:14 PM (5) Minora Feature Detector: Importing peaks for F1 (scan filter: FTMS + p NSI Full ms [350.00-1800.00])...
  
06/12/2022 10:14 PM (5) Minora Feature Detector: F1: Found 333503 chromatographic peaks.
  
06/12/2022 10:14 PM (5) Minora Feature Detector: F1: Importing auto traces...
  
06/12/2022 10:14 PM (5) Minora Feature Detector: F1: Imported 235287 auto traces.
  
06/12/2022 10:14 PM (5) Minora Feature Detector: Importing peak to spectrum links...
  
06/12/2022 10:15 PM (5) Minora Feature Detector: Found 541 features (F1: 541)
  
06/12/2022 10:15 PM (5) Minora Feature Detector: Writing data took 97.9 ms.
  
06/12/2022 10:15 PM (5) Minora Feature Detector: Creating features for PSMs took 2.6 s.
  
06/12/2022 10:15 PM (5) Minora Feature Detector: F1: Unidentified feature detection took 34.6 s
  
06/12/2022 10:15 PM (5) Minora Feature Detector: Found 68787 unidentified features (F1 : 68787).
  
06/12/2022 10:15 PM (5) Minora Feature Detector: -- Total execution of Minora Feature Detector (5) took 4 min 37 s --
  
06/12/2022 10:15 PM (7) IMP-ptmRS: Starting IMP-ptmRS node 2.0, IMP-ptmRS 1.0.0.0 - based on phosphoRS3 Taus T. et al., J. Proteome Res. 2011, 10, 5354-62
  
06/12/2022 10:15 PM (7) IMP-ptmRS: More nodes and documentations can be found here http://ms.imp.ac.at/?goto=pd-nodes.
  
06/12/2022 10:15 PM (7) IMP-ptmRS: Scoring with ptmRS (1.0.0.0) for Sequest HT (3) with a fragment ion mass tolerance of 0.02 m/z allowing a maximum of 10 PTMs with neutral loss and 25 isoforms per peptide.
  
06/12/2022 10:15 PM (7) IMP-ptmRS: Use mass accuracy correction.
  
06/12/2022 10:15 PM (7) IMP-ptmRS: (7) Localizing 1xOxidation, elementTarget: M (delta mass: 15.9949, Neutral-losses:H(4) C O S - 63.998285)
  
06/12/2022 10:15 PM (7) IMP-ptmRS: (7) Localizing 1xDeamidated, elementTarget: N (delta mass: 0.9840, no neutral loss)
  
06/12/2022 10:15 PM (7) IMP-ptmRS: (7) Localizing 1xDehydro, elementTarget: C (delta mass: -1.0078, no neutral loss)
  
06/12/2022 10:15 PM (7) IMP-ptmRS: (7) FITs for CID\_CAD: b, y; FITs with NLs: no
  
06/12/2022 10:15 PM (7) IMP-ptmRS: (7) FITs for HCD: b, y; FITs with NLs: b, y
  
06/12/2022 10:15 PM (7) IMP-ptmRS: (7) FITs for ECD\_ETD: c, zRadical, zPrime; FITs with NLs: no
  
06/12/2022 10:15 PM (7) IMP-ptmRS: (7) FITs for EThcD: b, y, c, zRadical, zPrime; FITs with NLs: b, y
  
06/12/2022 10:15 PM (7) IMP-ptmRS: (7) Workload level: #spectra: 13335.
  
06/12/2022 10:15 PM (7) IMP-ptmRS: (7) Workload level: #spectra per package: 40000.
  
06/12/2022 10:15 PM (7) IMP-ptmRS: (7) Workload level: #parallel tasks: 8.
  
06/12/2022 10:15 PM (7) IMP-ptmRS: (7) Finished collecting spectra
  
06/12/2022 10:16 PM (7) IMP-ptmRS: Writing current ptmRS results to file. Puffer has following size 20983
  
06/12/2022 10:16 PM (7) IMP-ptmRS: -- Total execution of IMP-ptmRS (7) took 24.5 s --
  
06/12/2022 10:16 PM Job Execution: Finished D:\OG\_skin\Search\_file.msf
  
06/12/2022 10:16 PM Job Execution: ----- Total Job execution took: 5 h 7 min. -----
  
  
  
Top
